# Synthesis and Functionalization of Thiophosphonium Salts: A Divergent Approach to Access Thioether, Thioester, and Dithioester Derivatives

**DOI:** 10.1021/acs.orglett.3c02422

**Published:** 2023-08-23

**Authors:** Gurupada Hazra, Ahmad Masarwa

**Affiliations:** Institute of Chemistry, The Center for Nanoscience and Nanotechnology, and Casali Center for Applied Chemistry, The Hebrew University of Jerusalem, Jerusalem 9190401, Israel

## Abstract

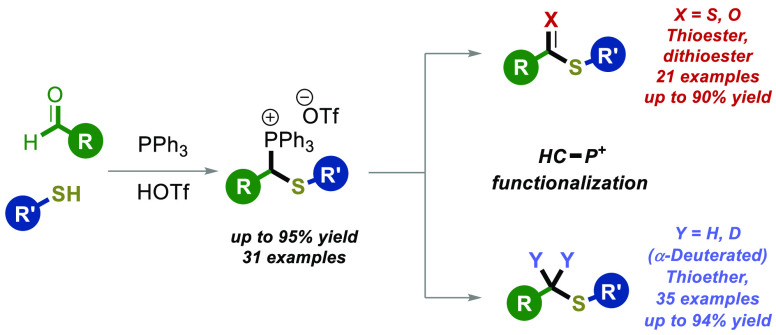

Herein, we report a straightforward practical method for efficiently obtaining a diverse range of thiophosphonium salts. This method involves the direct coupling of commercially available thiols and aldehydes with Ph_3_P and TfOH. The setup is simple and carried out in a metal-free manner. The synthetic utility of these salts is demonstrated through various examples of C–P bond functionalizations, enabling the synthesis of thioether, deuterated thioether, thioester, and dithioester derivatives. These products, which serve as valuable building blocks, are obtained in high yields.

Organophosphonium salts containing C–^+^P moieties,^1,2^ in particular organophosphorus-based Wittig salts, are among the most utilized reagents in organic synthesis for constructing the C–C double bond.^3^ In recent years, new methods for C–^+^P bond functionalization have been developed that use organophosphonium compounds in novel ways enabling new bond formation.^4−10^ For example, there are few examples of selective C–^+^PPh_3_ group functionalization to give products featuring new C–O, C–N, C–S, and C–C bonds.^4−8,10,11^ Despite these advancements, there are still limitations that must be addressed. Therefore, it is important to investigate additional methods for synthesizing new variants of organophosphonium species and their functionalizations. Such exploration is expected to greatly enhance the existing approaches for derivatizing noble organophosphonium salts and enable the creation of new connections.^10^

In this regard, recently, our research group has successfully described a versatile method for synthesizing benzhydryl triarylphosphonium salts (**VII**) through a convenient one-pot, regioselective four-component coupling reaction using readily available starting materials (Scheme 1A).^8^ The resulting benzhydryl phosphonium salt building blocks exhibited excellent utility, as demonstrated by their selective postfunctionalization of C-selective electrophilic benzylic C(sp^3^)–^+^PPh_3_ groups. This allowed for a range of transformations including aminations, thiolations, and arylations, leading to the creation of bioactive arylated scaffolds (Scheme 1A).^8^ In this method, benzhydrylamines, benzhydrylthioethers, and triarylmethanes, structural motifs that are present in many pharmaceuticals and agrochemicals, are respectively readily accessed.^8^ Furthermore, Chu^12,13^ and co-workers have developed an efficient metal-free difluoroalkylation reaction of these organophosphonium salts (**VII**) with difluoroenol silyl ethers (Scheme 1A).^12,13^

**Scheme 1:**
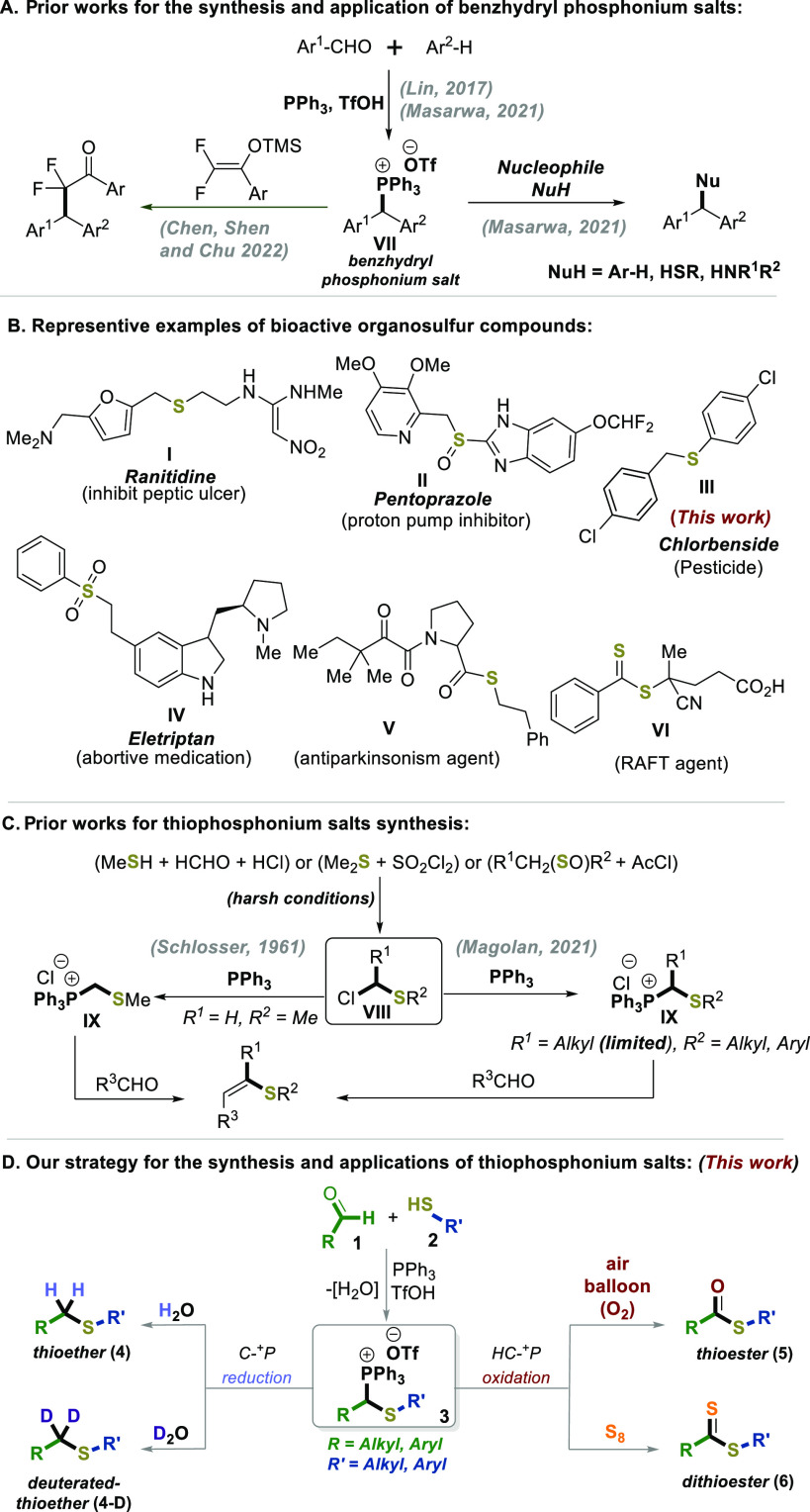
Overview of This Work

As part of our overarching strategies to synthesize a diverse range of thio-based bioactive compounds (**I–VI**), encompassing thioether, thioester, and dithioester derivatives, we recognized the potential of thiophosphonium salts as versatile core scaffolds for these molecules (Scheme 1B).^14−17^

However, the synthesis and application of thiophosphonium salts have been relatively uncommon, with few reports addressing their exploration (Scheme 1C). Notably, in 1961, Schlosser^18a^ described the use of α-chloro sulfides (**VIII**) as transient intermediates in the synthesis of thiophosphonium salts (Scheme 1C). More recently, in 2021, Magolan^18b^ utilized a similar approach, utilizing α-chloro sulfides (**VIII**) as a starting point for the synthesis of thiophosphonium salts **IX** (Scheme 1C).^18,19^ These salts (**IX**) were subsequently converted to vinyl sulfides,^18^ which further underwent transformations leading to the formation of ketones and indoles.^18,19^ It is important to highlight that the synthesis of α-chloro sulfides (**VIII**) necessitates the use of demanding reaction conditions.^18^ Moreover, it should be noted that the versatility of these reactions is predominantly limited to thioalkyl phosphonium salts (**IX**).^18^ Therefore, it is imperative that a complementary, general, milder, and diversifiable method for thiophosphonium salts will be developed.

With this goal in mind, we envisioned an operationally simple strategy to synthesize thiophosphonium salts **3** and convert them into valuable thio-based motifs (**4**–**6**, **III**) through the C(sp^3^)–^+^P transformation (Scheme 1D). In fact, drawing inspiration from our recent report^8^ and Lin’s^20^ work (Scheme 1A), our method involves a simple four-component reaction utilizing readily accessible and commercially available starting materials, i.e., aldehydes **1** and thiols **2** (Scheme 1D).

To test our hypothesis, we first treated *p*-anisaldehyde (**1a**) with thiophenol (**2a**), triphenylphosphine (PPh_3_), and triflic acid in CH_3_CN for 24 h at 45 °C to obtain the desired phosphonium salt **3a** in 97% isolated yield (Scheme 2A,B). Notably, no conversion was observed in the absence of either PPh_3_ or TfOH (for full details, see the Supporting Information (SI), pp S55–56).

**Scheme 2:**
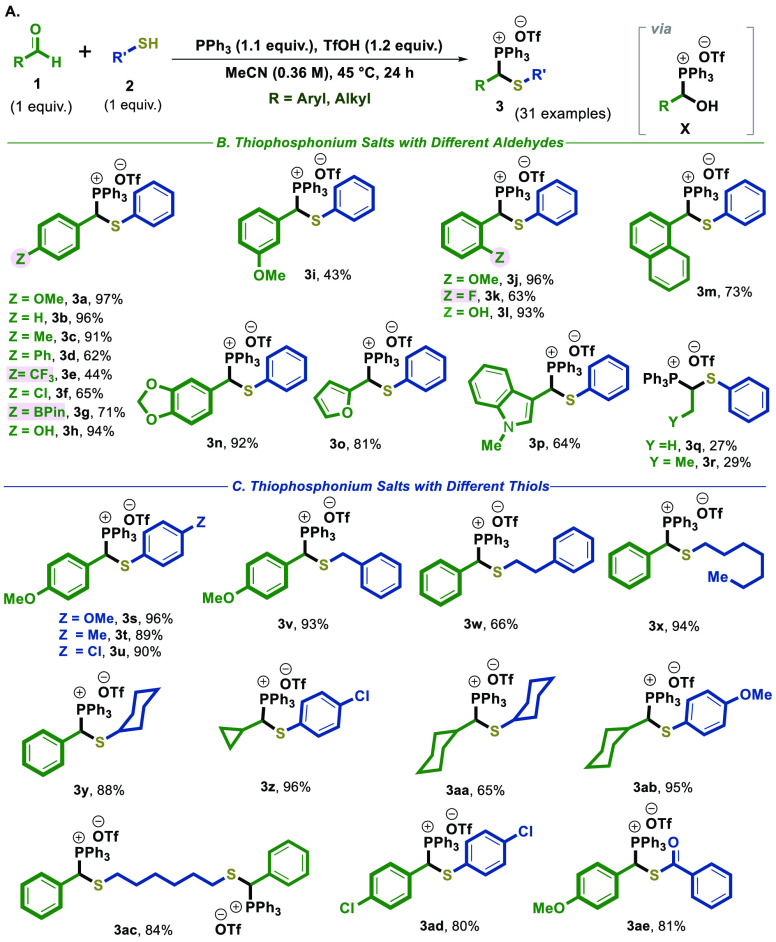
Synthesis of Thiophosphonium Salts (**3**) Reaction conditions: **1** (1.46 mmol), **2** (1.46 mmol), PPh_3_ (1.6 mmol), TfOH (1.75 mmol) in 4 mL of MeCN at 45 °C for 24 h. All yields of the reactions are isolated after washing with diethyl ether.

Next, a series of thiophosphonium salts (**3b**–**3u**) were synthesized from thiophenol **2a** coupling different aldehyde derivatives (**1**) bearing aromatic moieties which substituted with electron-donating groups (EDG; e.g., **1a**–**c**), electron-withdrawing groups (EWG; e.g., **1d**–**g**, **1k**), and phenolic derivatives (**1a**, **1h**, **1j**, **1l**, **1n**) as well as *N*-methylindole-based derivatives (**1p**). The reaction showed high functional group tolerance as evidenced by fluorine-containing (**3k**), chlorine-containing (**3f**), boron-containing (**3g**), and heteroarene-containing (**3o**, **3p**) substrates (Scheme 2). In addition, different types of aliphatic aldehydes both cyclic and acyclic (**3z–ab**, **3q**, **3r**, Scheme 2A–C) were explored. The reaction also demonstrated fruitful applicability to a diverse range of aromatic and aliphatic thiols, as well (**3a–3ad**, Scheme 2B,C). Importantly, the utilization of thiobenzoic acid (**2j**) has proven to be effective in the formation of the fascinating benzoylthiophosphonium salt (**3ae**).

In all cases, the phosphonium thio-alkylation-type reaction occurs selectively via a nucleophilic-substitution-type scenario of hydroxytriphenylphosphonium intermediate^8,21,22^**X** (for more details on this thio-alkylation-type mechanism, see the mechanistic studies in the SI, pp S54–55).^21,22^ Moreover, most of these products (**3**) were purified by simple precipitation, an additional advantage. The one-pot, four-component coupling approach can be carried out on a gram scale (see**3a**, 3.65 mmol, 95%, Scheme 2). Using the same approach, dithiophosphonium salt **3ac** was prepared from benzaldehyde (**1b**) and 1,6-hexanedithiol (**2i**).

We next explored the synthetic applications of these thiophosphonium products (**3**), and we aimed to demonstrate their synthetic utility in selective transformations of the C–^+^P bonds and the synthesis of bioactive chemicals. To this end, various representative applications were conceived for these valuable salts **3** as depicted in Scheme 3 and 4. For instance, we conducted selective reduction and oxidation protocols of the C–^+^P, enabling the synthesis of the valuable thioether, deuterated-thioether, thioester, and dithioester derivatives.^14,15,17,23−25^

**Scheme 3:**
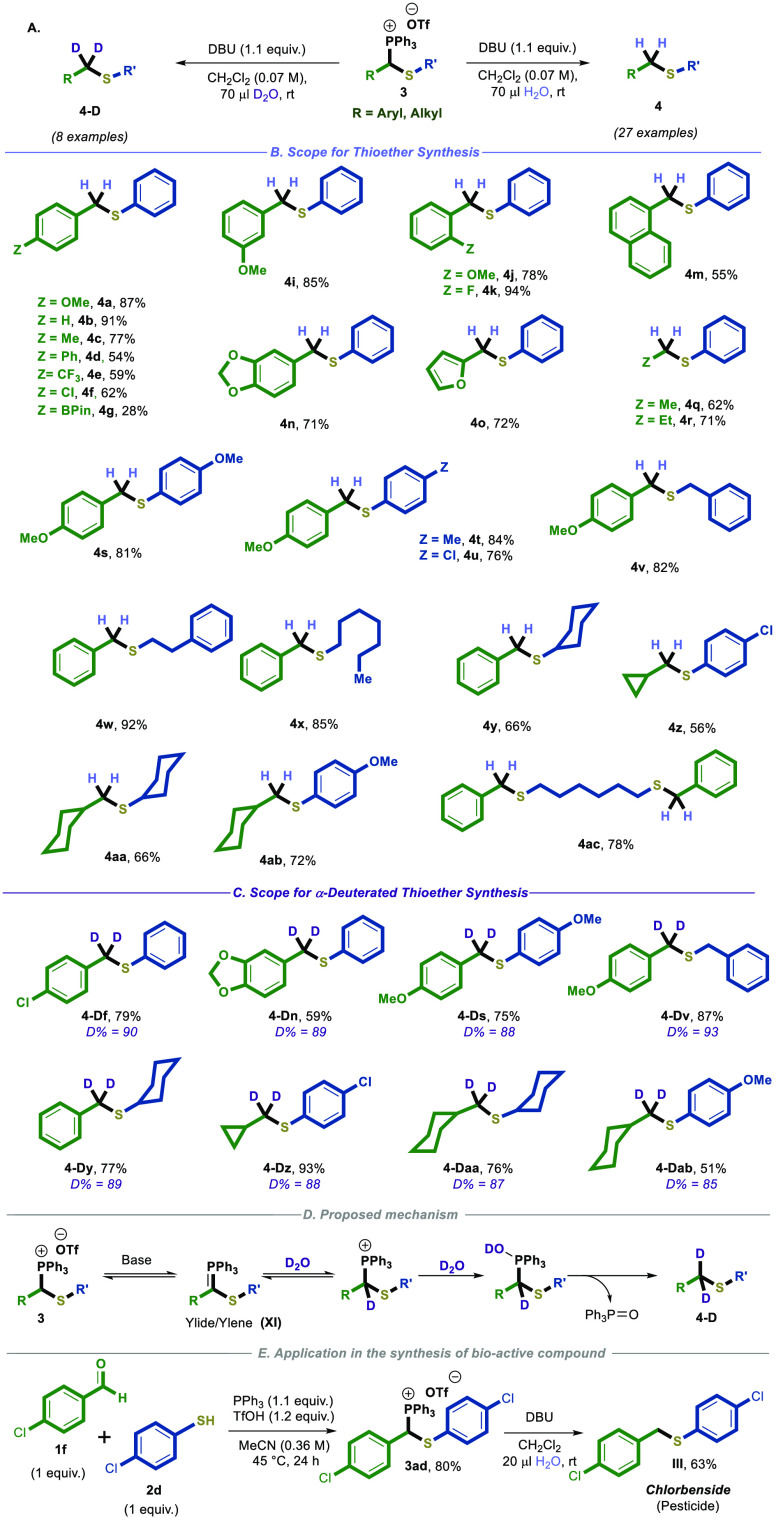
Reduction of Thiophosphonium Salts to Thioethers and α-Deuterated Thioethers Reaction conditions: **3** (0.2 mmol), **DBU** (0.22 mmol), and 70 μL of H_2_O or D_2_O in 3 mL of CH_2_Cl_2_ at room temperature. All yields of the reactions are isolated after column chromatography.

**Scheme 4:**
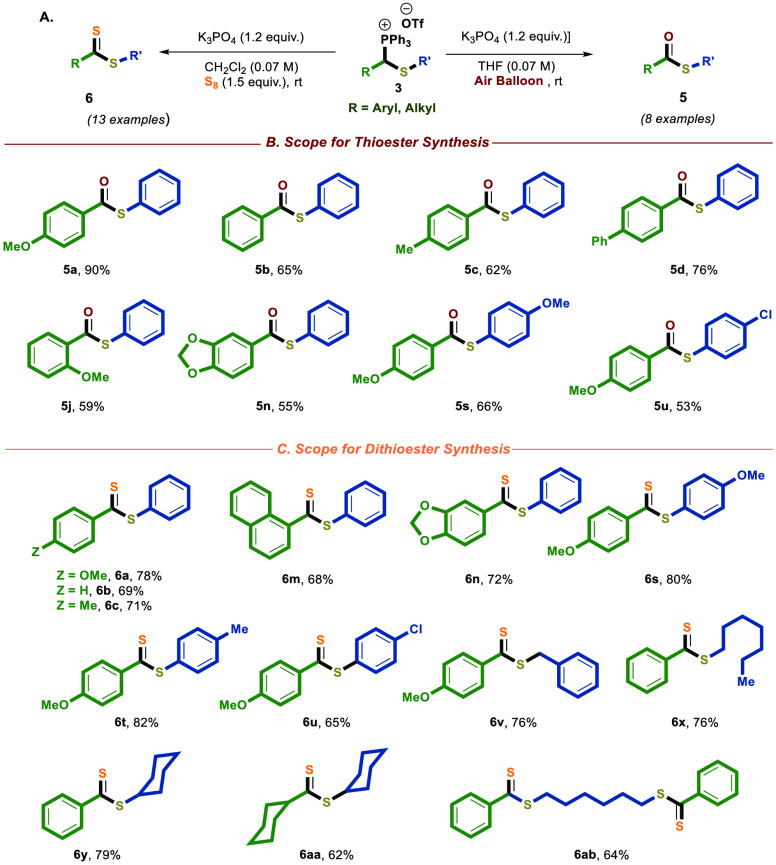
Oxidation of Thiophosphonium Salts to Thioesters and Dithioesters Reaction conditions: **3** (0.2 mmol), **K**_**3**_**PO4** (0.24 mmol), and air balloon in 3 mL of dry THF at room temperature (for thioester synthesis) or **3** (0.2 mmol), **K**_**3**_**PO**_**4**_ (0.24 mmol), and S_8_ (0.30 mmol) in 3 mL of dry CH_2_Cl_2_ at room temperature (for dithioester synthesis) All the yields of the reactions are isolated after column chromatography.

In this regard, a hydrolysis-based reduction protocol was developed for thiophosphonium salts **3** using H_2_O (Scheme 3A,B). In this method, we explored the reduction of a thiophosphonium salt, specifically utilizing **3a** as a standard salt for the reaction optimization. Various organic and inorganic bases were employed for the reaction, and the Supporting Information provides further details on these bases (see SI, pp S23–24). Ultimately, DBU was identified as the optimal base for the hydrolytic reduction of the thiophosphonium salt, leading to the formation of thioether **4a** in an isolated yield of 87% (Scheme 3A,B).

Subsequently, we successfully synthesized a variety of thioether derivatives by adapting the standard reaction conditions outlined in Scheme 3. The reaction exhibited a broad scope, encompassing aryls with substituents such as −OMe, −F, −Cl, −Ph, −Bpin, and −Me, in good yields. Notably, even alicyclic and cyclic aliphatic substituents on the thiol part yielded the desired thioether derivatives in good yields. Furthermore, replacing water with D_2_O provided a valuable opportunity to produce almost fully α-deuterated thioether (**4-D**), exhibiting excellent deuterium labeling (up to 93% D-incorporation) and good yields (Scheme 3C).

A possible mechanism for hydrolysis-based reduction pathway is proposed in Scheme 3D.^26−29^ On the basis of reports in the literature, this mechanistic process involves the formation of ylide/ylene **XI**, followed by protonation/deuteration of ylene **XI**. Subsequently, the cleavage of the C(sp^3^)–^+^P bond takes place through P-selective nucleophilic substitution, resulting in the liberation of triphenylphosphine oxide (Ph_3_P=O).^3,26−29^

Finally, we also successfully prepared the pesticide chemical chlorbenside^16^**III** from the commercially available 4-chlorobenzaldehyde **1f** and 4-chlorothiophenol **2d** in only two steps.^16,17^ This was achieved through the selective coupling reaction of **1f** and **2d** to generate thiophosphonium salt **3ad**, which was then reacted with DBU and water through a CH–^+^P group hydrolysis-based reduction reaction in a single operation (Scheme 3E).

Encouraged by these results of the reduction methodology, we contemplated the possibility of synthesizing important thioester^30^ and dithioester^23^ motifs using the thiophosphonium salt **3**.^24^ We imagined that this could be achieved by the generation of phosphorus ylide (**XI**) from salt **3** following by a direct oxidation of this ylide (**XI**).^31,32^

Our initial optimization efforts focused on the formation of thioester **5a** from thiophosphonium salt **3** and a base under air (a source of O_2_), via the corresponding ylide **XI**.^31,33^ After many experiments, the use of K_3_PO_4_ in dry THF was found to be the best reaction condition to obtain the thioester product **5a** with a yield of 90%.

Next, we explored the reaction’s scope; we employed identical reaction conditions. Consequently, thiophosphonium salt **3** effectively participated in the reaction, resulting in the formation of the desired product **5** with good yields, as depicted in Scheme 4A,B.

To further showcase the synthetic utility of salts **3**, we developed their transformation into dithioesters **6** (Scheme 4A,C).^15^ To this end, we employed basic reaction conditions to treat thiophosphonium salt **3** with elemental sulfur (S_8_).^32,34^ We conducted a thorough exploration of the optimal reaction conditions and determined that employing dry CH_2_Cl_2_ and K_3_PO_4_ as the standard reaction conditions resulted in the highest yield of dithioester **6a**, with approximately 78% yield (for more details, see the Supporting Information). Additionally, we showed that various thiophosphonium salts **3** could tolerate these reaction conditions, leading to the formation of the desired dithioester **6** with good yields, as depicted in Scheme 4C. Of note, a possible mechanism for the oxidation pathways is proposed in the SI (see SI, p S59).^33,34b,34c^

In conclusion, we have developed a simple and broadly straightforward protocol for the direct synthesis of a diverse range of thiophosphonium salts. This protocol involves the one-pot, four-component coupling reaction of commercially available thiols and aldehydes with Ph_3_P and TfOH. The utility of the resulting thiophosphonium salt building blocks was demonstrated by the chemoselective postfunctionalization of C(sp^3^)–^+^PPh_3_ groups to achieve divergent reduction and oxidations protocols. In this way, thioether, deuterated-thioether, thioester, and dithioester derivatives, structural motifs that are present in many important chemicals, are readily accessed. This includes the synthesis of the pesticide chemical chlorbenside from simple commercially available materials in only two steps. The setup of these C–P group postfunctionalizations is simple, and the products were obtained in good yields. These protocols should greatly simplify access to bioactive relevant chemicals and further advance their use in a variety of new applications.

## Data Availability

The data underlying this study are available in the published article and its Supporting Information.

## Supporting Information Available

Experimental procedures, product characterization data, and NMR spectra (PDF)
