# Supplementary material for: Synthesis and Functionalization of Thiophosphonium Salts: A Divergent Approach to Access Thioether, Thioester, and Dithioester Derivatives
Source: Org Lett. 2023 Aug 23;25(34):6396–400. doi: 10.1021/acs.orglett.3c02422 (PMC10476193; doi:10.1021/acs.orglett.3c02422)

Supporting Information

Synthesis and Functionalization of Thiophosphonium Salts: A Divergent Approach to Access Thioether, Thioester, and Dithioester Derivatives

Gurupada Hazra, and Ahmad Masarwa*

Institute of Chemistry, The Center for Nanoscience and Nanotechnology, and Casali Center for Applied Chemistry, The Hebrew University of Jerusalem, Jerusalem, 9190401, (Israel)

****Corresponding Author:*** *E-mail: Ahmad.Masarwa1@mail.huji.ac.il*

Table of Contents

Materials, Methods, and General Remarks--S-2

Typical Procedure-1 for the Synthesis of Thiophosphonium Salts (3) and Characterizations--S-3

Optimization for Reductive Hydrolysis of Thiophosphonium Salts (3) towards Thioether (4) Synthesis---S-23

Typical Procedure-2 for the Synthesis of Thioether (4) and Characterizations---------S-24

Typical Procedure-3 for the Synthesis of Deuterated Thioether (4-D) and Characterizations---S-36

Optimization for Oxidation Reaction of Thiophosphonium Salts (3) towards Thioester (5) ---S-41

Typical Procedure-4 for the Synthesis of Thioester (5) Characterizations-------------S-42

Optimization for Oxidation Reaction of Thiophosphonium Salts (3) towards Dithioester(6)--S-46

Typical Procedure-5 for the Synthesis of Dithioesters (6) and Characterizations-----S-47

NMR Studies towards Reaction Progress--S-54

References--S-60

NMR Spectral Graphics---S-62

Materials, Methods, and General Remarks:

Unless stated otherwise, reactions were performed in oven-dried glassware (vials) fitted with either rubber septa or plastic screw cap and were stirred with Teflon-coated magnetic stirring bars. Commercially obtained reagents such as trifluoromethanesulfonic acid (TfOH), PPh_3, aldehydes **1**, and thiophenol **2** were used as received. Commercial grade solvents, i.e., acetonitrile, dimethylsulphoxide, dimethylformamide, 1,4-dioxane, methanol, and ethanol were used as received unless mentioned. Dry tetrahydrofuran (THF) and dichloromethane were used from a solvent purification system and kept over anh. $MgSO_4$ before use. Room temperatures were almost 20 ˚C to 23 ˚C during the experiments unless mentioned the temperatures, for heating we used oil-bath. Thin-layer chromatography was performed using silica gel 60 F-254 precoated plates (0.25 mm) and was visualized by UV irradiation, Hanessian's stain, anisaldehyde stain, and other stains. Silica gel of particle size 230 - 400 mesh was used for Flash chromatography. ^{1}H and ^{13}C NMR spectra were recorded using 400 and 500 MHz spectrometers with ^{13}C operating frequencies of 100 and 125 MHz, respectively. ^{31}P and ^{19}F operation frequencies were 162 MHz, 203 MHz and 376 MHz, 471 MHz, respectively. D NMR spectra were recorded using 500 MHz spectrometers with 77 MHz frequency. Chemical shifts (δ) are reported in ppm relative to the residual solvent ($CDCl_3$) signal (δ = 7.26 for ^{1}H NMR and δ = 77.16 for ^{13}C NMR), (DMSO–*d*6) signal (δ = 2.50 (p) for ^{1}H NMR; δ = 39.52 (septet) for ^{13}C NMR), (CD_3CN) signal (δ = 1.96 (p) for ^{1}H NMR; δ = 118.26 for ^{13}C NMR). Data for ^{1}H NMR spectra are reported as follows: chemical shift (multiplicity, coupling constants, and the number of hydrogen). Abbreviations are as follows: s (singlet), d (doublet), t (triplet), q (quartet), m (multiplet), dd (doublet of doublet), dt (doublet of triplet), ddd (doublet of doublet of doublet) brs (broad singlet). High-Resolution Mass Spectrometry (HRMS) data were recorded mostly on maXis impact 282001.00128 mass spectrometer using the 'APCI_pos_SolidProbe.m' method in the positive mode using methanol as a solvent.

Typical Procedure-1 for the Synthesis of Thiophosphonium Salts (3) and Characterizations:

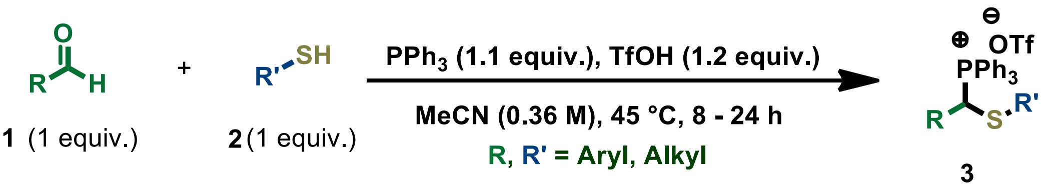

A dry 15 mL screw-capped vial equipped with a magnetic stir bar and teflon cap was charged with triphenylphosphine (PPh_3) (1.6 mmol; 1.1 equiv), MeCN (4 mL) and TfOH (1.76 mmol; 1.2 equiv). The reaction mixture was stirred for 5 minutes at room temperature. Then, it was charged with aldehyde (**1**) (1.46 mmol; 1.0 equiv) and again stirred for another 30 minutes at room temperature. After that, the corresponding thiophenol (**2**) (1.46 mmol; 1.0 equiv) was added to the vial and the reaction mixture was stirred at 45 ˚C (pre-heated oil bath was used) for 8 h – 24 h. Next, the reaction was stopped, and the reaction solvent was evaporated to dryness by using rotavapor. Then the crude reaction mass was washed with diethyl ether, precipitated by dissolving minimum volume ethanol/n-pentane or ethyl acetate/n-pentane or diethyl ether/n-pentane. The precipitated product dried over high vacuum and isolated as pure thiophosphonium salts and characterized below (Note: In few cases salts were purified by column chromatography as mentioned during characterization; this reaction condition was little modified from our previous work[1]; no further optimizations were performed.).

Supplementary Table 1: Scope of Thiophosphonium Salt (**3**) Products.

Entry	Aldehyde derivative (1)	Thiol derivative (2)	Solvent	Temp. [°C]	Time [h]	Product (3)	Yield [a, b] [%]
1	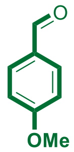 (1a)	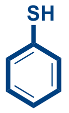 (2a)	MeCN	45	24 h	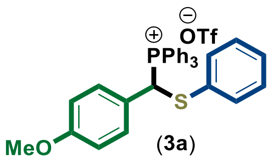 (3a)	97
2	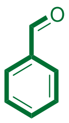 (1b)	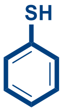 (2a)	MeCN	45	24 h	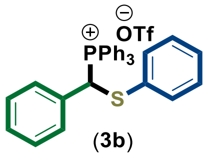 (3b)	96

**3**	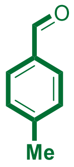 (1c)	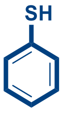 (2a)	MeCN	45	24 h	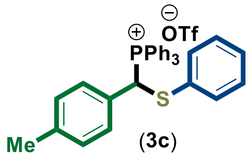 (3c)	91
**4**	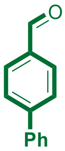 (1d)	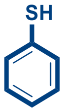 (2a)	MeCN	45	20 h	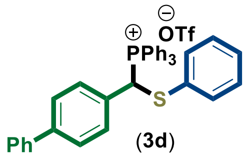 (3d)	62
**5**	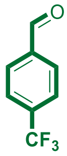 (1e)	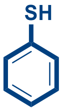 (2a)	MeCN	45	8 h	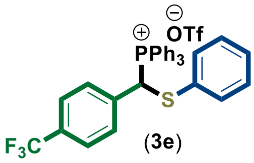 (3e)	44
**6**	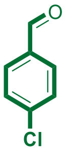 (1f)	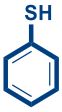 (2a)	MeCN	45	20 h	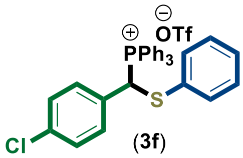 (3f)	65
**7**	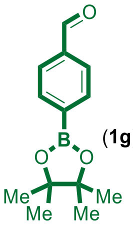 (1g)	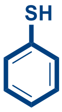 (2a)	MeCN	45	15 h	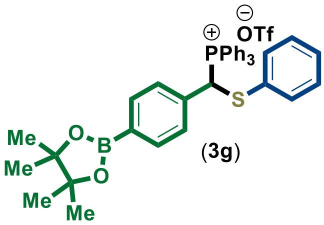 (3g)	71
**8**	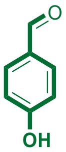 (1h)	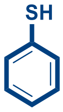 (2a)	MeCN	45	24 h	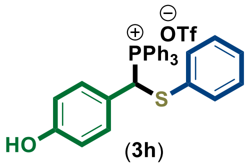 (3h)	94
**9**	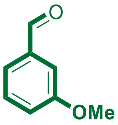 (1i)	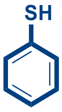 (2a)	MeCN	45	20 h	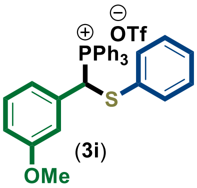 (3i)	43
**10**	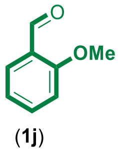 (1j)	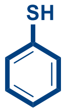 (2a)	MeCN	45	24 h	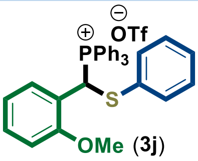 (3j)	96

**11**	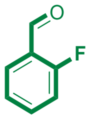 (**1k**)	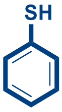 (**2a**)	MeCN	45	20 h	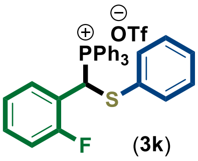	63
**12**	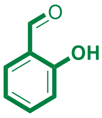 (**1l**)	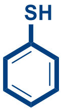 (**2a**)	MeCN	60	24 h	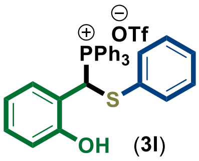	93
**13**	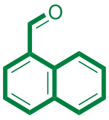 (**1m**)	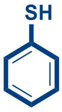 (**2a**)	MeCN	45	20 h	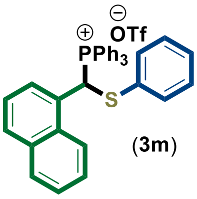	73
**14**	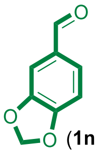 (**1n**)	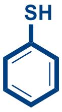 (**2a**)	MeCN	45	24 h	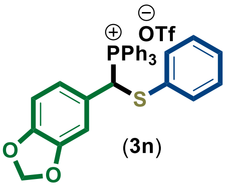	92
**15**	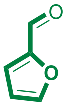 (**1o**)	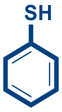 (**2a**)	MeCN	45	20 h	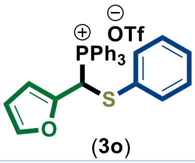	81
**16**	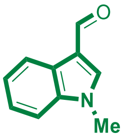 (**1p**)	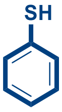 (**2a**)	MeCN	80	20 h	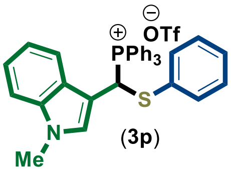	64
**17**	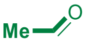 (**1q**)	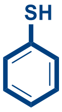 (**2a**)	MeCN	80	20 h	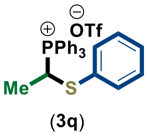	27
**18**	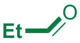 (**1r**)	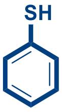 (**2a**)	MeCN	45	20 h	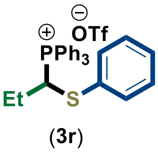	29
**19**	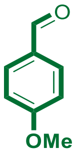 (**1a**)	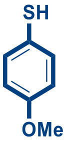 (**2b**)	MeCN	45	23 h	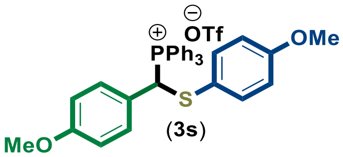	96

**20**	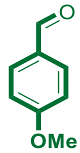 (1a)	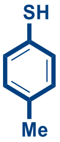 (2c)	MeCN	45	24 h	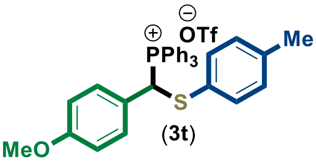 (3t)	89
**21**	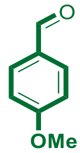 (1a)	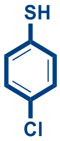 (2d)	MeCN	45	24 h	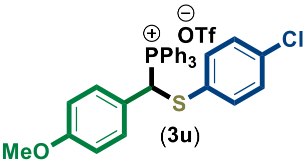 (3u)	90
**22**	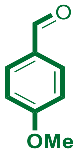 (1a)	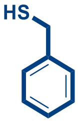 (2e)	MeCN	45	22 h	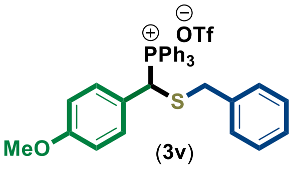 (3v)	93
**23**	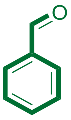 (1b)	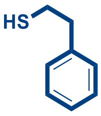 (2f)	MeCN	45	22 h	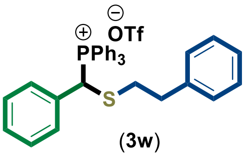 (3w)	66
**24**	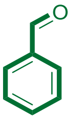 (1b)	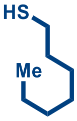 (2g)	MeCN	45	20 h	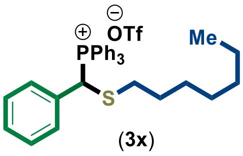 (3x)	94
**25**	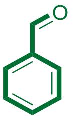 (1b)	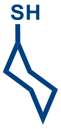 (2h)	MeCN	45	20 h	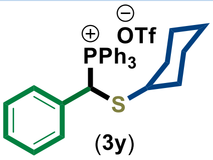 (3y)	88
**26**	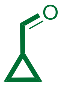 (1z)	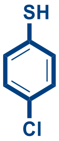 (2d)	MeCN	45	24 h	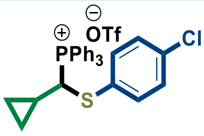 (3z)	96
**27**	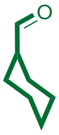 (1y)	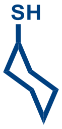 (2h)	MeCN	45	24 h	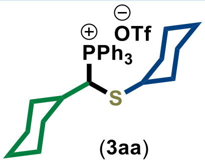 (3aa)	65
**28**	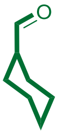 (1y)	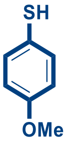 (2b)	MeCN	45	24 h	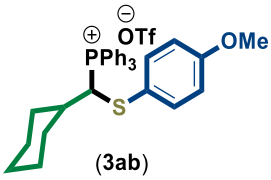 (3ab)	95

**29**	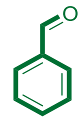 (**1b**)	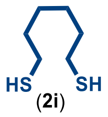 (**2i**)	MeCN	45	24 h	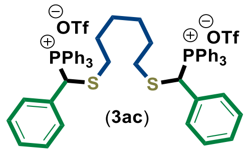 (**3ac**)	84
**30**	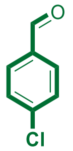 (**1f**)	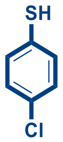 (**2d**)	MeCN	45	24 h	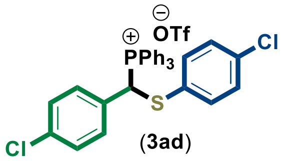 (**3ad**)	80
**31**	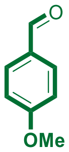 (**1a**)	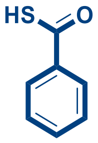 (**2j**)	MeCN	45	24 h	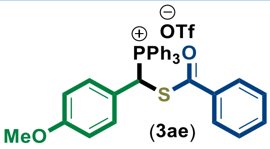 (**3ae**)	81

[a]Reactions were carried out with 1.46 mmol (1.0 equiv) of aldehyde derivative **1**, 1.46 mmol (1.0 equiv) of thiol derivative **2**, 1.6 mmol (1.1 equiv) of PPh_3, and 01.76 mmol (1.2 equiv) of TfOH. [b] Isolated yields. **Note (1):** Thiophosphonium salts **3d**, **3e**, **3f**, **3i**, **3k**, **3m**, **3q**, **3r**, and **3x** were purified by Flash column chromatography over silica gel (gradient elution: $MeOH/CH_2Cl_2$ = 1/99 to 3/97).

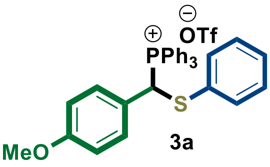

3a

((4-methoxyphenyl)(phenylthio)methyl)triphenylphosphonium) trifluoromethanesulfonate (3a):[1]

Following Typical Procedure-1, thiophosphonium salt **3a** was isolated (97%, 906 mg) as a white solid (without column chromatography); **^{1}H NMR (400 MHz, $CDCl_3$)** δ/ppm: 7.76 (t, J = 7.3 Hz, 3H), 7.61 (ddt, J = 11.7, 7.9, 5.8 Hz, 12H), 7.30 (dd, J = 6.3, 2.8 Hz, 2H), 7.22 – 7.11 (m, 3H), 7.04 (d, J = 7.0 Hz, 2H), 6.65 (d, J = 8.7 Hz, 2H), 6.37 (d, J = 13.8 Hz, 1H), 3.69 (s, 3H); **^{13}C NMR (100 MHz, $CDCl_3$)** δ/ppm: 160.3 (d, J = 2.5 Hz), 135.3 (d, J = 2.8 Hz), 134.8 (d, J = 9.3 Hz), 132.8, 132.1 (d, J = 5.5 Hz), 131.2 (d, J = 7.0 Hz), 130.1 (d, J = 12.5 Hz), 129.5, 128.9, 122.5 (d, J = 3.3 Hz), 120.9 (q, J = 321.0 Hz), 116.9 (d, J = 84.3 Hz); 114.4 (d, J = 1.7 Hz), 55.3, 46.8 (d, J = 46.6 Hz); **^{31}P NMR (203 MHz, $CDCl_3$)** δ/ppm: 24.2; **^{19}F NMR (376 MHz, $CDCl_3$)** δ/ppm: -78.1;

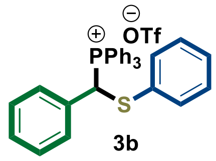

3b

Triphenyl(phenyl(phenylthio)methyl)phosphonium trifluoromethanesulfonate (3b):[1] Following Typical Procedure-1, thio-phosphonium salt **3b** was isolated (96%, 856 mg) as a white solid (without column chromatography); **^{1}H NMR (400 MHz, CDCl$_3$)** δ/ppm: 7.79 (ddd, J = 6.7, 5.3, 2.1 Hz, 3H), 7.72 – 7.55 (m, 12H), 7.36 – 7.25 (m, 3H), 7.22 – 7.11 (m, 7H), 6.34 (d, J = 14.1 Hz, 1H); **^{13}C NMR (100 MHz, CDCl$_3$)** δ/ppm: 135.5 (d, J = 3.1 Hz), 134.9 (d, J = 9.3 Hz), 133.0, 131.4 (d, J = 3.3 Hz), 131.1 (d, J = 7.0 Hz), 130.9 (d, J = 5.6 Hz), 130.2 (d, J = 12.6 Hz), 129.8 (d, J = 2.8 Hz), 129.7, 129.2, 129.2 (d, J = 2.0 Hz), 120.7 (q, J = 320.2 Hz),116.8 (d, J = 84.5 Hz), 47.8 (d, J = 46.0 Hz); **^{31}P NMR (162 MHz, CDCl$_3$)** δ/ppm: 24.0; **^{19}F NMR (376 MHz, CDCl$_3$)** δ/ppm: -78.3;

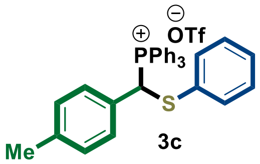

3c

Triphenyl((phenylthio)(p-tolyl)methyl)phosphonium trifluoromethanesulfonate (3c): Following Typical Procedure-1, thio-phosphonium salt **3c** was isolated (91%, 830 mg) as a white solid (without column chromatography); **^{1}H NMR (400 MHz, CDCl$_3$)** δ/ppm: 7.77 – 7.73 (m, 3H), 7.61 (tddd, J = 8.3, 6.8, 5.1, 1.5 Hz, 12H), 7.39 – 7.23 (m, 2H), 7.23 – 7.08 (m, 3H), 7.08 – 6.89 (m, 4H), 6.29 (d, J = 13.8 Hz, 1H), 2.24 (d, J = 1.9 Hz, 3H); **^{13}C NMR (100 MHz, CDCl$_3$)** δ/ppm: 139.9 (d, J = 3.0 Hz), 135.4 (d, J = 2.9 Hz), 134.8 (d, J = 9.4 Hz), 132.7, 131.3 (d, J = 6.7 Hz), 130.6 (d, J = 5.5 Hz), 130.1 (d, J = 12.6 Hz), 129.8 (d, J = 2.0 Hz), 129.6, 129.0, 128.0 (d, J = 3.3 Hz), 120.9 (q, J = 320.8 Hz), 116.8 (d, J = 84.6 Hz), 47.4 (d, J = 46.1 Hz), 21.2; **^{31}P NMR (162 MHz, CDCl$_3$)** δ/ppm: 23.6; **^{19}F NMR (376 MHz, CDCl$_3$)** δ/ppm: -78.1; **HRMS** [APCI] was calculated for $[C_{32}H_{28}PS]^+$ $[M]^+$: m/z 475.1644, found = 475.1642.

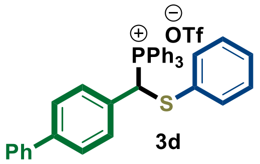

3d

([1,1'-biphenyl]-4-yl(phenylthio)methyl)triphenylphosphonium trifluoromethanesulfonate (3d): Following Typical Procedure-1, thiophosphonium salt **3d** was isolated (62%, 830 mg), R_f = 0.39 (10% MeOH in CH_2Cl_2) as a colorless gummy gel after

column chromatography by using 1: 99 (MeOH/CH_2Cl_2) to 2: 98 (MeOH/CH_2Cl_2) as an eluent system; **^{1}H NMR (400 MHz, $CDCl_3$)** δ/ppm: 7.78 – 7.71 (m, 9H), 7.61 (td, J = 7.8, 3.7 Hz, 6H), 7.54 – 7.49 (m, 2H), 7.44 – 7.34 (m, 7H), 7.27 (dd, J = 8.3, 2.0 Hz, 2H), 7.23 – 7.19 (m, 3H), 6.73 (d, J = 14.3 Hz, 1H); **^{13}C NMR (100 MHz, $CDCl_3$)** δ/ppm:142.2 (d, J = 3.0 Hz), 139.6, 135.4 (d, J = 2.9 Hz), 135.0 (d, J = 9.4 Hz), 132.8, 132.2 (d, J = 10.0 Hz), 131.5 (d, J = 5.5 Hz), 130.1 (d, J = 12.5 Hz), 129.7, 129.1, 129.0, 128.6 (d, J = 12.2 Hz), 128.1, 127.5 (d, J = 2.0 Hz), 127.1, 121.0 (q, J = 320.8 Hz), 117.1 (d, J = 84.4 Hz), 46.7 (d, J = 45.7 Hz); **^{31}P NMR (162 MHz, $CDCl_3$)** δ/ppm: 24.0; **^{19}F NMR (376 MHz, $CDCl_3$)** δ/ppm: -78.1; **HRMS** [APCI] was calculated for $[C_{37}H_{30}PS]^+$ $[M]^+$: m/z 537.1800, found = 537.1814.

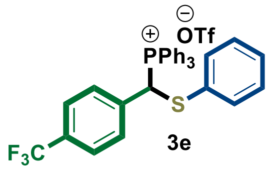

Triphenyl((phenylthio)(4-(trifluoromethyl)phenyl)methyl)phosphonium trifluoromethanesulfonate (3e): Following Typical Procedure-1, thiophosphonium salt **3e** was isolated (44%, 436 mg), R_f = 0.43 (10% MeOH in CH_2Cl_2) as a colorless gummy gel (after column chromatography by using 1: 99 (MeOH/CH_2Cl_2) to 2: 98 (MeOH/CH_2Cl_2) as an eluent system; **^{1}H NMR (400 MHz, $CDCl_3$)** δ/ppm: 7.81 – 7.73 (m, 9H), 7.65 – 7.60 (m, 6H), 7.42 – 7.34 (m, 6H), 7.22 – 7.20 (m, 3H), 6.92 (d, J = 14.9 Hz, 1H); **^{13}C NMR (100 MHz, $CDCl_3$)** δ/ppm: 136.0 (d, J = 1.7 Hz), 135.6 (d, J = 3.1 Hz), 134.9 (d, J = 9.5 Hz), 134.2 (d, J = 9.8 Hz), 132.9, 131.5 (d, J = 5.3 Hz), 130.3 (d, J = 12.5 Hz), 130.3, 129.8, 129.3, 125.8 (dd, J = 6.2, 3.1 Hz), 123.6 (q, J = 321 Hz), 120.8 (q, J = 320.7 Hz), 116.7 (d, J = 84.8 Hz), 46.0 (d, J = 46.0 Hz); **^{31}P NMR (162 MHz, $CDCl_3$)** δ/ppm: 24.8, **^{19}F NMR (376 MHz, $CDCl_3$)** δ/ppm: -62.9, -78.2; **HRMS** [APCI] was calculated for $[C_{32}H_{25}F_3PS]^+$ $[M]^+$: m/z 529.1361, found = 529.1376.

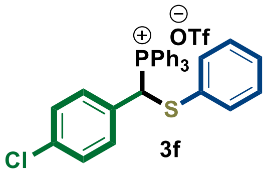

((4-chlorophenyl)(phenylthio)methyl)triphenylphosphonium trifluoromethanesulfonate (3f): Following Typical Procedure-1, thiophosphonium salt **3f** was isolated (65%, 612 mg), R_f = 0.26 (10% MeOH in CH_2Cl_2) as a colorless gummy gel (after column chromatography by using 1: 99 (MeOH/CH_2Cl_2) to 2: 98 (MeOH/CH_2Cl_2) as an eluent system; **^{1}H NMR (400**

MHz, $CDCl_3$) δ/ppm: 7.81 – 7.71 (m, 9H), 7.65 – 7.60 (m, 6H), 7.36 – 7.34 (m, 2H), 7.22 – 7.19 (m, 3H), 7.18 – 7.10 (m, 4H), 6.77 (d, J = 14.7 Hz, 1H); **^{13}C NMR (100 MHz, $CDCl_3$)** δ/ppm: 135.7 (d, J = 3.6 Hz), 135.5 (d, J = 2.8 Hz), 134.9 (d, J = 9.5 Hz), 132.4 (d, J = 5.5 Hz), 132.2 (d, J = 10.0 Hz), 130.2 (d, J = 12.7 Hz), 130.0 (d, J = 3.6 Hz), 129.7, 129.2, 129.2 (d, J = 2.3 Hz), 128.6 (d, J = 12.0 Hz), 121.0 (q, J = 320.7 Hz), 116.8 (d, J = 84.6 Hz), 45.9 (d, J = 46.3 Hz); **^{31}P NMR (162 MHz, $CDCl_3$)** δ/ppm: 24.3; **^{19}F NMR (376 MHz, $CDCl_3$)** δ/ppm: -78.2; **HRMS** [APCI] was calculated for $[C_{31}H_{25}ClPS]^+$ $[M]^+$: m/z 495.1098, found = 495.1113.

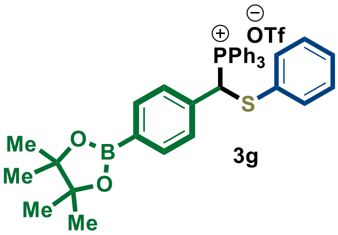

3g

Triphenyl((phenylthio)(4-(4,4,5,5-tetramethyl-1,3,2-dioxaborolan-2-yl)phenyl)methyl)phosphonium trifluoromethanesulfonate (3g): Following Typical Procedure-1, thiophosphonium salt **3g** was isolated (71%, 763 mg) as a white solid (without column chromatography); **^{1}H NMR (400 MHz, CDCl3)** δ/ppm: 7.80 – 7.76 (m, 3H), 7.68 – 7.57 (m, 14H), 7.30 – 7.28 (m, 2H), 7.19 – 7.18 (m, 3H), 7.13 (dd, J = 8.2, 1.8 Hz, 2H), 6.39 (d, J = 14.4 Hz, 1H), 1.31 (s, 12H); **^{13}C NMR (100 MHz, $CDCl_3$)** δ/ppm: 135.5 (d, J = 2.9 Hz), 135.3 (d, J = 1.9 Hz), 134.9 (d, J = 9.4 Hz), 134.3, 132.8, 132.5 (d, J = 10.0 Hz), 131.0 (d, J = 6.9 Hz), 130.4 (d, J = 12.6 Hz), 130.2 (d, J = 12.6 Hz), 129.4 (d, J = 13 Hz), 129.1, 121.0 (q, J = 320.7 Hz), 116.8 (d, J = 84.8 Hz), 84.3, 47.3 (d, J = 45.8 Hz), 25.0 (d, J = 3.3 Hz); **^{31}P NMR (162 MHz, $CDCl_3$)** δ/ppm: 24.1; **^{19}F NMR (376 MHz, $CDCl_3$)** δ/ppm: -78.1; **^{11}B NMR (128 MHz, $CDCl_3$)** δ/ppm: 29.1; **HRMS** [APCI] was calculated for $[C_{37}H_{37}BO_2PS]^+$ $[M]^+$: m/z 587.2339, found = 587.2371.

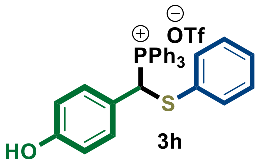

3h

((4-hydroxyphenyl)(phenylthio)methyl)triphenylphosphonium trifluoromethanesulfonate (3h): Following Typical Procedure-1, thiophosphonium salt **3h** was isolated (94%, 860 mg) as a light orange solid (without column chromatography); **^{1}H NMR (400 MHz, DMSO)** δ/ppm: 9.87 (brs, 1H), 7.95 – 7.88 (m, 3H), 7.78 – 7.72 (m, 12H), 7.36 – 7.28 (m, 5H), 6.97 (dd, J = 8.7, 2.0 Hz, 2H), 6.92 (d, J = 15.5 Hz, 1H), 6.64 (d, J = 8.5 Hz, 2H); **^{13}C NMR (100 MHz, DMSO)** δ/ppm: 158.3 (d, J = 2.7 Hz), 135.3 (d, J = 2.6 Hz), 134.6 (d, J = 9.5 Hz), 131.9 (d, J = 5.3 Hz), 131.8, 131.3 (d, J = 8.4 Hz), 130.0 (d, J = 12.3 Hz), 129.4, 128.7, 120.8 (d, J = 3.5 Hz), 120.7 (q, J = 322.5 Hz), 116.9 (d, J = 84.3 Hz), 115.6

(d, J = 1.1 Hz), 44.3 (d, J = 46.9 Hz); **^{31}P NMR (162 MHz, DMSO)** δ/ppm: 23.2; **^{19}F NMR (376 MHz, DMSO)** δ/ppm: -77.7; **HRMS** [APCI] was calculated for $[C_{31}H_{26}OPS]^+$ $[M]^+$: m/z 477.1436, found = 477.1449.

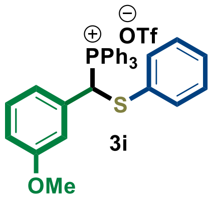

3i

((3-methoxyphenyl)(phenylthio)methyl)triphenylphosphonium trifluoromethanesulfonate (3i): Following Typical Procedure-1, thiophosphonium salt **3i** was isolated (43%, 402 mg), R_f = 0.37 (10% MeOH in CH_2Cl_2) as a pale yellow gummy gel (after column chromatography by using 1: 99 (MeOH/CH_2Cl_2) to 2: 98 (MeOH/CH_2Cl_2) as an eluent system; **^{1}H NMR (400 MHz, $CDCl_3$)** δ/ppm: 7.76 (t, J = 7.0 Hz, 3H), 7.62 (ddt, J = 11.6, 7.9, 5.8 Hz, 12H), 7.33 (dd, J = 6.0, 3.2 Hz, 2H), 7.18 (d, J = 2.9 Hz, 3H), 7.07 (t, J = 8.1 Hz, 1H), 6.79 (d, J = 8.1 Hz, 1H), 6.74 (d, J = 3.9 Hz, 2H), 6.44 (d, J = 13.8 Hz, 1H), 3.52 (s, 3H); **^{13}C NMR (100 MHz, $CDCl_3$)** δ/ppm: 159.8 (d, J = 1.8 Hz), 135.4 (d, J = 2.8 Hz), 134.9 (d, J = 9.4 Hz), 132.8, 132.7 (d, J = 3.1 Hz), 131.2 (d, J = 6.5 Hz), 130.1 (d, J = 12.6 Hz), 130.0, 129.6, 129.0, 122.8 (d, J = 5.6 Hz), 121.0 (q, J = 321.0 Hz), 116.9 (d, J = 84.5 Hz), 116.7 (d, J = 2.8 Hz), 115.4 (d, J = 5.5 Hz), 55.5, 47.6 (d, J = 45.7 Hz); **^{31}P NMR (162 MHz, $CDCl_3$)** δ/ppm: 23.8; **^{19}F NMR (376 MHz, $CDCl_3$)** δ/ppm: -78.1; **HRMS** [APCI] was calculated for $[C_{32}H_{28}OPS]^+$ $[M]^+$: m/z 491.1593, found = 491.1604.

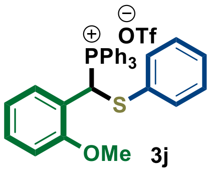

3j

((2-methoxyphenyl)(phenylthio)methyl)triphenylphosphonium trifluoromethanesulfonate (3j): Following Typical Procedure-1, thiophosphonium salt **3j** was isolated (96%, 897 mg) as a white solid (without column chromatography); **^{1}H NMR (400 MHz, $CDCl_3$)** δ/ppm: 7.88 – 7.82 (m, 3H), 7.66 – 7.61 (m, 6H), 7.53 – 7.47 (m, 6H), 7.38 (ddt, J = 8.4, 7.4, 1.8 Hz, 1H), 7.31 – 7.25 (m, 1H), 7.22 (ddd, J = 8.4, 4.5, 1.3 Hz, 2H), 7.18 – 7.13 (m, 2H), 7.01 (dt, J = 7.7, 1.9 Hz, 1H), 6.89 (t, J = 7.5 Hz, 1H), 6.82 (d, J = 8.4 Hz, 1H), 6.16 (d, J = 14.7 Hz, 1H), 3.43 (s, 3H); **^{13}C NMR (100 MHz, $CDCl_3$)** δ/ppm: 156.6 (d, J = 5.4 Hz), 135.7 (d, J = 3.0 Hz), 134.4 (d, J = 9.4 Hz), 132.6 (d, J = 5.6 Hz), 132.2, 131.8 (d, J = 2.9 Hz),

130.7 (d, J = 4.7 Hz), 130.3 (d, J = 12.5 Hz), 129.8, 129.4, 122.7, 121.3 (q, J = 321.0 Hz), 119.5, 116.7 (d, J = 84.6 Hz), 111.4, 55.6, 43.0 (d, J = 48.8 Hz); **^{31}P NMR (162 MHz, CDCl$_3$)** δ/ppm: 22.8; **^{19}F NMR (376 MHz, CDCl$_3$)** δ/ppm: -78.0; **HRMS** [APCI] was calculated for $[C_{32}H_{28}OPS]^+$ $[M]^+$: m/z 491.1593, found = 491.1622.

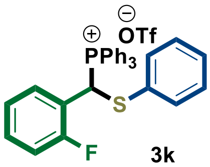

3k

((2-fluorophenyl)(phenylthio)methyl)triphenylphosphonium trifluoromethanesulfonate (3k): Following Typical Procedure-1, thiophosphonium salt **3k** was isolated (63%, 578 mg), R_f = 0.35 (10% MeOH in CH_2Cl_2) as a colorless gummy gel (after column chromatography by using 1: 99 (MeOH/CH_2Cl_2) to 2: 98 (MeOH/CH_2Cl_2) as an eluent system; **^{1}H NMR (400 MHz, CDCl$_3$)** δ/ppm: 7.88 – 7.83 (m, 3H), 7.69 – 7.63 (m, 6H), 7.61 – 7.55 (m, 6H), 7.42 – 7.35 (m, 1H), 7.32 – 7.27 (m, 1H), 7.25 – 7.17 (m, 4H), 7.15 – 7.09 (m, 2H), 6.99 – 6.95 (m, 1H), 6.00 (dd, J = 14.8, 1.5 Hz, 1H); **^{13}C NMR (100 MHz, CDCl$_3$)** δ/ppm: 136.1 (d, J = 2.9 Hz), 134.7 (d, J = 9.5 Hz), 132.9, 132.4 (d, J = 2.6 Hz), 132.3 (d, J = 2.8 Hz), 131.9 (d, J = 4.8 Hz), 131.6 (d, J = 5.7 Hz), 130.6 (d, J = 12.7 Hz), 130.0, 129.9, 125.9 – 125.8 (m), 121.1 (q, J = 321.0 Hz), 119.5 (dd, J = 13.0, 1.9 Hz), 116.3 (dd, J = 21.9, 1.7 Hz), 116.2 (d, J = 84.9 Hz), 43.5 (d, J = 49.4 Hz); **^{31}P NMR (162 MHz, CDCl$_3$)** δ/ppm: 23.8; **^{19}F NMR (376 MHz, CDCl$_3$)** δ/ppm: -78.0, -113.1; **HRMS** [APCI] was calculated for $[C_{31}H_{25}FPS]^+$ $[M]^+$: m/z 479.1393, found = 479.1401.

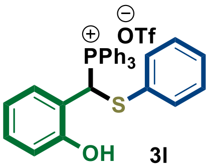

3l

((2-hydroxyphenyl)(phenylthio)methyl)triphenylphosphonium trifluoromethanesulfonate (3l):[1] Following Typical Procedure-1, thiophosphonium salt **3l** was isolated (93%, 851 mg) as a white solid (without column chromatography); **^{1}H NMR (400 MHz, CDCl$_3$)** δ/ppm: 7.81 – 7.76 (m, 3H), 7.61 – 7.53 (m, 12H), 7.24 – 7.11 (m, 7H), 6.67 – 6.65 (m, 2H), 6.36 (d, J = 14.3 Hz, 1H); **^{13}C NMR (100 MHz, CDCl$_3$)** δ/ppm: 155.0 (d, J = 6.1 Hz), 135.6 (d, J = 2.9 Hz), 134.4 (d, J = 9.4 Hz), 133.1 (d, J = 5.7 Hz), 131.5, 131.4 (d, J = 2.6 Hz), 130.2 (d, J = 12.4 Hz), 129.9, 129.6 (d, J = 4.2 Hz), 129.0, 120.7 (q, J = 320.0 Hz), 120.3 (d, J = 2.4 Hz), 118.0 (d, J = 1.3 Hz), 117.3 (d, J = 84.4 Hz), 117.2 (d, J = 1.5 Hz), 41.5 (d, J = 49.0 Hz); **^{31}P NMR (162 MHz, CDCl$_3$)** δ/ppm: 23.4; **^{19}F NMR (376 MHz, CDCl$_3$)** δ/ppm: -78.1.

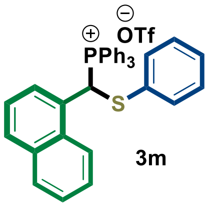

3m

Naphthalen-1-yl(phenylthio)methyl)triphenylphosphonium trifluoromethanesulfonate (3m): Following Typical Procedure-1, thiophosphonium salt **3m** was isolated (73%, 704 mg), R_f = 0.38 (10% MeOH in CH_2Cl_2) as a gummy gel (after column chromatography by using 1: 99 (MeOH/CH_2Cl_2) to 2: 98 (MeOH/CH_2Cl_2) as an eluent system; **^{1}H NMR (400 MHz, CDCl$_3$)** δ/ppm: 7.86 – 7.72 (m, 6H), 7.56 – 7.51 (m, 12H), 7.40 – 7.34 (m, 2H), 7.32 – 7.26 (m, 4H), 7.21 – 7.09 (m, 3H), 6.71 (d, J = 13.6 Hz, 1H); **^{13}C NMR (100 MHz, CDCl$_3$)** δ/ppm: 135.7 (d, J = 2.7 Hz), 134.9 (d, J = 9.3 Hz), 133.3, 132.2 (d, J = 5.3 Hz), 131.7 (d, J = 5.7 Hz), 130.8(d, J = 3.0 Hz), 130.2 (d, J = 12.4 Hz), 129.9, 129.6, 129.4 (d, J = 5.8 Hz), 128.8, 128.6 (d, J = 41.5 Hz), 128.4, 127.7 (d, J = 3.0 Hz), 126.8, 125.0 (d, J = 1.8 Hz), 122.3, 121.2 (q, J = 321.0 Hz), 116.3 (d, J = 84.4 Hz), 44.6 (d, J = 47.3 Hz); **^{31}P NMR (162 MHz, CDCl$_3$)** δ/ppm: 23.7; **^{19}F NMR (376 MHz, CDCl$_3$)** δ/ppm: -78.0; **HRMS** [APCI] was calculated for $[C_{35}H_{28}PS]^+$ $[M]^+$: m/z 511.1644, found = 511.1648.

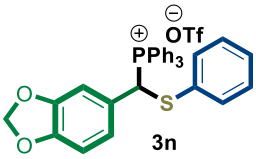

3n

(Benzo[d][1,3]dioxol-5-yl(phenylthio)methyl)triphenylphosphonium trifluoromethanesulfonate (3n): Following Typical Procedure-1, thiophosphonium salt **3n** was isolated (92%, 879 mg) as a white solid (without column chromatography); **^{1}H NMR (400 MHz, CDCl$_3$)** δ/ppm: 7.78 – 7.74 (m, 3H), 7.71 – 7.66 (m, 6H), 7.64 – 7.59 (m, 6H), 7.39 – 7.28 (m, 2H), 7.24 – 7.15 (m, 3H), 6.73 (dt, J = 8.1, 2.2 Hz, 1H), 6.57 (dd, J = 8.1, 0.4 Hz, 1H), 6.51 (t, J = 1.7 Hz, 1H), 6.39 (d, J = 14.2 Hz, 1H), 5.90 (dd, J = 5.7, 1.3 Hz, 2H); **^{13}C NMR (100 MHz, CDCl$_3$)** δ/ppm: 148.8 (d, J = 3.0 Hz), 148.2 (d, J = 2.2 Hz), 135.5 (d, J = 2.9 Hz), 134.9 (d, J = 9.4 Hz), 132.8, 131.0 (d, J = 7.1 Hz), 130.2 (d, J = 12.5 Hz), 129.7, 129.1, 125.7 (d, J = 6.8 Hz), 124.4 (d, J = 3.5 Hz), 120.8 (q, J = 320.5 Hz), 116.9 (d, J = 84.4 Hz), 110.2 (d, J = 4.6 Hz), 108.6 (d, J = 2.0 Hz), 101.8, 47.3 (d, J = 46.8 Hz); **^{31}P NMR (162 MHz, CDCl$_3$)** δ/ppm: 23.5; **^{19}F NMR (376 MHz, CDCl$_3$)** δ/ppm: -78.2; **HRMS** [APCI] was calculated for $[C_{32}H_{26}O_2PS]^+$ $[M]^+$: m/z 505.1386, found = 505.1367.

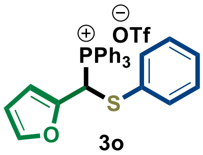

3o

(Furan-2-yl(phenylthio)methyl)triphenylphosphonium trifluoromethanesulfonate (3o): Following Typical Procedure-1, thiophosphonium salt **3o** was isolated (81%, 710 mg) as a pink solid (without column chromatography); **^{1}H NMR (400 MHz, $CDCl_3$)** δ/ppm: 7.80 – 7.75 (m, 9H), 7.66 – 7.60 (m, 6H), 7.31 – 7.29 (m, 2H), 7.26 – 7.22 (m, 4H), 6.71 (d, *J* = 15.8 Hz, 1H), 6.59 (td, *J* = 3.3, 0.8 Hz, 1H), 6.19 (ddd, *J* = 3.1, 1.8, 1.1 Hz, 1H); **^{13}C NMR (100 MHz, $CDCl_3$)** δ/ppm: 144.5 (d, *J* = 2.7 Hz), 143.8 (d, *J* = 6.7 Hz), 135.4 (d, *J* = 2.9 Hz), 134.6 (d, *J* = 9.9 Hz), 133.3, 130.7, 130.1 (d, *J* = 12.7 Hz), 129.6, 129.4, 120.8 (q, *J* = 320.6 Hz), 117.1 (d, *J* = 85.4 Hz), 115.1 (d, *J* = 7.9 Hz), 111.6 (d, *J* = 1.6 Hz), 41.8 (d, *J* = 51.6 Hz); **^{31}P NMR (162 MHz, $CDCl_3$)** δ/ppm: 24.5; **^{19}F NMR (376 MHz, $CDCl_3$)** δ/ppm: -78.2; **HRMS** [APCI] was calculated for $[C_{29}H_{24}OPS]^+$ $[M]^+$: m/z 451.1280, found = 451.1273.

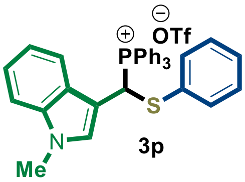

3p

((1-methyl-1H-indol-3-yl)(phenylthio)methyl)triphenylphosphonium trifluoromethanesulfonate (3p): Following Typical Procedure-1, thiophosphonium salt **3p** was isolated (64%, 620 mg) as a light pink solid (without column chromatography); **^{1}H NMR (400 MHz, $CDCl_3$)** δ/ppm: 7.78 – 7.67 (m, 9H), 7.58 – 7.53 (m, 6H), 7.30 – 7.24 (m, 3H), 7.18 – 7.12 (m, 5H), 6.89 – 6.81 (m, 2H), 6.69 (d, *J* = 13.3 Hz, 1H), 3.66 (d, *J* = 0.8 Hz, 3H); **^{13}C NMR (125 MHz, $CDCl_3$)** δ/ppm: 137.0, 135.2 (d, *J* = 2.7 Hz), 134.9 (d, *J* = 9.2 Hz), 132.7, 132.4, 132.2, 130.9, 130.1 (d, *J* = 12.3 Hz), 129.6, 128.9, 126.5 (d, *J* = 3.5 Hz), 122.6, 121.0 (q, *J* = 321.1 Hz), 119.9 (d, *J* = 52.7 Hz), 117.8 (d, *J* = 83.6 Hz), 110.1, 103.3 (d, *J* = 2.2 Hz), 42.3 (d, *J* = 50.1 Hz), 33.3; **^{31}P NMR (162 MHz, $CDCl_3$)** δ/ppm: 20.4; **^{19}F NMR (376 MHz, $CDCl_3$)** δ/ppm: -78.1; **HRMS** [APCI] was calculated for $[C_{34}H_{29}NPS]^+$ $[M]^+$: m/z 514.1753, found = 514.1768.

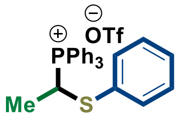

3q

Triphenyl(1-(phenylthio)ethyl)phosphonium trifluoromethanesulfonate (3q): Following Typical Procedure-1, thiophosphonium salt **3q** was isolated (27%, 216 mg), R_f = 0.43 (10% MeOH in CH_2Cl_2) as a light yellow gummy gel (after column chromatography by using 1: 99 ($MeOH/CH_2Cl_2$) to 2: 98 ($MeOH/CH_2Cl_2$) as an eluent system; **^{1}H NMR (400 MHz, $CDCl_3$)** δ/ppm: 7.84 (dd, J = 12.1, 7.5 Hz, 6H), 7.77 – 7.74 (m, 3H), 7.67 – 7.61 (m, 6H), 7.37 (dd, J = 6.4, 2.9 Hz, 2H), 7.25 (dd, J = 6.5, 3.3 Hz, 3H), 5.74 – 5.59 (m, 1H), 1.72 (dd, J = 17.8, 7.2 Hz, 3H).**^{13}C NMR (100 MHz, $CDCl_3$)** δ/ppm: 135.2 (d, J = 3.0 Hz), 134.3 (d, J = 9.4 Hz), 132.7, 131.1 (d, J = 4.5 Hz), 130.4 (d, J = 12.4 Hz), 129.8, 128.9, 121.1(q, J = 320.6 Hz), 117.3 (d, J = 84.9 Hz), 35.7 (d, J = 47.7 Hz), 18.1; **^{31}P NMR (162 MHz, $CDCl_3$)** δ/ppm: 27.9; **^{19}F NMR (376 MHz, $CDCl_3$)** δ/ppm: -78.1; **HRMS** [APCI] was calculated for $[C_{26}H_{24}PS]^+$ $[M]^+$: m/z 399.1331, found = 399.1343.

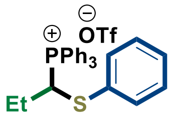

3r

Triphenyl(1-(phenylthio)propyl)phosphonium trifluoromethanesulfonate (3r): Following Typical Procedure-1, thiophosphonium salt **3r** was isolated (29%, 238 mg), R_f = 0.48 (10% MeOH in CH_2Cl_2) as a colorless gummy gel (after column chromatography by using 1: 99 ($MeOH/CH_2Cl_2$) to 2: 98 ($MeOH/CH_2Cl_2$) as an eluent system; **^{1}H NMR (400 MHz, $CDCl_3$)** δ/ppm: 7.83 – 7.78 (m, 6H), 7.74 – 7.69 (m, 3H), 7.61 – 7.54 (m, 6H), 7.27 – 7.23 (m, 2H), 7.22 – 7.20 (m, 3H), 5.38 – 5.33 (m, 1H), 2.30 – 2.25 (m, 1H), 1.80 – 1.76 (m, 1H), 1.49 – 1.46 (m, 3H); **^{13}C NMR (100 MHz, $CDCl_3$)** δ/ppm: 135.0 (d, J = 2.9 Hz), 134.2 (d, J = 9.2 Hz), 132.5 (d, J = 1.5 Hz), 131.7, 130.3 (d, J = 12.3 Hz), 129.7, 128.4, 121.05 (dd, J = 320.7 Hz), 117.6 (d, J = 84.4 Hz), 43.2 (d, J = 45.8 Hz), 25.3 (d, J = 1.7 Hz), 11.6 (d, J = 12.9 Hz); **^{31}P NMR (162 MHz, $CDCl_3$)** δ/ppm: 26.5; **^{19}F NMR (376 MHz, $CDCl_3$)** δ/ppm: -78.1; **HRMS** [APCI] was calculated for $[C_{27}H_{26}PS]^+$ $[M]^+$: m/z 413.1487, found = 4131492.

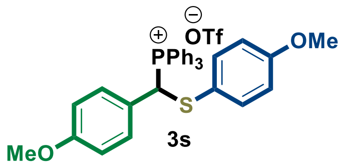

((4-methoxyphenyl)((4-methoxyphenyl)thio)methyl)triphenylphosphonium trifluoromethanesulfonate (3s): Following Typical Procedure-1, thiophosphonium salt **3s** was isolated (96%, 940 mg) as a white solid (without column chromatography); **^{1}H NMR (400 MHz, CDCl$_3$)** *δ*/ppm: 7.80 – 7.74 (m, 3H), 7.68 – 7.58 (m, 12H), 7.22 – 7.12 (m, 2H), 6.98 (dd, *J* = 8.8, 2.0 Hz, 2H), 6.74 – 6.65 (m, 4H), 6.14 (d, *J* = 13.5 Hz, 1H), 3.73 (s, 3H), 3.71 (s, 3H); **^{13}C NMR (100 MHz, CDCl$_3$)** *δ*/ppm: 160.7, 160.4 (d, *J* = 2.7 Hz), 135.8, 135.4 (d, *J* = 2.9 Hz), 134.8 (d, *J* = 9.4 Hz), 132.1 (d, *J* = 5.6 Hz), 130.1 (d, *J* = 12.4 Hz), 122.7 (d, *J* = 3.3 Hz), 121.4 (d, *J* = 7.2 Hz), 120.9 (q, *J* = 320.7 Hz),117.1 (d, *J* = 84.3 Hz), 115.2, 114.5 (d, J = 1.3 Hz), 55.5, 55.4, 48.0 (d, *J* = 45.4 Hz); **^{31}P NMR (162 MHz, CDCl$_3$)** *δ*/ppm: 22.9; **^{19}F NMR (376 MHz, CDCl$_3$)** *δ*/ppm: -78.1; ; **HRMS** [APCI] was calculated for $[C_{33}H_{30}O_2PS]^+$ $[M]^+$: m/z 521.1699, found = 521.1690.

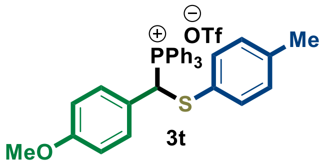

((4-methoxyphenyl)(p-tolylthio)methyl)triphenylphosphonium trifluoromethanesulfonate (3t): Following Typical Procedure-1, thiophosphonium salt **3t** was isolated (89%, 851 mg) as a white solid (without column chromatography); **^{1}H NMR (400 MHz, CDCl$_3$)** *δ*/ppm: 7.80 – 7.75 (m, 3H), 7.68 – 7.55 (m, 12H), 7.20 – 7.13 (m, 2H), 7.08 – 6.93 (m, 4H), 6.67 (d, *J* = 8.5 Hz, 2H), 6.20 (d, *J* = 13.8 Hz, 1H), 3.72 (s, 3H), 2.21 (s, 3H); **^{13}C NMR (100 MHz, CDCl$_3$)** *δ*/ppm: 160.5 (d, *J* = 2.6 Hz), 139.5, 135.4 (d, *J* = 3.0 Hz), 134.8 (d, *J* = 9.4 Hz), 133.2, 132.2 (d, *J* = 5.5 Hz), 130.4, 130.1 (d, *J* = 12.4 Hz), 127.6 (d, *J* = 7.2 Hz), 122.7 (d, *J* = 3.4 Hz), 120.8 (q, J = 320.6 Hz), 117.0 (d, *J* = 84.3 Hz), 114.5 (d, *J* = 1.7 Hz), 55.4, 47.5 (d, *J* = 46.1 Hz), 21.2; **^{31}P NMR (162 MHz, CDCl$_3$)** *δ*/ppm: 23.2; **^{19}F NMR (376 MHz, CDCl$_3$)** *δ*/ppm: -78.2; **HRMS** [ESI] was calculated for $[C_{33}H_{30}OPS]^+$ $[M]^+$: m/z 505.1749, found = 505.1742.

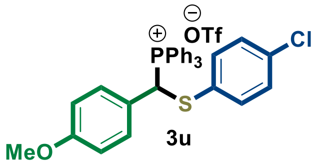

(((4-chlorophenyl)thio)(4-methoxyphenyl)methyl)triphenylphosphonium trifluoromethanesulfonate (3u): Following Typical Procedure-1, thiophosphonium salt **3u** was isolated (90%, 887 mg) as a white solid (without column chromatography); **^{1}H NMR (400 MHz, CDCl$_3$)** *δ*/ppm: 7.78 – 7.75 (m, 3H), 7.74 – 7.66 (m, 6H), 7.66 – 7.56 (m, 6H), 7.35 – 7.28 (m, 2H), 7.17 – 7.09 (m, 2H), 7.09 – 7.02 (m, 2H), 6.67 – 6.63 (m, 2H), 6.63 (d, *J* = 8.0 Hz, 1H), 3.71 (s, 3H); **^{13}C NMR (100 MHz, CDCl$_3$)** *δ*/ppm: 160.5 (d, *J* = 2.8 Hz), 135.4 (d, *J* = 2.9 Hz), 135.2, 134.9 (d, *J* = 9.4 Hz), 134.3, 132.4 (d, *J* = 5.5 Hz), 130.1 (d, *J* = 12.5 Hz), 129.7, 129.3 (d, *J* = 7.7 Hz), 122.1 (d, *J* = 3.8 Hz), 121.0 (q, *J* = 321.0 Hz), 117.0 (d, *J* = 84.4 Hz), 114.4, 55.4, 46.3 (d, *J* = 46.7 Hz); **^{31}P NMR (162 MHz, CDCl$_3$)** *δ*/ppm: 23.4; **^{19}F NMR (376 MHz, CDCl$_3$)** *δ*/ppm: -78.2; **HRMS** [APCI] was calculated for $[C_{32}H_{27}ClOPS]^+$ $[M]^+$: m/z 525.1203, found = 525.1198.

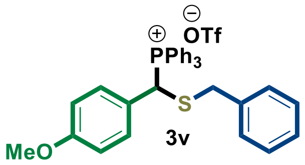

((Benzylthio)(4-methoxyphenyl)methyl)triphenylphosphonium trifluoromethanesulfonate (3v): Following Typical Procedure-1, thiophosphonium salt **3v** was isolated (93%, 889 mg) as a white solid (without column chromatography); **^{1}H NMR (400 MHz, CDCl$_3$)** *δ*/ppm: 7.80 – 7.75 (m, 3H), 7.66 – 7.51 (m, 12H), 7.27 – 7.19 (m, 3H), 7.15 – 7.07 (m, 4H), 6.81 – 6.72 (m, 2H), 6.00 (d, *J* = 16.3 Hz, 1H), 3.95 (d, *J* = 12.3 Hz, 1H), 3.83 (d, *J* = 12.3 Hz, 1H), 3.78 (s, 3H); **^{13}C NMR (100 MHz, CDCl$_3$)** *δ*/ppm: 160.6 (d, *J* = 2.9 Hz), 135.5, 135.4 (d, J = 2.9 Hz), 134.7 (d, *J* = 9.4 Hz), 132.4 (d, *J* = 5.4 Hz), 130.2 (d, *J* = 12.4 Hz), 129.6, 128.8, 127.9, 122.3 (d, *J* = 4.0 Hz), 121.0 (q, *J* = 320.8 Hz), 117.1 (d, *J* = 84.9 Hz), 114.5 (d, *J* = 2.1 Hz), 55.5 (s), 43.27 (d, *J* = 49.7 Hz), 38.1 (d, *J* = 7.4 Hz); **^{31}P NMR (162 MHz, CDCl$_3$)** *δ*/ppm: 24.0; **^{19}F NMR (376 MHz, CDCl$_3$)** *δ*/ppm: -78.1; **HRMS** [APCI] was calculated for $[C_{33}H_{30}OPS]^+$ $[M]^+$: m/z 505.1749, found = 505.1766.

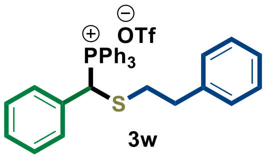

3w

((Phenethylthio)(phenyl)methyl)triphenylphosphonium trifluoromethanesulfonate (3w): Following Typical Procedure-1, thiophosphonium salt **3w** was isolated (66%, 615 mg) as a gel after washed with diethyl ether (without column chromatography); **^{1}H NMR (400 MHz, $CDCl_3$)** δ/ppm: 7.78 – 7.74 (m, 3H), 7.68 – 7.57 (m, 12H), 7.31 – 7.27 (m, 1H), 7.25 – 7.09 (m, 7H), 7.00 (dd, J = 5.1, 3.0 Hz, 2H), 6.35 (d, J = 16.2 Hz, 1H), 3.07 – 2.92 (m, 2H), 2.85–2.72 (m, 2H); **^{13}C NMR (100 MHz, $CDCl_3$)** δ/ppm: 139.4, 135.2 (d, J = 2.9 Hz), 134.8 (d, J = 9.3 Hz), 131.8 (d, J = 3.8 Hz), 131.1 (d, J = 5.6 Hz), 130.1 (d, J = 12.5 Hz), 129.6 (d, J = 3.0 Hz), 129.2 (d, J = 2.2 Hz), 128.7, 128.4, 126.4, 121.0 (q, J = 319.7 Hz), 117.2 (d, J = 85.0 Hz), 43.5 (d, J = 48.9 Hz), 35.7 (d, J = 6.7 Hz), 35.0; **^{31}P NMR (162 MHz, $CDCl_3$)** δ/ppm: 25.1; **^{19}F NMR (376 MHz, $CDCl_3$)** δ/ppm: -78.3; **HRMS** [APCI] was calculated for $[C_{33}H_{30}PS]^+$ $[M]^+$: m/z 489.1800, found = 489.1792.

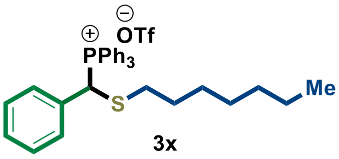

3x

((Heptylthio)(phenyl)methyl)triphenylphosphonium trifluoromethanesulfonate (3x): Following Typical Procedure-1, thiophosphonium salt **3x** was isolated (94%, 868 mg), R_f = 0.30 (10% MeOH in CH_2Cl_2) as a colorless gummy gel (after column chromatography by using 1: 99 (MeOH/CH_2Cl_2) to 2: 98 (MeOH/CH_2Cl_2) as an eluent system; **^{1}H NMR (400 MHz, $CDCl_3$)** δ/ppm: 7.80 – 7.75 (m, 3H), 7.73 – 7.67 (m, 6H), 7.65 – 7.60 (m, 6H), 7.29 – 7.24 (m, 3H), 7.22 – 7.18 (m, 2H), 6.32 (d, J = 16.6 Hz, 1H), 2.82 – 2.73 (m, 2H), 1.49 – 1.40 (m, 2H), 1.20 – 1.10 (m, 8H), 0.81 (t, J = 7.1 Hz, 3H); **^{13}C NMR (100 MHz, $CDCl_3$)** δ/ppm: 135.2 (d, J = 2.9 Hz), 134.8 (d, J = 9.3 Hz), 132.0 (d, J = 3.6 Hz), 131.1 (d, J = 5.6 Hz), 130.1 (d, J = 12.5 Hz), 129.5 (d, J = 3.2 Hz), 128.9 (d, J = 2.4 Hz), 121.0 (q, J = 320.6 Hz), 117.4 (d, J = 84.9 Hz), 43.5 (d, J = 48.9 Hz), 35.0, 34.9, 31.5, 28.5, 28.3, 22.5, 14.0; **^{31}P NMR (162 MHz, $CDCl_3$)** δ/ppm: 25.0; **^{19}F NMR (376 MHz, $CDCl_3$)** δ/ppm: -78.1; **HRMS** [APCI] was calculated for $[C_{32}H_{36}PS]^+$ $[M]^+$: m/z 483.2270, found = 483.2249.

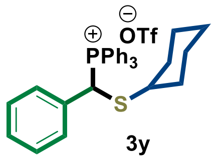

3y

((Cyclohexylthio)(phenyl)methyl)triphenylphosphonium trifluoromethanesulfonate (3y): Following Typical Procedure-1, thiophosphonium salt **3y** was isolated (88%, 792 mg) as a white solid (without column chromatography); **^{1}H NMR (400 MHz, CDCl$_3$)** δ/ppm: 7.78 – 7.74 (m, 3H), 7.71 – 7.57 (m, 12H), 7.25 (ddt, *J* = 9.7, 3.6, 1.8 Hz, 3H), 7.17 (dd, *J* = 8.0, 7.5 Hz, 2H), 6.21 (d, *J* = 17.1 Hz, 1H), 3.07 – 2.92 (m, 1H), 2.00 – 1.85 (m, 1H), 1.84 – 1.70 (m, 1H), 1.62 (dd, *J* = 9.8, 4.5 Hz, 1H), 1.56 – 1.37 (m, 2H), 1.37 – 1.08 (m, 5H); **^{13}C NMR (100 MHz, CDCl$_3$)** δ/ppm: 135.2 (d, *J* = 2.9 Hz), 134.8 (d, *J* = 9.2 Hz), 132.7 (d, *J* = 3.2 Hz), 131.0 (d, *J* = 5.6 Hz), 130.0 (d, *J* = 12.4 Hz), 129.4 (d, *J* = 3.1 Hz), 128.9 (d, *J* = 2.2 Hz), 121.0 (q, *J* = 320.8 Hz), 117.4 (d, *J* = 85.0 Hz), 48.1 (d, *J* = 6.4 Hz), 42.8 (d, *J* = 48.4 Hz), 33.3 (d, *J* = 92.5 Hz), 25.5 (t, *J* = 6.9 Hz); **^{31}P NMR (162 MHz, CDCl$_3$)** δ/ppm: 25.3; **^{19}F NMR (376 MHz, CDCl$_3$)** δ/ppm: -78.1; **HRMS** [APCI] was calculated for $[C_{31}H_{32}PS]^+$ $[M]^+$: m/z 467.1957, found = 467.1951.

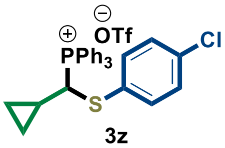

3z

(((4-chlorophenyl)thio)(cyclopropyl)methyl)triphenylphosphonium trifluoromethanesulfonate (3z): Following Typical Procedure-1, thiophosphonium salt **3z** was isolated (96%, 853 mg) as a white solid (without column chromatography); **^{1}H NMR (400 MHz, CDCl$_3$)** δ/ppm: 7.89 – 7.83 (m, 6H), 7.76 – 7.72 (m, 3H), 7.63 – 7.58 (m, 6H), 7.23– 7.20 (m, 2H), 7.16– 7.13 (m, 2H), 5.17 (dd, *J* = 10.2, 8.8 Hz, 1H), 1.22 – 1.12 (m, 1H), 0.75 (dd, *J* = 9.0, 4.1 Hz, 1H), 0.65 – 0.52 (m, 3H); **^{13}C NMR (100 MHz, CDCl$_3$)** δ/ppm: 135.1 (d, *J* = 2.9 Hz), 134.8, 134.5 (d, *J* = 9.5 Hz), 133.7, 130.1 (d, *J* = 12.4 Hz), 129.6, 120.9 (q, *J* = 320.8 Hz), 117.4 (d, *J* = 84.2 Hz), 45.8 (d, *J* = 47.2 Hz), 12.3 (d, *J* = 2.9 Hz), 8.0 (d, *J* = 2.7 Hz), 6.1 (d, *J* = 8.9 Hz); **^{31}P NMR (162 MHz, CDCl$_3$)** δ/ppm: 26.8; **^{19}F NMR (376 MHz, CDCl$_3$)** δ/ppm: -78.2; **HRMS** [APCI] was calculated for $[C_{28}H_{25}ClPS]^+$ $[M]^+$: m/z 459.1098, found = 459.1121.

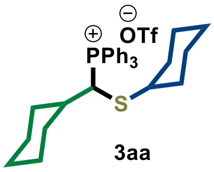

3aa

(Cyclohexyl(cyclohexylthio)methyl)triphenylphosphonium trifluoromethanesulfonate (3aa): Following Typical Procedure-1, thiophosphonium salt **3aa** was isolated (65%, 591 mg) as a white solid (without column chromatography); **^{1}H NMR (400 MHz, CDCl$_3$)** *δ*/ppm: 7.94 – 7.90 (m, 6H), 7.79– 7.74 (m, 3H), 7.69 – 7.64(m, 6H), 4.73 (dd, *J* = 13.6, 2.6 Hz, 1H), 2.51 – 2.30 (m, 1H), 2.17 (d, *J* = 9.1 Hz, 1H), 2.07 – 1.83 (m, 2H), 1.66 (ddd, *J* = 31.6, 26.4, 12.2 Hz, 3H), 1.54 – 1.39 (m, 4H), 1.39 – 1.14 (m, 5H), 1.10 – 0.96 (m, 3H), 0.96 – 0.82 (m, 3H); **^{13}C NMR (100 MHz, CDCl$_3$)** *δ*/ppm: 134.8 (d, *J* = 2.9 Hz), 134.5 (d, *J* = 9.0 Hz), 130.1 (d, *J* = 12.2 Hz), 121.0 (q, *J* = 321.1 Hz),118.9 (d, *J* = 83.2 Hz), 49.2, 46.1 (d, *J* = 44.0 Hz), 39.7 (d, *J* = 2.4 Hz), 34.1, 33.0, 31.9 (d, *J* = 11.9 Hz), 29.9 (d, *J* = 1.5 Hz), 26.4 (d, *J* = 1.1 Hz), 26.0, 25.7 (d, *J* = 33.5 Hz), 25.4 (d, *J* = 2.6 Hz); **^{31}P NMR (162 MHz, CDCl$_3$)** *δ*/ppm: 24.7; **^{19}F NMR (376 MHz, CDCl$_3$)** *δ*/ppm: -78.1; **HRMS** [APCI] was calculated for $[C_{31}H_{38}PS]^+$ $[M]^+$: m/z 473.2426, found = 473.2444.

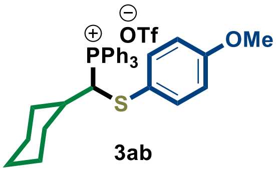

3ab

(Cyclohexyl((4-methoxyphenyl)thio)methyl)triphenylphosphonium trifluoromethanesulfonate (3ab): Following Typical Procedure-1, thiophosphonium salt **3ab** was isolated (95%, 897 mg) as a white solid (without column chromatography); **^{1}H NMR (400 MHz, CDCl$_3$)** *δ*/ppm: 7.74 – 7.72 (m, 9H), 7.57 – 7.57 (m, 6H), 6.97 (d, *J* = 6.9 Hz, 2H), 6.65 (d, *J* = 6.9 Hz, 2H), 4.82 (d, *J* = 10.3 Hz, 1H), 3.74 (s, 3H), 2.20 – 2.08 (m, 2H), 1.98 – 1.80 (m, 2H), 1.55 (t, *J* = 11.8 Hz, 2H), 1.34 – 1.22 (m, 2H), 1.13 – 0.91 (m, 3H); **^{13}C NMR (100 MHz, CDCl$_3$)** *δ*/ppm: 160.0, 135.0 (d, *J* = 2.8 Hz), 134.3, 134.1 (d, *J* = 5.6 Hz), 130.2 (d, *J* = 12.2 Hz), 123.4, 121.0 (q, *J* = 321.1 Hz), 118.0 (d, *J* = 83.0 Hz), 115.2, 55.6, 51.4 (d, *J* = 40.7 Hz), 39.3, 32.1 (d, *J* = 11.8 Hz), 27.9 (d, *J* = 330.1 Hz), 25.6 (d, *J* = 62.7 Hz); **^{31}P NMR (162 MHz, CDCl$_3$)** *δ*/ppm: 23.16; **^{19}F NMR (376 MHz, CDCl$_3$)** *δ*/ppm: -78.1; **HRMS** [ESI] was calculated for $[C_{32}H_{34}OPS]^+$ $[M]^+$: m/z 497.2062, found = 497.2083.

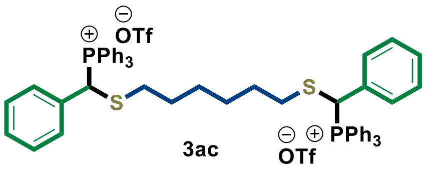

3ac

(Hexane-1,6-diylbis(sulfanediyl))bis(phenylmethylene))bis(triphenylphosphonium) trifluoromethanesulfonate (3ac): Following Typical Procedure-1, thiophosphonium salt **3ac** was isolated (84%, 1412 mg), $R_f = 0.34$ (10% MeOH in CH_2Cl_2) as a gummy gel (after column chromatography by using 1: 99 (MeOH/CH_2Cl_2) to 2: 98 (MeOH/CH_2Cl_2) as an eluent system; **^{1}H NMR (400 MHz, $CDCl_3$)** δ/ppm: 7.78 – 7.56 (m, 6H), 7.70 – 7.58 (m, 24H), 7.30 – 7.13 (m, 10H), 6.21 (dd, J = 16.3, 10.8 Hz, 2H), 2.70 – 2.55 (m, 4H), 1.46 – 1.35 (m, 4H), 1.17 – 1.06 (m, 4H); **^{13}C NMR (100 MHz, $CDCl_3$)** δ/ppm: 135.3 (d, J = 2.8 Hz), 134.9 (dd, J = 9.4, 2.1 Hz), 132.1 (dd, J = 6.1, 3.4 Hz), 131.1 (dd, J = 5.5, 2.1 Hz), 130.1 (d, J = 12.5 Hz), 129.6 (dd, J = 5.0, 3.2 Hz), 129.0 (dd, J = 5.0, 2.2 Hz), 121.0 (q, J = 321.0 Hz), 117.3 (dd, J = 85.0, 4.6 Hz), 43.5 (dd, J = 48.9, 5.8 Hz), 34.6 (dd, J = 13.4, 6.4 Hz), 28.3 (d, J = 3.2 Hz), 27.5 (d, J = 7.2 Hz); **^{31}P NMR (162 MHz, $CDCl_3$)** δ/ppm: 25.2; **^{19}F NMR (376 MHz, $CDCl_3$)** δ/ppm: -78.1.

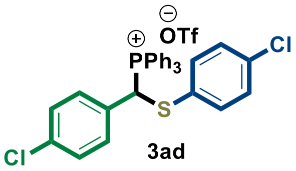

3ad

((4-chlorophenyl)((4-chlorophenyl)thio)methyl)triphenylphosphonium trifluoromethanesulfonate (3ad): Following Typical Procedure-1, thiophosphonium salt **3ad** was isolated (80%, 794 mg) as a white solid (without column chromatography); **^{1}H NMR (400 MHz, $CDCl_3$)** δ/ppm: 7.81 – 7.71 (m, 9H), 7.65 – 7.60 (m, 6H), 7.35 – 7.31 (m, 2H), 7.15 – 7.08 (m, 6H), 6.82 (d, J = 14.5 Hz, 1H); **^{13}C NMR (100 MHz, $CDCl_3$)** δ/ppm: 135.8 (d, J = 3.6 Hz), 135.5 (d, J = 2.9 Hz), 135.4, 134.9 (d, J = 9.5 Hz), 134.2, 132.4 (d, J = 5.3 Hz), 130.2 (d, J = 12.5 Hz), 129.8, 129.5 (d, J = 3.7 Hz), 129.2 (d, J = 2.0 Hz), 128.7 (d, J = 7.7 Hz), 121.1 (q, J = 321.0 Hz), 116.6 (d, J = 84.8 Hz), 45.6 (d, J = 46.4 Hz); **^{31}P NMR (162 MHz, $CDCl_3$)** δ/ppm: 24.2; **^{19}F NMR (376 MHz, $CDCl_3$)** δ/ppm: -78.1; **HRMS** [APCI] was calculated for $[C_{31}H_{24}Cl_2PS]^+$ $[M]^+$: m/z 529.0708, found = 529.0712.

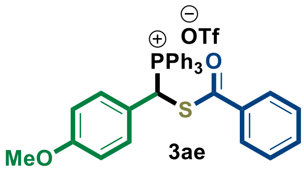

3ae

((Benzoylthio)(4-methoxyphenyl)methyl)triphenylphosphonium trifluoromethanesulfonate (3ae): Following Typical Procedure-1, thiophosphonium salt **3ae** was isolated (81%, 791 mg) as a white solid (without column chromatography); **^{1}H NMR (500 MHz, CD_3CN)** δ/ppm: 7.90 – 7.83 (m, 4H), 7.68 – 7.60 (m, 13H), 7.51 (dd, J = 8.1, 1.0 Hz, 1H), 7.41 (ddd, J = 8.0, 7.4, 1.6 Hz, 1H), 7.30 (td, J = 7.6, 1.1 Hz, 1H), 7.15 – 7.10 (m, 2H), 6.78 – 6.76 (m, 2H), 6.34 (d, J = 15.3 Hz, 1H), 3.72 (s, 3H); **^{13}C NMR (125 MHz, CD_3CN)** δ/ppm: 167.5, 161.6 (d, J = 2.7 Hz), 135.4 (d, J = 3.0 Hz), 135.8 (d, J = 9.6 Hz), 135.4 (d, J = 8.6 Hz), 133.8, 133.0 (d, J = 5.1 Hz), 132.3, 131.5 (d, J = 7.5 Hz), 131.0 (d, J = 12.3 Hz), 128.3, 122.9 (d, J = 3.8 Hz), 122.0 (q, J = 321.1 Hz), 117.7 (d, J = 85.0 Hz), 115.4 (d, J = 2.2 Hz), 56.1, 46.2 (d, J = 48.8 Hz); **^{31}P NMR (162 MHz, CD_3CN)** δ/ppm: 24.0; **^{19}F NMR (471 MHz, CD_3CN)** δ -79.2.

Optimization for Reductive Hydrolysis of Thiophosphonium Salts (3a) towards Thioether (4a) Synthesis:

Optimization of Reaction Conditions: [a], [b]

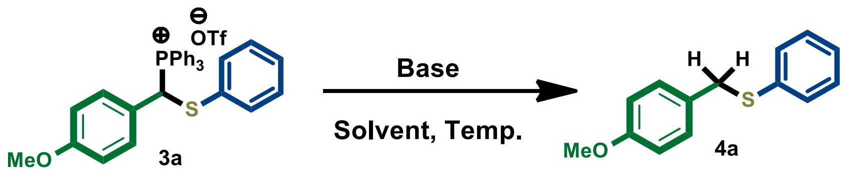

Entry	Base	Solvent (0.07 M)	Temperature (˚C)	Time (h)	Yield **4a** (%)
1	DBU	THF	rt	40	85
2	BABCO	THF	rt	40	48
3	DMAP	THF	rt	40	<5
4	Et_3N	THF	rt	40	<5
5	Cs_2CO_3	THF	rt	40	32
6	KO^tBu	THF	rt	40	18
7	NaH	THF	rt	40	21
8	DBU	DMF	rt	40	61
9	DBU	MeCN	rt	40	39
10	DBU	DMSO	rt	40	35
11	DBU	EtOAc	rt	40	47
12	DBU	CH_2Cl_2	rt	40	85
13[c]	DBU	CH_2Cl_2	rt	40	<5
14[d]	DBU	CH_2Cl_2	rt	40	<5
15[e]	DBU	CH_2Cl_2	rt	40	18
16	DBU	CH_2Cl_2	45	20	80

17[f]	DBU	CH_2Cl_2	rt	40	87

Reaction condition: [a]All the reactions were performed in 0.1 mmol scale of thiophosphonium salt (**3a**) with 1.1 equiv. of Base and 1.5 mL solvent in each case. [b] All are isolated yields, rt = room temperature (22 ˚C – 23 ˚C). [c] 0.1 equiv. of Base was used. [d] 0.2 equiv. of Base was used. [e] 0.5 equiv. of Base was used. [f] 35 µL H_2O was added to the reaction. DBU = 1,8-Diazabicyclo(5.4.0)undec-7-ene, DMAP = 4-Dimethylaminopyridine, DABCO = 1,4-diazabicyclo[2.2. 2]octane.

Typical Procedure-2 for the Synthesis of Thioether (4) and Characterizations:

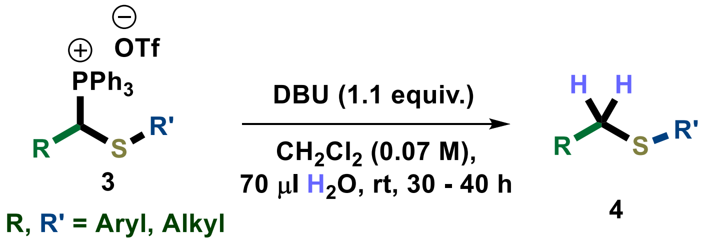

A 5 mL dry screw cap vial equipped with magnetic stir bar was sequentially charged with thiophosphonium salt **3** (0.2 mmol, 1 equiv.), 2 mL CH_2Cl_2, DBU (0.22 mmol, 1.1 equiv. in 1 mL CH_2Cl_2), 70 µL H_2O at room temperature. The vial was fitted with a Teflon cap and the reaction mixture was allowed to stir for the period of 30 h – 40 h. At the end of the reaction, solvent evaporated and purified by flash column chromatography by using n-hexane or 1: 99 (ethyl acetate: n-hexane) to 2: 98 (ethyl acetate: n-hexane) as an eluent. The synthesized thioethers (**4**) are characterized as given below, the data are identical with the literature.

Supplementary Table 2: Synthesis of Thioether (**4**) Products.

Entry	Substrate (3)	Time [h]	Product (4)	Yield [a], [b] [%]
1	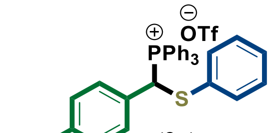 (3a)	40 h	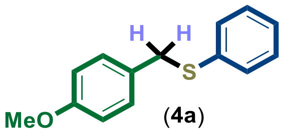 (4a)	87
2	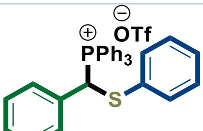 (3b)	40 h	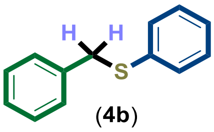 (4b)	91

**3**	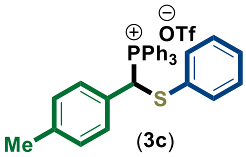 (3c)	40 h	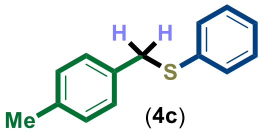 (4c)	77
**4**	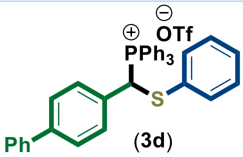 (3d)	38 h	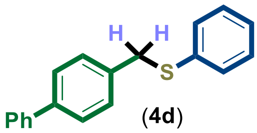 (4d)	54
**5**	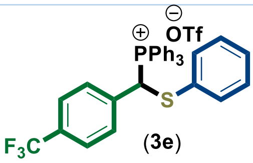 (3e)	35 h	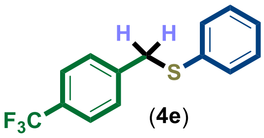 (4e)	59
**6**	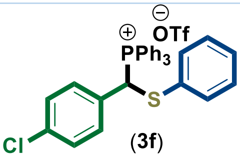 (3f)	40 h	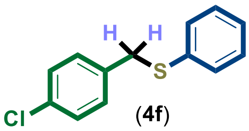 (4f)	62
**7**	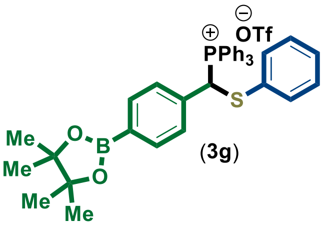 (3g)	35 h	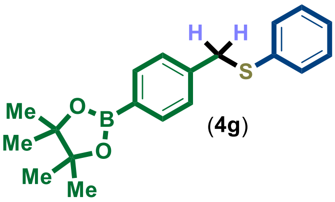 (4g)	28
**8**	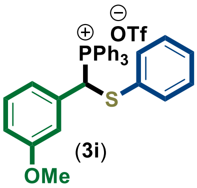 (3i)	40 h	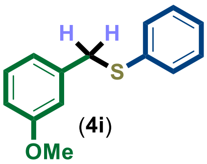 (4i)	85
**9**	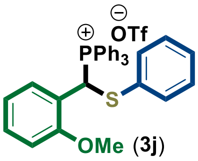 (3j)	40 h	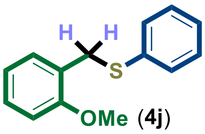 (4j)	78
**10**	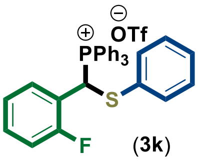 (3k)	34 h	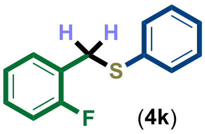 (4k)	94
**11**	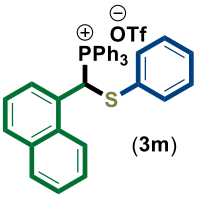 (3m)	32 h	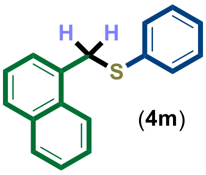 (4m)	55

12	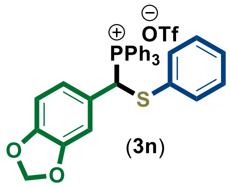 (3n)	33 h	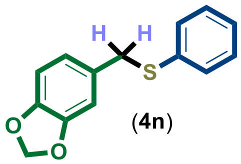 (4n)	71
13	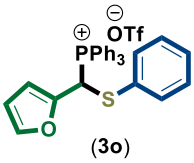 (3o)	30 h	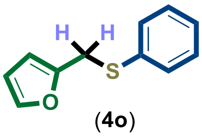 (4o)	72
14	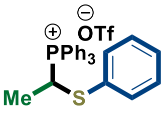 (3q)	40 h	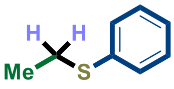 (4q)	62
15	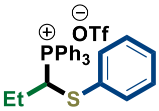 (3r)	40 h	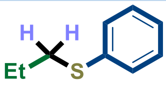 (4r)	71
16	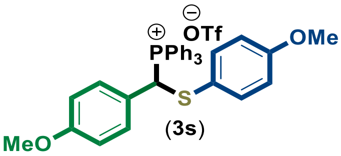 (3s)	39 h	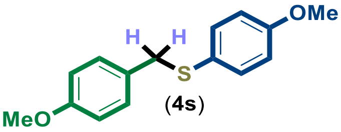 (4s)	81
17	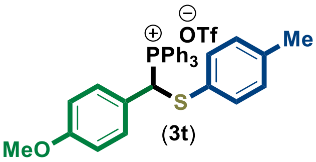 (3t)	40 h	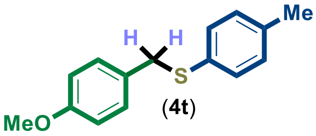 (4t)	84
18	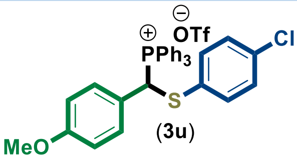 (3u)	40 h	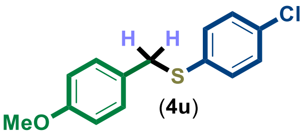 (4u)	76
19	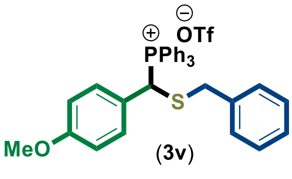 (3v)	33 h	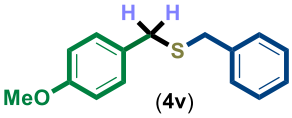 (4v)	82
20	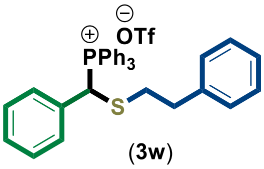 (3w)	40 h	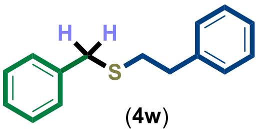 (4w)	92

**21**	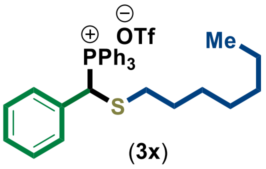 (**3x**)	40 h	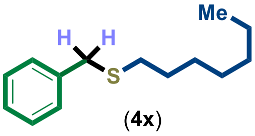 (**4x**)	85
**22**	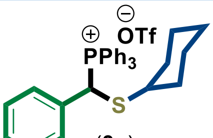 (**3y**)	32 h	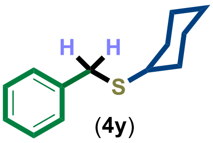 (**4y**)	66
**23**	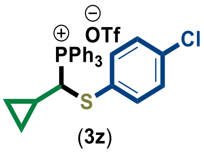 (**3z**)	35 h	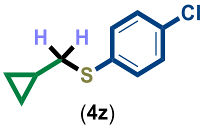 (**4z**)	56
**24**	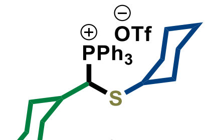 (**3aa**)	32 h	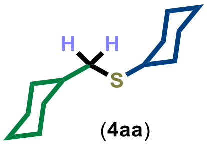 (**4aa**)	66
**25**	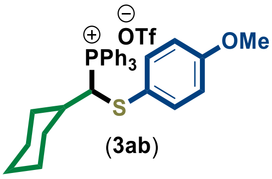 (**3ab**)	36 h	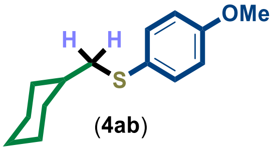 (**4ab**)	72
**26**	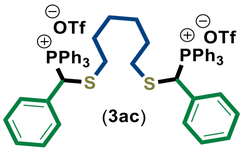 (**3ac**)	38 h	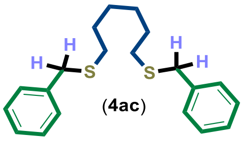 (**4ac**)	78
**27**	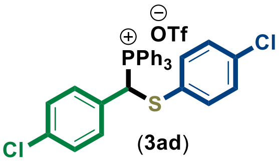 (**3ad**)	36 h	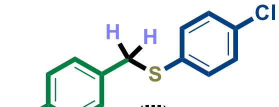 (**III**)	63

[a] All the reactions were performed in 0.2 mmol scale following the typical procedure-2. [b] Isolated yields

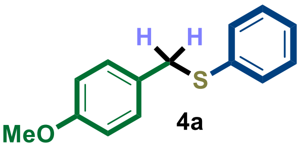

4a

(4-methoxybenzyl)(phenyl)sulfane (4a):[(2)]

Following Typical Procedure-2, thioether **4a** was isolated (87%, 40 mg), R_f = 0.57 (10% EtOAc in n-hexane) as a white solid; **^{1}H NMR (500 MHz, CDCl$_3$)** δ/ppm: 7.30 (ddd, J = 4.2, 3.3, 1.7

Hz, 2H), 7.28 – 7.23 (m, 2H), 7.23 – 7.15 (m, 3H), 6.85 – 6.79 (m, 2H), 4.08 (s, 2H), 3.79 (s, 3H); **^{13}C NMR (126 MHz, $CDCl_3$)** δ/ppm: 158.9, 136.7, 130.1, 129.9, 129.5, 129.0, 126.4, 114.0, 55.4, 38.6. **Note**: The NMR data were identical to those reported in the literature. (2)

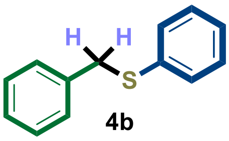

4b

Benzyl(phenyl)sulfane (4b): (3)

Following Typical Procedure-2, thioether **4b** was isolated (91%, 37 mg), R_f = 0.60 (10% EtOAc in n-hexane) as a white solid; **^{1}H NMR (400 MHz, $CDCl_3$)** δ/ppm: 7.31 – 7.26 (m, 6H), 7.26 – 7.21 (m, 3H), 7.19 – 7.14 (m, 1H), 4.11 (s, 2H); **^{13}C NMR (100 MHz, $CDCl_3$)** δ/ppm: 137.6, 136.5, 130.0, 129.0, 128.9, 128.6, 127.3, 126.5, 39.2. **Note**: The NMR data were identical to those reported in the literature. (3)

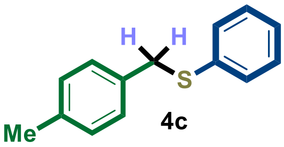

4c

(4-methylbenzyl)(phenyl)sulfane (4c): (3)

Following Typical Procedure-2, thioether **4c** was isolated (77%, 33 mg), R_f = 0.58 (10% EtOAc in n-hexane) as a white solid; **^{1}H NMR (400 MHz, $CDCl_3$)** δ/ppm: 7.32 – 7.28 (m, 2H), 7.27 – 7.21 (m, 2H), 7.20 – 7.14 (m, 3H), 7.08 (dd, J = 8.3, 0.6 Hz, 2H), 4.08 (s, 2H), 2.31 (s, 3H); **^{13}C NMR (100MHz, $CDCl_3$)** δ/ppm: 136.9, 136.8, 134.4, 129.7, 129.3, 128.9, 128.8, 126.3, 38.8, 21.2. **Note**: The NMR data were identical to those reported in the literature. (3)

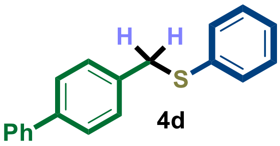

4d

([1,1'-biphenyl]-4-ylmethyl)(phenyl)sulfane (4d): (3)

Following Typical Procedure-2, thioether **4d** was isolated (54%, 30 mg), R_f = 0.33 (10% EtOAc in n-hexane) as a white solid; **^{1}H NMR (400 MHz, $CDCl_3$)** δ/ppm: 7.58 – 7.55 (m, 2H), 7.53 – 7.50 (m, 2H), 7.48 – 7.39 (m, 2H), 7.39 – 7.30 (m, 5H), 7.30 – 7.23 (m, 2H), 7.22 – 7.13 (m, 1H), 4.15 (s, 2H); **^{13}C NMR (101 MHz, $CDCl_3$)** δ/ppm: 140.8, 140.2, 136.7, 136.5, 130.0, 129.4, 129.0, 128.9, 127.4, 127.4, 127.2, 126.5, 38.9. **Note**: The NMR data were identical to those reported in the literature. (3)

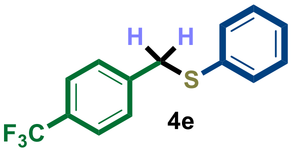
4e

Phenyl(4-(trifluoromethyl)benzyl)sulfane (4e): [(3)]

Following Typical Procedure-2, thioether **4e** was isolated (59%, 32 mg), R_f = 0.50 (10% EtOAc in n-hexane) as a colorless oil; **^{1}H NMR (400 MHz, CDCl$_3$)** δ/ppm: 7.55 (d, J = 8.0 Hz, 2H), 7.39 (d, J = 8.0 Hz, 2H), 7.33 – 7.23 (m, 5H), 4.15 (s, 2H); **^{13}C NMR (100 MHz, CDCl$_3$)** δ/ppm: 142.0, 135.5, 130.6, 129.5 (q, J = 32.7 Hz), 129.2, 129.1, 127.0, 125.5(q, J = 3.8 Hz), 124.2 (q, J = 271.0 Hz), 39.0; **^{19}F NMR (376 MHz, CDCl$_3$)** δ/ppm: -62.5. **Note**: The NMR data were identical to those reported in the literature. [(3)]

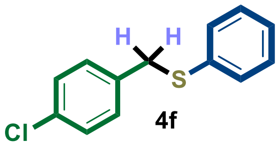
4f

(4-chlorobenzyl)(phenyl)sulfane (4f): [(3)]

Following Typical Procedure-2, thioether **4f** was isolated (62%, 29 mg), R_f = 0.58 (10% EtOAc in n-hexane) as a white solid; **^{1}H NMR (400 MHz, CDCl$_3$)** δ/ppm: 7.31 – 7.16 (m, 9H), 4.06 (s, 1H); **^{13}C NMR (100 MHz, CDCl$_3$)** δ/ppm: 136.3, 135.8, 133.1, 130.4, 130.2, 129.0, 128.7, 126.8, 38.7. **Note**: The NMR data were identical to those reported in the literature. [(3)]

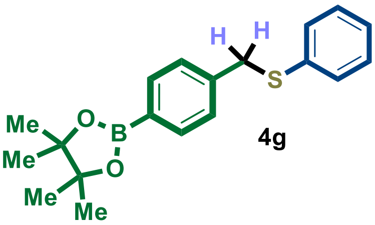
4g

4,4,5,5-tetramethyl-2-(4-((phenylthio)methyl)phenyl)-1,3,2-dioxaborolane (4g):

Following Typical Procedure-2, thioether **4g** was isolated (28%, 19 mg), R_f = 0.36 (10% EtOAc in n-hexane) as a white solid; **^{1}H NMR (400 MHz, CDCl$_3$)** δ/ppm: 7.74 – 7.72 (m, 2H), 7.31 – 7.28 (m, 4H), 7.26 – 7.22 (m, 2H), 7.19 – 7.15 (m, 1H), 4.11 (s, 2H), 1.34 (s, 12H); **^{13}C NMR (100 MHz, CDCl$_3$)** δ/ppm: 140.9, 136.3, 135.1, 130.1, 129.2, 129.0, 128.3, 126.5, 83.9, 39.3, 25.0; **^{11}B NMR (128 MHz, CDCl$_3$)** δ/ppm: 31.1; **HRMS** [APCI] was calculated for $[C_{19}H_{24}BO_2S]^+$ $[M + H]^+$: m/z 327.1585, found = 327.1573.

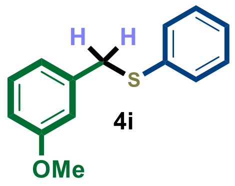

4i

(3-methoxybenzyl)(phenyl)sulfane (4i): (2)

Following Typical Procedure-2, thioether **4i** was isolated (85%, 39 mg), R_f = 0.40 (10% EtOAc in n-hexane) as a colorless liquid; **^{1}H NMR (400 MHz, CDCl$_3$)** δ/ppm: 7.33 – 7.30 (m, 2H), 7.28 – 7.25 (m, 1H), 7.22 – 7.16 (m, 1H), 6.90 – 6.87 (m, 1H), 6.84 – 6.83 (m, 1H), 6.78 (ddd, J = 8.2, 2.6, 0.8 Hz, 1H), 4.10 (s, 2H), 3.76 (s, 3H); ^{13}C NMR (100 MHz, CDCl$_3$) δ/ppm: 159.8, 139.2, 136.5, 130.0, 129.6, 129.0, 126.5, 121.3, 114.3, 113.1, 55.3, 39.2. **Note**: The NMR data were identical to those reported in the literature. (2)

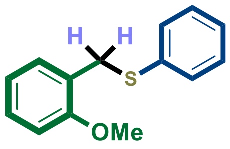

4j

(2-methoxybenzyl)(phenyl)sulfane (4j): (4)

Following Typical Procedure-2, thioether **4j** was isolated (78%, 36 mg), R_f = 0.48 (10% EtOAc in n-hexane) as a colorless liquid; **^{1}H NMR (400 MHz, CDCl$_3$)** δ/ppm: 7.27 – 7.24 (m, 2H), 7.19 – 7.07 (m, 5H), 6.80 – 6.76 (m, 2H), 4.08 (s, 2H), 3.74 (s, 3H); **^{13}C NMR (100 MHz, CDCl$_3$)** δ/ppm: 157.4, 137.1, 130.3, 130.0, 128.8, 128.6, 126.2, 125.9, 120.5, 110.7, 55.6, 33.4. **Note**: The NMR data were identical to those reported in the literature. (4)

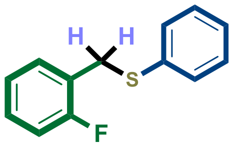

4k

(2-fluorobenzyl)(phenyl)sulfane (4k): (5)

Following Typical Procedure-2, thioether **4k** was isolated (94%, 41 mg), R_f = 0.73 (10% EtOAc in n-hexane) as a colorless liquid; **^{1}H NMR (400 MHz, CDCl$_3$)** δ/ppm: 7.34 – 7.31 (m, 2H), 7.28 – 7.23 (m, 3H), 7.22 – 7.17 (m, 2H), 7.05 – 6.99 (m, 2H), 4.13 (s, 3H); **^{13}C NMR (100 MHz, CDCl$_3$)** δ/ppm: 160.9 (d, J = 247.0 Hz), 135.8, 131.0 (d, J = 3.7 Hz), 130.7, 129.1, 129.0, 129.0, 126.8, 125.0 (d, J = 14.9 Hz), 124.2 (d, J = 3.6 Hz), 115.5 (d, J = 21.8 Hz), 32.3 (d, J =

3.3 Hz); **^{19}F NMR (376 MHz, CDCl$_3$)** δ/ppm: -117.8. **Note**: The NMR data were identical to those reported in the literature. (5)

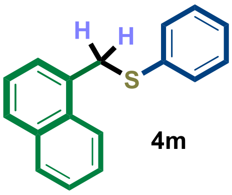

4m

(Naphthalen-1-ylmethyl)(phenyl)sulfane (4m): (5)

Following Typical Procedure-2, thioether **4m** was isolated (55%, 28 mg), R_f = 0.52 (10% EtOAc in n-hexane) as a light yellow solid,

^{1}H NMR (400 MHz, CDCl$_3$) δ/ppm: 8.17 – 8.14 (m, 1H), 7.88 – 7.86(m, 1H), 7.78 (dd, J = 8.6, 3.9 Hz, 1H), 7.58 – 7.49 (m, 2H), 7.36 – 7.34 (m, 4H), 7.29 – 7.25 (m, 2H), 7.23 – 7.19 (m, 1H), 4.57 (s, 2H); **^{13}C NMR (100 MHz, CDCl$_3$)** δ/ppm: 136.8, 134.1, 132.8, 131.6, 130.3, 129.0, 128.9, 128.4, 127.4, 126.6, 126.3, 125.9, 125.4, 124.0, 37.3. **Note**: The NMR data were identical to those reported in the literature. (5)

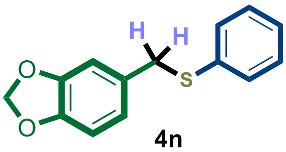

4n

5-((phenylthio)methyl)benzo[d][1,3]dioxole (4n): (2)

Following Typical Procedure-2, thioether **4n** was isolated (71%, 35 mg), R_f = 0.39 (10% EtOAc in n-hexane) as a colorless liquid; **^{1}H NMR (400 MHz, CDCl$_3$)** δ/ppm: 7.32 – 7.29 (m, 2H), 7.28 – 7.24 (m, 2H), 7.20 – 7.16 (m, 1H), 6.82 (m, 1H), 6.73 – 6.68 (m, 2H), 5.92 (s, 2H), 4.04 (s, 2H); **^{13}C NMR (100 MHz, CDCl$_3$)** δ/ppm: 147.8, 146.9, 136.4, 131.3, 130.0, 129.0, 126.5, 122.2, 109.3, 108.2, 101.1, 39.1. **Note**: The NMR data were identical to those reported in the literature. (2)

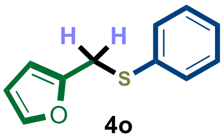

4o

2-((phenylthio)methyl)furan (4o): (6)

Following Typical Procedure-2, thioether **4o** was isolated (72%, 28 mg), R_f = 0.61 (10% EtOAc in n-hexane) as a colorless liquid; **^{1}H NMR (400 MHz, CDCl$_3$)** δ/ppm: 7.36 – 7.34 (m, 3H), 7.30 – 7.25 (m, 2H), 7.23 – 7.19 (m, 1H), 6.27 – 6.26 (m, 1H), 6.09 (dq, J = 3.2, 0.8 Hz, 1H),

4.10 (s, 2H); **^{13}C NMR (100 MHz, CDCl$_3$)** δ/ppm: 151.1, 142.3, 135.6, 130.7, 129.0, 126.9, 110.6, 108.0, 31.8. **Note**: The NMR data were identical to those reported in the literature. (6)

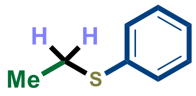

4q

Ethyl(phenyl)sulfane (4q): (3)

Following Typical Procedure-2, thioether **4q** was isolated (62%, 17 mg), R_f = 0.89 (10% EtOAc in n-hexane) as a colorless liquid; **^{1}H NMR (400 MHz, CDCl$_3$)** δ/ppm: 7.40 – 7.37 (m, 2H), 7.36 – 7.31 (m, 2H), 7.24 – 7.20 (m, 1H), 3.00 (q, J = 7.4 Hz, 2H), 1.37 (t, J = 7.4 Hz, 3H); **^{13}C NMR (100 MHz, CDCl$_3$)** δ/ppm: 136.8, 129.2, 129.0, 125.9, 27.8, 14.5. **Note**: The NMR data were identical to those reported in the literature. (3)

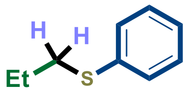

4r

Phenyl(propyl)sulfane (4r): (7)

Following Typical Procedure-2, thioether **4r** was isolated (71%, 22 mg), R_f = 0.83 (10% EtOAc in n-hexane) as a colorless liquid; **^{1}H NMR (400 MHz, CDCl$_3$)** δ/ppm: 7.39 – 7.36 (m, 2H), 7.32 (tt, J = 8.5, 1.3 Hz, 2H), 7.22 – 7.18 (m, 1H), 2.96 – 2.93 (m, 2H), 1.71 (dt, J = 14.6, 7.3 Hz, 2H), 1.07 (t, J = 7.4 Hz, 3H); **^{13}C NMR (100 MHz, CDCl$_3$)** δ/ppm: 137.1, 129.1, 128.9, 125.8, 35.7, 22.6, 13.6. **Note**: The NMR data were identical to those reported in the literature. (7)

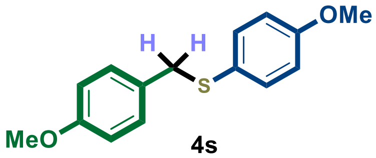

4s

(4-methoxybenzyl)(4-methoxyphenyl)sulfane (4s):[8]

Following Typical Procedure-2, thioether **4s** was isolated (81%, 42 mg), R_f = 0.38 (10% EtOAc in n-hexane) as a white solid; **^{1}H NMR (400 MHz, CDCl$_3$)** δ/ppm: 7.25 – 7.23 (m, 2H), 7.11 – 7.09 (m, 2H), 6.79 – 6.77 (m, 4H), 3.94 (s, 2H), 3.77 (s, 6H); **^{13}C NMR (100 MHz, CDCl$_3$)** δ/ppm: 159.2, 158.7, 134.1, 132.8, 130.2, 130.1, 126.4, 114.5, 113.9, 55.4, 55.3, 40.7. **Note**: The NMR data were identical to those reported in the literature. (7)

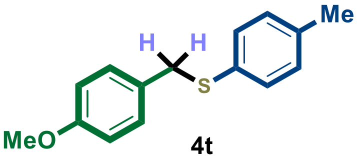

4t

(4-methoxybenzyl)(p-tolyl)sulfane (4t): [8]

Following Typical Procedure-2, thioether **4t** was isolated (84%, 41 mg), R_f = 0.48 (10% EtOAc in n-hexane) as a colorless liquid, **^{1}H NMR (400 MHz, CDCl$_3$)** δ/ppm: 7.24 – 7.18 (m, 4H), 7.10 – 7.06 (m, 2H), 6.84 – 6.81 (m, 2H), 4.04 (s, 2H), 3.79 (s, 3H), 2.32 (s, 3H); **^{13}C NMR (100 MHz, CDCl$_3$)** δ/ppm: 158.8, 136.5, 132.8, 130.7, 130.0, 129.9, 129.7, 114.0, 55.3, 39.3, 21.2. **Note**: The NMR data were identical to those reported in the literature. [8]

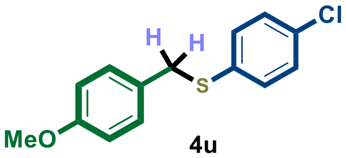

4u

(4-chlorophenyl)(4-methoxybenzyl)sulfane (4u): [9]

Following Typical Procedure-2, thioether **4u** was isolated (76%, 40 mg), R_f = 0.56 (10% EtOAc in n-hexane) as a white solid; **^{1}H NMR (400 MHz, CDCl$_3$)** δ/ppm: 7.20 (s, 4H), 7.19 – 7.15 (m, 2H), 6.83 – 6.79(m, 2H), 4.03 (s, 2H), 3.77 (s, 3H); **^{13}C NMR (100 MHz, CDCl$_3$)** δ/ppm: 159.0, 135.0, 132.5, 131.5, 130.0, 129.1, 129.0, 114.1, 55.4, 38.8. **Note**: The NMR data were identical to those reported in the literature. [9]

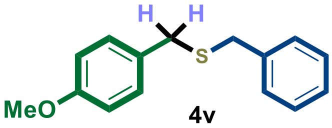

4v

Benzyl(4-methoxybenzyl)sulfane (4v): [10]

Following Typical Procedure-2, thioether **4v** was isolated (82%, 40 mg), R_f = 0.48 (10% EtOAc in n-hexane) as a colorless liquid; **^{1}H NMR (400 MHz, CDCl$_3$)** δ/ppm: 7.40 – 7.25 (m, 7H), 6.93 – 6.89 (m, 2H), 3.86 (s, 3H), 3.66 (s, 2H), 3.62 (s, 2H); **^{13}C NMR (100 MHz, CDCl$_3$)** δ/ppm: 158.7, 138.4, 130.2, 129.1, 128.6, 127.0, 114.0, 55.4, 35.6, 35.1. **Note**: The NMR data were identical to those reported in the literature. [10]

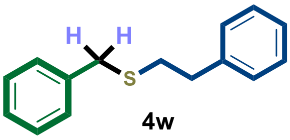

4w

Benzyl(phenethyl)sulfane (4w): (11)

Following Typical Procedure-2, thioether **4w** was isolated (92%, 40 mg), R_f = 0.67 (10% EtOAc in n-hexane) as a colorless liquid; **^{1}H NMR (400 MHz, CDCl$_3$)** δ/ppm: 7.36 – 7.17 (m, 10H), 3.75 (s, 2H), 2.87 (dd, J = 9.2, 6.5 Hz, 2H), 2.72 – 2.68 (m, 2H); **^{13}C NMR (100 MHz, CDCl$_3$)** δ/ppm: 140.7, 138.5, 129.0, 128.6, 128.6, 127.1, 126.4, 36.6, 36.1, 32.9. **Note**: The NMR data were identical to those reported in the literature. (11)

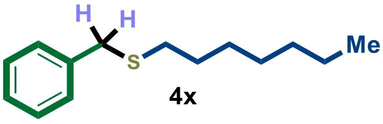

4x

Benzyl(heptyl)sulfane (4x): (12)

Following Typical Procedure-2, thioether **4w** was isolated (85%, 38 mg), R_f = 0.62 (10% EtOAc in n-hexane) as a colorless liquid; **^{1}H NMR (400 MHz, CDCl$_3$)** δ/ppm: 7.28 – 7.18 (m, 5H), 3.67 (s, 2H), 2.38 (dd, J = 8.0, 6.9 Hz, 2H), 1.56 – 1.49 (m, 2H), 1.26 – 1.21 (m, 8H), 0.85 (t, J = 6.9 Hz, 3H); **^{13}C NMR (100 MHz, CDCl$_3$)** δ/ppm: 138.8, 128.9, 128.5, 127.0, 36.4, 31.8, 31.5, 29.3, 29.0, 29.0, 22.7, 14.2. **Note**: The NMR data were identical to those reported in the literature. (12)

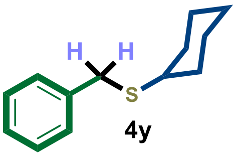

4y

Benzyl(cyclohexyl)sulfane (4y): (13)

Following Typical Procedure-2, thioether **4y** was isolated (66%, 27 mg), R_f = 0.70 (10% EtOAc in n-hexane) as a colorless liquid; **^{1}H NMR (400 MHz, CDCl$_3$)** δ/ppm: 7.38 – 7.31 (m, 4H), 7.29 – 7.26 (m, 1H), 3.78 (s, 2H), 2.63 – 2.57 (m, 1H), 2.00 – 1.96 (m, 2H), 1.80 – 1.76 (m, 2H), 1.63 – 1.62 (m, 1H), 1.42 – 1.25 (m, 5H); **^{13}C NMR (100 MHz, CDCl$_3$)** δ/ppm: 139.1, 128.9, 128.5, 126.9, 43.0, 34.7, 33.5, 26.1, 26.0. **Note**: The NMR data were identical to those reported in the literature. (13)

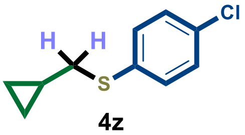

4z

(4-chlorophenyl)(cyclopropylmethyl)sulfane (4z): [14]

Following Typical Procedure-2, thioether **4z** was isolated (56%, 23 mg), R_f = 0.77 (10% EtOAc in n-hexane) as a colorless liquid; **^{1}H NMR (400 MHz, CDCl$_3$)** δ/ppm: 7.32 – 7.29 (m, 2H), 7.28 – 7.24 (m, 2H), 2.86 (d, J = 7.0 Hz, 2H), 1.10 – 1.00 (m, 1H), 0.62 – 0.57 (m, 2H), 0.28 – 0.24 (m, 2H); **^{13}C NMR (100 MHz, CDCl$_3$)** δ/ppm: 135.7, 132.0, 130.9, 129.0, 40.0, 10.7, 5.7.
Note: The NMR data were identical to those reported in the literature. [14]

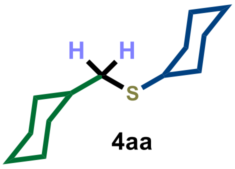

4aa

Cyclohexyl(cyclohexylmethyl)sulfane (4aa):

Following Typical Procedure-2, thioether **4aa** was isolated (66%, 28 mg), R_f = 0.73 (10% EtOAc in n-hexane) as a colorless liquid; **^{1}H NMR (400 MHz, CDCl$_3$)** δ/ppm: 2.59 – 1.54 (m, 1H), 2.41 (d, J = 6.9 Hz, 2H), 1.98 – 1.94 (m, 2H), 1.87 – 1.81 (m, 2H), 1.77 – 1.60 (m, 6H), 1.48 – 1.37 (m, 1H), 1.32 – 1.12 (m, 8H), 0.98 – 0.88 (m, 2H); **^{13}C NMR (100 MHz, CDCl$_3$)** δ/ppm: 44.2, 38.5, 37.9, 34.0, 33.1, 26.6, 26.3, 26.3, 26.0; **HRMS** [APCI] was calculated for $[C_{13}H_{25}S]^+$ $[M + H]^+$: m/z 213.1671, found = 213.1697.

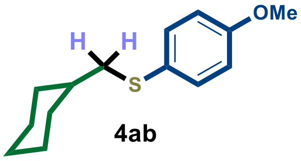

4ab

(Cyclohexylmethyl)(4-methoxyphenyl)sulfane (4ab):

Following Typical Procedure-2, thioether **4ab** was isolated (72%, 34 mg), R_f = 0.65 (10% EtOAc in n-hexane) as a colorless liquid; **^{1}H NMR (400 MHz, CDCl$_3$)** δ/ppm: 7.34 – 7.30 (m, 2H), 6.85 – 6.81 (m, 2H), 3.79 (s, 3H), 2.72 (d, J = 6.9 Hz, 2H), 1.89 – 1.85 (m, 2H), 1.73 – 1.62 (m, 3H), 1.52 – 1.41 (m, 1H), 1.27 – 1.09 (m, 3H), 1.01 – 0.91 (m, 2H); **^{13}C NMR (100 MHz, CDCl$_3$)** δ/ppm: 158.7, 132.6, 127.8, 114.6, 55.4, 43.4, 37.7, 32.8, 26.5, 26.2; **HRMS** [APCI] was calculated for $[C_{14}H_{21}OS]^+$ $[M + H]^+$: m/z 237.1308, found = 237.1332.

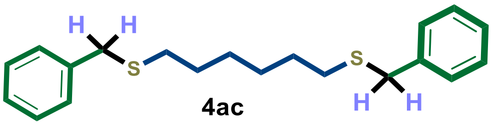

1,6-bis(benzylthio)hexane (4ac): [15]

Following Typical Procedure-2, thioether **4ac** was isolated (78%, 52 mg), R_f = 0.52 (10% EtOAc in n-hexane) as a colorless liquid; **^{1}H NMR (400 MHz, $CDCl_3$)** δ/ppm: 7.23 – 7.12 (m, 10H), 3.60 (s, 4H), 2.31 – 2.28 (m, 4H), 1.47 – 1.39 (m, 4H), 1.24 – 1.20 (m, 4H); **^{13}C NMR (100 MHz, $CDCl_3$)** δ/ppm: 138.8, 129.0, 128.6, 127.0, 36.4, 31.4, 29.2, 28.5. **Note**: The NMR data were identical to those reported in the literature. [15]

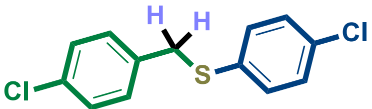

(4-chlorobenzyl)(4-chlorophenyl)sulfane (III): [16]

Following Typical Procedure-2, thioether **4ad** was isolated (63%, 34 mg), R_f = 0.64 (10% EtOAc in n-hexane) as a white solid; **^{1}H NMR (400 MHz, $CDCl_3$)** δ/ppm: 7.26 – 7.16 (m, 8H), 4.02 (s, 2H); **^{13}C NMR (100 MHz, $CDCl_3$)** δ/ppm: 135.9, 134.1, 133.3, 133.0, 132.0, 130.2, 129.2, 128.8, 39.0. **Note**: The NMR data were identical to those reported in the literature. [16]

Typical Procedure-3 for the Synthesis of α-Deuterated thioether (4-D) and Characterizations:

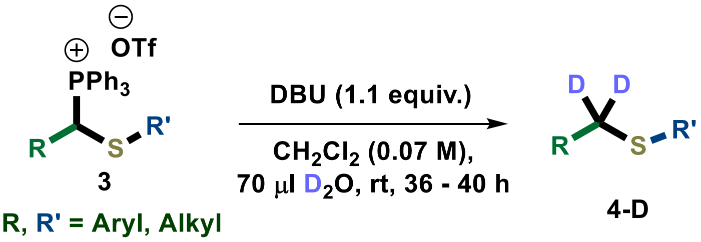

A 5 mL dry screw cap vial equipped with magnetic stir bar was sequentially charged with thiophosphonium salt **3** (0.2 mmol, 1 equiv.), 2 mL dry CH_2Cl_2, DBU (0.22 mmol, 1.1 equiv. in 1 mL dry CH_2Cl_2), 70 µL D_2O at room temperature. The vial was fitted with a Teflon cap, and the reaction mixture was allowed to stir for the period of 36 h – 40 h. At the end of the reaction, solvent evaporated and purified by flash column chromatography by using n-hexane

or 1: 99 (ethyl acetate: n-hexane) to 2: 98 (ethyl acetate: n-hexane) as an eluent. The synthesized deuterated thioethers (**4-D**) are characterized as given below, the data are identical with the literature.

Supplementary Table 3: Synthesis of α-Deuterated Thioether (**4-D**) Products.

**Entry**	**Substrate (3)**	**Time [h]**	**Product (4-D)**	**Yield[a], [b] [%]**
**1**	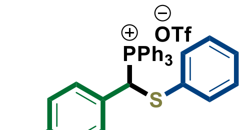 (3f)	40 h	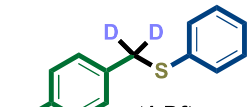 (4-Df)	79
**2**	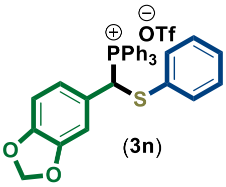 (3n)	40 h	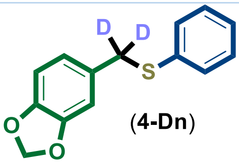 (4-Dn)	59
**3**	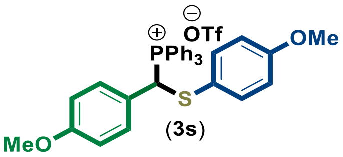 (3s)	39 h	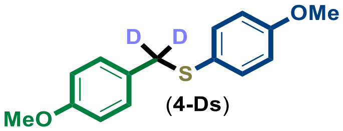 (4-Ds)	75
**4**	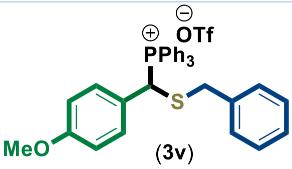 (3v)	39 h	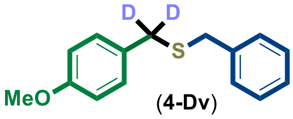 (4-Dv)	87
**5**	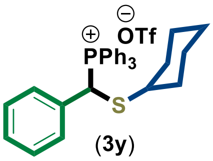 (3y)	36 h	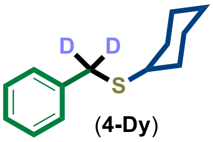 (4-Dy)	77
**6**	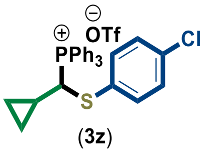 (3z)	36 h	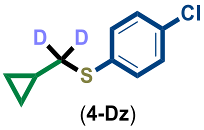 (4-Dz)	93
**7**	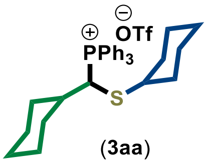 (3aa)	38 h	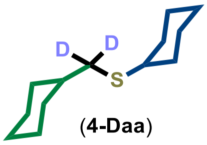 (4-Daa)	76

**8**	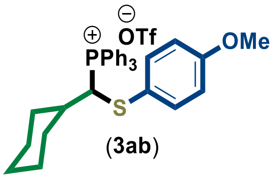 (3ab)	38 h	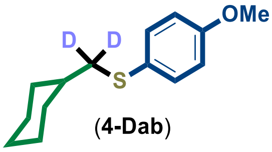 (4-Dab)	51

[a] All the reactions were performed in 0.2 mmol scale following the typical procedure-3. [b] Isolated yields. **Note:** The % D incorporation for all the deuterated molecules were determined by ^{1}H NMR

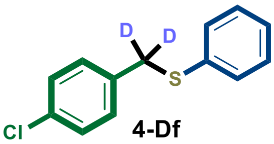

4-Df

((4-chlorophenyl)methyl-d2)(phenyl)sulfane (4-Df):

Following Typical Procedure-3, deuterated thioether **4-Df** was isolated (79%, 38 mg), R_f = 0.59 (10% EtOAc in n-hexane) as a white solid; **^{1}H NMR (400 MHz, CDCl$_3$)** δ/ppm: 7.32 – 7.20 (m, 9H), 4.06 (m, 0.2H, 90% labeled); **^{13}C NMR (100 MHz, CDCl$_3$)** δ/ppm: 136.1, 135.8, 133.1, 130.4, 130.2, 129.0, 128.7, 126.8, 38.7 – 37.9 (m, labeled, 1C). **HRMS** [APCI] was calculated for $[C_{13}H_9D_2ClS]^+$ $[M]^+$: m/z 236.0396, found = 236.0409. **Note**: For the compound **4-Df**, 90% D-incorporation was observed by ^{1}H NMR

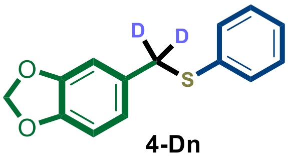

4-Dn

5-((phenylthio)methyl-d2)benzo[d][1,3]dioxole (4-Dn):

Following Typical Procedure-3, deuterated thioether **4-Dn** was isolated (59%, 29 mg), R_f = 0.47 (10% EtOAc in n-hexane) as a white solid; **^{1}H NMR (400 MHz, CDCl$_3$)** δ/ppm: 7.34 – 7.31 (m, 2H), 7.30 – 7.25 (m, 2H), 7.22 – 7.18 (m, 1H), 6.83 (dd, J = 1.5, 0.7 Hz, 1H), 6.75 – 6.60 (m, 2H), 5.94 (s, 2H), 4.04 (m, 0.22H, 89% labeled); **^{13}C NMR (100 MHz, CDCl$_3$)** δ/ppm: 147.8, 146.9, 136.4, 131.2, 130.0, 129.0, 126.5, 122.1, 109.3, 108.2, 101.1, 39.1 – 38.3 (m, labeled, 1C). **Note**: For the compound **4-Dn**, 89% D-incorporation was observed by ^{1}H NMR

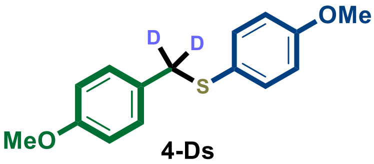

4-Ds

(4-methoxyphenyl)((4-methoxyphenyl)methyl-d2)sulfane (4-Ds):

Following Typical Procedure-3, deuterated thioether **4-Ds** was isolated (75%, 40 mg), R_f = 0.39 (10% EtOAc in n-hexane) as a white solid; **^{1}H NMR (400 MHz, CDCl$_3$)** δ/ppm: 7.26 – 7.23 (m, 2H), 7.12 – 7.08 (m, 2H), 6.80 – 6.77 (m, 2H), 3.94 (m, 0.24H, 88% labeled), 3.77 (s, 6H); **^{13}C NMR (100 MHz, CDCl$_3$)** δ/ppm: 159.2, 158.7, 134.1, 130.1, 130.1, 126.3, 114.5, 113.9, 55.4, 55.3, 40.7 – 39.9 (m, labeled, 1C); **^{2}H NMR (77 MHz, CDCl$_3$)** δ/ppm: 3.87; **HRMS** [APCI] was calculated for $[C_{15}H_{14}D_2O_2S]^+$ $[M]^+$: m/z 262.0996, found = 262.1039. **Note**: For the compound **4-Ds**, 88% D-incorporation was observed by ^{1}H NMR

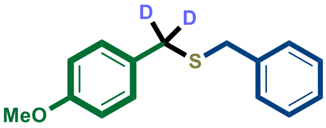

4-Dv

Benzyl((4-methoxyphenyl)methyl-d2)sulfane (4-Dv):

Following Typical Procedure-3, deuterated thioether **4-Dv** was isolated (87%, 43 mg), R_f = 0.53 (10% EtOAc in n-hexane) as a colorless oil; **^{1}H NMR (400 MHz, CDCl$_3$)** δ/ppm: 7.38 – 7.24 (m, 7H), 6.93 – 6.89 (m, 2H), 3.86 (s, 3H), 3.66 (s, 2H), 3.61 (s, 0.14H, 93% labeled); **^{13}C NMR (100 MHz, CDCl$_3$)** δ/ppm:158.7, 138.4, 130.2, 130.0, 129.1, 128.6, 127.0, 114.0, 55.4, 35.6, 35.0 – 34.1 (m, labeled, 1C); **HRMS** [APCI] was calculated for $[C_{15}H_{14}D_2OS]^+$ $[M]^+$: m/z 246.1047, found = 246.1087. **Note**: For the compound **4-Dv**, 93% D-incorporation was observed by ^{1}H NMR

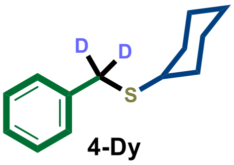

4-Dy

Cyclohexyl(phenylmethyl-d2)sulfane (4-Dy):

Following Typical Procedure-3, deuterated thioether **4-Dy** was isolated (77%, 32 mg), R_f = 0.66 (10% EtOAc in n-hexane) as a colorless oil; **^{1}H NMR (400 MHz, CDCl$_3$)** δ/ppm: 7.39 – 7.33 (m, 4H), 7.31 – 7.26 (m, 1H), 3.78 (m, 0.22H, 89% labeled), 2.64 – 2.59 (m, 1H), 2.01 – 1.98 (m, 2H), 1.80 – 1.78 (m, 2H), 1.65 – 1.61 (m, 1H), 1.38 – 1.28 (m, 5H); **^{13}C NMR (100**

MHz, $CDCl_3$) δ/ppm: 139.0, 128.9, 128.6, 126.9, 43.0, 34.7 – 33.9 (m, labeled, 1C), 33.5, 26.1, 26.0; **HRMS** [APCI] was calculated for $[C_{13}H_{17}D_2S]^+$ $[M+H]^+$: m/z 209.1328, found = 209.1334; **Note**: For the compound **4-Dy**, 89% D-incorporation was observed by ^{1}H NMR

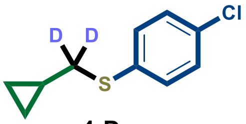

4-Dz

(4-chlorophenyl)(cyclopropylmethyl-d2)sulfane (4-Dz):

Following Typical Procedure-3, deuterated thioether **4-Dz** was isolated (93%, 38 mg), R_f = 0.72 (10% EtOAc in n-hexane) as a colorless oil; **^{1}H NMR (400 MHz, $CDCl_3$)** δ/ppm: 7.34 – 7.30 (m, 2H), 7.30 – 7.27 (m, 2H), 2.86 (dt, J = 7.0, 1.8 Hz, 0.24H, 88% labeled), 1.10 – 1.03 (m, 1H), 0.63 – 0.61 (m, 2H), 0.30 – 0.26 (m, 2H); **^{13}C NMR (100 MHz, $CDCl_3$)** δ/ppm: 135.7, 131.9, 130.8, 129.0, 40.0 – 39.1 (m, labeled, 1C), 10.5, 5.7; **HRMS** [APCI] was calculated for $[C_{10}H_{10}D_2ClS]^+$ $[M+H]^+$: m/z 201.0468, found = 201.0479; **Note**: For the compound **4-Dz**, 88% D-incorporation was observed by ^{1}H NMR

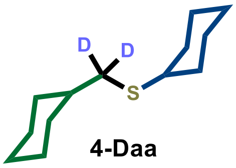

4-Daa

Cyclohexyl(cyclohexylmethyl-d2)sulfane (4-Daa):

Following Typical Procedure-3, deuterated thioether **4-Daa** was isolated (76%, 33 mg), R_f = 0.84 (10% EtOAc in n-hexane) as a colorless oil; **^{1}H NMR (400 MHz, $CDCl_3$)** δ/ppm: 2.58 – 2.54 (m, 1H), 2.40 – 2.38 (m, 0.26H, 87% labeled), 1.98 – 1.94 (m, 2H), 1.86 – 1.59 (m, 8H), 1.44– 1.42 (m, 1H), 1.39 – 1.15 (m, 8H), 0.98 – 0.86 (m, 2H); **^{13}C NMR (100 MHz, $CDCl_3$)** δ/ppm: 44.1, 38.3, 37.9 – 36.9 (m, labeled, 1C), 34.0, 33.0, 26.6, 26.3, 26.3, 26.0; **HRMS** [APCI] was calculated for $[C_{13}H_{23}D_2S]^+$ $[M+H]^+$: m/z 215.1797, found = 215.1819; **Note**: For the compound **4-Daa**, 87% D-incorporation was observed by ^{1}H NMR

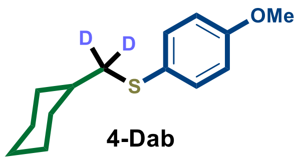

4-Dab

(Cyclohexylmethyl-d2)(4-methoxyphenyl)sulfane (4-Dab):

According to the above general procedure (C), deuterated thioether **4-Dab** was isolated (51%, 25 mg), R_f = 0.63 (10% EtOAc in n-hexane) as a colorless oil; **^{1}H NMR (400 MHz, $CDCl_3$)** δ/ppm: 7.34 – 7.30 (m, 2H), 6.85 – 6.81(m, 2H), 3.79 (s, 3H), 2.87 – 2.45 (m, 0.30H, 85% labeled), 1.90 – 1.84 (m, 2H), 1.74 – 1.59 (m, 3H), 1.49 – 1.43 (m, 1H), 1.27 – 1.10 (m, 3H), 1.01 – 0.89 (m, 2H); **^{13}C NMR (100 MHz, $CDCl_3$)** δ/ppm: 158.6, 132.6, 127.8, 114.6, 55.4, 43.4 – 42.3 (m, labeled, 1C), 37.5, 32.8, 26.5, 26.2; **HRMS** [APCI] was calculated for $[C_{14}H_{19}D_2OS]^+$ $[M+H]^+$: m/z 239.1433, found = 239.1461; **Note**: For the compound **4-Dab**, 85% D-incorporation was observed by ^{1}H NMR

Optimization for Oxidation of Thiophosphonium Salts (3) towards Thioester (5):

Optimization of Reaction Conditions: [a], [b]

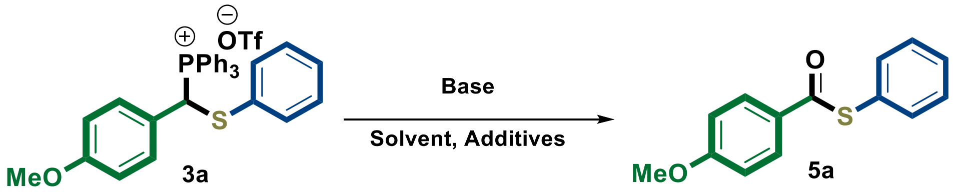

Entry	Base	Additives	Solvent [c] (0.07 M)	Yield (**5a**) (%)
1	NaH	None	THF	22
2	KO^{t}Bu	None	THF	NR
3	Cs_2CO_3	None	THF	45
4	K_3PO_4	None	THF	37
5	KOH	None	THF	ND
6	K_2HPO_4	None	THF	05

7	K_3PO_4	Air ballon	THF	90
8	Cs_2CO_3	Air ballon	THF	89
9	K_3PO_4	Air ballon	CH_2Cl_2	65
10	Cs_2CO_3	Air ballon	CH_2Cl_2	86
11	K_3PO_4	Air ballon	MeCN	87
12	Cs_2CO_3	Air ballon	MeCN	74

Reaction condition: [a] All the reactions were performed in 0.1 mmol scale of thiophosphonium salt (**3a**) with 1.2 equiv. of Base and 1.5 mL solvent in each case at room temperature for 36 h. [b] All are isolated yields, room temperature (22 ˚C – 23 ˚C). [c] All the solvents dried by over anh. MgSO4 before use.

Typical Procedure-4 for the Synthesis of Thioester (5) and Characterizations:

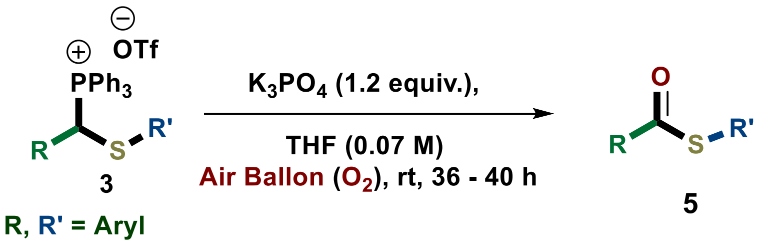

A 5 mL dry screw cap vial equipped with magnetic stir bar was sequentially charged with thiophosphonium salt **3** (0.2 mmol, 1 equiv.), K_3PO_4 (0.24 mmol, 1.2 equiv.), dry THF 3 mL at room temperature. The vial was fitted with a rubber septum, then an air balloon put on it via needle and the reaction mixture was allowed to stir for the period of 36 h – 40 h at room temperature. At the end of the reaction, solvent evaporated and purified by flash column chromatography by using 3: 97 (ethyl acetate: n-hexane) to 5: 95 (ethyl acetate: n-hexane) as an eluent. The synthesized thioesters (**5**) are characterized as given below; the data are identical with the literature.

Supplementary Table 4: Synthesis of Thioester (**5**) Products.

Entry	Substrate (3)	Time [h]	Product (5)	Yield [a], [b] [%]
**1**	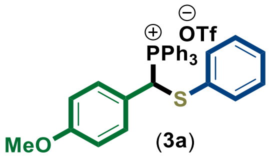	36 h	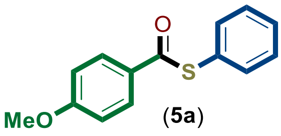	90

**2**	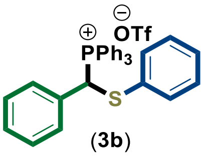 (**3b**)	38 h	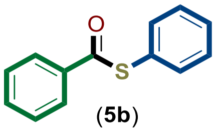 (**5b**)	65
**3**	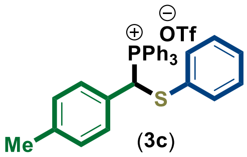 (**3c**)	38 h	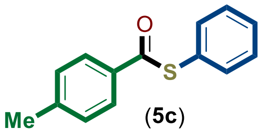 (**5c**)	62
**4**	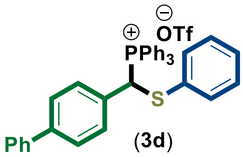 (**3d**)	40 h	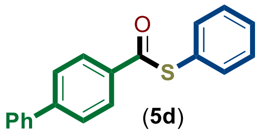 (**5d**)	76
**5**	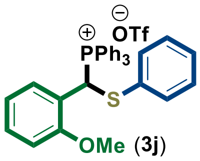 (**3j**)	40 h	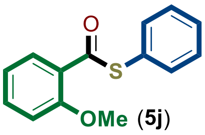 (**5j**)	59
**6**	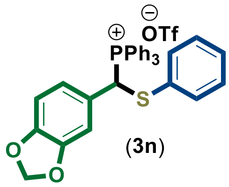 (**3n**)	38 h	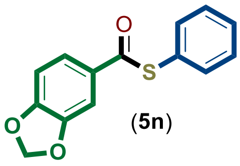 (**5n**)	55
**7**	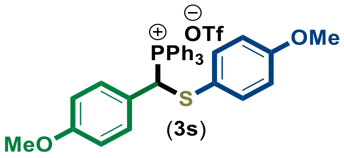 (**3s**)	39 h	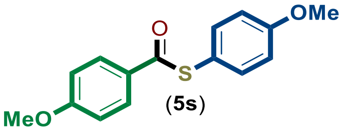 (**5s**)	66
**8**	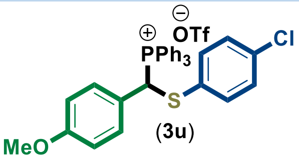 (**3u**)	39 h	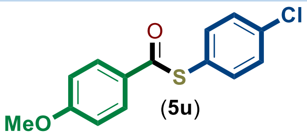 (**5u**)	53

[a] All the reactions were performed in 0.2 mmol scale following the typical procedure-4. [b] Isolated yields**.** (**Note**: This reaction is sensitive to moisture).

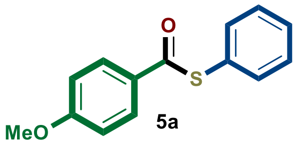

S-phenyl 4-methoxybenzothioate (5a): (16)

Following Typical Procedure-4, thioester **5a** was isolated (90%, 44 mg), R_f = 0.34 (10% EtOAc in n-hexane) as a white solid; **^{1}H NMR (400 MHz, CDCl₃)** δ/ppm: 8.09 – 7.92 (m, 2H), 7.55 – 7.50 (m, 2H), 7.48 – 7.40 (m, 3H), 7.03 – 6.90 (m, 2H), 3.88 (s, 3H); **^{13}C NMR (100MHz, CDCl₃)** δ/ppm: 188.7, 164.1, 135.3, 129.8, 129.5, 129.5, 129.3, 127.8, 114.0, 55.7; **Note**: The NMR data were identical to those reported in the literature. (16)

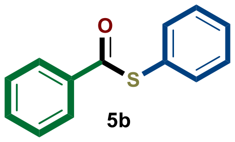

S-phenyl benzothioate (5b): (17)

Following Typical Procedure-4, thioester **5b** was isolated (65%, 28 mg), R_f = 0.54 (10% EtOAc in n-hexane) as a colorless solid; **^{1}H NMR (400 MHz, CDCl₃)** δ/ppm: 8.04 (dt, J = 8.5, 1.6 Hz, 2H), 7.65 – 7.59 (m, 1H), 7.56 – 7.44 (m, 7H); **^{13}C NMR (100 MHz, CDCl₃)** δ/ppm: 190.3, 136.8, 135.2, 133.8, 129.7, 129.4, 128.9, 127.6, 127.5; **Note**: The NMR data were identical to those reported in the literature. (17)

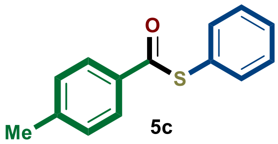

S-phenyl 4-methylbenzothioate (5c): (17)

Following Typical Procedure-4, thioester **5c** was isolated (62%, 28 mg), R_f = 0.57 (10% EtOAc in n-hexane) as a white solid; **^{1}H NMR (400 MHz, CDCl₃)** δ/ppm: 7.93 (d, J = 8.2 Hz, 2H), 7.55 – 7.44 (m, 5H)., 7.33 – 7.26 (m, 2H), 2.43 (s, 3H); **^{13}C NMR (100 MHz, CDCl₃)** δ/ppm: 189.9, 144.7, 135.3, 134.2, 129.6, 129.3, 127.7, 21.9; **Note**: The NMR data were identical to those reported in the literature. (17)

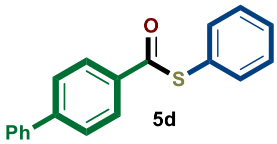

S-phenyl [1,1'-biphenyl]-4-carbothioate (5d): [18]

Following Typical Procedure-4, thioester **5d** was isolated (76%, 44 mg), R_f = 0.50 (10% EtOAc in n-hexane) as a white solid; **^{1}H NMR (400 MHz, CDCl$_3$)** δ/ppm: 8.13 – 8.07 (m, 2H), 7.75 – 7.68 (m, 2H), 7.68 – 7.62 (m, 2H), 7.60 – 7.52 (m, 2H), 7.52 – 7.45 (m, 5H), 7.45 – 7.39 (m, 1H); **^{13}C NMR (100 MHz, CDCl$_3$)** δ/ppm: 189.8, 146.6, 139.9, 135.5, 135.3, 129.7, 129.4, 129.1, 128.5, 128.2, 127.5, 127.4; **Note**: The NMR data were identical to those reported in the literature. [18]

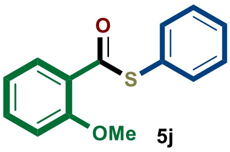

S-phenyl 2-methoxybenzothioate (5j): [17]

Following Typical Procedure-4, thioester **5j** was isolated (59%, 29 mg), R_f = 0.25 (10% EtOAc in n-hexane) as a colorless oil; **^{1}H NMR (400 MHz, CDCl$_3$)** δ/ppm: 7.86 – 7.77 (m, 1H), 7.63 – 7.35 (m, 6H), 7.19 – 6.93 (m, 2H), 3.97 (s, 3H); **^{13}C NMR (100 MHz, CDCl$_3$)** δ/ppm: 189.3, 158.3, 135.1, 134.1, 130.1, 129.4, 129.2, 128.9, 126.5, 120.7, 112.2, 56.0; **Note**: The NMR data were identical to those reported in the literature. [17]

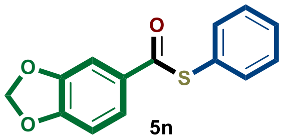

S-phenyl benzo[d][1,3]dioxole-5-carbothioate (5n): [18]

Following Typical Procedure-4, thioester **5n** was isolated (55%, 28 mg), R_f = 0.41 (10% EtOAc in n-hexane) as a white solid; **^{1}H NMR (400 MHz, CDCl$_3$)** δ/ppm: 7.69 (dd, J = 8.2, 1.8 Hz, 1H), 7.53 – 7.41 (m, 6H), 6.88 (d, J = 8.2 Hz, 1H), 6.07 (s, 2H); **^{13}C NMR (100 MHz, CDCl$_3$)** δ/ppm: 188.5, 152.4, 148.3, 135.3, 131.2, 129.6, 129.4, 127.6, 123.8, 108.3, 107.6, 102.2; **Note**: The NMR data were identical to those reported in the literature. [18]

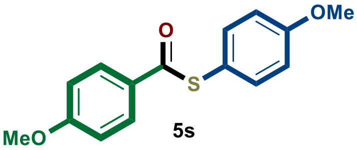

5s

S-(4-methoxyphenyl) 4-methoxybenzothioate (5s): (18)

Following Typical Procedure-4, thioester **5s** was isolated (66%, 36 mg), R_f = 0.22 (10% EtOAc in n-hexane) as a colorless crystal; **^{1}H NMR (400 MHz, CDCl$_3$)** δ/ppm: 8.02 – 7.98 (m, 2H), 7.45 – 7.38 (m, 2H), 7.00 – 6.97 (m, 2H), 6.97 – 6.93 (m, 2H)., 3.88 (s, 3H), 3.85 (s, 3H); **^{13}C NMR (100 MHz, CDCl$_3$)** δ/ppm: 189.6, 164.1, 160.8, 136.9, 129.8, 129.6, 118.3, 115.0, 114.0, 55.7, 55.5; **Note**: The NMR data were identical to those reported in the literature. (18)

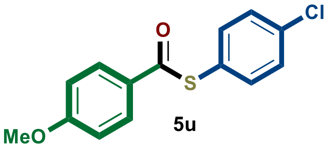

5u

S-(4-chlorophenyl) 4-methoxybenzothioate (5u): (19)

Following Typical Procedure-4, thioester **5u** was isolated (53%, 30 mg), R_f = 0.45 (10% EtOAc in n-hexane) as a light-yellow solid; **^{1}H NMR (400 MHz, CDCl$_3$)** δ/ppm: 8.01 – 7.92 (m, 2H), 7.50 – 7.36 (m, 4H), 7.04 – 6.92 (m, 2H), 3.89 (s, 3H); **^{13}C NMR (100 MHz, CDCl$_3$)** δ/ppm: 188.2, 164.3, 136.5, 136.0, 129.9, 129.6, 129.3, 126.3, 114.1, 55.7; **Note**: The NMR data were identical to those reported in the literature. (19)

Optimization for Oxidation of Thiophosphonium Salts (3) towards Dithioester (6)

Optimization of Reaction Conditions: [a], [b]

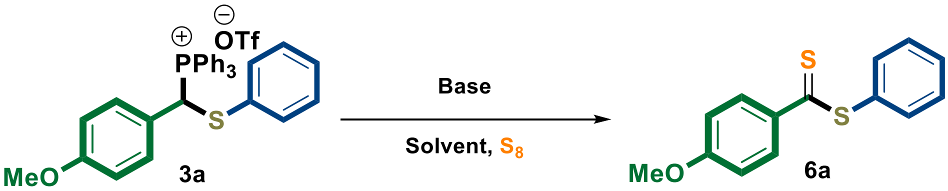

Entry	Base (1.2 equiv.)	S_8 (equiv.)	Solvent [c] (0.07 M)	Yield (**6a**) (%)
1	NaH	1.5	THF	38
2	KOtBu	1.5	THF	07

3	Cs_2CO_3	1.5	THF	35
4	K_3PO_4	1.5	THF	43
5	$EtN(^iPr)_2$	1.5	THF	10
6	DBU	1.5	THF	16
7	K_3PO_4	1.5	CH_2Cl_2	78
8	K_3PO_4	1.5	MeCN	42
9	K_3PO_4	1.5	1,4-dioxane	40
10	K_3PO_4	1.5	DMF	23
11	K_3PO_4	1.5	DMSO	51
12	K_3PO_4	2	CH_2Cl_2	68
13	K_3PO_4	3	CH_2Cl_2	69

Reaction condition: [a] All the reactions were performed in 0.1 mmol scale of thio-phosphonium salt (**3a**) with 1.2 equiv. of Base and 1.5 mL solvent in each case at room temperature. [b] Isolated yields, room temperature (22 ˚C – 23 ˚C). [c] All the solvents dried by over anh. MgSO4 before

Typical Procedure-5 for the Synthesis of Dithioesters (6) and Characterizations:

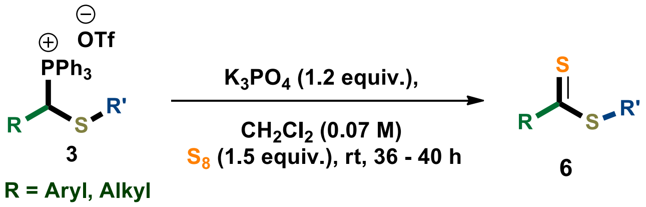

A 5 mL dry screw cap vial equipped with magnetic stir bar was sequentially charged with thiophosphonium salt **3** (0.2 mmol, 1 equiv.), K_3PO_4 (0.24 mmol, 1.2 equiv.), S_8 (0.3 mmol, 1.5 equiv.) and dry CH_2Cl_2 (3 mL) at room temperature. The vial was fitted with a Teflon cap, then the reaction mixture was allowed to stir for the period of 36 h – 40 h at room temperature. At the end of the reaction, solvent evaporated and purified by flash column chromatography by using 0.5: 99.5 (ethyl acetate: n-hexane) to 1: 99 (ethyl acetate: n-hexane) as an eluent. The synthesized dithioesters (**6**) are characterized as given below.

Supplementary Table 5: Synthesis of Dithioester (**6**) Products.

Entry	Substrate (3)	Time [h]	Product (6)	Yield [a], [b] [%]
**1**	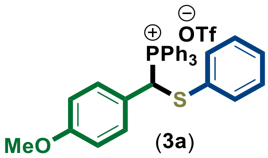 (3a)	40 h	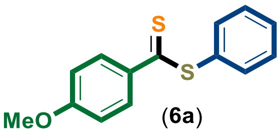 (6a)	78
**2**	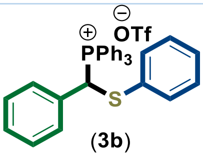 (3b)	40 h	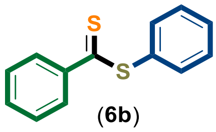 (6b)	69
**3**	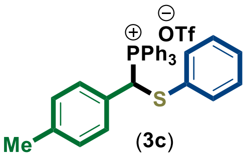 (3c)	40 h	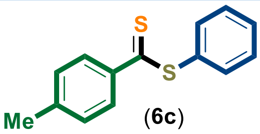 (6c)	71
**4**	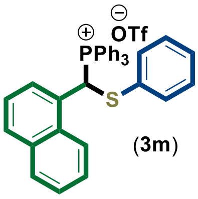 (3m)	39 h	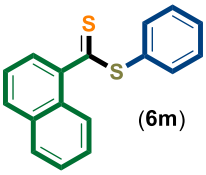 (6m)	68
**5**	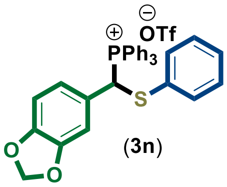 (3n)	38 h	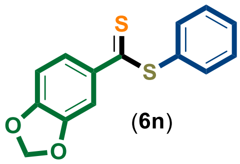 (6n)	72
**6**	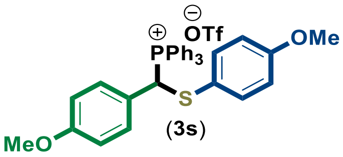 (3s)	39 h	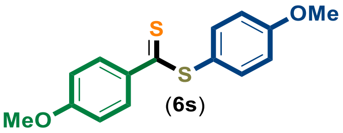 (6s)	80
**7**	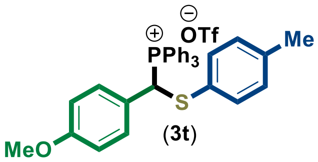 (3t)	40 h	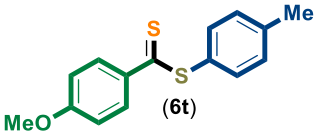 (6t)	82
**8**	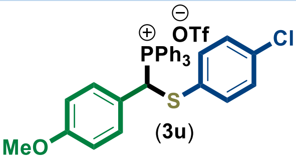 (3u)	40 h	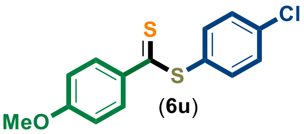 (6u)	65

**9**	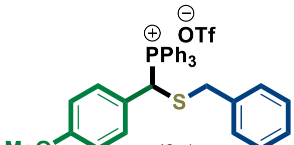 (3v)	37 h	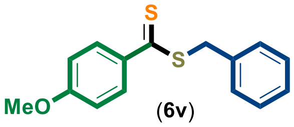 (6v)	76
**10**	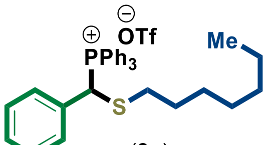 (3x)	40 h	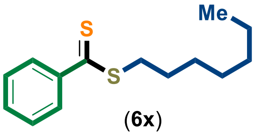 (6x)	76
**11**	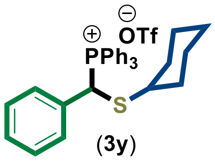 (3y)	39 h	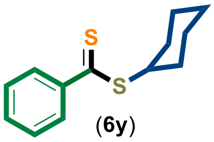 (6y)	79
**12**	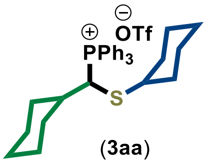 (3aa)	38 h	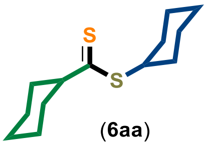 (6aa)	62
**13**	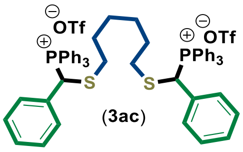 (3ac)	39 h	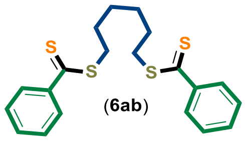 (6ab)	64

[a] All the reactions were performed in 0.2 mmol scale following the typical procedure-5. [b] Isolated yields**.**

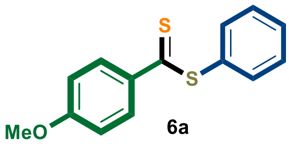

6a

Phenyl 4-methoxybenzodithioate (6a): [(20)]

Following Typical Procedure-5, dithioester **6a** was isolated (78%, 41 mg), R_f = 0.21 (5% EtOAc in n-hexane) as a red solid; **^{1}H NMR (400 MHz, CDCl$_3$)** δ/ppm: 8.22 – 8.11 (m, 2H), 7.59 – 7.46 (m, 5H), 6.97 – 6.87 (m, 2H), 3.89 (s, 3H); **^{13}C NMR (100 MHz, CDCl$_3$)** δ/ppm: 225.9, 164.0, 137.7, 135.8, 131.6, 130.3, 129.6, 129.4, 113.7, 55.7; **Note**: The NMR data were identical to those reported in the literature. [(20)]

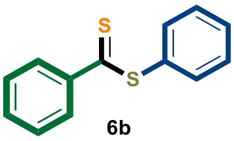

6b

Phenyl benzodithioate (6b): [21]

Following Typical Procedure-5, dithioester **6b** was isolated (69%, 32 mg), R_f = 0.25 (5% EtOAc in n-hexane) as a purple oil; **^{1}H NMR (400 MHz, $CDCl_3$)** δ/ppm: 8.11 – 8.06 (m, 2H), 7.57 (ddd, J = 7.0, 5.0, 3.5 Hz, 1H), 7.51 (dddd, J = 6.6, 3.9, 3.3, 1.8 Hz, 5H), 7.45 – 7.40 (m, 2H); **^{13}C NMR (100 MHz, $CDCl_3$)** δ/ppm: 228.6, 144.8, 135.5, 132.8, 131.5, 130.5, 129.8, 128.5, 127.1; **Note**: The NMR data were identical to those reported in the literature. [21]

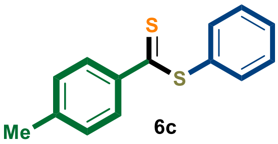

6c

Phenyl 4-methylbenzodithioate (6c): [20]

Following Typical Procedure-5, dithioester **6c** was isolated (71%, 35 mg), R_f = 0.42 (5% EtOAc in n-hexane) as a red solid; **^{1}H NMR (400 MHz, $CDCl_3$)** δ/ppm: 8.06 – 8.02 (m, 2H), 7.54 – 7.47 (m, 5H), 7.24 – 7.20 (m, 2H), 2.41 (s, 3H); **^{13}C NMR (100 MHz, $CDCl_3$)** δ/ppm: 228.0, 143.8, 142.3, 135.6, 131.6, 130.4, 129.7, 129.2, 127.2, 21.7; **Note**: The NMR data were identical to those reported in the literature. [20]

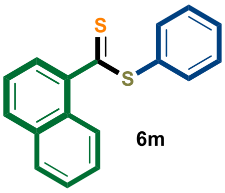

6m

Phenyl naphthalene-1-carbodithioate (6m): [22]

Following Typical Procedure-5, dithioester **6m** was isolated (68%, 38 mg), R_f = 0.33 (5% EtOAc in n-hexane) as a red solid; **^{1}H NMR (400 MHz, $CDCl_3$)** δ/ppm: 8.28 (dd, J = 6.9, 2.1 Hz, 1H), 7.94 – 7.82 (m, 2H), 7.63 (dd, J = 7.1, 1.1 Hz, 1H), 7.60 – 7.46 (m, 8H), **^{13}C NMR (100 MHz, $CDCl_3$)** δ/ppm: 232.0, 144.8, 135.0, 133.8, 131.6, 130.6, 130.3, 129.8, 129.4, 128.4, 127.1, 126.5, 125.1, 124.8, 124.1; **Note**: The NMR data were identical to those reported in the literature. [22]

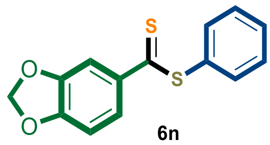
6n

Phenyl benzo[d][1,3]dioxole-5-carbodithioate (6n):

Following Typical Procedure-5, dithioester **6n** was isolated (72%, 40 mg), R_f = 0.23 (5% EtOAc in n-hexane) as a purple oil; **^{1}H NMR (400 MHz, CDCl$_3$)** δ/ppm: 7.85 (dd, J = 8.3, 1.9 Hz, 1H), 7.69 (d, J = 1.9 Hz, 1H), 7.53 – 7.46 (m, 5H), 6.83 (d, J = 8.3 Hz, 1H), 6.05 (s, 2H); **^{13}C NMR (100 MHz, CDCl$_3$)** δ/ppm: 225.7, 152.0, 148.1, 139.4, 135.7, 131.6, 130.4, 129.7, 122.6, 108.2, 107.7, 102.1; **HRMS** [APCI] was calculated for $[C_{14}H_{11}O_2S_2]^+$ $[M+H]^+$: m/z 275.0195, found = 275.0222.

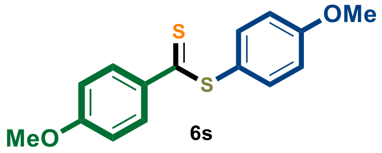
6s

4-methoxyphenyl 4-methoxybenzodithioate (6s):

Following Typical Procedure-5, dithioester **6s** was isolated (80%, 47 mg), R_f= 0.15 (5% EtOAc in n-hexane) as a red solid; **^{1}H NMR (400 MHz, CDCl$_3$)** δ/ppm: 8.20 – 8.16 (m, 2H), 7.42 – 7.34 (m, 2H), 7.05 – 6.99 (m, 2H), 6.94 – 6.87 (m, 2H), 3.88 (s, 3H), 3.87 (s, 3H); **^{13}C NMR (100 MHz, CDCl$_3$)** δ/ppm: 227.3, 163.9, 161.3, 137.7, 137.2, 129.4, 122.5, 115.3, 113.6, 55.7, 55.5; **HRMS** [APCI] was calculated for $[C_{15}H_{15}O_2S_2]^+$ $[M+H]^+$: m/z 291.0508, found = 291.0517.

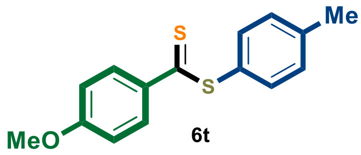
6t

p-tolyl 4-methoxybenzodithioate (6t): [23]

Following Typical Procedure-5, dithioester **6t** was isolated (82%, 45 mg), R_f= 0.25 (5% EtOAc in n-hexane) as a red solid; **^{1}H NMR (400 MHz, CDCl$_3$)** δ/ppm: 8.22 – 8.17 (m, 1H), 7.40 – 7.35 (m, 2H), 7.35 – 7.30 (m, 2H), 7.00 – 6.76 (m, 2H), 3.89 (s, 3H), 2.44 (s, 3H); **^{13}C NMR (100 MHz, CDCl$_3$)** δ/ppm: 226.5, 163.9, 140.7, 137.8, 135.5, 130.5, 129.3, 128.2, 113.6, 55.7, 21.7; **Note**: The NMR data were identical to those reported in the literature. [23]

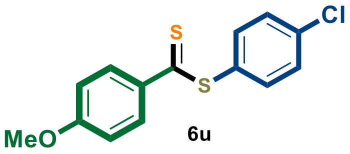

6u

4-Chlorophenyl 4-methoxybenzodithioate (6u):

Following Typical Procedure-5, dithioester **6u** was isolated (65%, 38 mg), R_f = 0.24 (5% EtOAc in n-hexane) as a red solid; **^{1}H NMR (400 MHz, CDCl$_3$)** δ/ppm: 8.20 – 8.16 (m, 2H), 7.49 – 7.45 (m, 2H), 7.40 (dt, J = 2.1, 1.3 Hz, 2H), 6.94 – 6.89 (m, 2H), 3.89 (s, 3H); **^{13}C NMR (100MHz, CDCl$_3$)** δ/ppm: 225.1, 164.1, 137.5, 137.1, 136.8, 130.0, 130.0, 129.4, 113.7, 55.7; **HRMS** [APCI] was calculated for $[C_{14}H_{12}ClOS_2]^+$ $[M+H]^+$: m/z 295.0013, found = 295.0017.

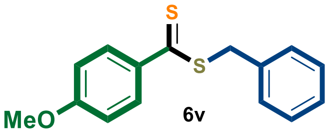

6v

Benzyl 4-methoxybenzodithioate (6v):

Following Typical Procedure-5, dithioester **6v** was isolated (76%, 42 mg), R_f = 0.22 (5% EtOAc in n-hexane) as a red solid; **^{1}H NMR (400 MHz, CDCl$_3$)** δ/ppm: 8.12 – 8.06 (m, 2H), 7.42 – 7.38 (m, 2H), 7.37 – 7.29 (m, 3H), 6.96 – 6.78 (m, 2H), 4.61 (s, 2H), 3.86 (s, 3H); **^{13}C NMR (100 MHz, CDCl$_3$)** δ/ppm: 225.3, 163.8, 137.9, 135.4, 129.4, 129.2, 128.8, 127.8, 113.6, 55.7, 42.1; **HRMS** [APCI] was calculated for $[C_{15}H_{15}OS_2]^+$ $[M+H]^+$: m/z 275.0559, found = 275.0577.

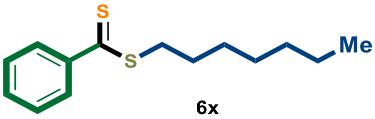

6x

Heptyl benzodithioate (6x):

Following Typical Procedure-5, dithioester **6x** was isolated (76%, 39 mg), R_f = 0.63 (5% EtOAc in n-hexane) as a reddish oil; **^{1}H NMR (400 MHz, CDCl$_3$)** δ/ppm: 7.99 (ddd, J = 6.8, 3.2, 1.6 Hz, 2H), 7.56 – 7.49 (m, 1H), 7.42 – 7.33 (m, 2H), 3.46 – 3.27 (m, 2H), 1.77 (dt, J = 15.1, 7.4 Hz, 2H), 1.52 – 1.42 (m, 2H), 1.42 – 1.24 (m, 6H), 0.90 (t, J = 6.9 Hz, 3H); **^{13}C NMR (100 MHz, CDCl$_3$)** δ/ppm: 229.0, 145.5, 132.3, 128.4, 127.0, 37.6, 31.8, 29.3, 29.0, 27.5, 22.7, 14.2; **HRMS** [APCI] was calculated for $[C_{14}H_{21}S_2]^+$ $[M+H]^+$: m/z 253.1079, found = 253.1100.

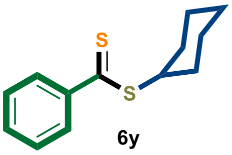

6y

Cyclohexyl benzodithioate (6y): [23]

Following Typical Procedure-5, dithioester **6y** was isolated (79%, 37 mg), R_f = 0.63 (5% EtOAc in n-hexane) as a red gel; **^{1}H NMR (400 MHz, CDCl$_3$)** δ/ppm: 7.99 – 7.94 (m, 2H), 7.57 – 7.47 (m, 1H), 7.43 – 7.32 (m, 2H), 4.04 (tt, J = 10.1, 3.7 Hz, 1H), 2.23 – 2.04 (m, 2H), 1.78 (ddd, J = 12.5, 8.4, 4.0 Hz, 2H), 1.73 – 1.46 (m, 5H), 1.37 (dddd, J = 13.0, 10.6, 7.1, 3.5 Hz, 1H); **^{13}C NMR (100 MHz, CDCl$_3$)** δ/ppm: 228.0, 145.6, 132.2, 128.3, 127.0, 49.7, 31.7, 26.1, 25.8; **Note**: The NMR data were identical to those reported in the literature. [23]

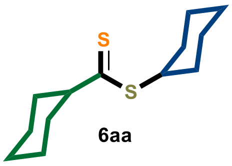

6aa

Cyclohexyl cyclohexanecarbodithioate (6aa):

Following Typical Procedure-5, dithioester **6aa** was isolated (62%, 30 mg), R_f = 0.86 (5% EtOAc in n-hexane) as a yellow oil; **^{1}H NMR (400 MHz, CDCl$_3$)** δ/ppm: 3.94 – 3.81 (m, 1H), 3.02 (tt, J = 11.5, 3.4 Hz, 1H), 2.04 – 1.96 (m, 2H), 1.92 – 1.77 (m, 4H), 1.76 – 1.58 (m, 7H), 1.54 – 1.39 (m, 3H), 1.39 – 1.22 (m, 4H); **^{13}C NMR (100 MHz, CDCl$_3$)** δ/ppm: 244.7, 60.7, 47.6, 34.8, 31.5, 26.3, 26.0, 25.8, 25.7; **HRMS** [APCI] was calculated for $[C_{13}H_{23}S_2]^+$ $[M+H]^+$: m/z 243.1236, found = 243.1261.

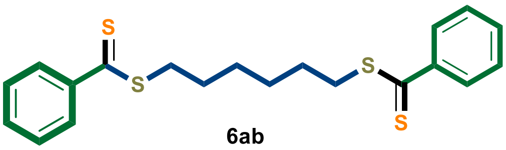

6ab

Hexane-1,6-diyl dibenzodithioate (6ab):

Following Typical Procedure-5, dithioester **6ab** was isolated (64%, 50 mg), R_f = 0.25 (5% EtOAc in n-hexane) as a red solid; **^{1}H NMR (400 MHz, CDCl$_3$)** δ/ppm: 8.01 – 7.96 (m, 4H), 7.52 (ddt, J = 7.0, 2.4, 0.9 Hz, 2H), 7.41 – 7.35 (m, 4H), 3.42 – 3.34 (m, 4H), 1.80 (p, J = 7.0 Hz, 4H), 1.66 – 1.48 (m, 4H); **^{13}C NMR (100 MHz, CDCl$_3$)** δ/ppm: 228.8, 145.4, 132.4, 128.4,

127.0, 37.2, 28.8, 27.3; **HRMS** [APCI] was calculated for $[C_{20}H_{23}S_4]^+$ $[M]^+$: m/z 391.0677, found = 391.0684.

NMR Studies towards Reaction Progress

<u>^{31}P NMR spectrum (162 MHz, CD_3CN) of the reaction progress to product **3a** *via* Intermediate-**A** and Intermediate-**B**:</u>

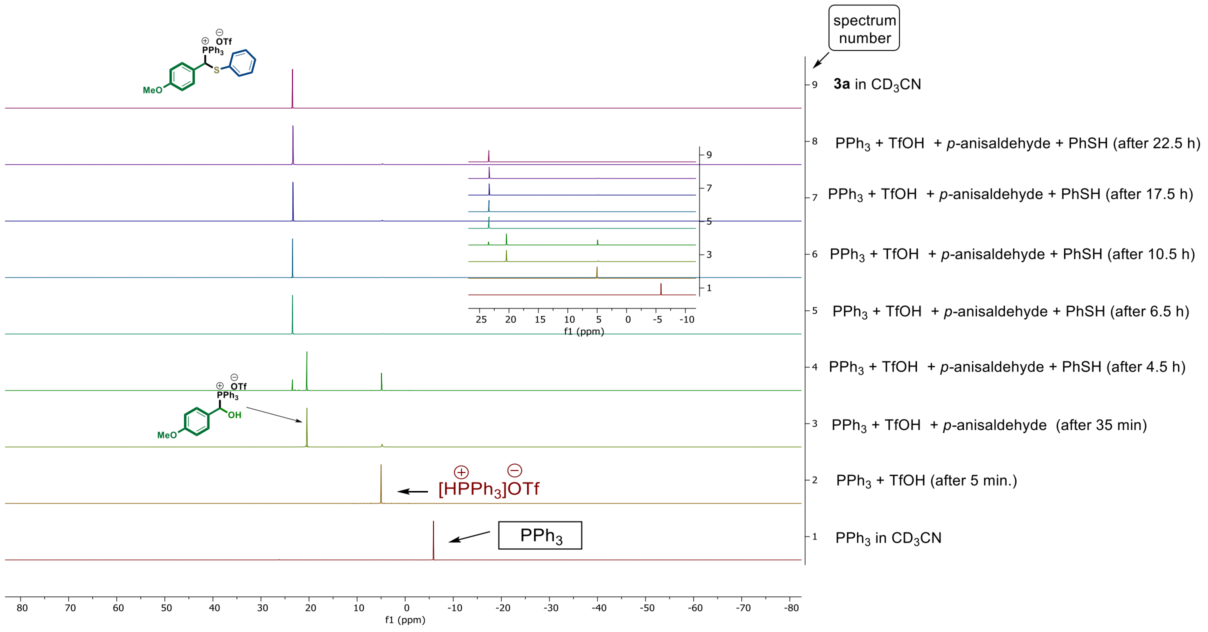

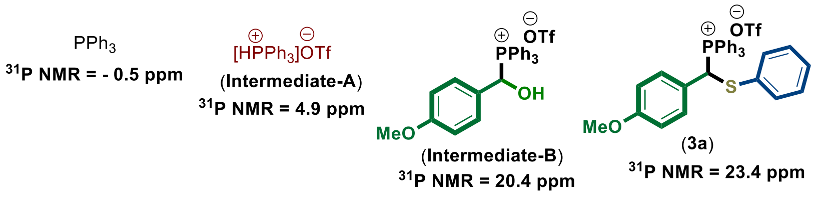

Discussion: After adding TfOH to PPh_3, the crude reaction mixture after 5 min showed that PPh_3 was completely converted to **Intermediate-A** signal (δ/ppm: 4.9) (spectra 1-2). Thirty minutes after the addition of aldehyde-**1a** to the crude mixture, the ^{31}P-NMR spectrum showed that >90% of the **Intermediate-A** signal was converted to a new ^{31}P-NMR signal **Intermediate-B** (δ/ppm: 20.4) (spectrum 3). Then, thiophenol **2a** was added to the reaction mixture and the reaction mixture was heated to 45 °C by oil-bath. After 4.5 h, the crude mixture exhibited the new ^{31}P-NMR signal at δ/ppm: 23.4 (salt **3a**) along with **Intermediate-B** and a small intensity of **Intermediate-A** (spectrum 4). Then, the reaction mixture was continued at 45 °C for 22.5 h. The ^{31}P-NMR reaction progress was recorded over time (spectra 4 - 8). We observed that after 6.5 h, **Intermediate-B** almost disappeared (spectrum 5). Up to 22.5 h, only

one phosphorous peak is observed the thiophosphonium salt **3a** ^{31}P-NMR signal (δ/ppm: 23.4) except very little intermediate-A observed as PPh_3 (1.1 equiv.) and TfOH (1.2 equiv.) used for each reaction.

<u>^{1}H NMR spectrum (400 MHz, CD_3CN) of the reaction progress to product **3a** *via* Intermediate-A and Intermediate-**B**:</u>

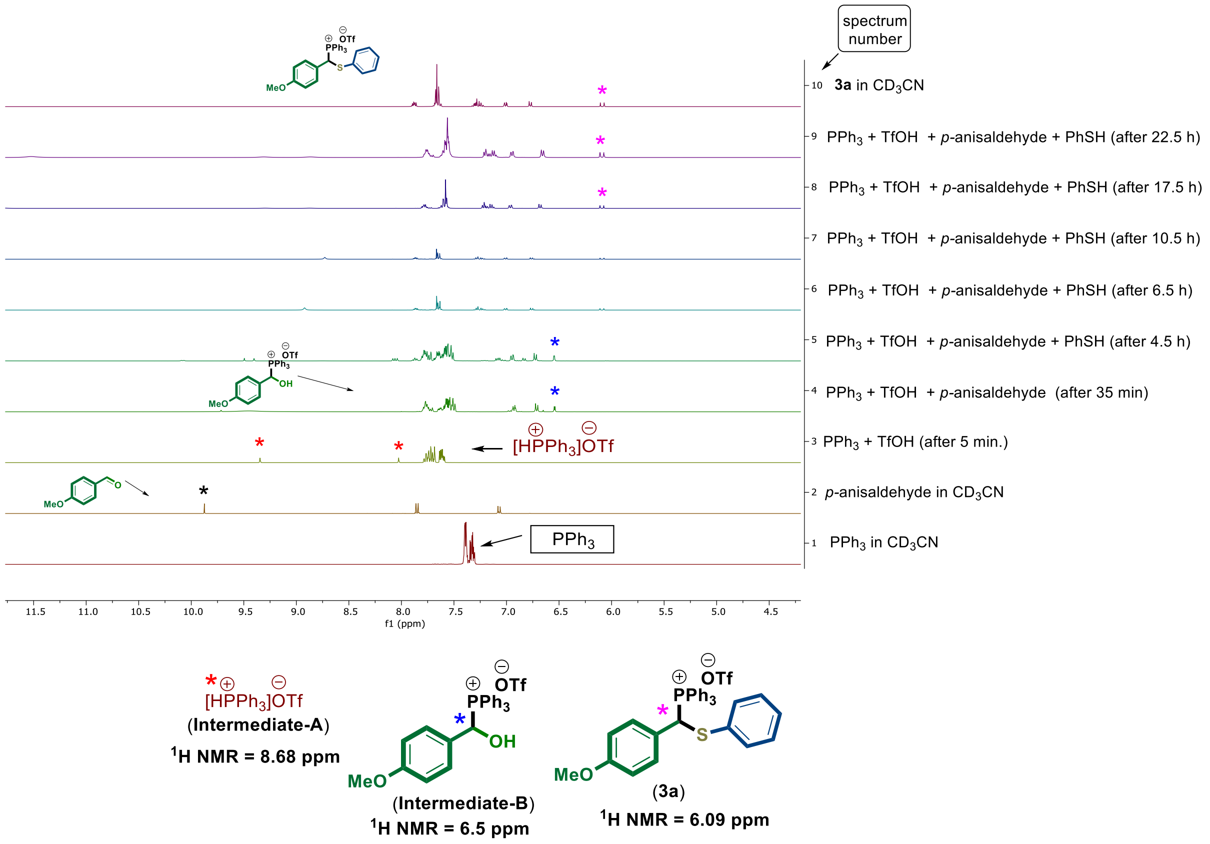

Discussion: After adding TfOH to PPh_3, the crude reaction mixture after 5 min showed that PPh_3 was completely converted to **Intermediate-A** signal (*δ*/ppm: 6.68) (spectra 1-3). Thirty minutes after the addition of aldehyde-**1a** to the crude mixture, the ^{1}H-NMR spectrum showed that >90% of **Intermediate-A** signal was converted to a new ^{1}H-NMR signal **Intermediate-B** (*δ*/ppm: 6.5) (spectrum 4). Then, thiophenol **2a** was added to the reaction mixture and the reaction mixture was heated to 45 °C by oil-bath. After 4.5 h, the crude mixture the disappearance of ^{1}H-NMR signal at δ/ppm: 6.5 observed and a small intensity of product **3a** observed (spectrum 6 -7). Then, the reaction mixture was continued at 45 °C for 22.5 h. The ^{1}H-NMR reaction progress was recorded over time (spectra 8 - 9). We observed ^{1}H NMR peak intensity correspond to product at *δ*/ppm: 6.09 (spectra 8 -9) increases. After 22.5 h the crude ^{1}H-NMR almost identical with the pure thiophosphonium salt **3a** (spectra 10).

^{31}P NMR spectrum (162 MHz, $CDCl_3$) of the reaction progress to thioether **4a**

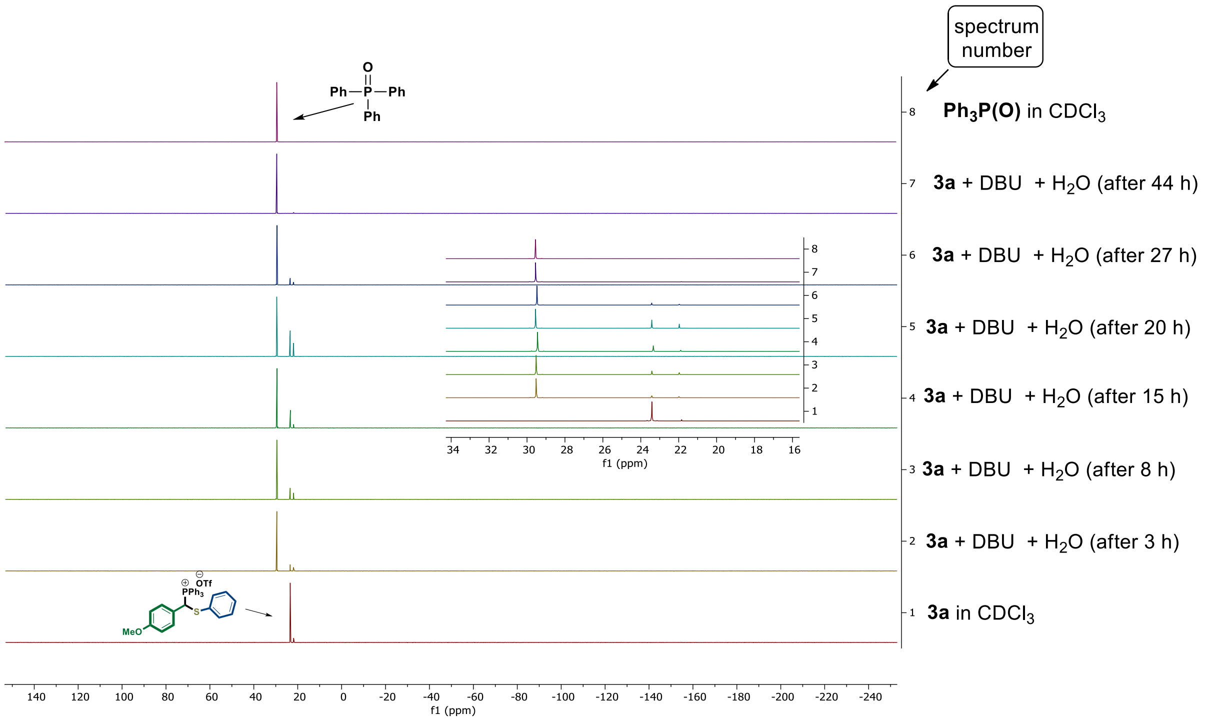

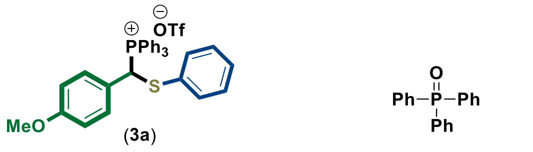

Discussion: According to the standard procedure, salt **3a**, base DBU, and H_2O have taken in a vial and stirred the reaction at room temperature, crude reaction mixture was sent for ^{31}P NMR to understand the reaction path. It is observed that after 3 h phosphorous peak corresponds to salt **3a (δ**/ppm: 24.2) almost disappeared and a new phosphorous peak at δ/ppm: 29.5 observed. It can be possible ylide (salt **3a**) very quickly converted to ylene which readily hydrolysed to produced triphenylphosphine oxide [$Ph_3P(O)$] as a side product with the desired product **4a**, i.e why intensity of the phosphorous peak δ/ppm: 29.5 increased over the time (spectra 3 – 7).

^{1}H NMR spectrum (400 MHz, $CDCl_3$) of the reaction progress to thioether **4a**

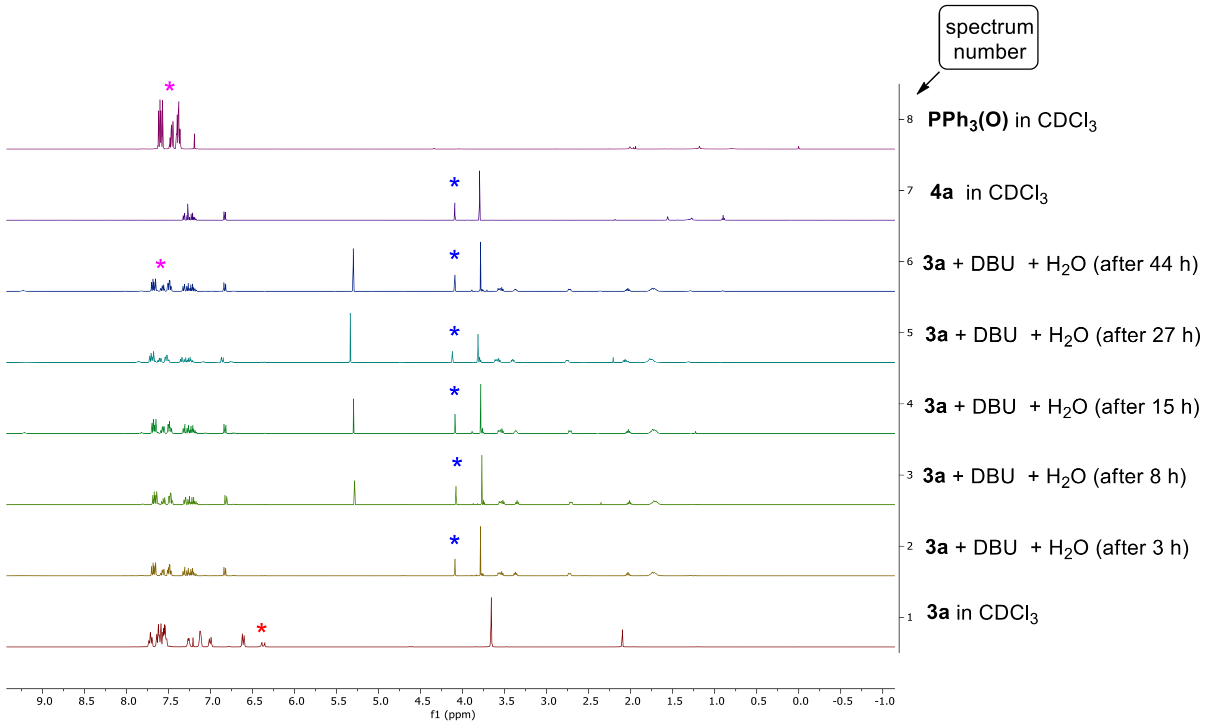

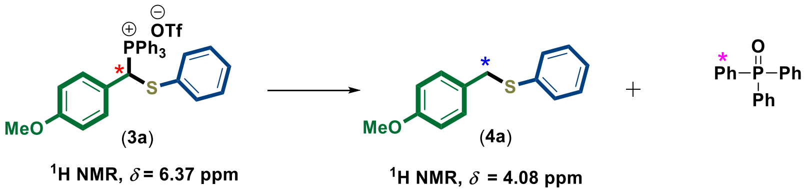

Discussion: According to the standard procedure, salt **3a**, base DBU, and H_2O have taken in a vial and stirred the reaction at room temperature, crude reaction mixture was sent for ^{1}H NMR to understand the reaction path. It is observed that after 3 h, proton peak corresponds to salt **3a** **(δ**/ppm: 6.37) disappeared and a new proton peak at δ/ppm: 4.08 was observed. It is the peak related to the methylene part of thioether **(4a)** and peak intensity gradually increase over the time (spectra 3 -6). By comparison with side product [$Ph_3P(O)$] the reaction is very clean and crude reaction contain only the mixture of desired product **4a** and side product [$Ph_3P(O)$] (spectra 6).

Reaction of salt 3a with *n*-BuLi followed by hydrolysis:

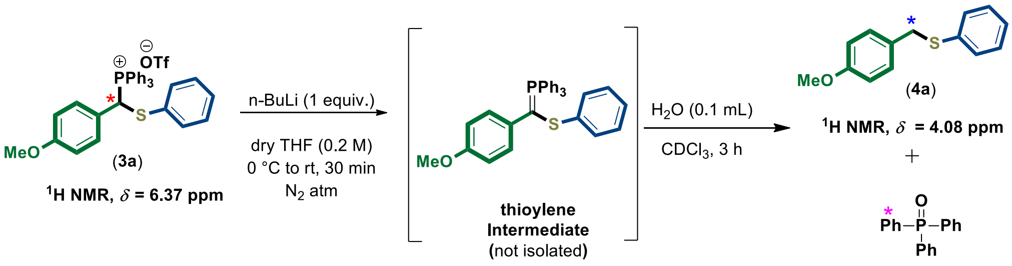

Crude reaction mixture after 30 min, ^{1}H NMR (400 MHz, $CDCl_3$):

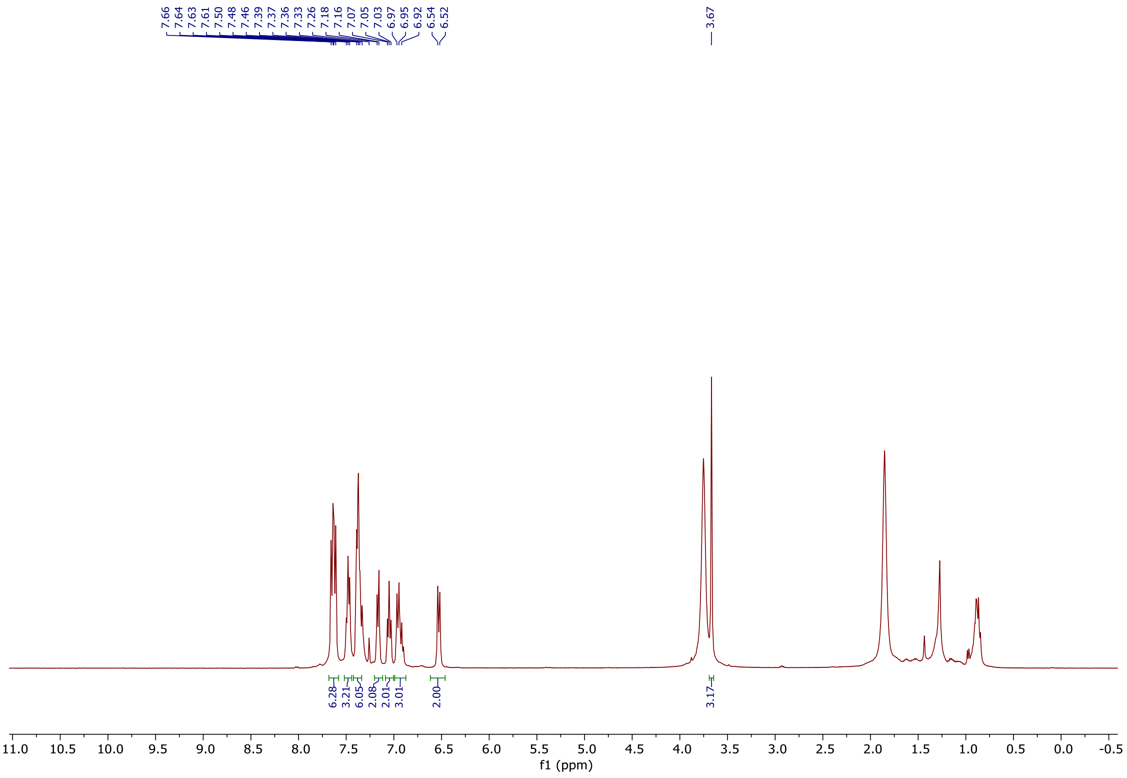

Crude reaction mixture after water addition (3 h), ^{1}H NMR (400 MHz, $CDCl_3$):

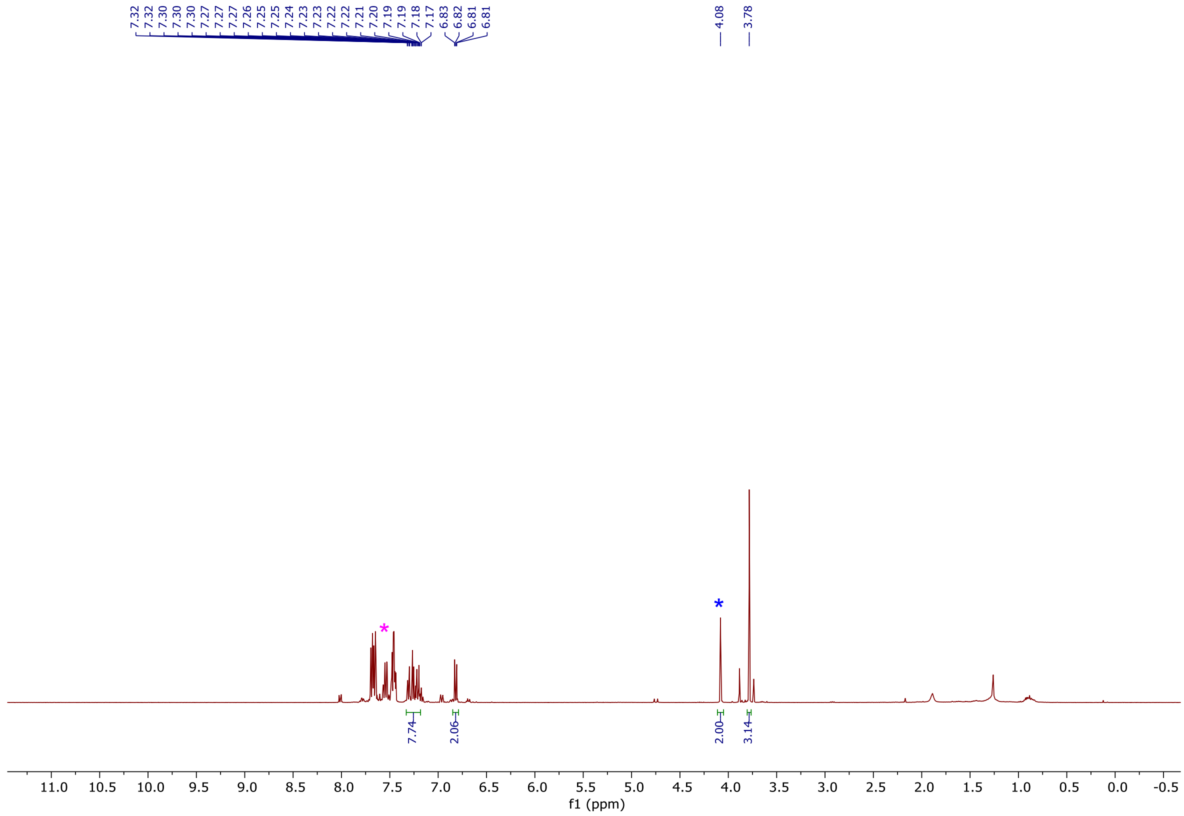

Dicussion: In an oven dried vial salt **3a** (0.1 mmol) was taken and fitted with rubber septum, then dry THF 0.5 mL was added to it, the reaction vial was kept in an ice bath (to maintain 0 °C), afterthat *n*-BuLi (0.1 mmol), was added to reaction dropwise, stirred the reaction for 30 min at room temperature, the reaction mixture was evaporated to dryness via rotavapor and send to NMR. After collecting NMR data, the $CDCl_3$ solution of reaction mixture was hydrolysed with 0.1 mL H_2O with 3h stirring at room temperature, NMR data of the crude reaction mixture was collected again. From the observation of ^1H NMR, we can assume that the peak at 4.08 ppm is for corresponding thioether **4a** which might be forming via thioylene intermediate through hydrolysis.

<u>Possible mechanism for thioester synthesis: [24]</u>

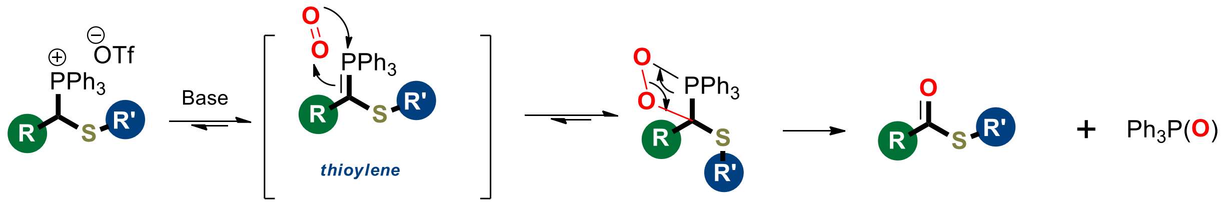

<u>Possible mechanism for dithioester synthesis:[25]</u>

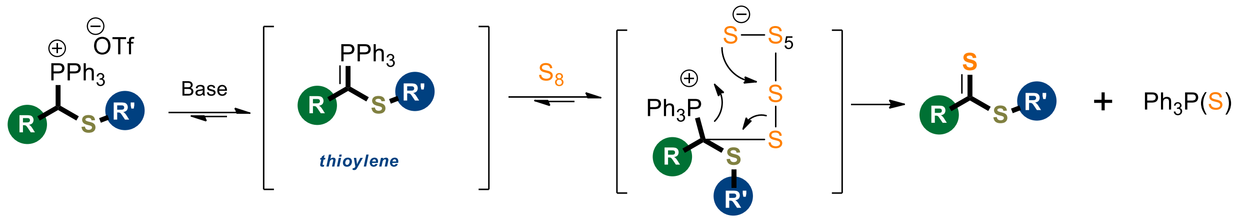

References:

(1) Babu, K. N.; Massarwe, F.; Shioukhi, I.; Masarwa, A. Sequential Selective C−H and C(sp3)−+P Bond Functionalizations: An Entry to Bioactive Arylated Scaffolds. *Angewandte Chemie International Edition* **2021**, *60* (50), 26199-26209. DOI: https://doi.org/10.1002/anie.202111164.

(2) Ranu, B. C.; Samanta, S.; Hajra, A. Unusual Cleavage of Ethers by Thiophenol on the Surface of Silica Gel Impregnated with Indium(III) Chloride under Microwave Irradiation: Efficient Procedure for the Synthesis of Thioethers through Transthioetherification. *Synlett* **2002**, *2002* (06), 0987-0989. DOI: 10.1055/s-2002-31911.

(3) Wu, F.; Wang, Y.; Qian, Y.; Xie, Z.-B.; Ke, Z.; Zhao, Y.; Liu, Z. A Green Route to Benzyl Phenyl Sulfide from Thioanisole and Benzyl Alcohol over Dual Functional Ionic Liquids. *Chemistry – An Asian Journal* **2023**, *18* (2), e202201078. DOI: https://doi.org/10.1002/asia.202201078.

(4) Villuendas, P.; Urriolabeitia, E. P. Ru-Catalyzed Regioselective CH-Hydroarylation of Alkynes with Benzylthioethers Using Sulfur as Directing Group. *Organic Letters* **2015**, *17* (12), 3178-3181. DOI: 10.1021/acs.orglett.5b01552.

(5) Liu, T.; Qiu, R.; Zhu, L.; Yin, S.-F.; Au, C.-T.; Kambe, N. Alkyl Sulfides as Promising Sulfur Sources: Metal-Free Synthesis of Aryl Alkyl Sulfides and Dialkyl Sulfides by Transalkylation of Simple Sulfides with Alkyl Halides. *Chemistry – An Asian Journal* **2018**, *13* (24), 3833-3837. DOI: https://doi.org/10.1002/asia.201801679.

(6) Fu, Y.; Su, Y.; Xu, Q.-s.; Du, Z.; Hu, Y.; Wang, K.-H.; Huang, D. CuI promoted sulfenylation of organozinc reagents with arylsulfonyl chlorides. *RSC Advances* **2017**, *7* (10), 6018-6022, 10.1039/C6RA27201K. DOI: 10.1039/C6RA27201K.

(7) Skolia, E.; Gkizis, P. L.; Kokotos, C. G. A sustainable photochemical aerobic sulfide oxidation: access to sulforaphane and modafinil. *Organic & Biomolecular Chemistry* **2022**, *20* (29), 5836-5844, 10.1039/D2OB01066F. DOI: 10.1039/D2OB01066F.

(8) Wang, G.; Gao, L.; Feng, Y.; Lin, L. Visible-Light-Activated Nickel Thiolates for C–S Couplings. *Organic Letters* **2023**, *25* (23), 4340-4344. DOI: 10.1021/acs.orglett.3c01474.

(9) Wang, Y.; Deng, J.; Chen, J.; Cao, F.; Hou, Y.; Yang, Y.; Deng, X.; Yang, J.; Wu, L.; Shao, X.; et al. Dechalcogenization of Aryl Dichalcogenides to Synthesize Aryl Chalcogenides via Copper Catalysis. *ACS Catalysis* **2020**, *10* (4), 2707-2712. DOI: 10.1021/acscatal.9b04931.

(10) Khezri, R.; Abbasi, M. Copper catalyzed reaction of alcohols, alkyl halides and Na2S2O3: an odorless and ligand-free route to unsymmetrical thioether synthesis. *New Journal of Chemistry* **2022**, *46* (33), 16172-16176, 10.1039/D2NJ03164G. DOI: 10.1039/D2NJ03164G.

(11) Shigeno, M.; Shishido, Y.; Hayashi, K.; Nozawa-Kumada, K.; Kondo, Y. KO-t-Bu Catalyzed Thiolation of β-(Hetero)arylethyl Ethers via MeOH Elimination/hydrothiolation. *European Journal of Organic Chemistry* **2021**, *2021* (28), 3932-3935. DOI: https://doi.org/10.1002/ejoc.202100597.

(12) Holland, H. L.; Brown, F. M.; Larsen, B. G. Biotransformation of organic sulfides-IV. Formation of chiral benzyl alkyl and phenyl alkyl sulfoxides by Helminthosporium species NRRL 4671. *Bioorganic & Medicinal Chemistry* **1994**, *2* (7), 647-652. DOI: https://doi.org/10.1016/0968-0896(94)85013-5.

(13) Grover, J.; Prakash, G.; Teja, C.; Lahiri, G. K.; Maiti, D. Metal-free photoinduced hydrogen atom transfer assisted C(sp3)–H thioarylation. *Green Chemistry* **2023**, *25* (9), 3431-3436, 10.1039/D3GC00359K. DOI: 10.1039/D3GC00359K.

(14) Li, F.; Pei, C.; Koenigs, R. M. Rhodium-Catalyzed Enamine Homologation of Sulfides with Triazoles as Carbene Precursor. *Organic Letters* **2020**, *22* (17), 6816-6821. DOI: 10.1021/acs.orglett.0c02330.

(15) Li, J.-R.; Du, M.; Bu, X.-H.; Zhang, R.-H. Extended chains via hydrogen bond linkages of dinuclear copper(II) and cadmium(II) complexes with a new flexible disulfoxide ligand.

Journal of Solid State Chemistry **2003**, *173* (1), 20-26. DOI: https://doi.org/10.1016/S0022-4596(03)00055-0.
(16)Clarke, A. K.; Parkin, A.; Taylor, R. J. K.; Unsworth, W. P.; Rossi-Ashton, J. A. Photocatalytic Deoxygenation of Sulfoxides Using Visible Light: Mechanistic Investigations and Synthetic Applications. *ACS Catalysis* **2020**, *10* (10), 5814-5820. DOI: 10.1021/acscatal.0c00690.
(17)Zhu, X.; Shi, Y.; Mao, H.; Cheng, Y.; Zhu, C. Tetraethylammonium Bromide-Catalyzed Oxidative Thioesterification of Aldehydes and Alcohols. *Advanced Synthesis & Catalysis* **2013**, *355* (18), 3558-3562. DOI: https://doi.org/10.1002/adsc.201300584.
(18)Xie, S.; Su, L.; Mo, M.; Zhou, W.; Zhou, Y.; Dong, J. Cu-Catalyzed Oxidative Thioesterification of Aroylhydrazides with Disulfides. *The Journal of Organic Chemistry* **2021**, *86* (1), 739-749. DOI: 10.1021/acs.joc.0c02328.
(19)Man, Y.; Zeng, X.; Xu, B. Synthesis of Thioesters from Aldehydes via N-Heterocyclic Carbene (NHC) Catalyzed Radical Relay. *Chemistry – A European Journal* **2023**, *29* (17), e202203716. DOI: https://doi.org/10.1002/chem.202203716.
(20)Takayama, R.; Yamada, A.; Fuchibe, K.; Ichikawa, J. Synthesis of Sulfanylated Difluoroalkenes: Electrophilic Difluoromethylidenation of Dithioesters with Difluorocarbene. *Organic Letters* **2017**, *19* (19), 5050-5053. DOI: 10.1021/acs.orglett.7b02222.
(21)Lim, Y. W.; Hewitt, R. J.; Burkett, B. A. The Dual Reactivity of 5-S/5-O-Phenyl-1,4,2-oxathiazoles: A Fragmentation Pathway That Affords Nitriles in the Presence of Water. *European Journal of Organic Chemistry* **2015**, *2015* (22), 4840-4842. DOI: https://doi.org/10.1002/ejoc.201500637.
(22)Nakayama, J.; Ozasa, H.; Hoshino, M. Benzyne-induced fragmentation reactions of 1,3-dithiolanes. *HETEROCYCLES* **1984**, *22* (5), 1053-1056. DOI: 10.3987/R-1984-05-1053.
(23)Yao, C.; Yang, J.; Lu, X.; Zhang, S.; Zhao, J. Ynamide-Mediated Thionoester and Dithioester Syntheses. *Organic Letters* **2020**, *22* (16), 6628-6631. DOI: 10.1021/acs.orglett.0c02402.
(24)Jefford, C. W.; Barchietto, G., Reaction of Phosphorus Ylids with Singlet Oxygen. T*etrahedron Lett*. **1977**, *18*, 4531-4534. DOI: https://doi.org/10.1016/S0040-4039(01)83557-7
(25)Kentaro, O.; Yuji, T.; Jun-ichi, S.; Takashi, K.; Isao, K.; Yasuo, K.; Yusuke, Y.; Shin ichi, Y.; Hiroshi, O. Reactions of Wittig Reagents with Episulfides or Elemental Sulfur. *Bulletin of the Chemical Society of Japan* **1988**, *61* (12), 4323-4327. DOI: 10.1246/bcsj.61.4323.

NMR Spectral Graphics

^{1}H NMR (400 MHz, $CDCl_3$) of **3a**

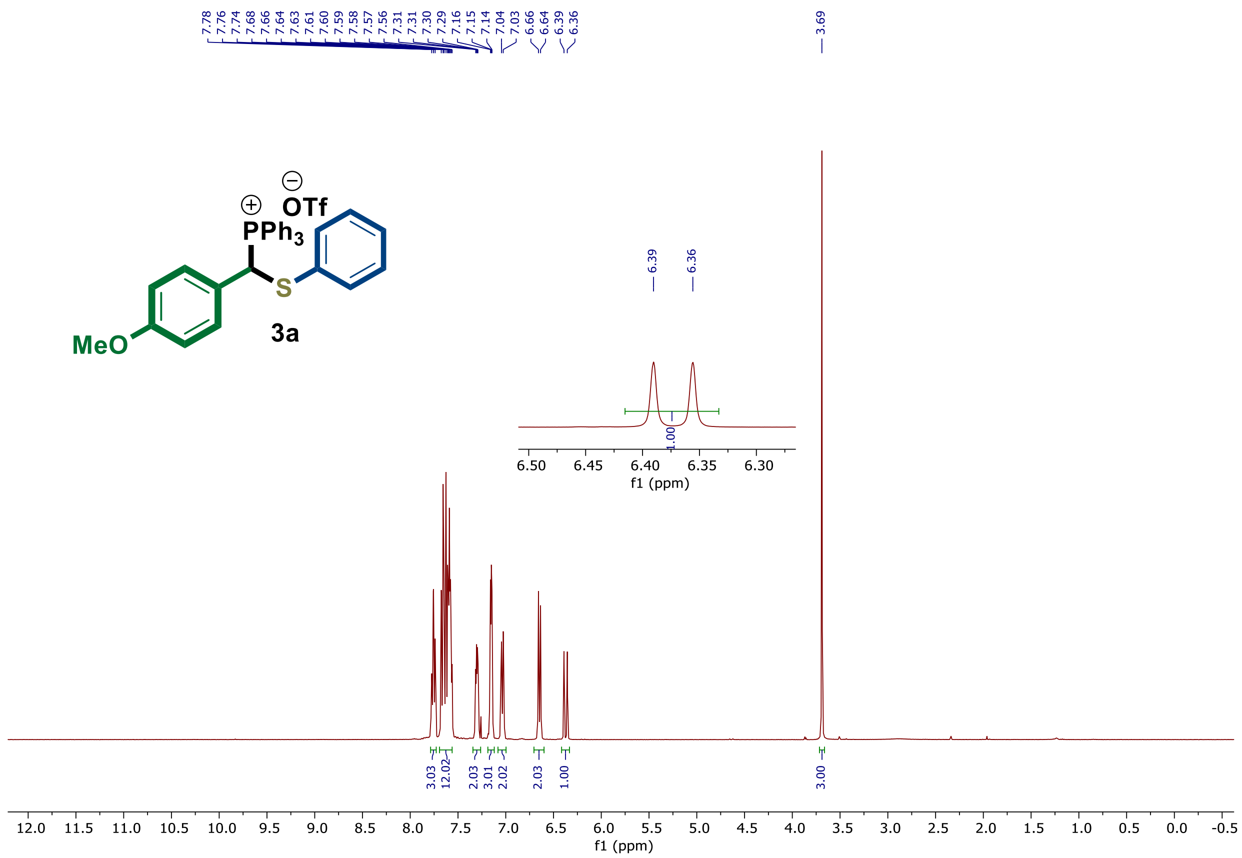

^{13}C NMR (100 MHz, $CDCl_3$) of **3a**

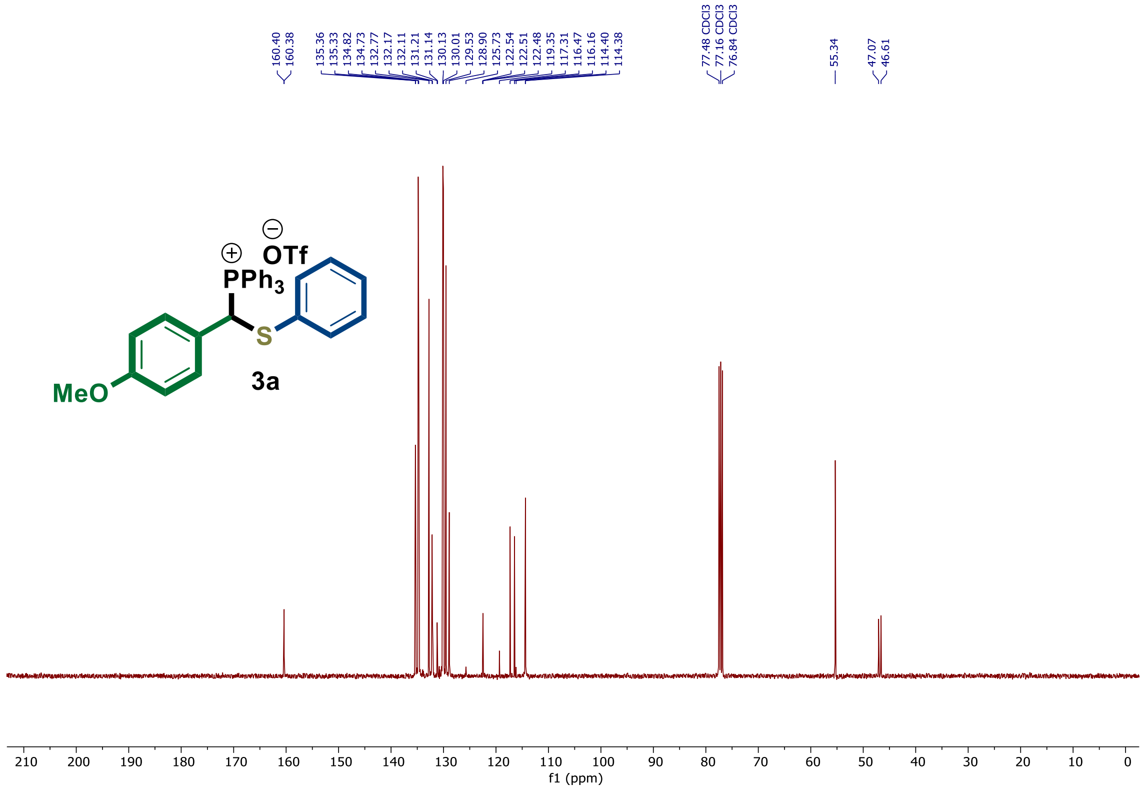

^{31}P NMR (203 MHz, $CDCl_3$) of **3a**

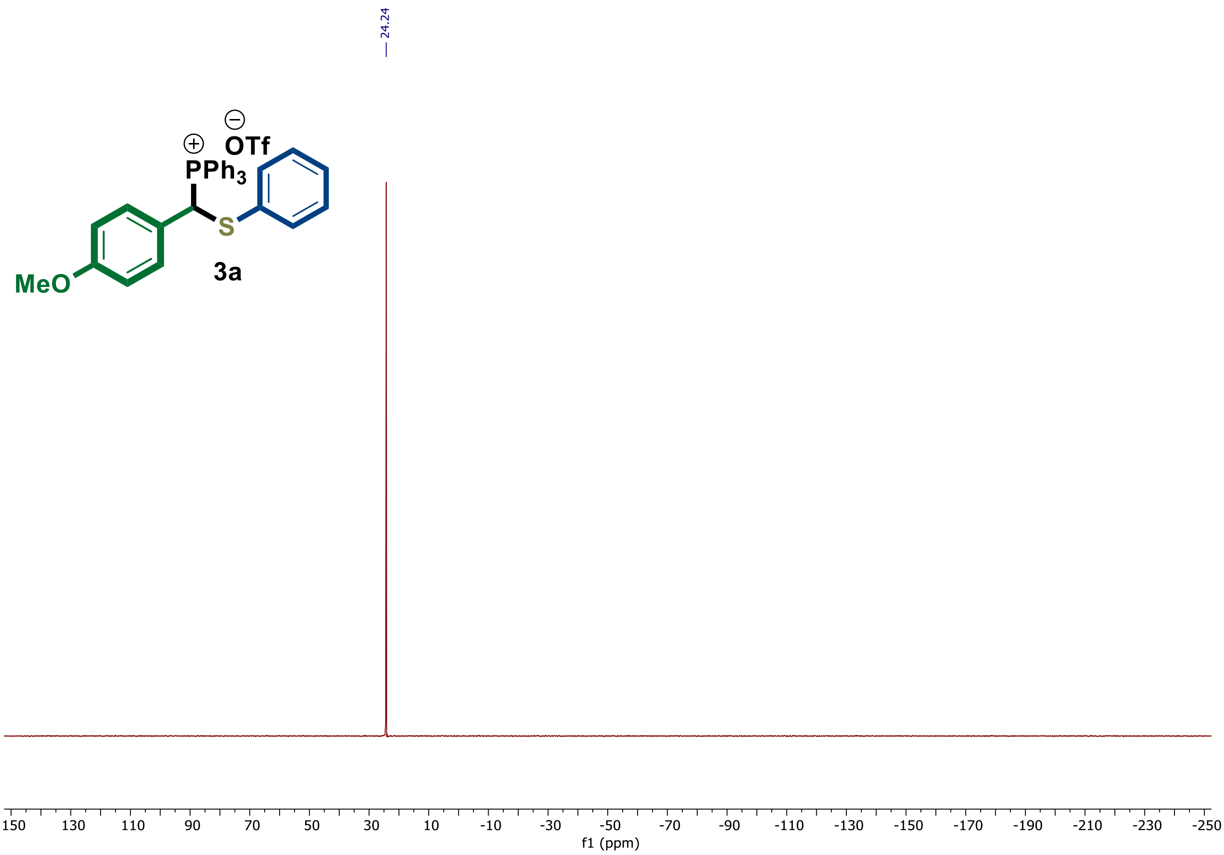

^{19}F NMR (376 MHz, $CDCl_3$) of **3a**

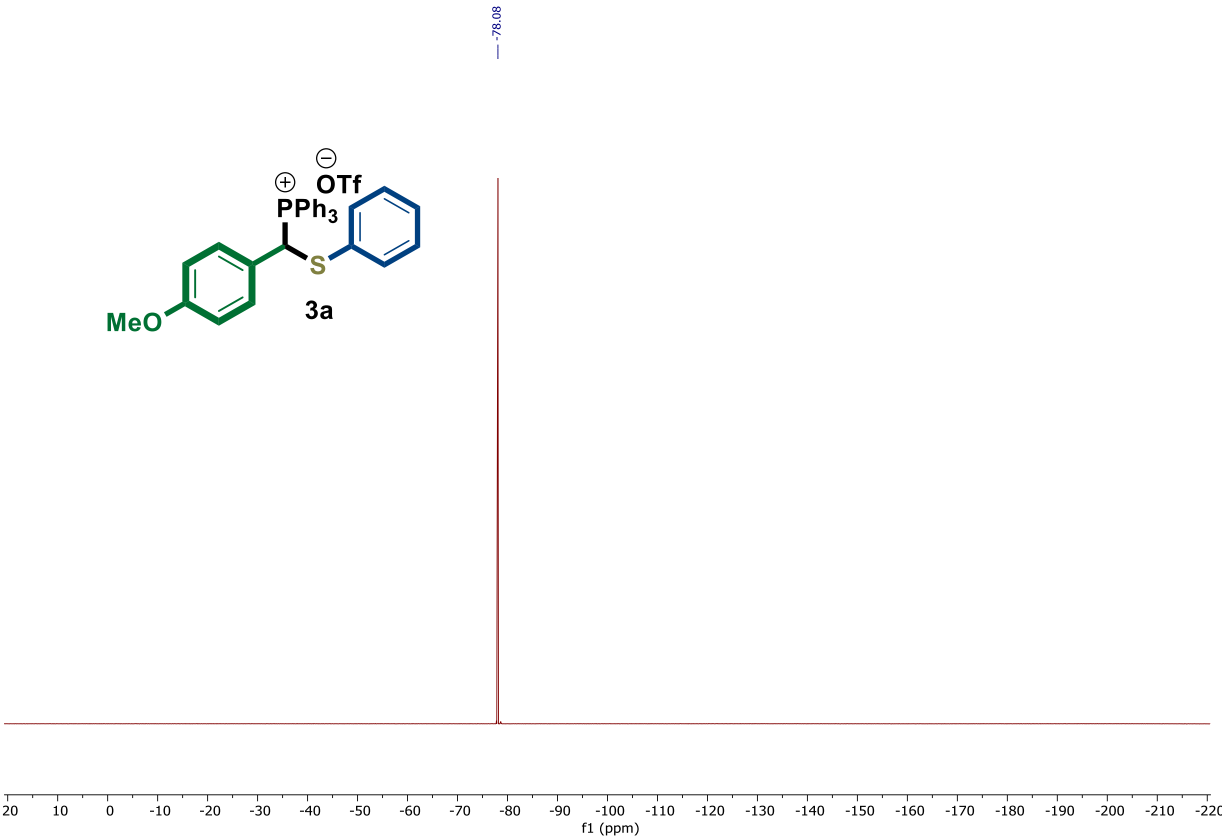

^{1}H NMR (400 MHz, $CDCl_3$) of **3b**

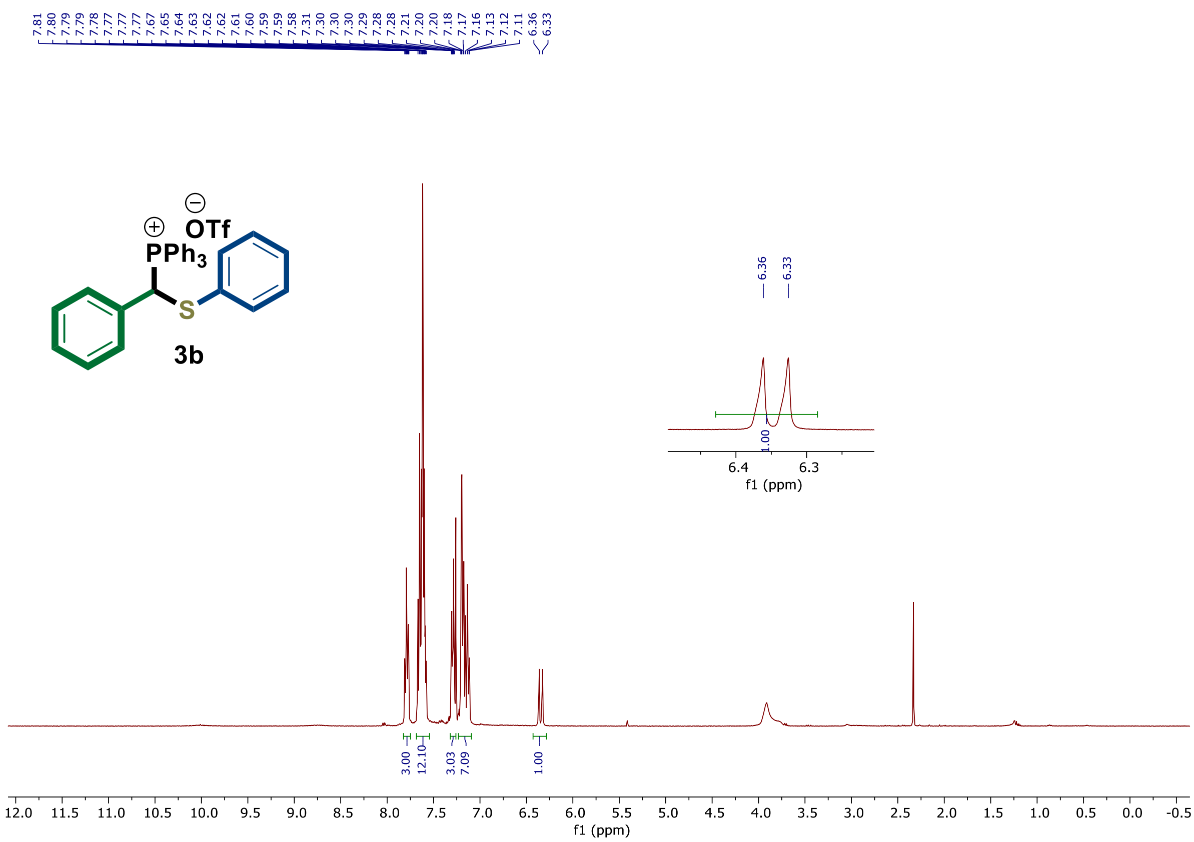

^{13}C NMR (100 MHz, $CDCl_3$) of **3b**

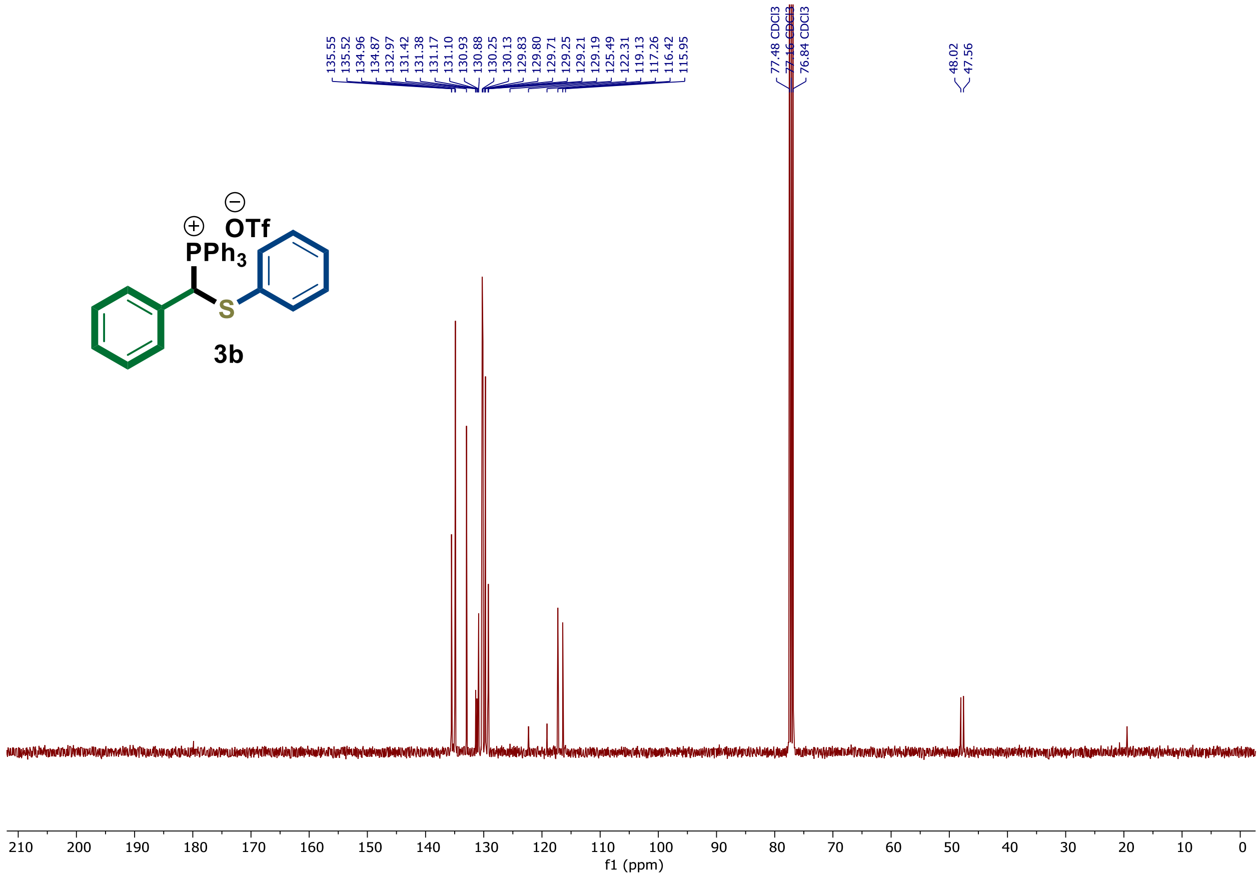

^{31}P NMR (162 MHz, $CDCl_3$) of **3b**

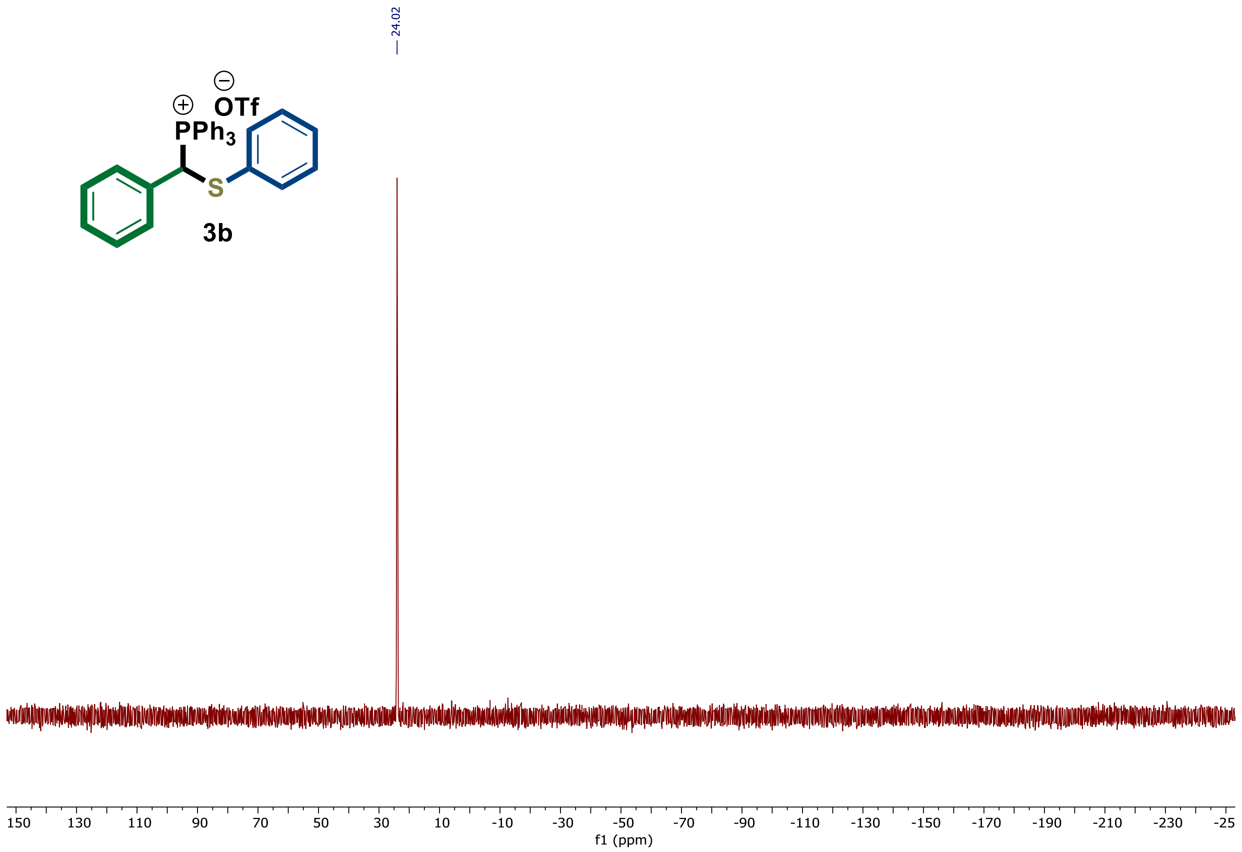

^{19}F NMR (376 MHz, $CDCl_3$) of **3b**

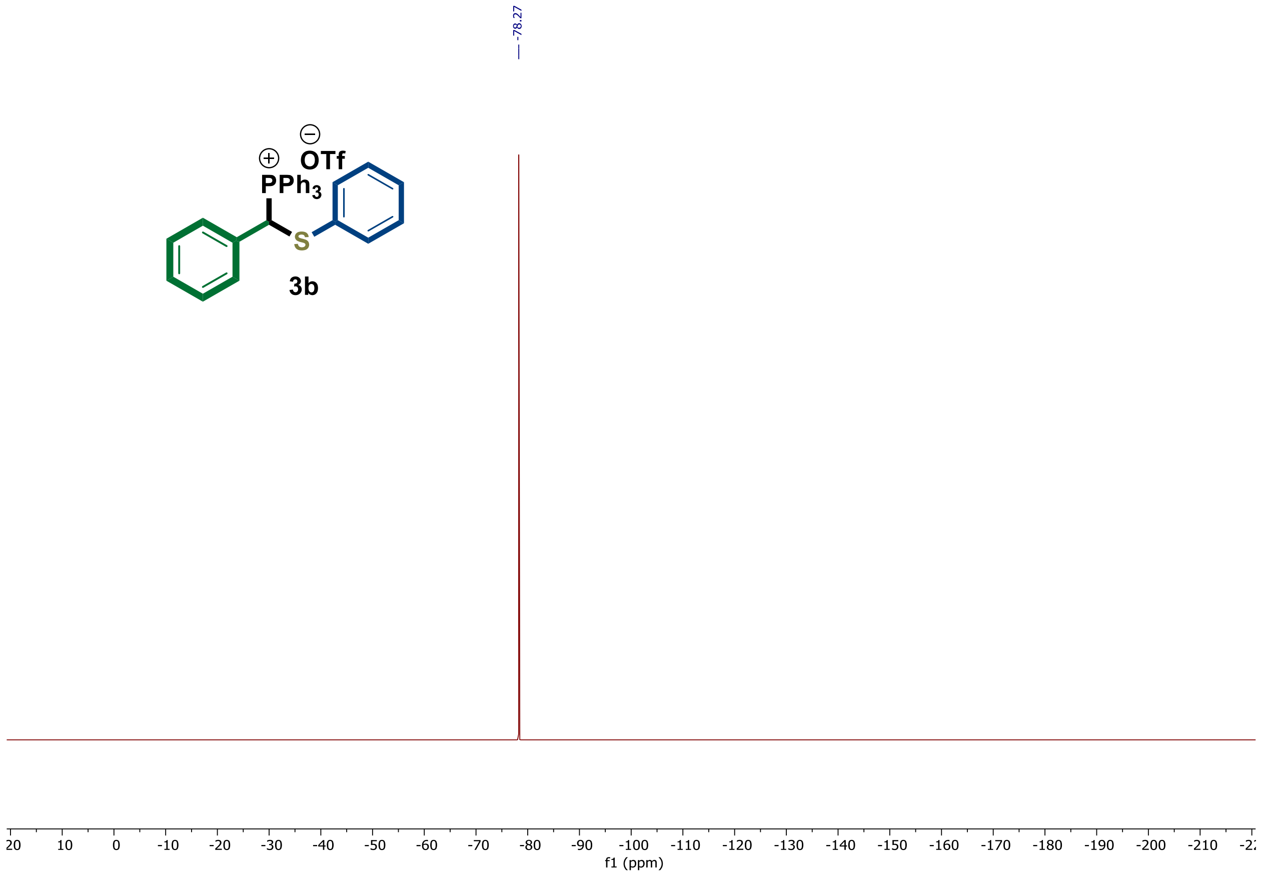

^{1}H NMR (400 MHz, $CDCl_3$) of **3c**

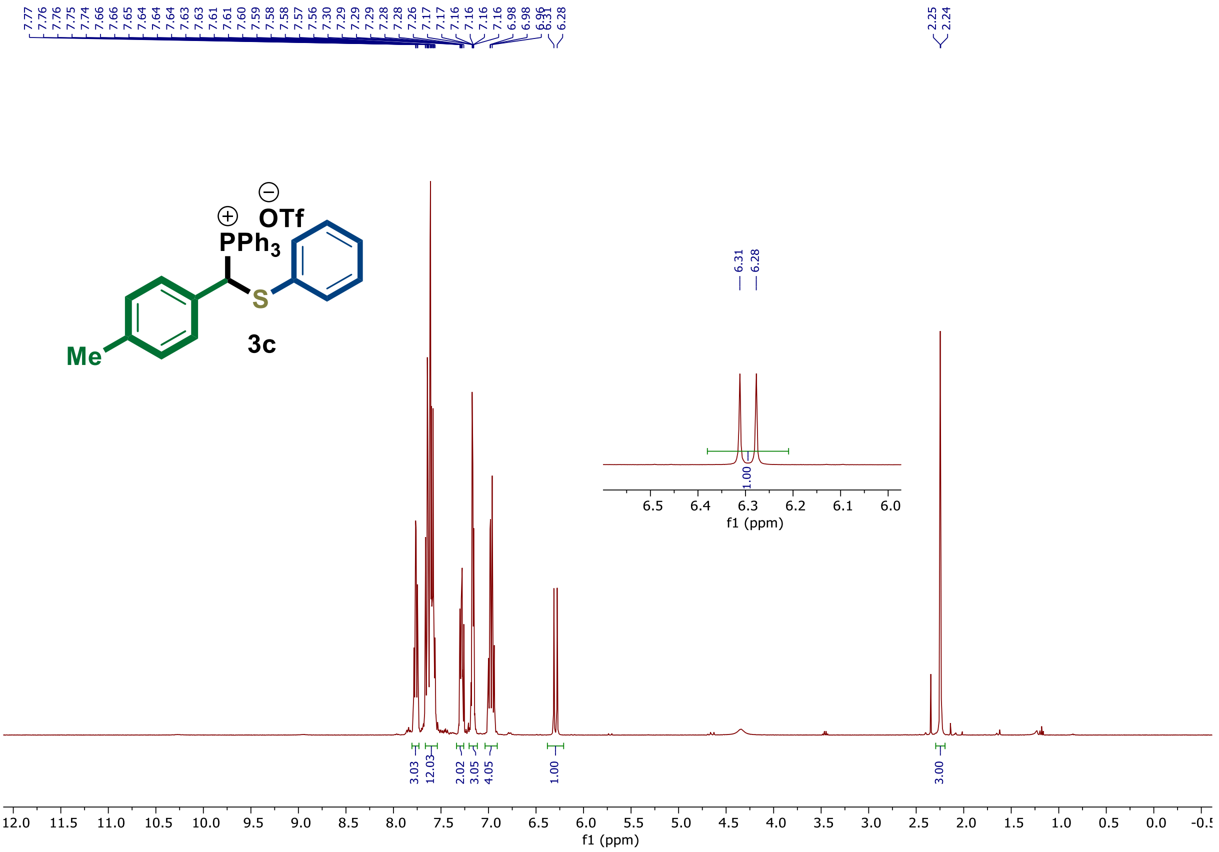

^{13}C NMR (100 MHz, $CDCl_3$) of **3c**

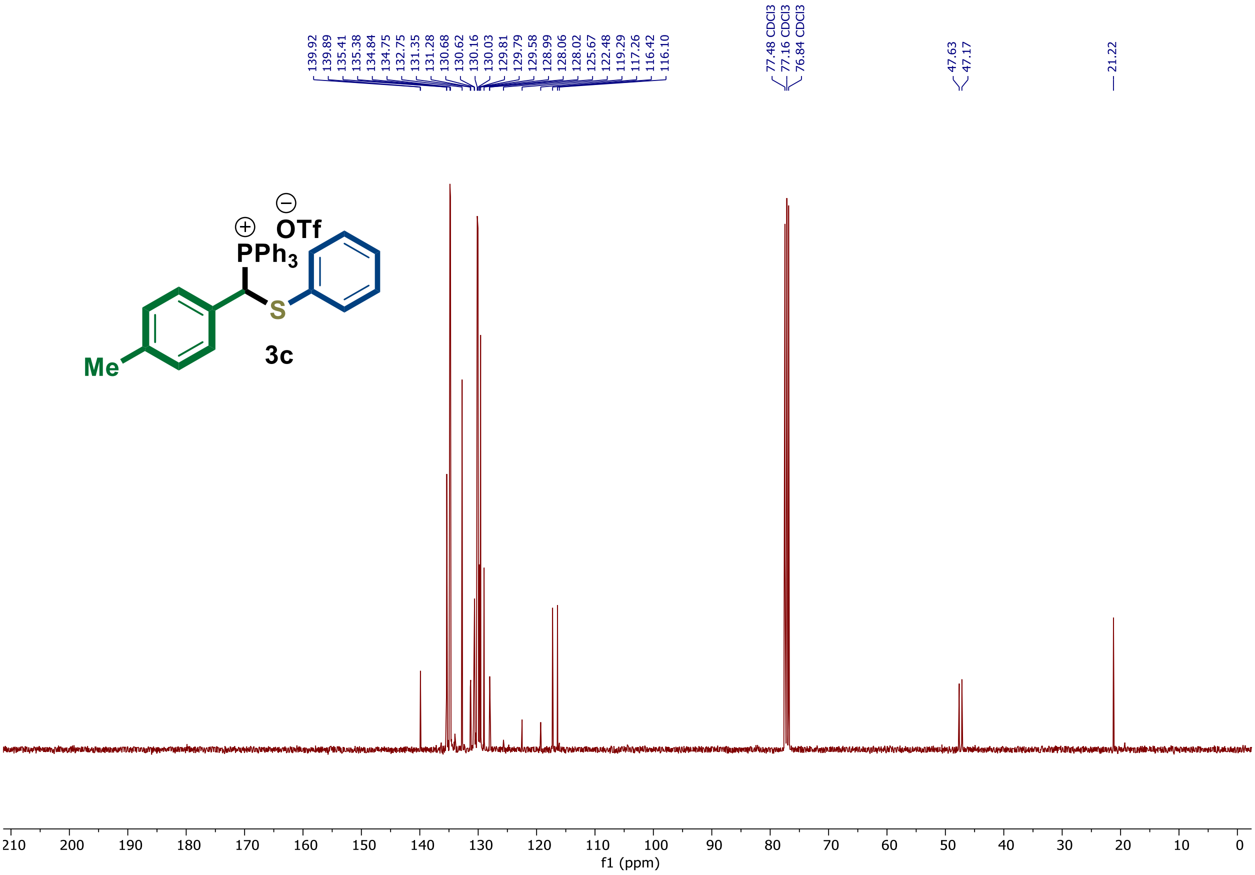

^{31}P NMR (162 MHz, $CDCl_3$) of **3c**

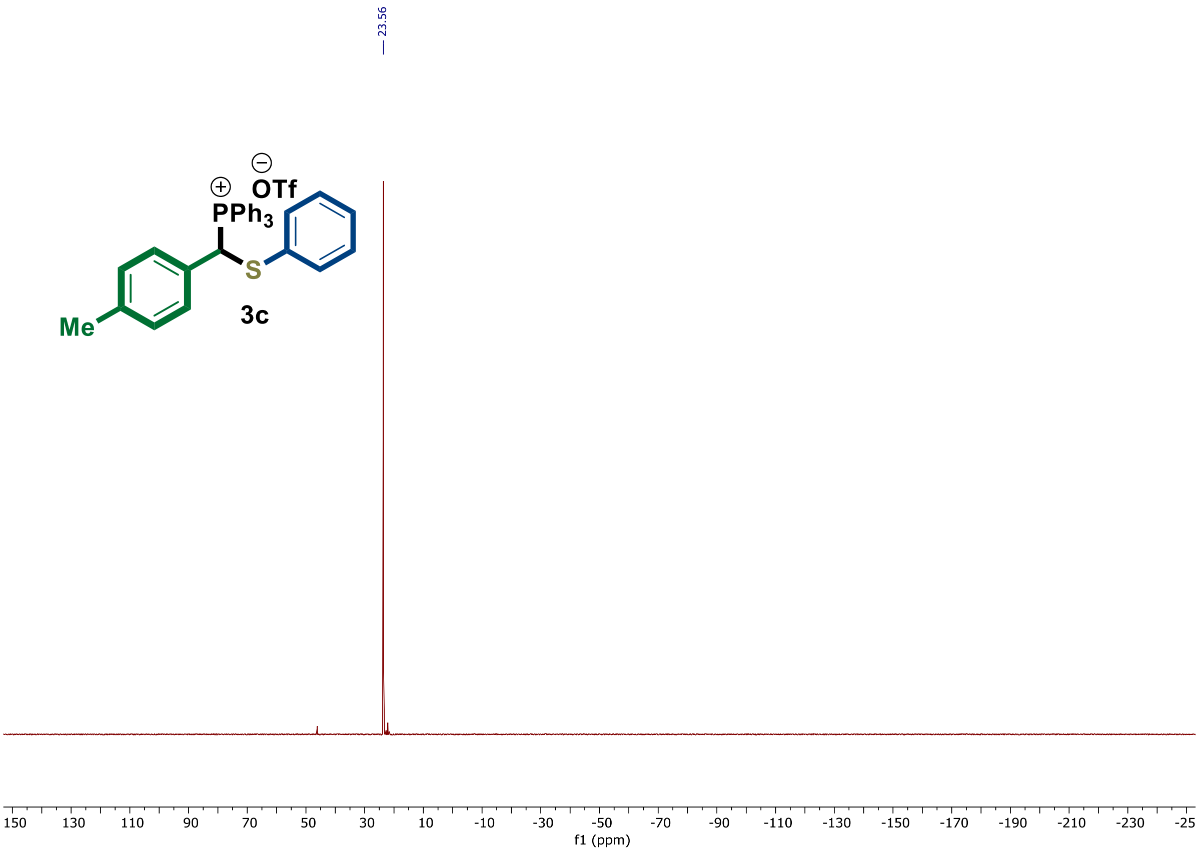

^{19}F NMR (376 MHz, $CDCl_3$) of **3c**

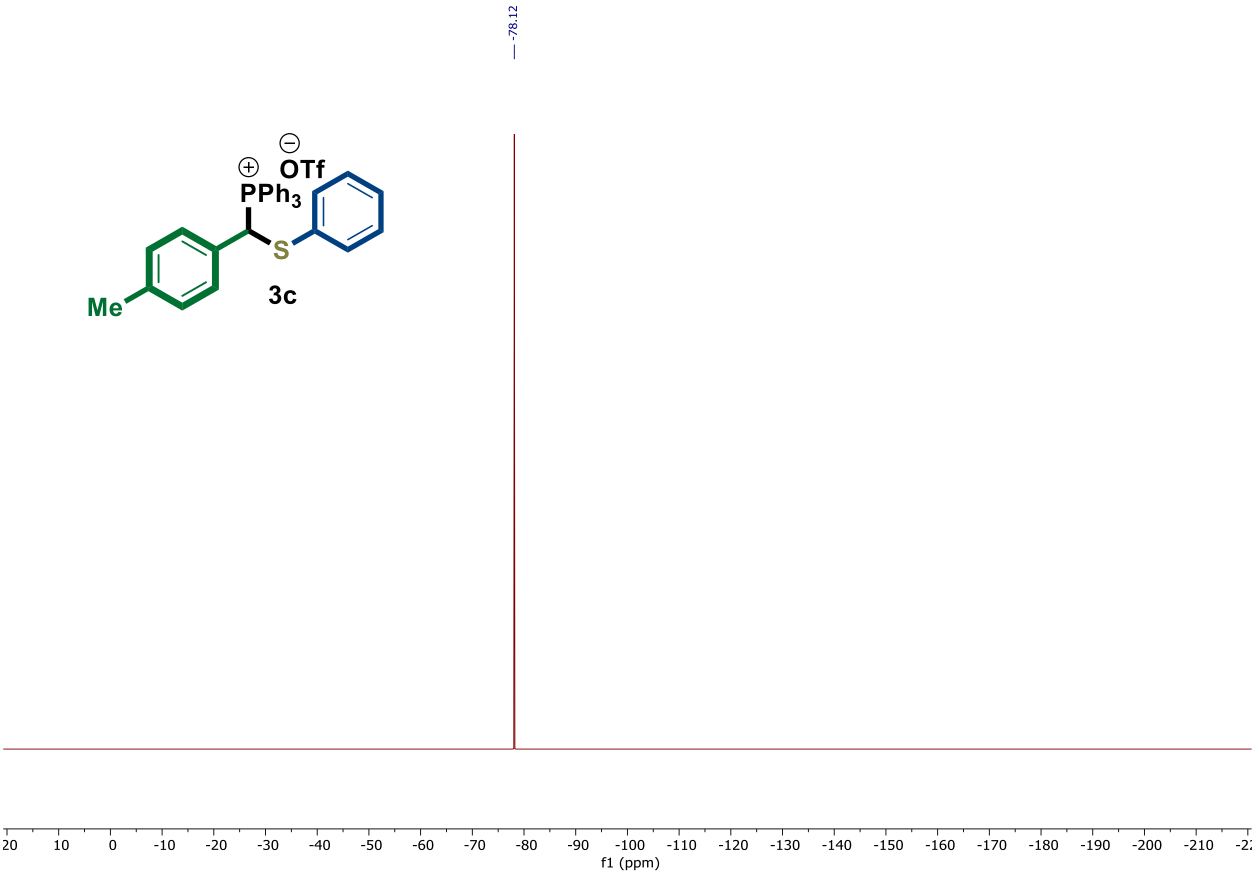

^{1}H NMR (400 MHz, CDCl$_3$) of **3d**

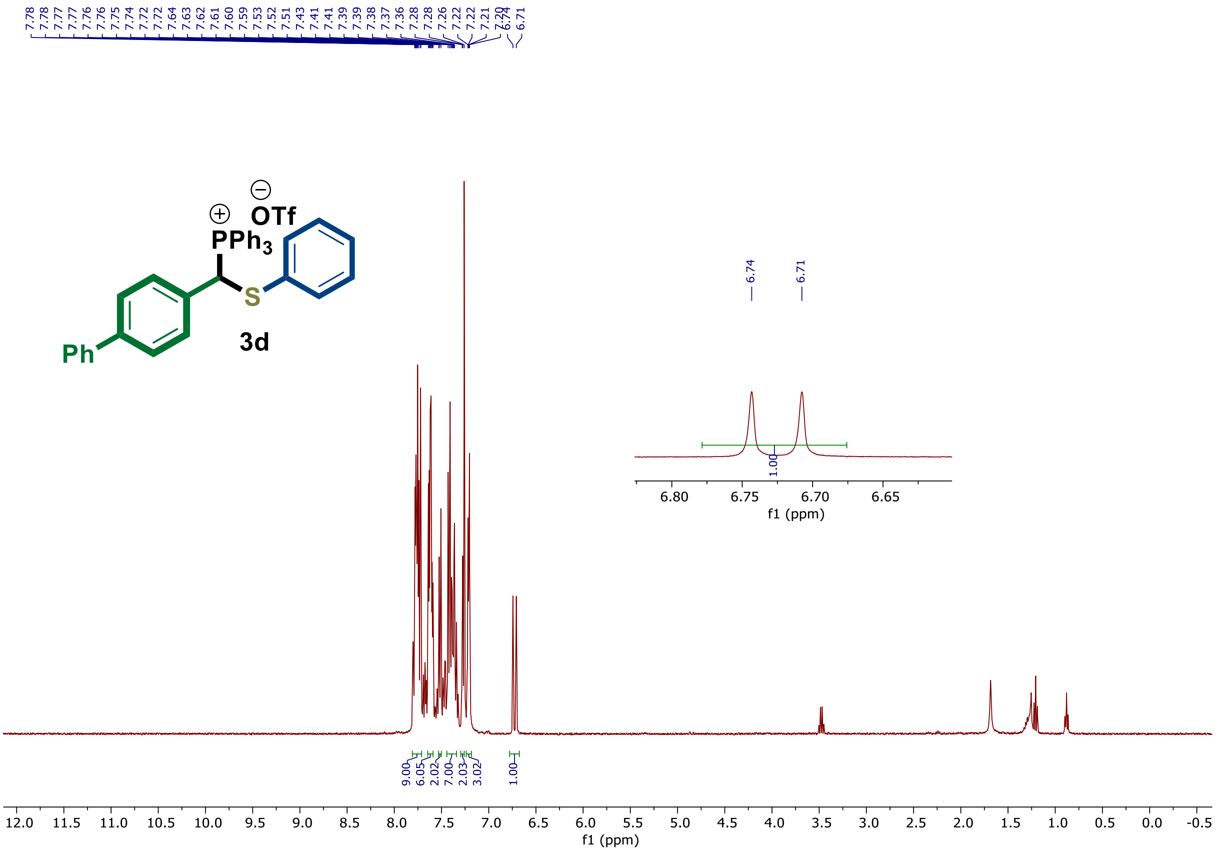

^{13}C NMR (100 MHz, CDCl$_3$) of **3d**

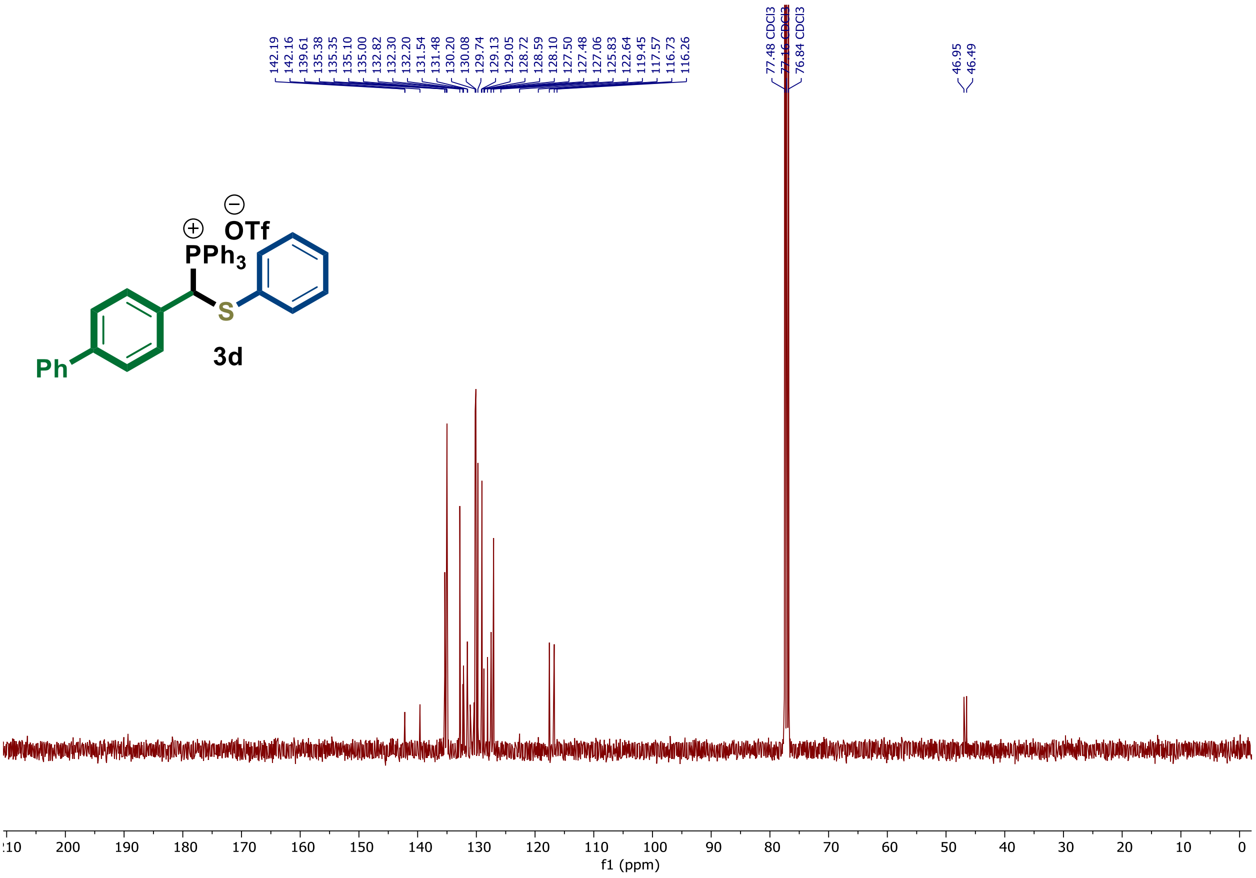

^{31}P NMR (162 MHz, $CDCl_3$) of **3d**

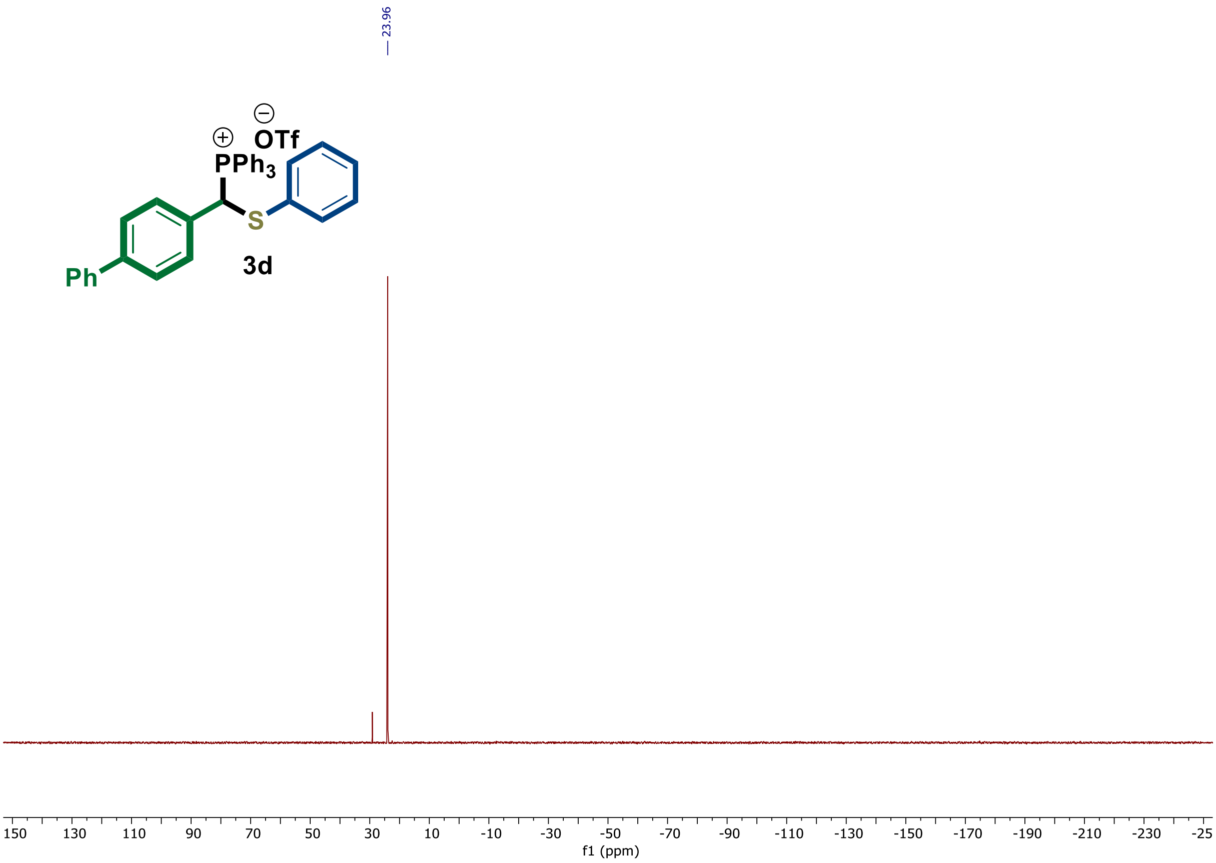

^{19}F NMR (376 MHz, $CDCl_3$) of **3d**

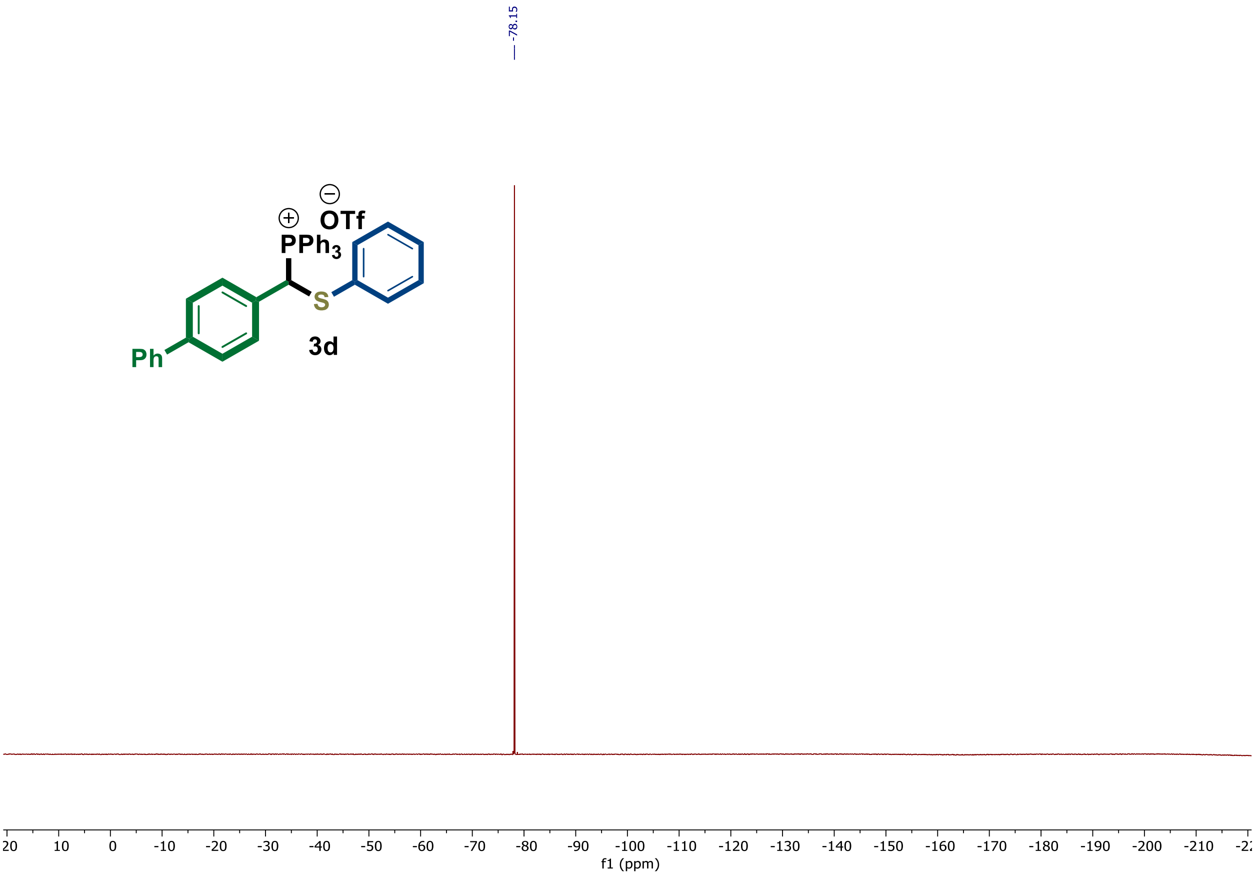

^{1}H NMR (400 MHz, $CDCl_3$) of **3e**

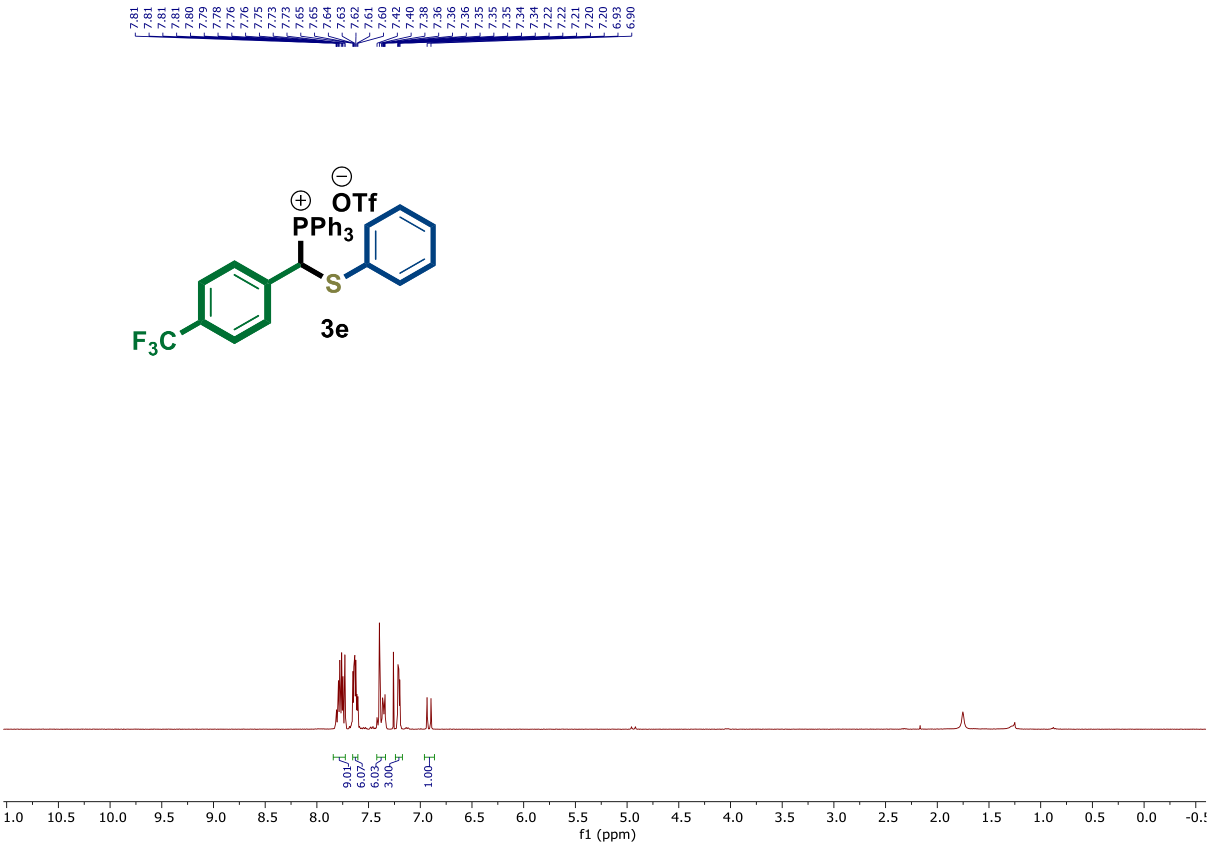

^{13}C NMR (100 MHz, $CDCl_3$) of **3e**

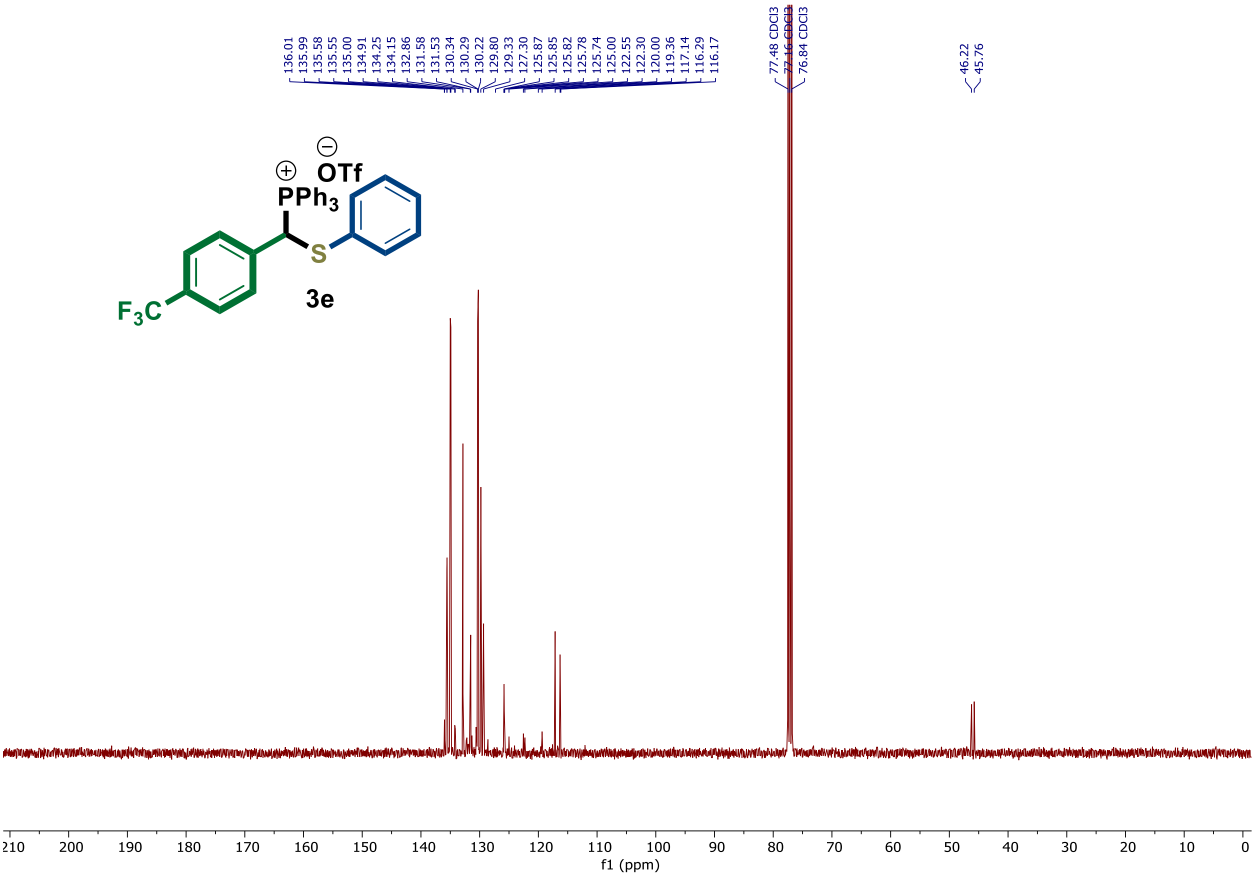

^{31}P NMR (162 MHz, $CDCl_3$) of **3e**

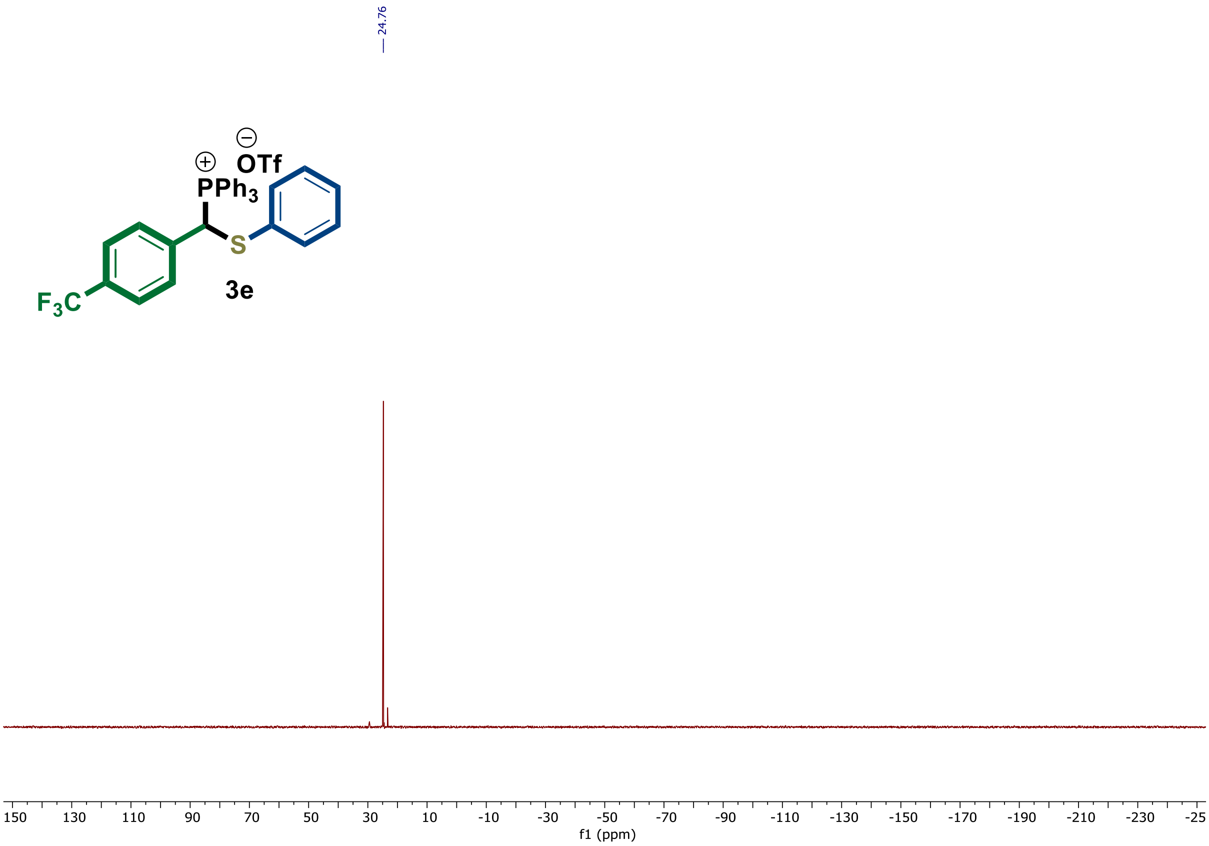

^{19}F NMR (376 MHz, $CDCl_3$) of **3e**

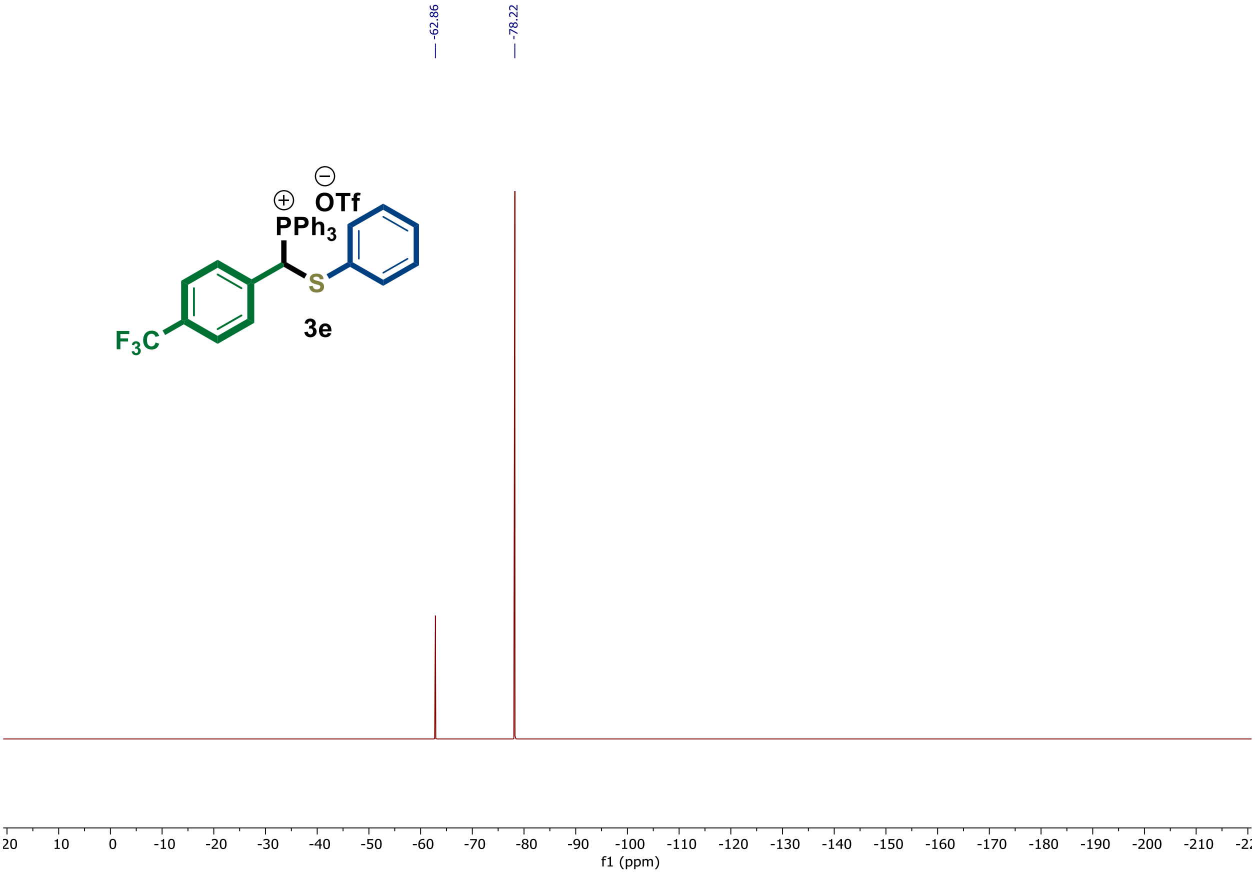

^{1}H NMR (400 MHz, $CDCl_3$) of **3f**

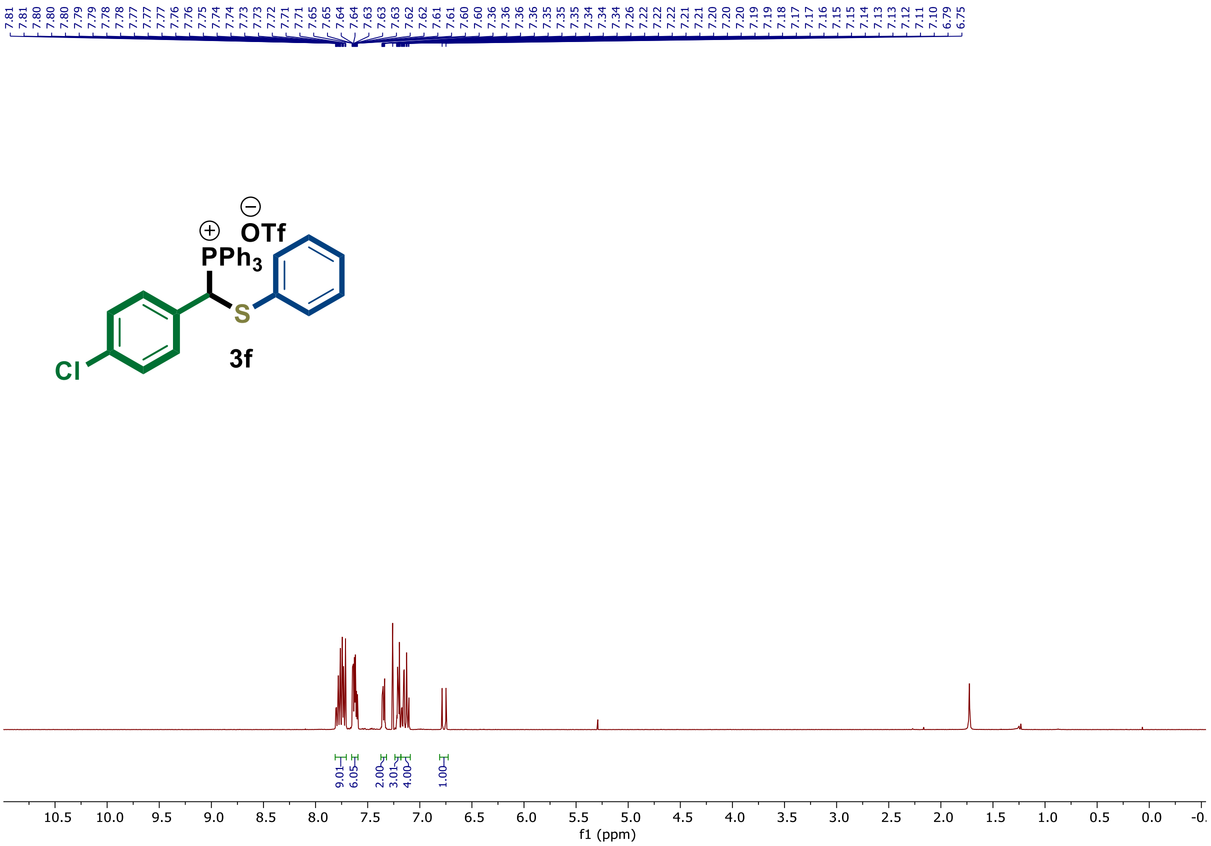

^{13}C NMR (100 MHz, $CDCl_3$) of **3f**

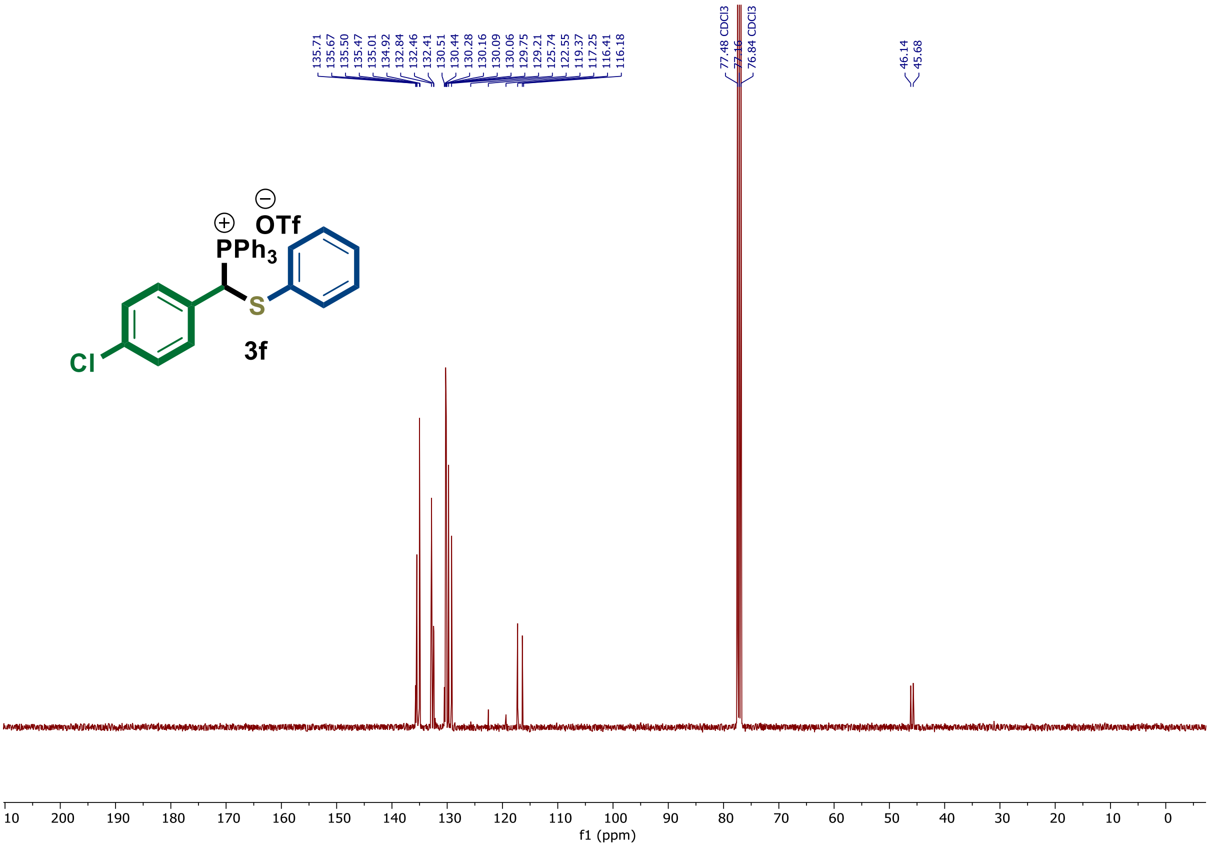

^{31}P NMR (162 MHz, $CDCl_3$) of **3f**

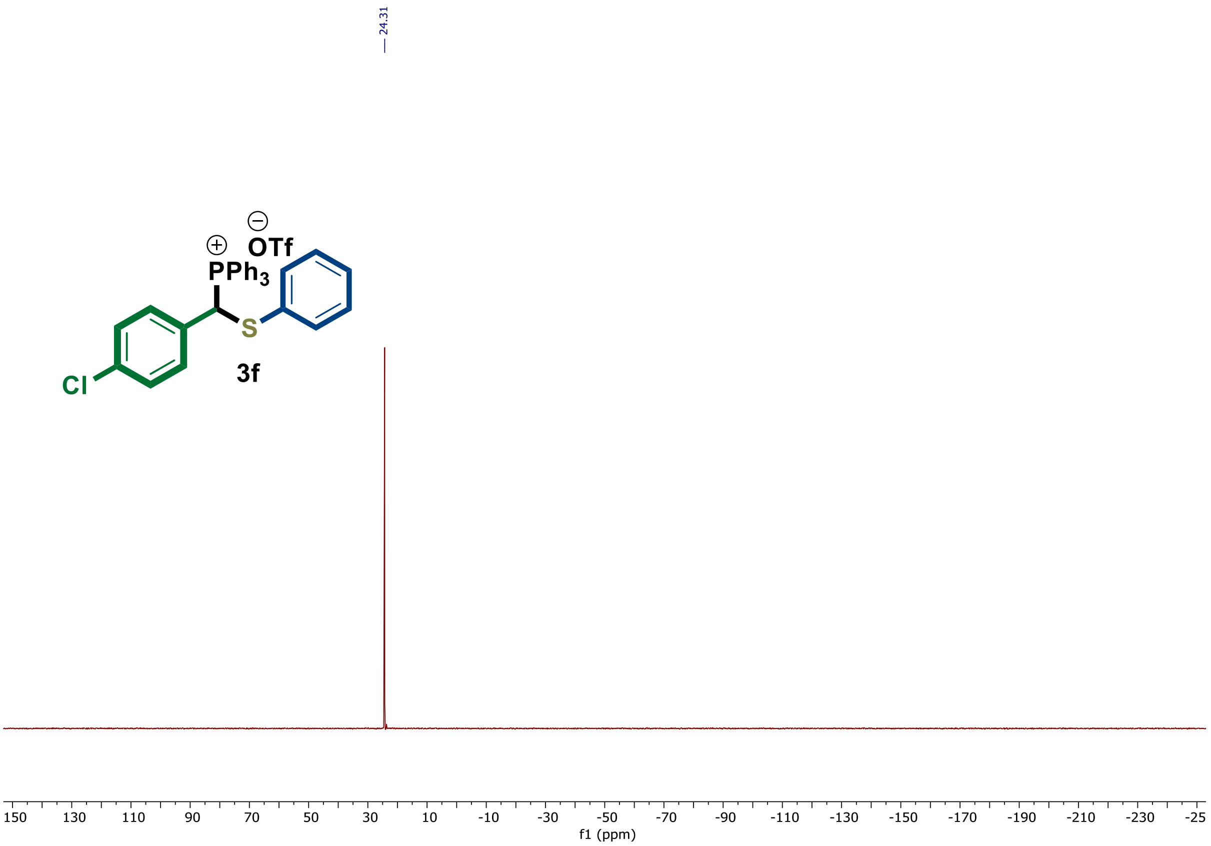

^{19}F NMR (376 MHz, $CDCl_3$) of **3f**

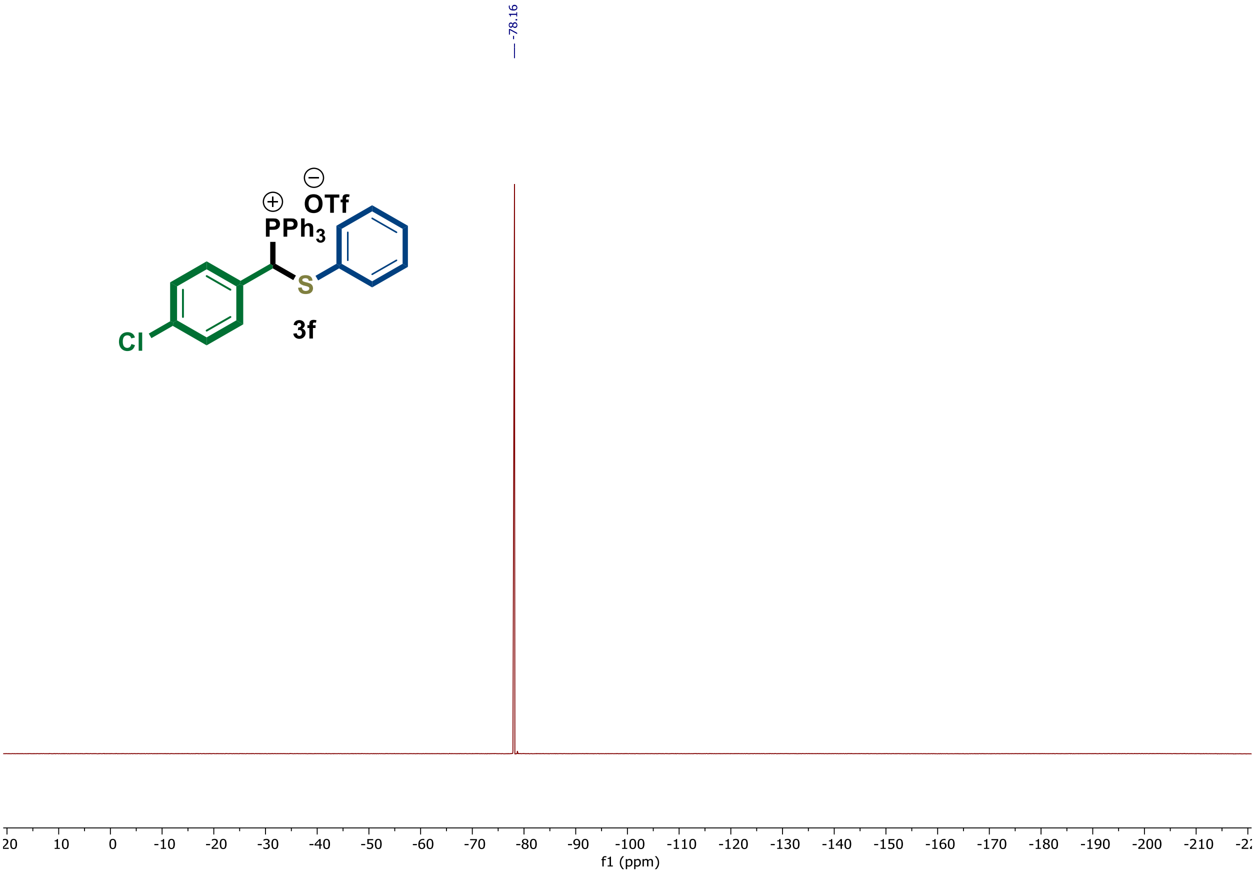

^{1}H NMR (400 MHz, $CDCl_3$) of **3g**

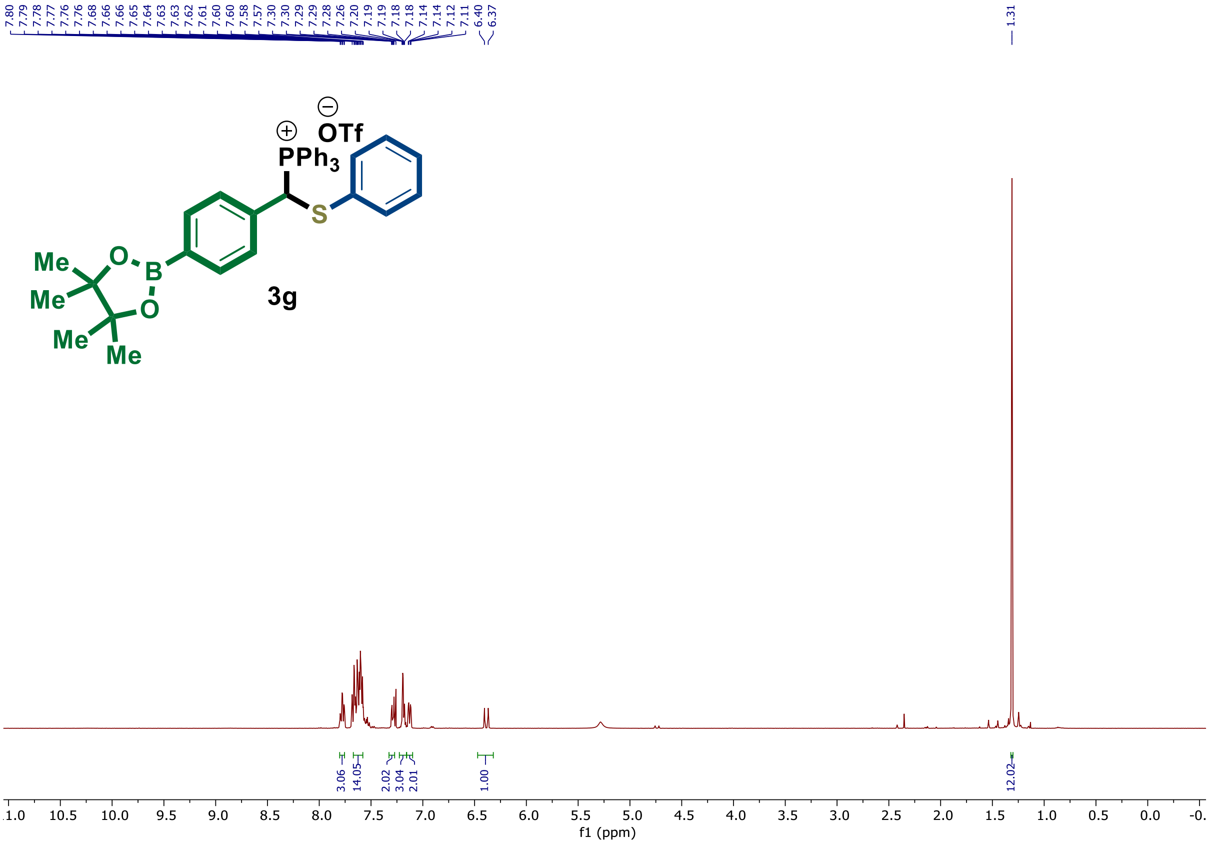

^{13}C NMR (100 MHz, $CDCl_3$) of **3g**

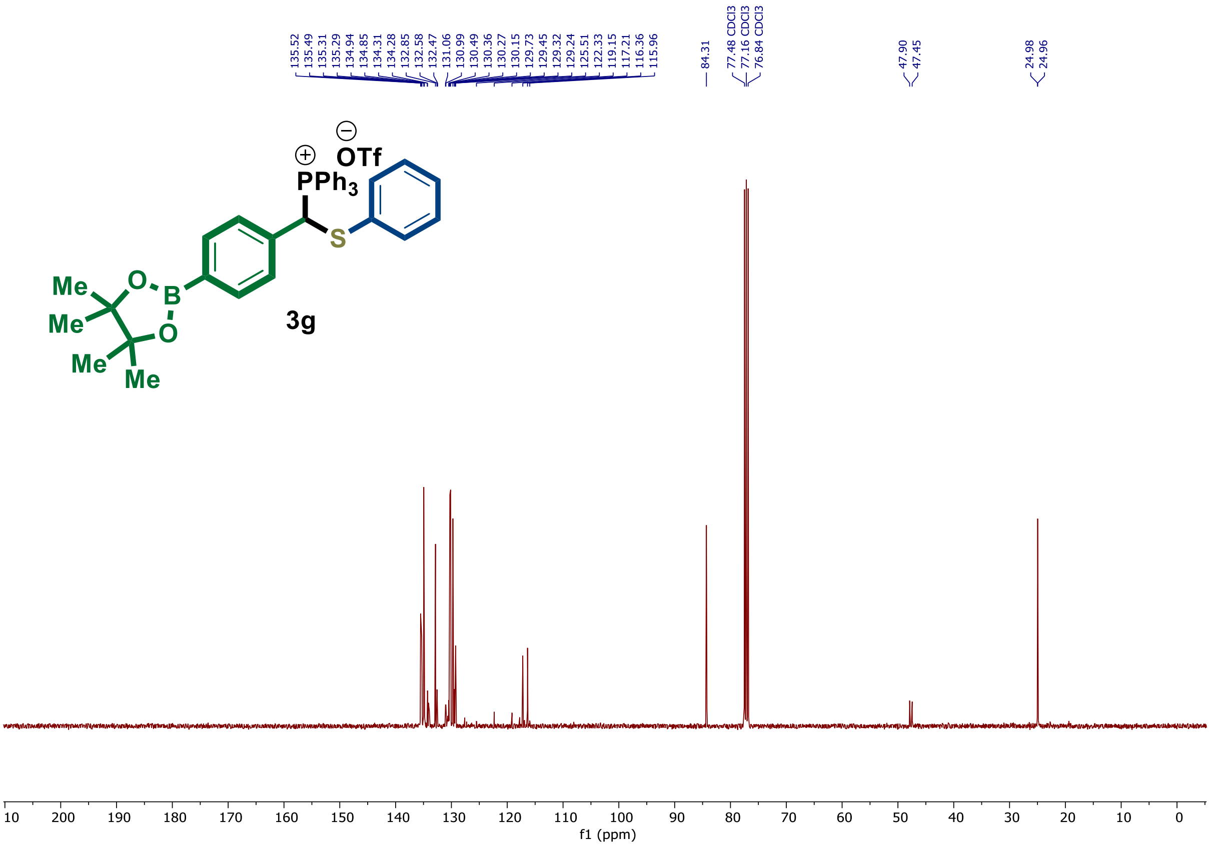

^{31}P NMR (162 MHz, $CDCl_3$) of **3g**

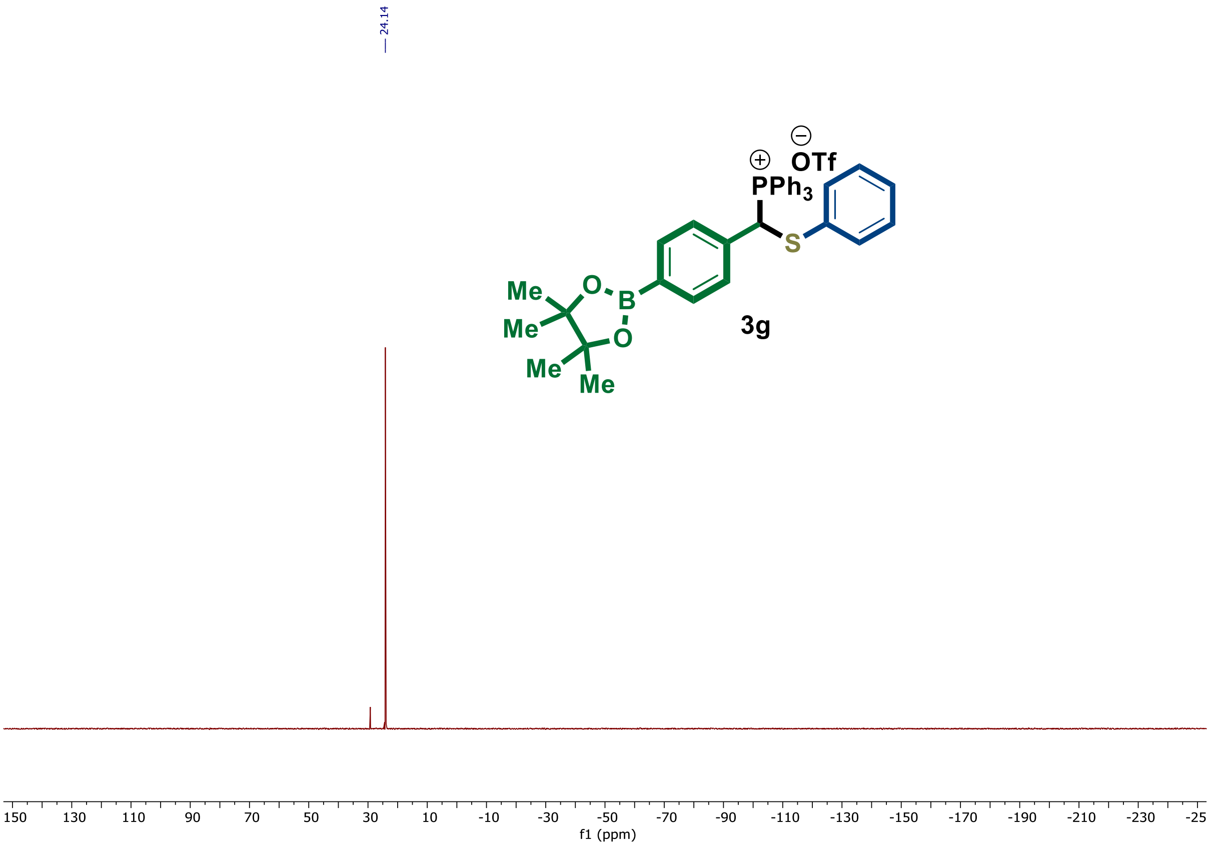

^{19}F NMR (376 MHz, $CDCl_3$) of **3g**

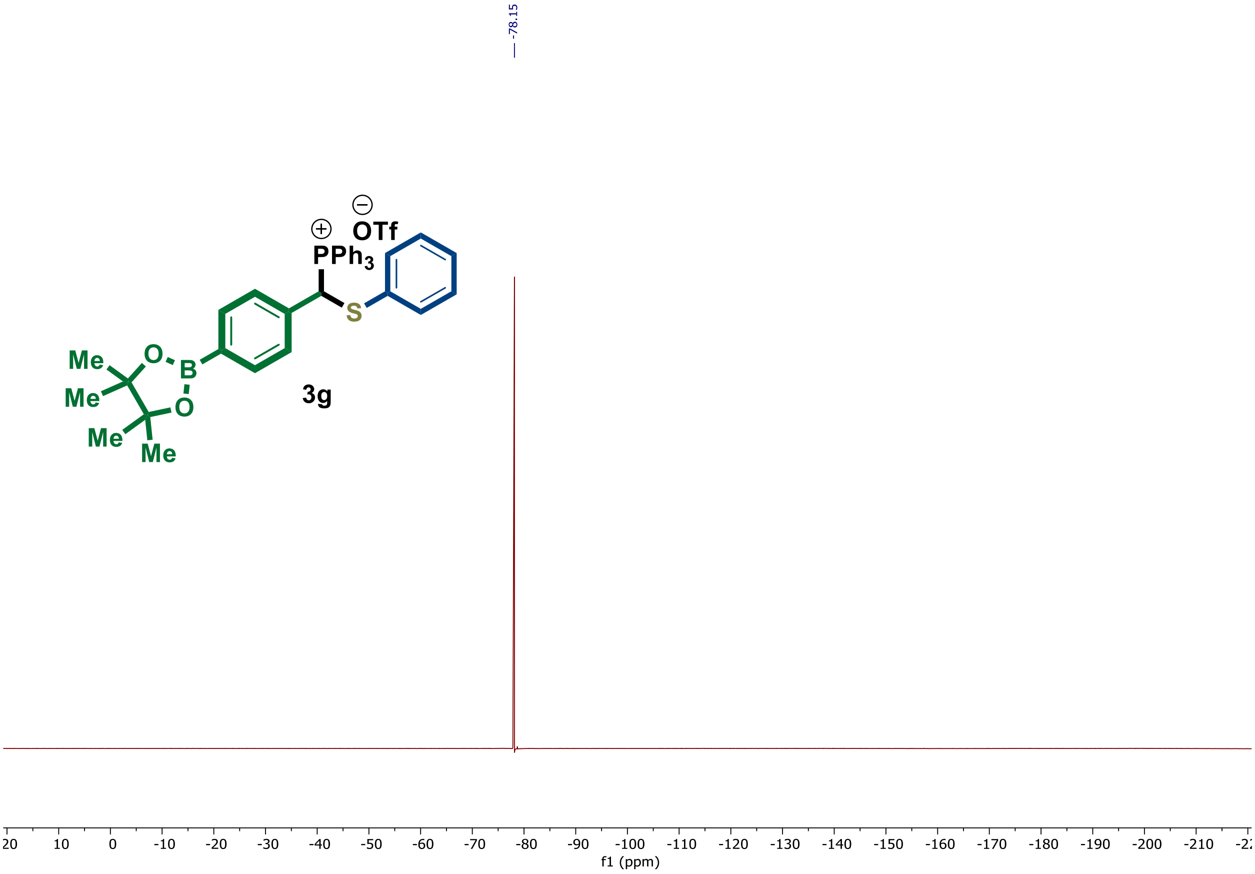

^{11}B NMR (128 MHz, CDCl$_3$) of **3g**

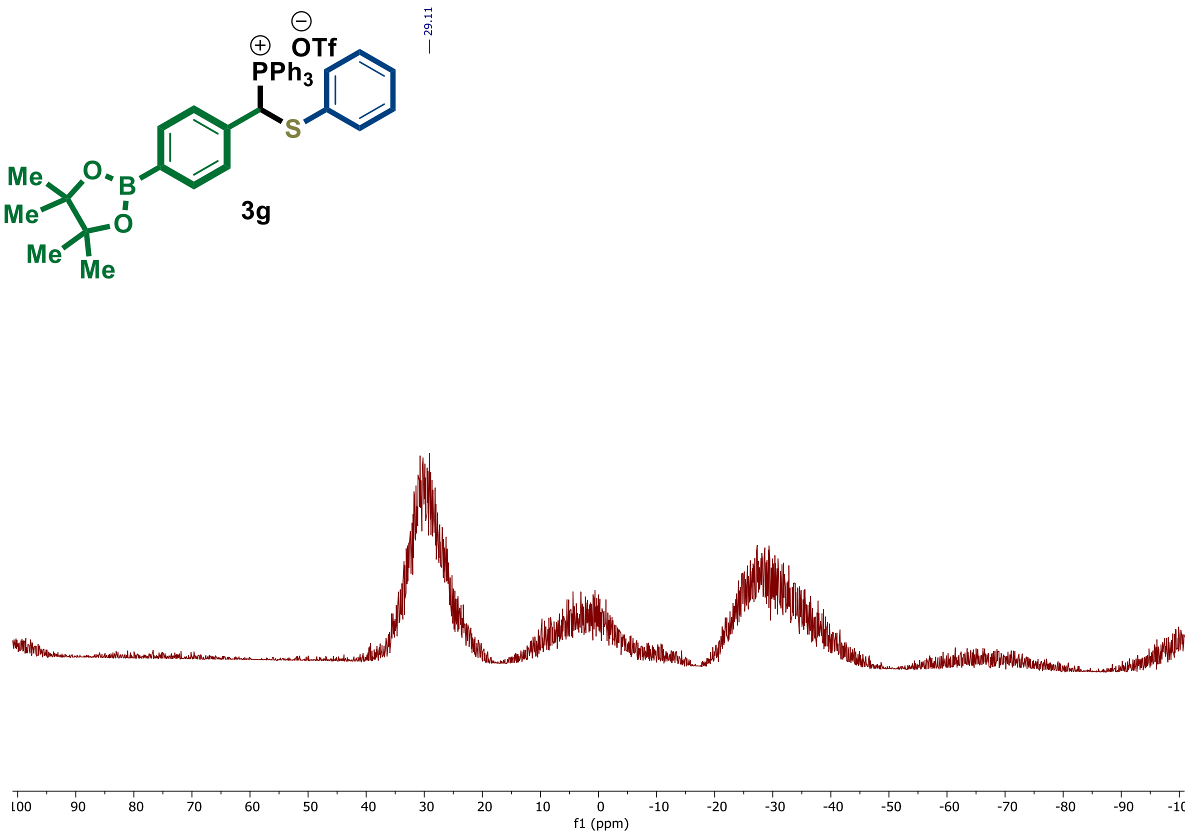

^{1}H NMR (400 MHz, DMSO-d6) of **3h**

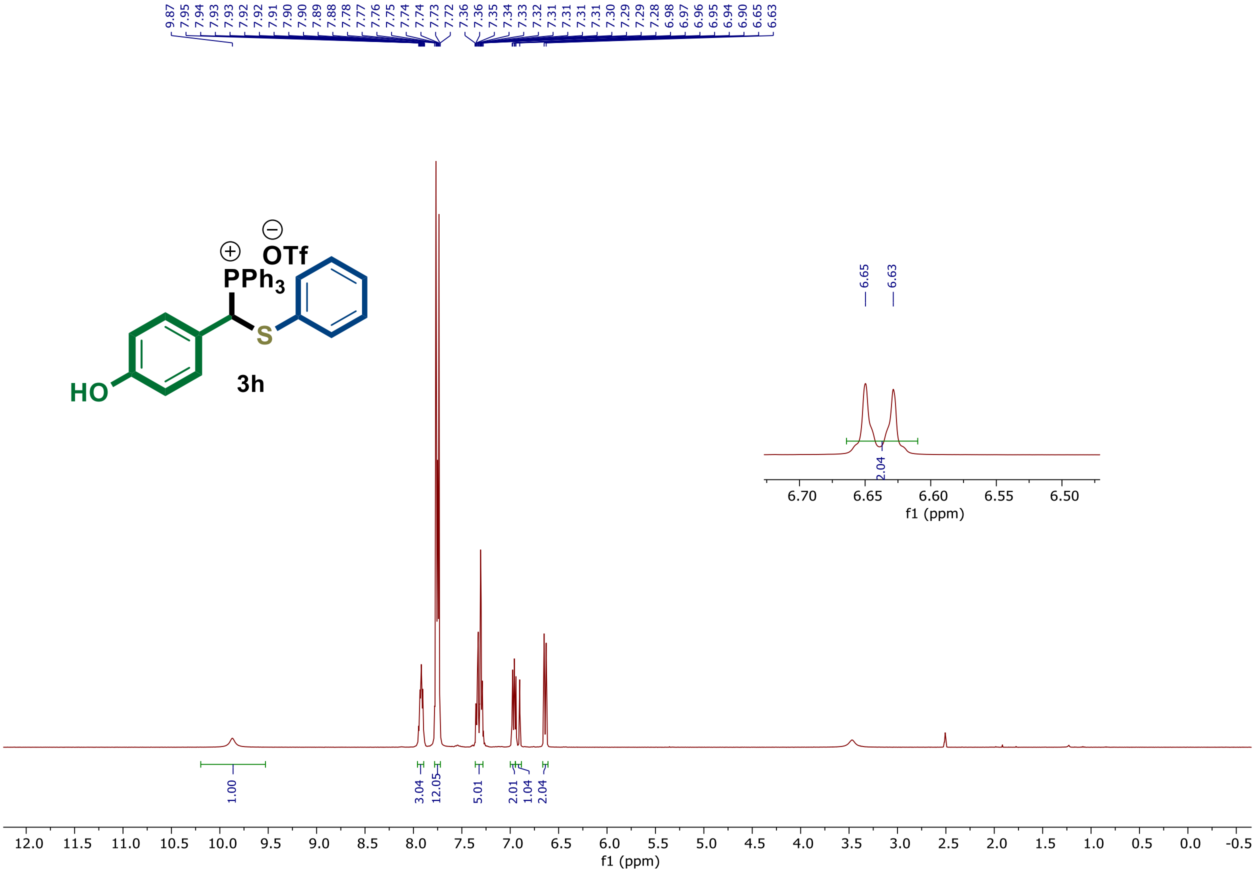

^{13}C NMR (100 MHz, DMSO-d6) of **3h**

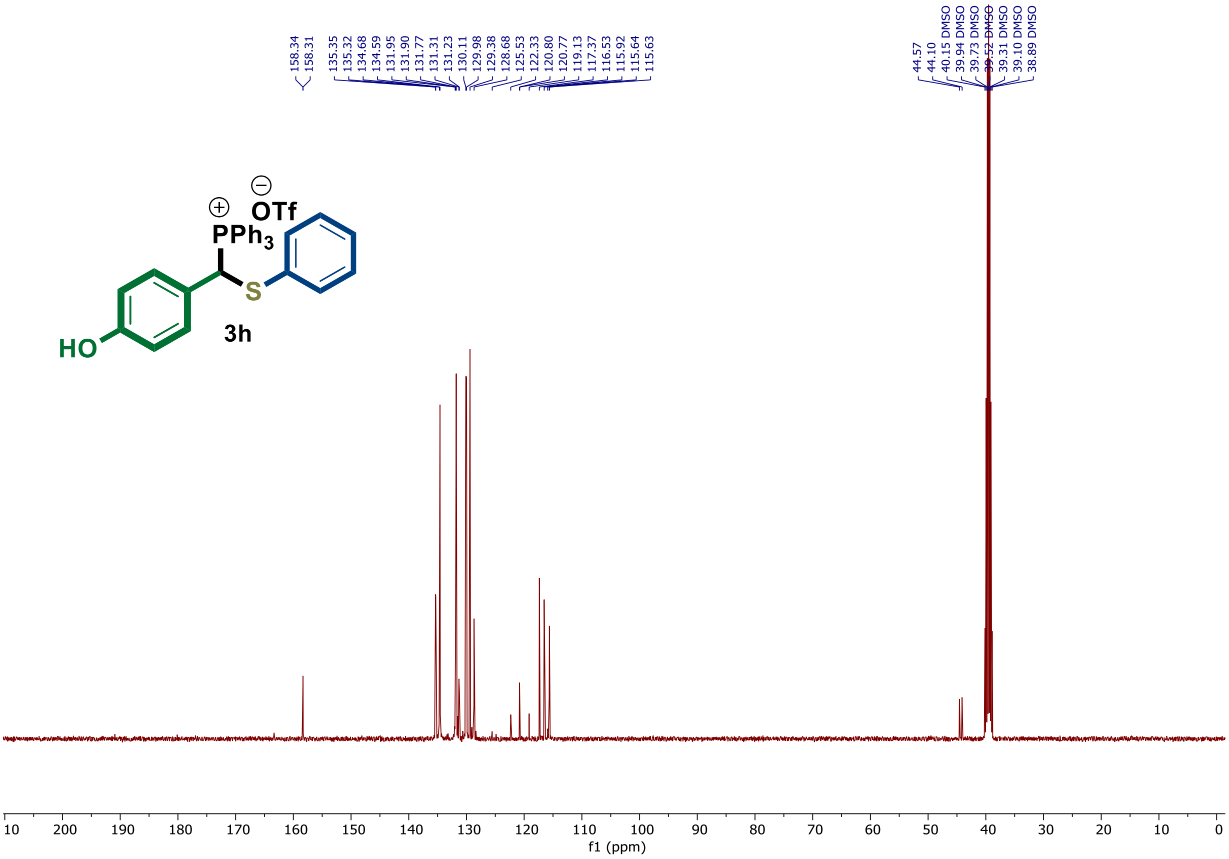

^{31}P NMR (162 MHz, DMSO-d6) of **3h**

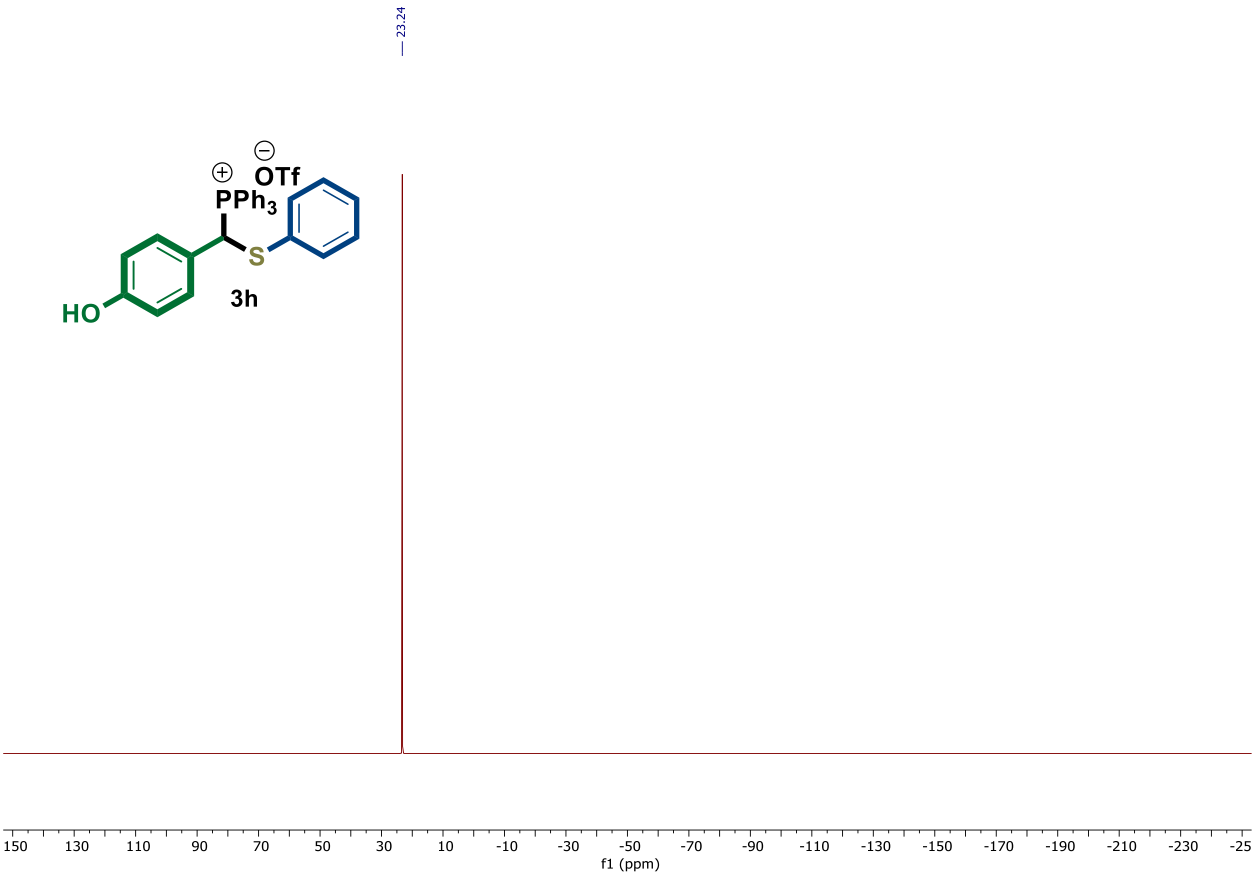

^{19}F NMR (376 MHz, DMSO-d6) of **3h**

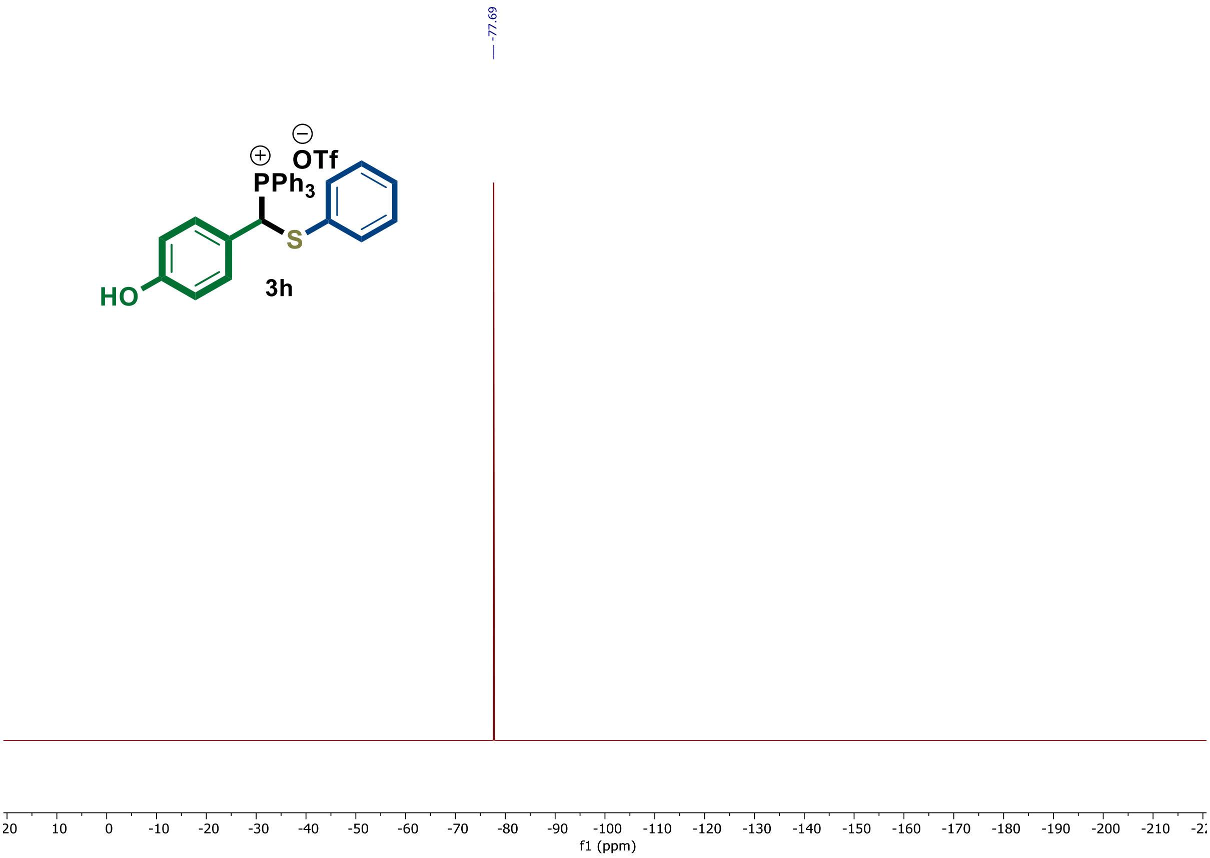

^{1}H NMR (400 MHz, $CDCl_3$) of **3i**

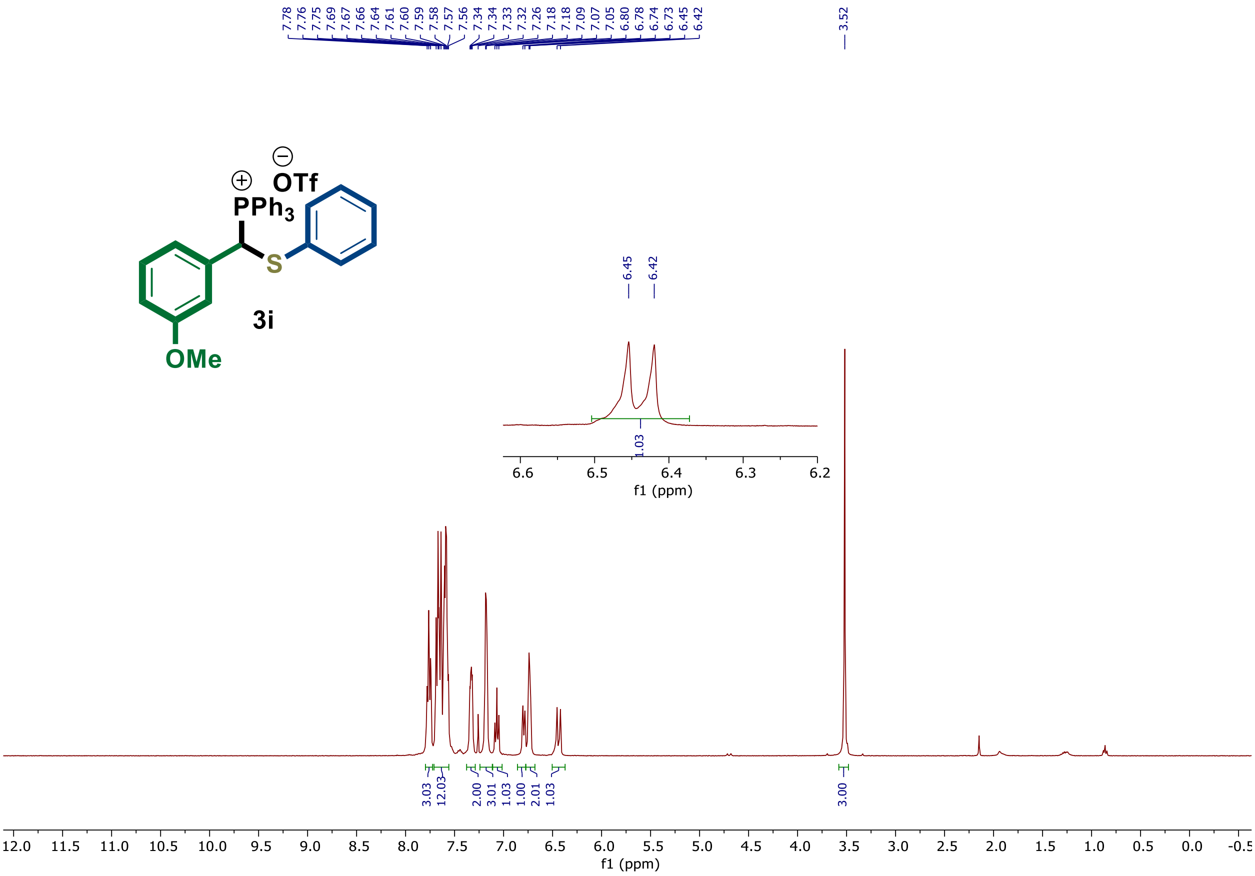

^{13}C NMR (100 MHz, $CDCl_3$) of **3i**

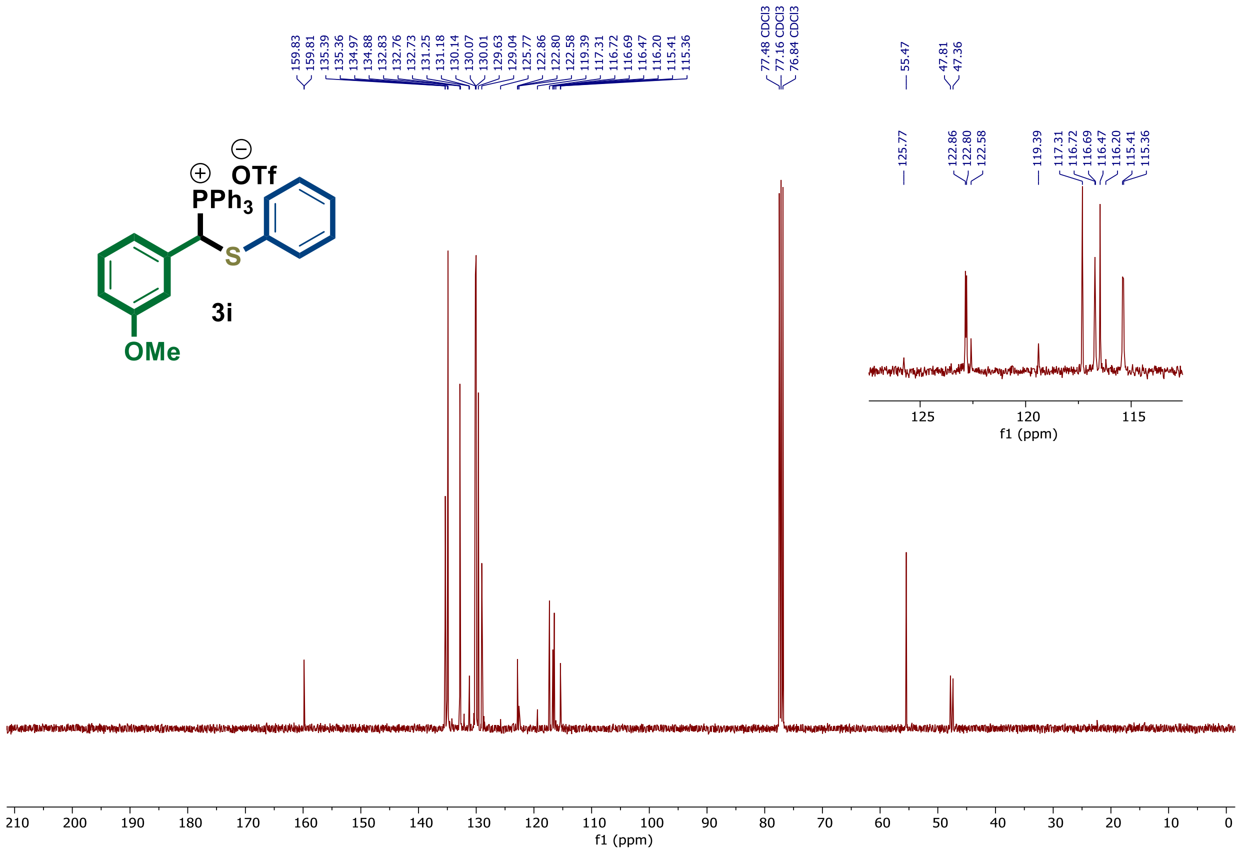

^{31}P NMR (162 MHz, $CDCl_3$) of **3i**

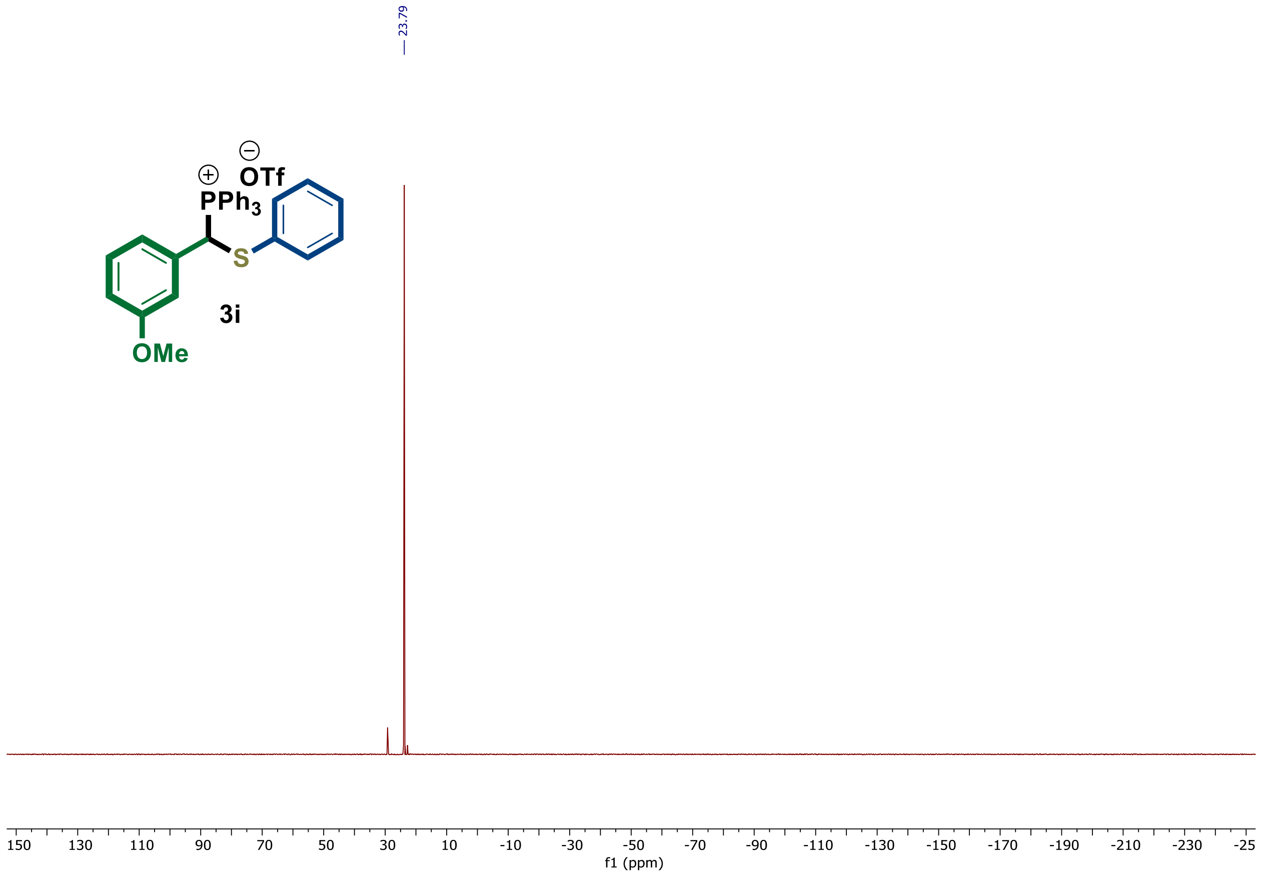

^{19}F NMR (376 MHz, CDCl$_3$) of **3i**

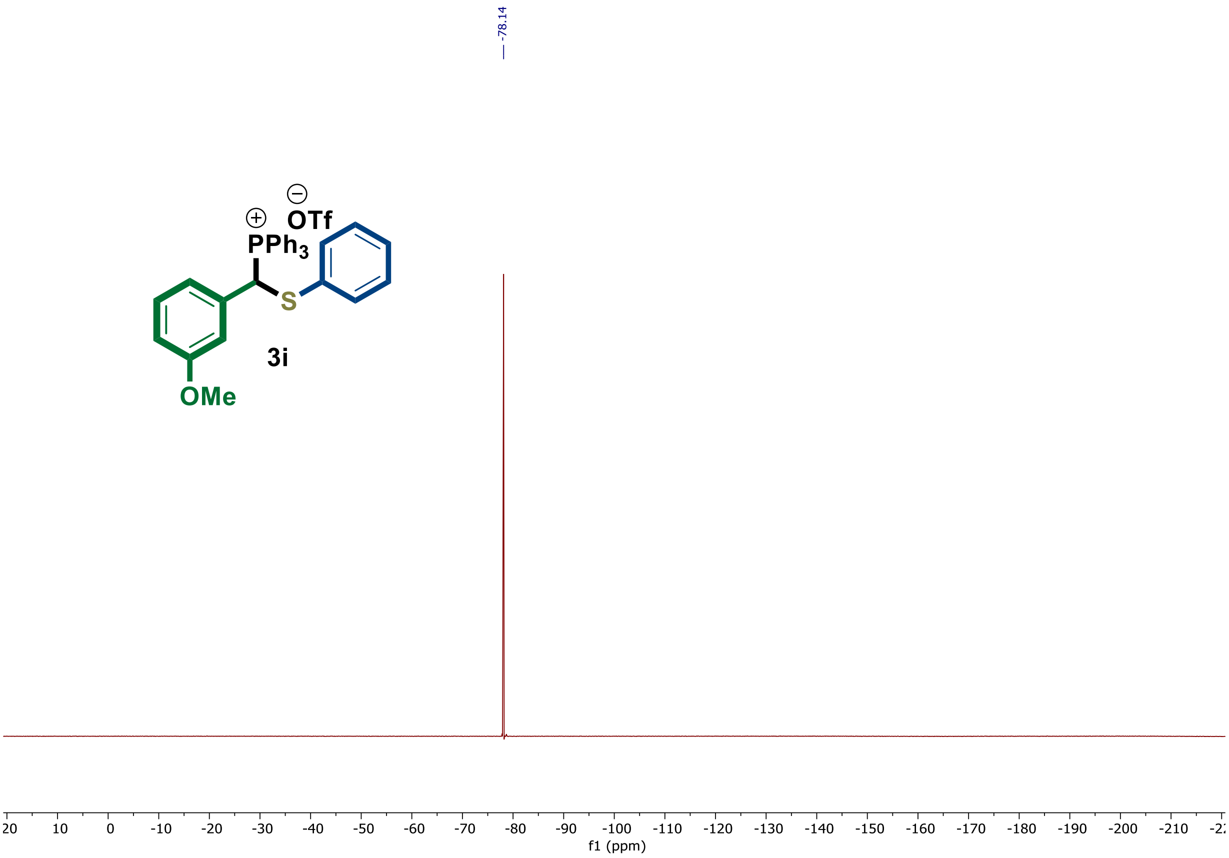

^{1}H NMR (400 MHz, CDCl$_3$) of **3j**

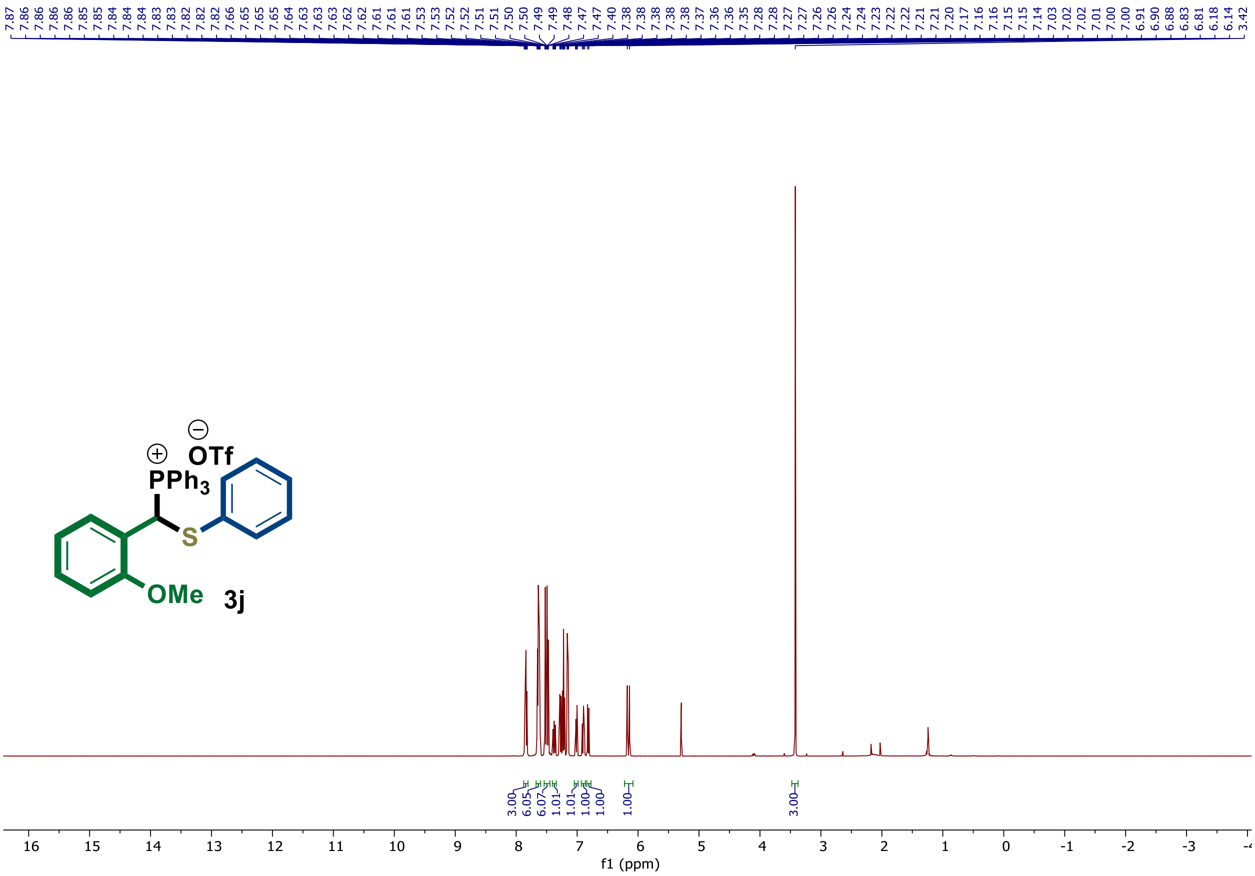

^{13}C NMR (100 MHz, $CDCl_3$) of **3j**

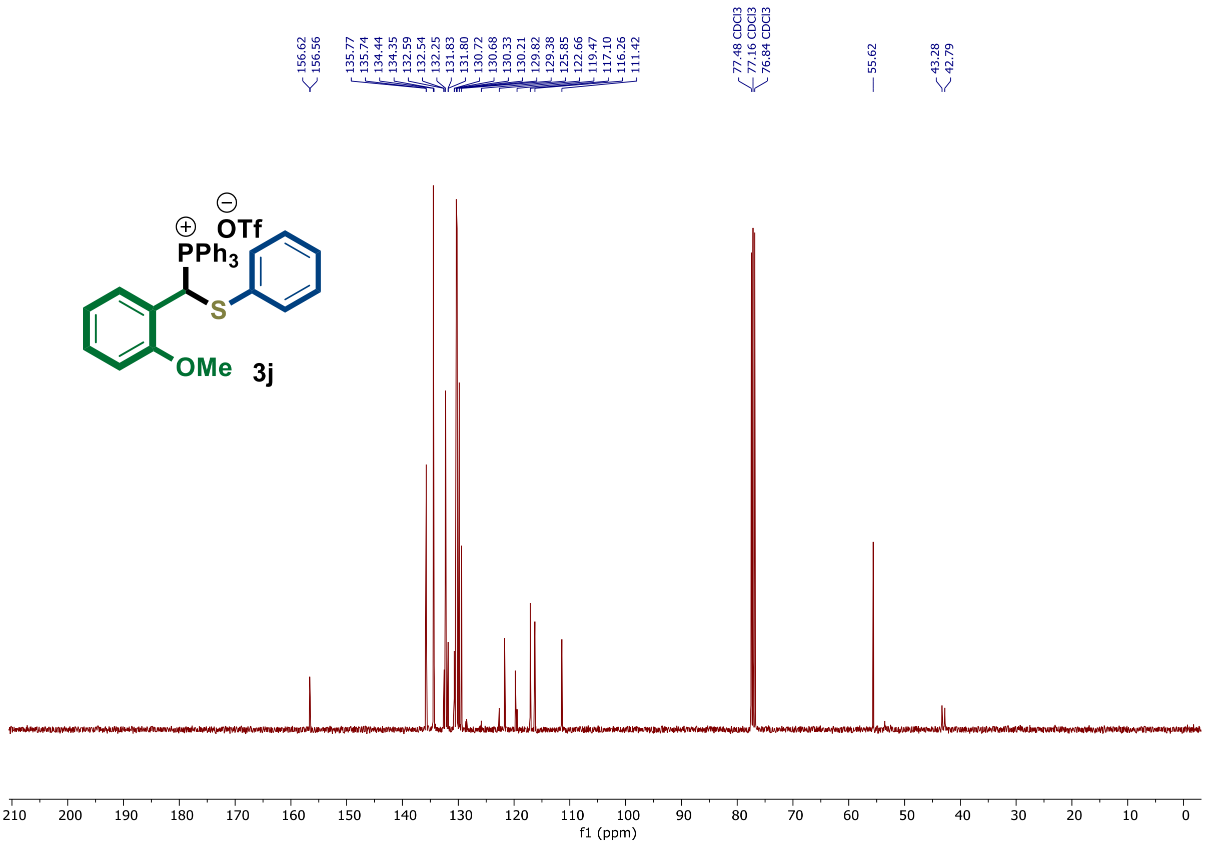

^{31}P NMR (162 MHz, $CDCl_3$) of **3j**

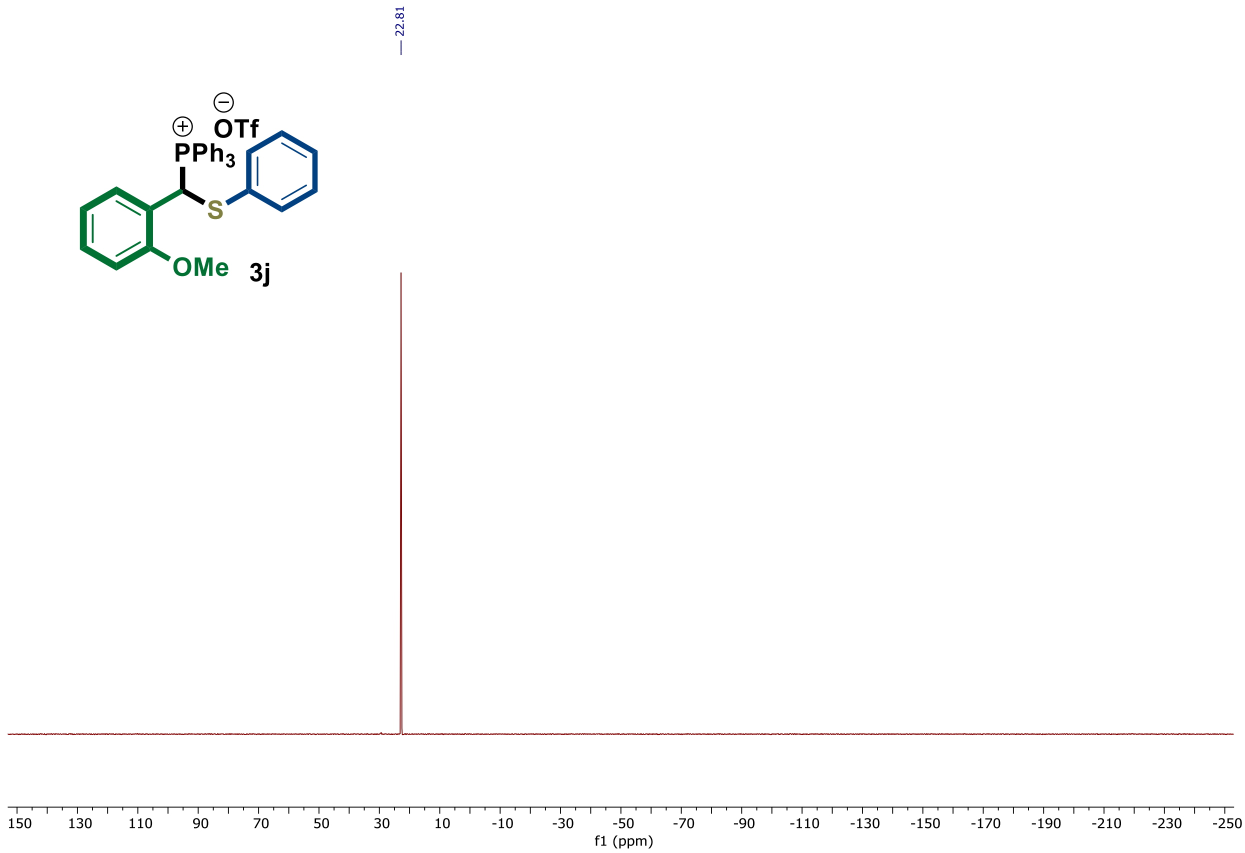

^{19}F NMR (376 MHz, CDCl$_3$) of **3j**

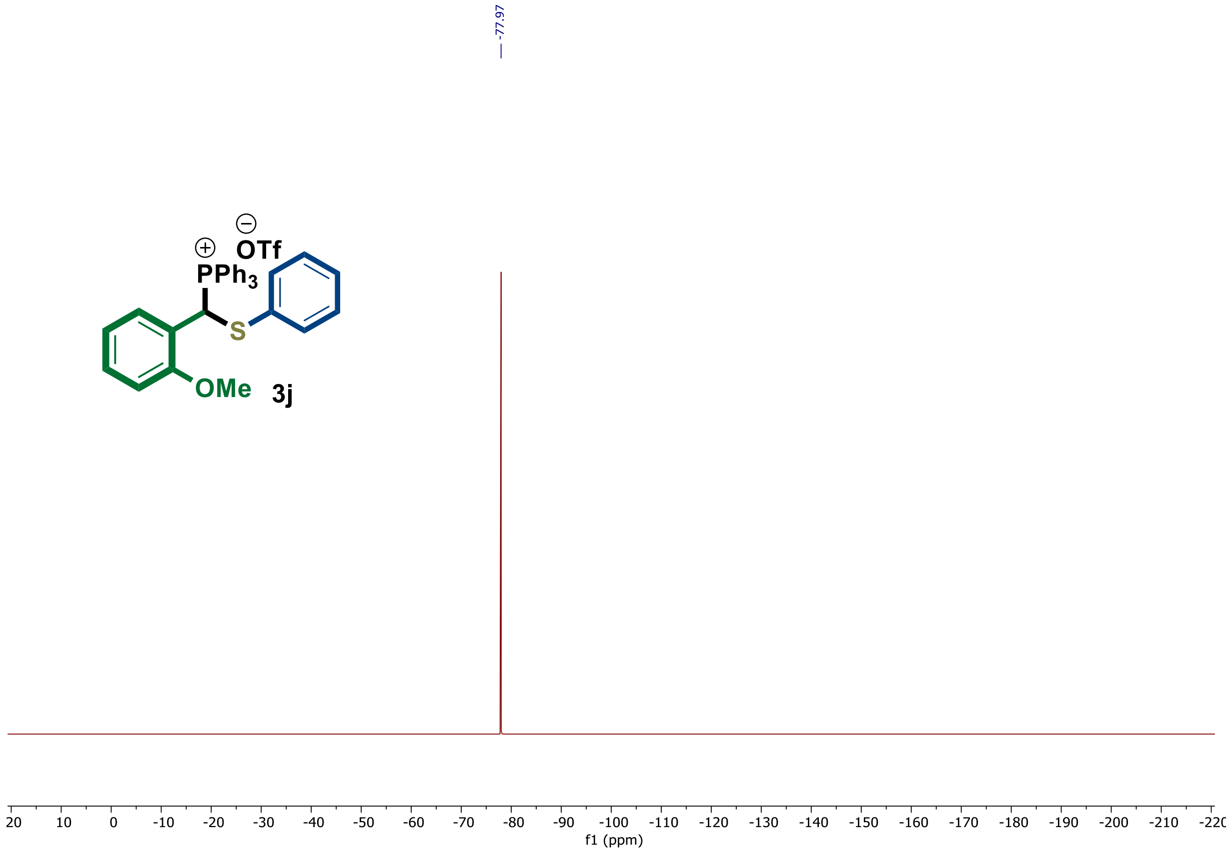

^{1}H NMR (400 MHz, CDCl$_3$) of **3k**

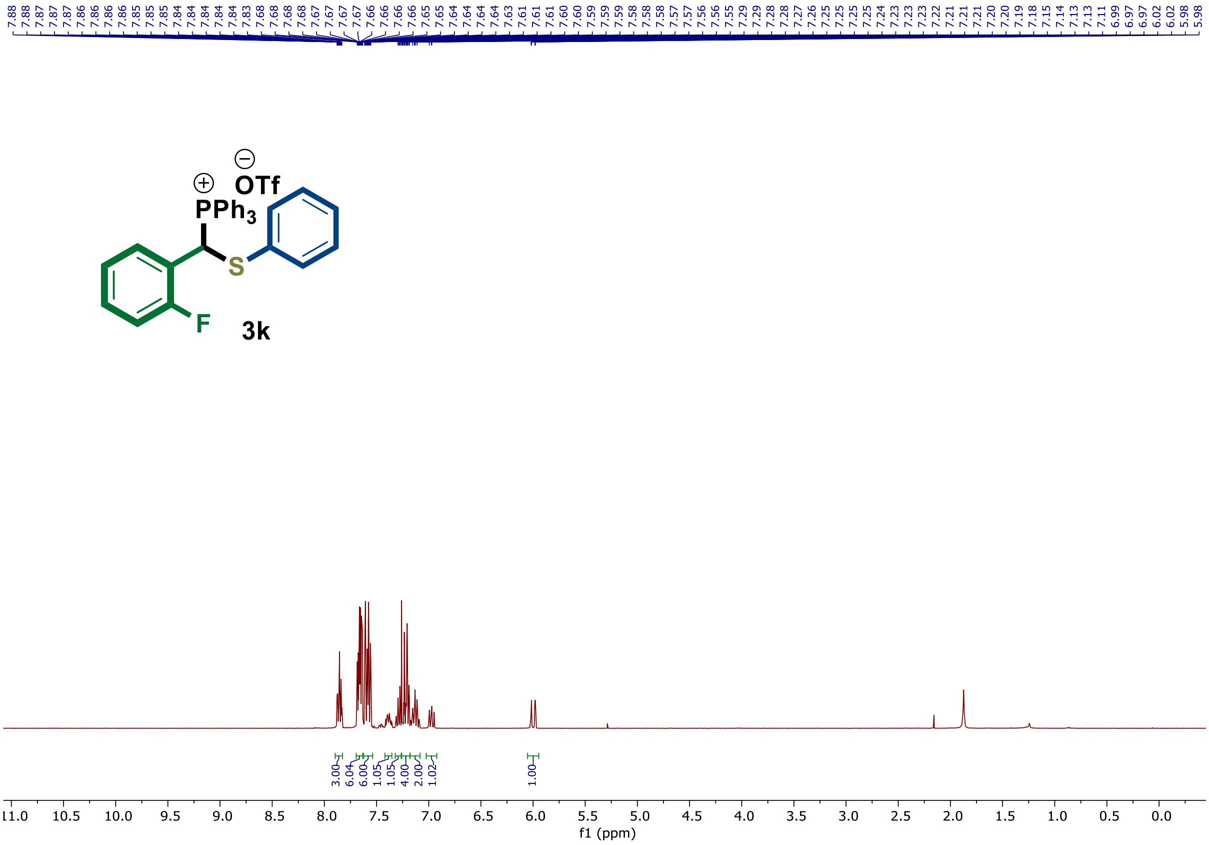

^{13}C NMR (100 MHz, $CDCl_3$) of **3k**

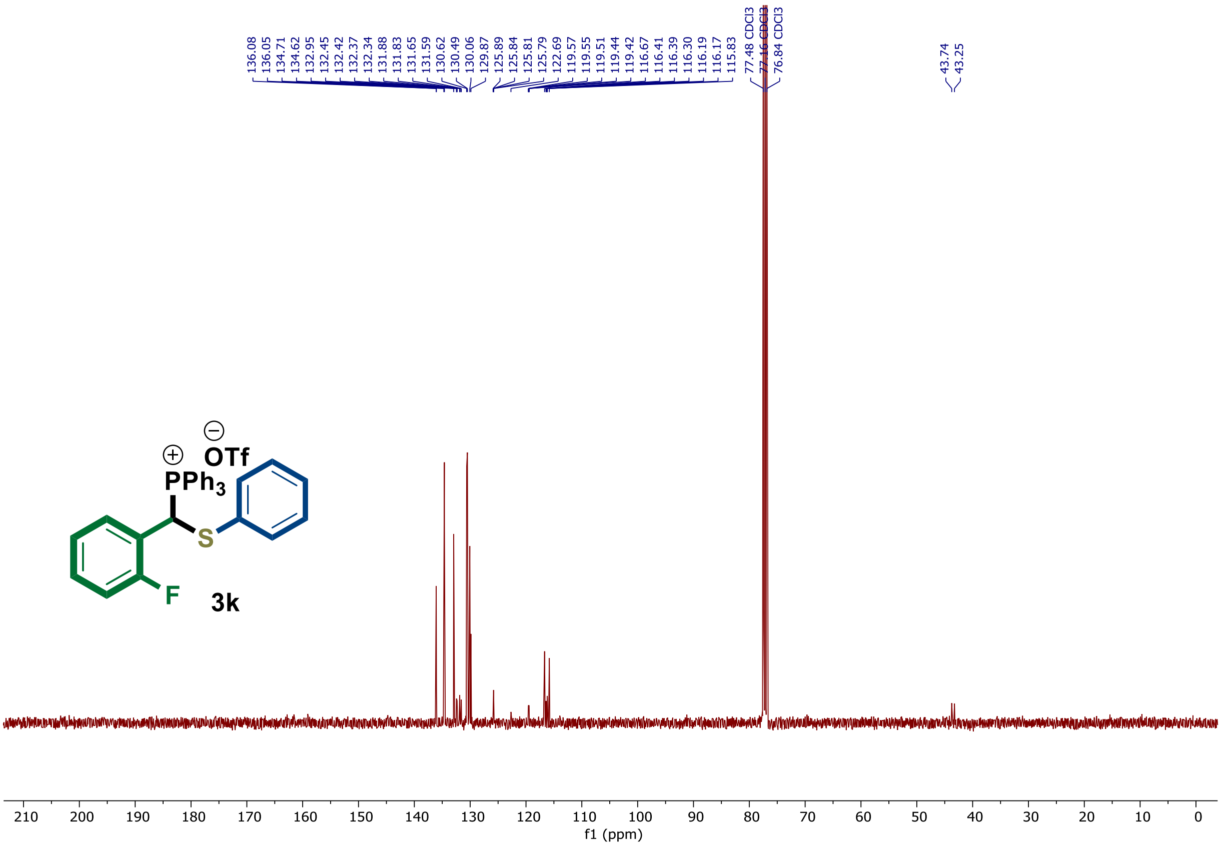

^{31}P NMR (162 MHz, $CDCl_3$) of **3k**

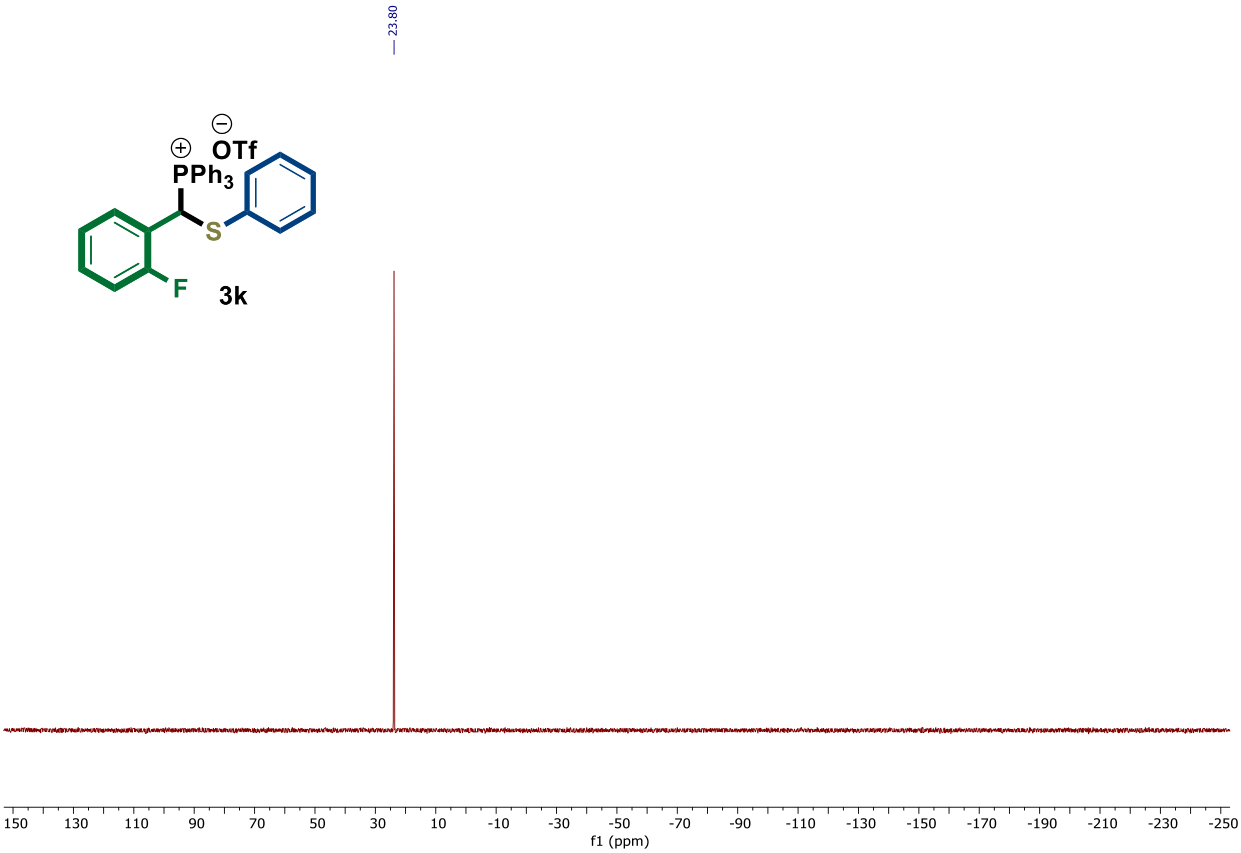

^{19}F NMR (376 MHz, CDCl$_3$) of **3k**

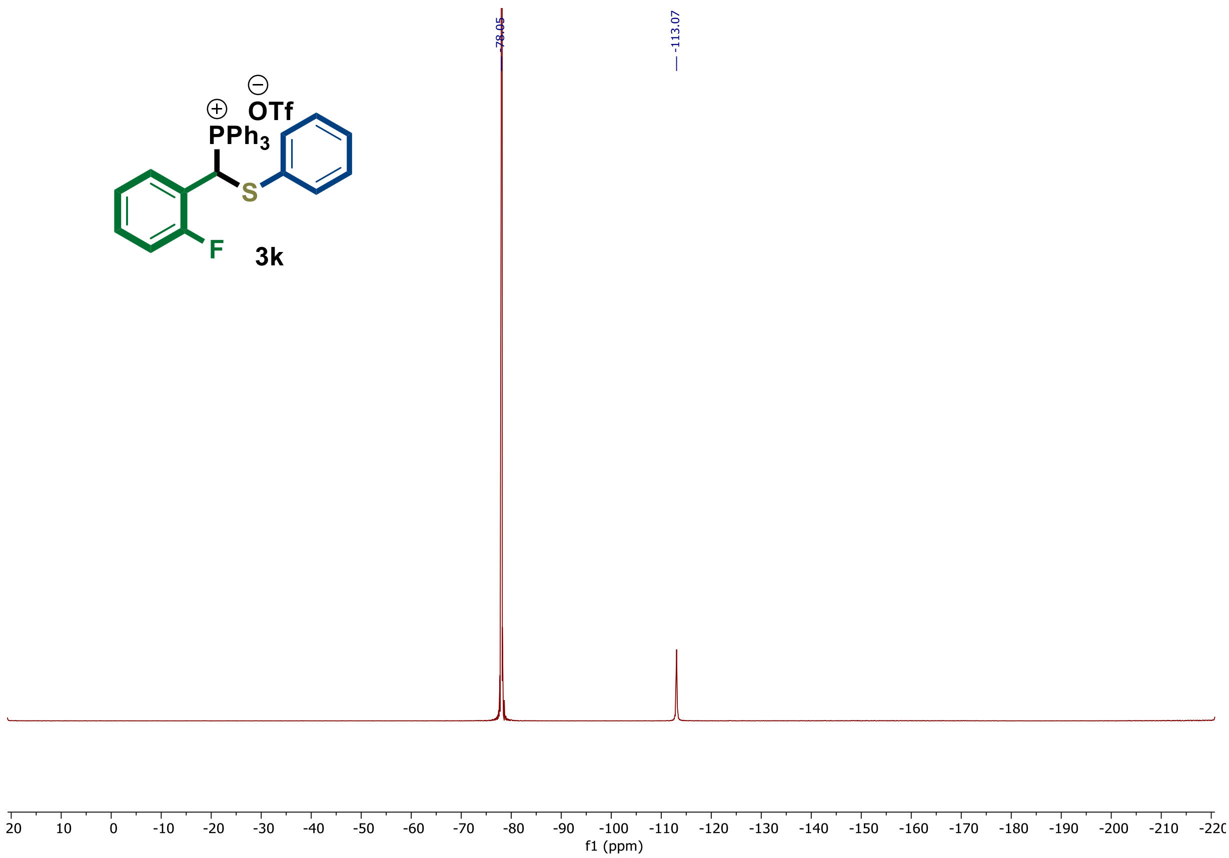

^{1}H NMR (400 MHz, CDCl$_3$) of **3l**

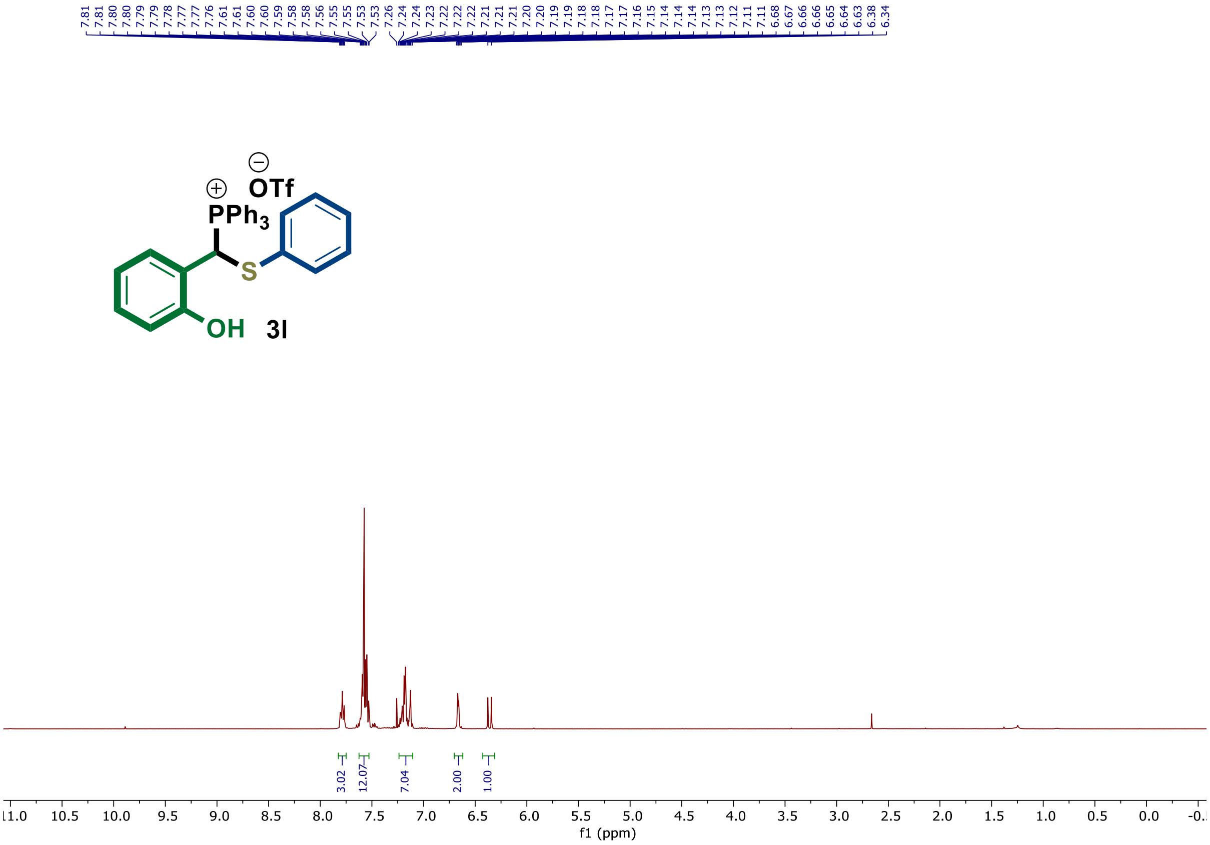

^{13}C NMR (100 MHz, $CDCl_3$) of **3l**

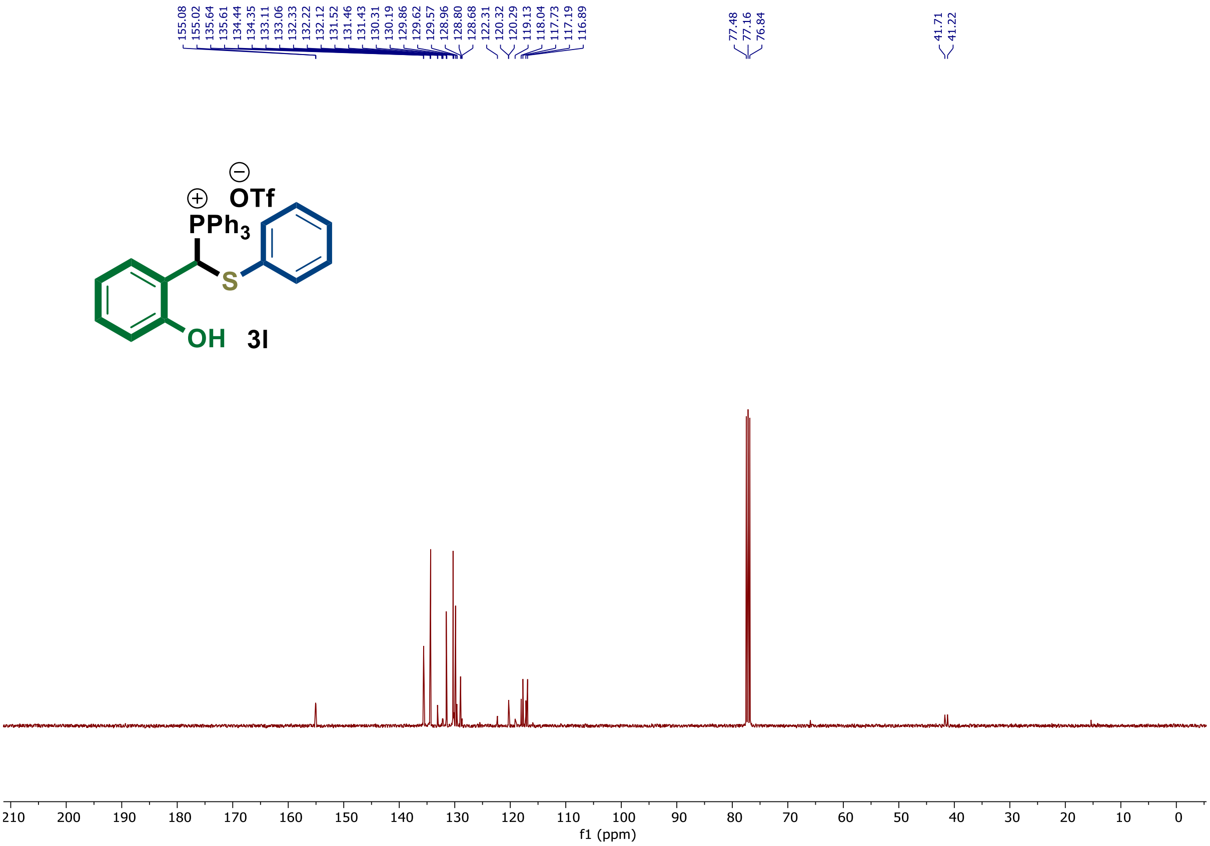

^{31}P NMR (162 MHz, $CDCl_3$) of **3l**

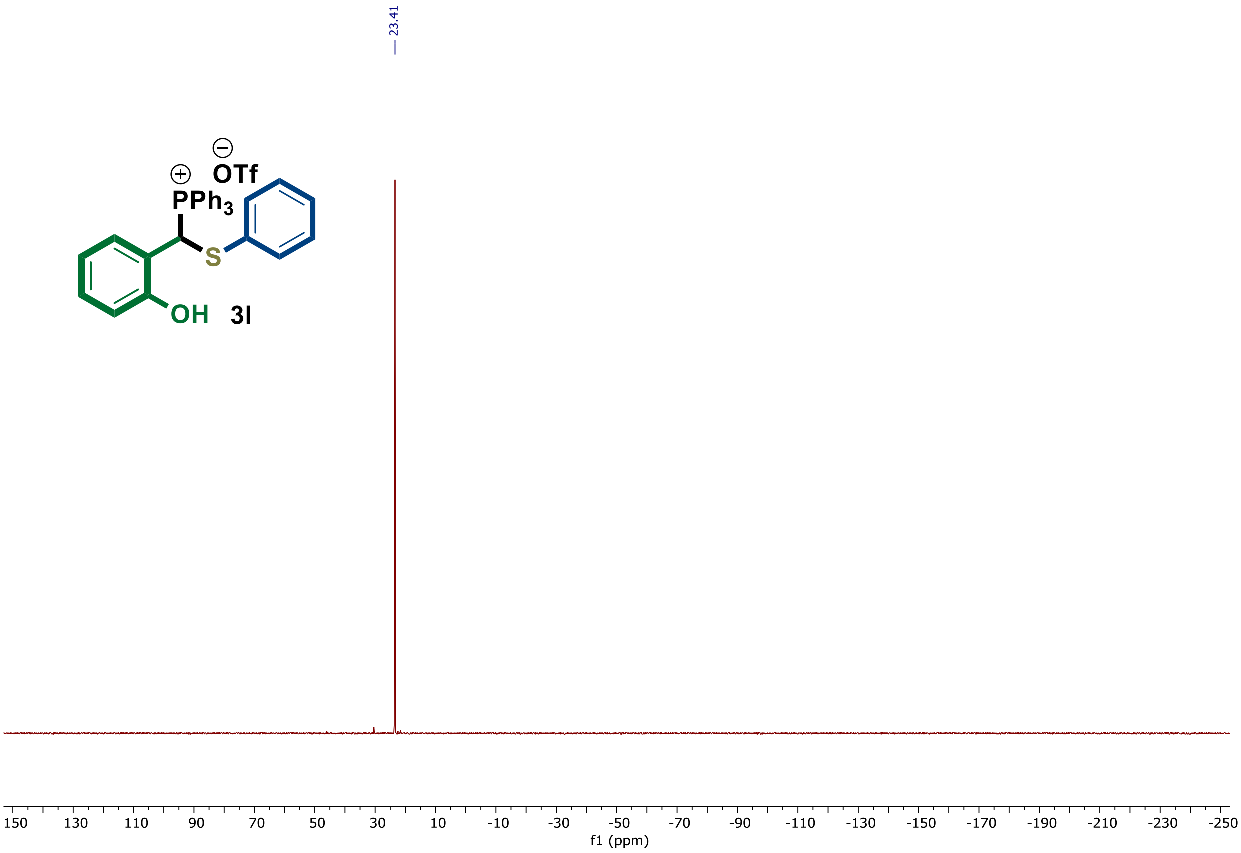

^{19}F NMR (376 MHz, $CDCl_3$) of **3l**

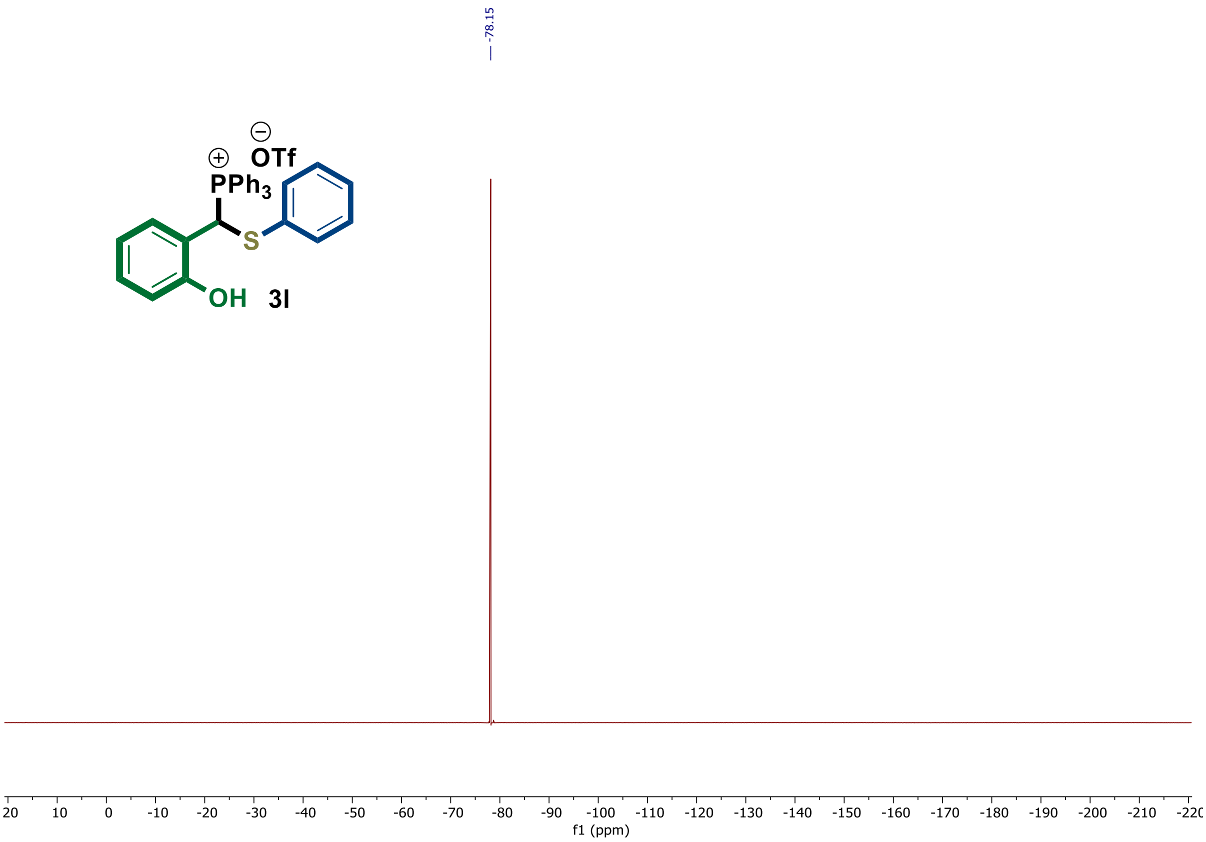

^{1}H NMR (400 MHz, $CDCl_3$) of **3m**

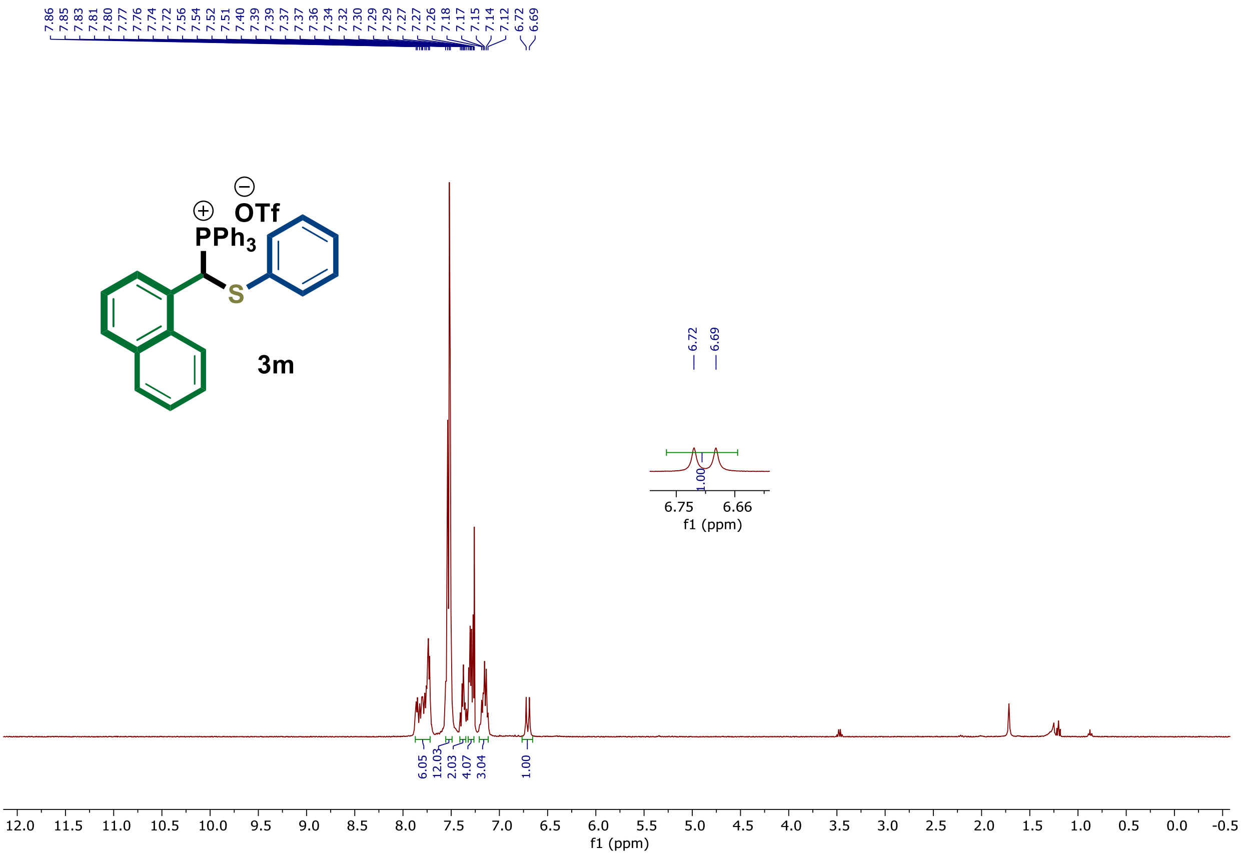

^{13}C NMR (100 MHz, CDCl$_3$) of **3m**

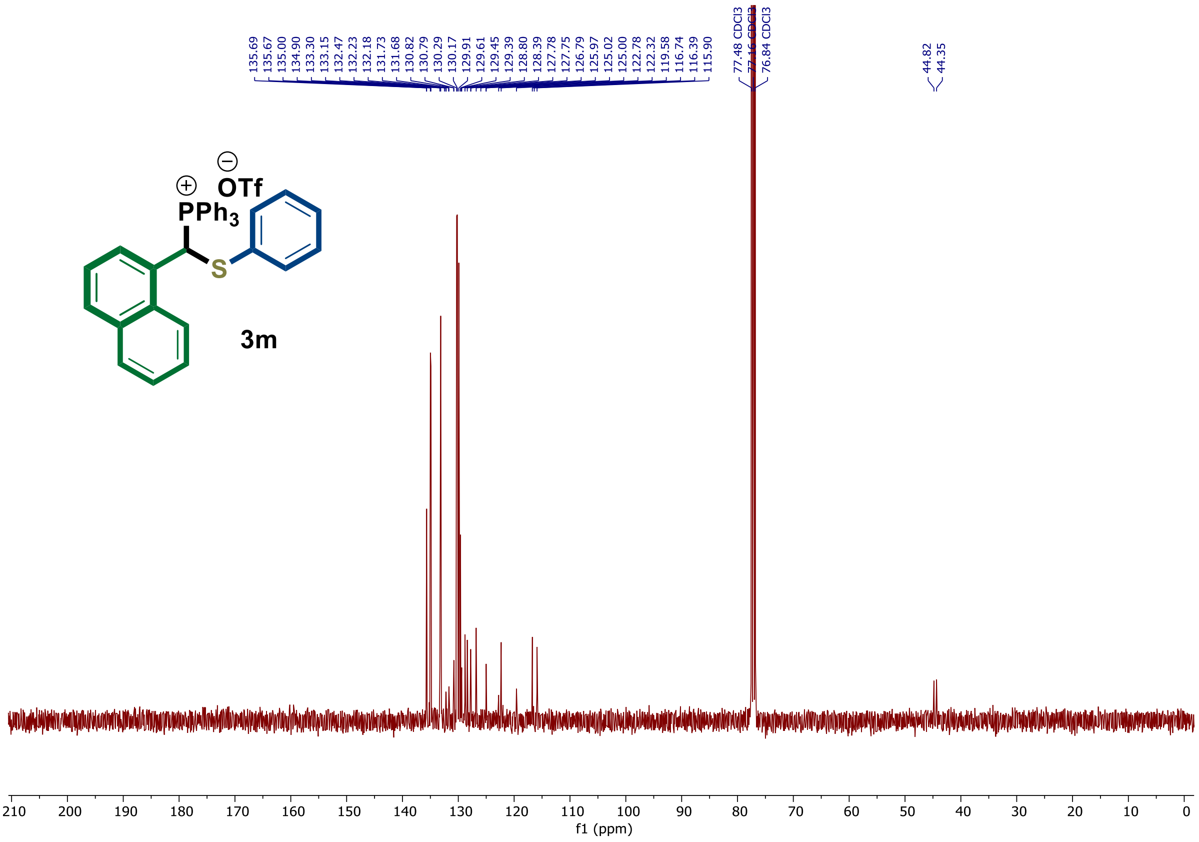

^{31}P NMR (162 MHz, CDCl$_3$) of **3m**

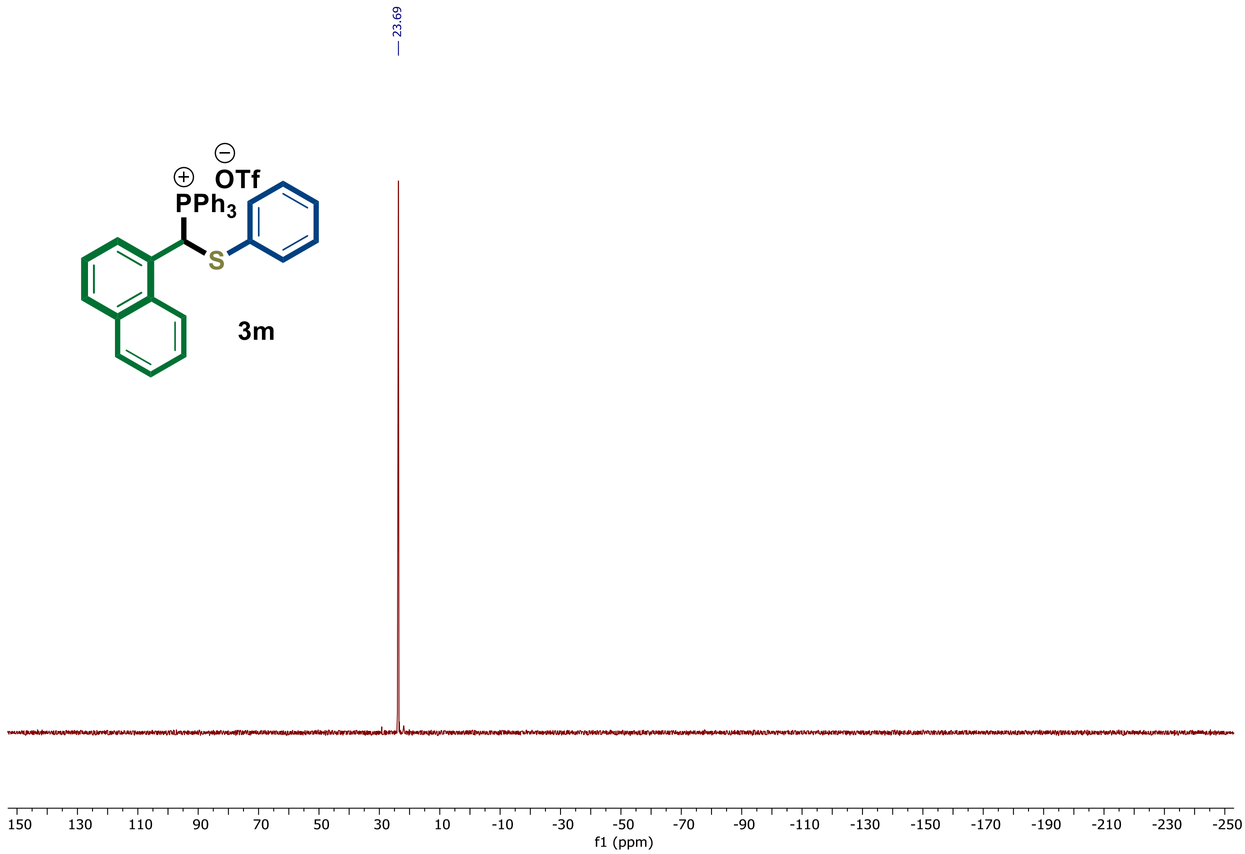

^{19}F NMR (376 MHz, $CDCl_3$) of **3m**

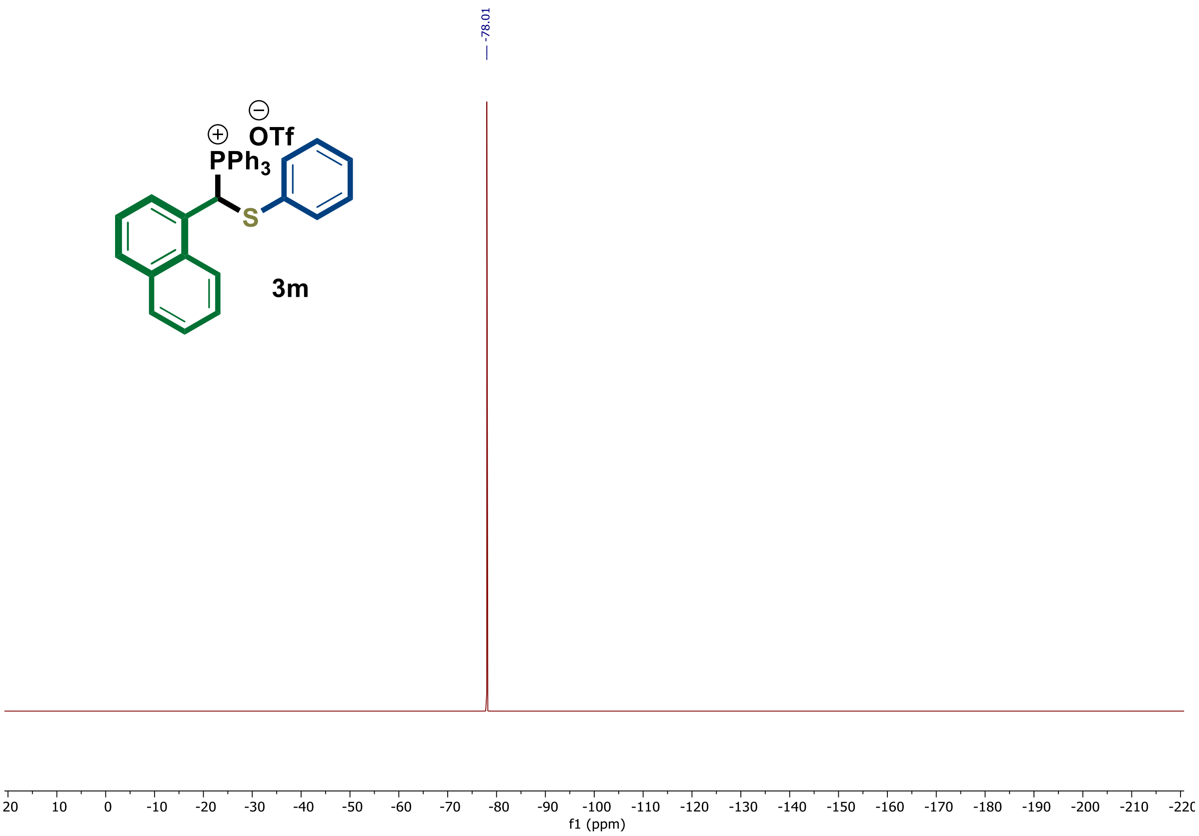

^{1}H NMR (400 MHz, $CDCl_3$) of **3n**

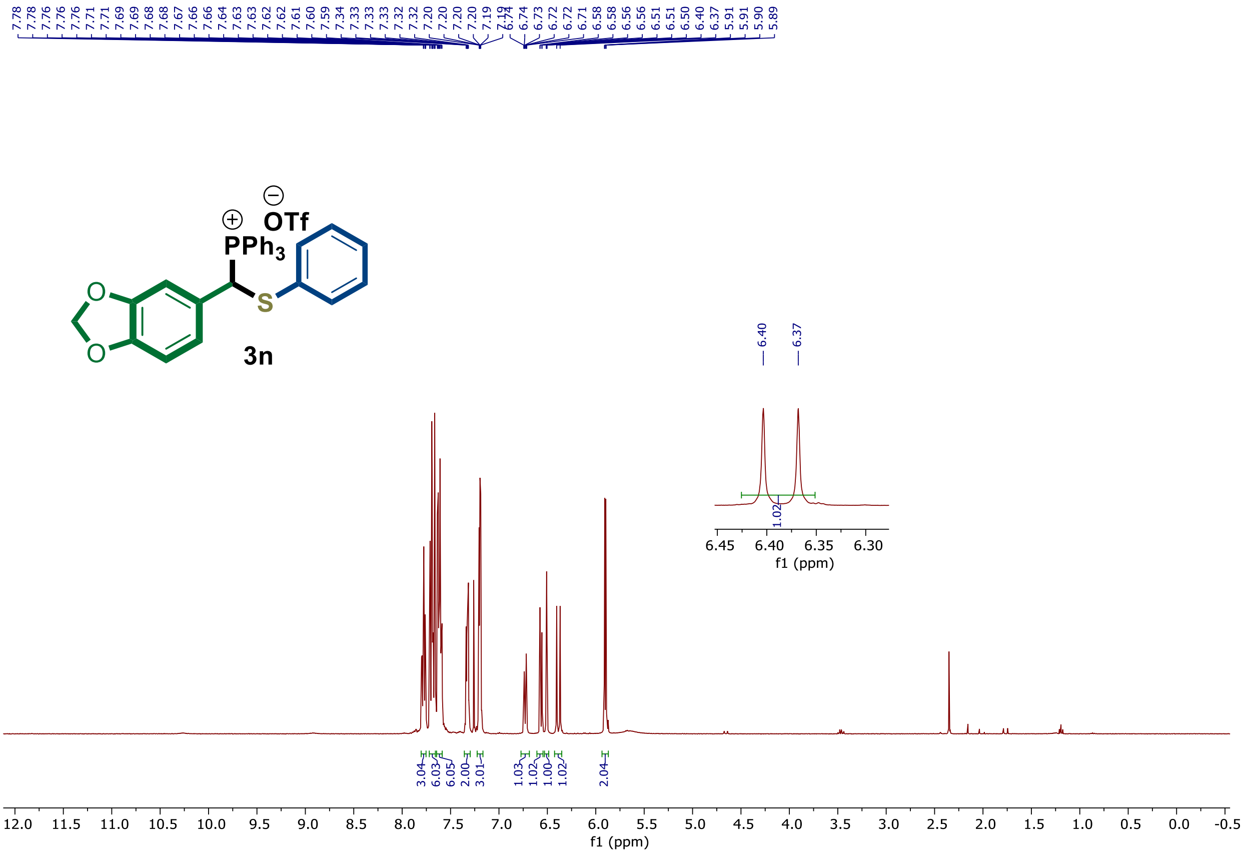

^{13}C NMR (100 MHz, CDCl$_3$) of **3n**

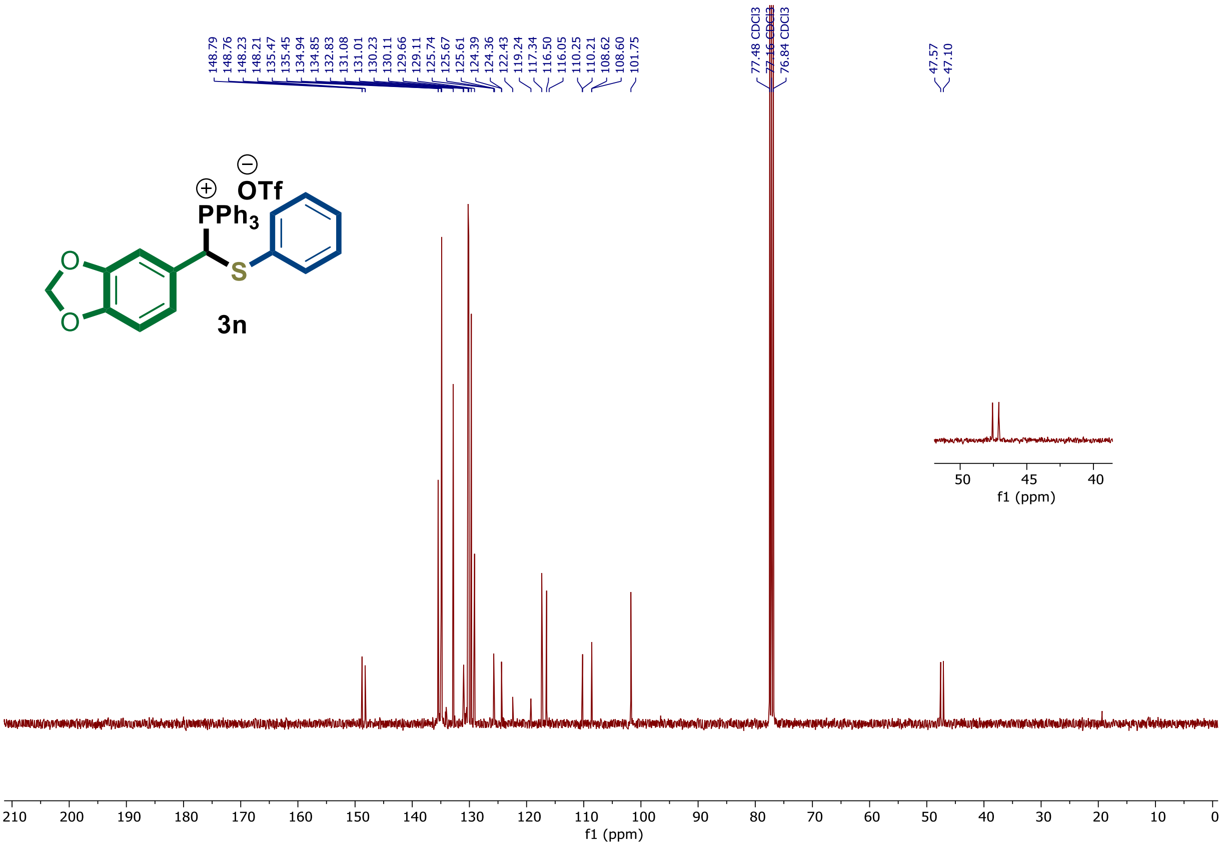

^{31}P NMR (162 MHz, CDCl$_3$) of **3n**

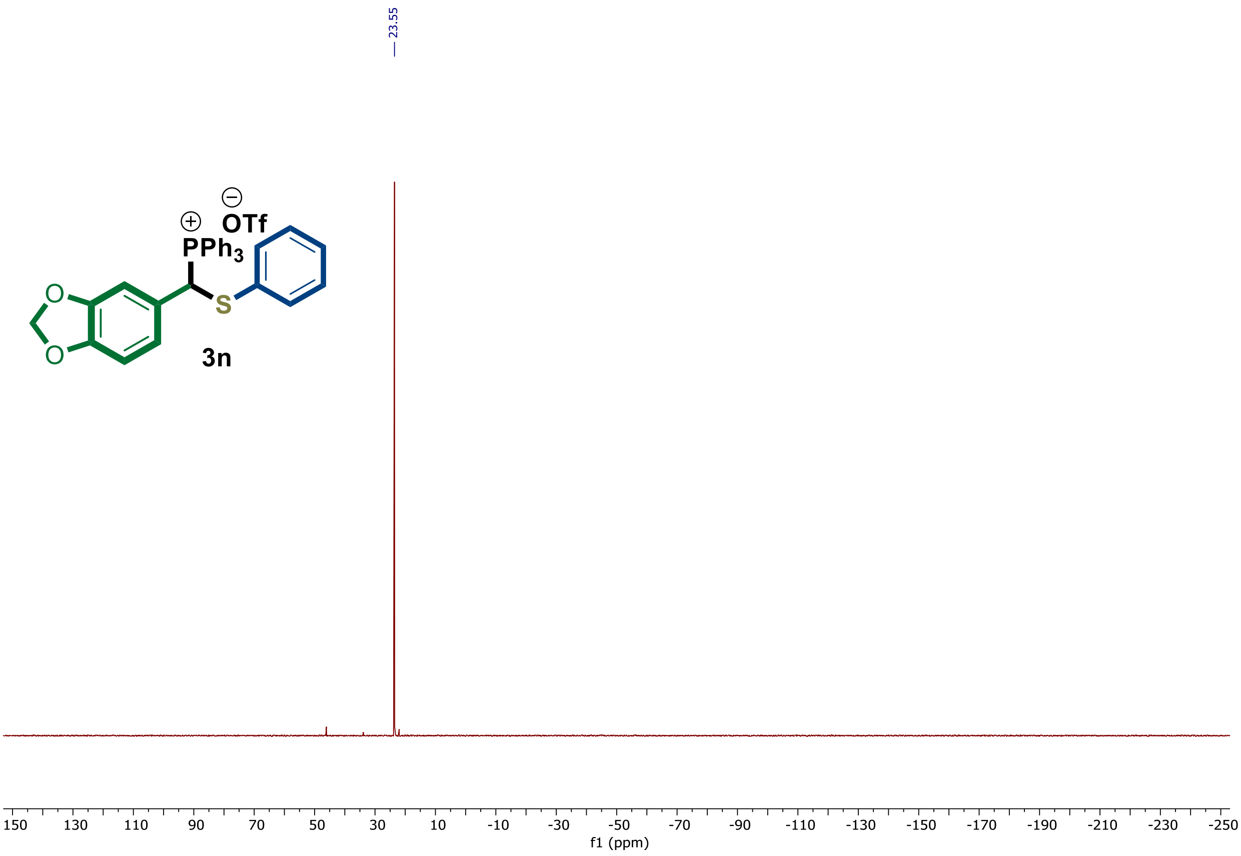

^{19}F NMR (376 MHz, $CDCl_3$) of **3n**

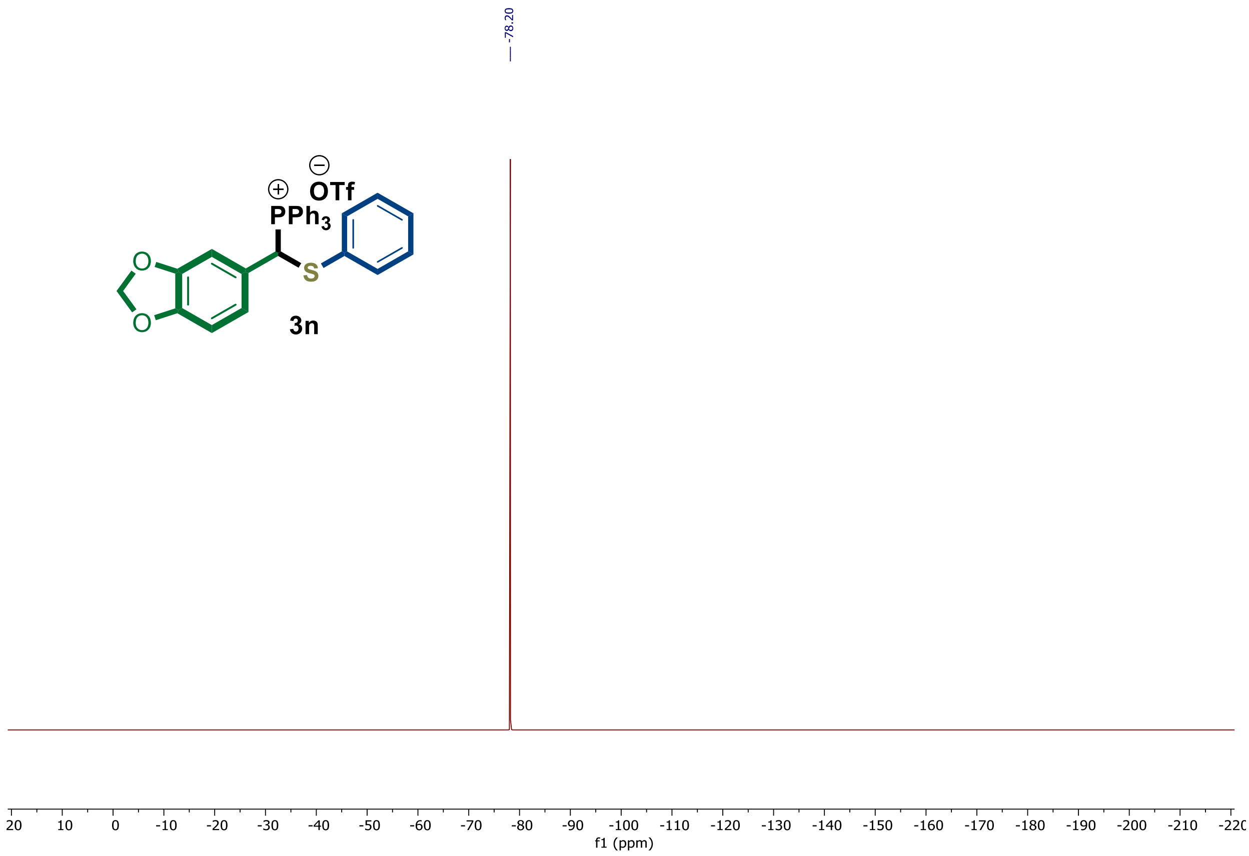

^{1}H NMR (400 MHz, $CDCl_3$) of **3o**

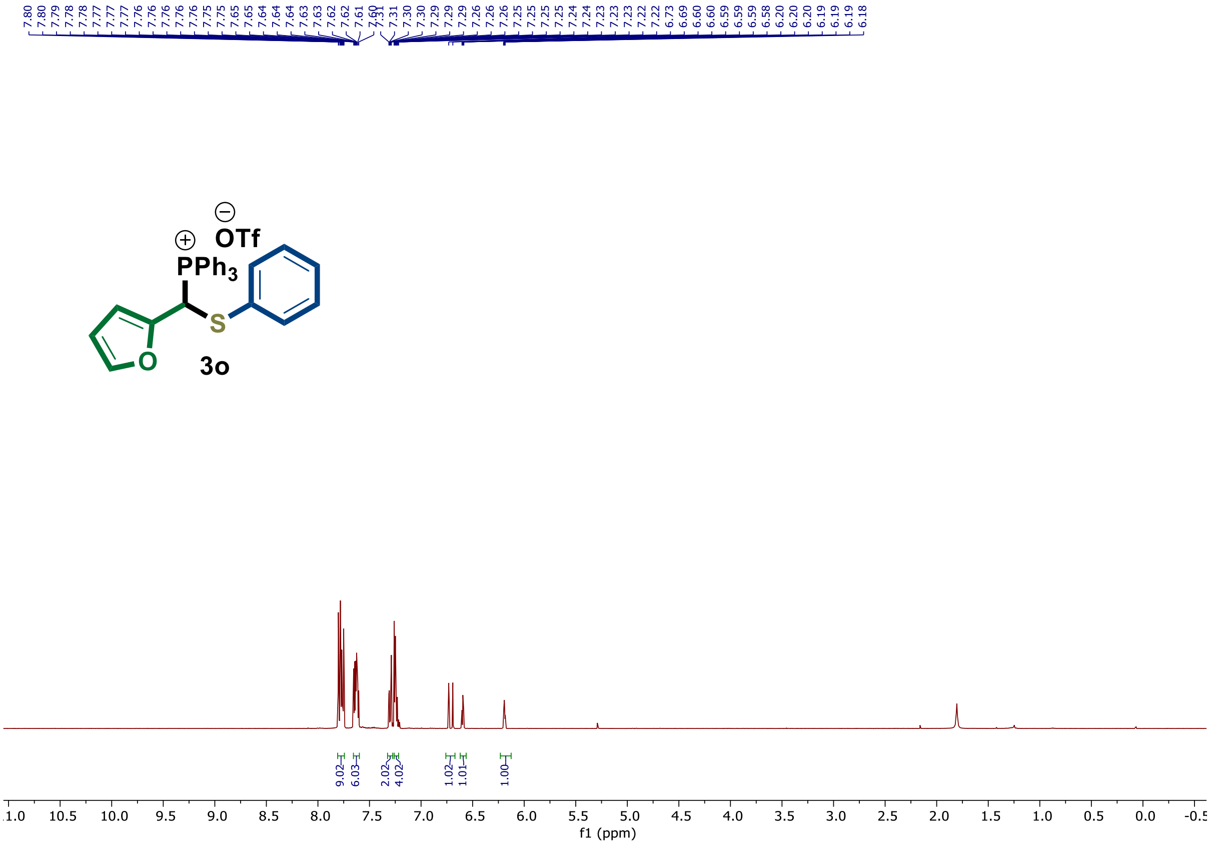

^{13}C NMR (100 MHz, CDCl$_3$) of **3o**

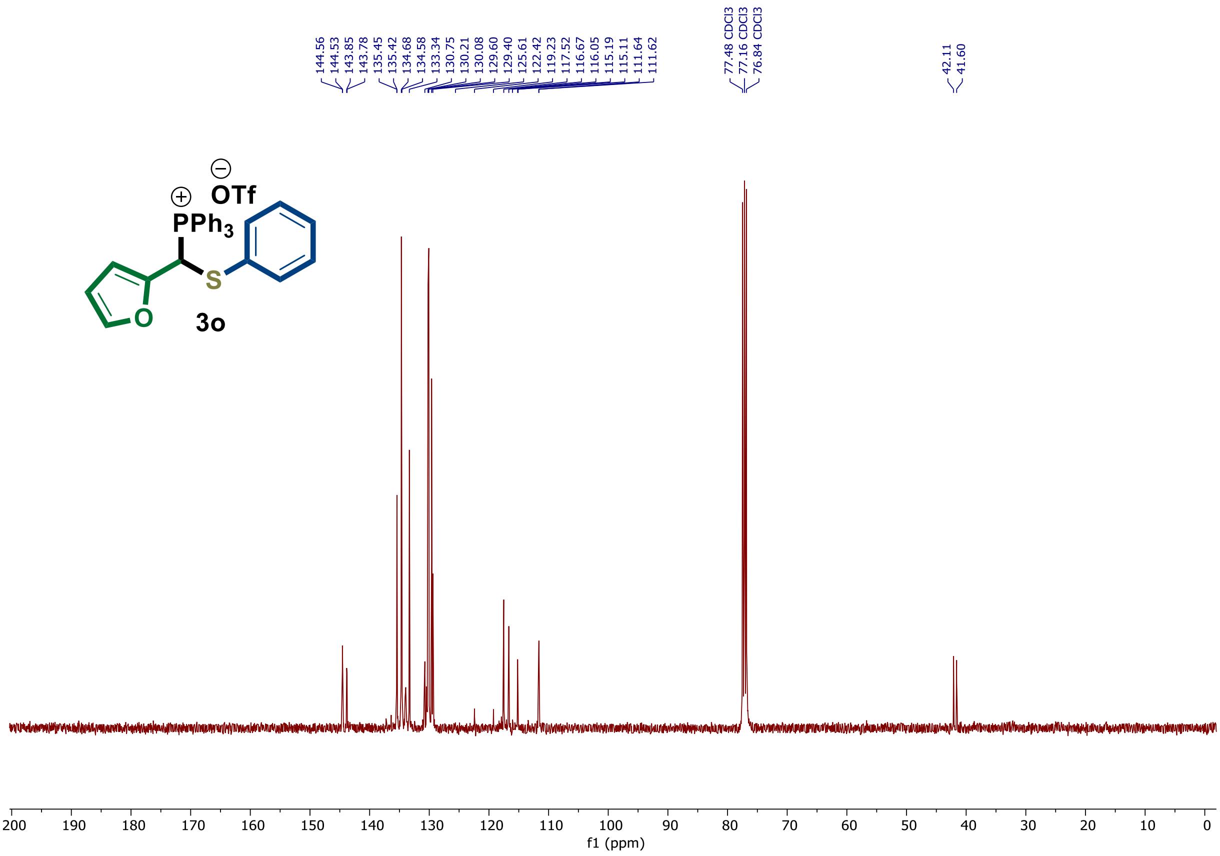

^{31}P NMR (162 MHz, CDCl$_3$) of **3o**

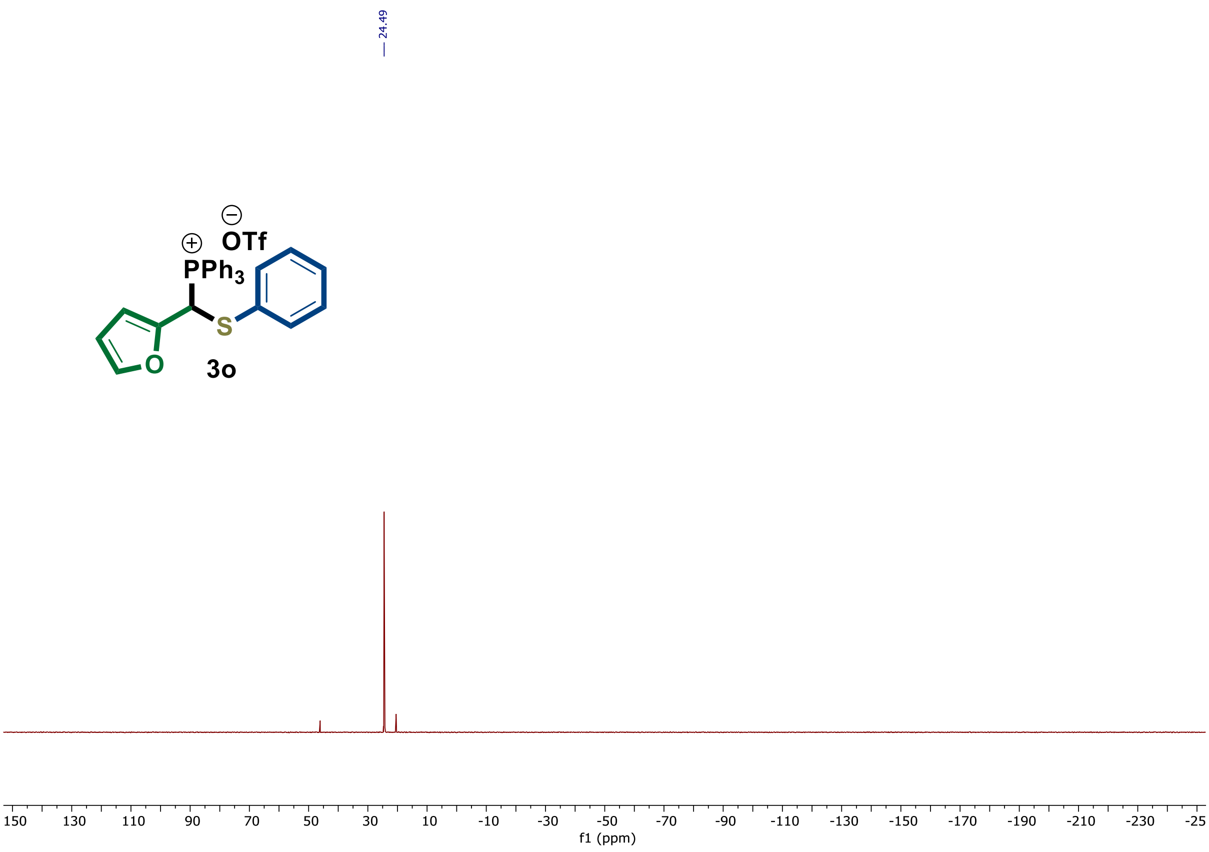

^{19}F NMR (376 MHz, $CDCl_3$) of **3o**

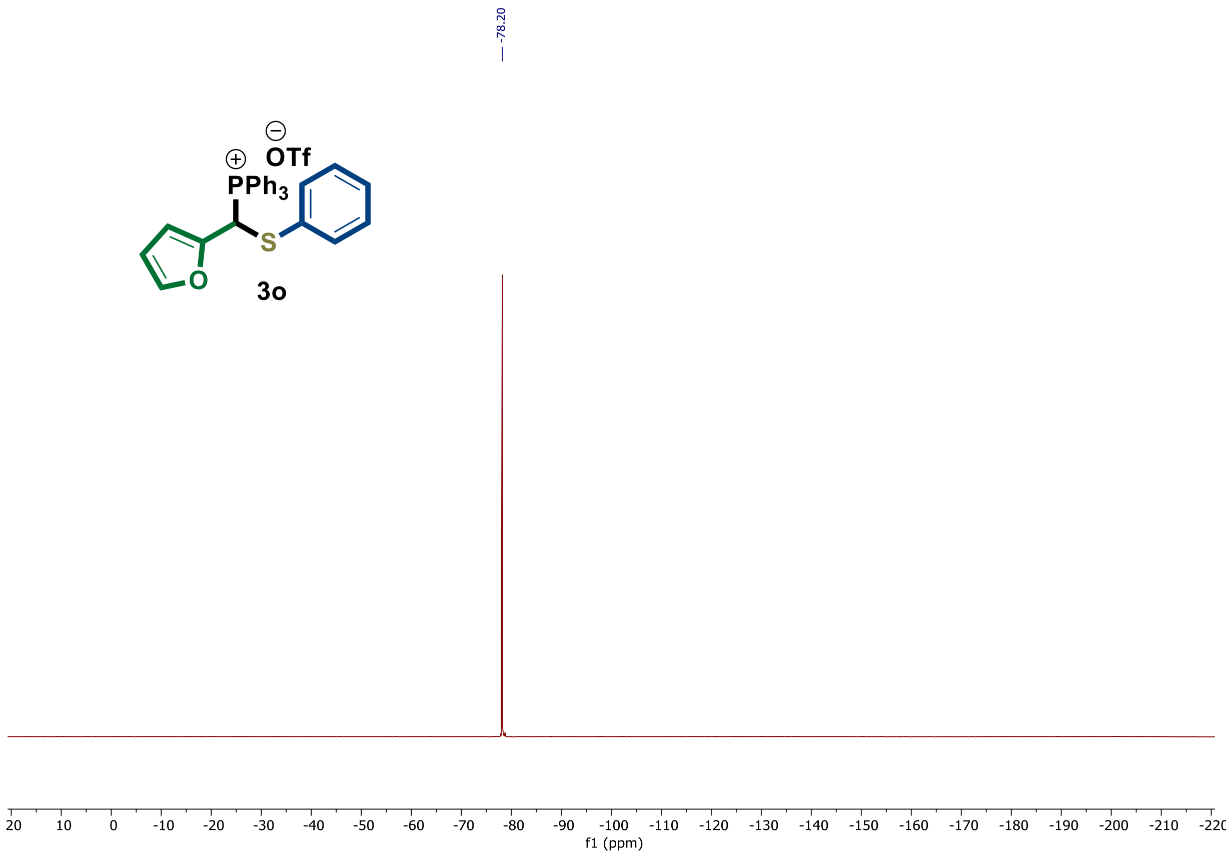

^{1}H NMR (400 MHz, $CDCl_3$) of **3p**

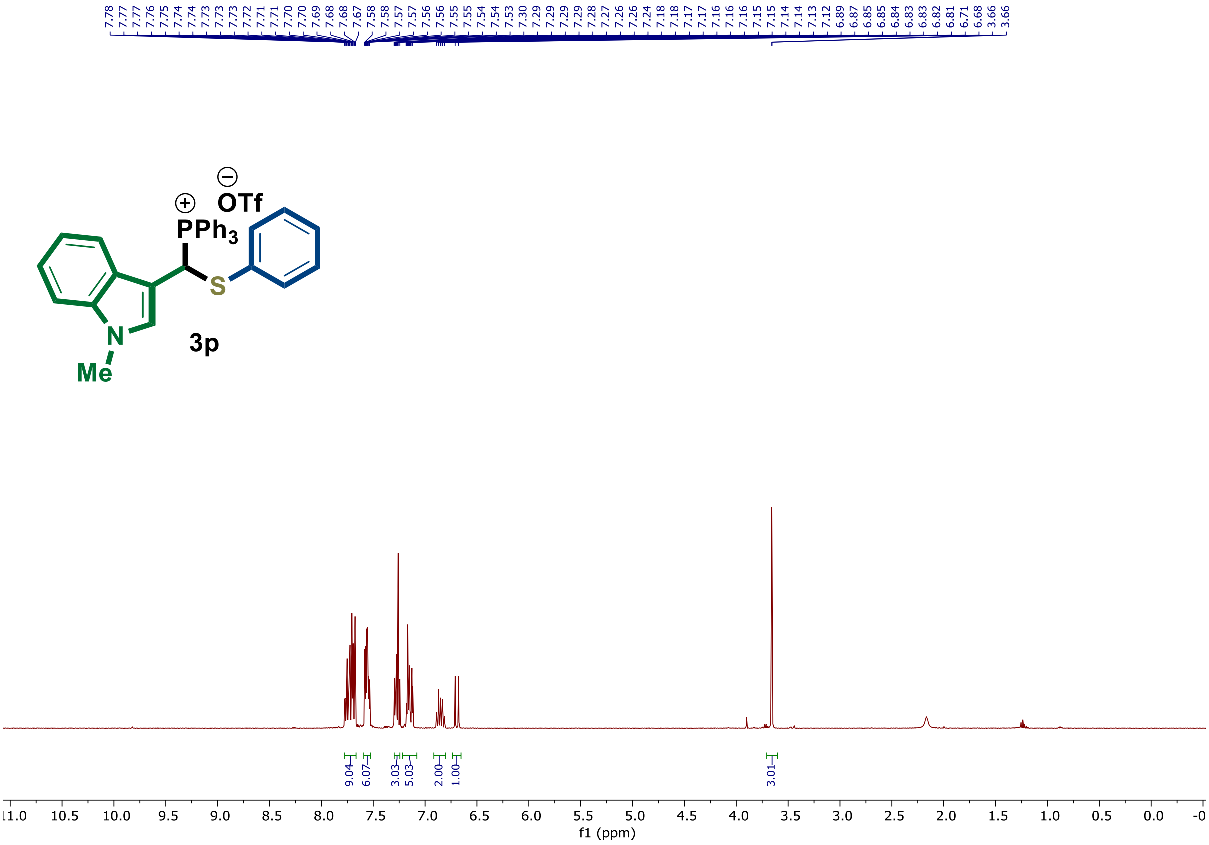

^{13}C NMR (125 MHz, $CDCl_3$) of **3p**

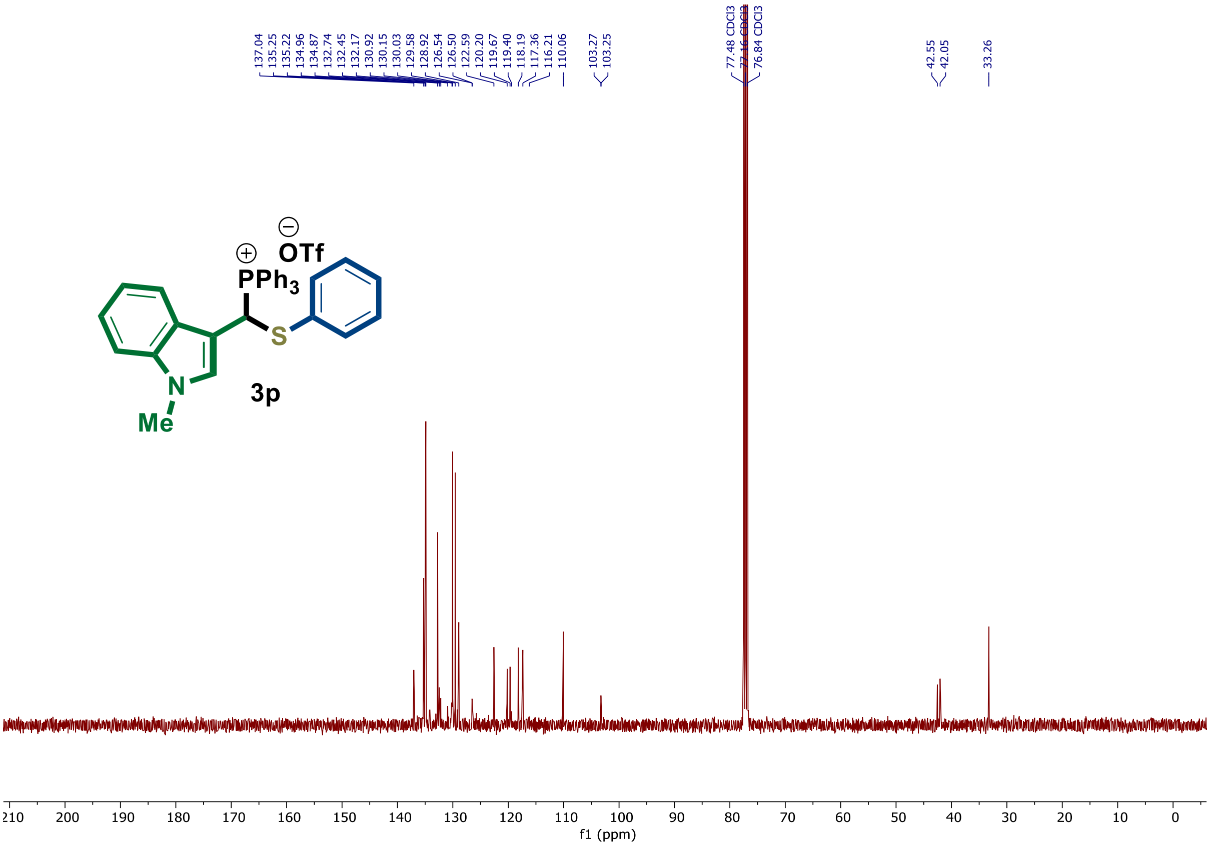

^{31}P NMR (162 MHz, $CDCl_3$) of **3p**

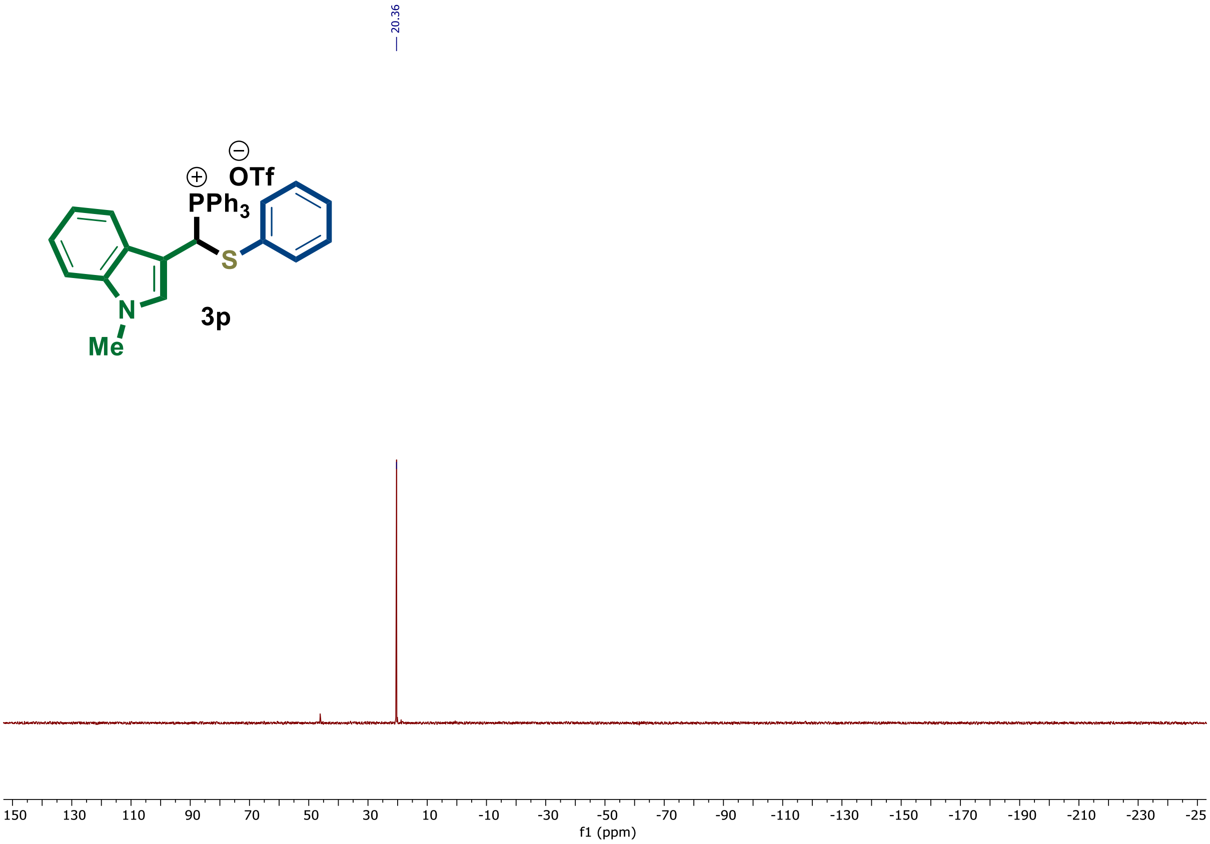

^{19}F NMR (376 MHz, $CDCl_3$) of **3p**

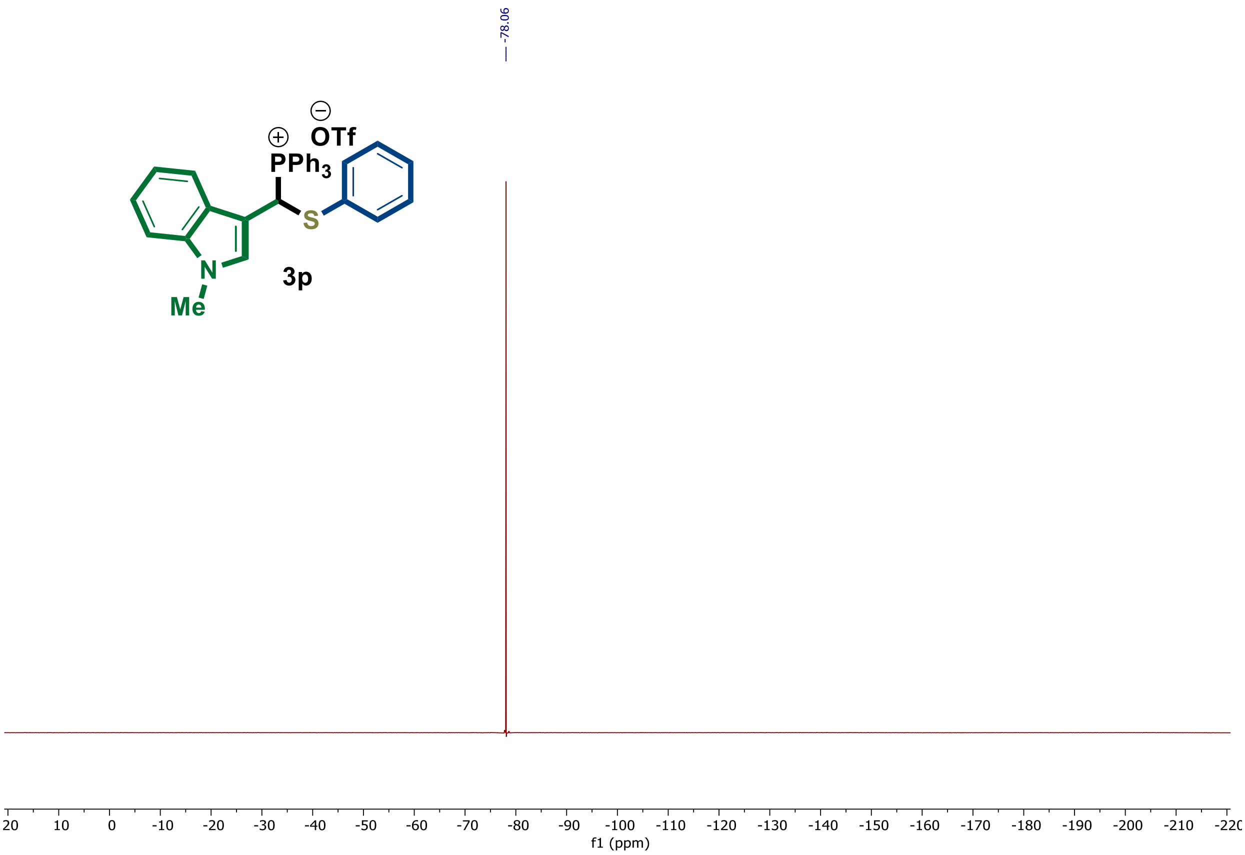

^{1}H NMR (400 MHz, $CDCl_3$) of **3q**

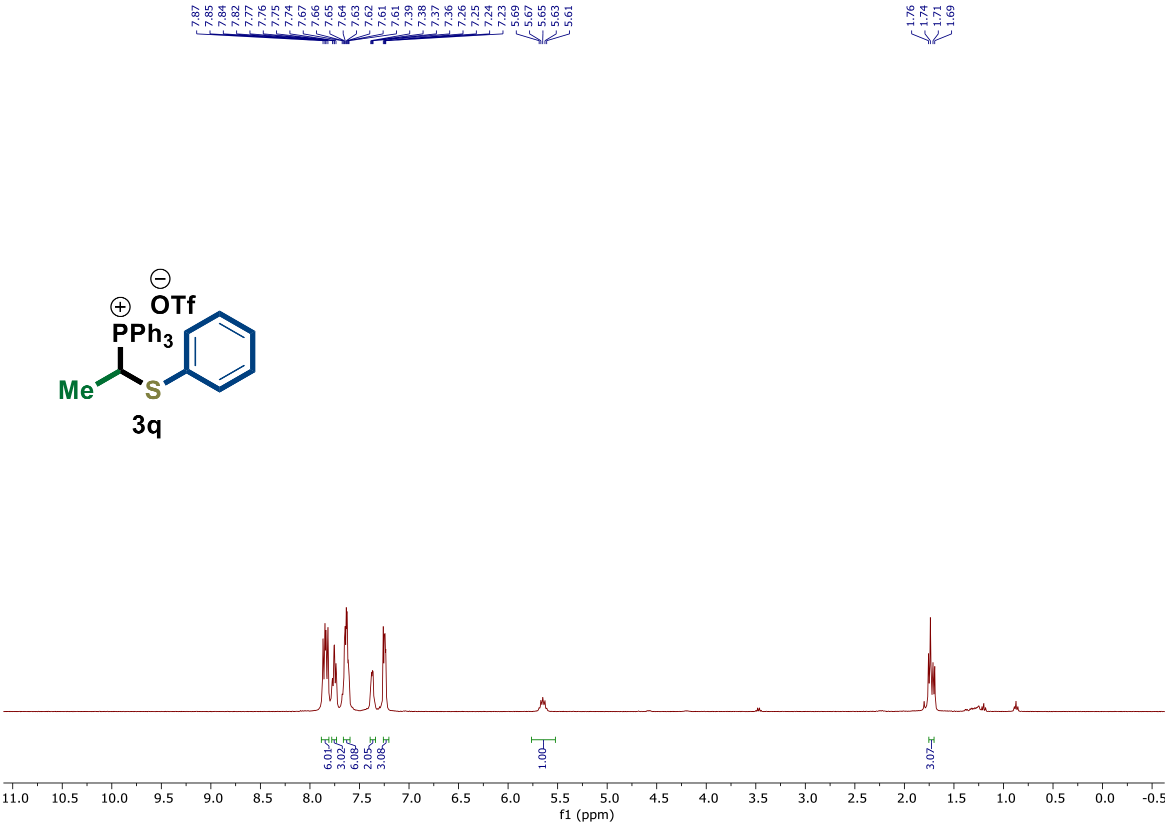

^{13}C NMR (100 MHz, $CDCl_3$) of **3q**

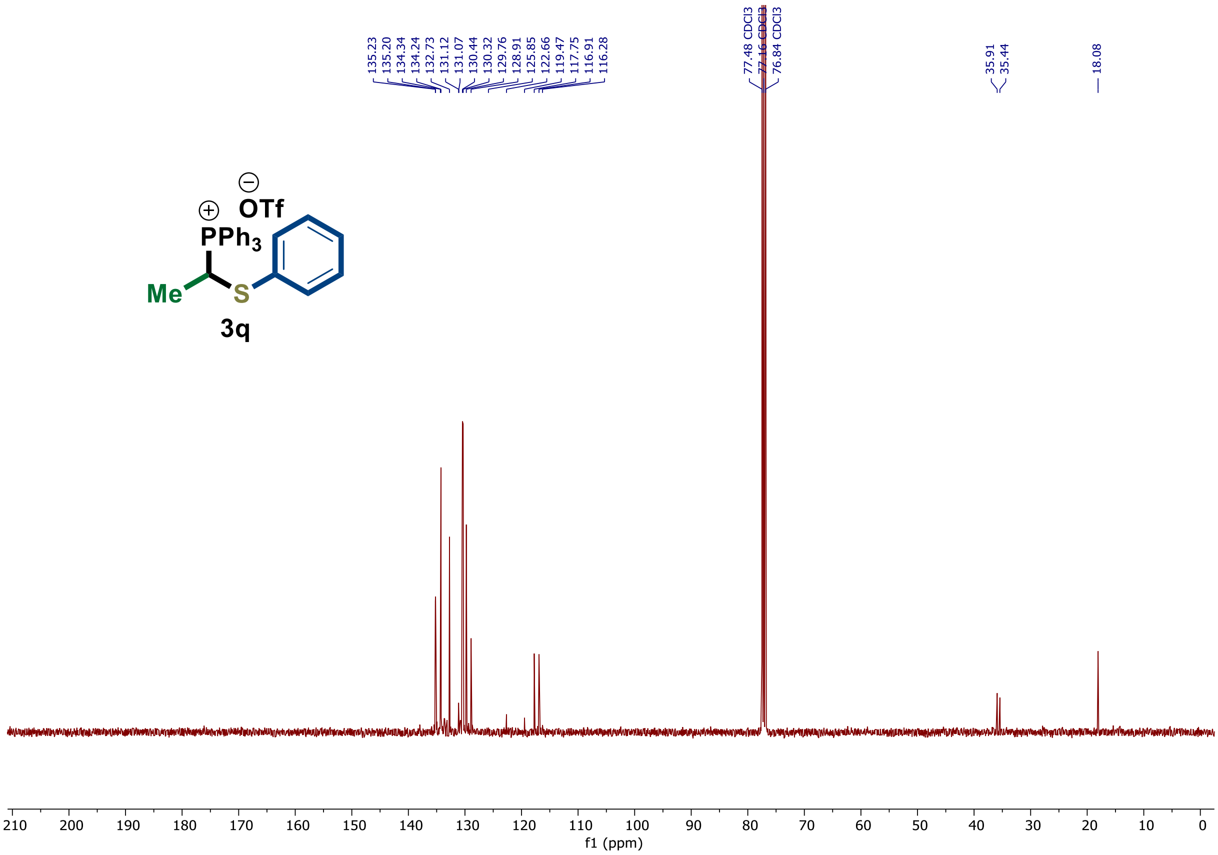

^{31}P NMR (162 MHz, $CDCl_3$) of **3q**

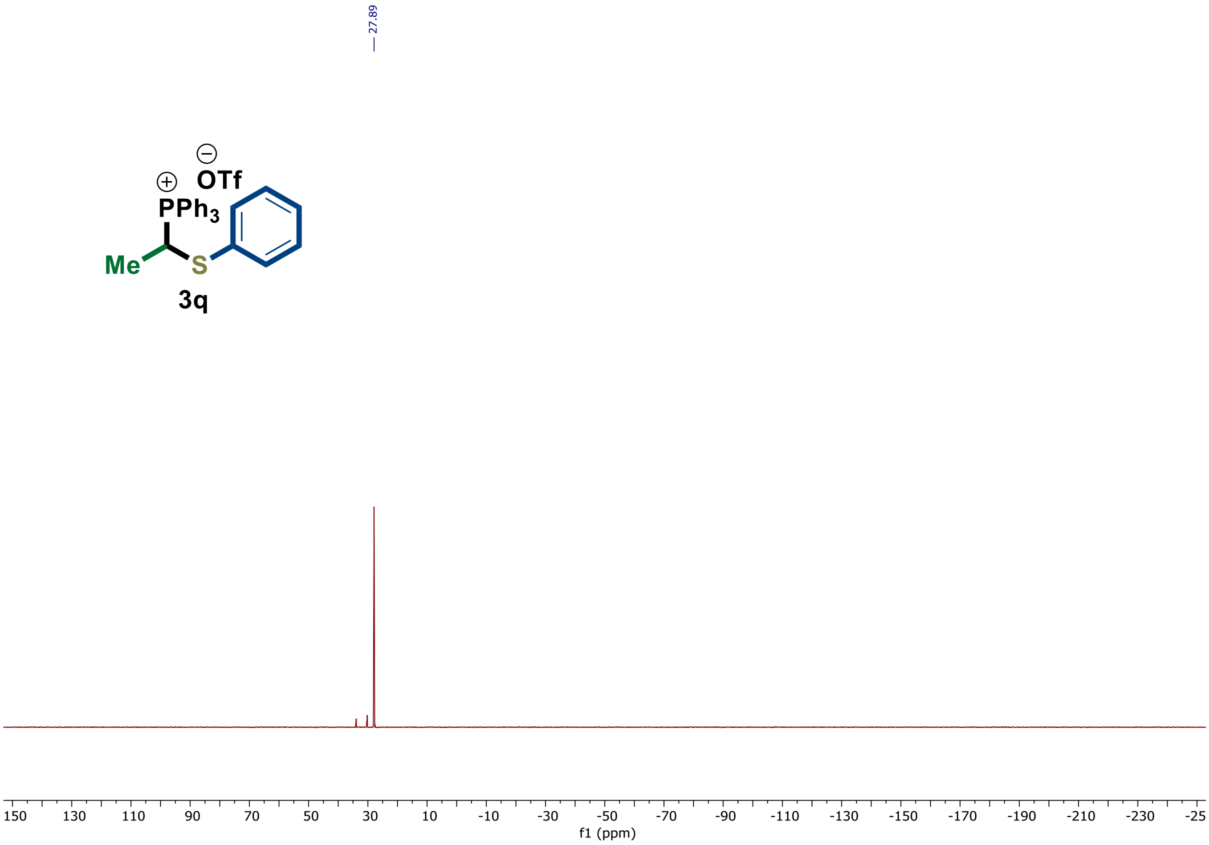

^{19}F NMR (376 MHz, $CDCl_3$) of **3q**

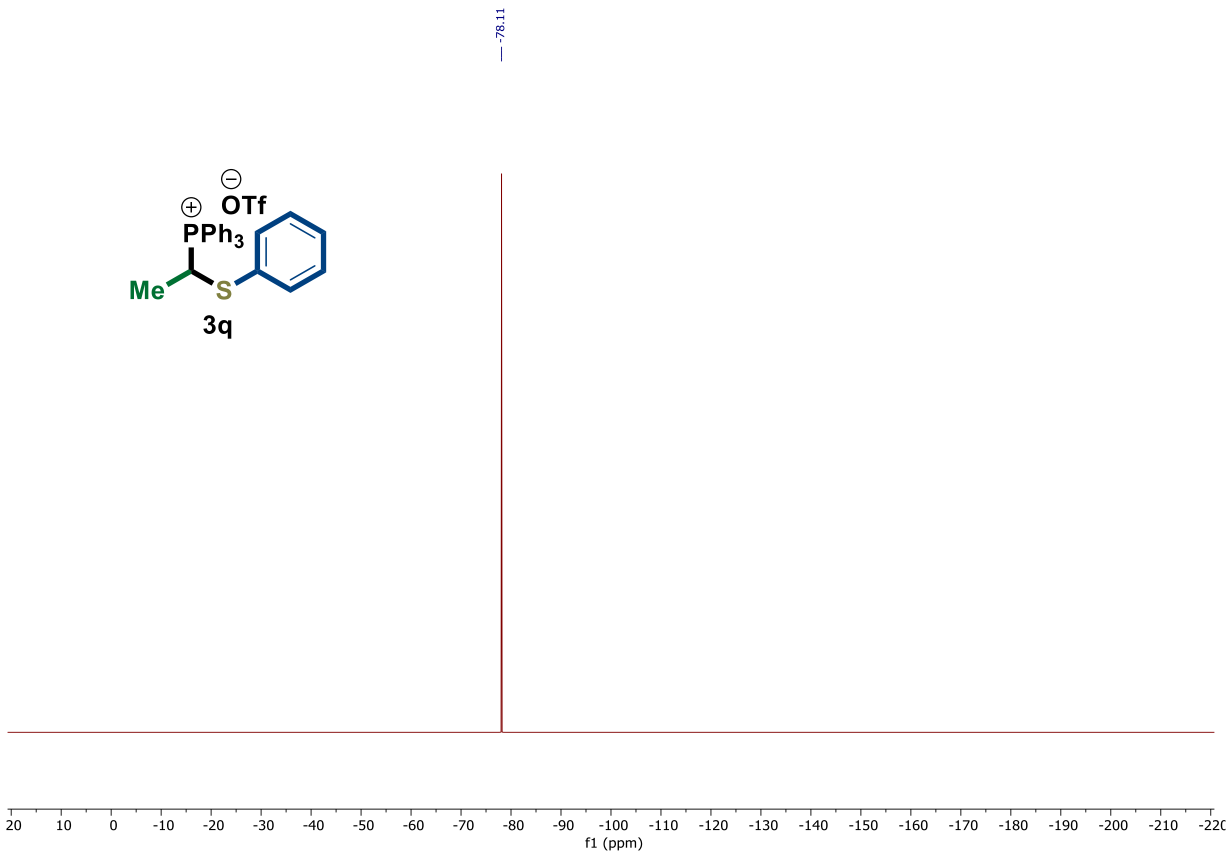

^{1}H NMR (400 MHz, $CDCl_3$) of **3r**

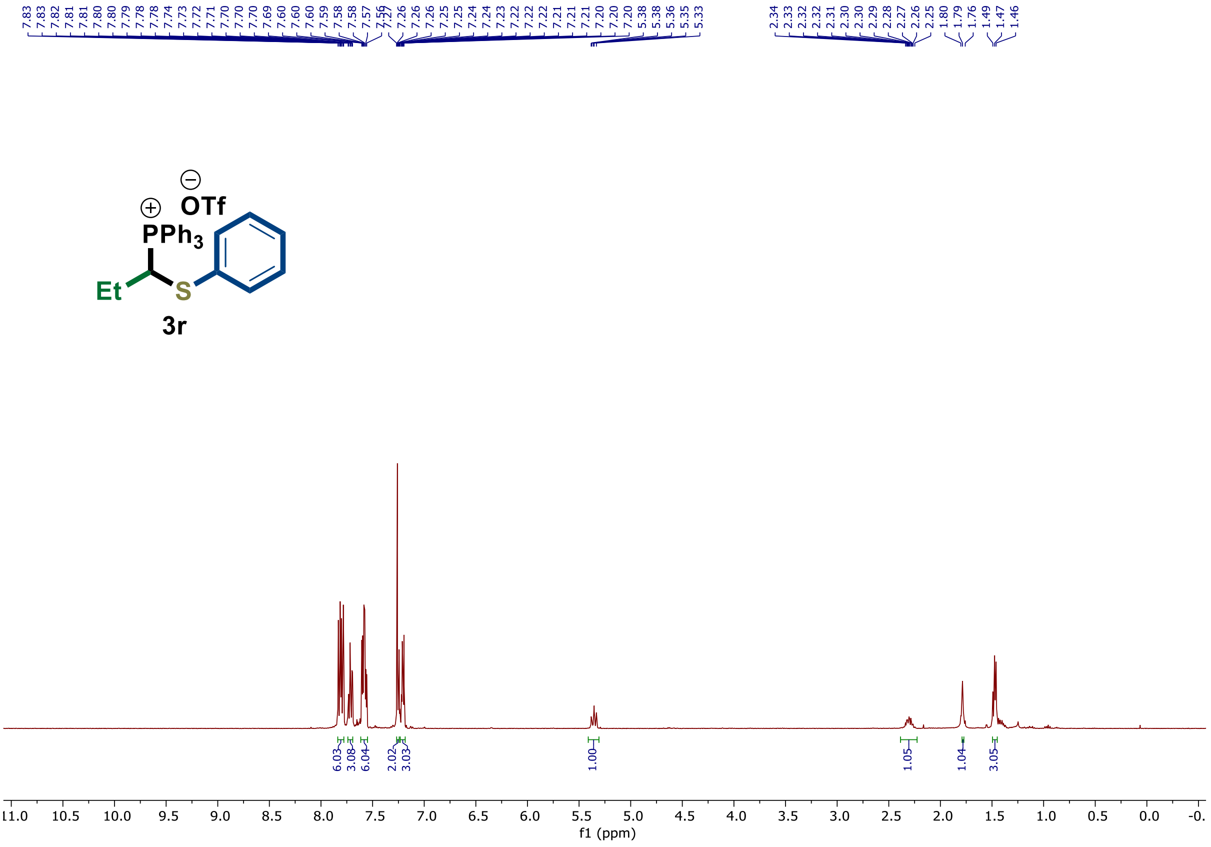

^{13}C NMR (100 MHz, $CDCl_3$) of **3r**

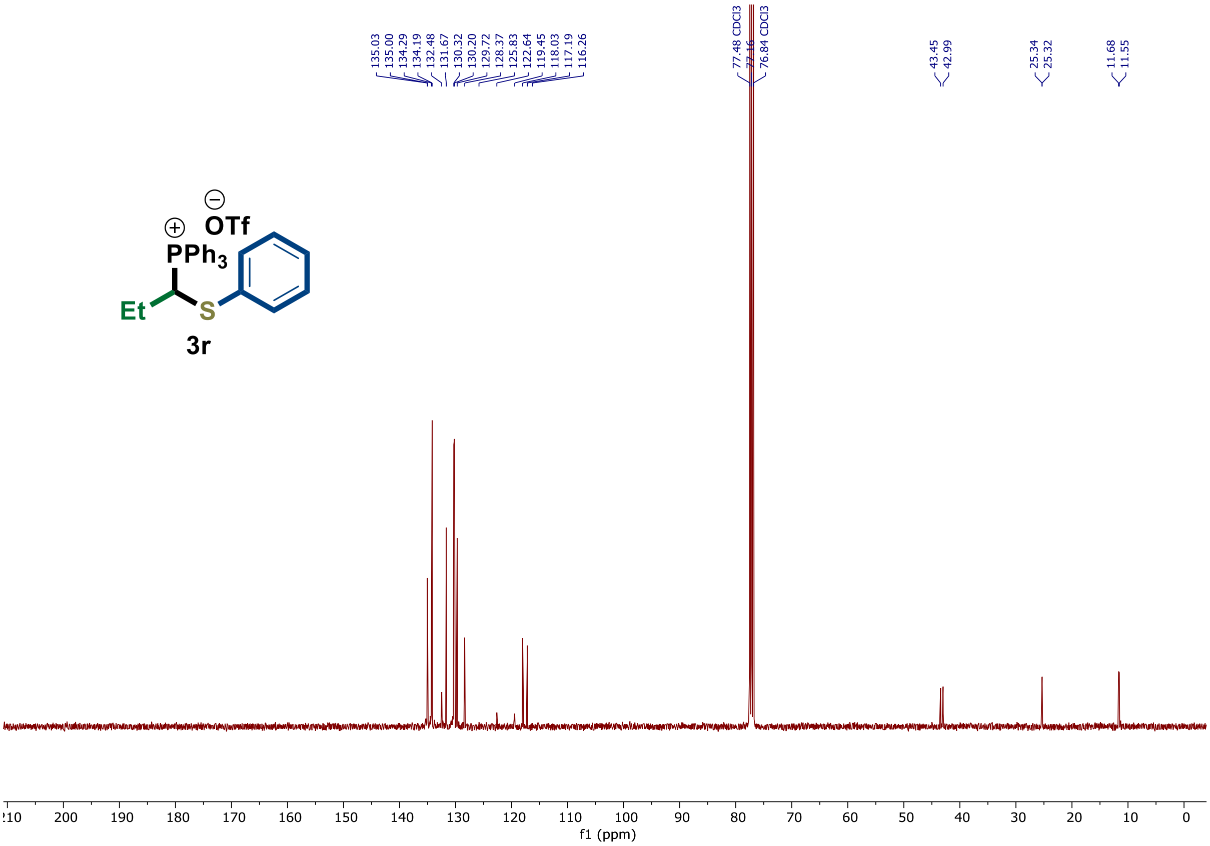

^{31}P NMR (162 MHz, $CDCl_3$) of **3r**

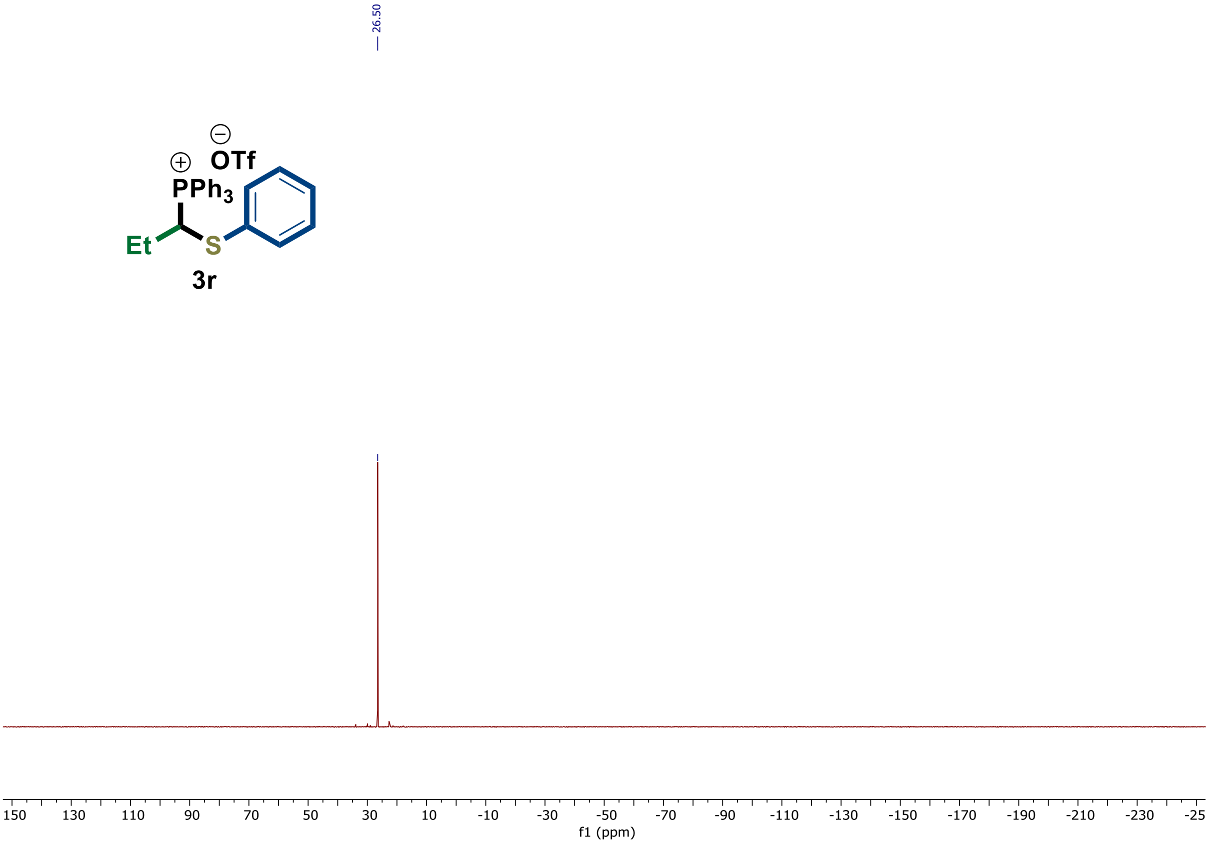

^{19}F NMR (376 MHz, $CDCl_3$) of **3r**

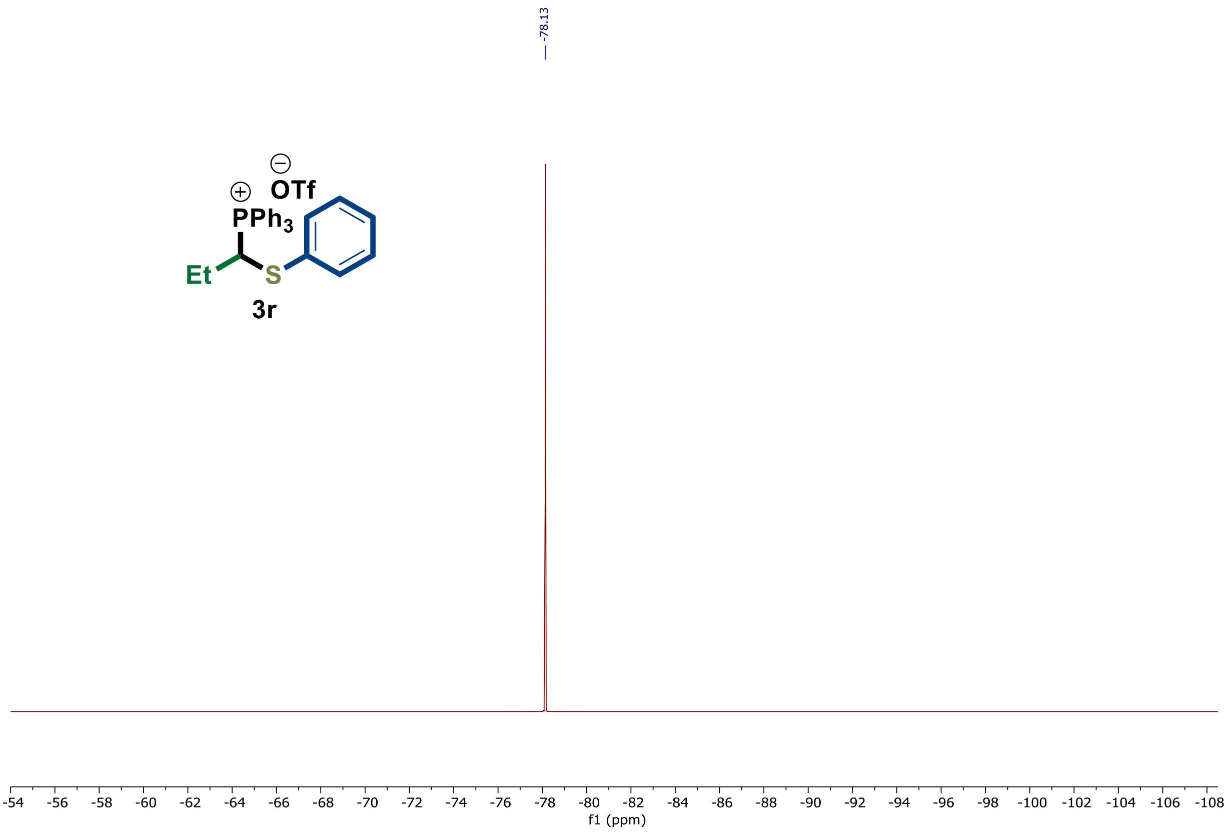

^{1}H NMR (400 MHz, $CDCl_3$) of **3s**

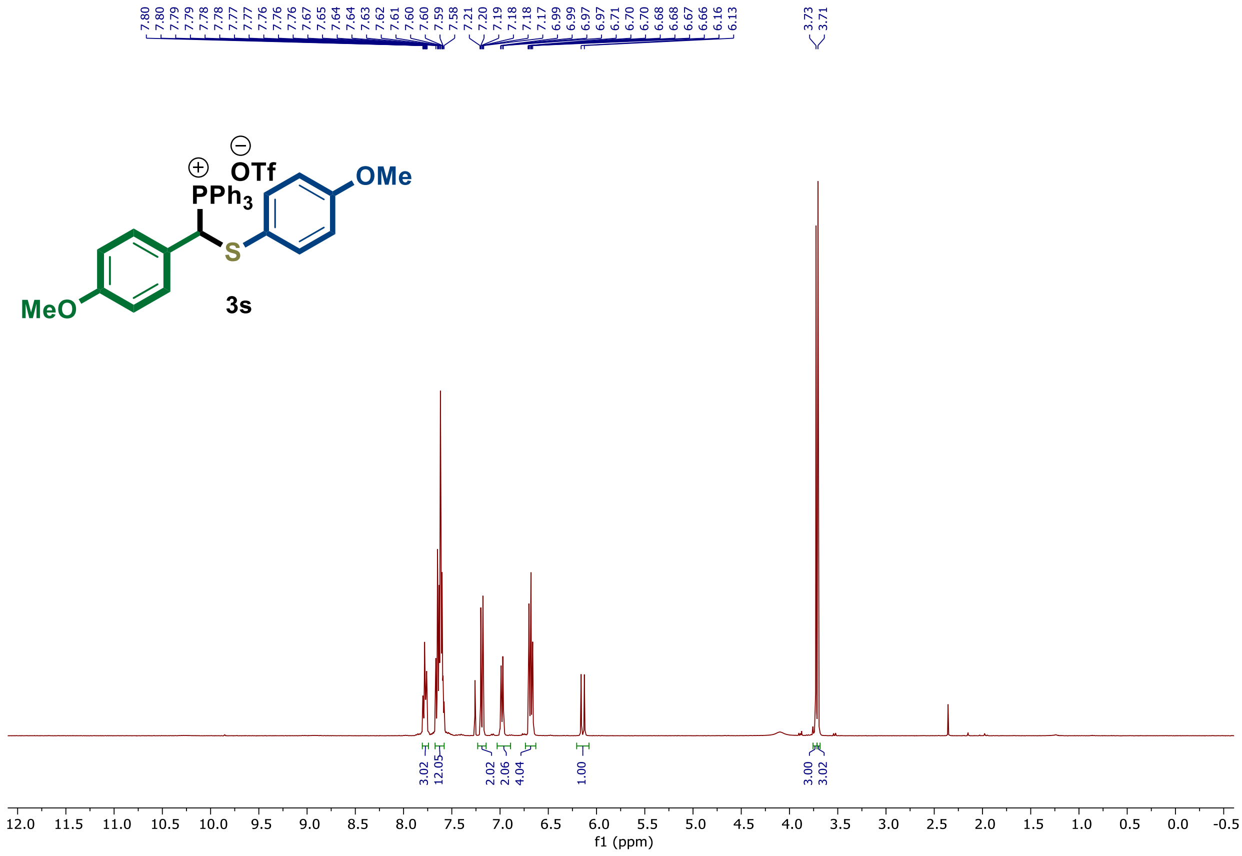

^{13}C NMR (100 MHz, CDCl$_3$) of **3s**

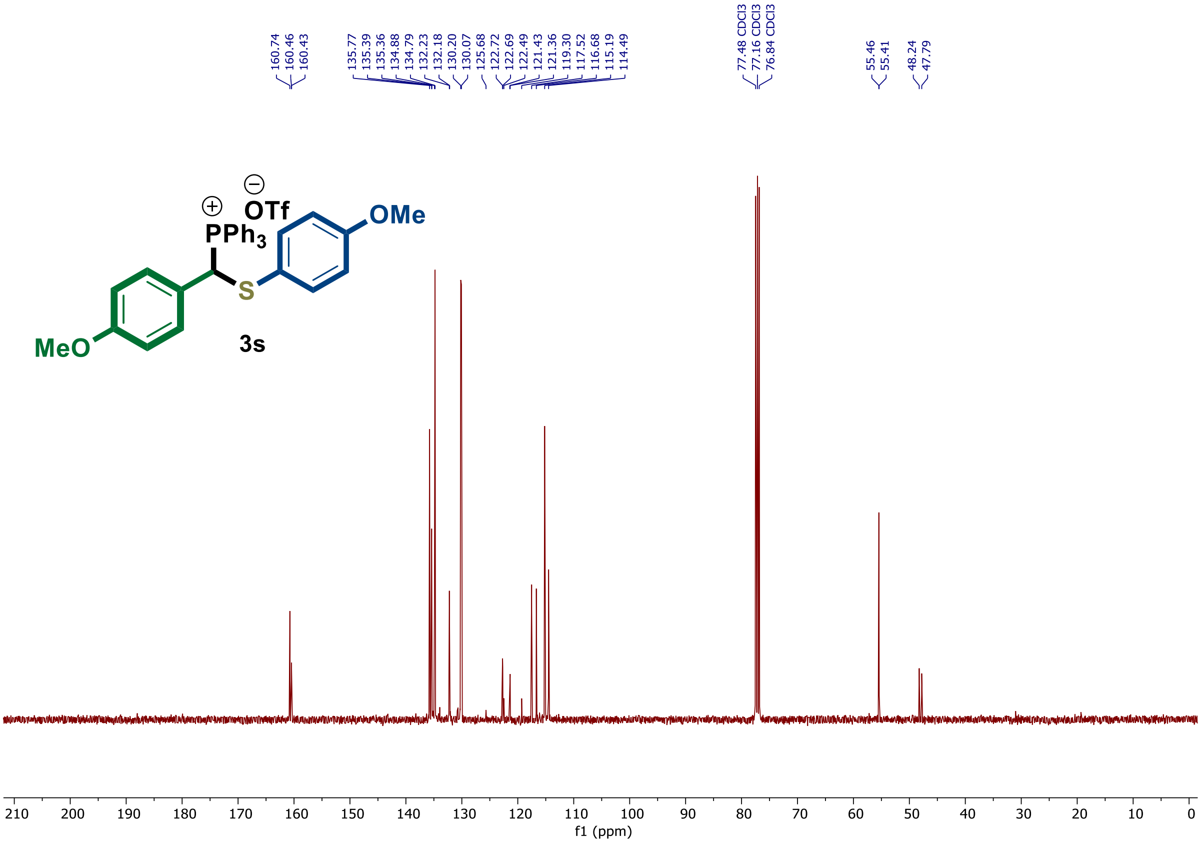

^{31}P NMR (162 MHz, CDCl$_3$) of **3s**

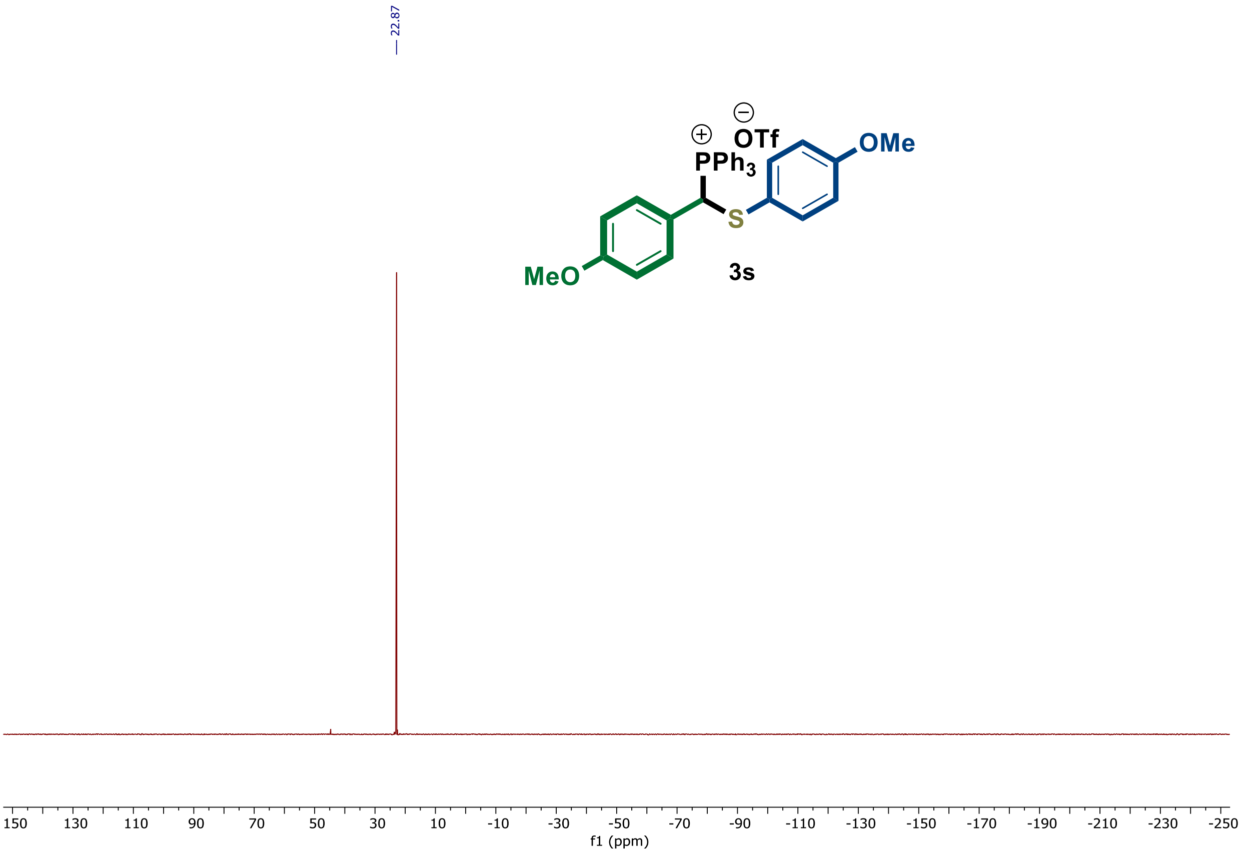

^{19}F NMR (376 MHz, $CDCl_3$) of **3s**

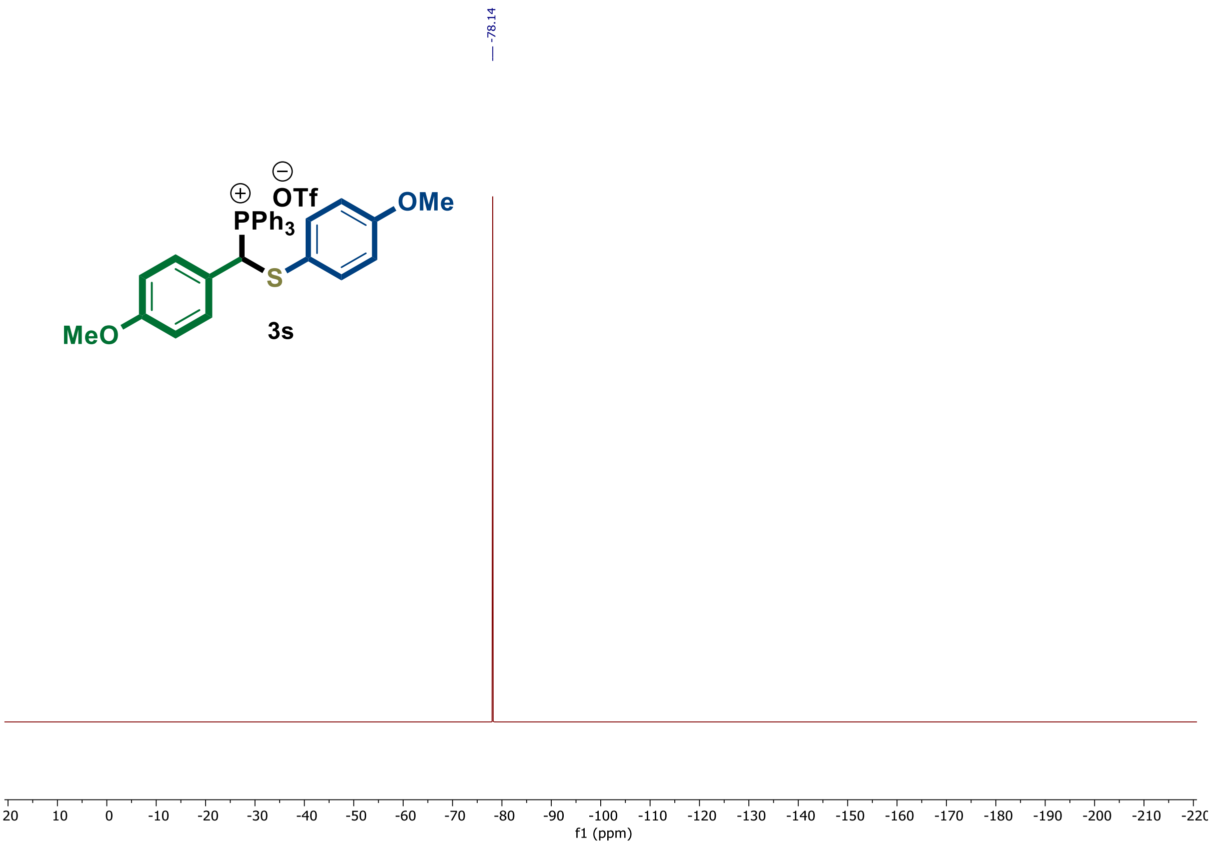

^{1}H NMR (400 MHz, $CDCl_3$) of **3t**

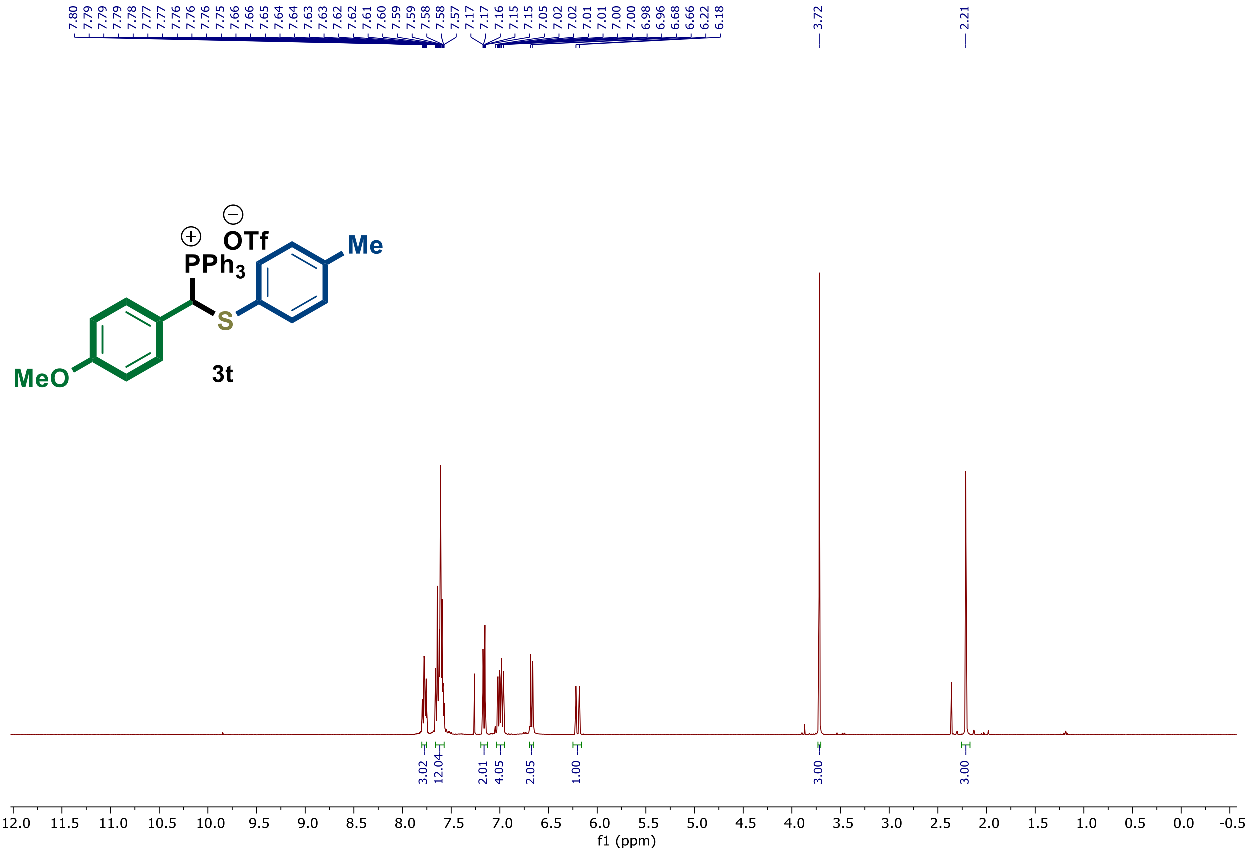

^{13}C NMR (100 MHz, $CDCl_3$) of **3t**

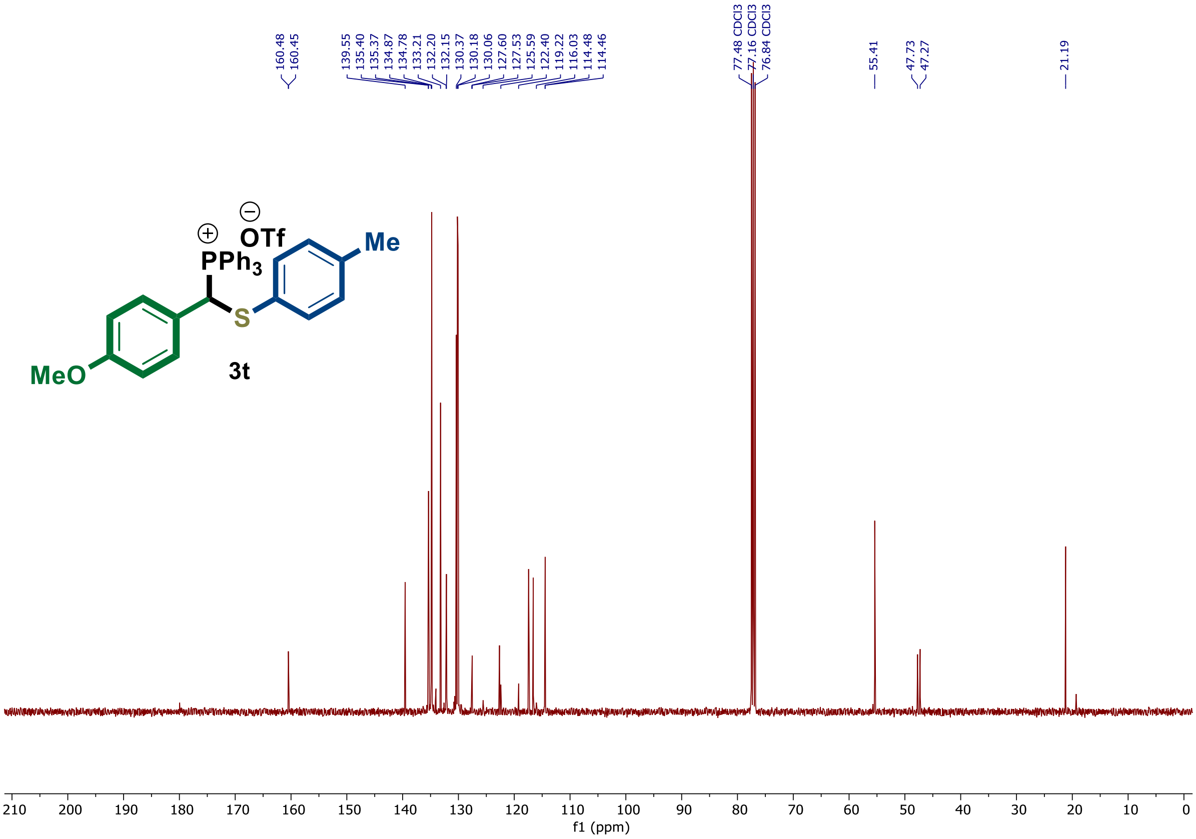

^{31}P NMR (162 MHz, $CDCl_3$) of **3t**

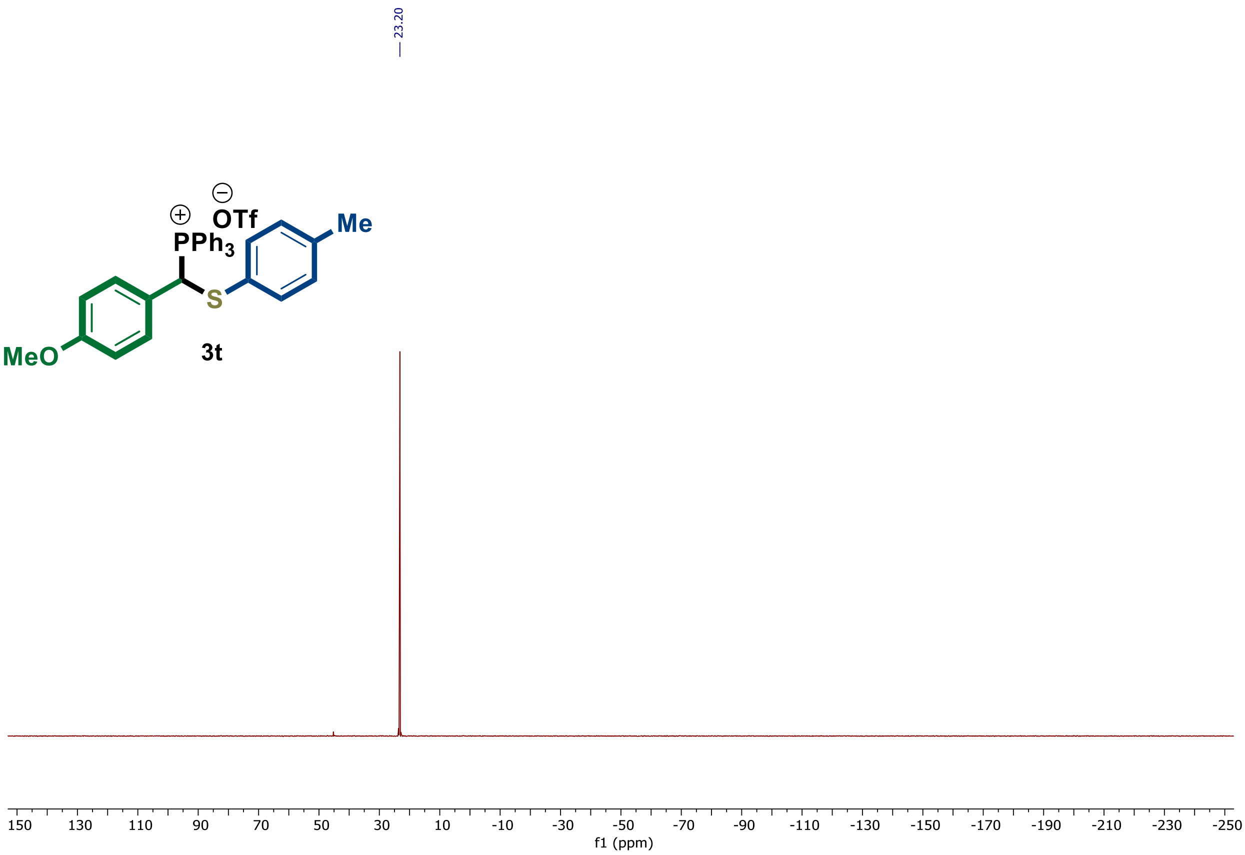

^{19}F NMR (376 MHz, $CDCl_3$) of **3t**

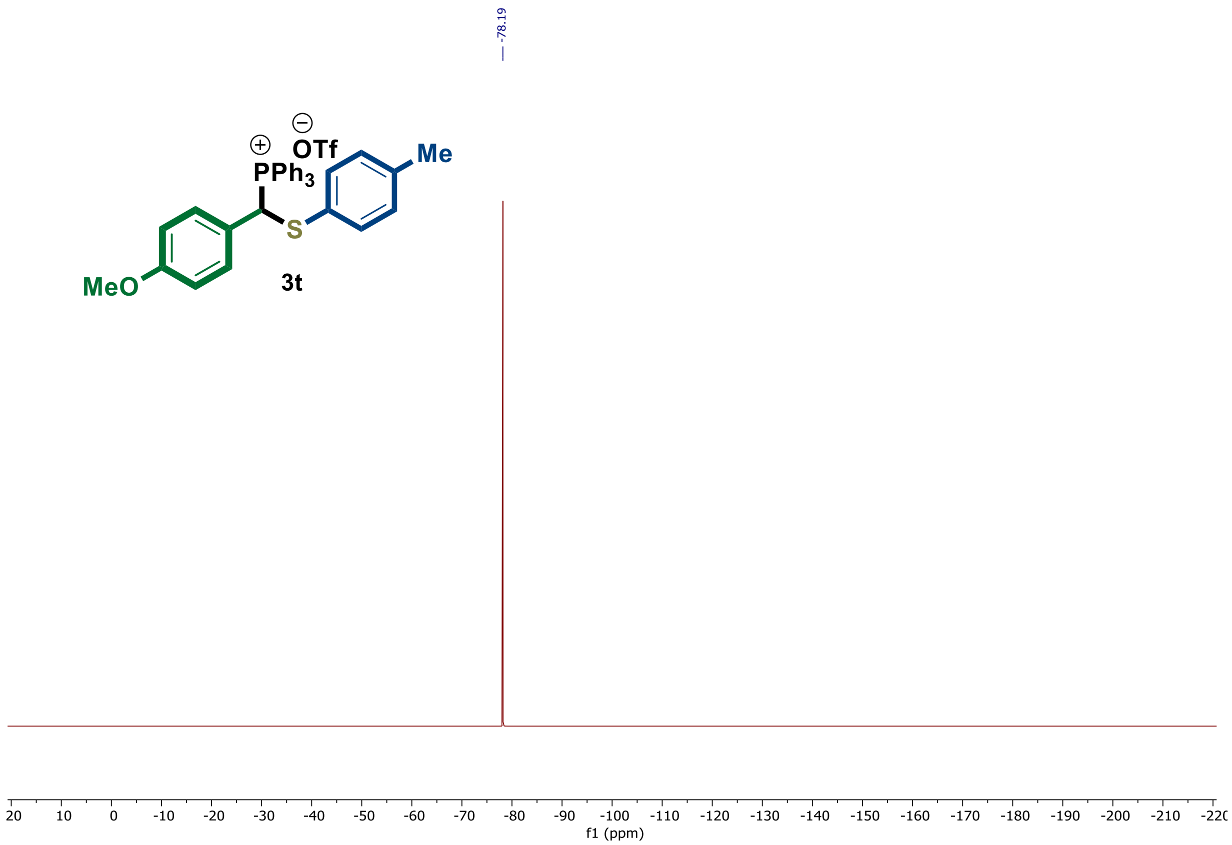

^{1}H NMR (400 MHz, $CDCl_3$) of **3u**

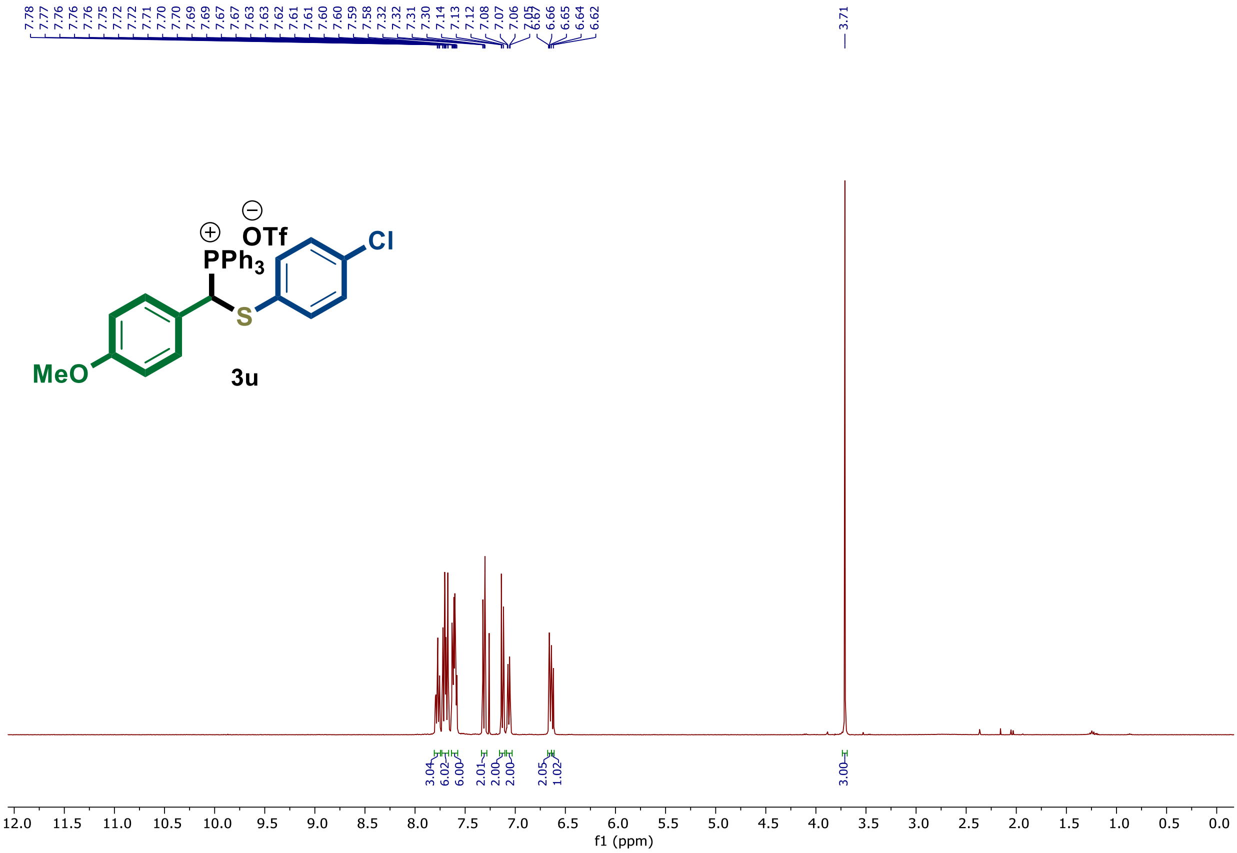

^{13}C NMR (100 MHz, CDCl$_3$) of **3u**

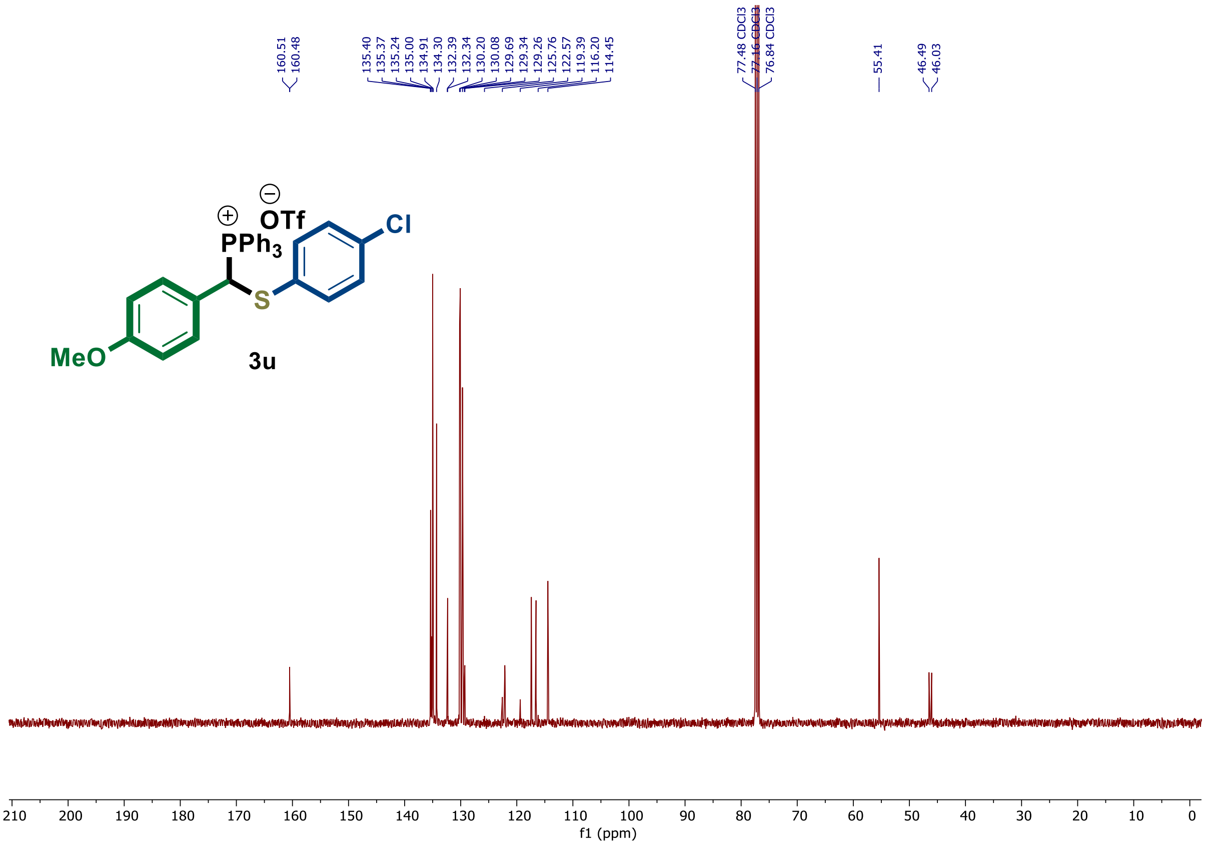

^{31}P NMR (162 MHz, CDCl$_3$) of **3u**

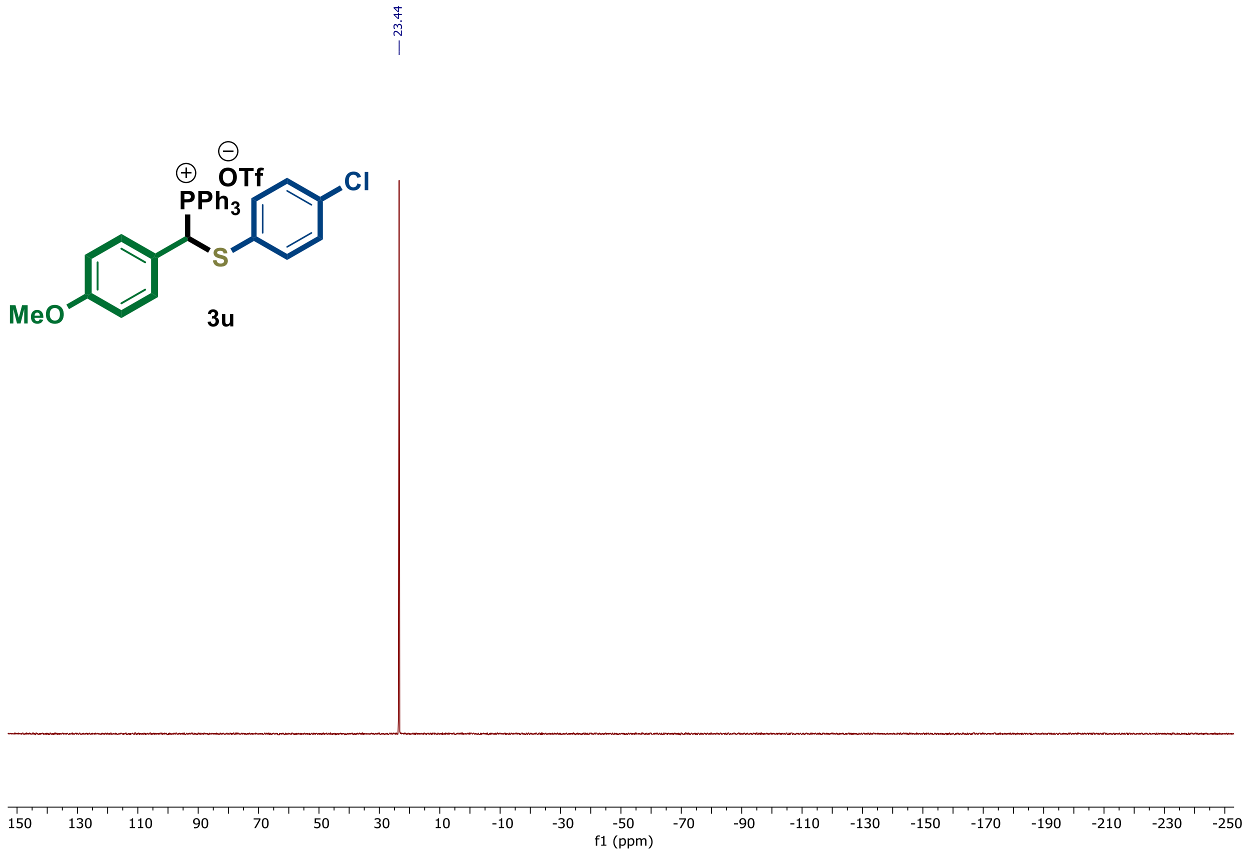

^{19}F NMR (376 MHz, CDCl$_3$) of **3u**

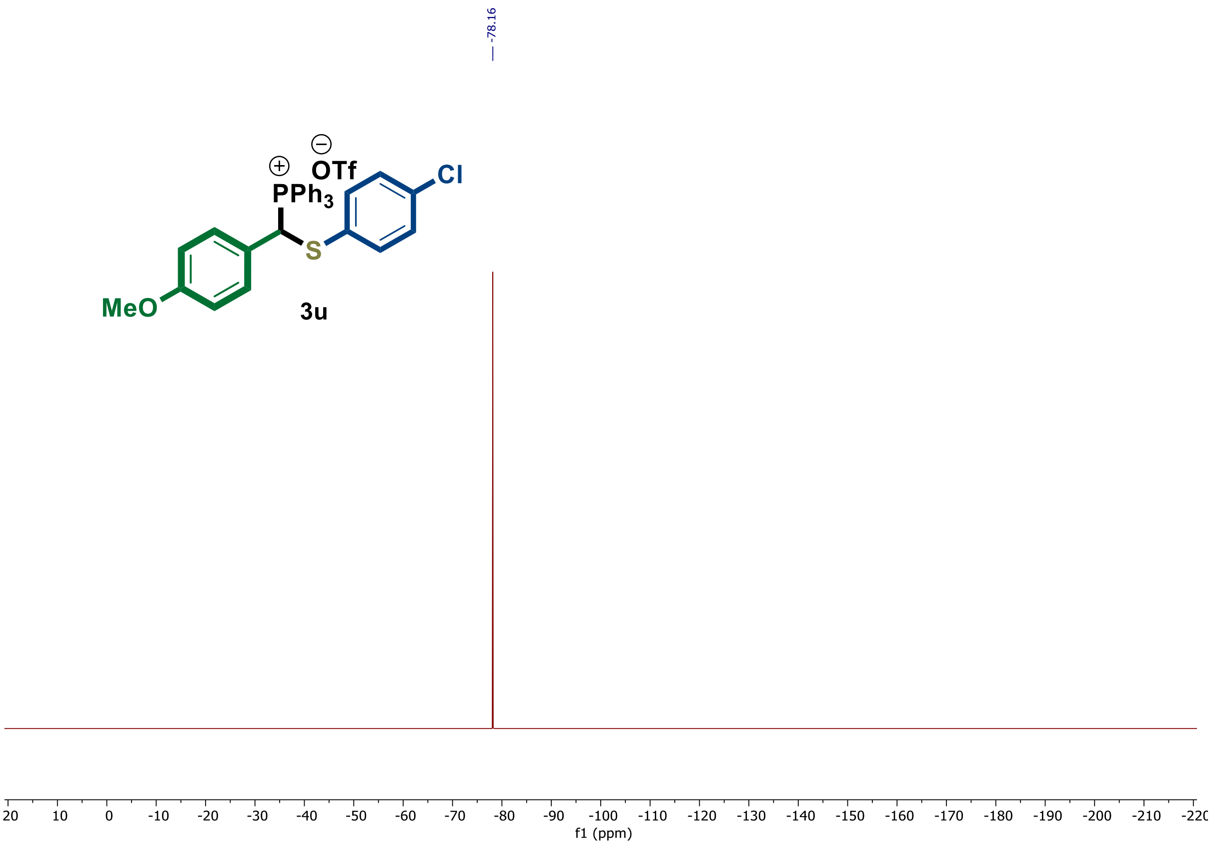

^{1}H NMR (400 MHz, CDCl$_3$) of **3v**

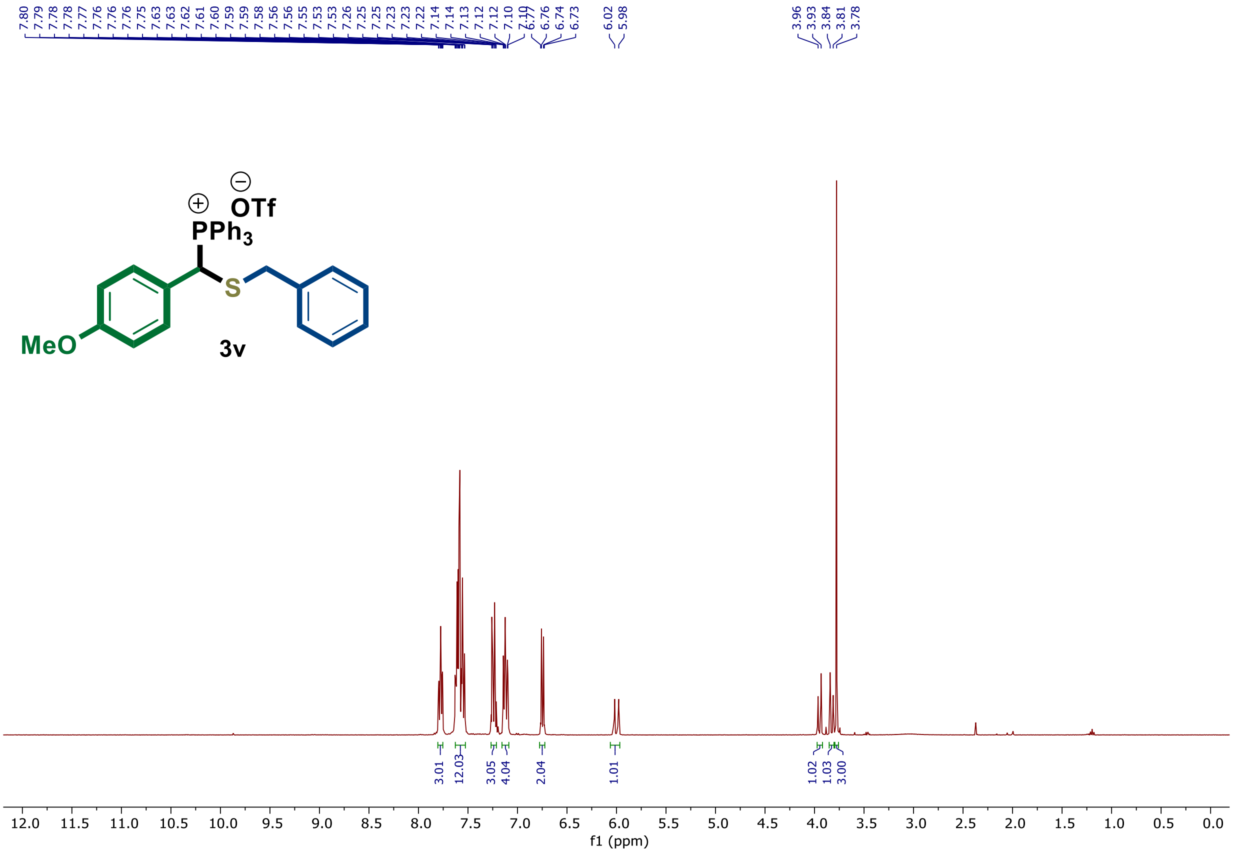

^{13}C NMR (100 MHz, CDCl$_3$) of **3v**

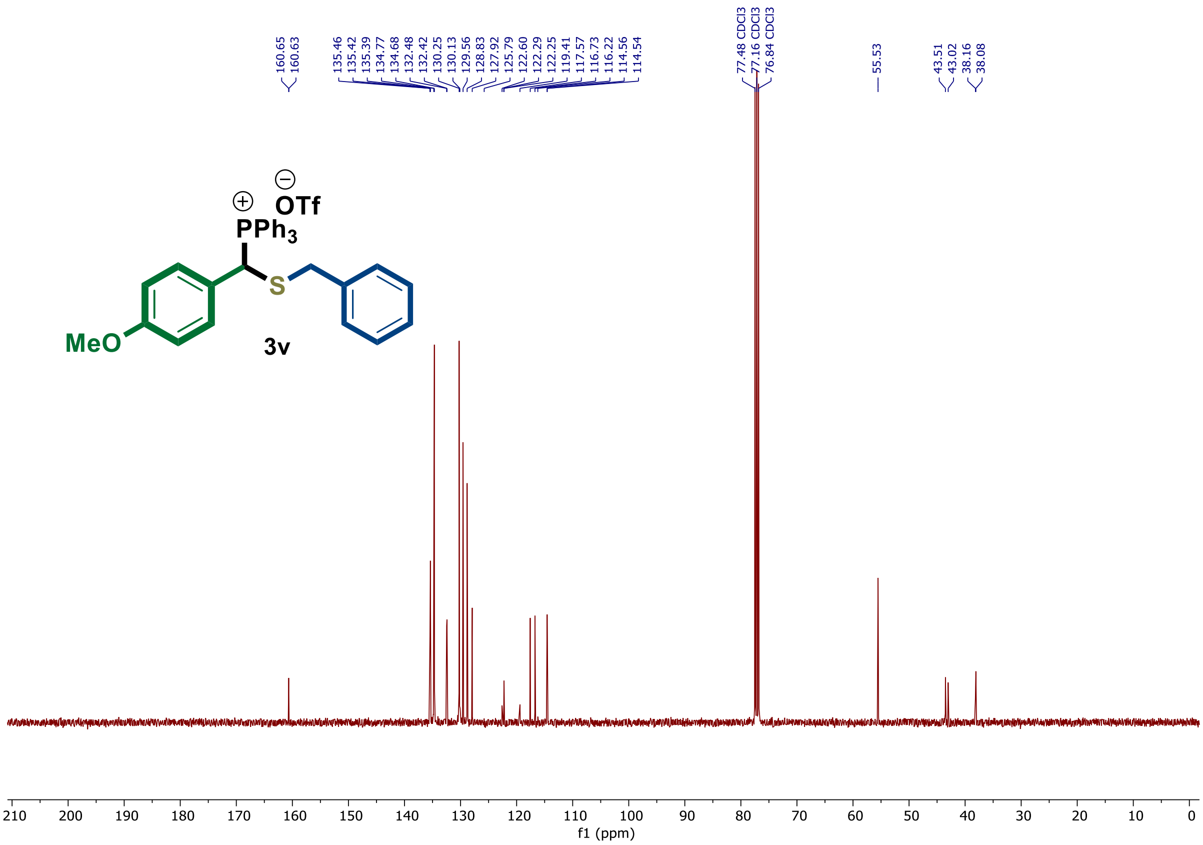

^{31}P NMR (162 MHz, CDCl$_3$) of **3v**

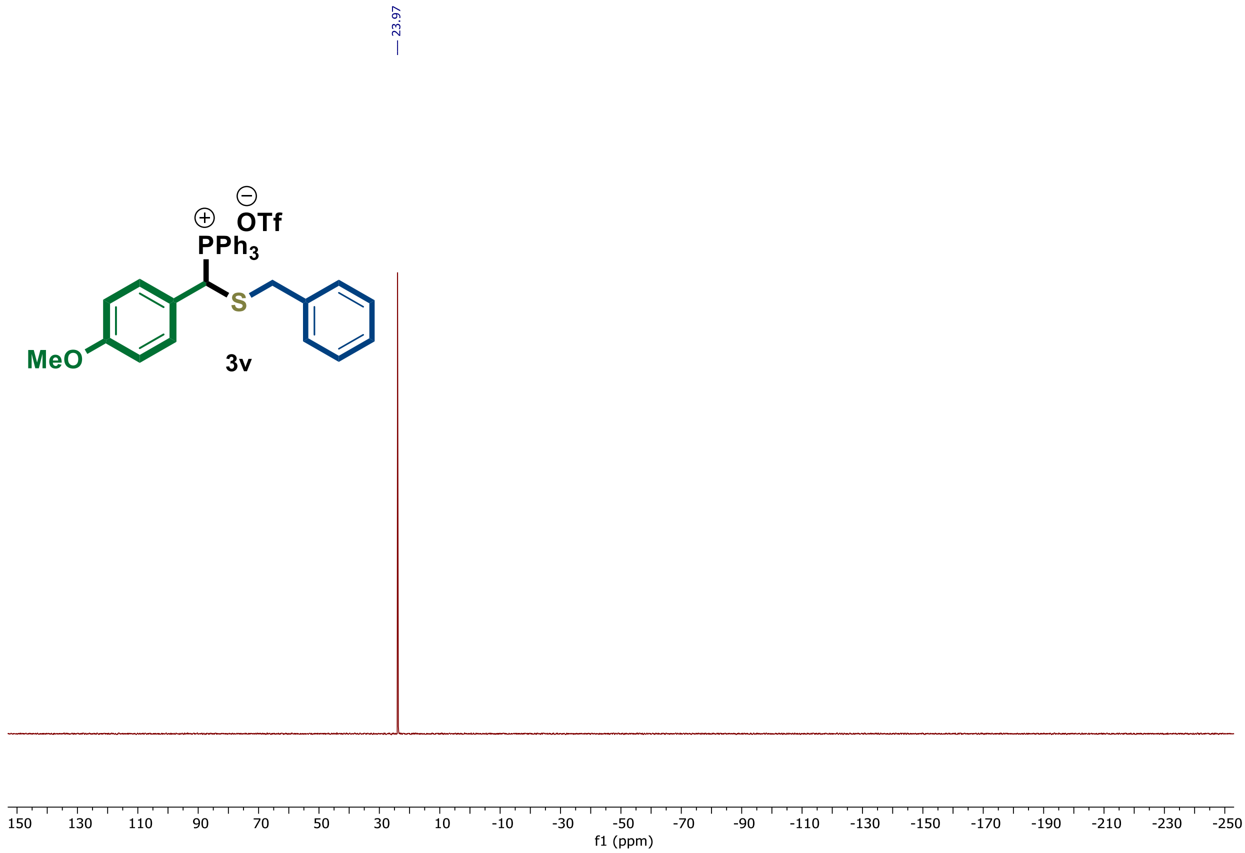

^{19}F NMR (376 MHz, CDCl$_3$) of **3v**

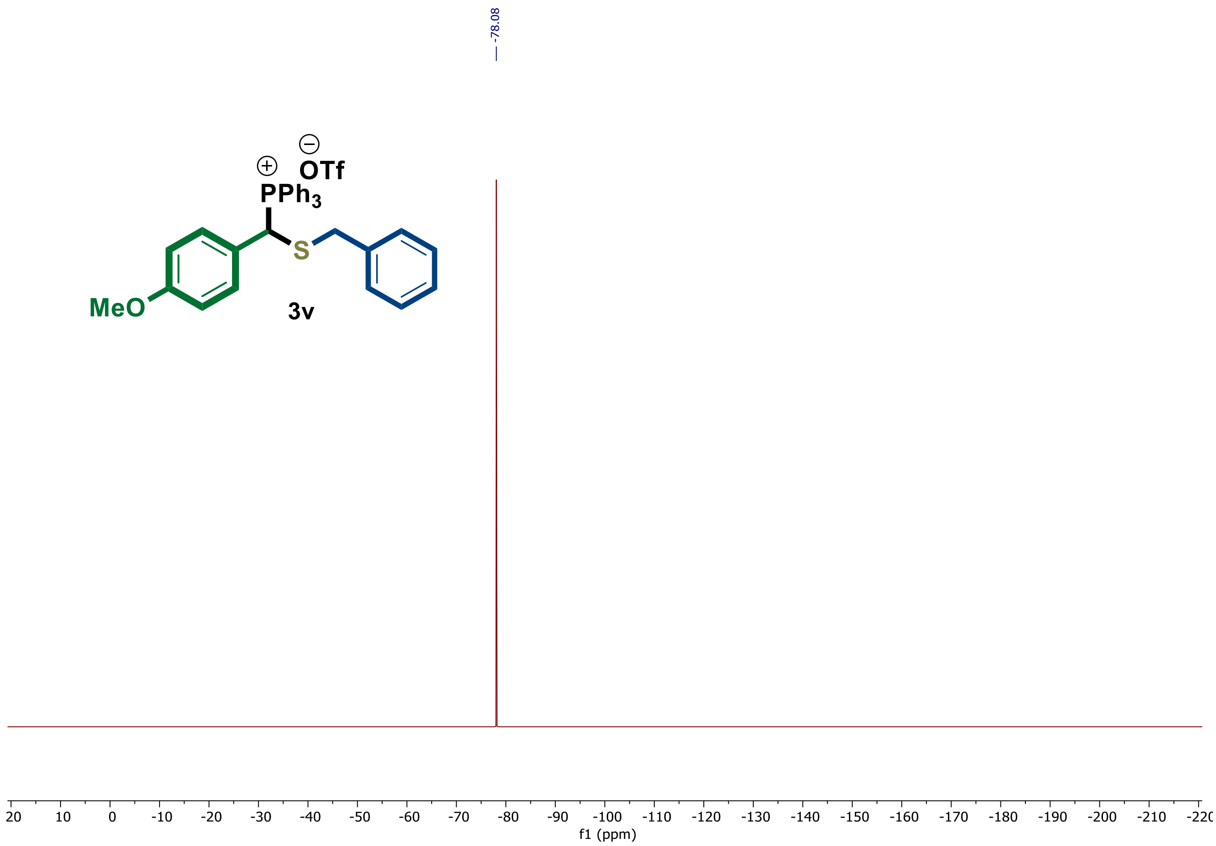

^{1}H NMR (400 MHz, CDCl$_3$) of **3w**

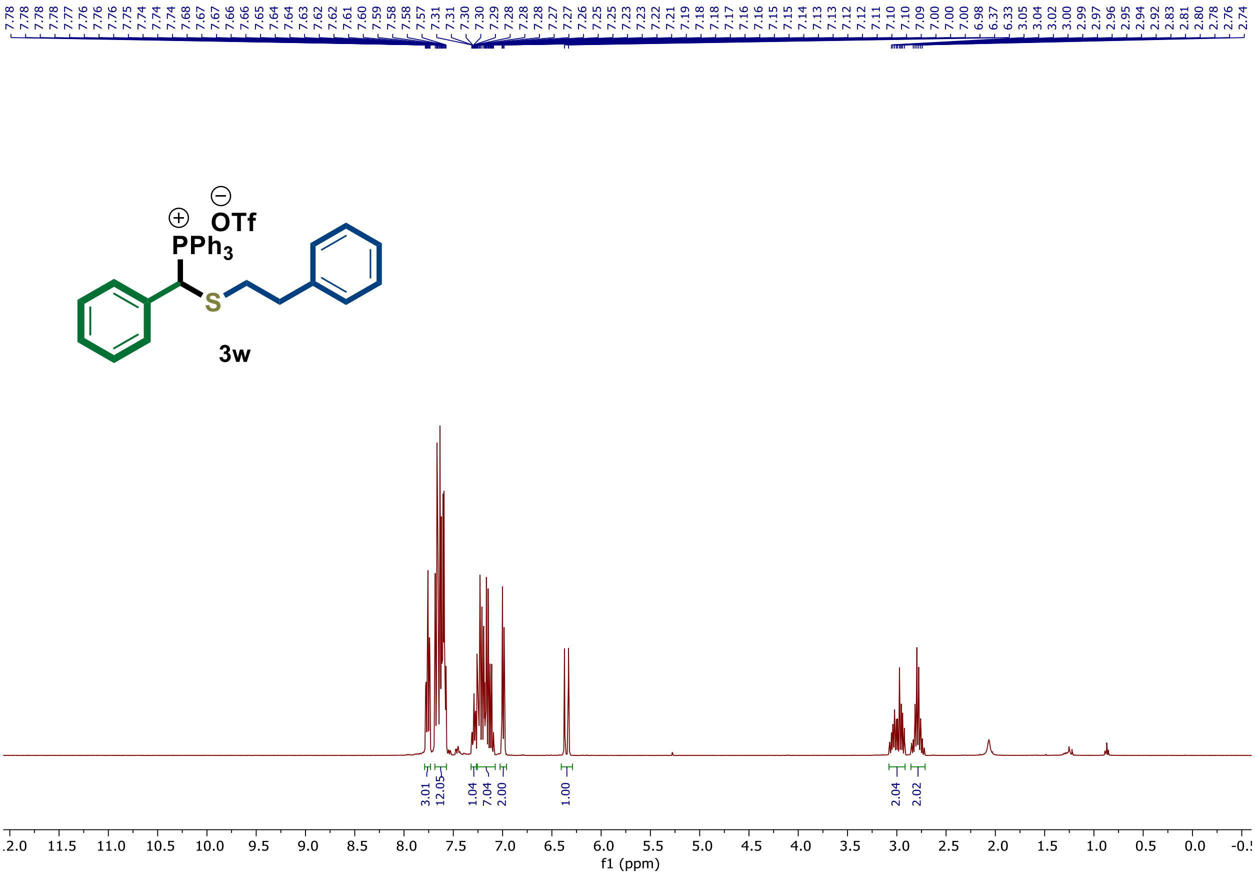

^{13}C NMR (100 MHz, $CDCl_3$) of **3w**

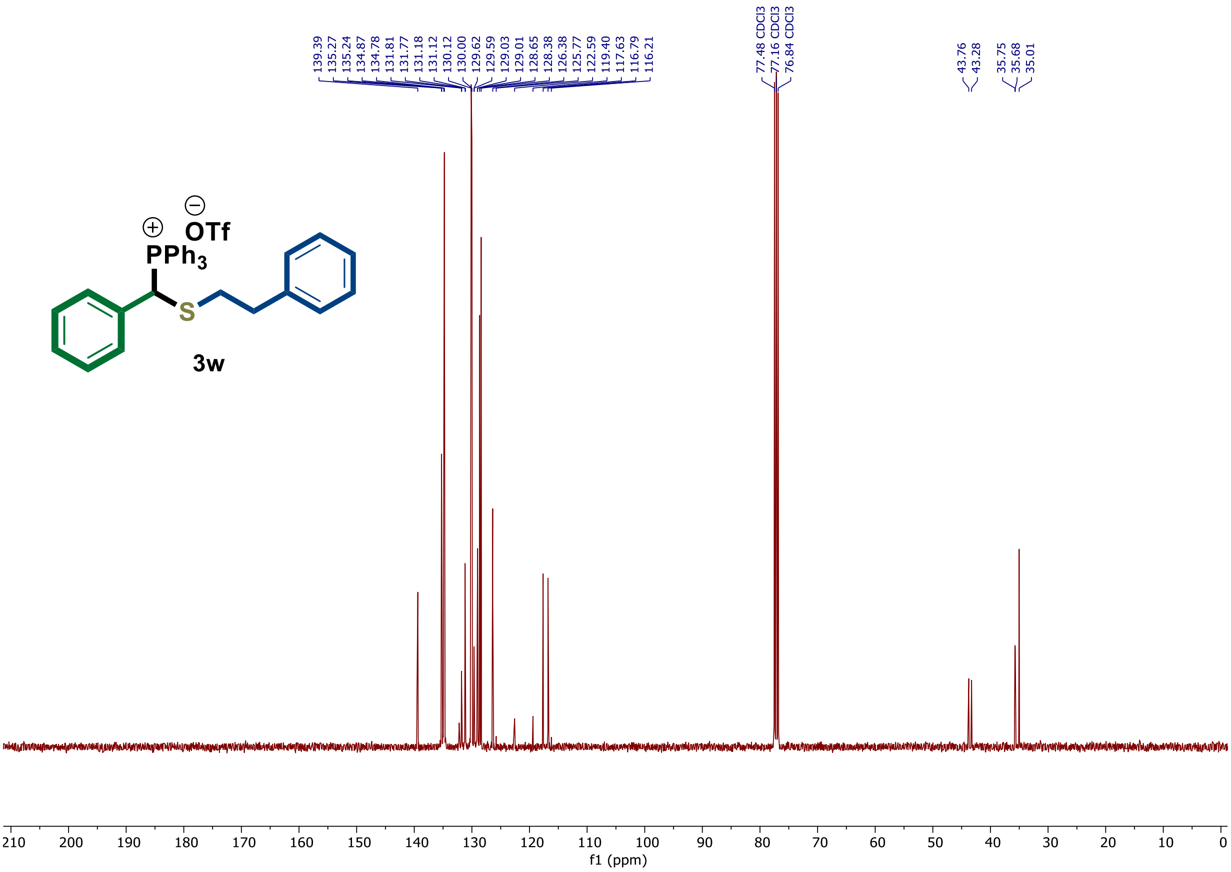

^{31}P NMR (162 MHz, $CDCl_3$) of **3w**

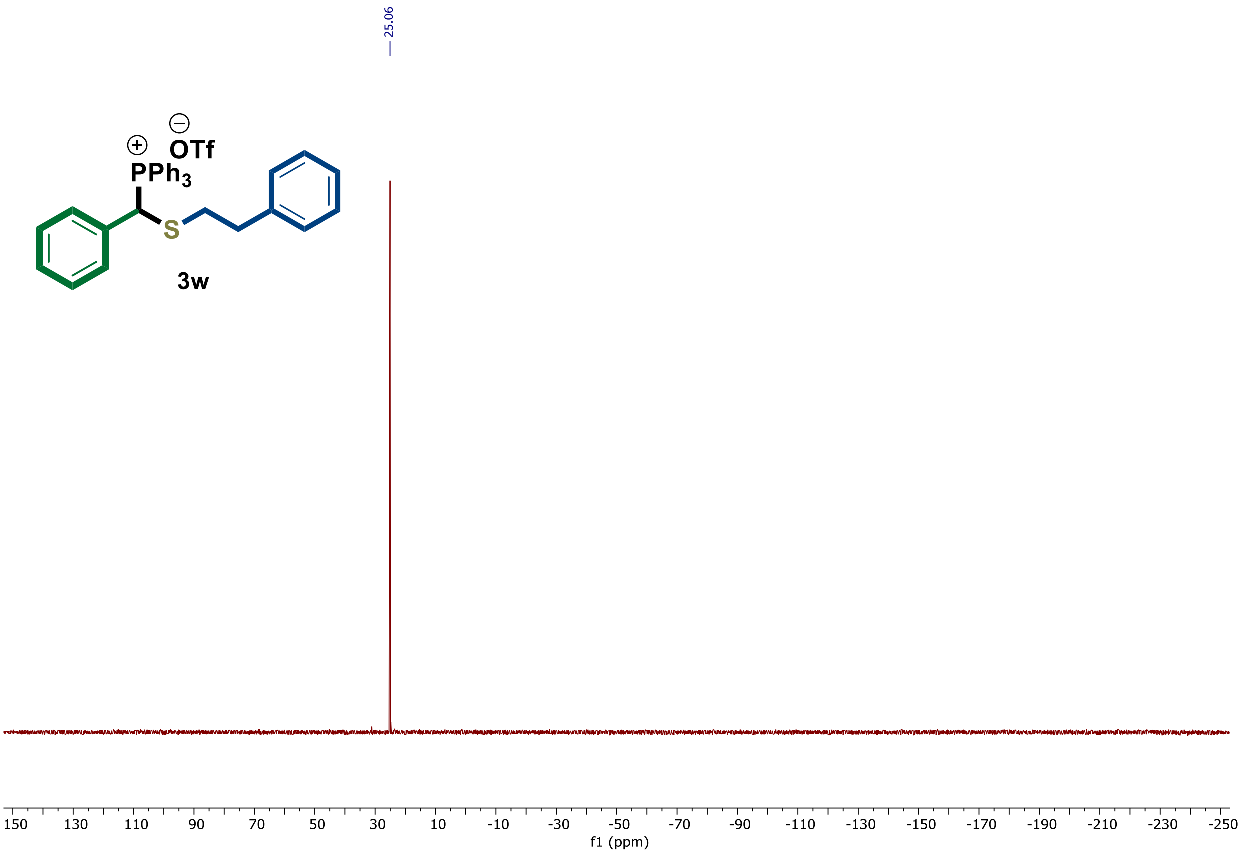

^{19}F NMR (376 MHz, $CDCl_3$) of **3w**

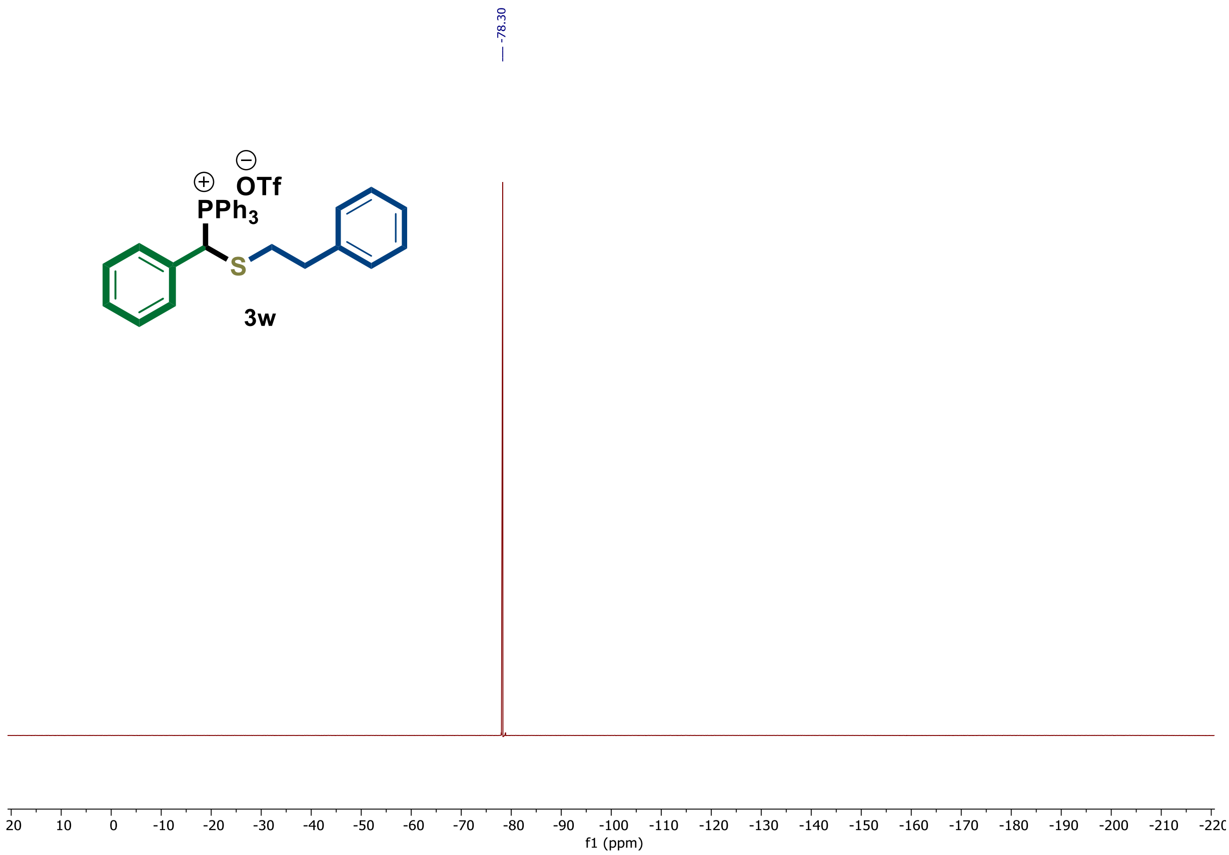

^{1}H NMR (400 MHz, $CDCl_3$) of **3x**

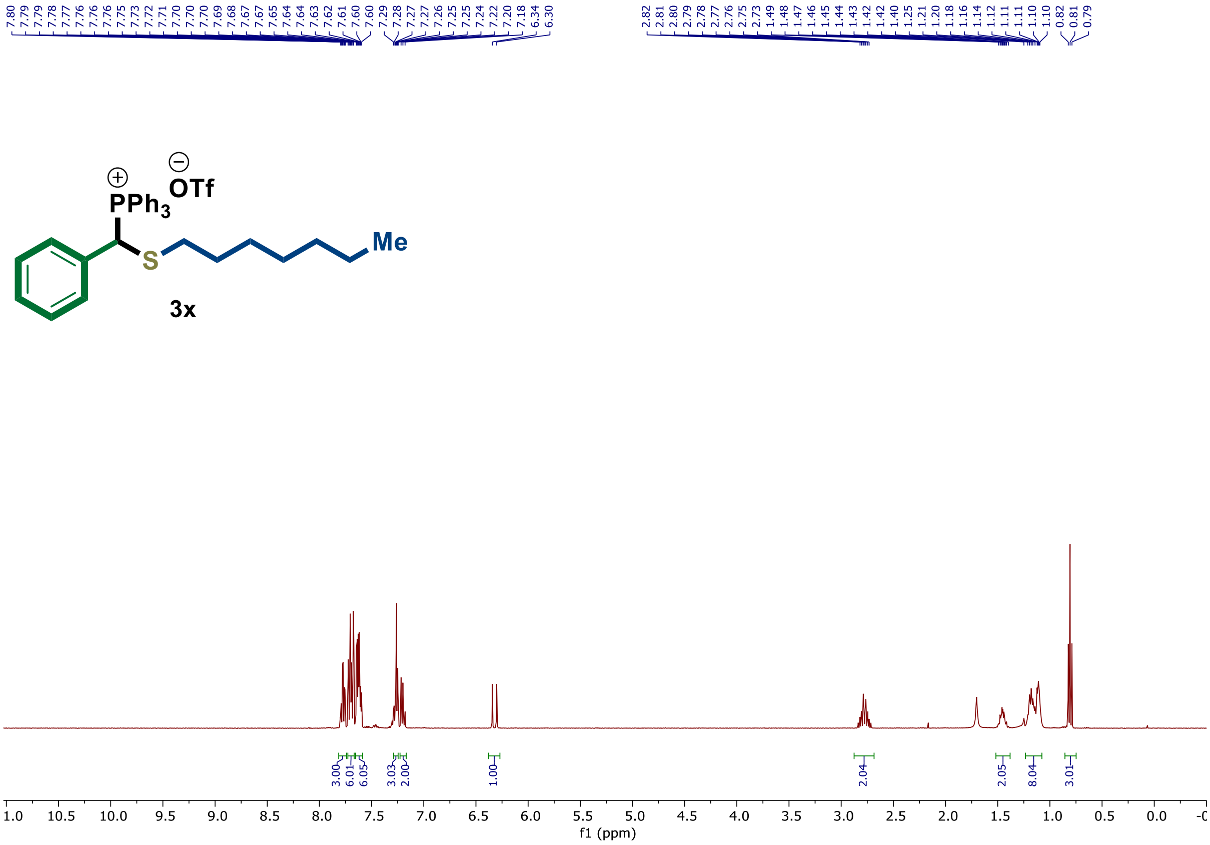

^{13}C NMR (100 MHz, CDCl$_3$) of **3x**

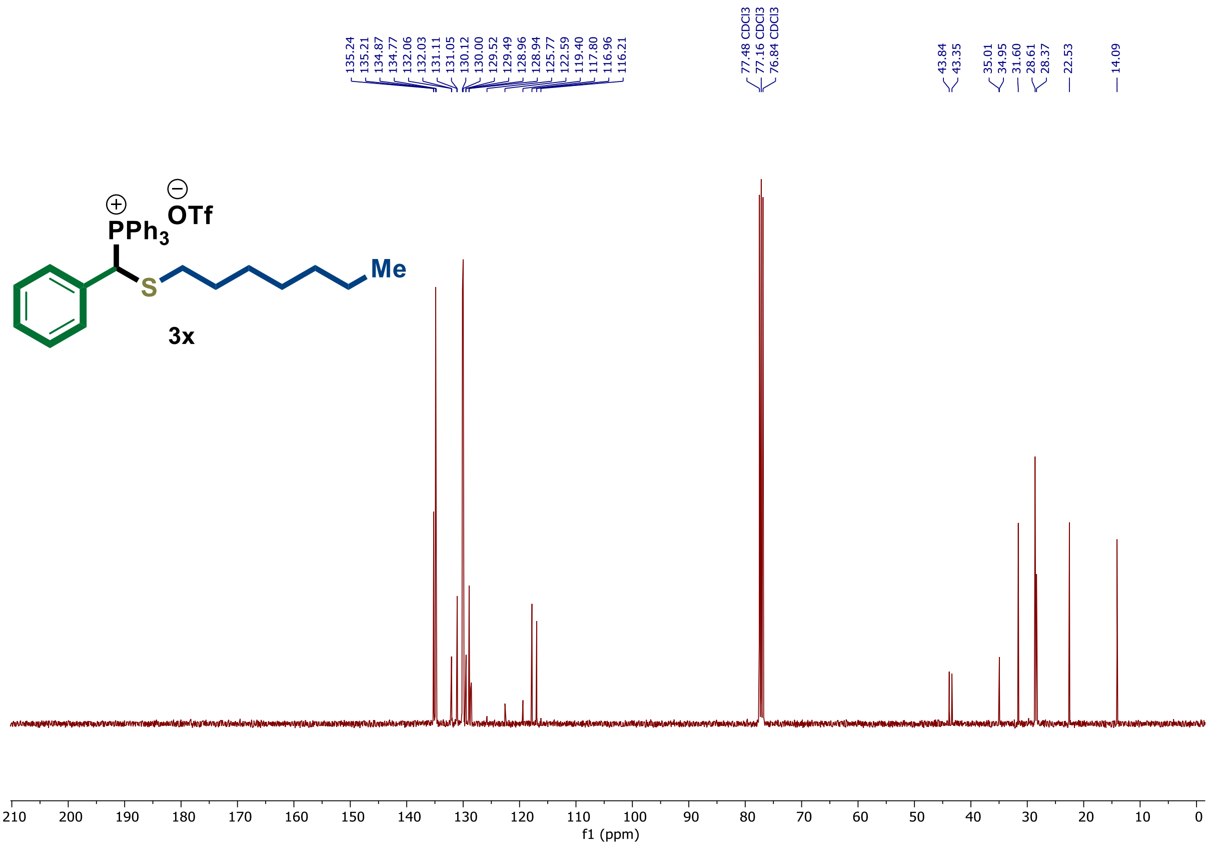

^{31}P NMR (162 MHz, CDCl$_3$) of **3x**

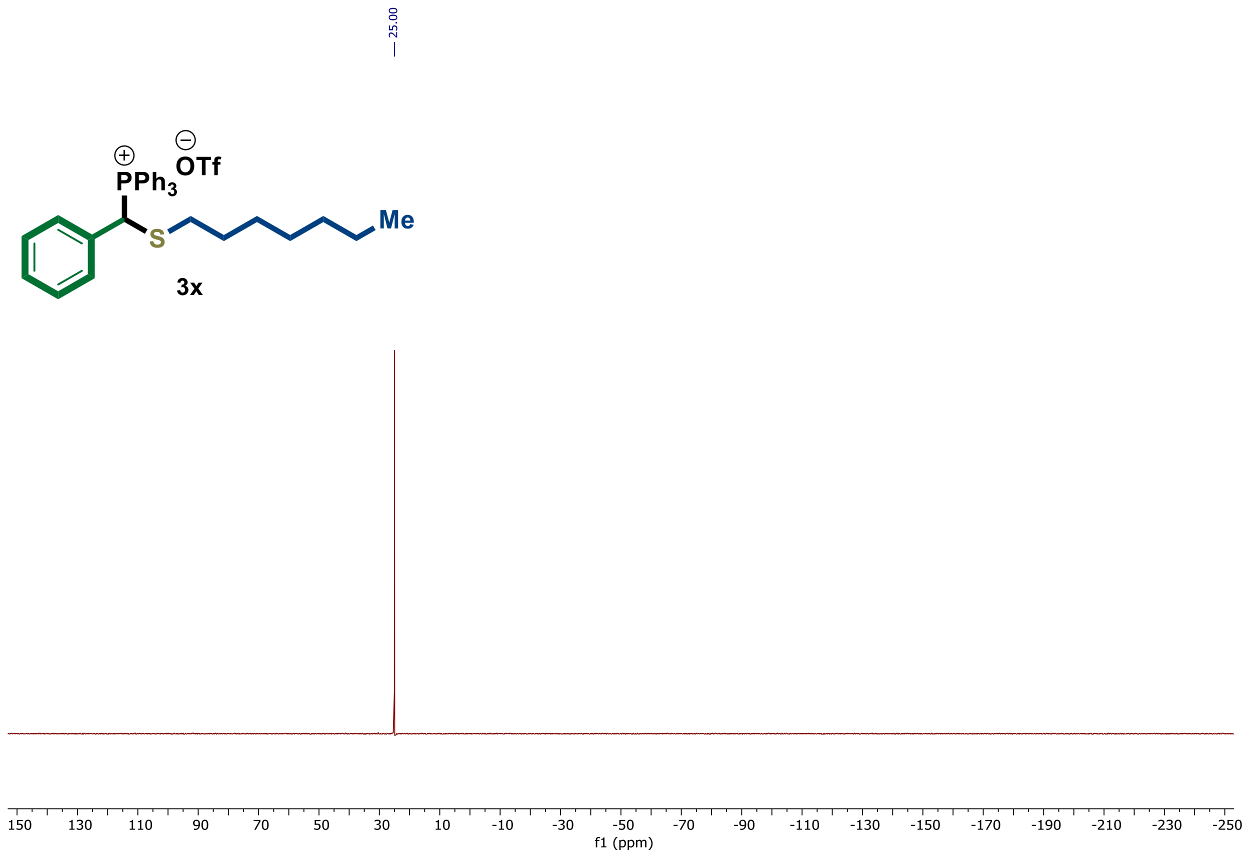

^{19}F NMR (376 MHz, CDCl$_3$) of **3x**

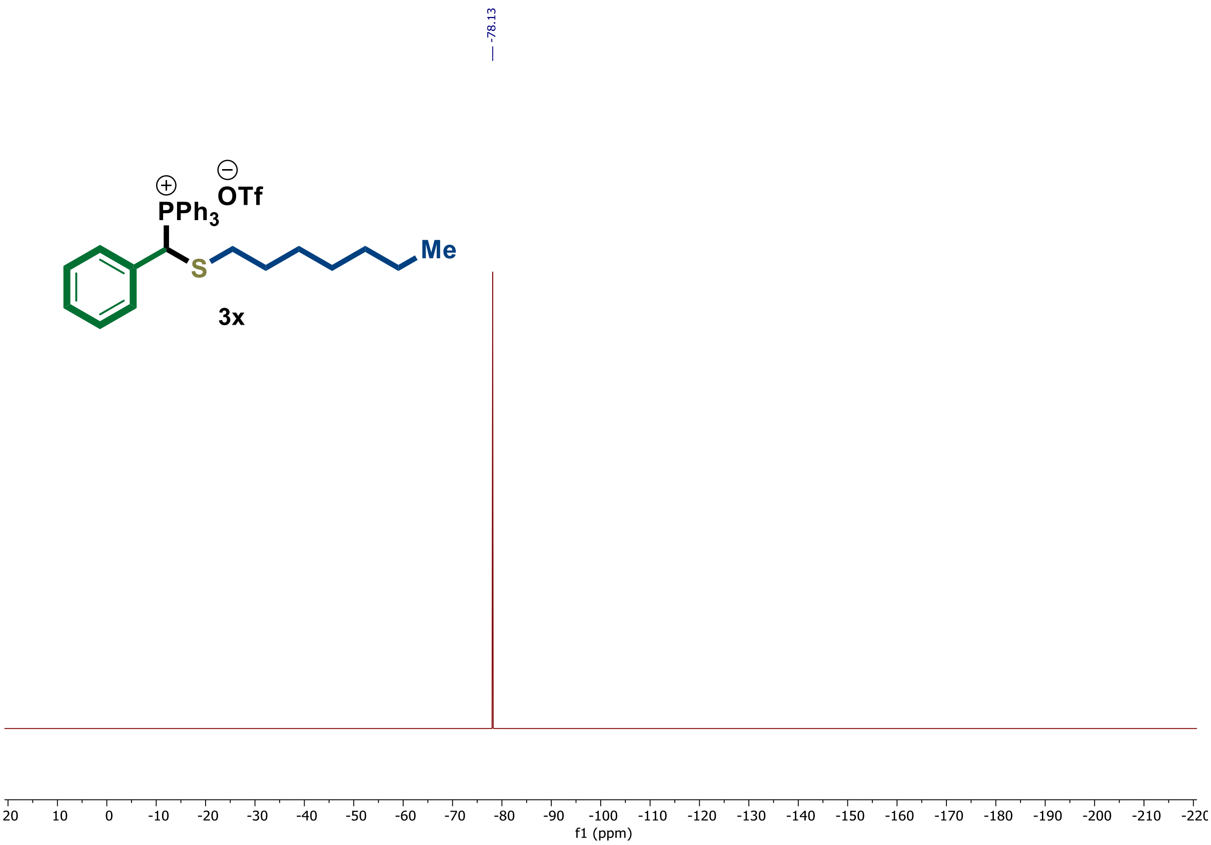

^{1}H NMR (400 MHz, CDCl$_3$) of **3y**

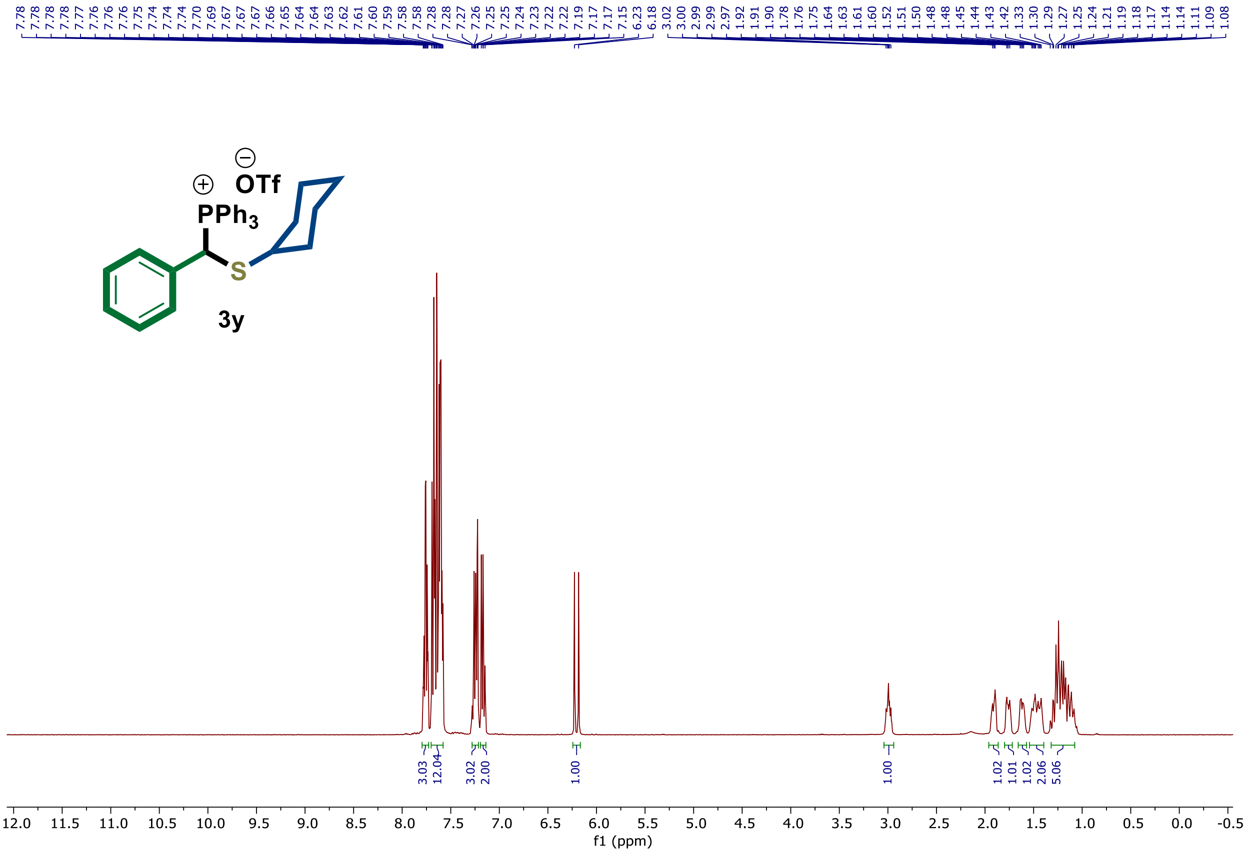

^{13}C NMR (100 MHz, CDCl$_3$) of **3y**

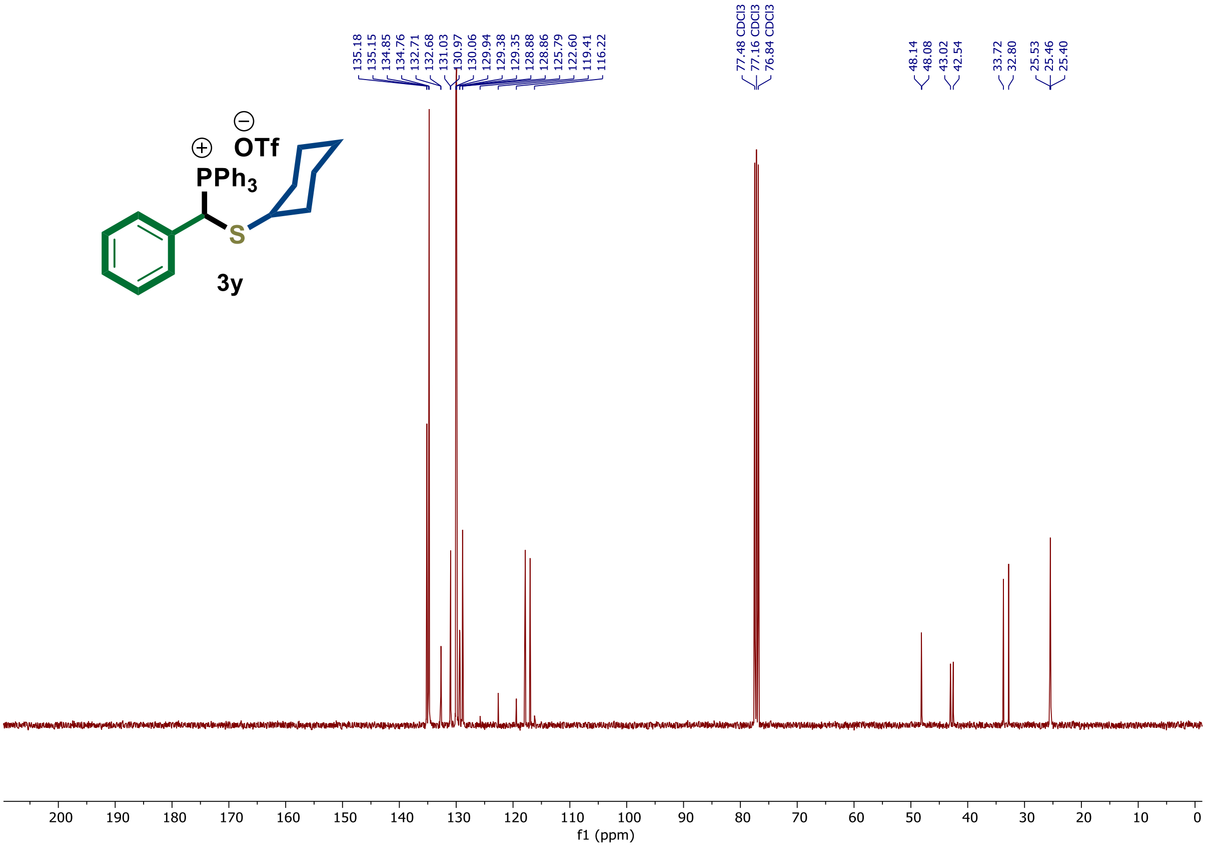

^{31}P NMR (162 MHz, CDCl$_3$) of **3y**

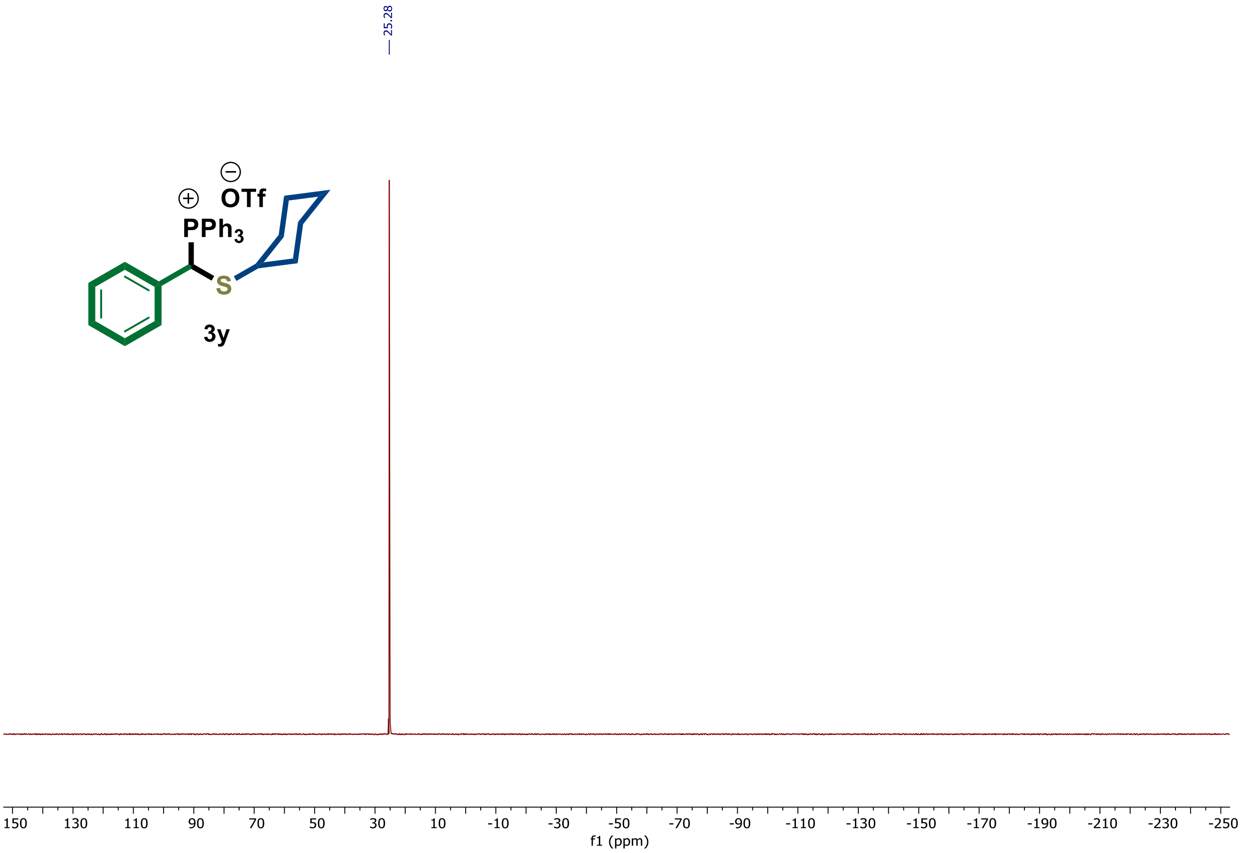

^{19}F NMR (376 MHz, $CDCl_3$) of **3y**

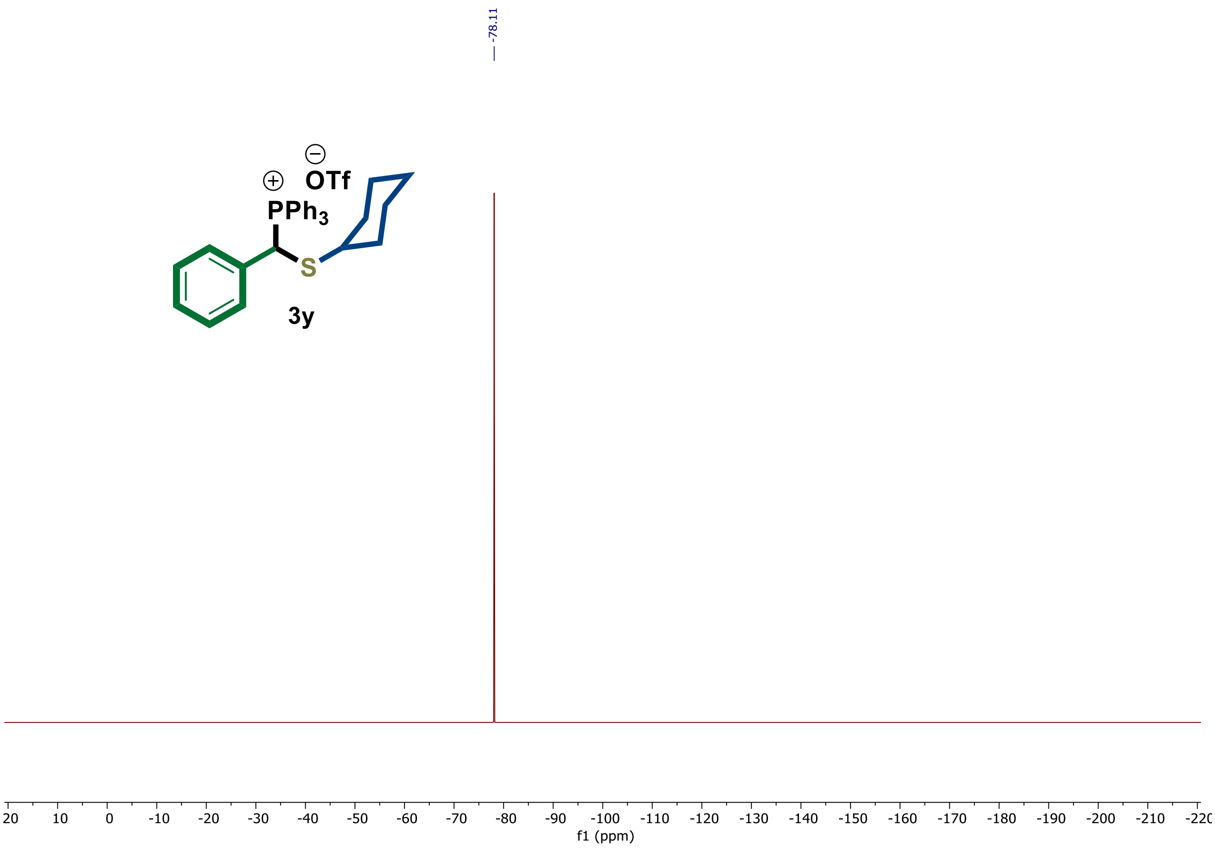

^{1}H NMR (400 MHz, $CDCl_3$) of **3z**

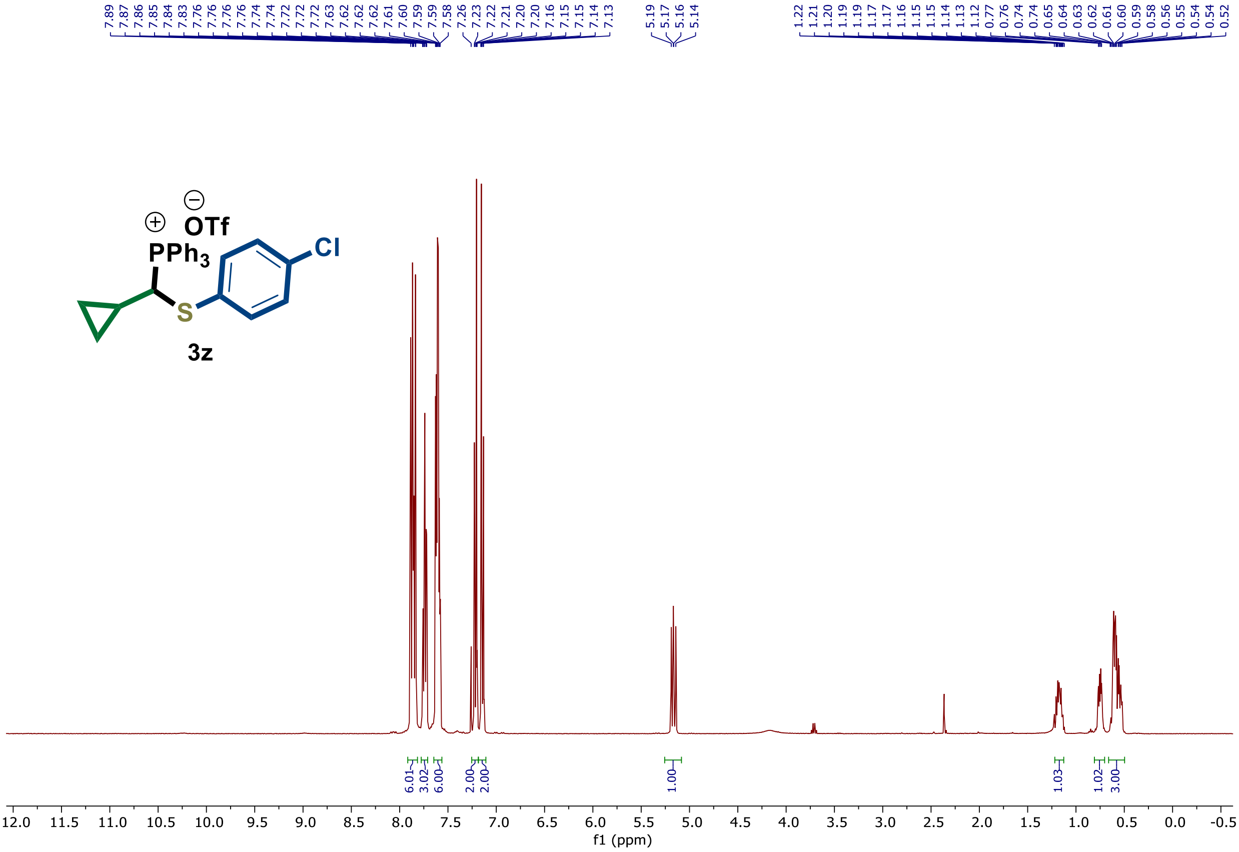

^{13}C NMR (100 MHz, $CDCl_3$) of **3z**

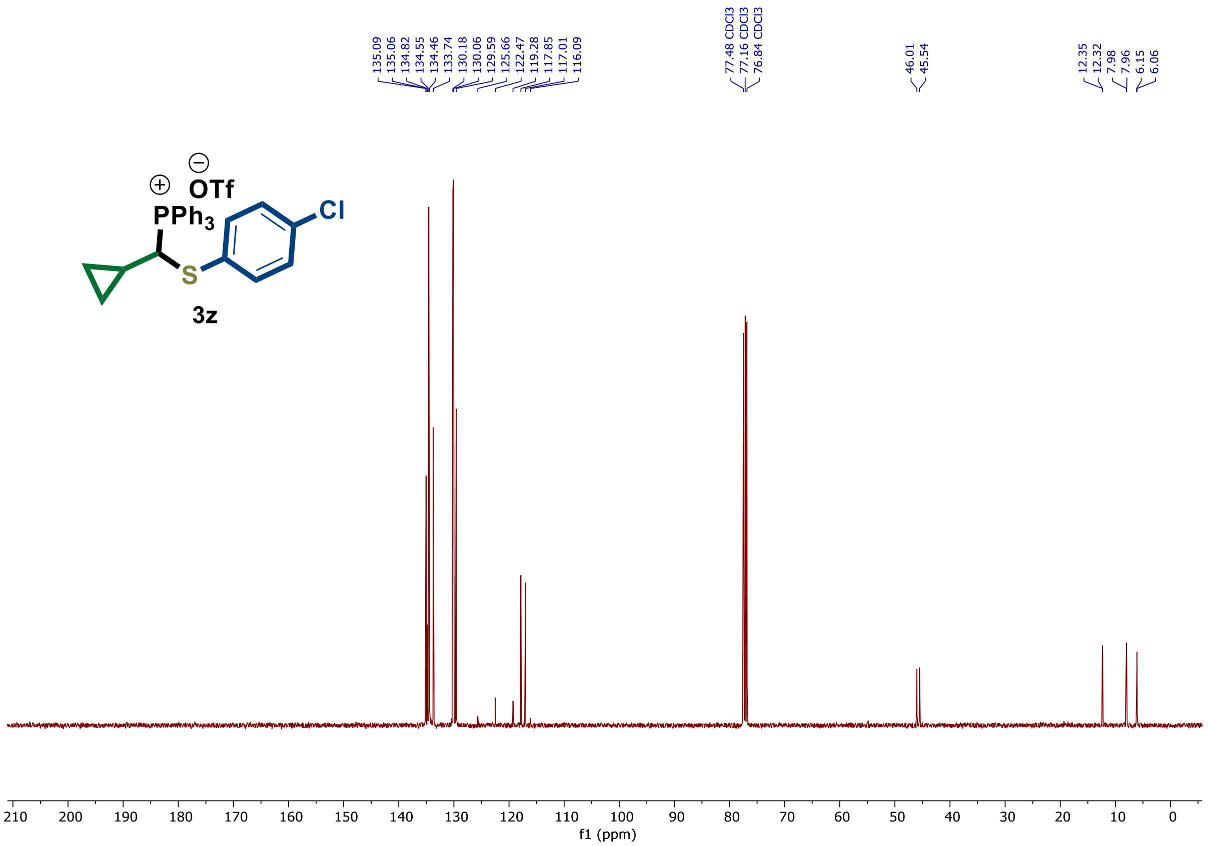

^{31}P NMR (162 MHz, $CDCl_3$) of **3z**

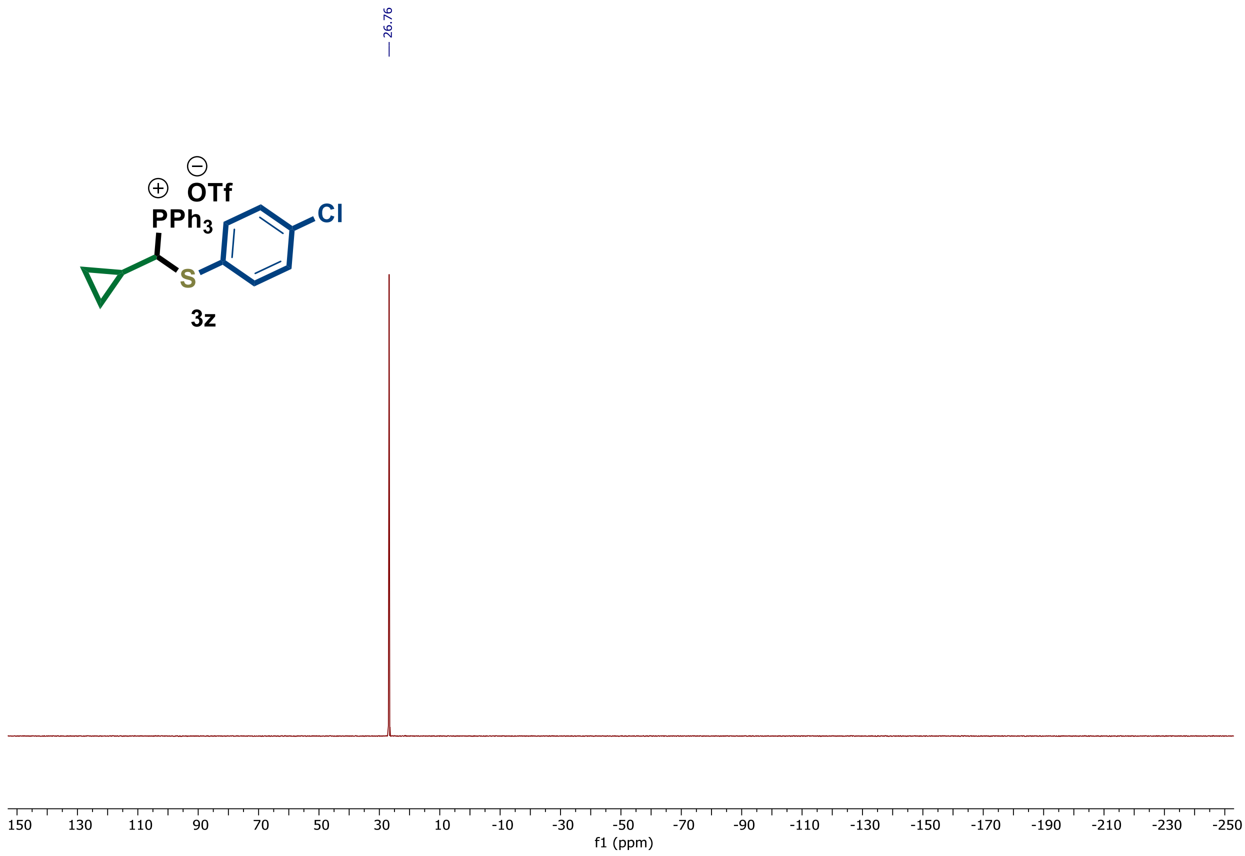

^{19}F NMR (376 MHz, CDCl$_3$) of **3z**

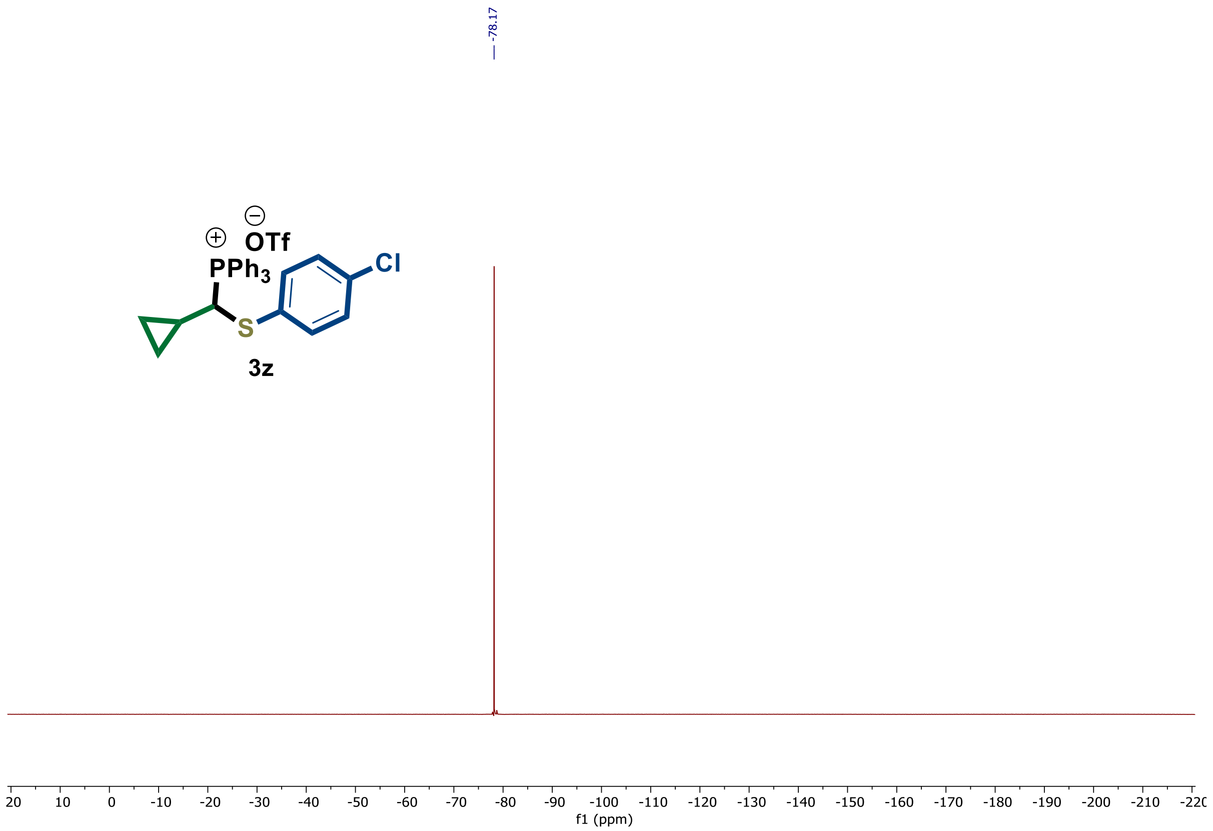

^{1}H NMR (400 MHz, CDCl$_3$) of **3aa**

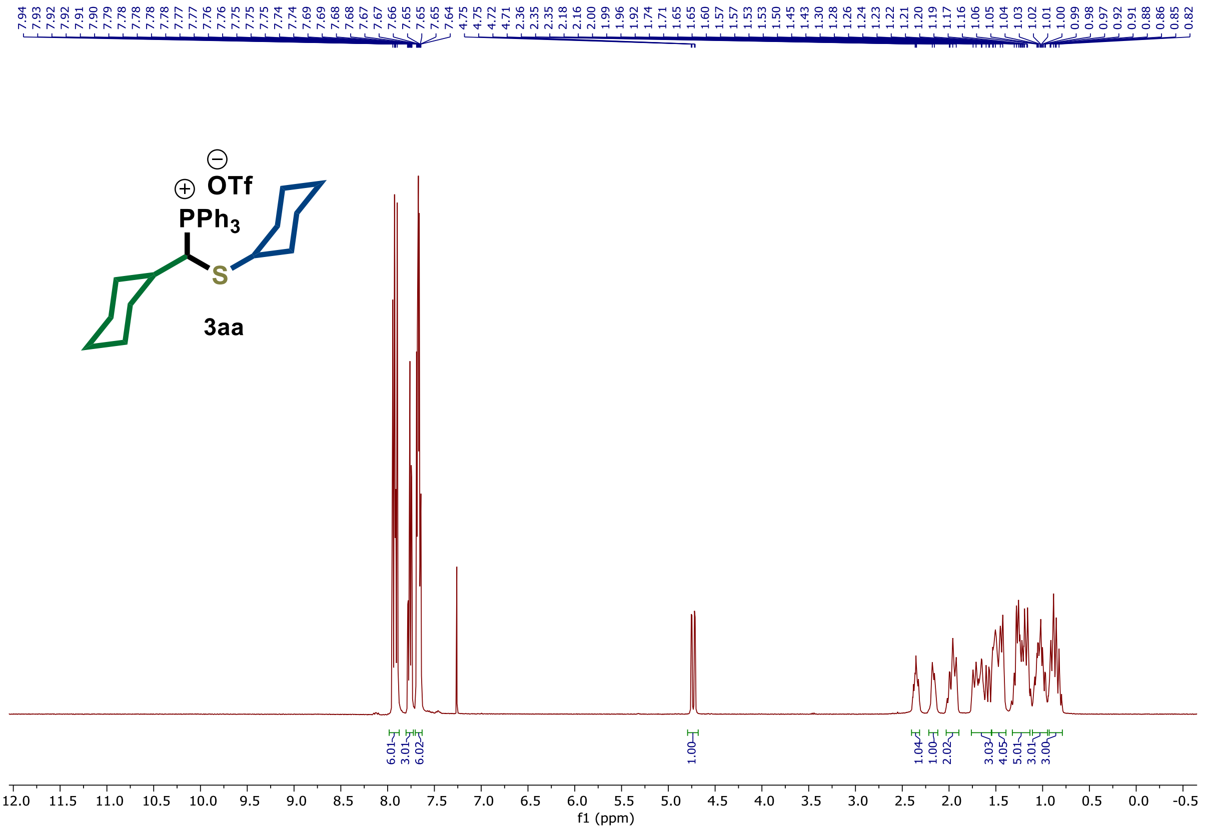

^{13}C NMR (100 MHz, $CDCl_3$) of **3aa**

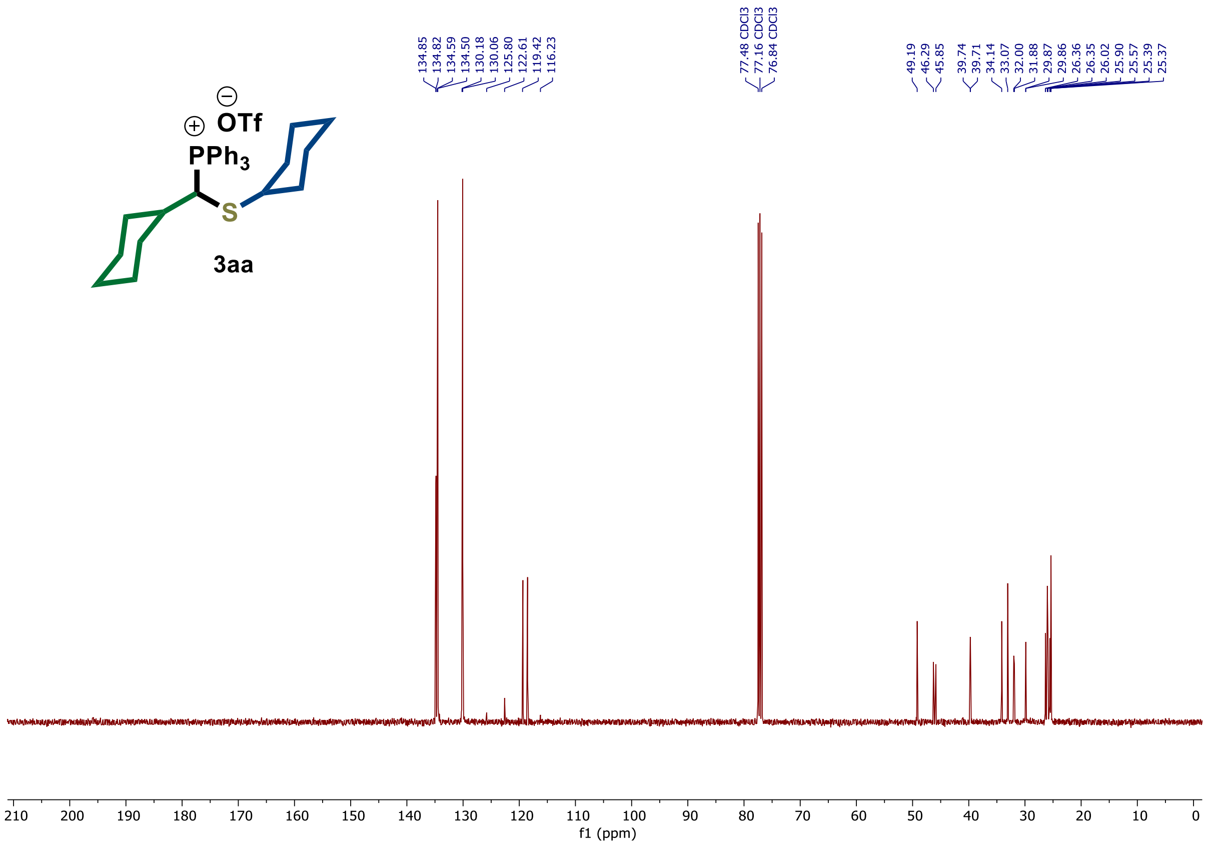

^{31}P NMR (162 MHz, $CDCl_3$) of **3aa**

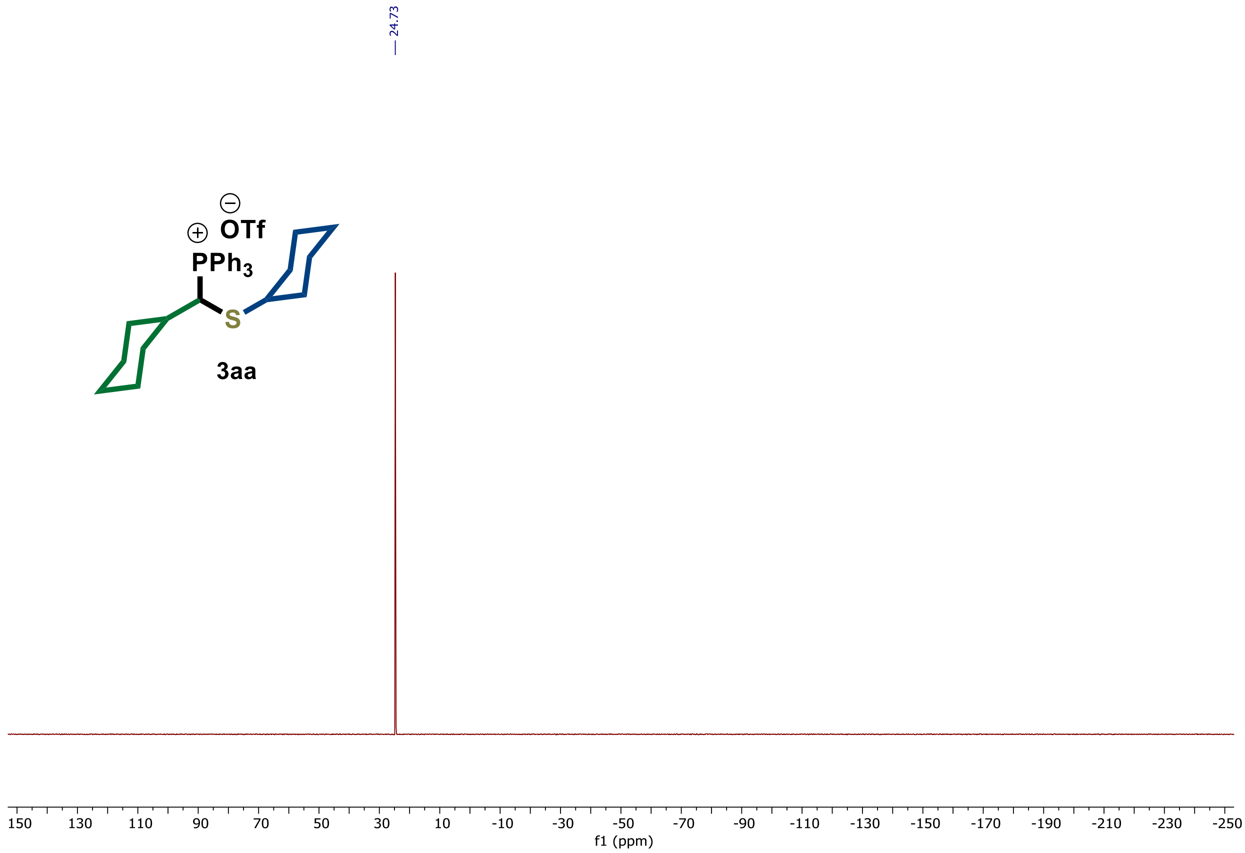

^{19}F NMR (376 MHz, $CDCl_3$) of **3aa**

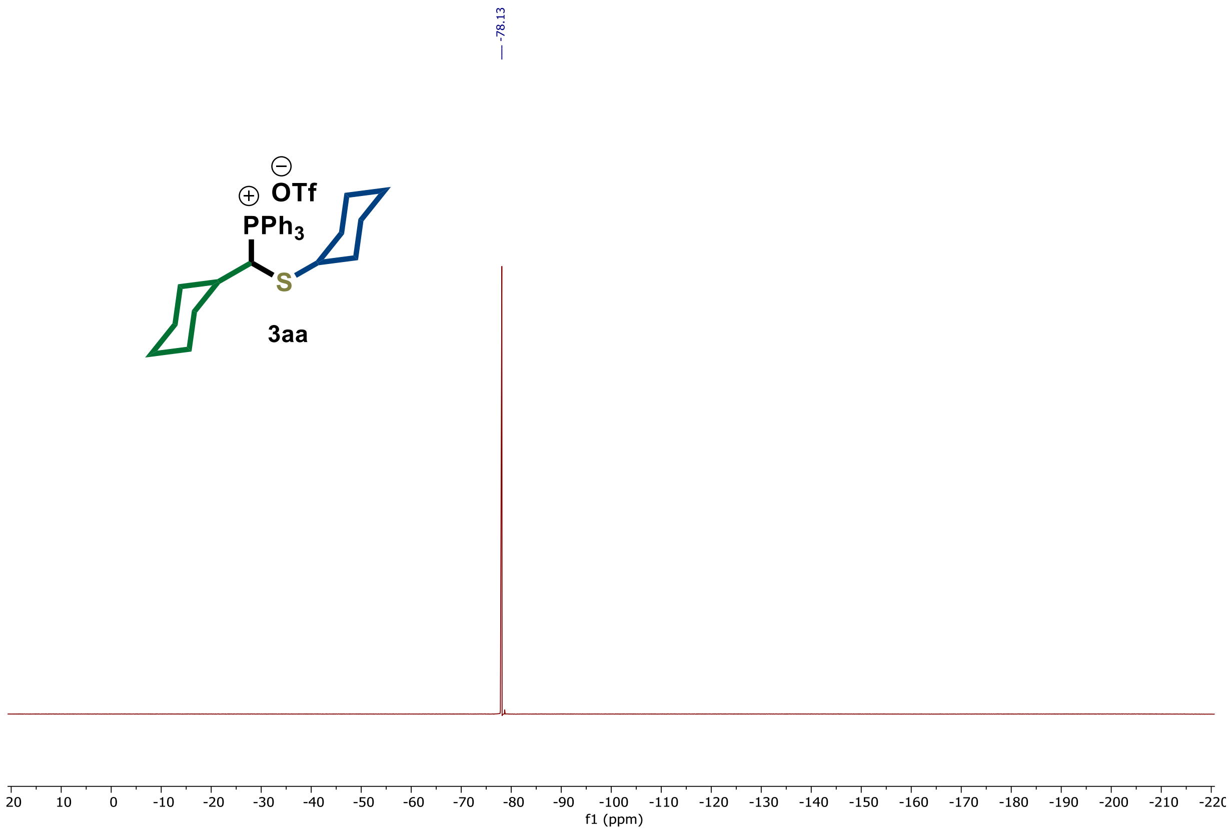

^{1}H NMR (400 MHz, $CDCl_3$) of **3ab**

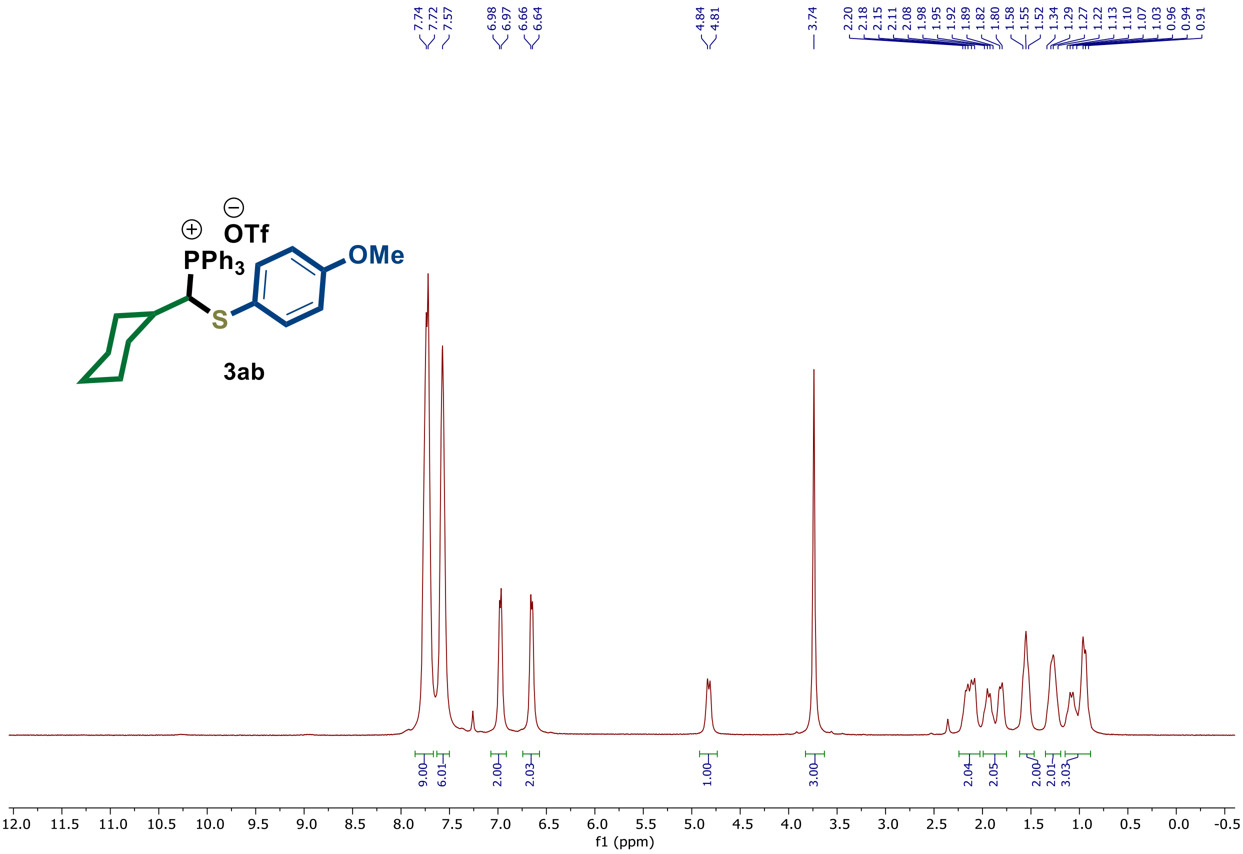

^{13}C NMR (100 MHz, CDCl$_3$) of **3ab**

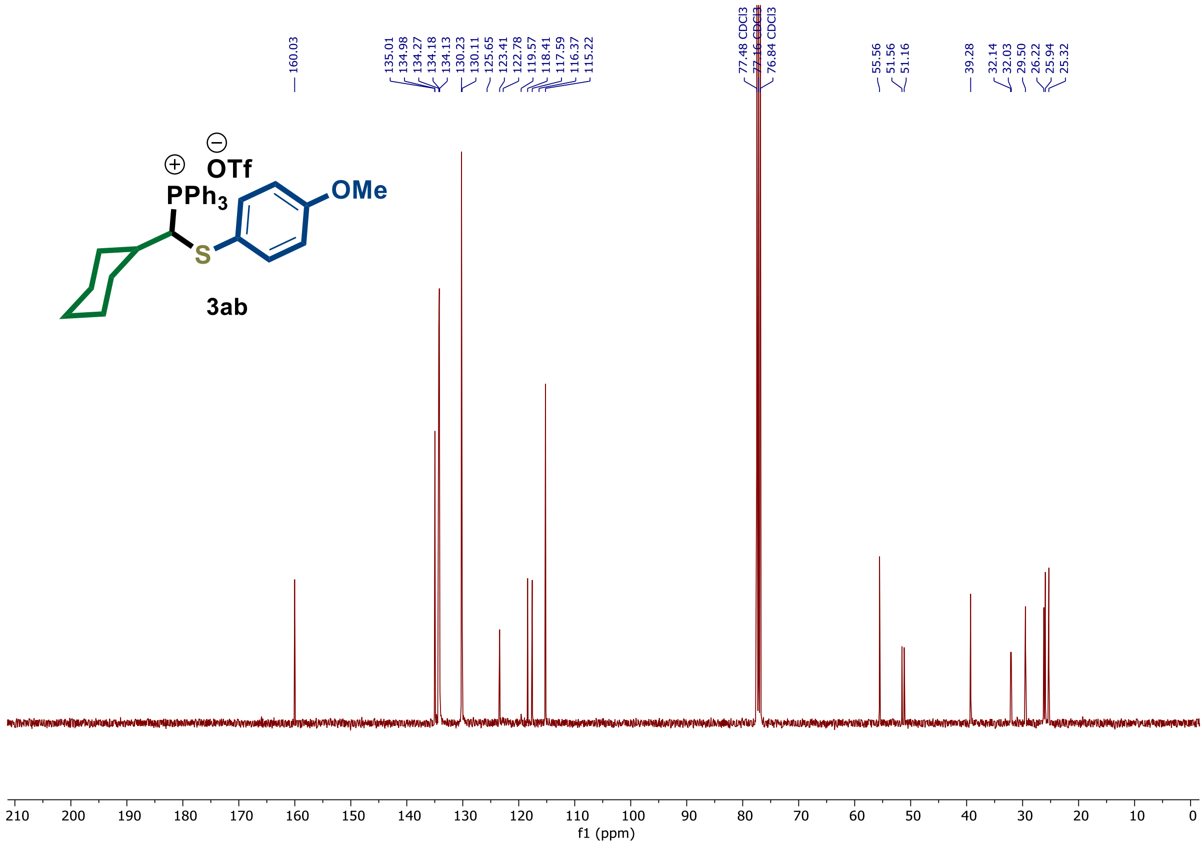

^{31}P NMR (162 MHz, CDCl$_3$) of **3ab**

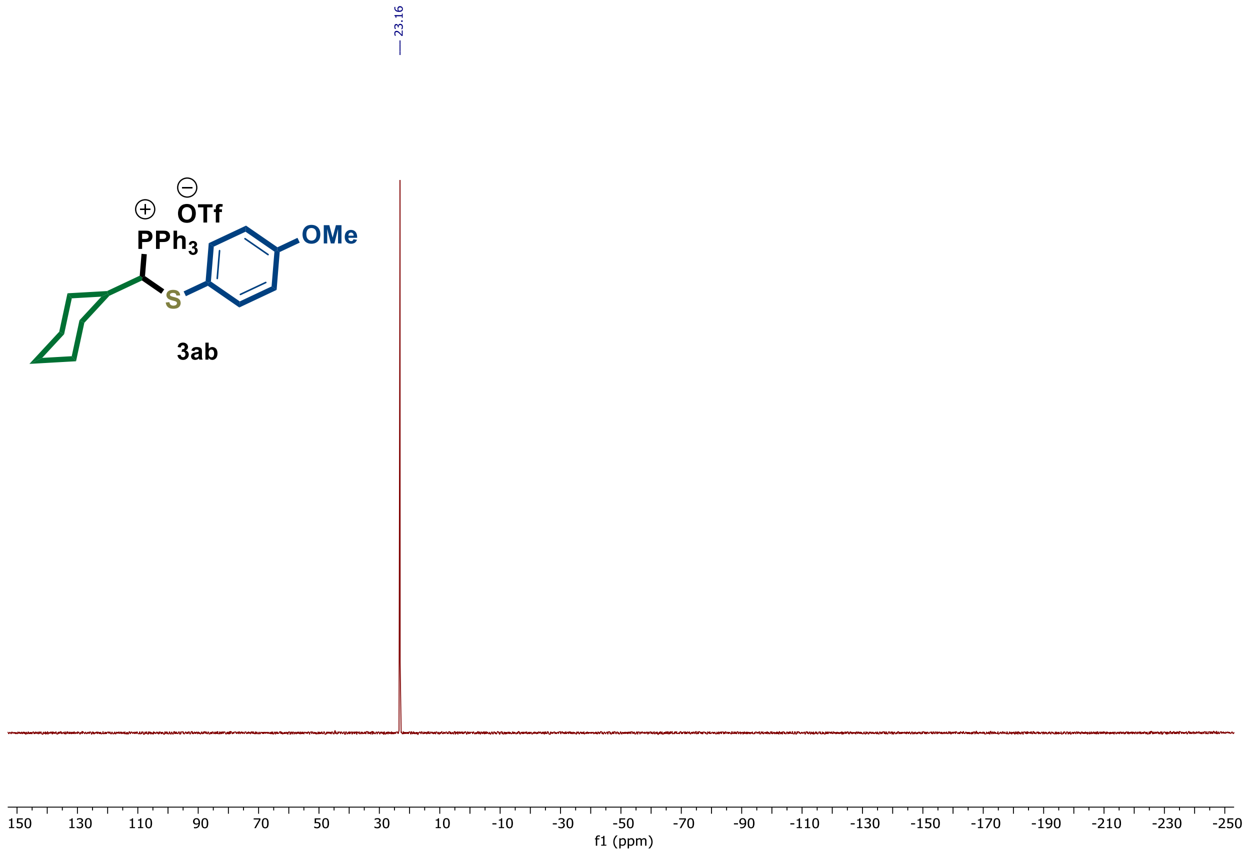

^{19}F NMR (376 MHz, CDCl$_3$) of **3ab**

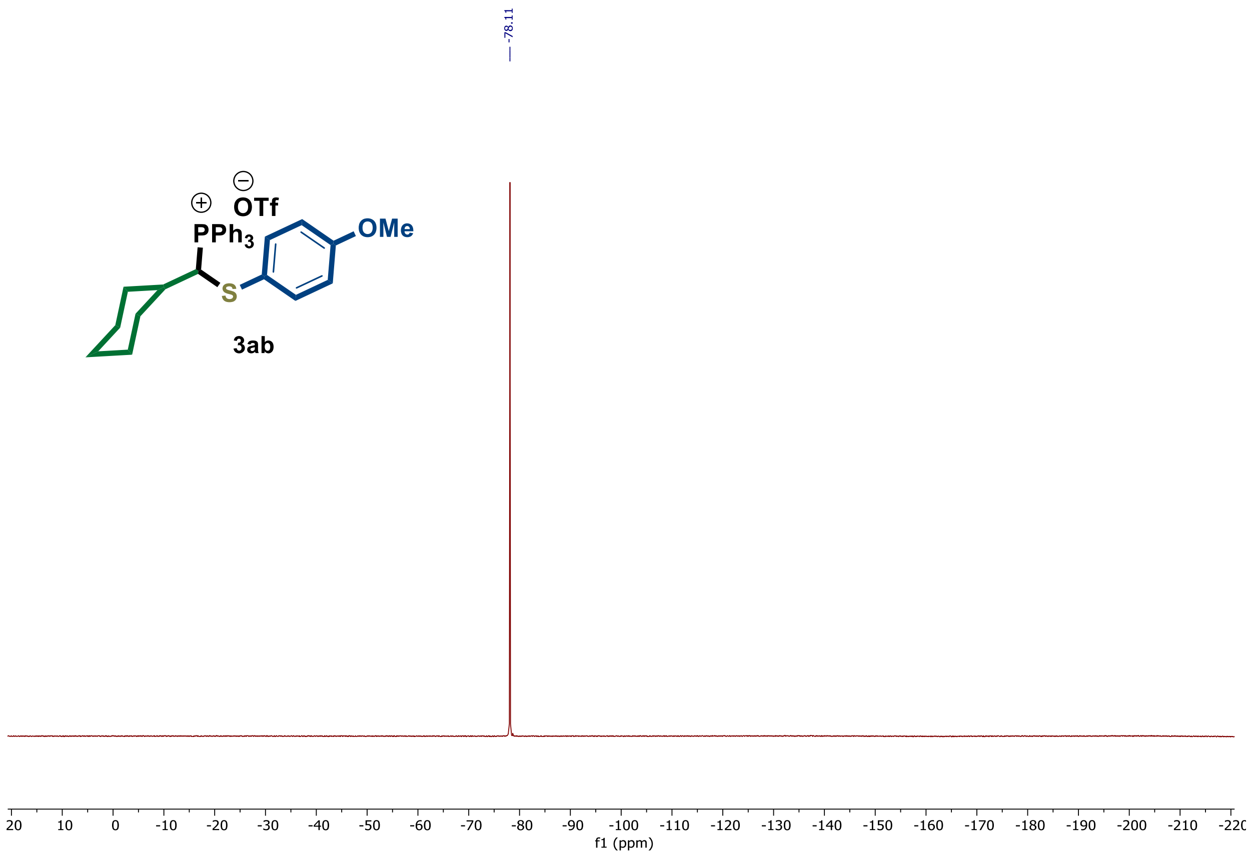

^{1}H NMR (400 MHz, CDCl$_3$) of **3ac**

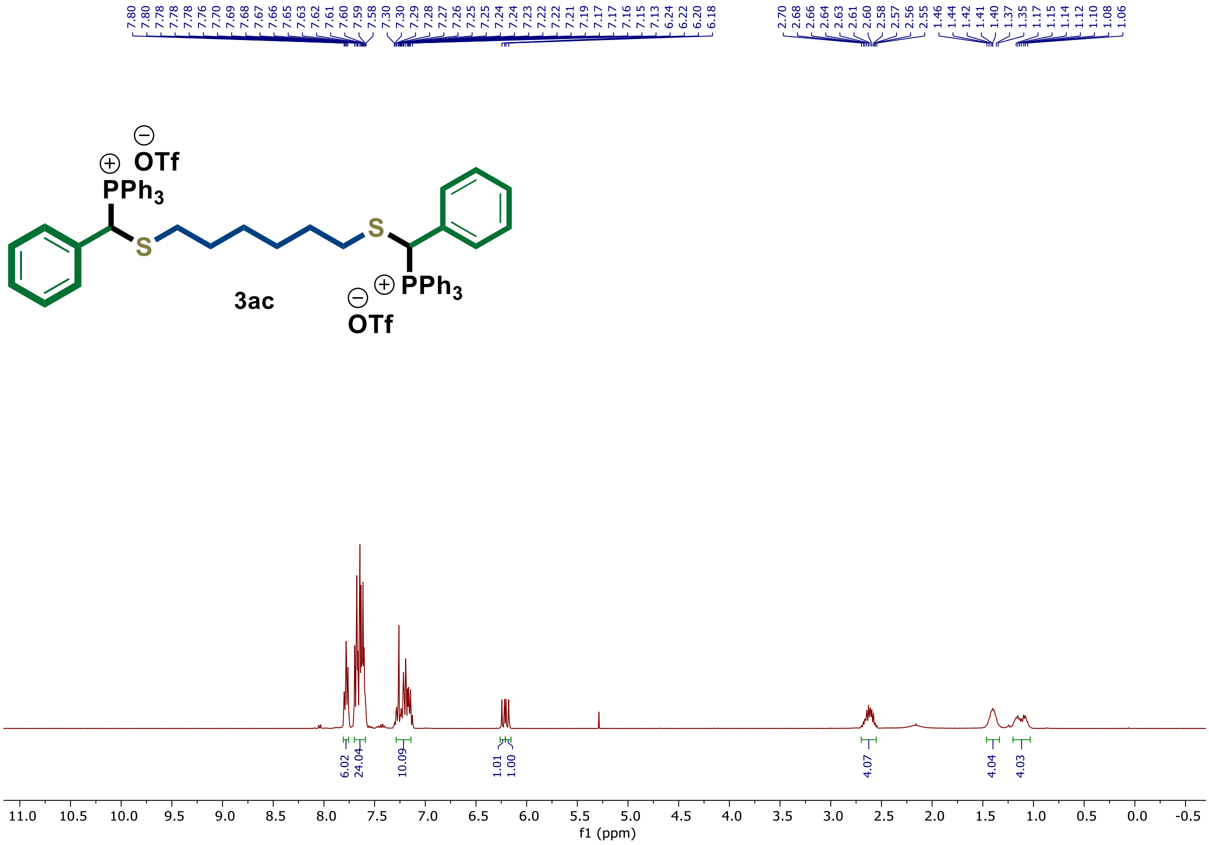

^{13}C NMR (100 MHz, CDCl$_3$) of **3ac**

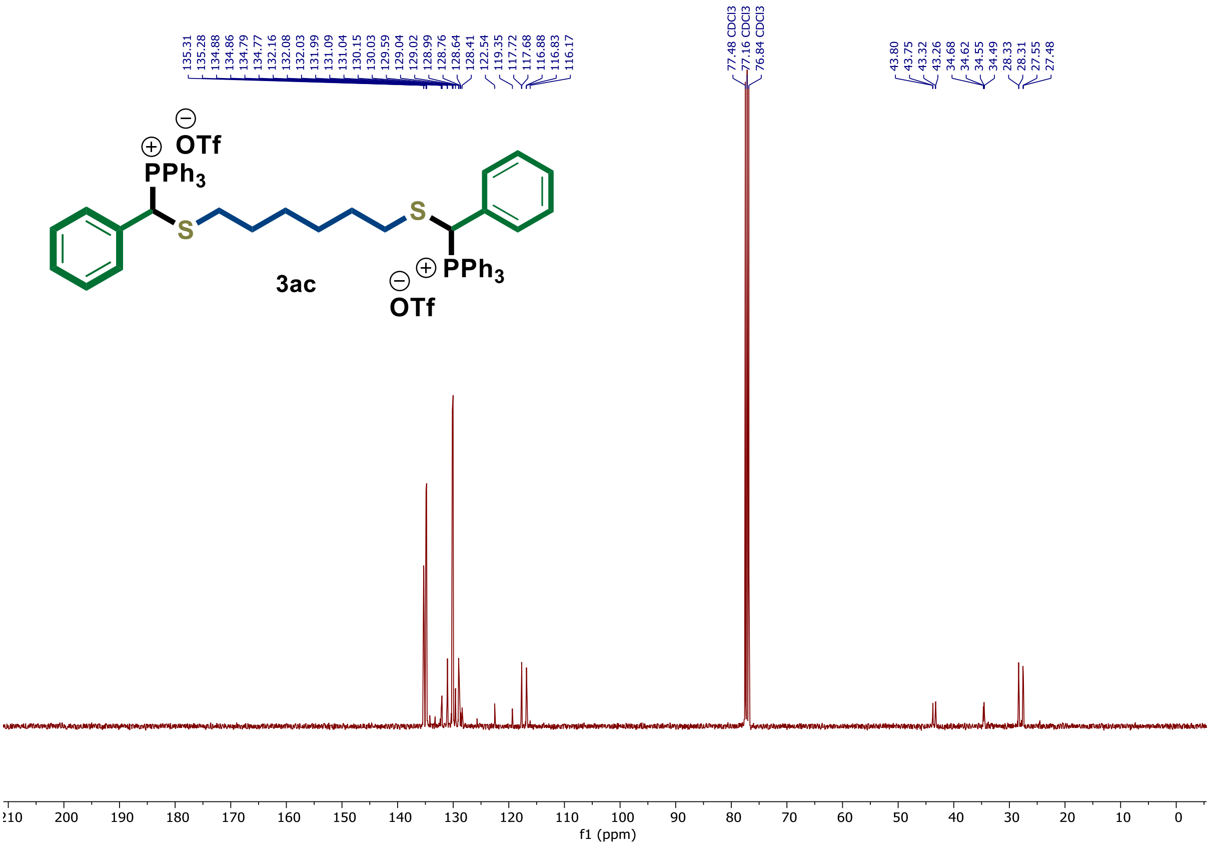

^{31}P NMR (162 MHz, CDCl$_3$) of **3ac**

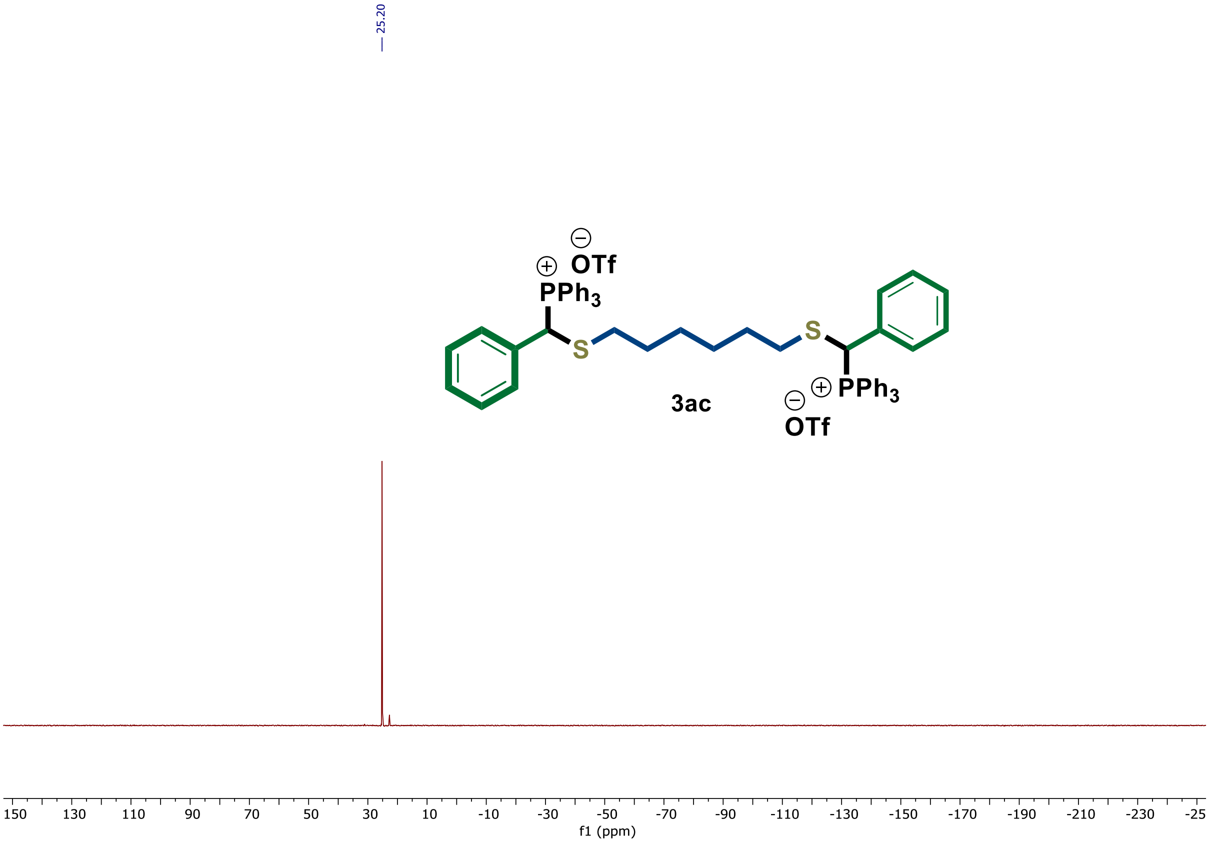

^{19}F NMR (376 MHz, $CDCl_3$) of **3ac**

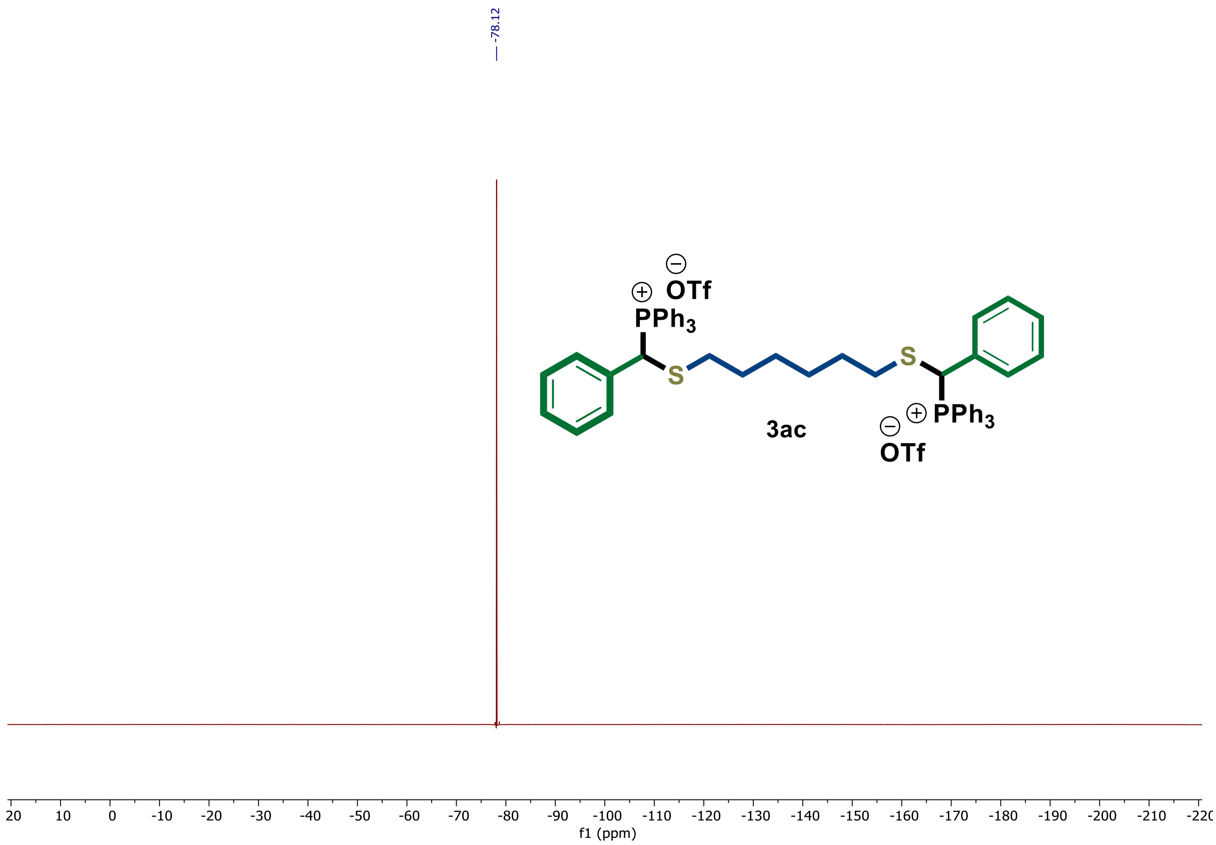

^{1}H NMR (400 MHz, $CDCl_3$) of **3ad**

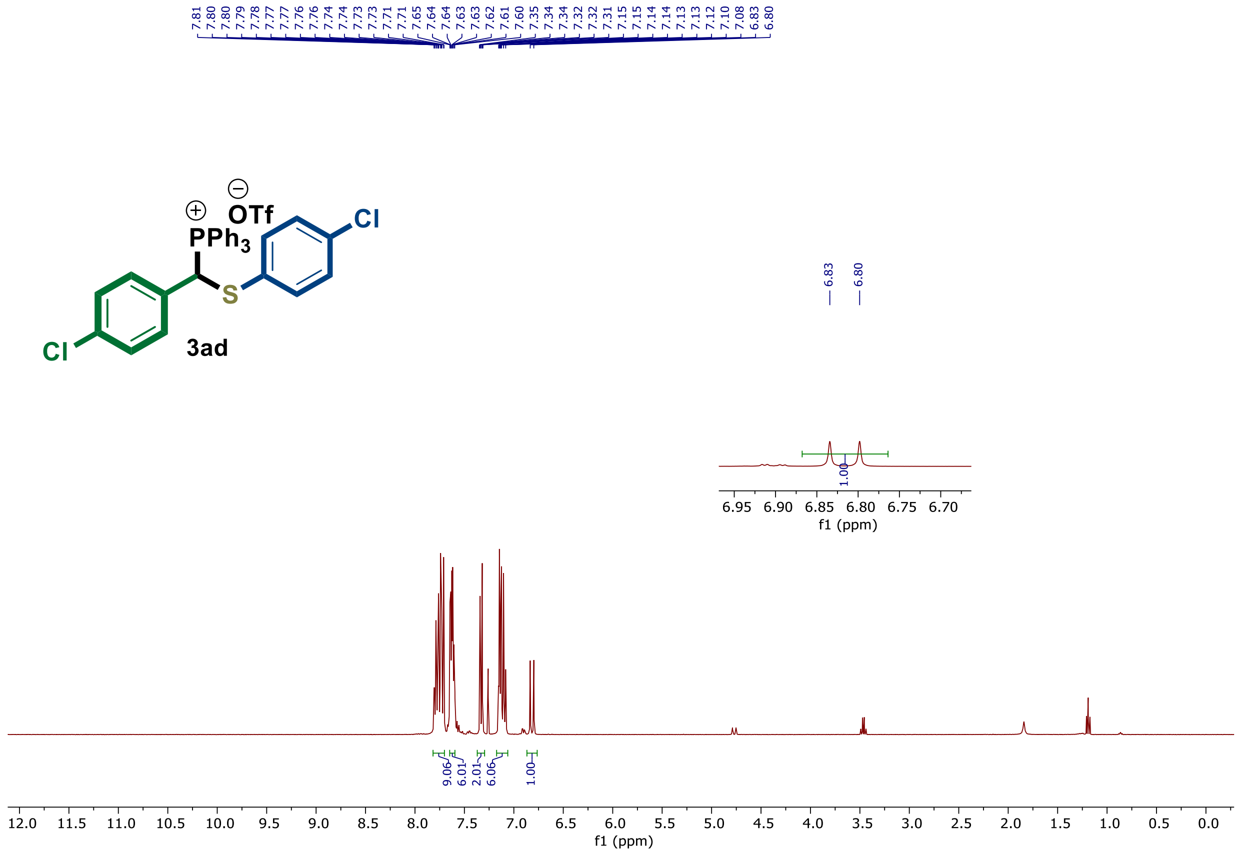

^{13}C NMR (100 MHz, $CDCl_3$) of **3ad**

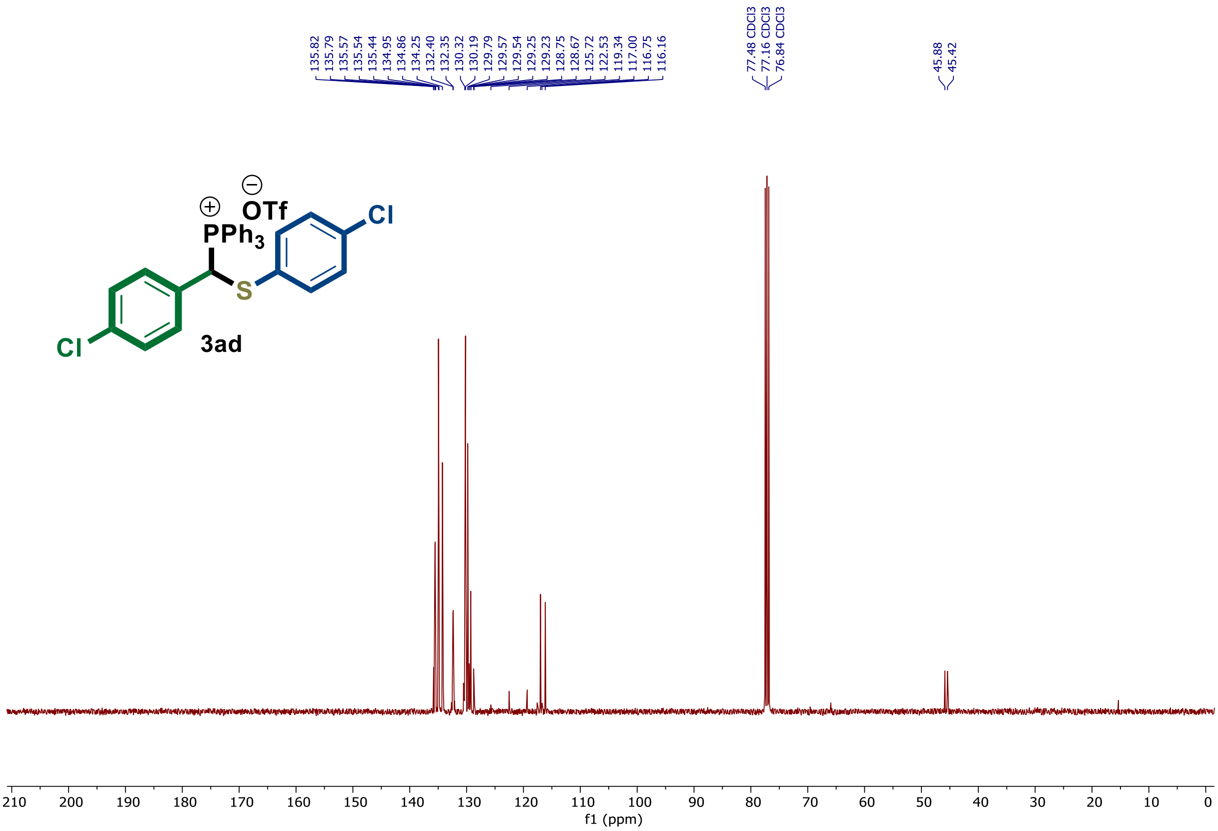

^{31}P NMR (162 MHz, $CDCl_3$) of **3ad**

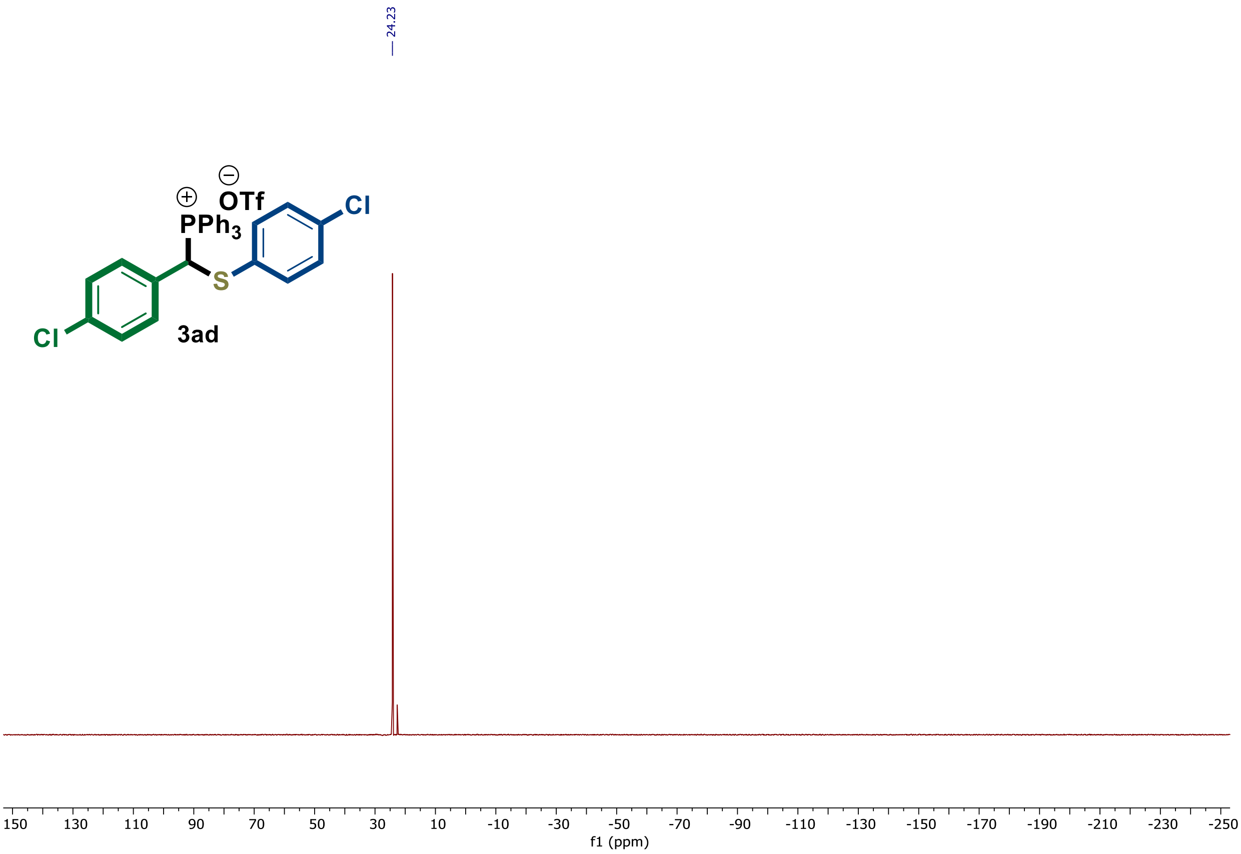

^{19}F NMR (376 MHz, CDCl$_3$) of **3ad**

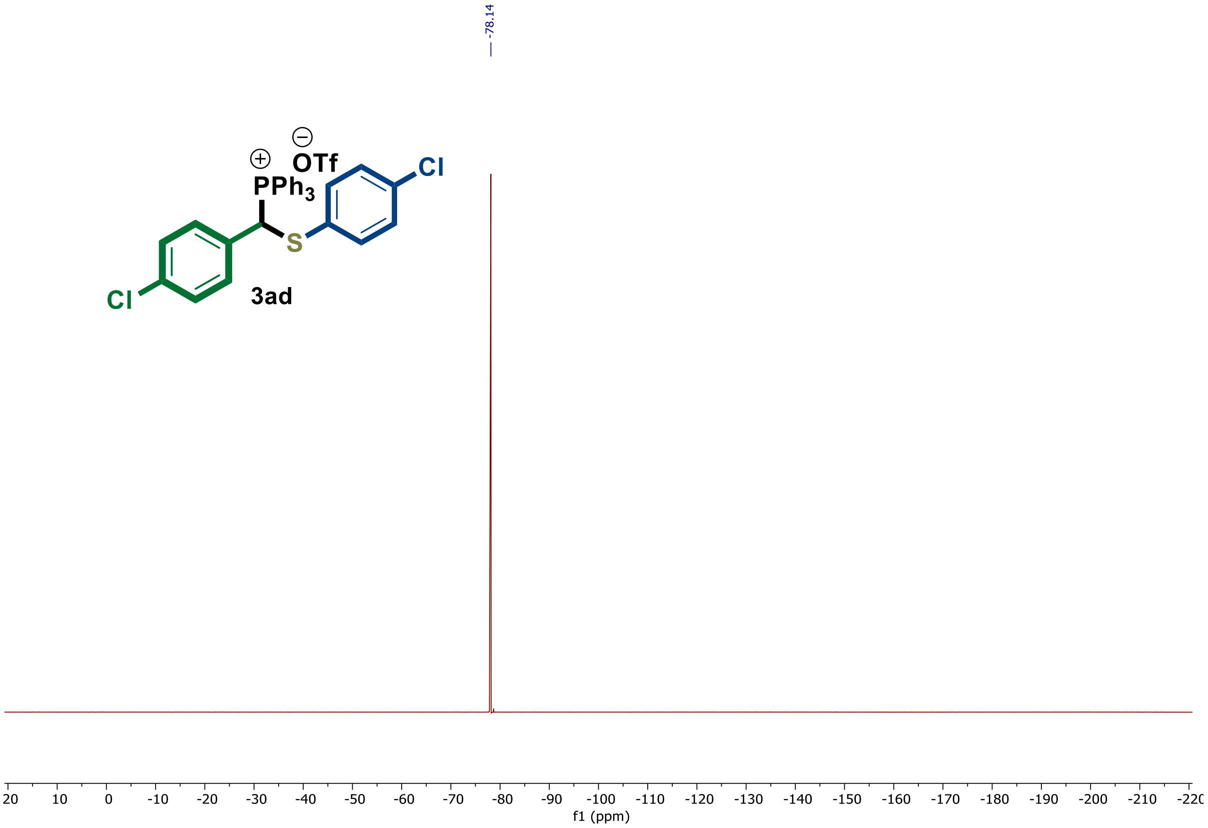

^{1}H NMR (500 MHz, CD$_3$CN) of **3ae**

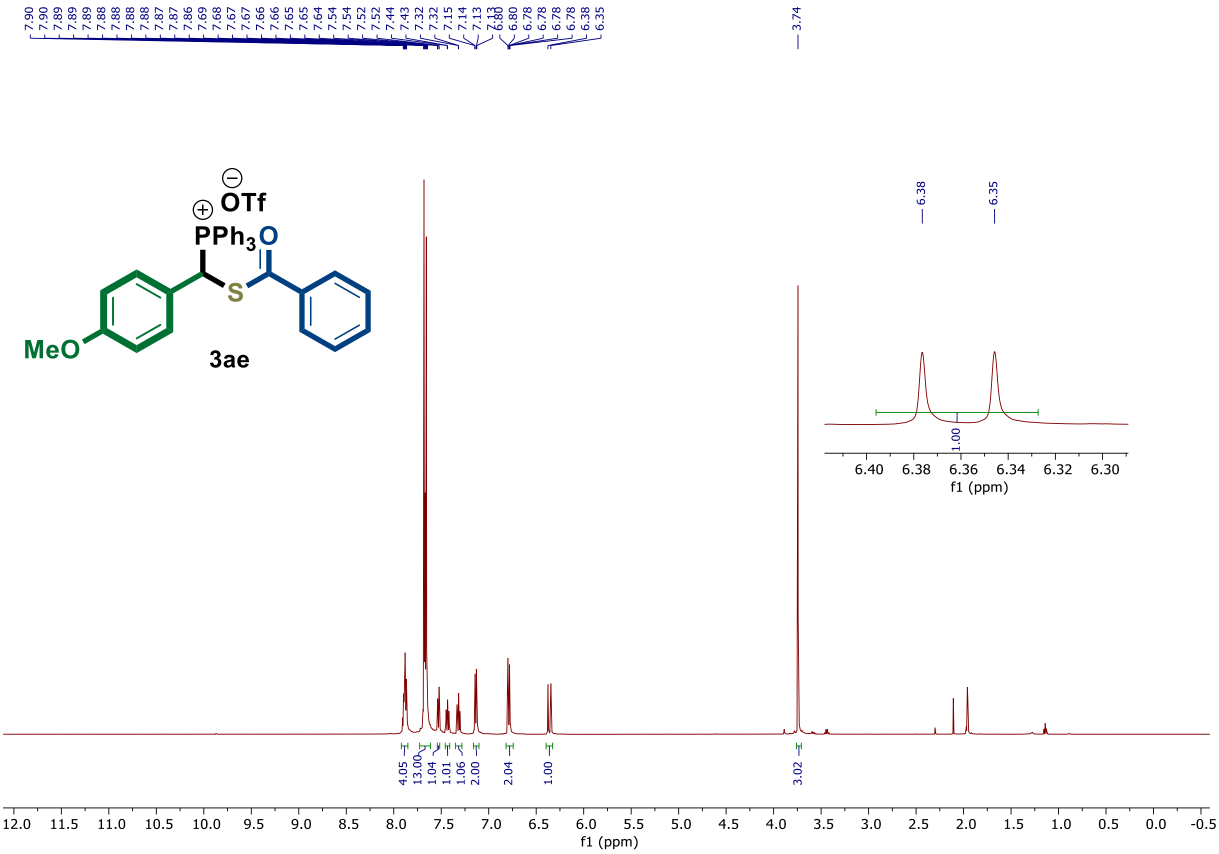

^{1}H NMR (125 MHz, CD3CN) of **3ae**

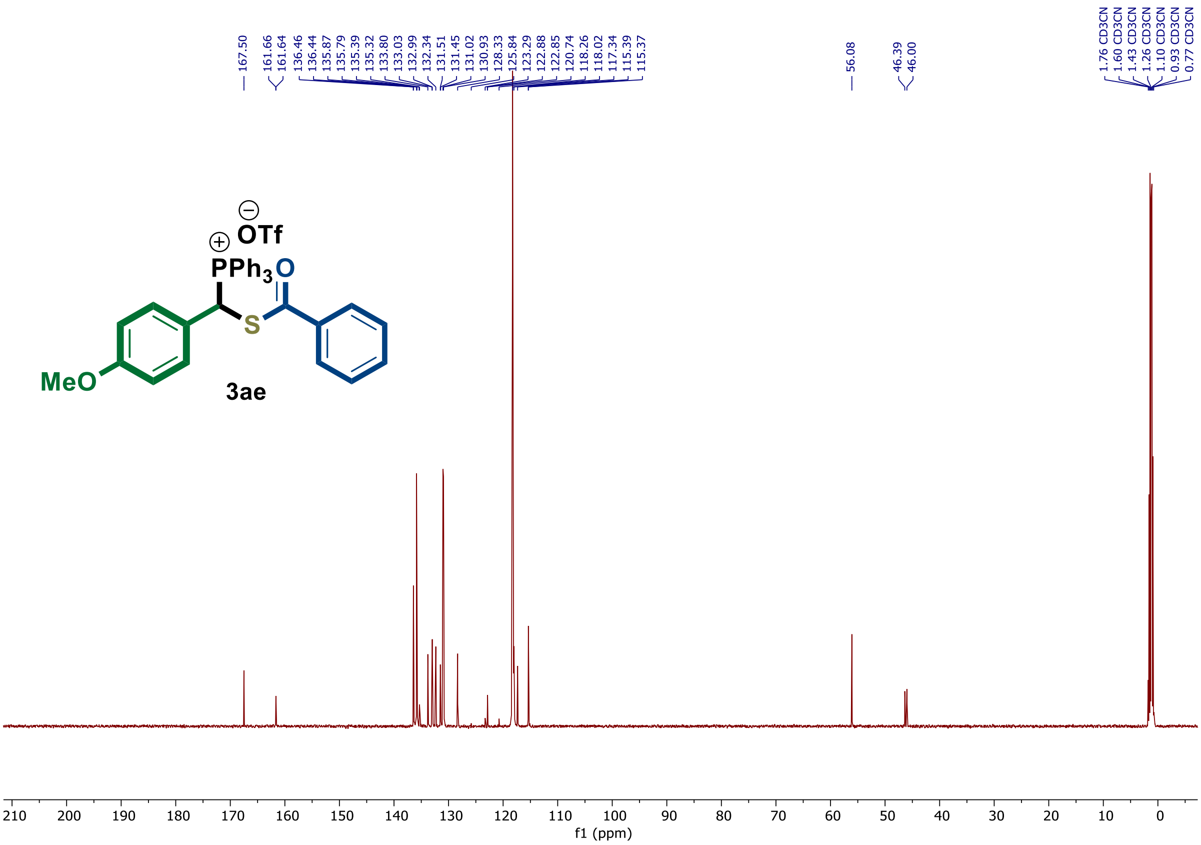

^{31}P NMR (162 MHz, CD3CN) of **3ae**

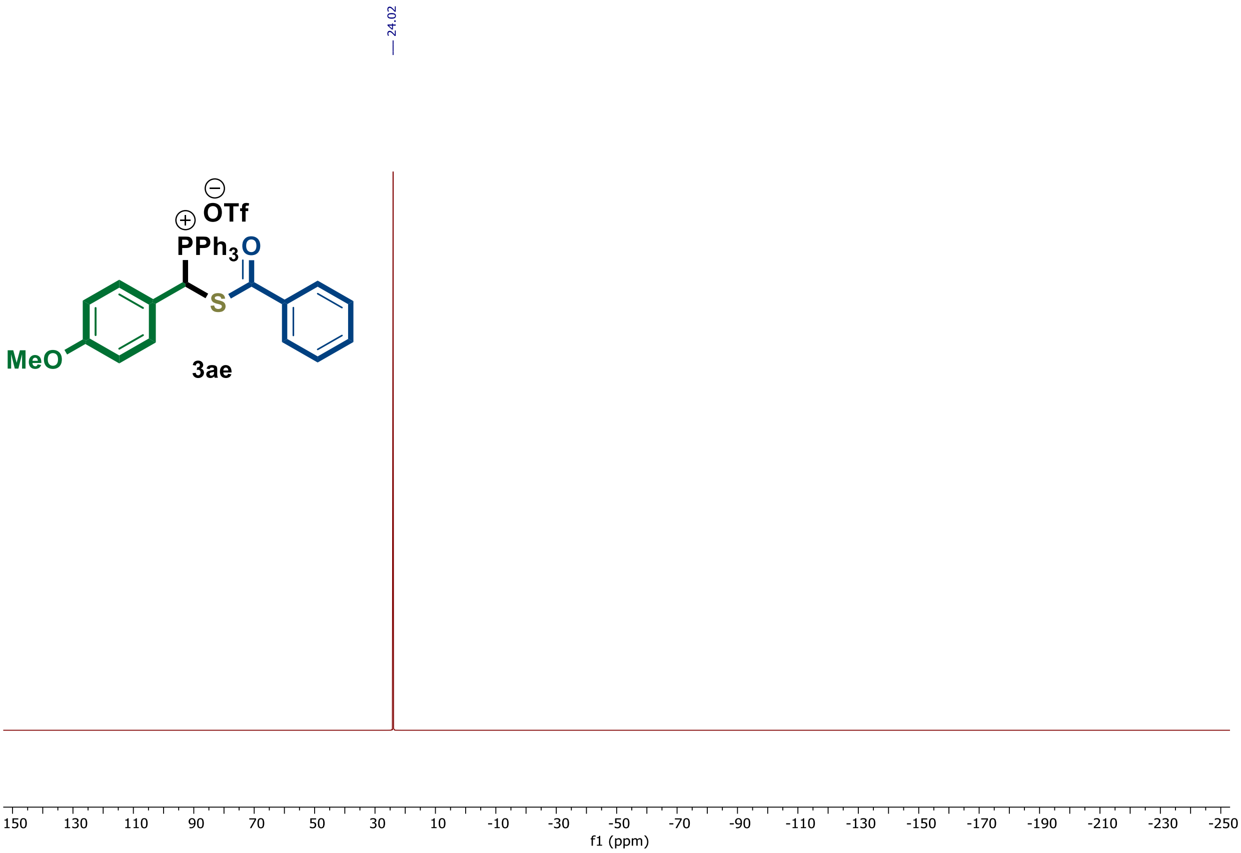

^{19}F NMR (471 MHz, CD_3CN) of **3ae**

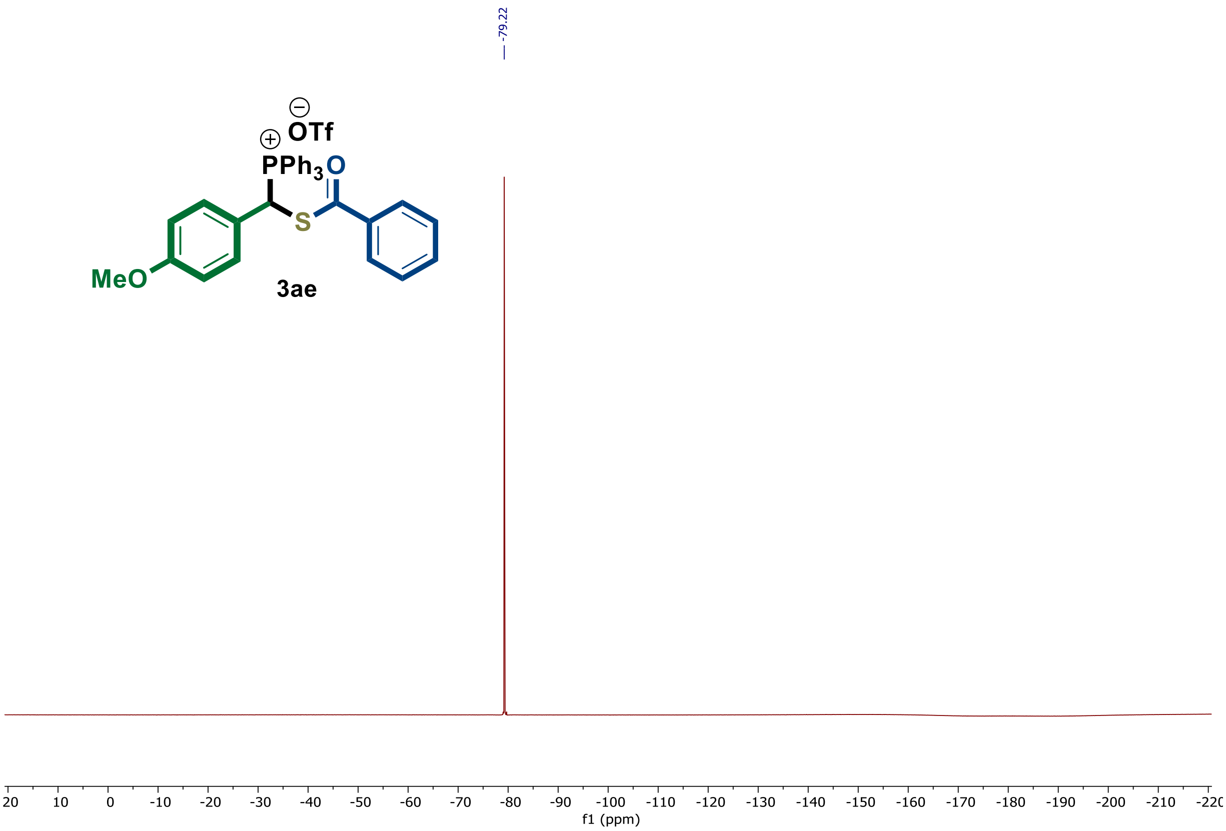

^{1}H NMR (500 MHz, $CDCl_3$) of **4a**

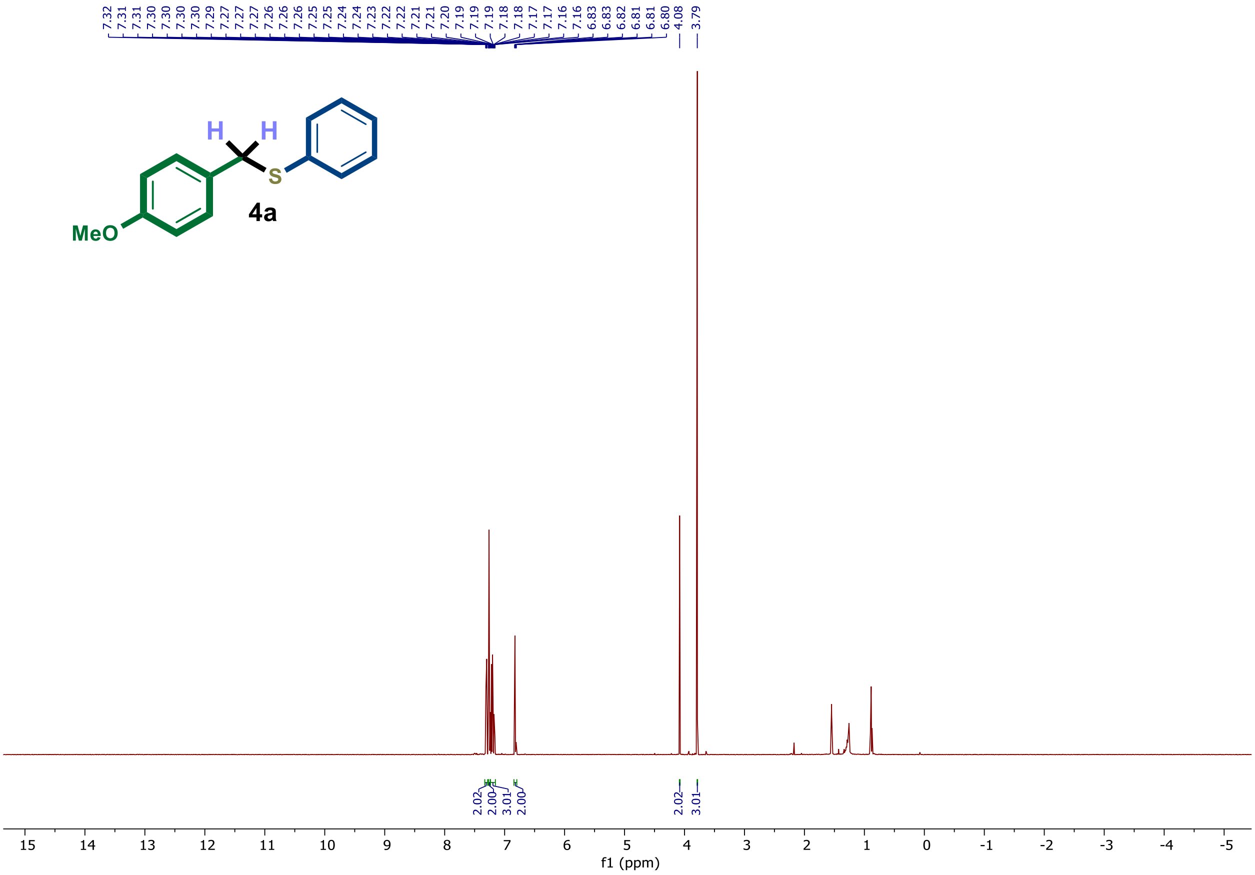

^{13}C NMR (126 MHz, $CDCl_3$) of **4a**

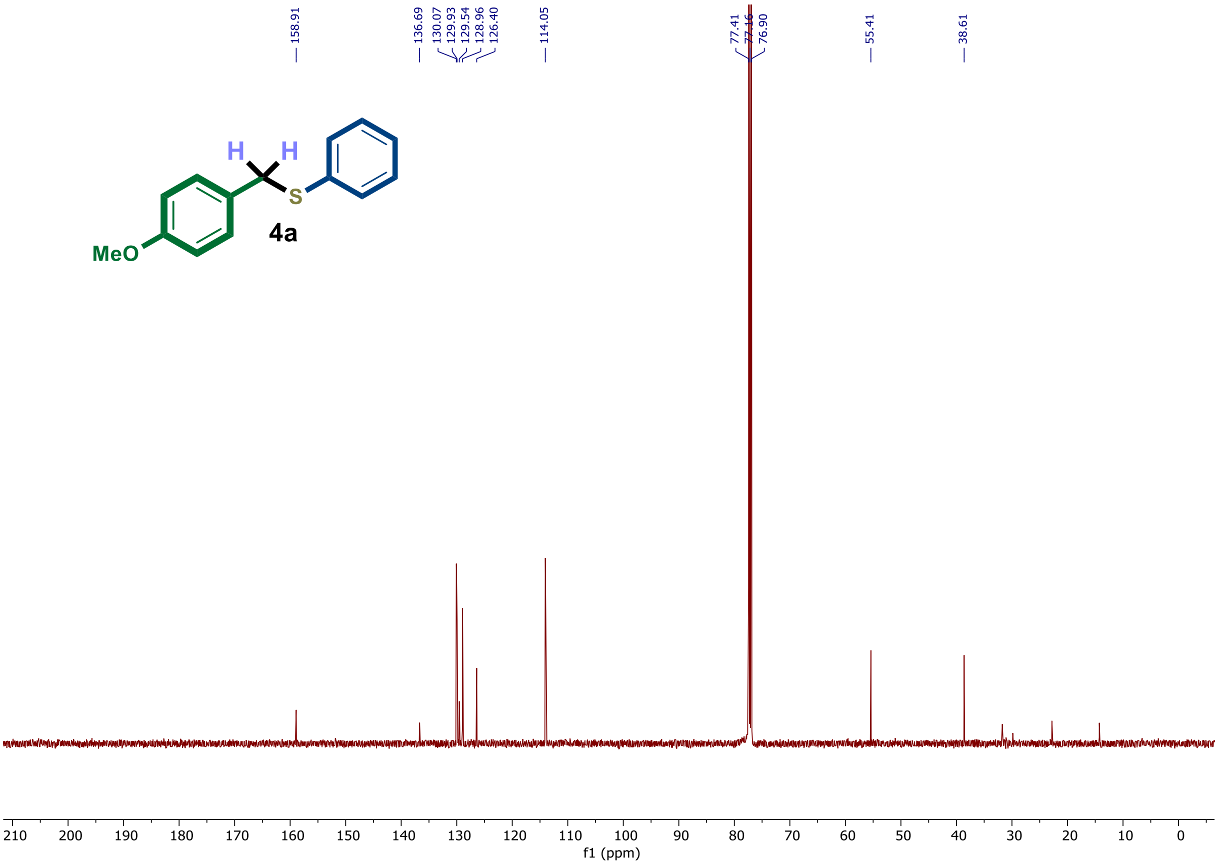

^{1}H NMR (400 MHz, $CDCl_3$) of **4b**

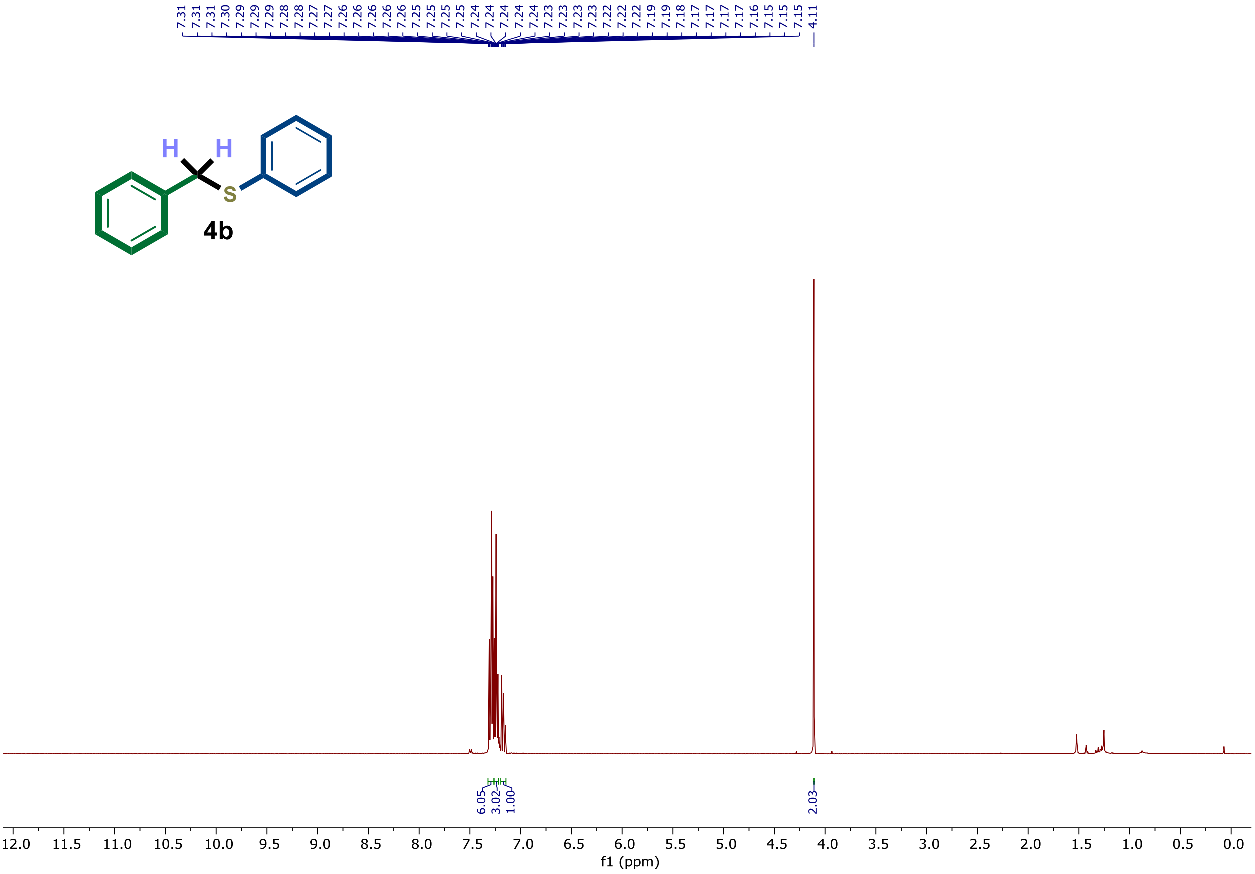

^{13}C NMR (100 MHz, CDCl3) of **4b**

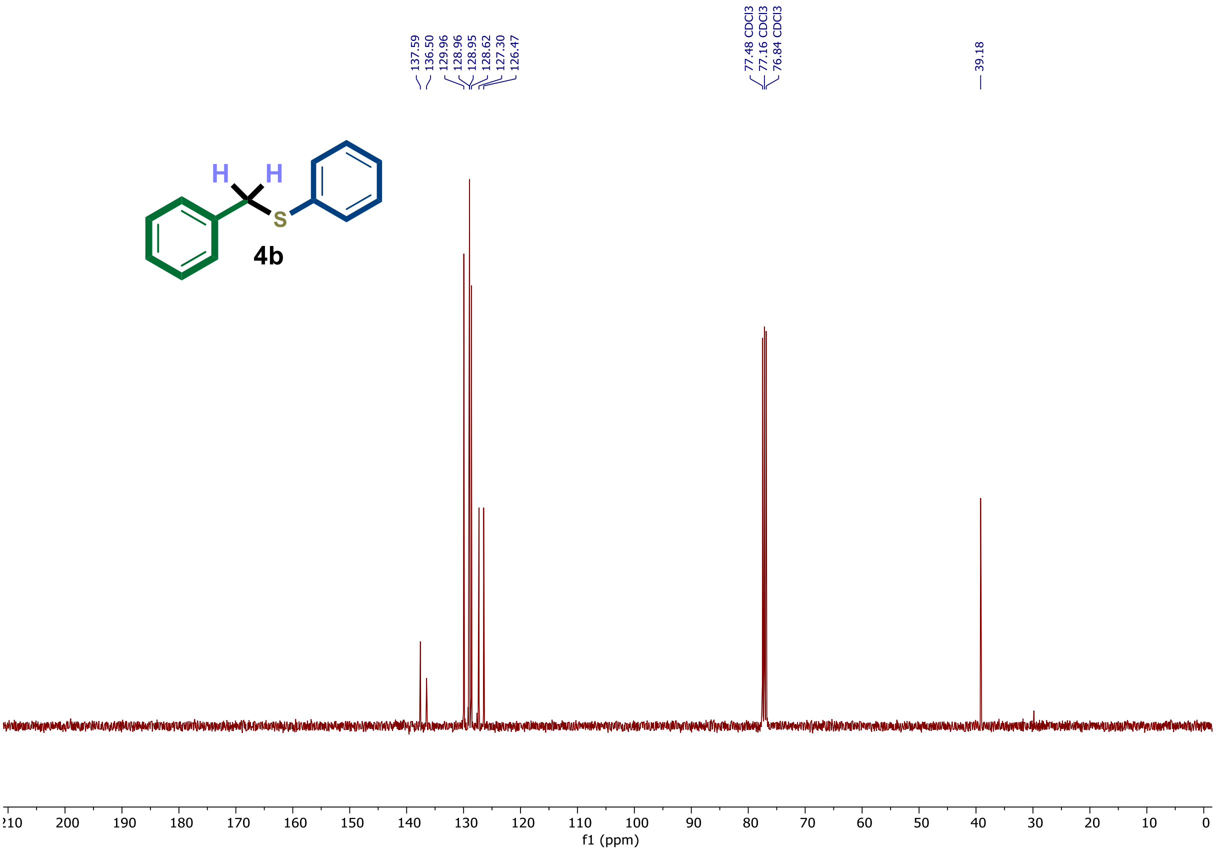

^{1}H NMR (400 MHz, CDCl3) of **4c**

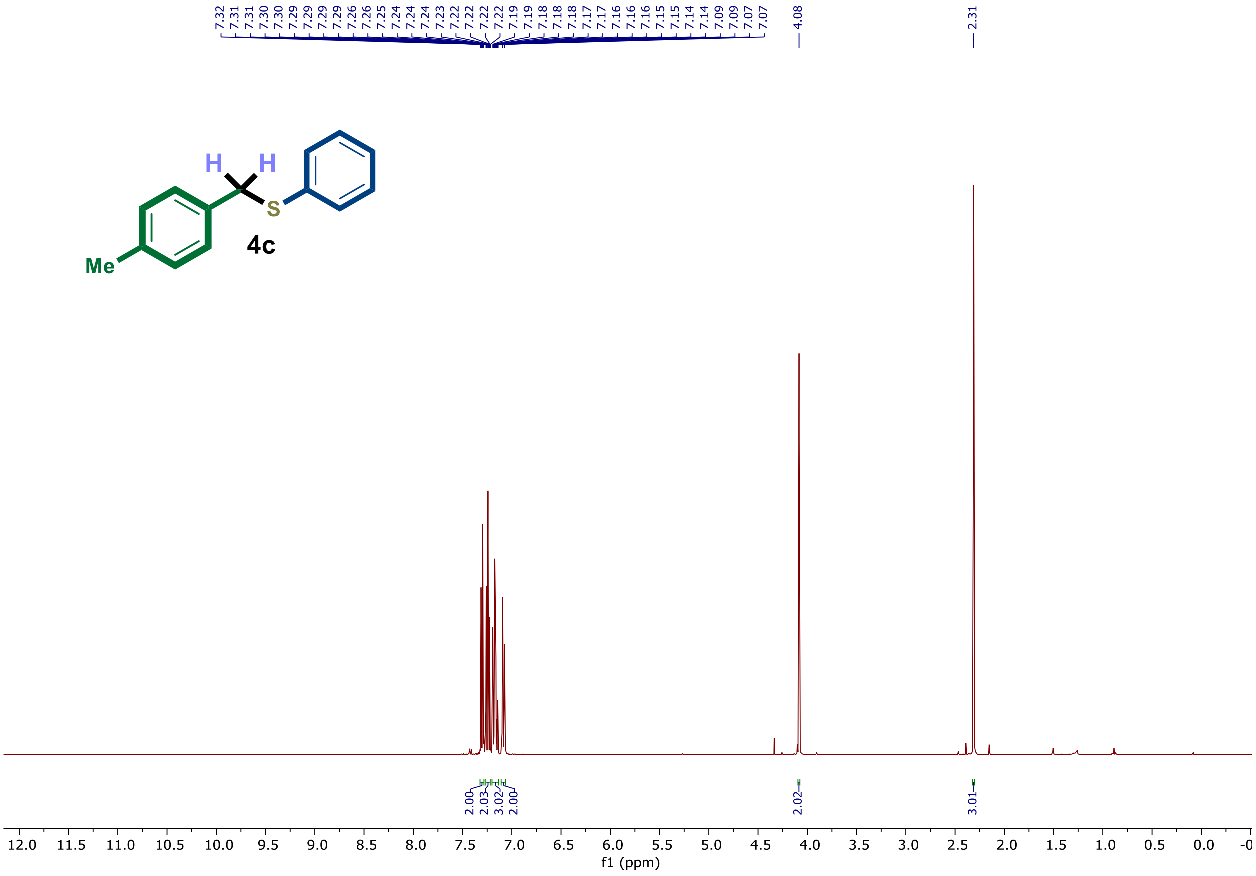

^{13}C NMR (100 MHz, CDCl$_3$) of **4c**

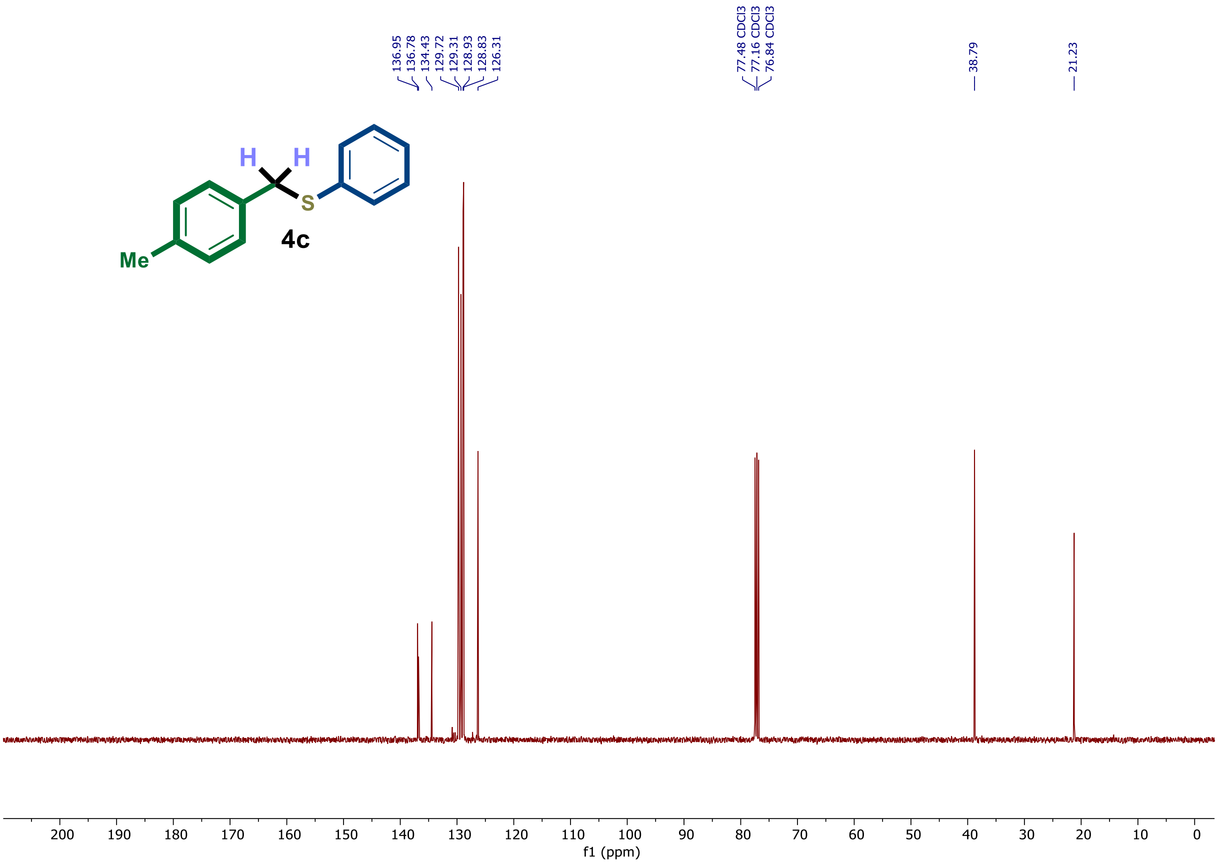

^{1}H NMR (400 MHz, CDCl$_3$) of **4d**

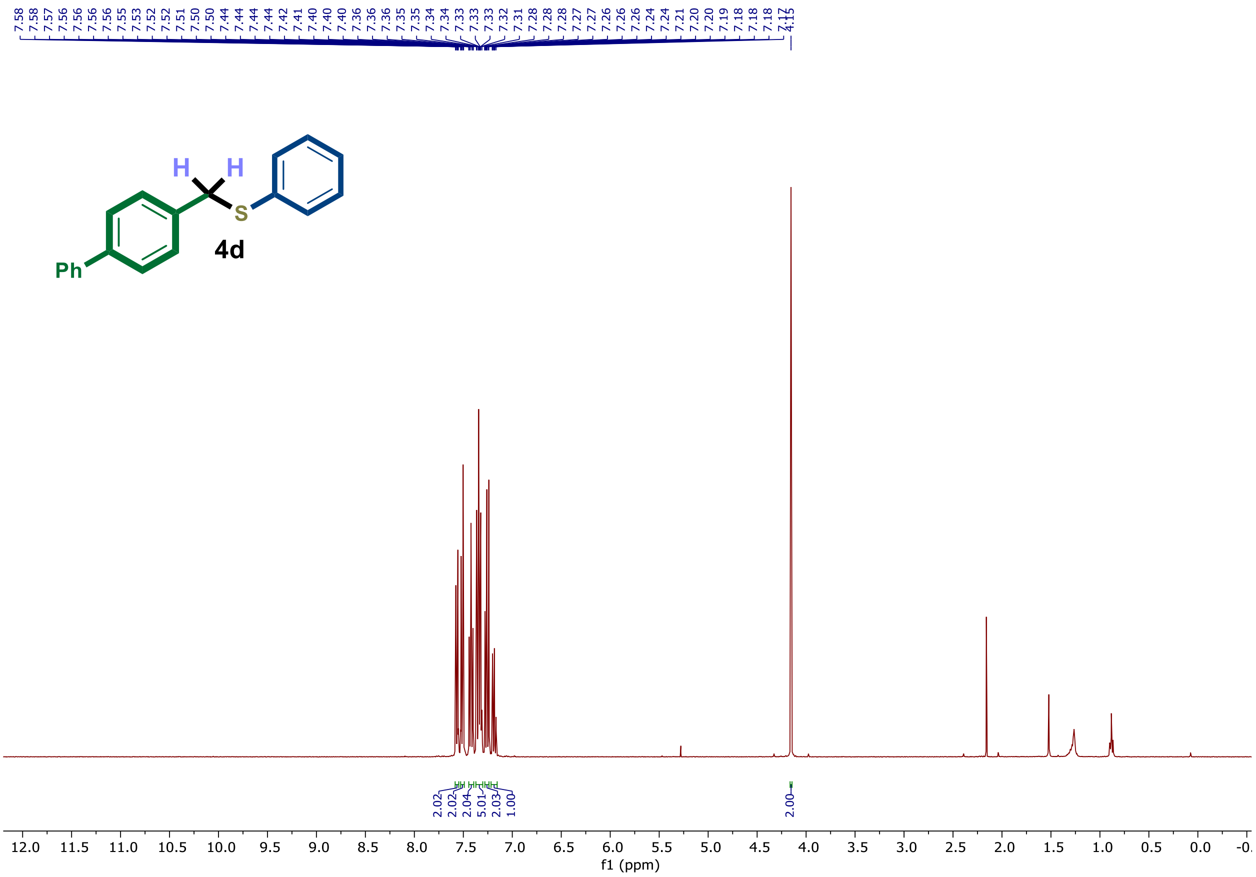

^{13}C NMR (100 MHz, $CDCl_3$) of **4d**

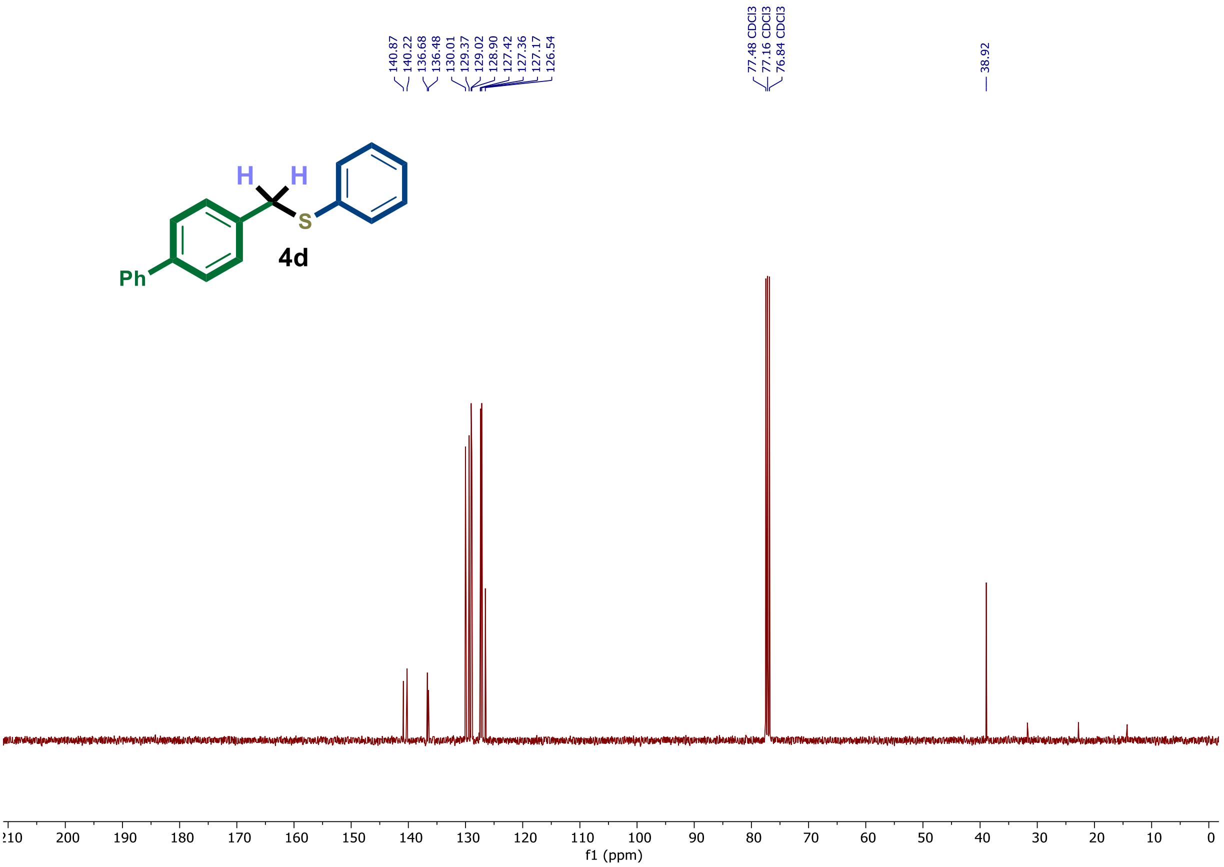

^{1}H NMR (400 MHz, $CDCl_3$) of **4e**

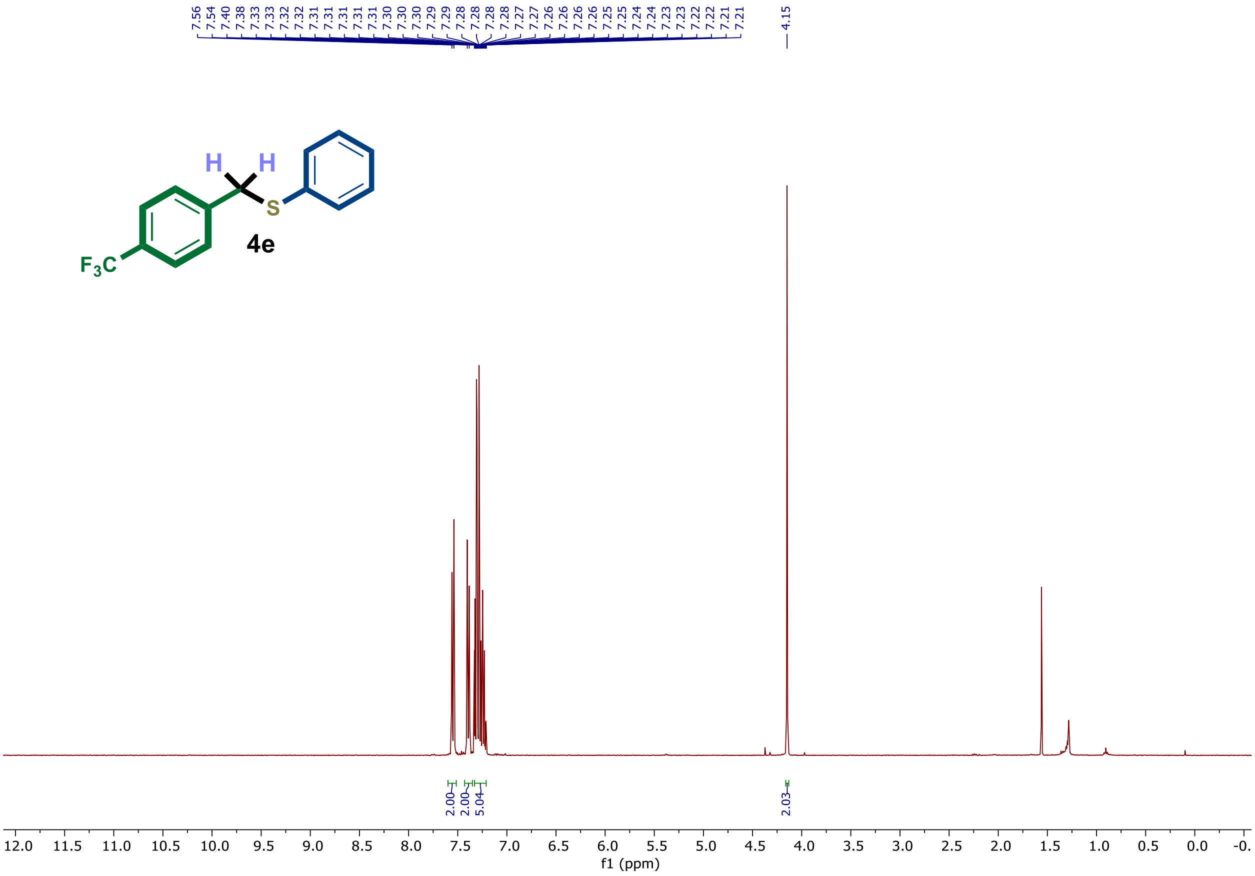

^{13}C NMR (100 MHz, $CDCl_3$) of **4e**

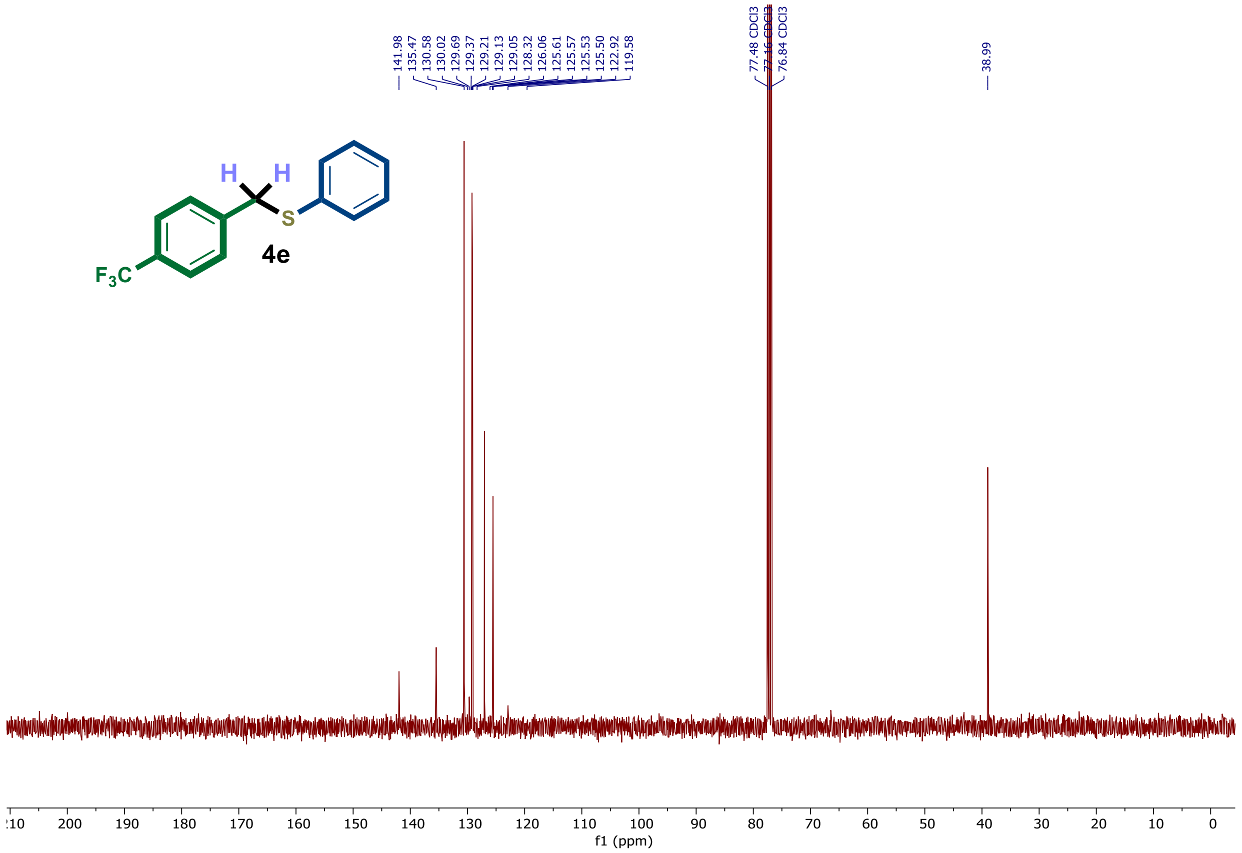

^{19}F NMR (376 MHz, $CDCl_3$) of **4e**

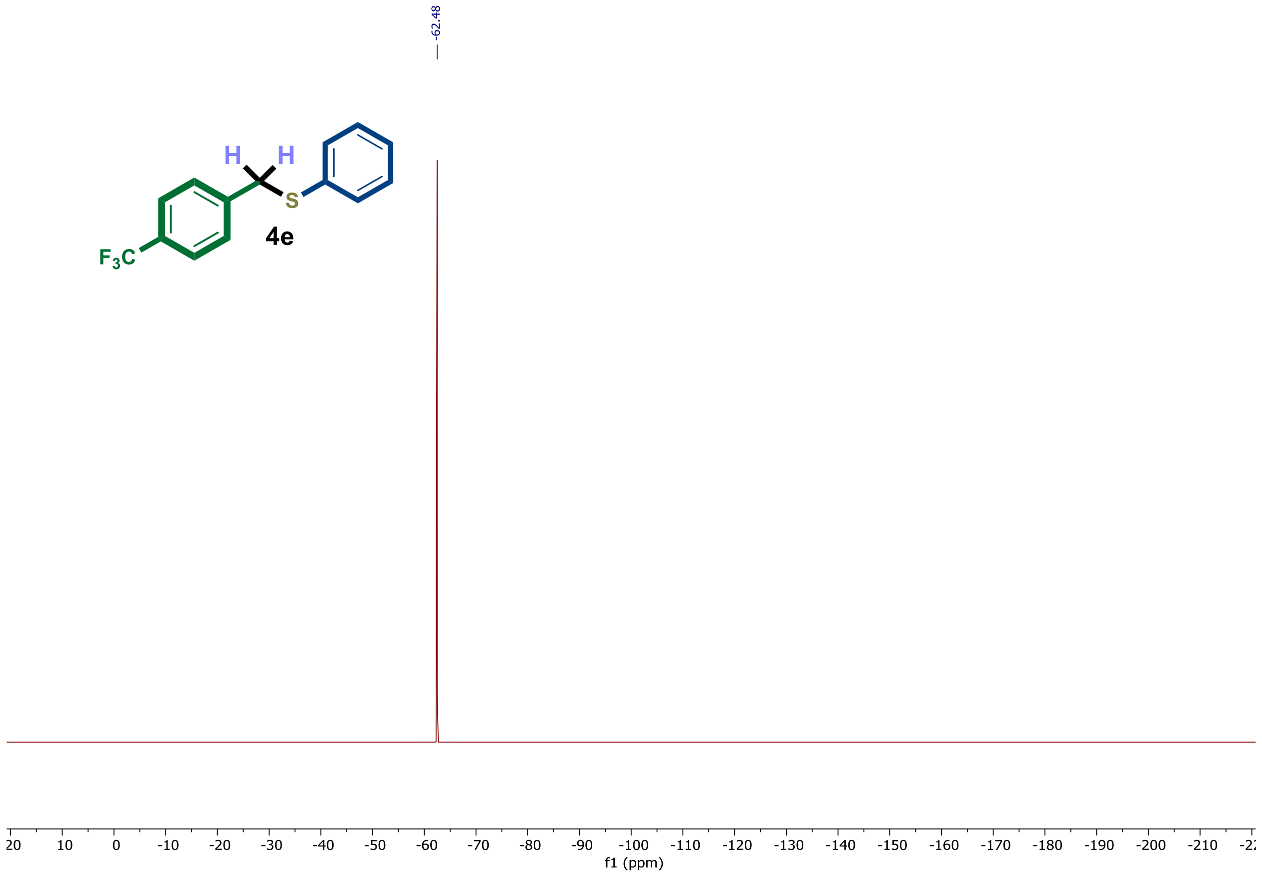

^{1}H NMR (400 MHz, $CDCl_3$) of **4f**

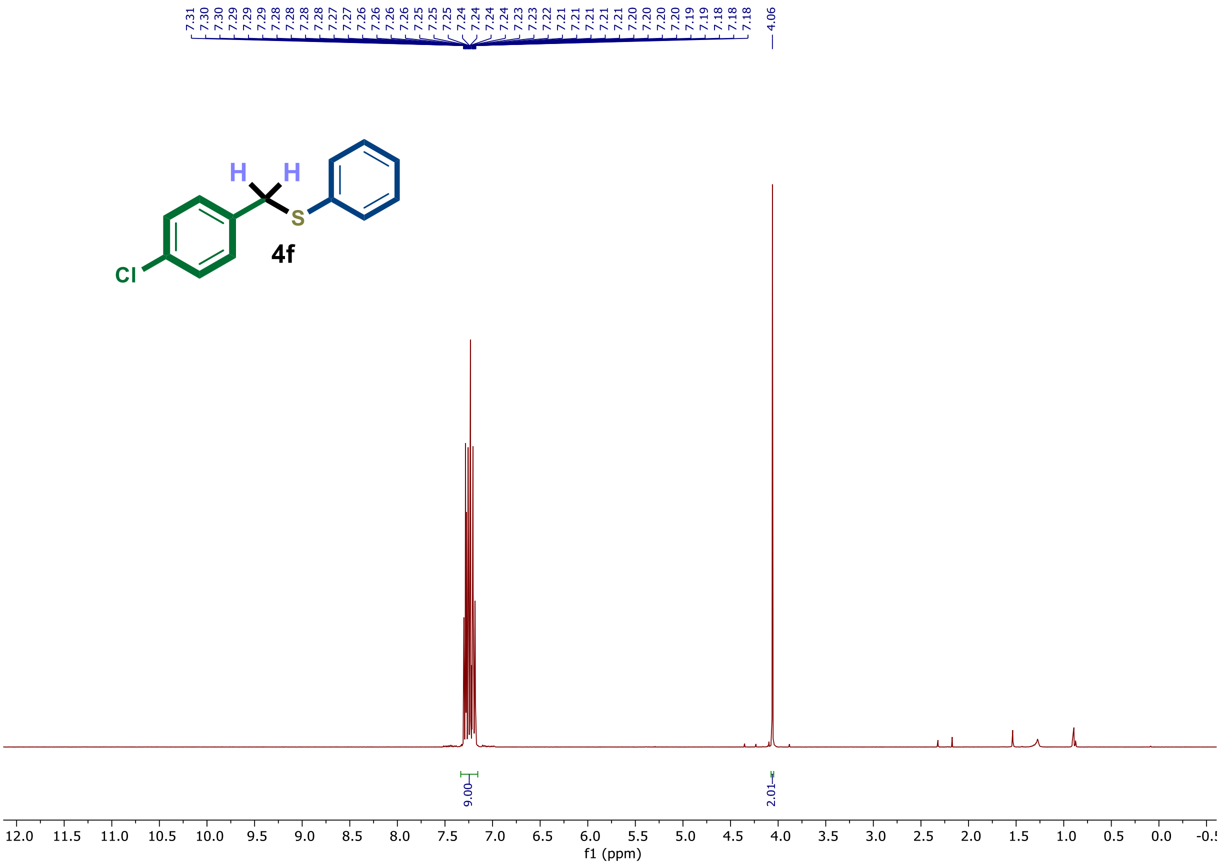

^{13}C NMR (100 MHz, $CDCl_3$) of **4f**

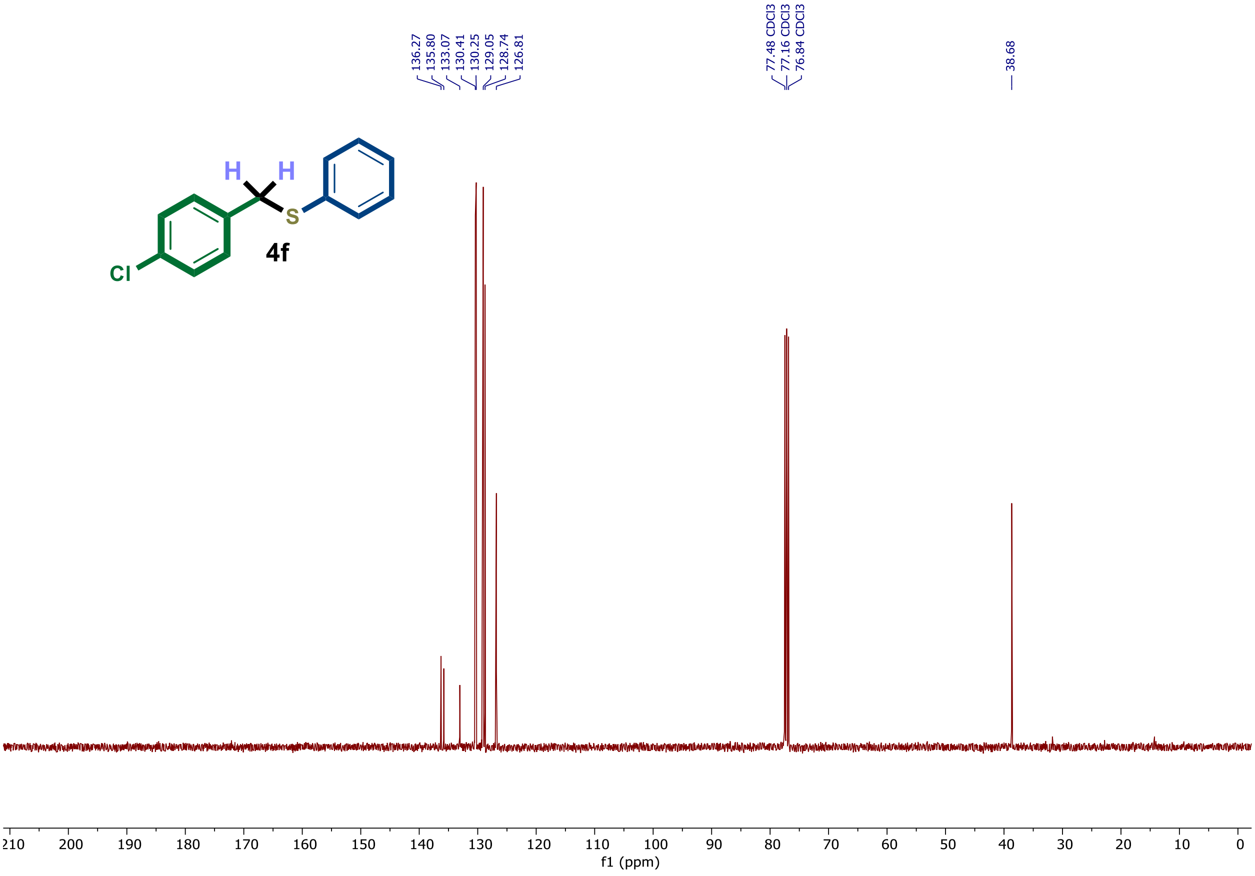

^{1}H NMR (400 MHz, $CDCl_3$) of **4g**

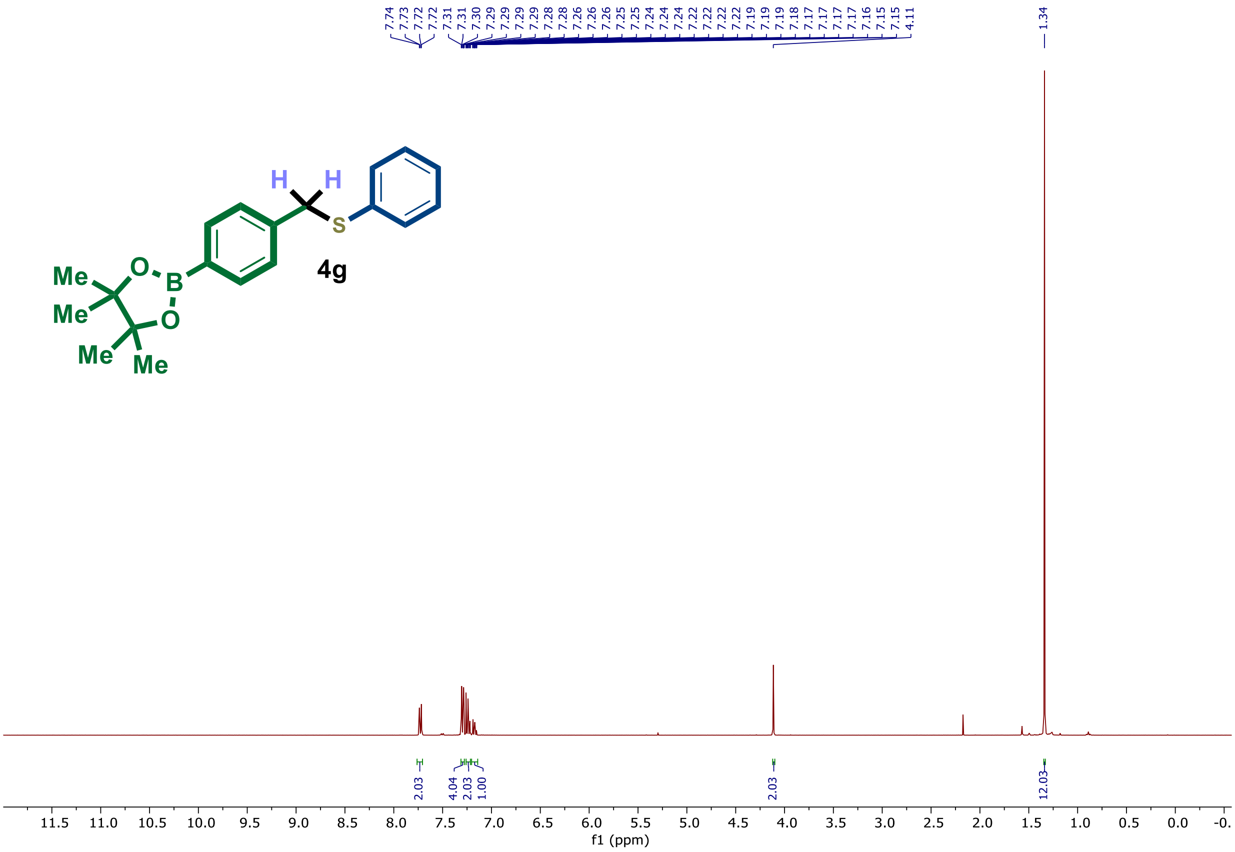

^{13}C NMR (100 MHz, $CDCl_3$) of **4g**

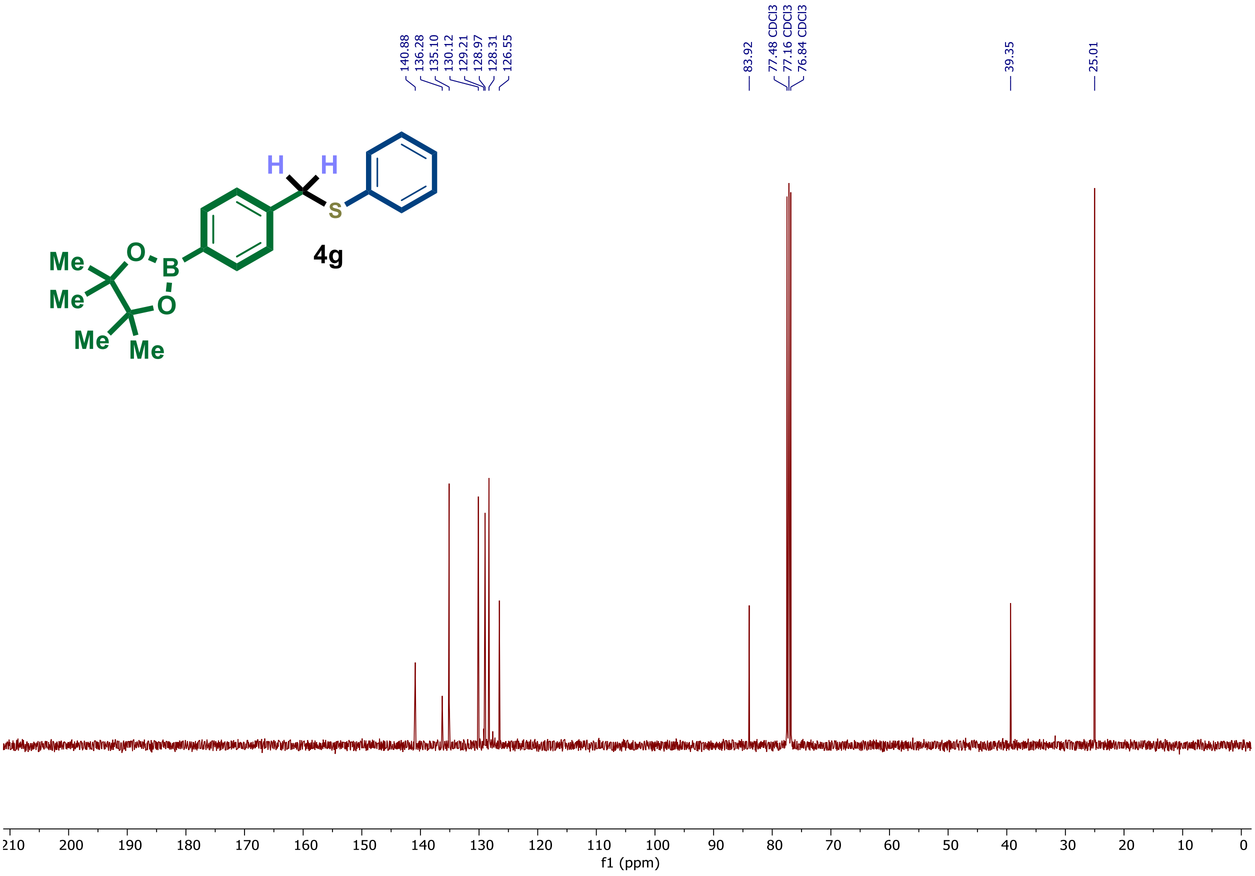

^{11}B NMR (128 MHz, $CDCl_3$) of **4g**

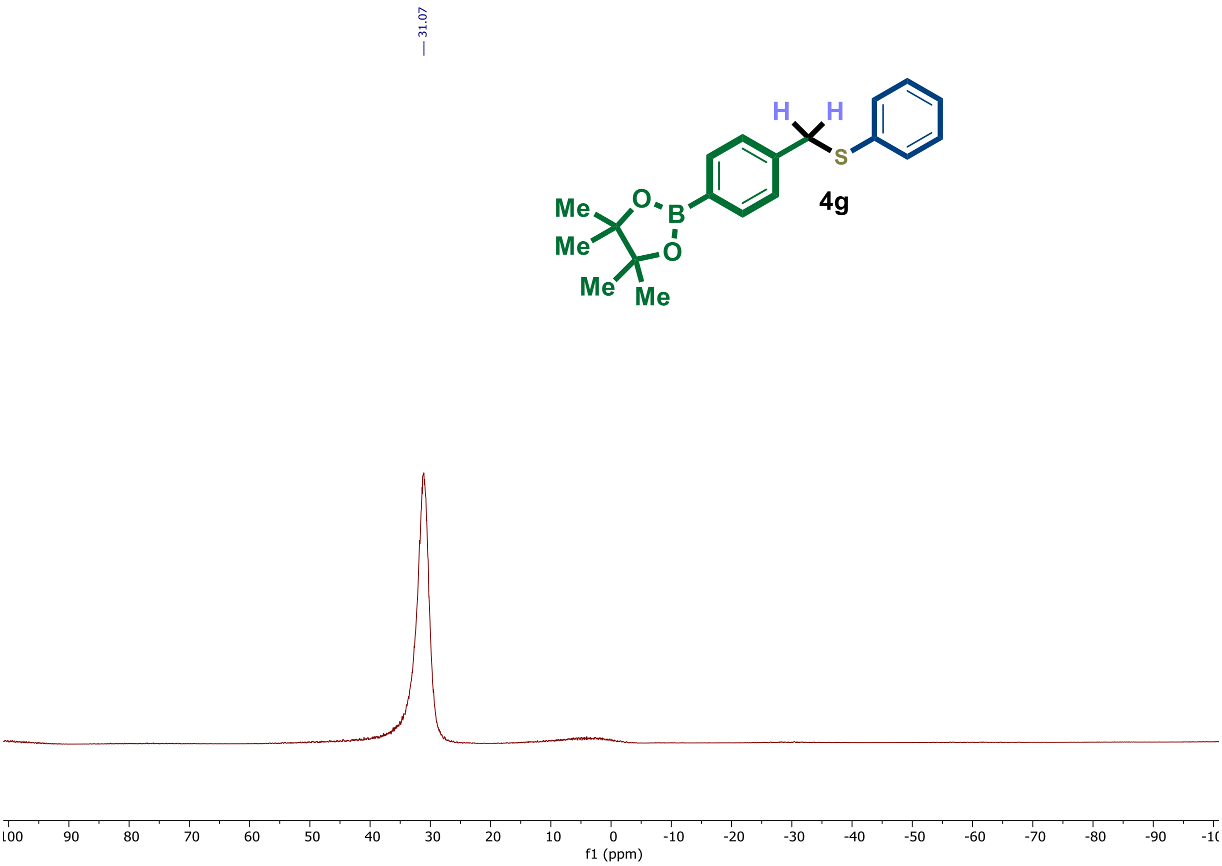

^{1}H NMR (400 MHz, $CDCl_3$) of **4i**

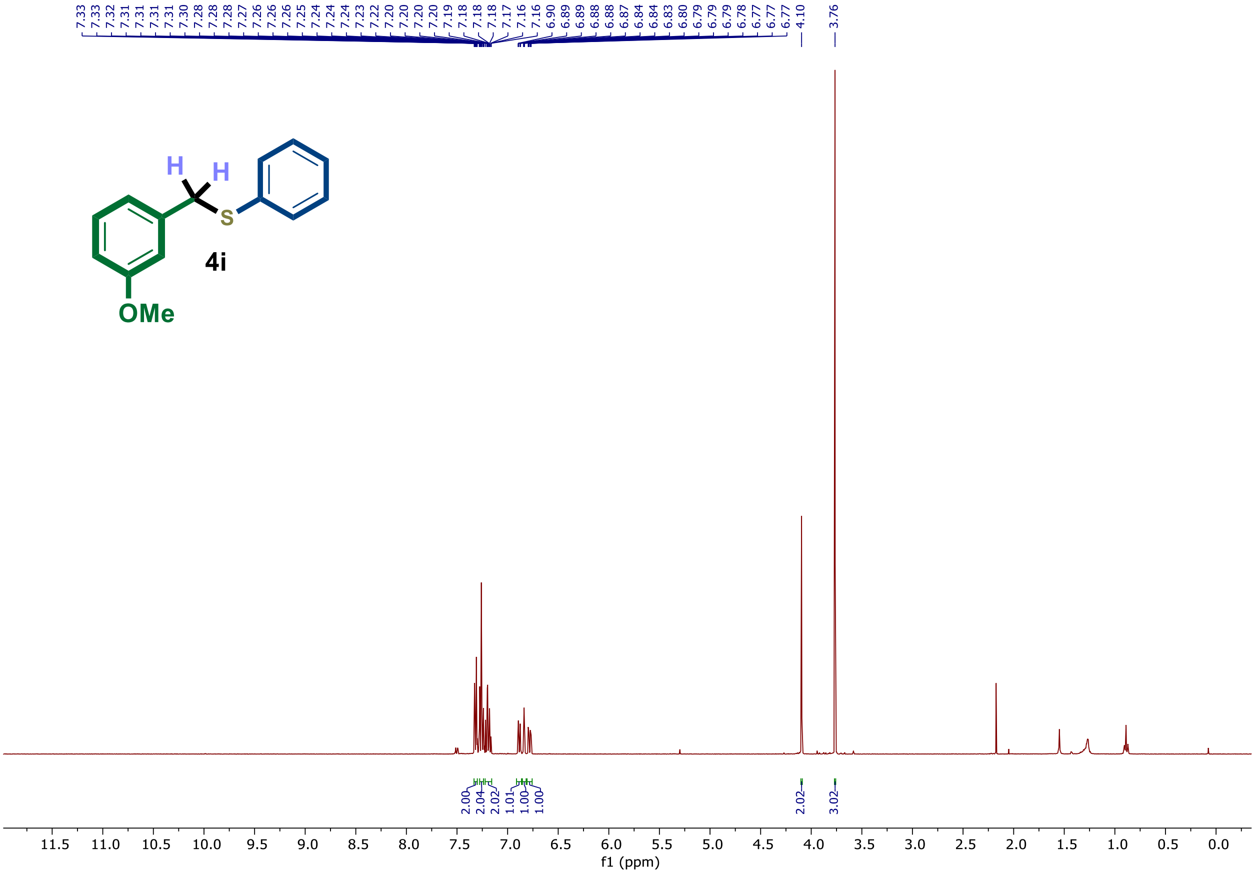

^{13}C NMR (100 MHz, CDCl$_3$) of **4i**

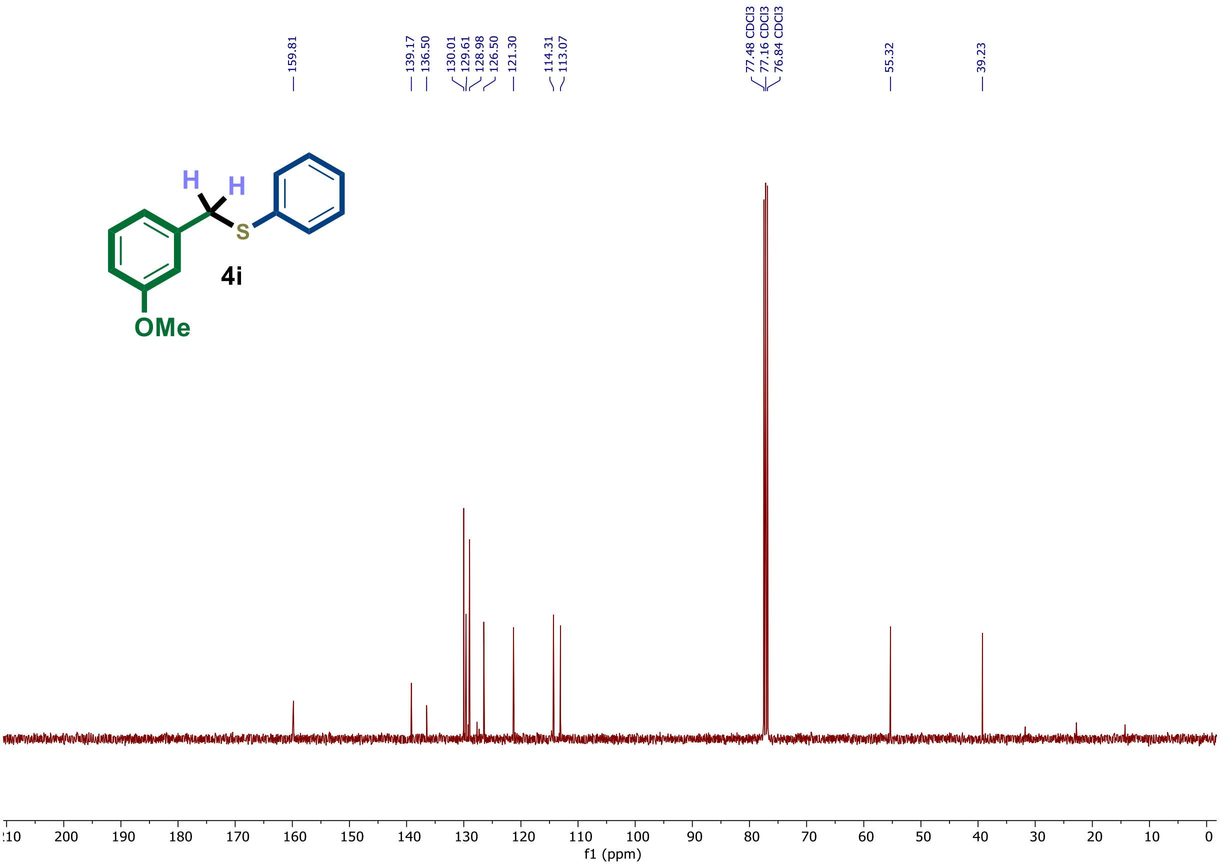

^{1}H NMR (400 MHz, CDCl$_3$) of **4j**

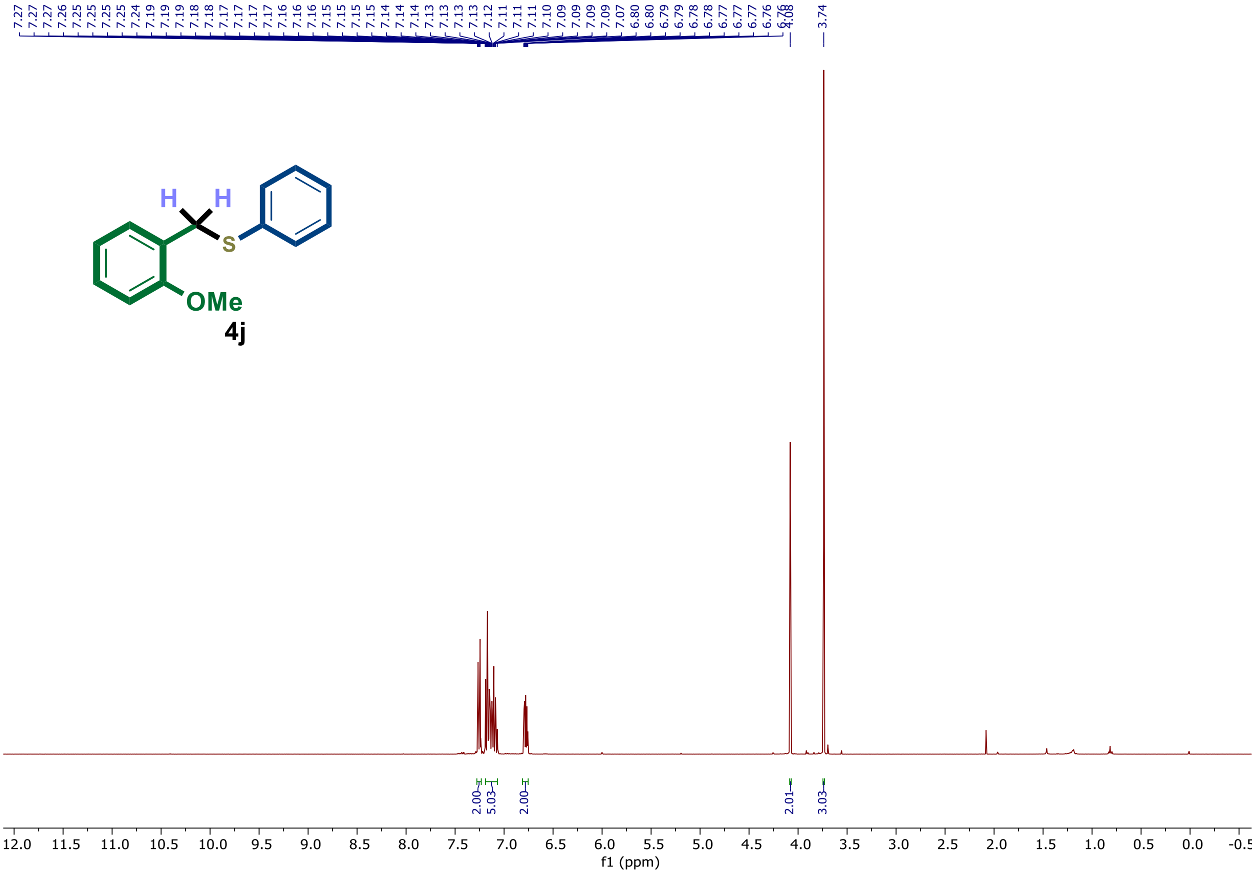

^{13}C NMR (100 MHz, CDCl$_3$) of **4j**

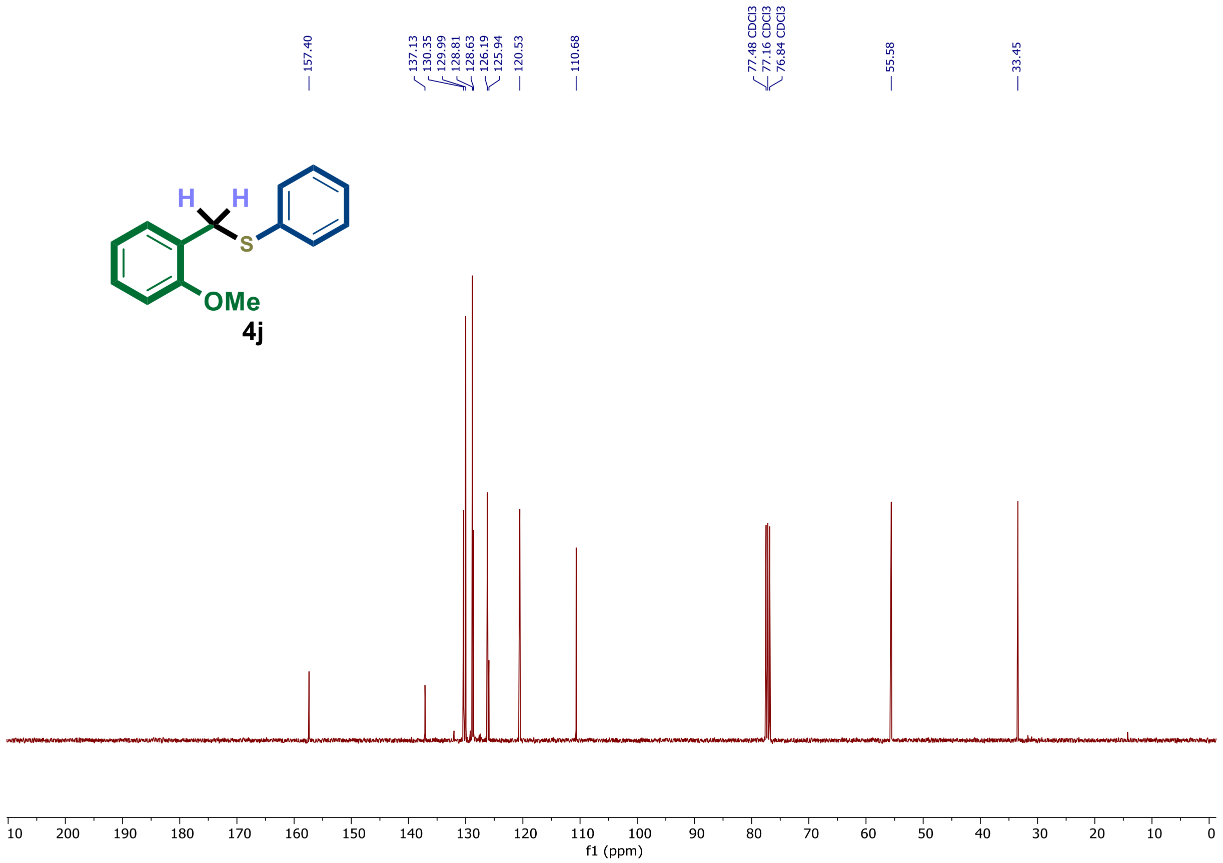

^{1}H NMR (400 MHz, CDCl$_3$) of **4k**

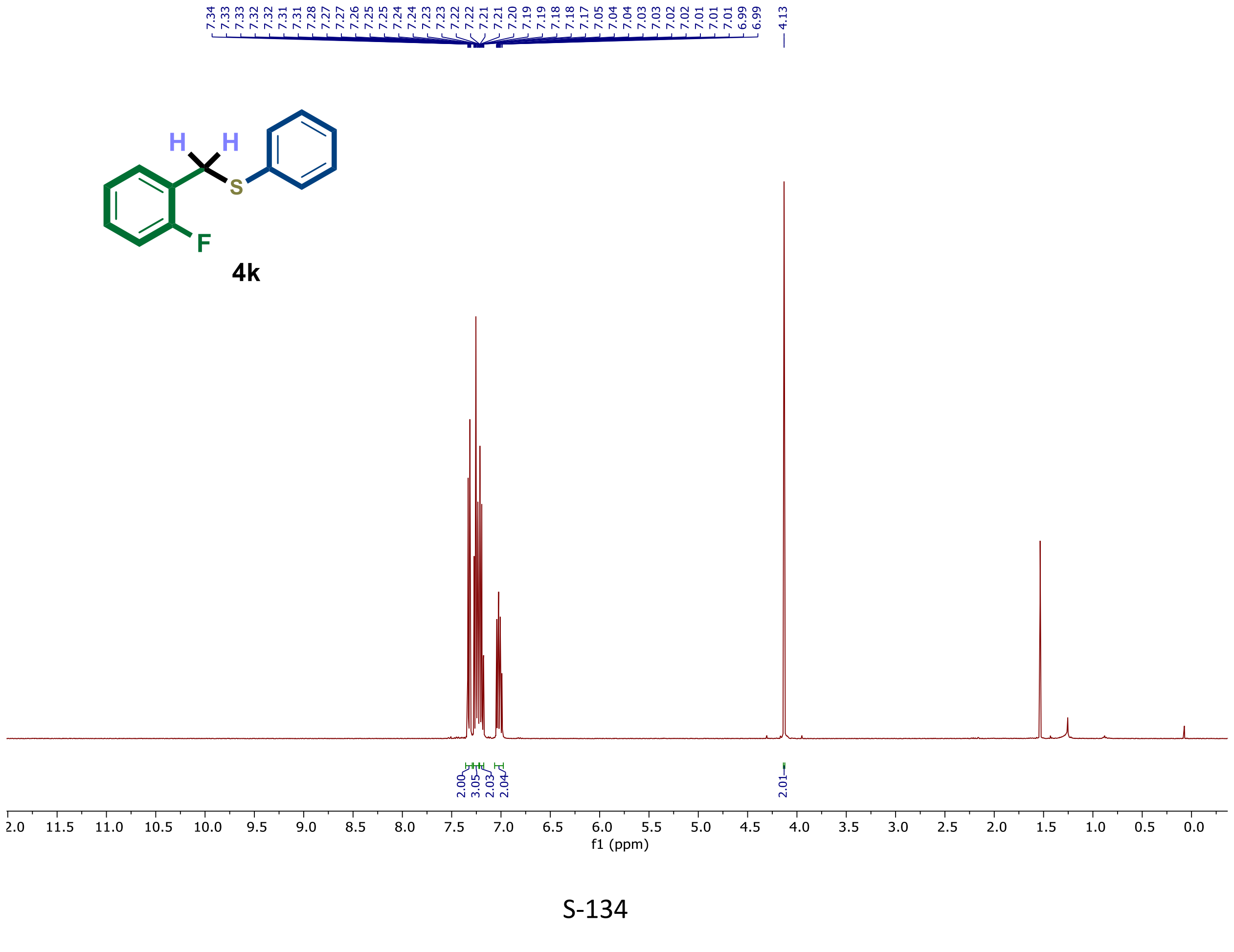

^{13}C NMR (100 MHz, $CDCl_3$) of **4k**

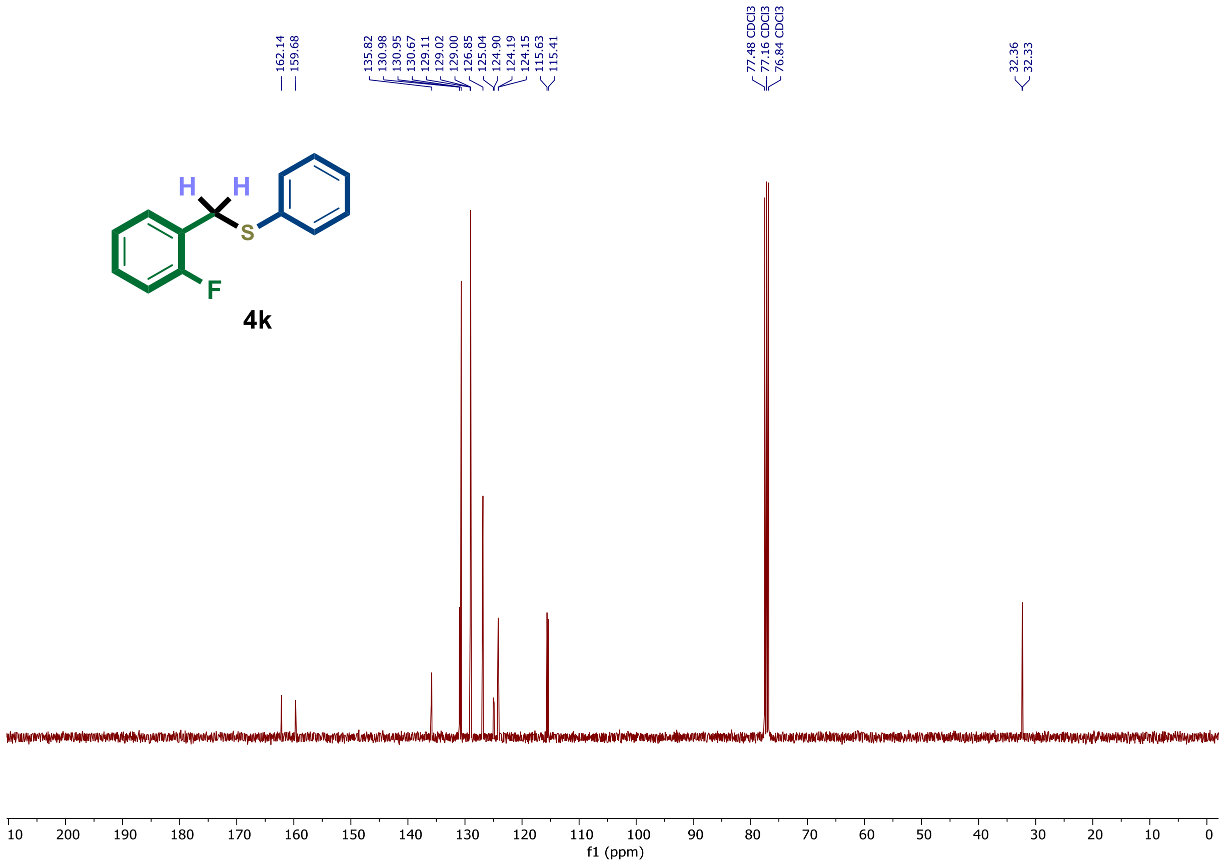

^{19}F NMR (376 MHz, $CDCl_3$) of **4k**

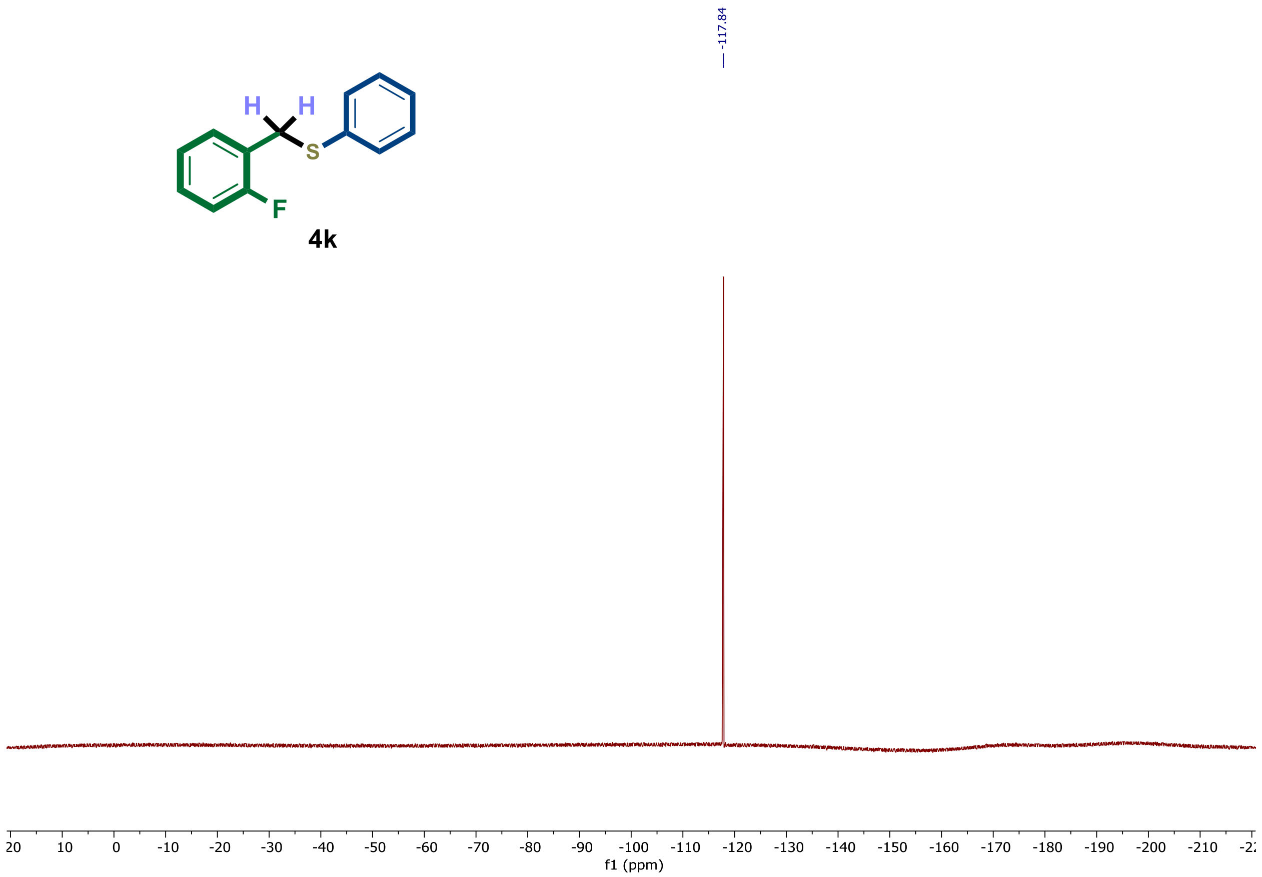

^{1}H NMR (400 MHz, $CDCl_3$) of **4m**

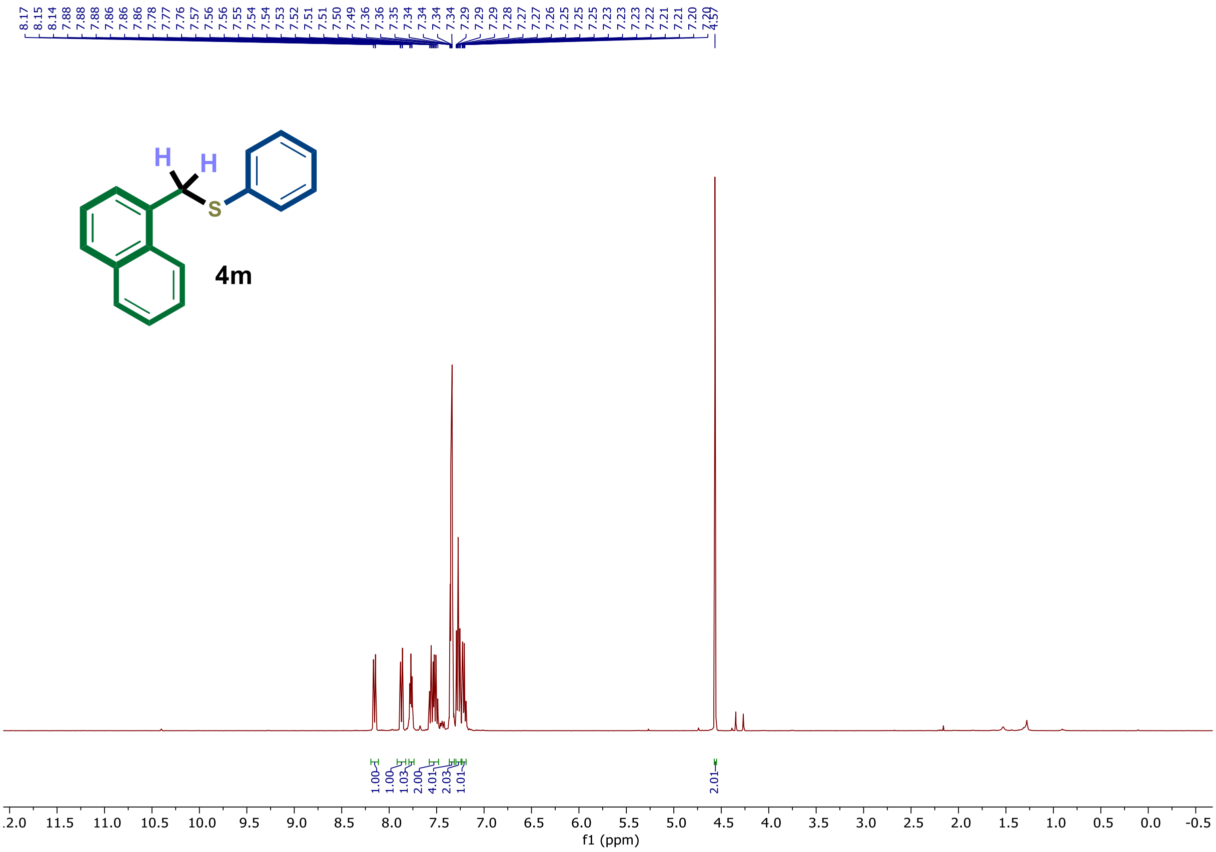

^{13}C NMR (100 MHz, $CDCl_3$) of **4m**

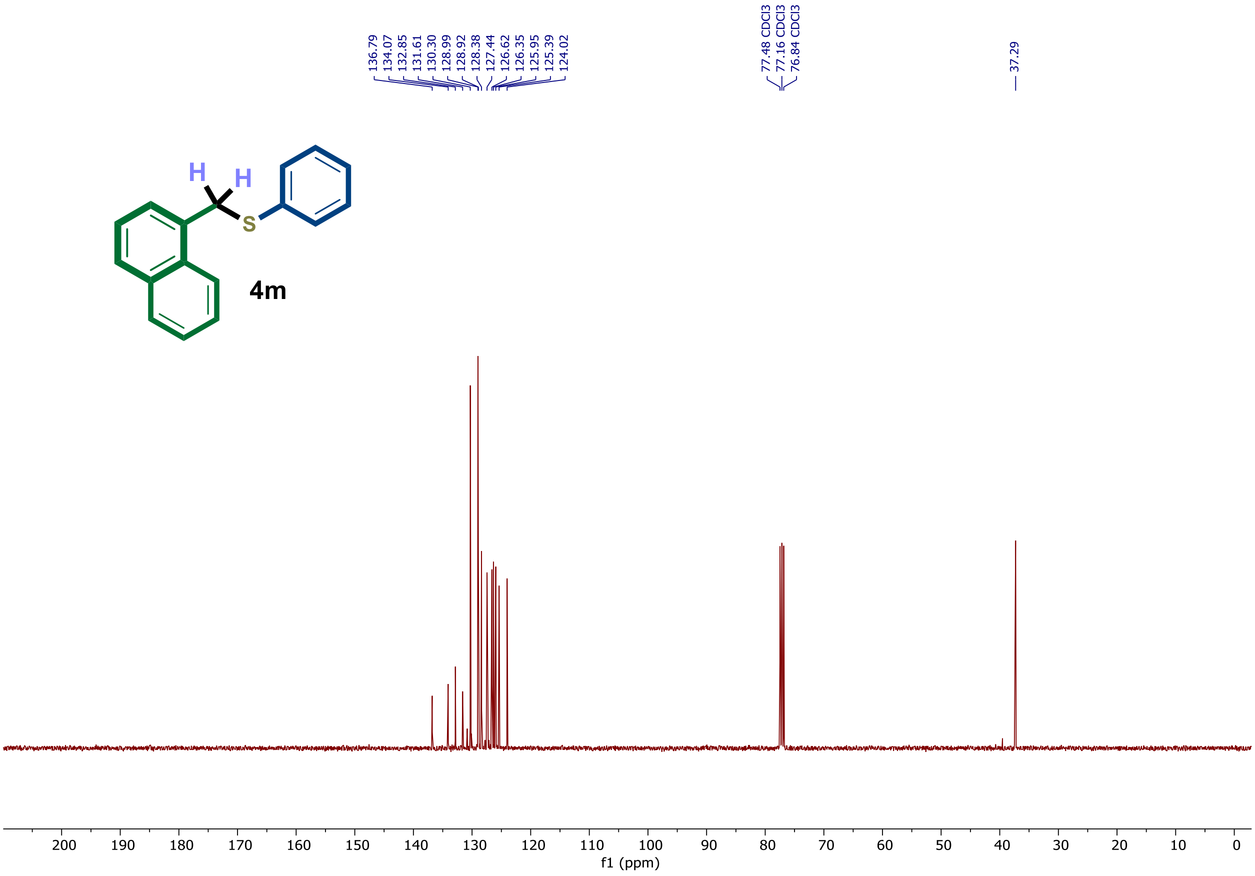

^{1}H NMR (400 MHz, CDCl$_3$) of **4n**

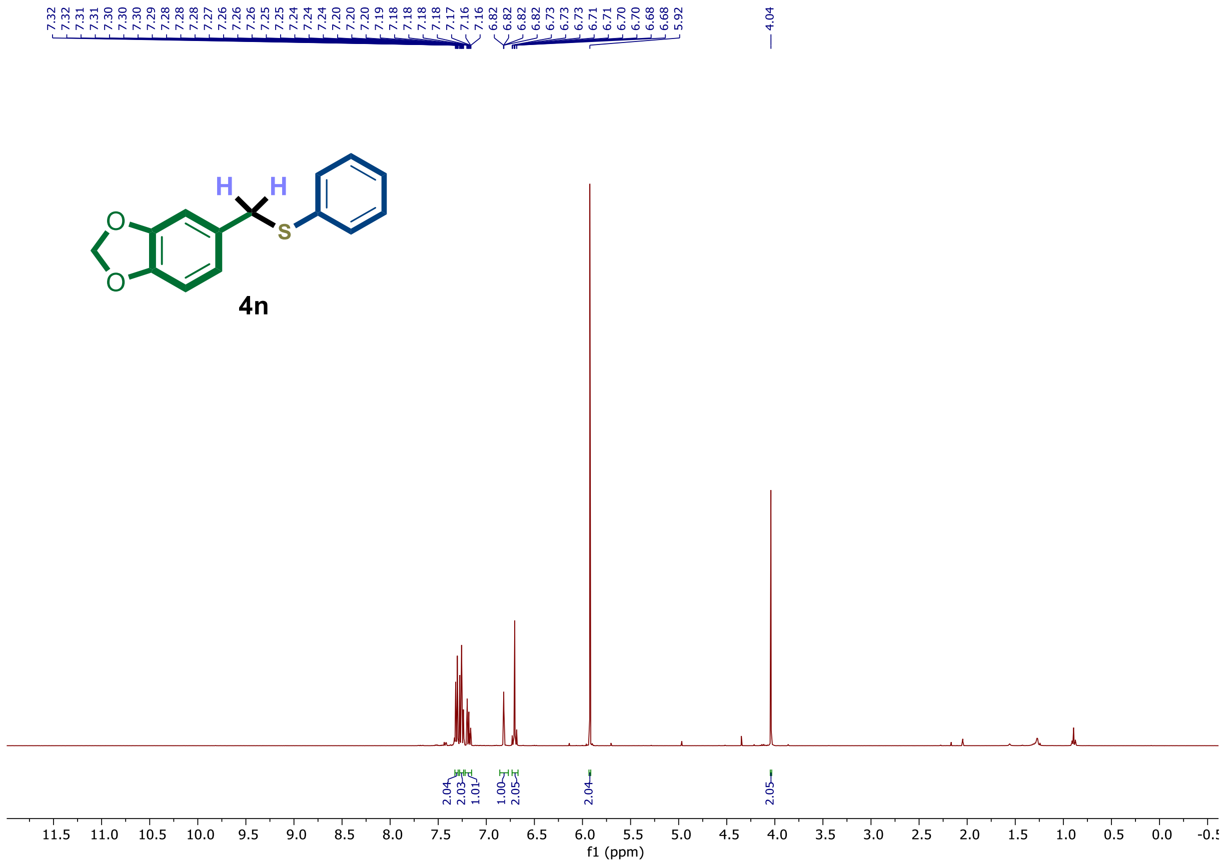

^{13}C NMR (100 MHz, CDCl$_3$) of **4n**

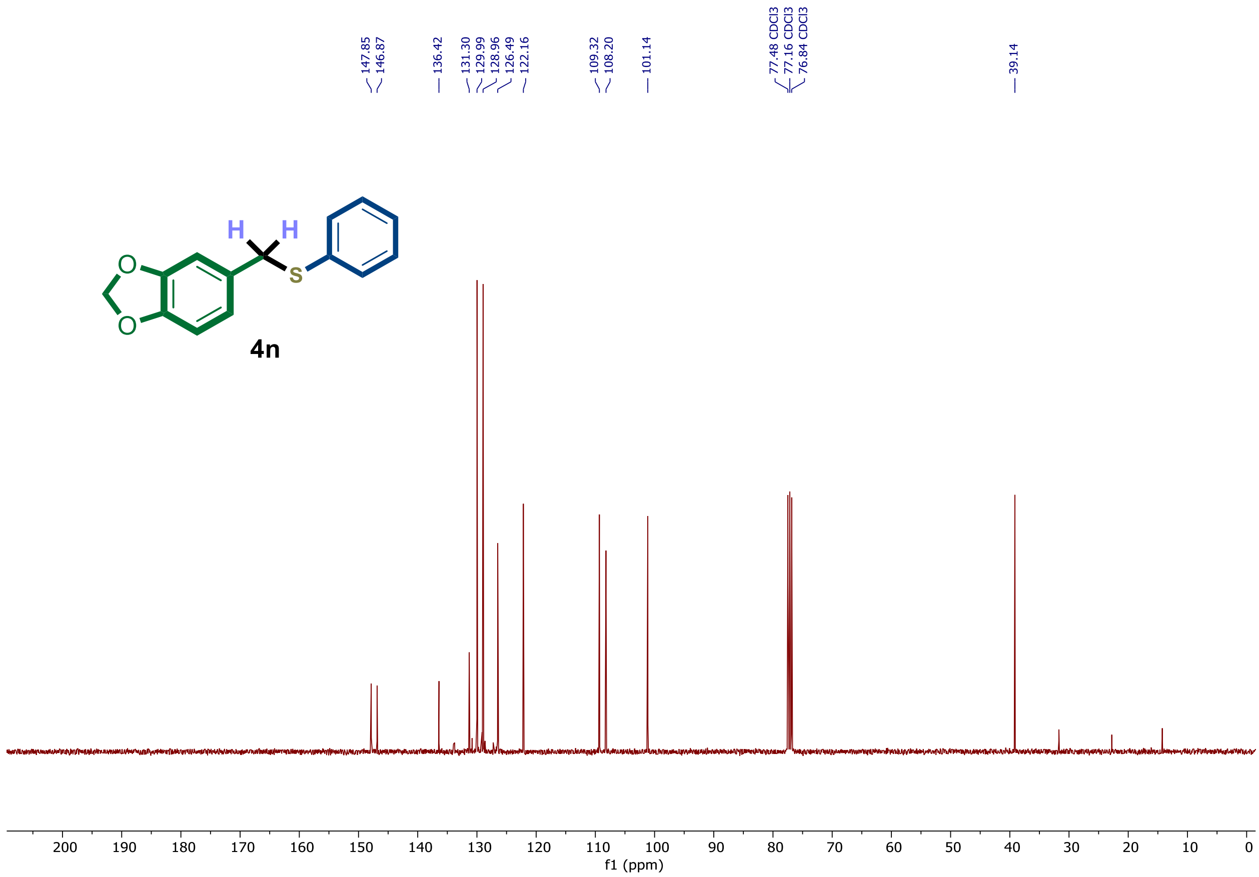

^{1}H NMR (400 MHz, $CDCl_3$) of **4o**

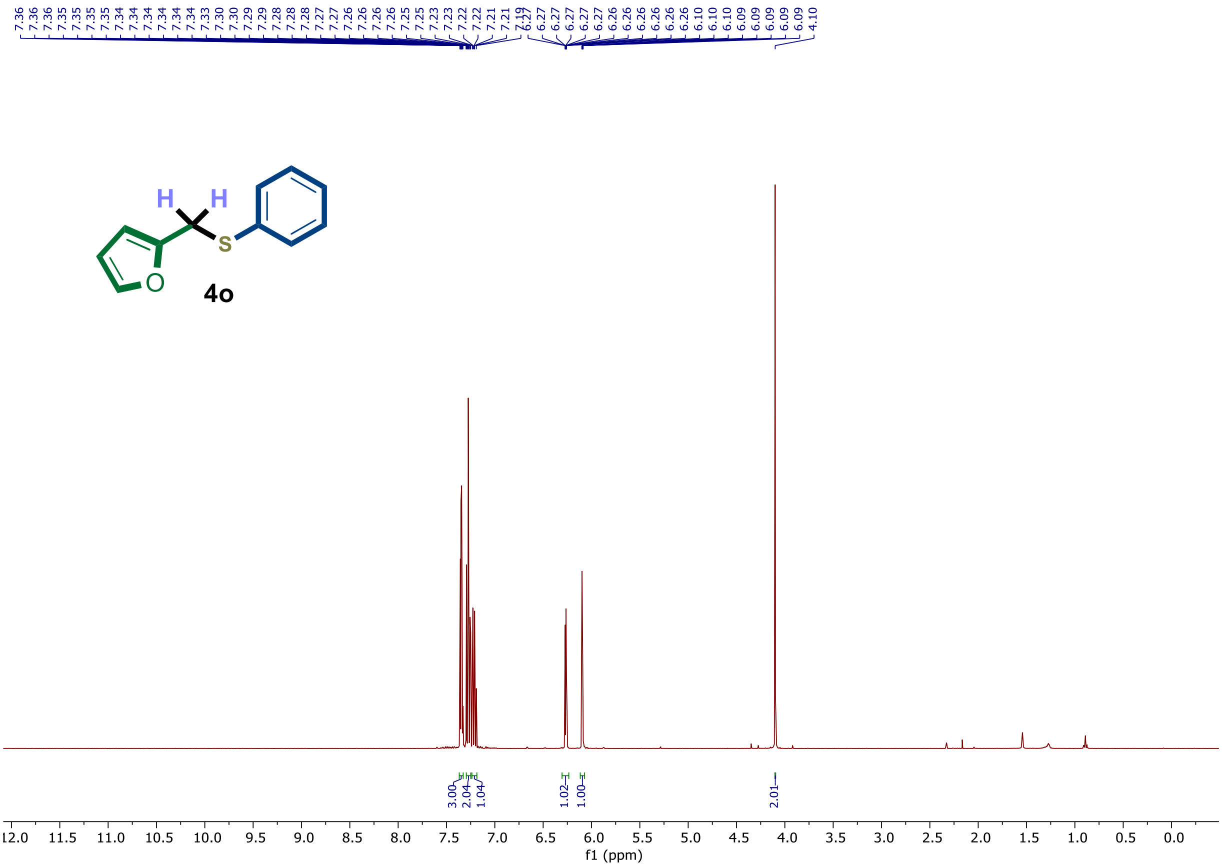

^{13}C NMR (100 MHz, $CDCl_3$) of **4o**

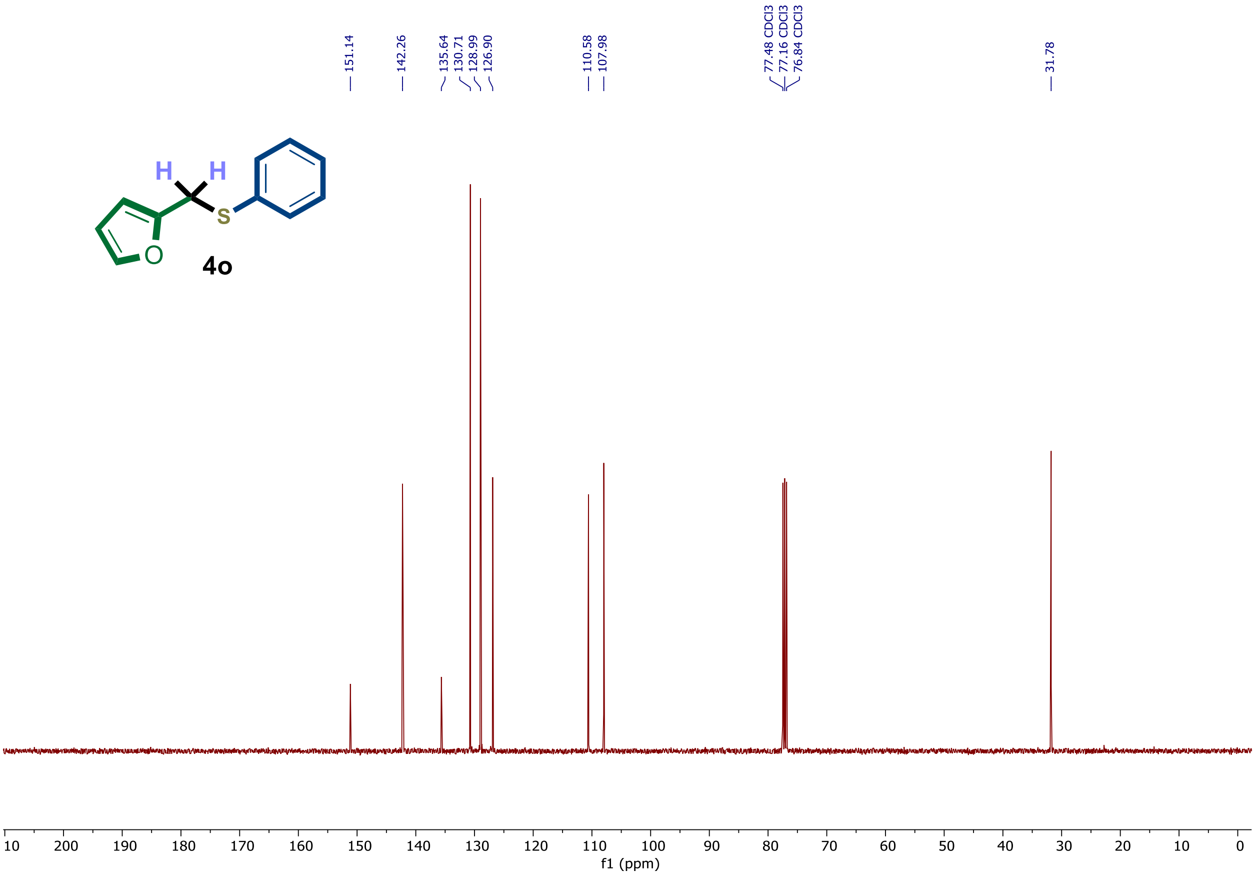

^{1}H NMR (400 MHz, $CDCl_3$) of **4q**

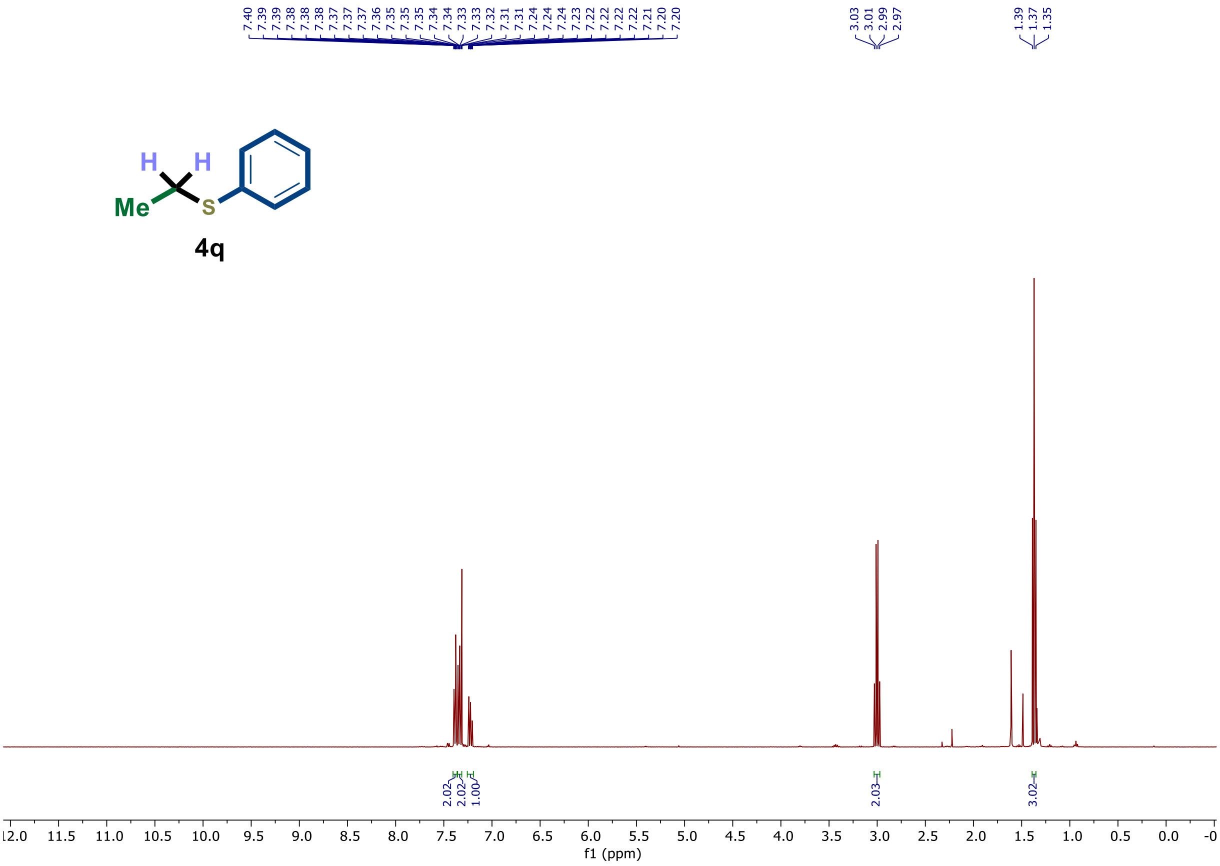

^{13}C NMR (100 MHz, $CDCl_3$) of **4q**

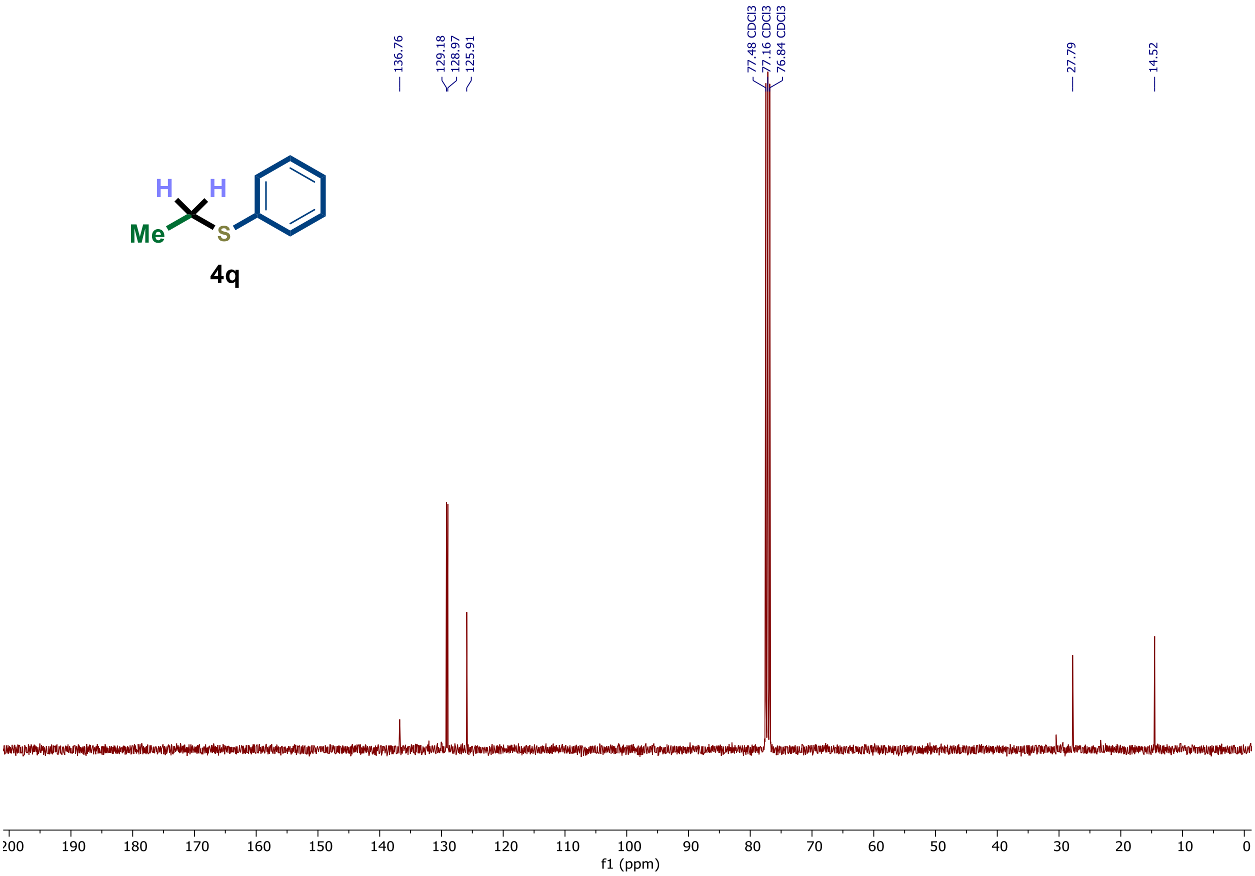

^{1}H NMR (400 MHz, $CDCl_3$) of **4r**

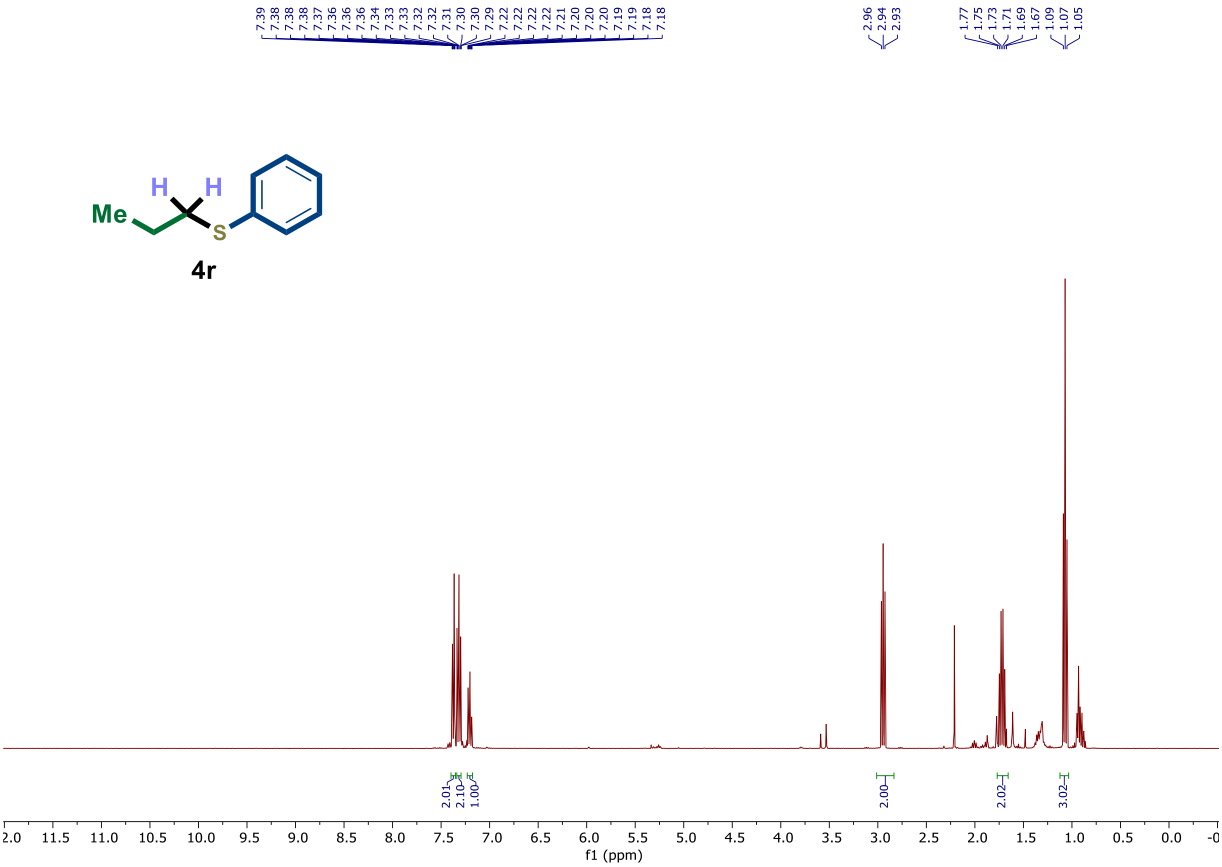

^{13}C NMR (100 MHz, $CDCl_3$) of **4r**

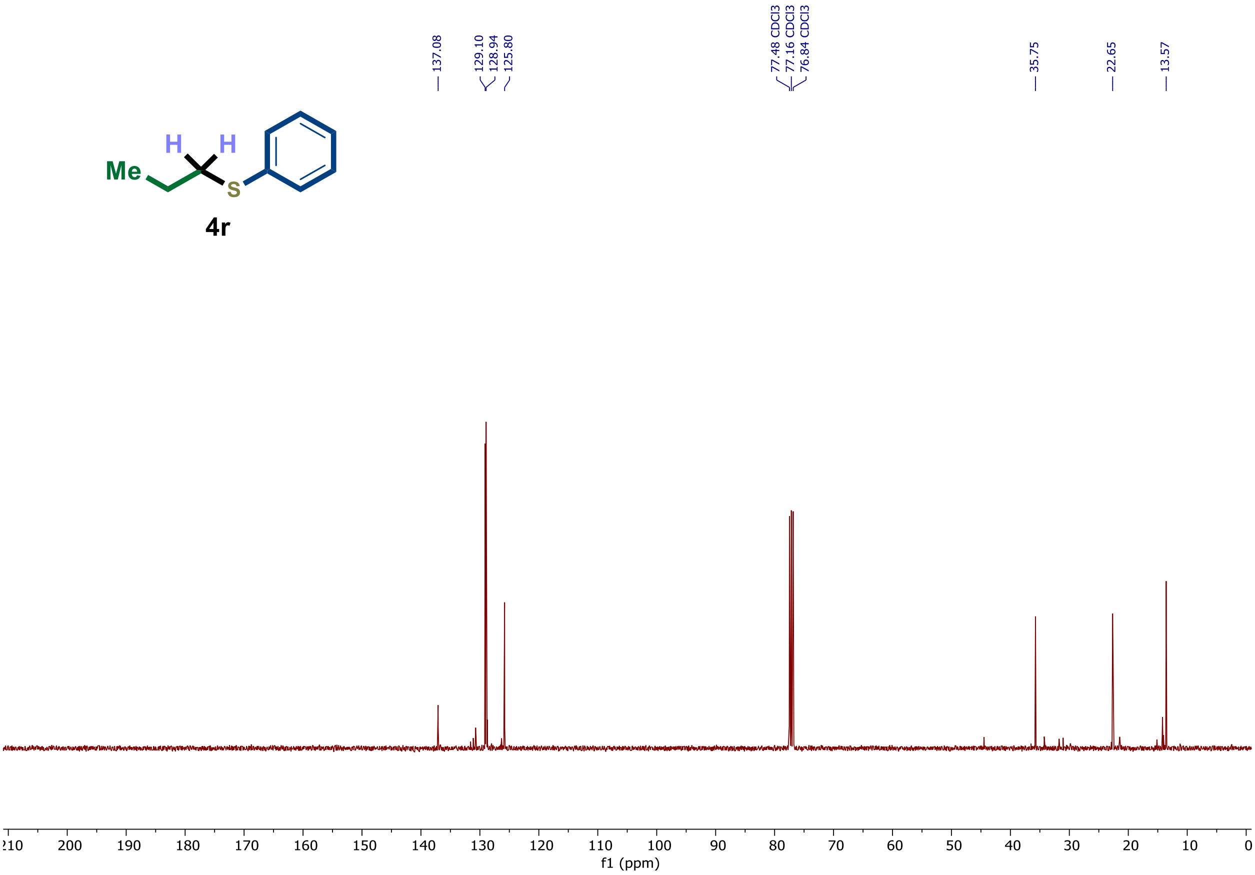

^{1}H NMR (400 MHz, $CDCl_3$) of **4s**

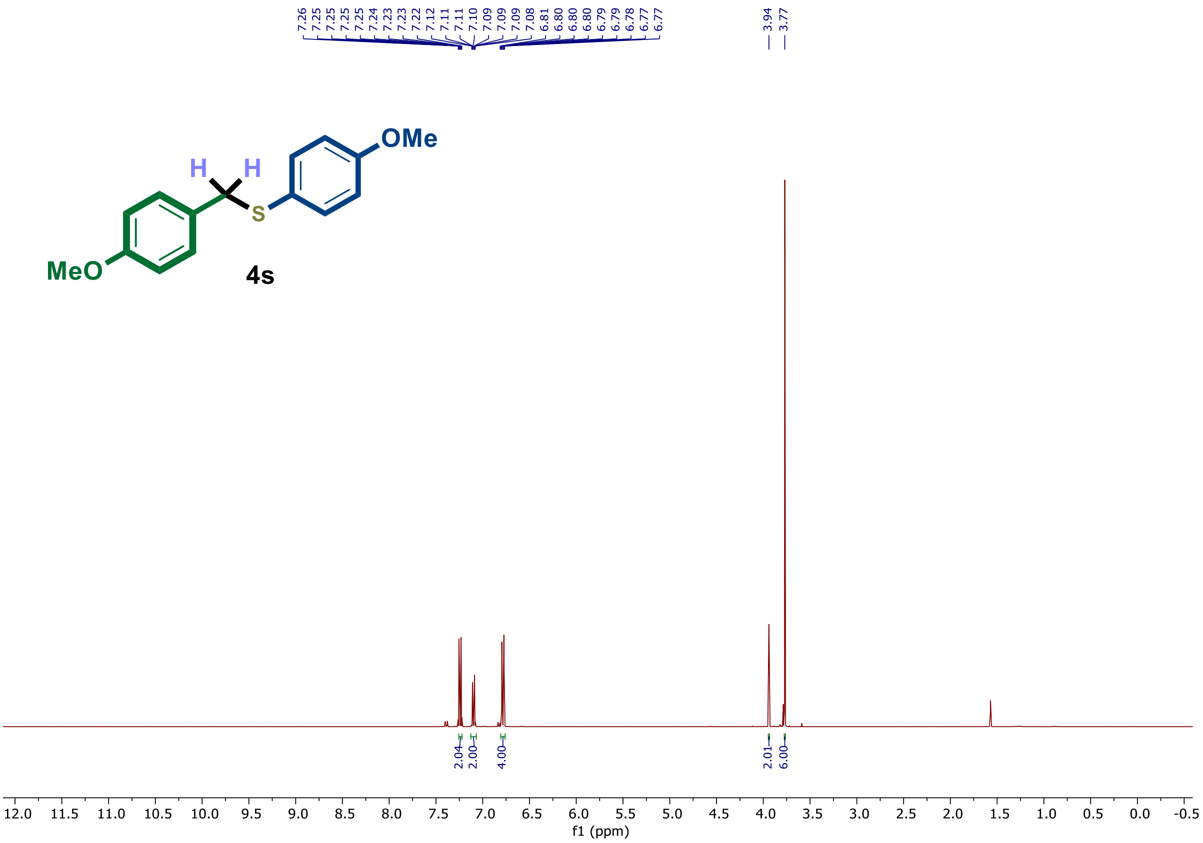

^{13}C NMR (100 MHz, $CDCl_3$) of **4s**

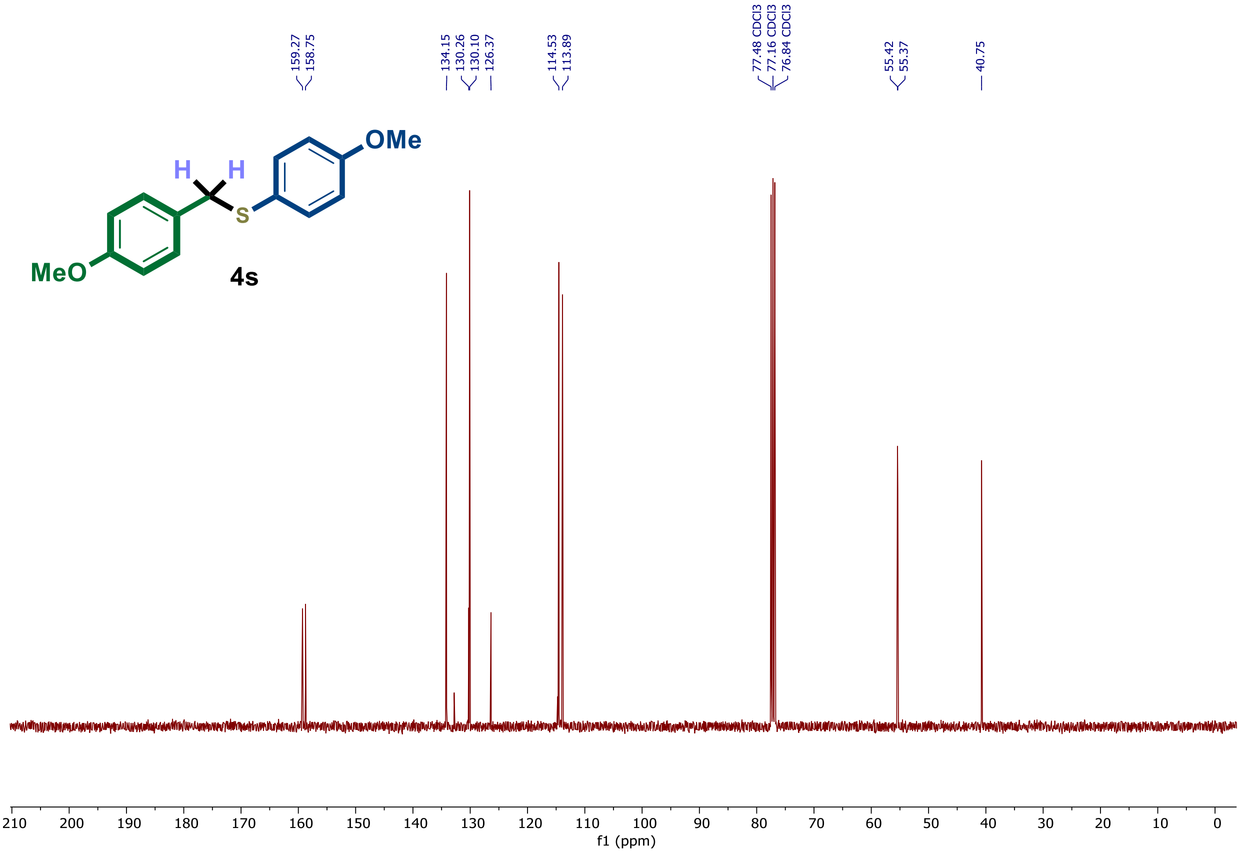

^{1}H NMR (400 MHz, $CDCl_3$) of **4t**

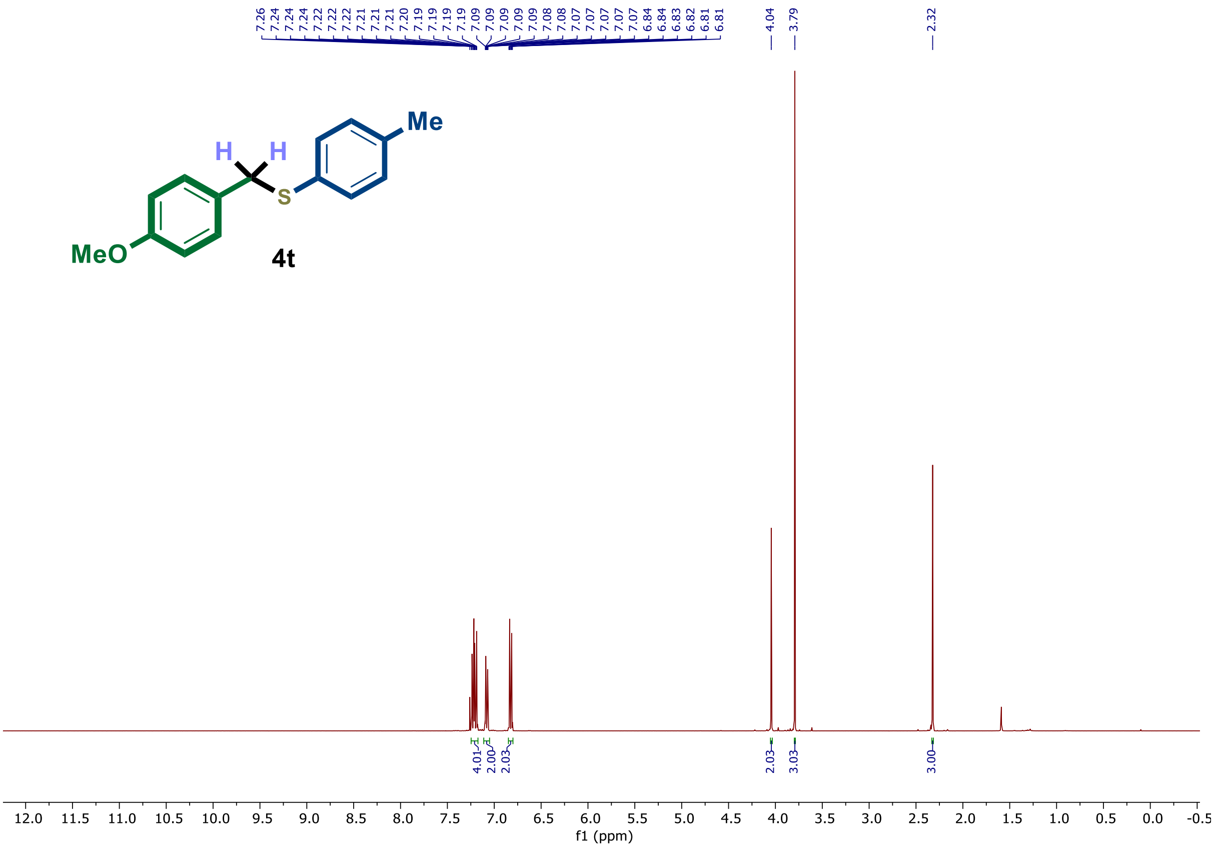

^{13}C NMR (100 MHz, $CDCl_3$) of **4t**

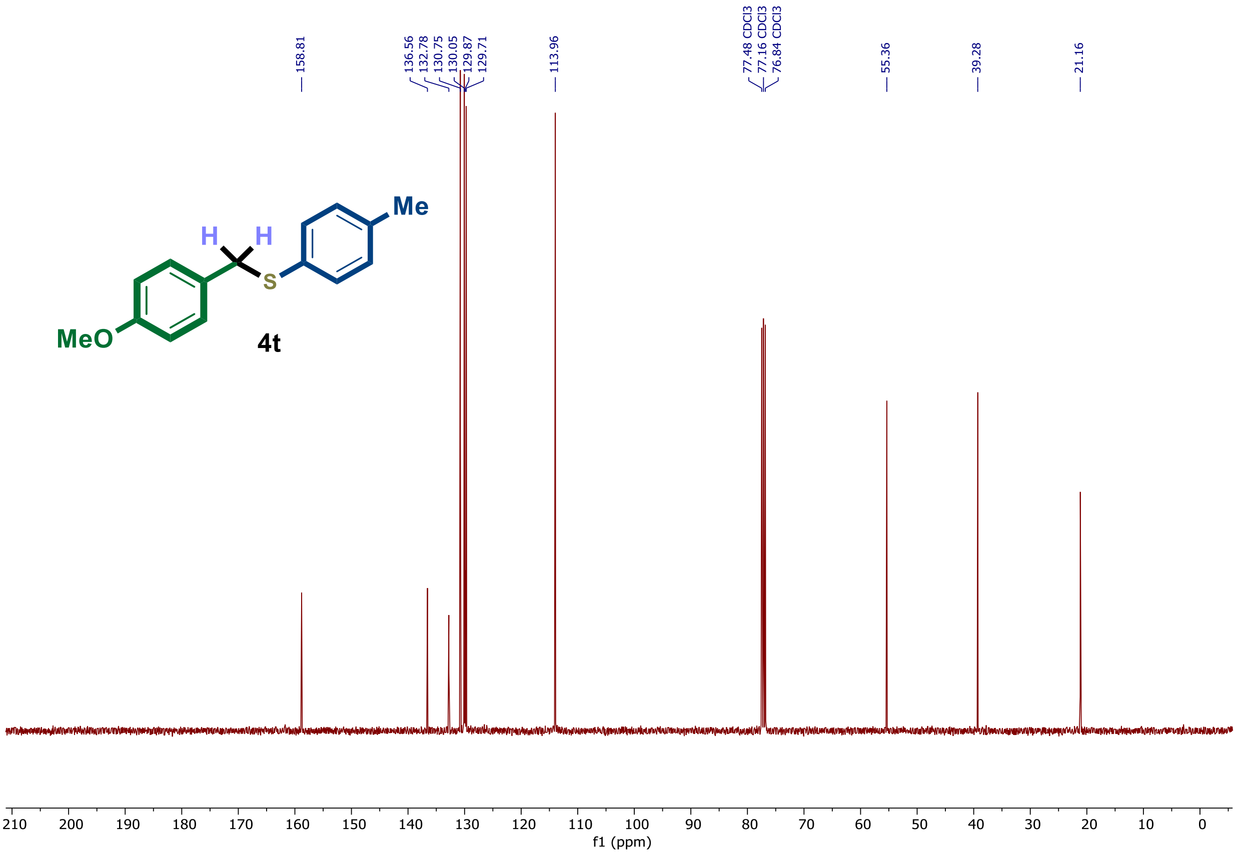

^{1}H NMR (400 MHz, $CDCl_3$) of **4u**

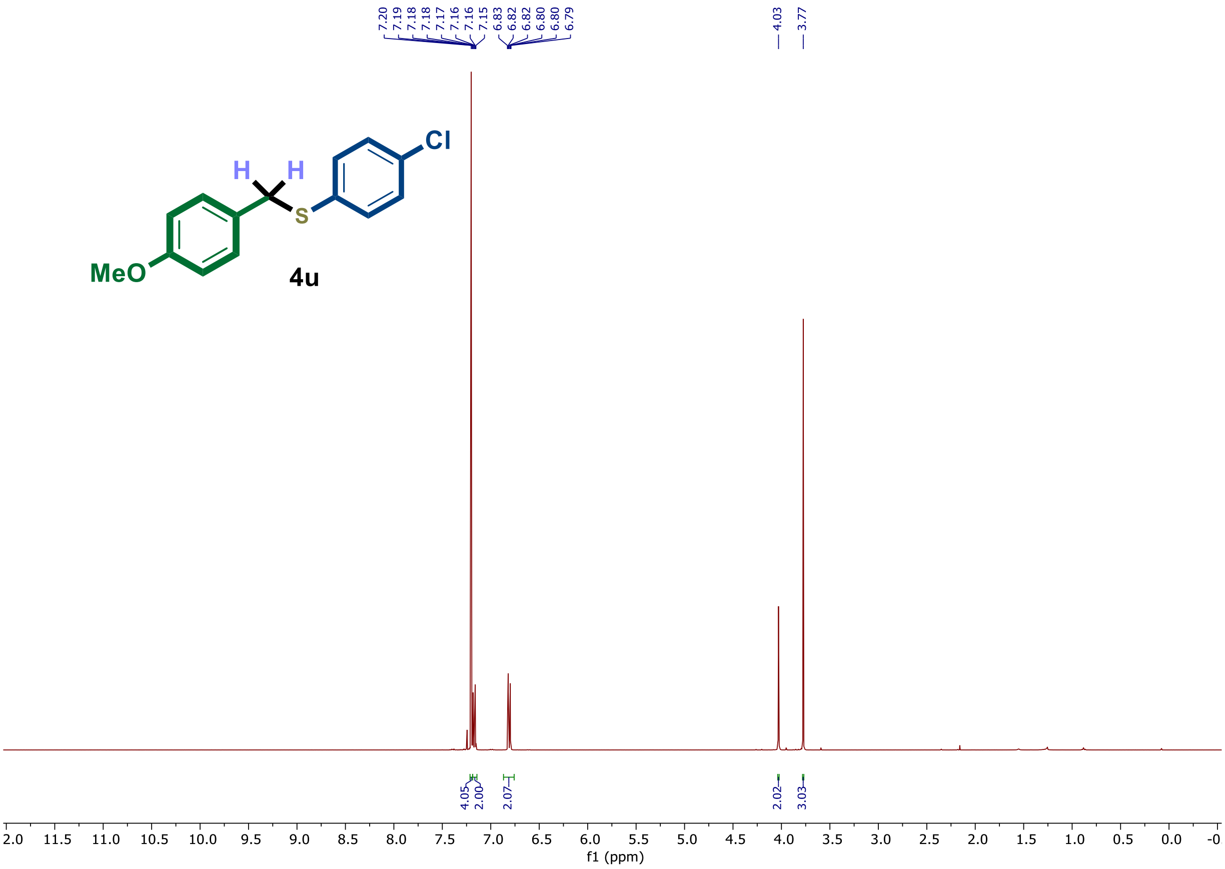

^{13}C NMR (100 MHz, $CDCl_3$) of **4u**

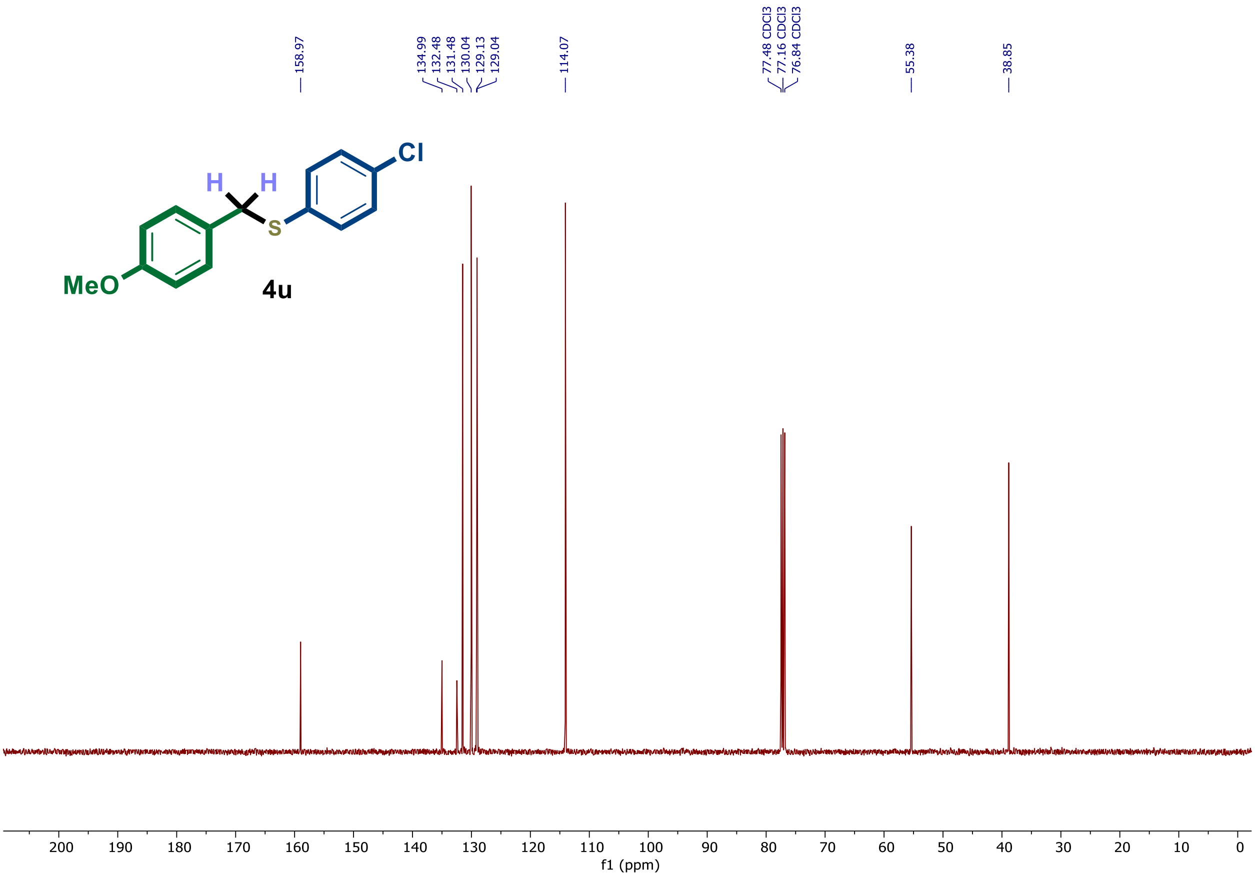

^{1}H NMR (400 MHz, $CDCl_3$) of **4v**

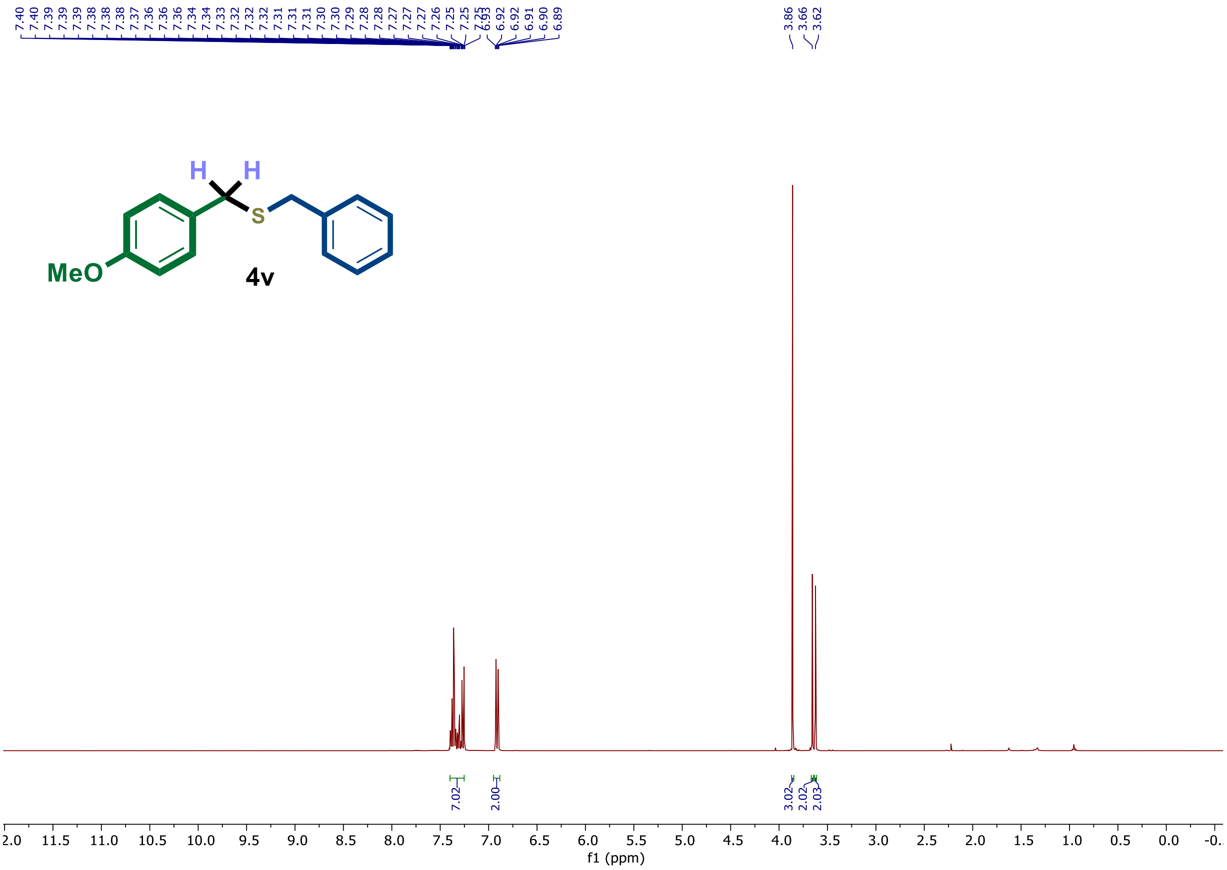

^{13}C NMR (100 MHz, $CDCl_3$) of **4v**

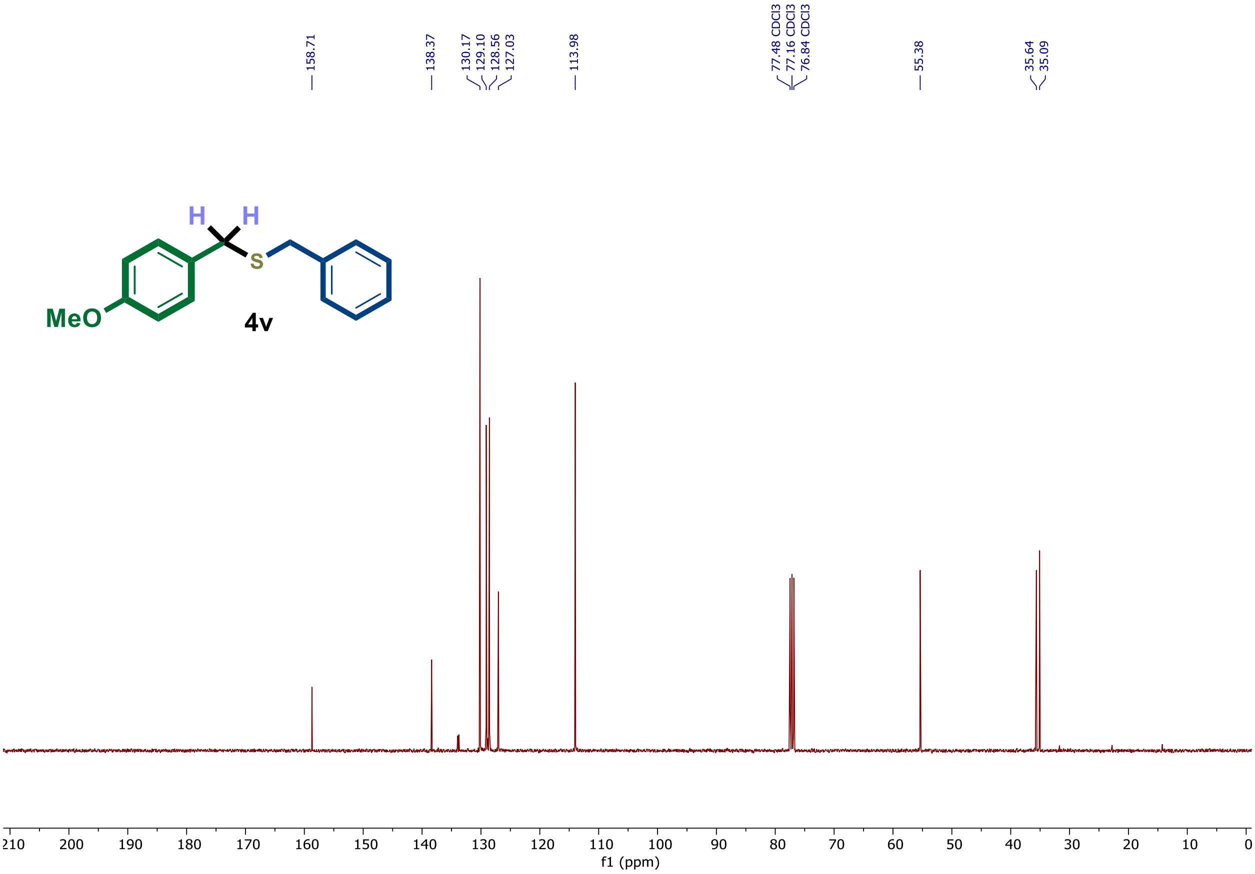

^{1}H NMR (400 MHz, $CDCl_3$) of **4w**

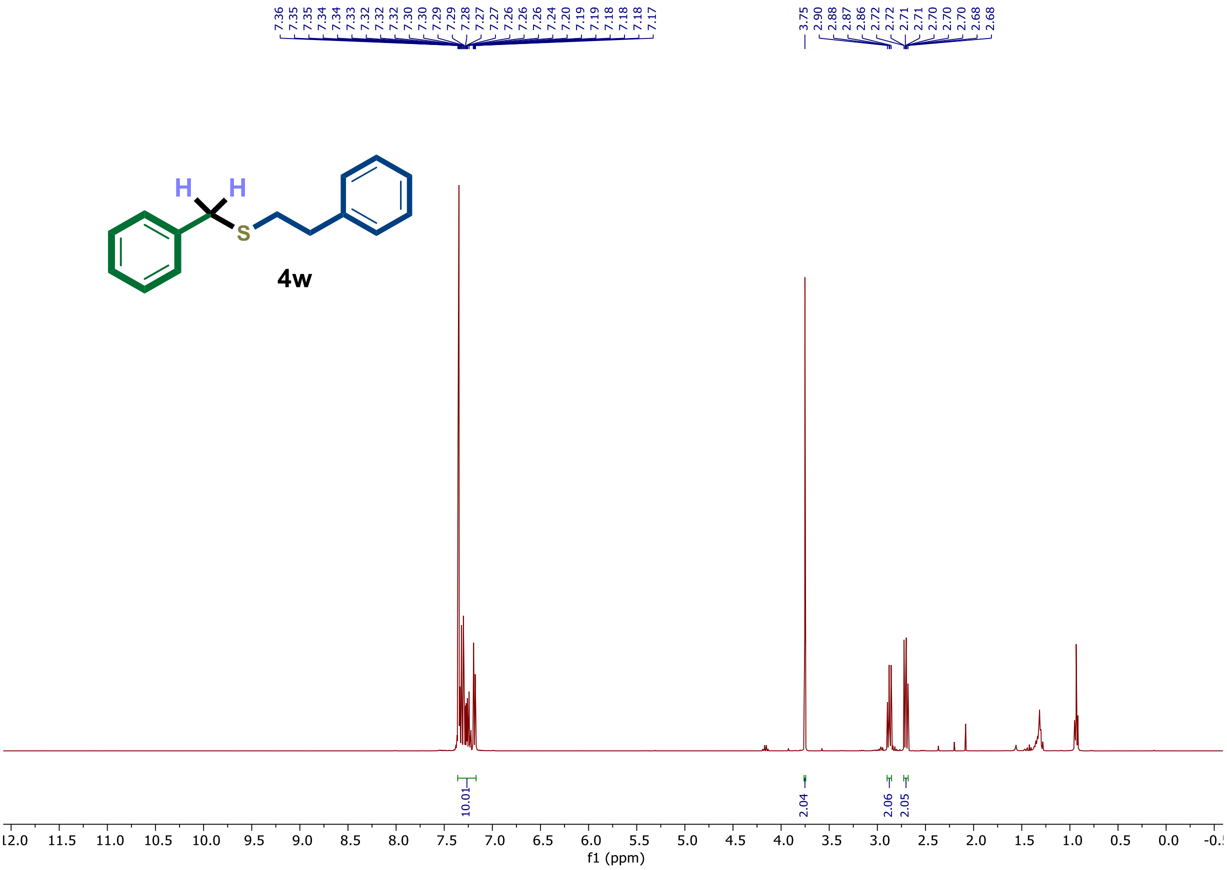

^{13}C NMR (100 MHz, $CDCl_3$) of **4w**

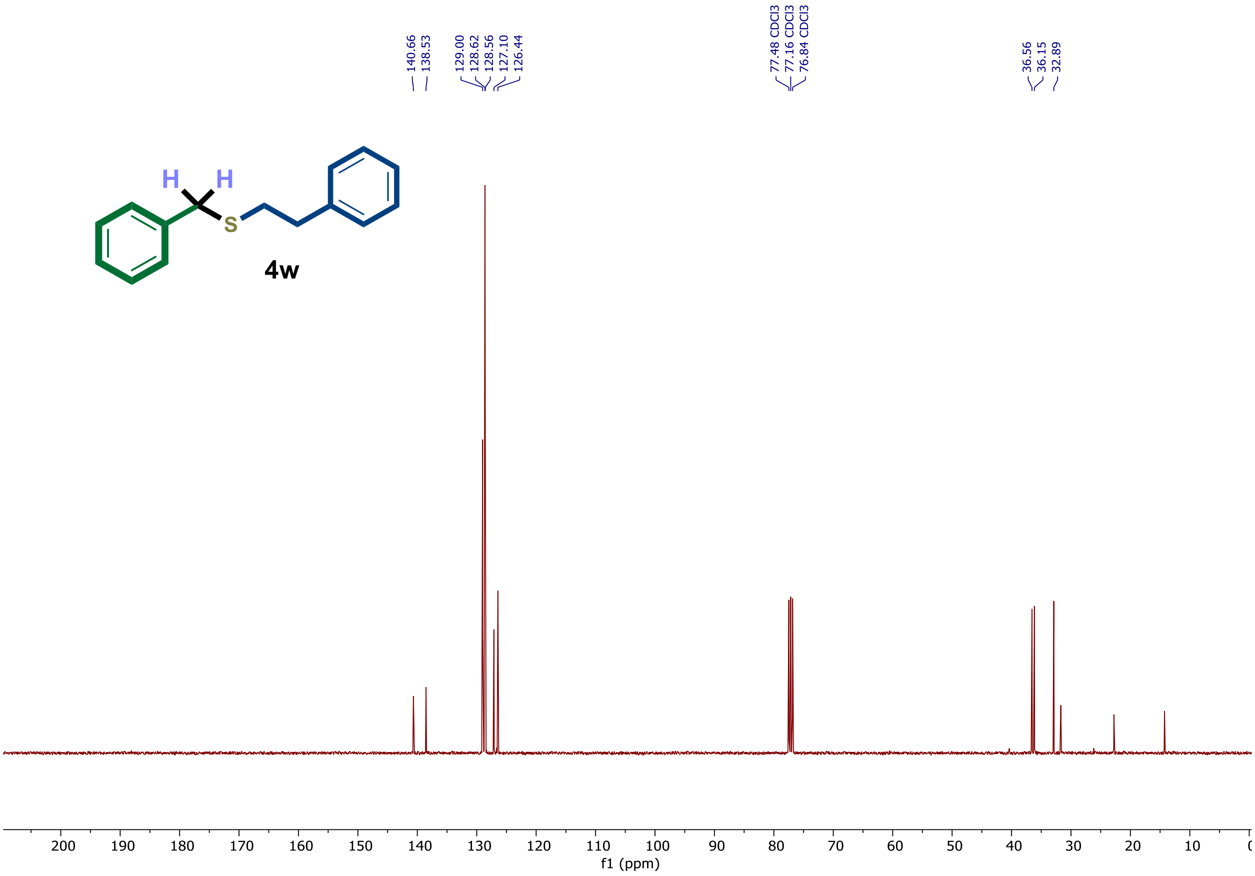

^{1}H NMR (400 MHz, $CDCl_3$) of **4x**

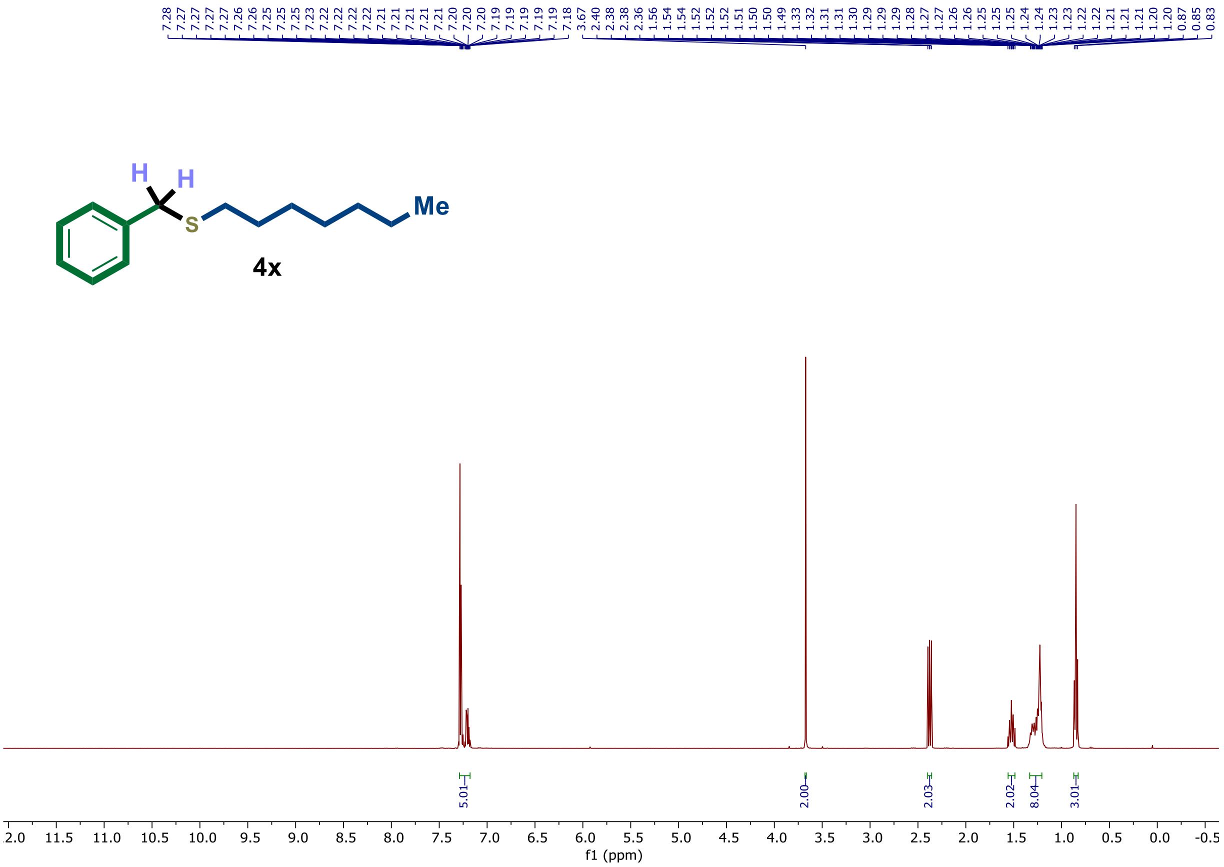

^{13}C NMR (100 MHz, $CDCl_3$) of **4x**

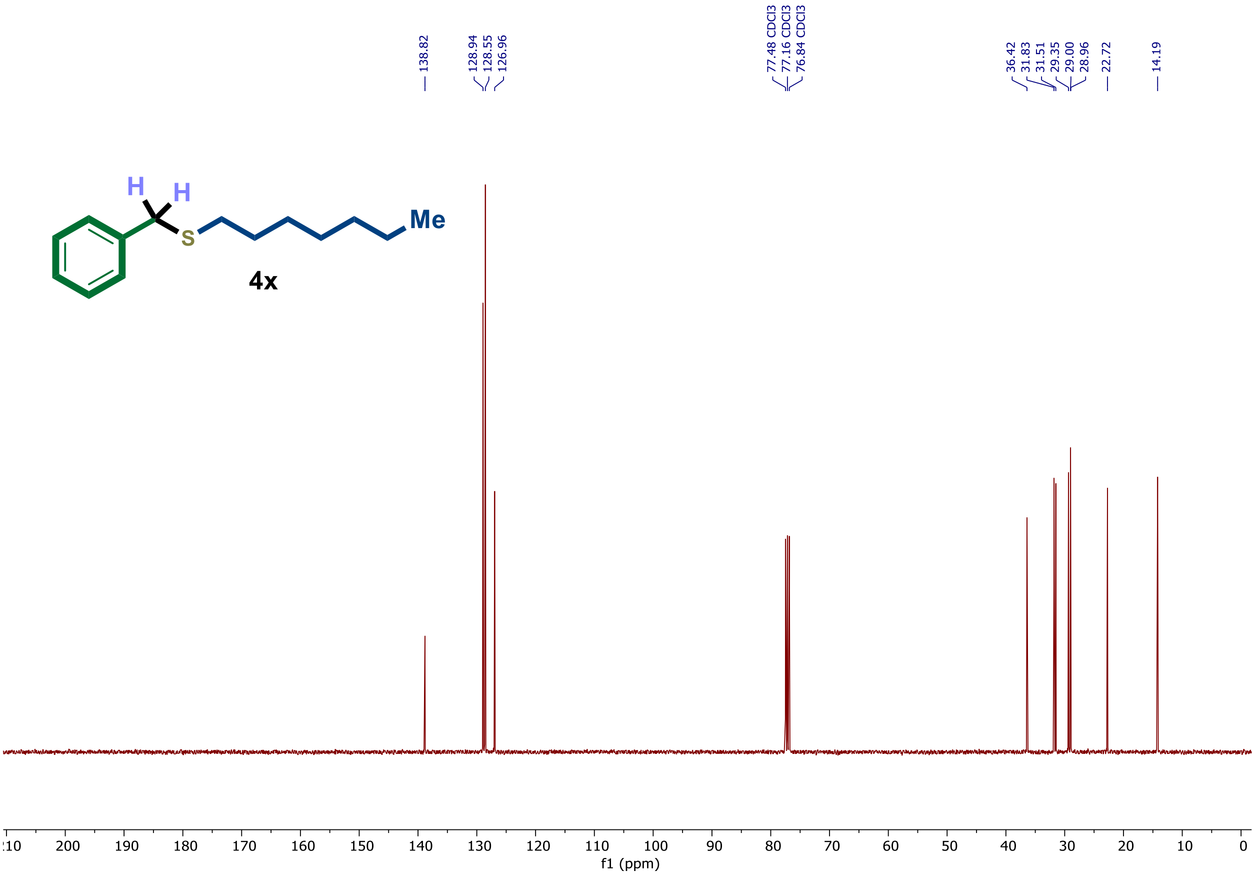

^{1}H NMR (400 MHz, $CDCl_3$) of **4y**

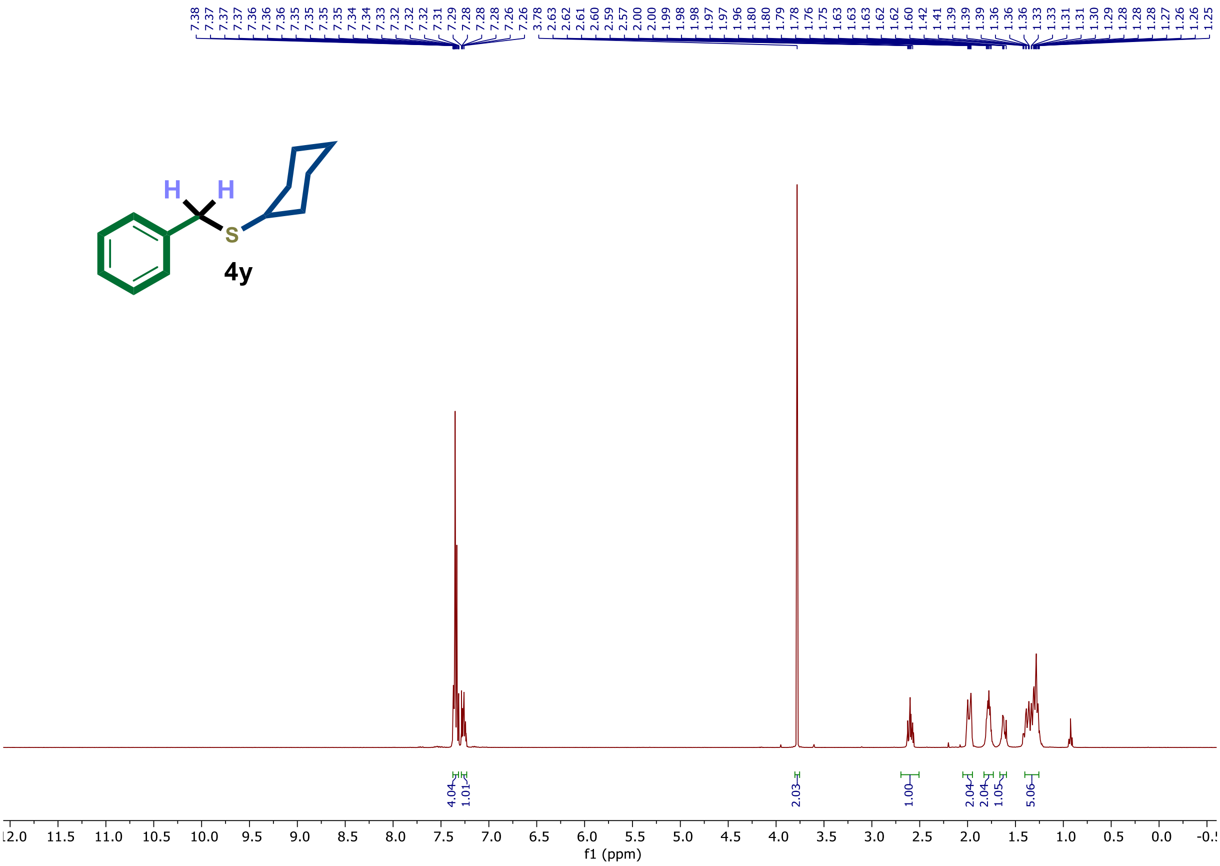

^{13}C NMR (100 MHz, $CDCl_3$) of **4y**

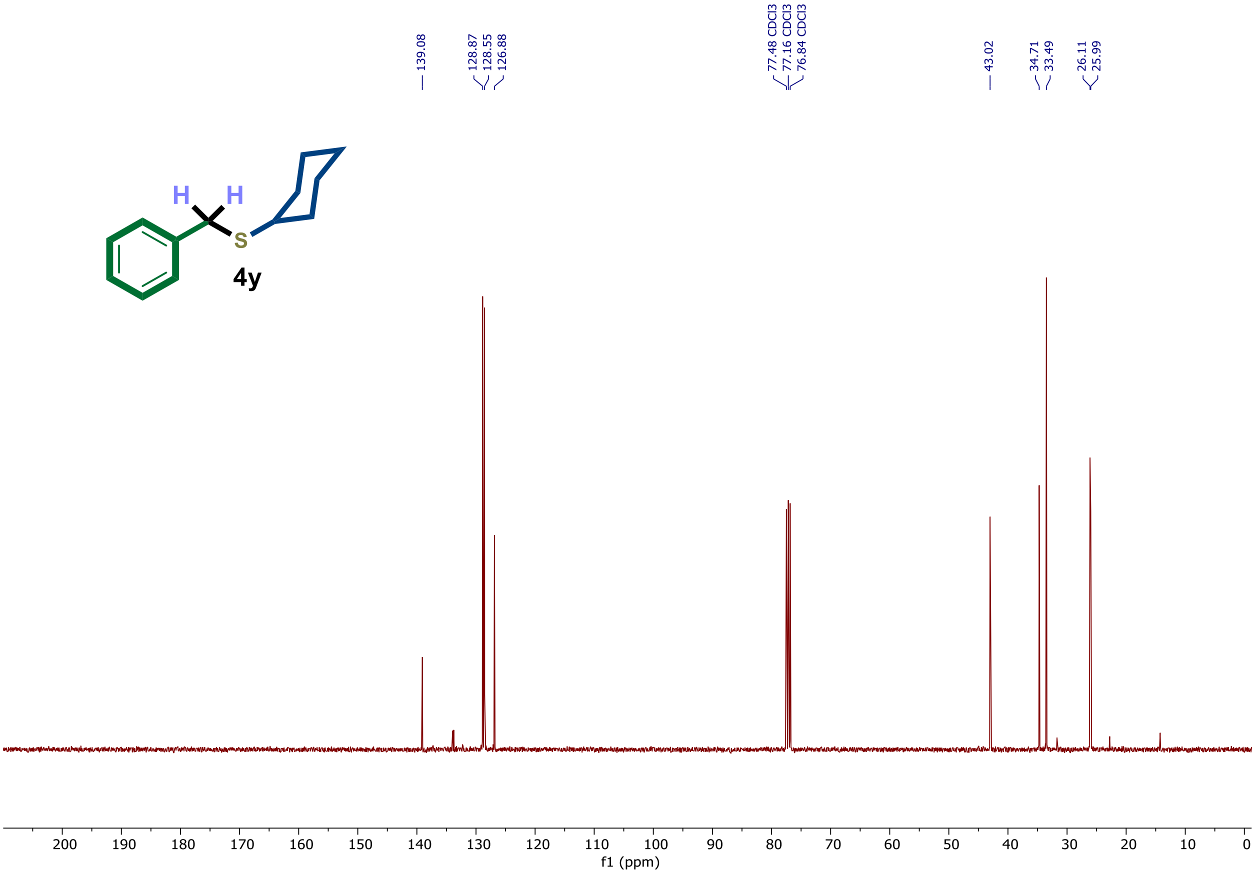

^{1}H NMR (400 MHz, $CDCl_3$) of **4z**

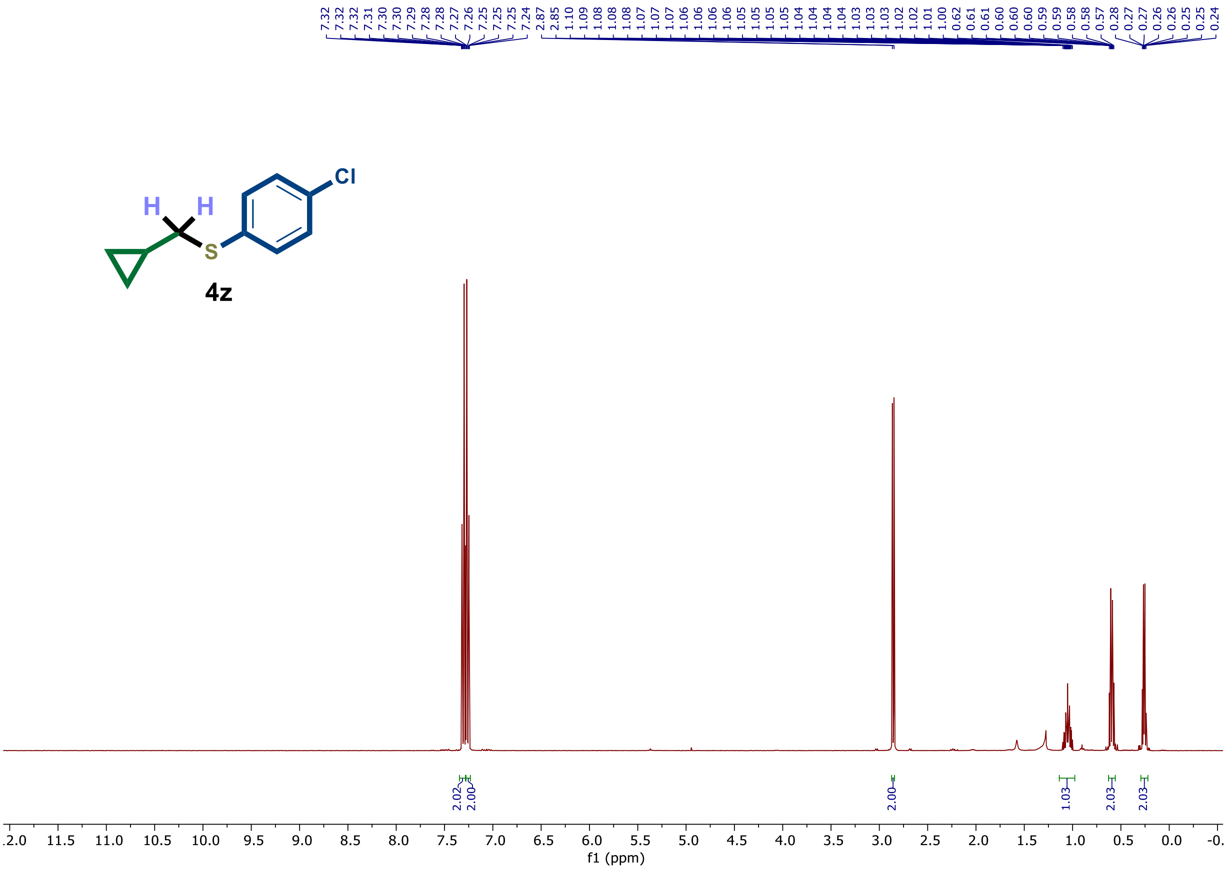

^{13}C NMR (100 MHz, $CDCl_3$) of **4z**

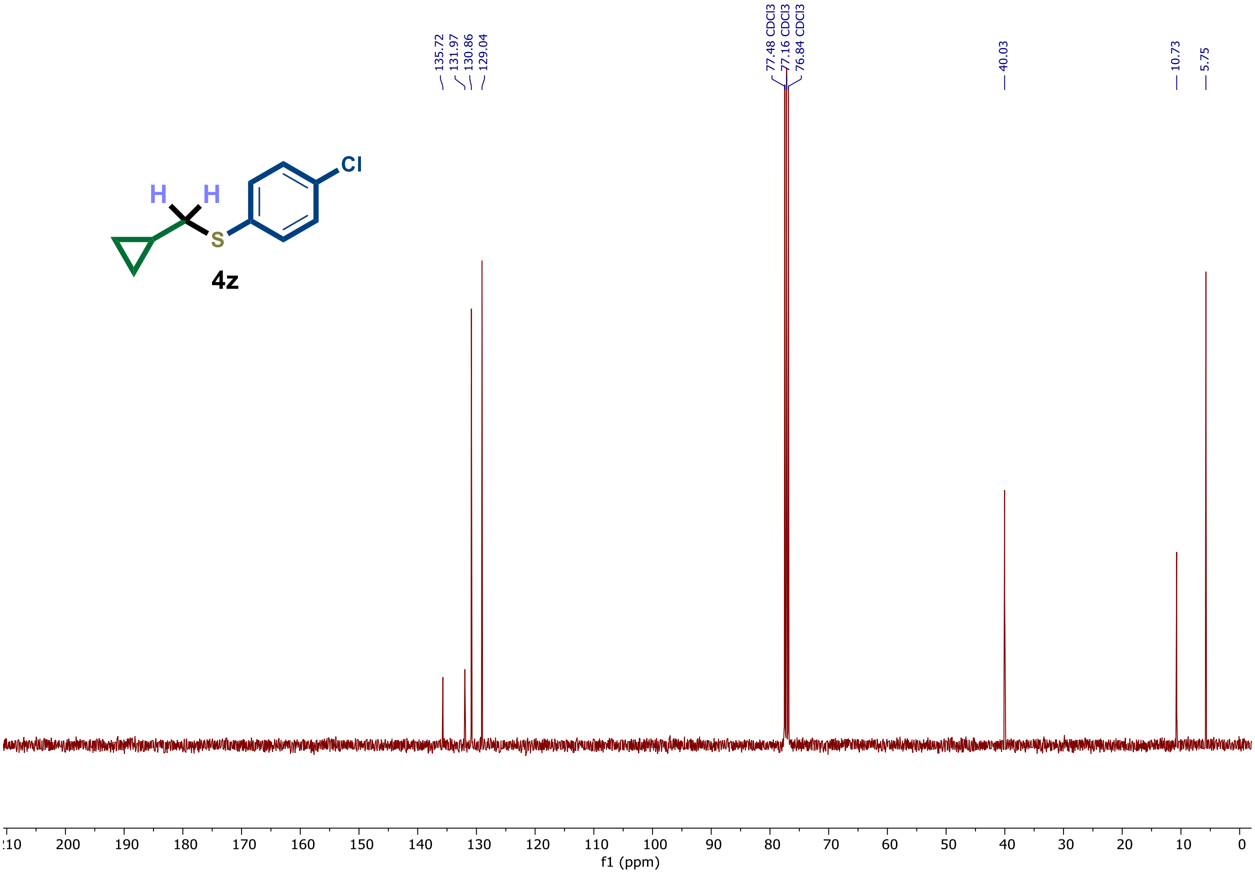

^{1}H NMR (400 MHz, $CDCl_3$) of **4aa**

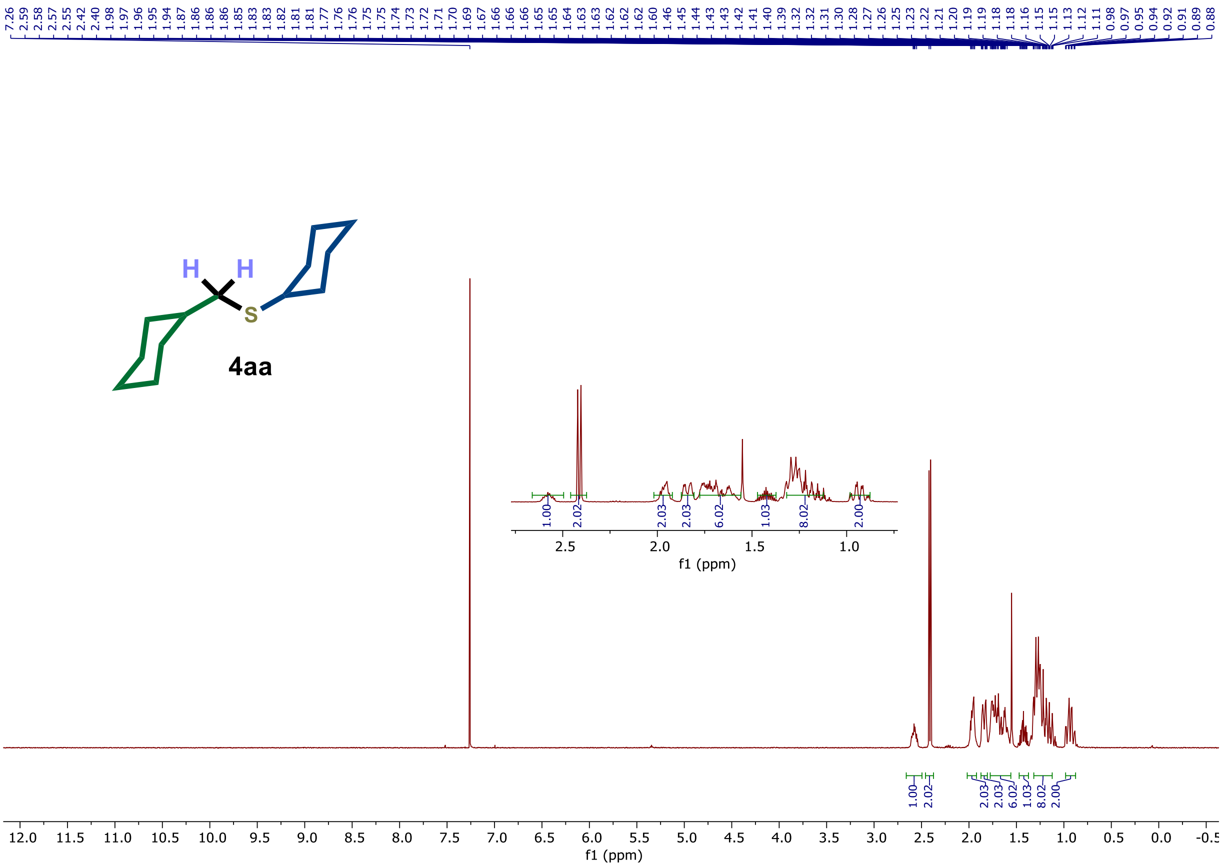

^{13}C NMR (100 MHz, $CDCl_3$) of **4aa**

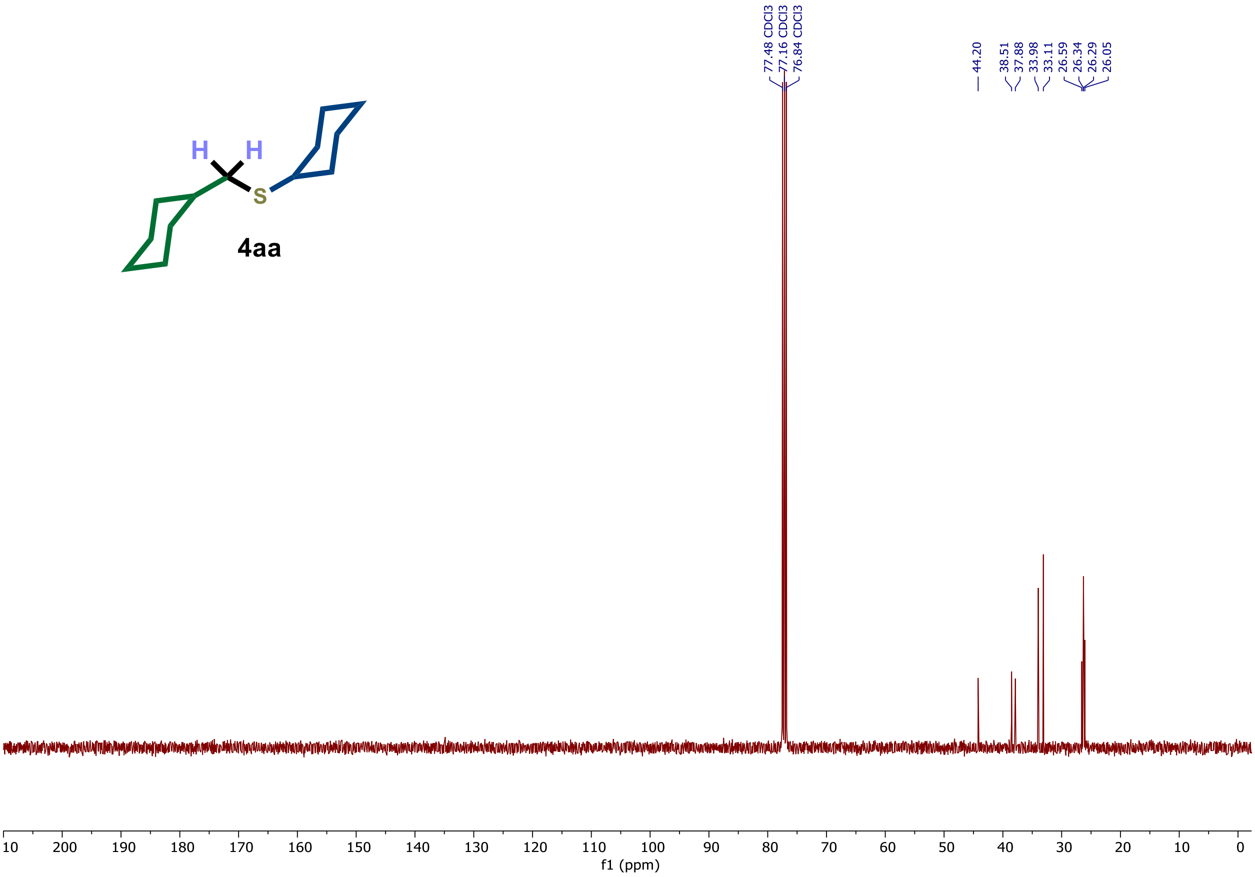

^{1}H NMR (400 MHz, $CDCl_3$) of **4ab**

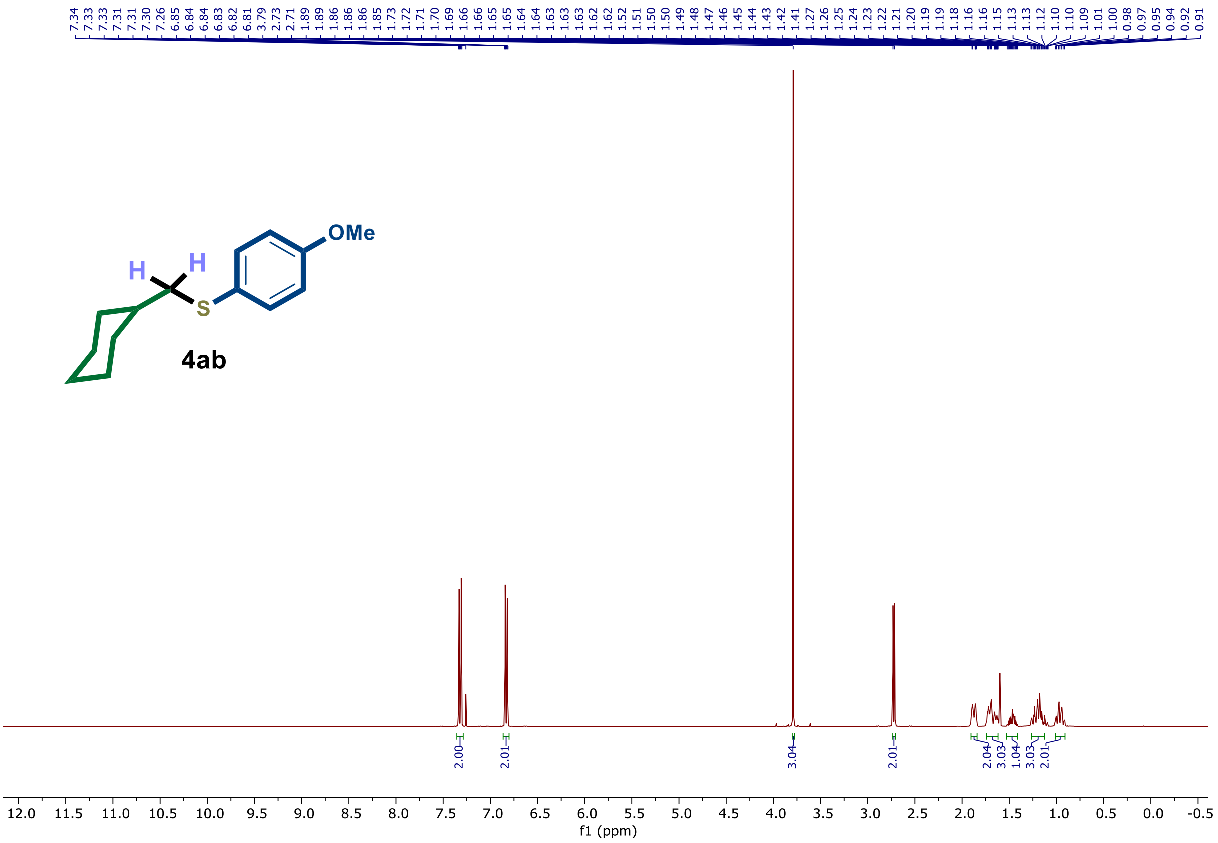

^{13}C NMR (100 MHz, $CDCl_3$) of **4ab**

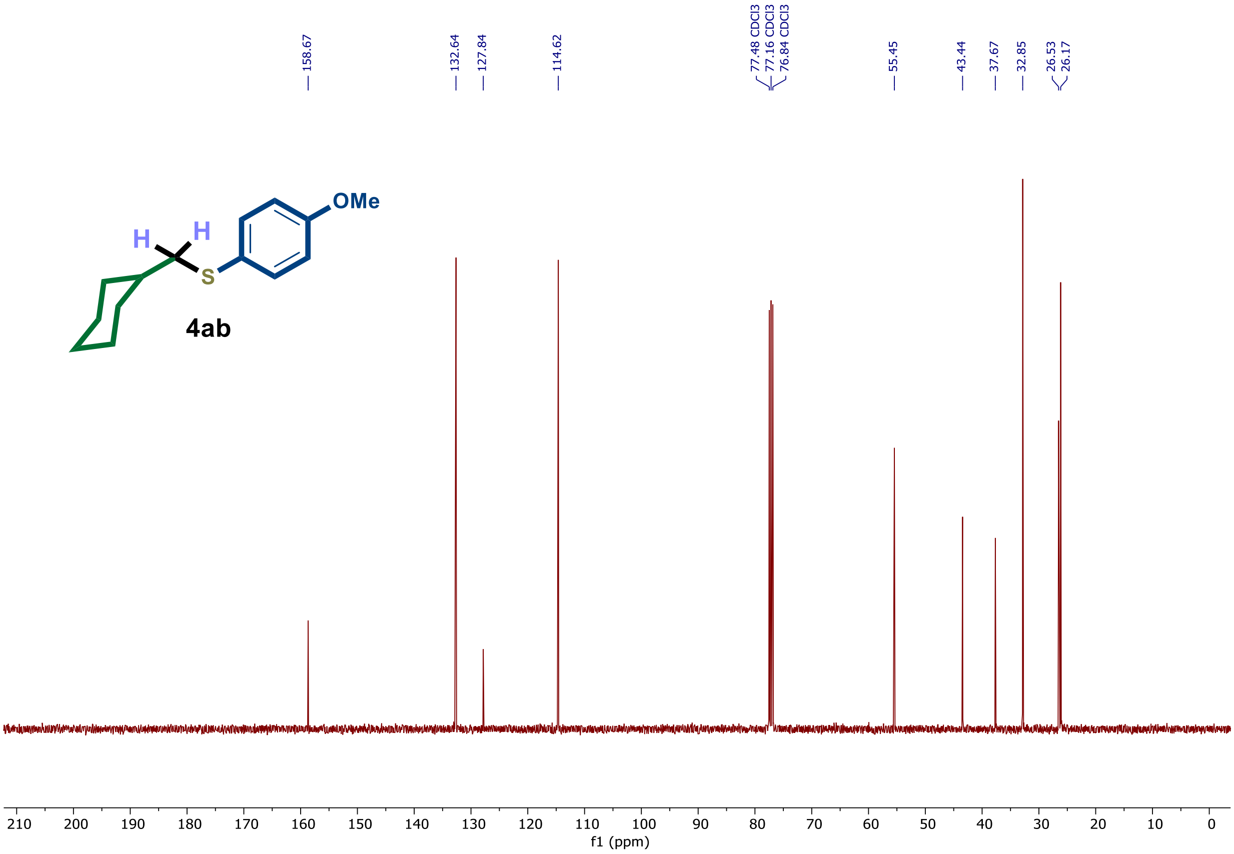

^{1}H NMR (400 MHz, $CDCl_3$) of **4ac**

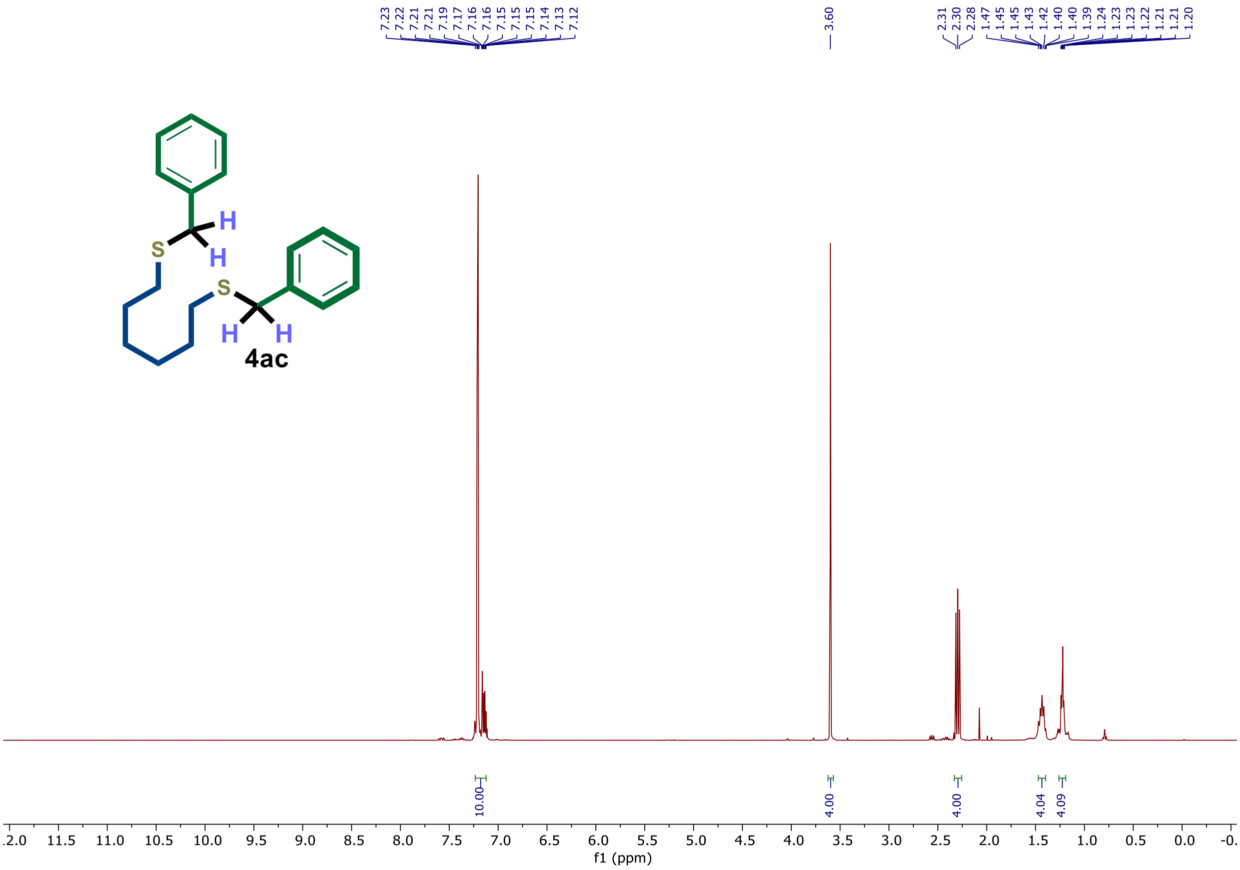

^{13}C NMR (100 MHz, $CDCl_3$) of **4ac**

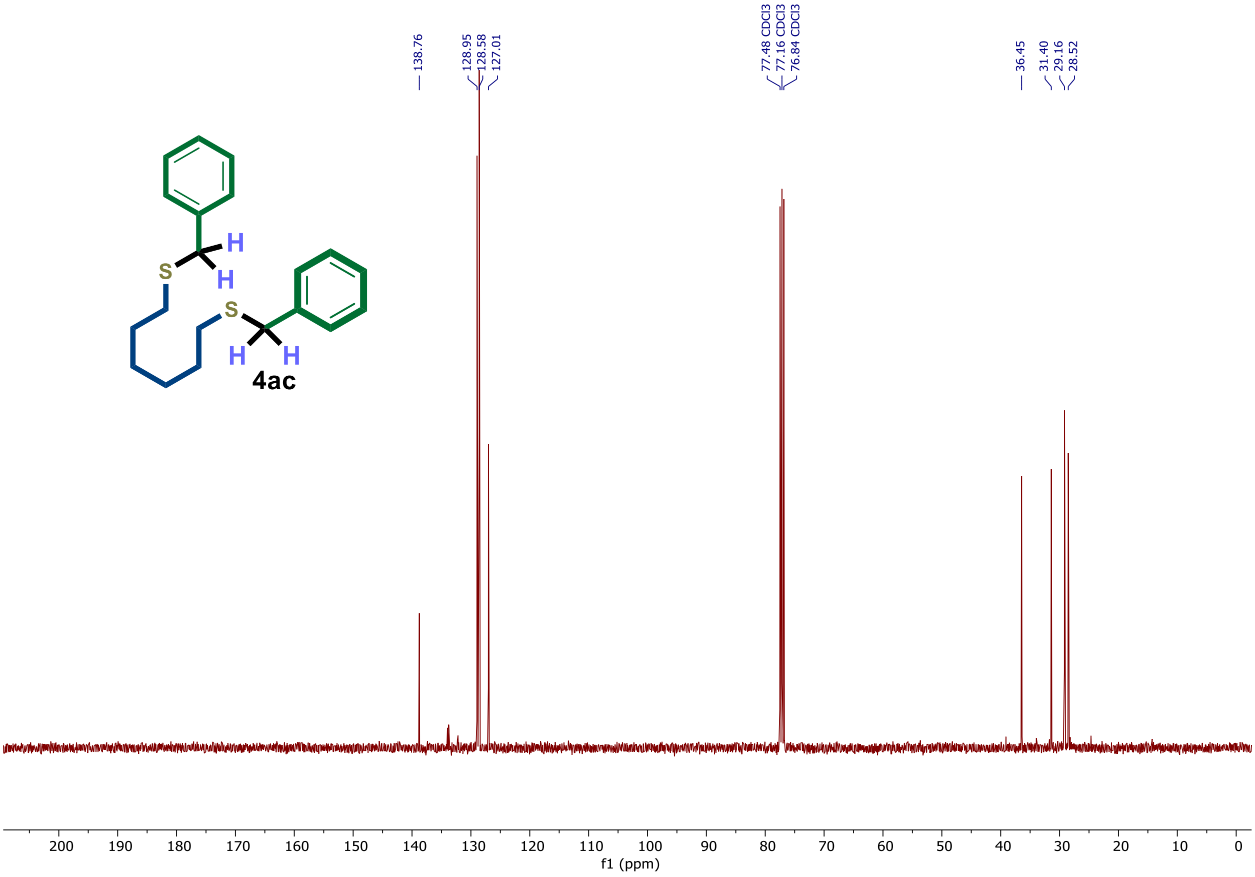

^{1}H NMR (400 MHz, $CDCl_3$) of **III**

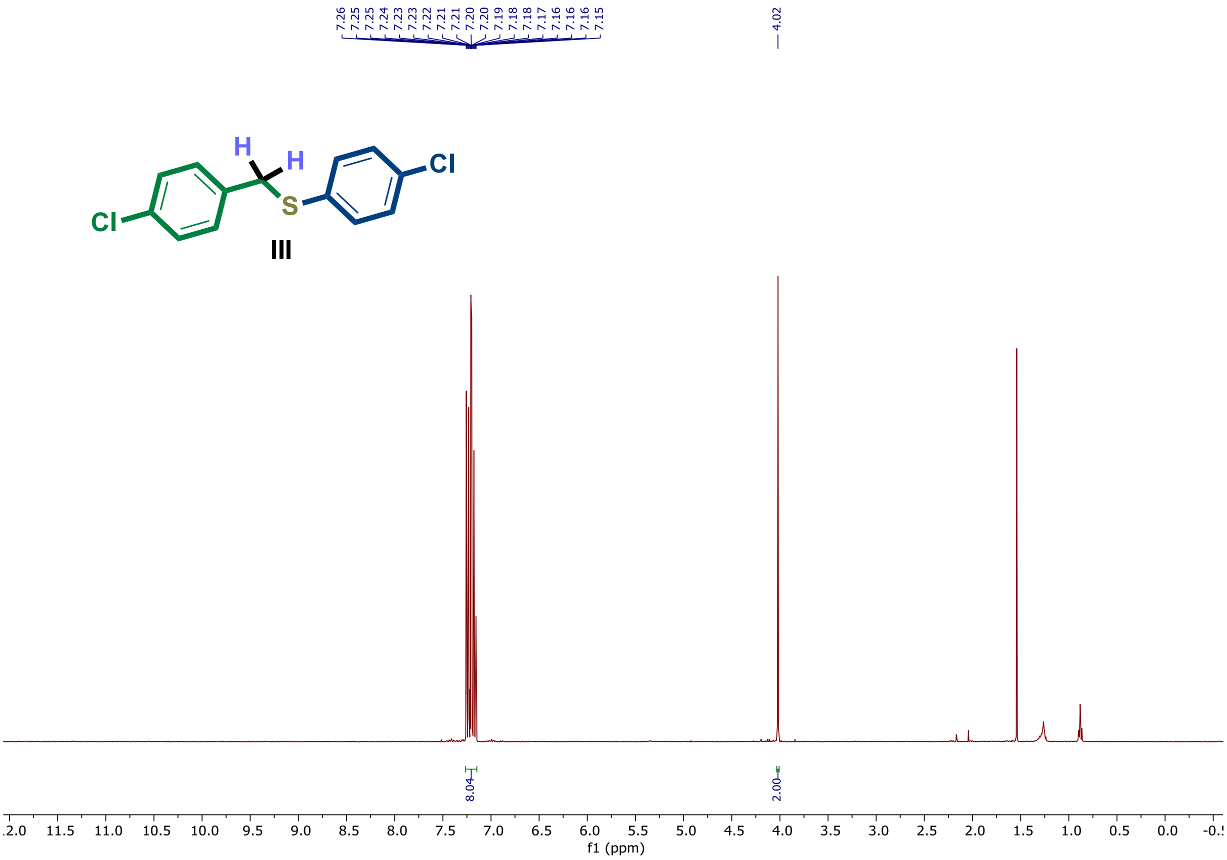

^{13}C NMR (100 MHz, $CDCl_3$) of **III**

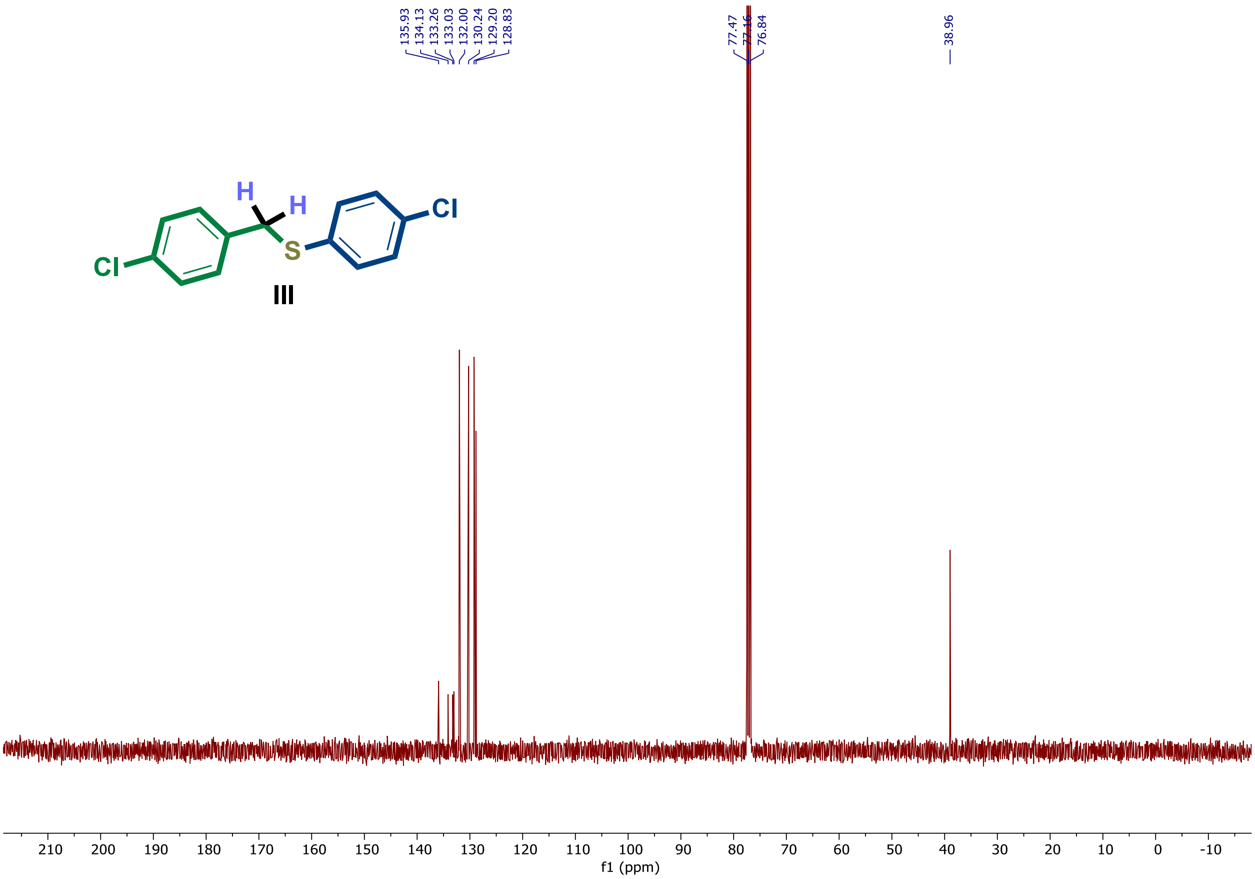

^{1}H NMR (400 MHz, $CDCl_3$) of **4-Df**

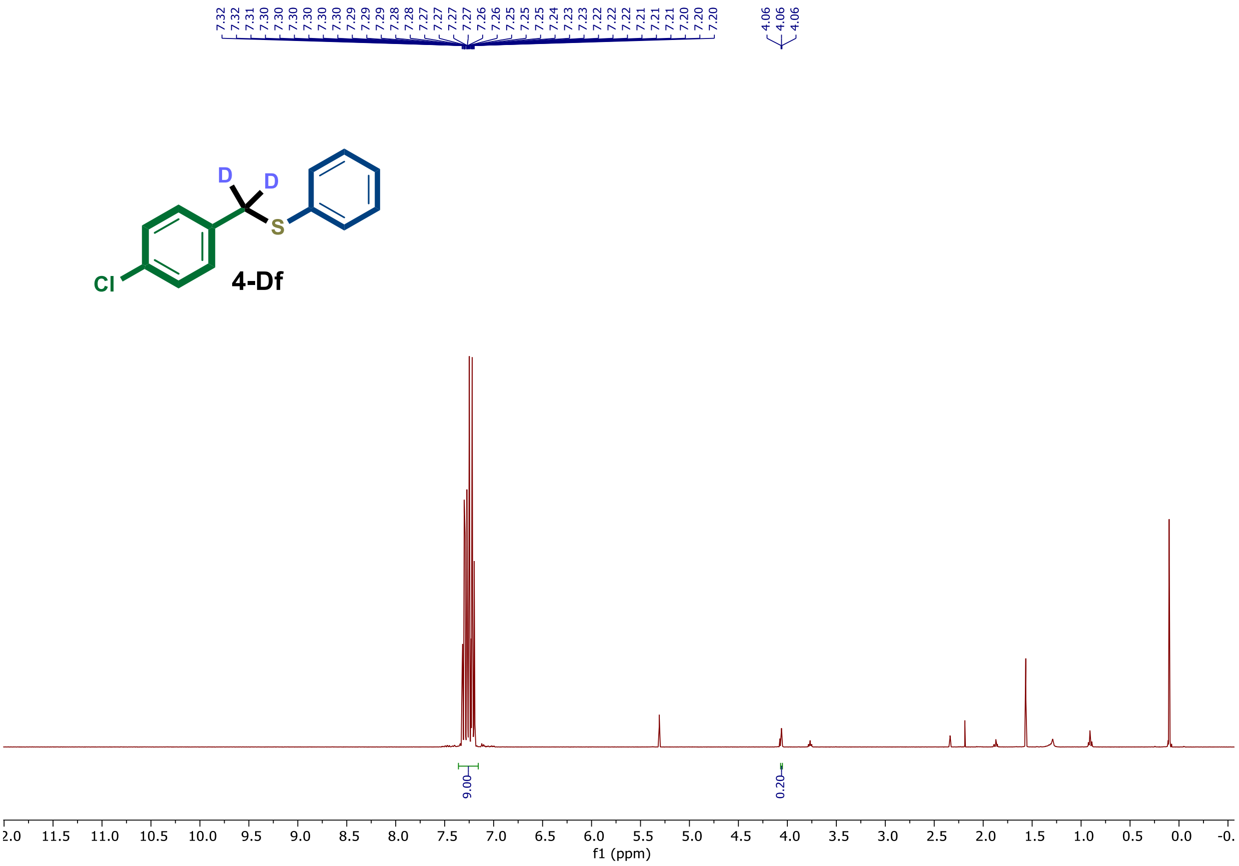

^{13}C NMR (100 MHz, $CDCl_3$) of **4-Df**

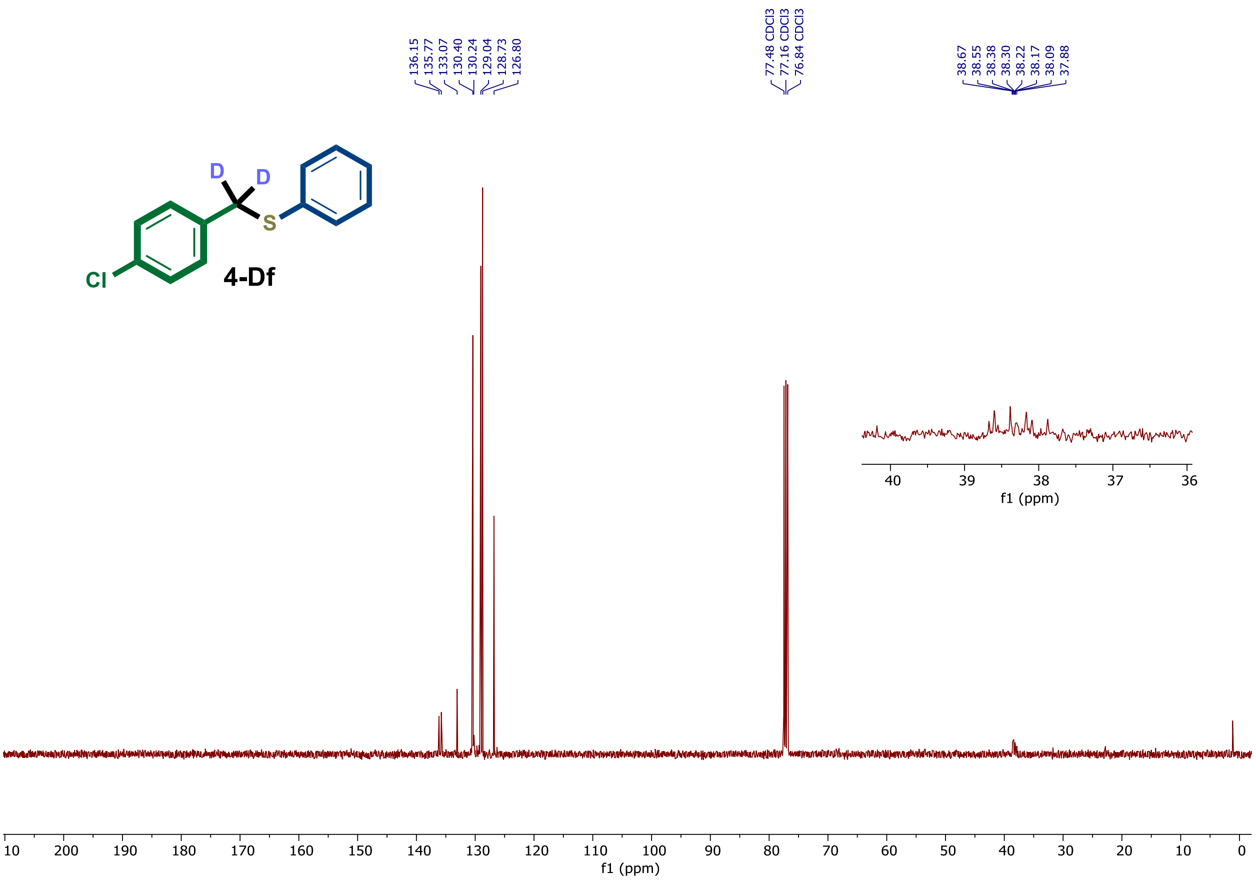

^{1}H NMR (400 MHz, $CDCl_3$) of **4-Dn**

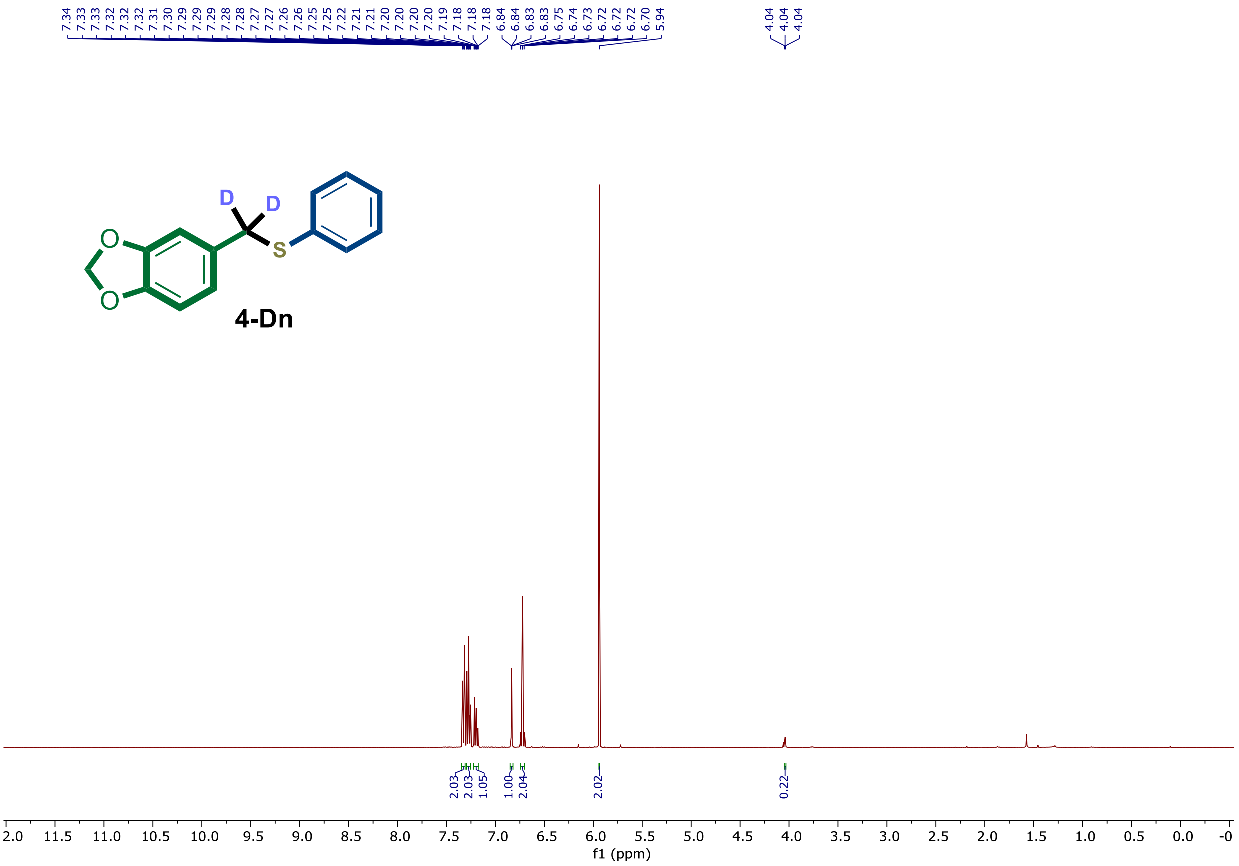

^{13}C NMR (100 MHz, $CDCl_3$) of **4-Dn**

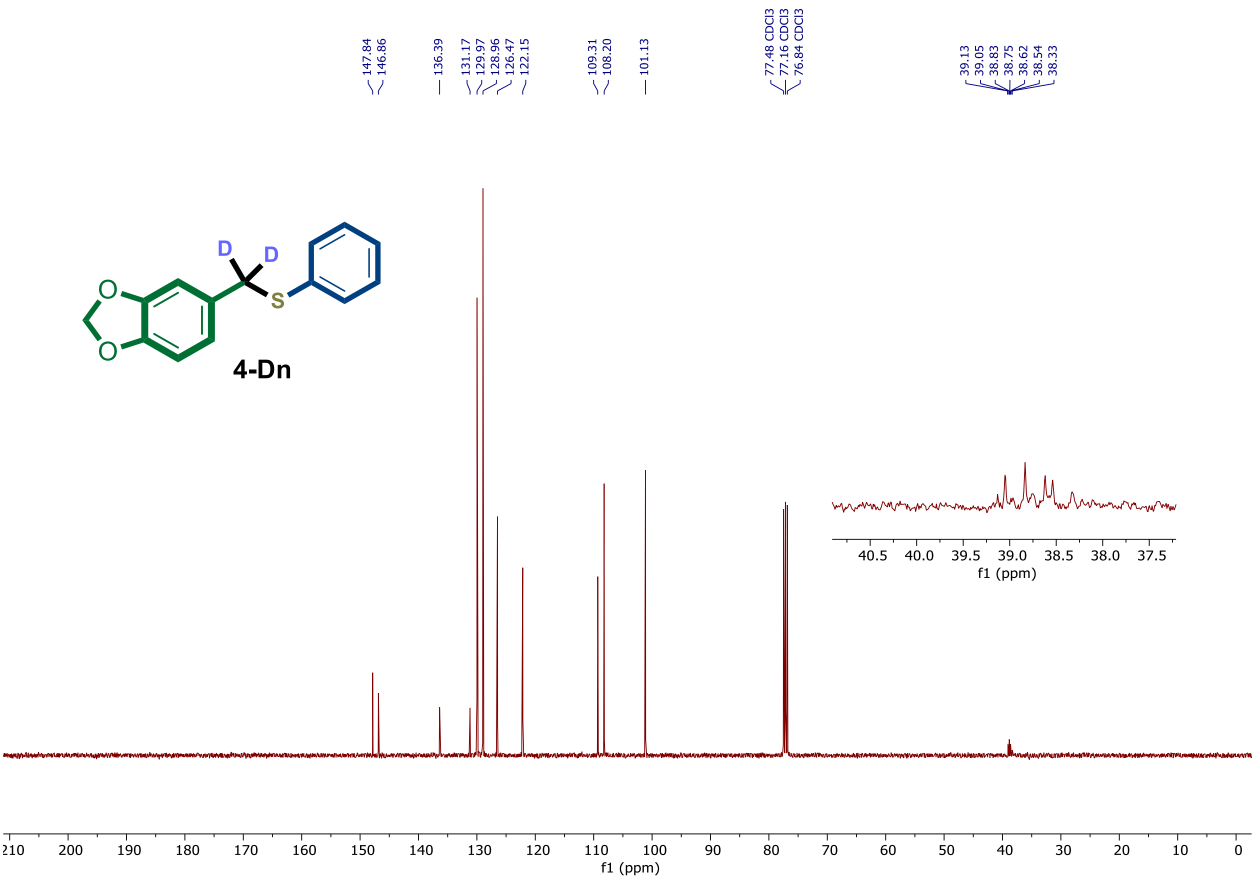

^{1}H NMR (400 MHz, $CDCl_3$) of **4-Ds**

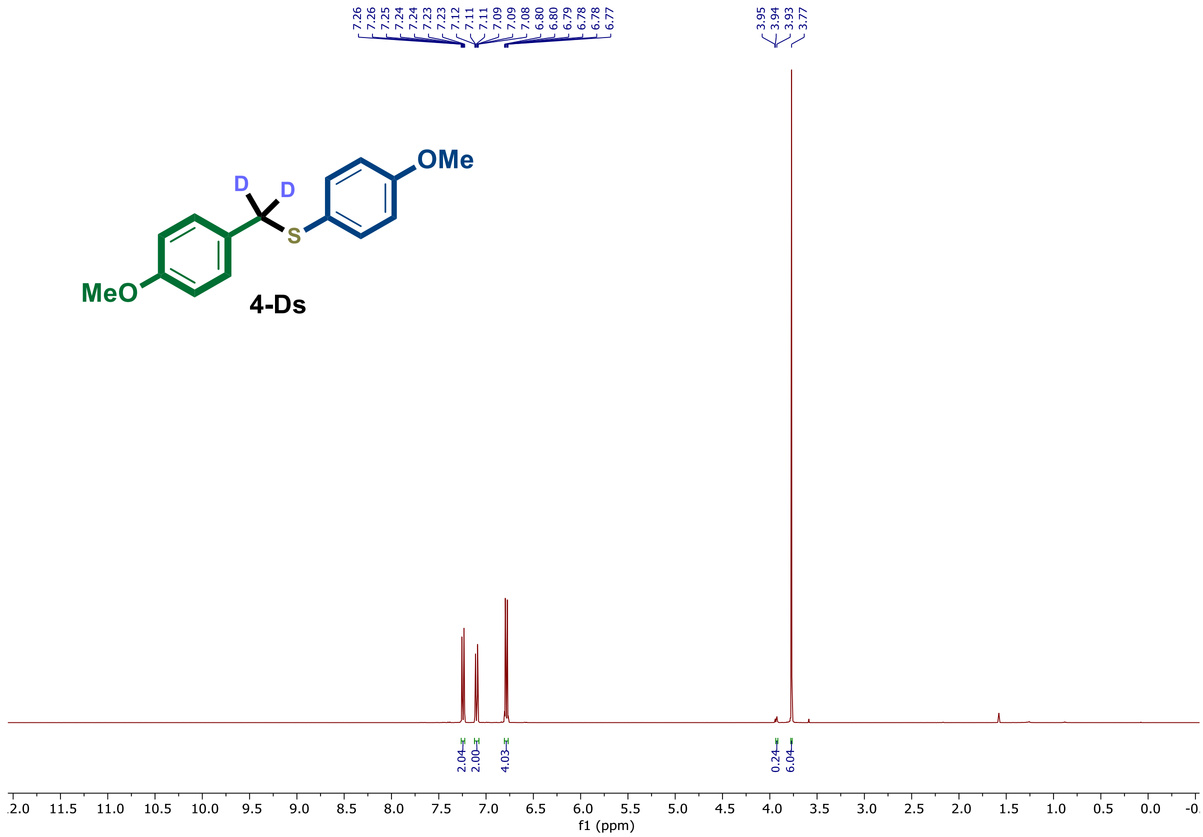

^{13}C NMR (100 MHz, $CDCl_3$) of **4-Ds**

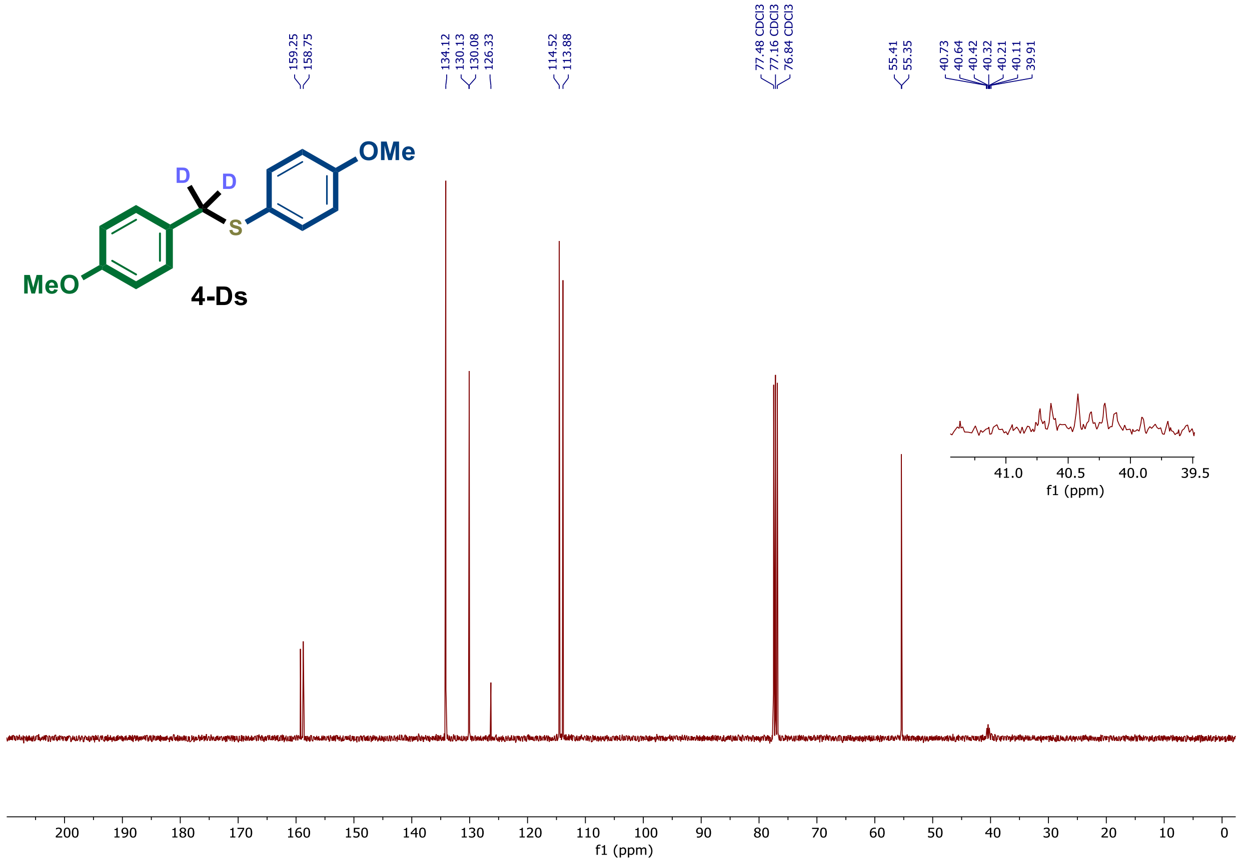

^{2}H NMR (77 MHz, $CDCl_3$) of **4-Ds**

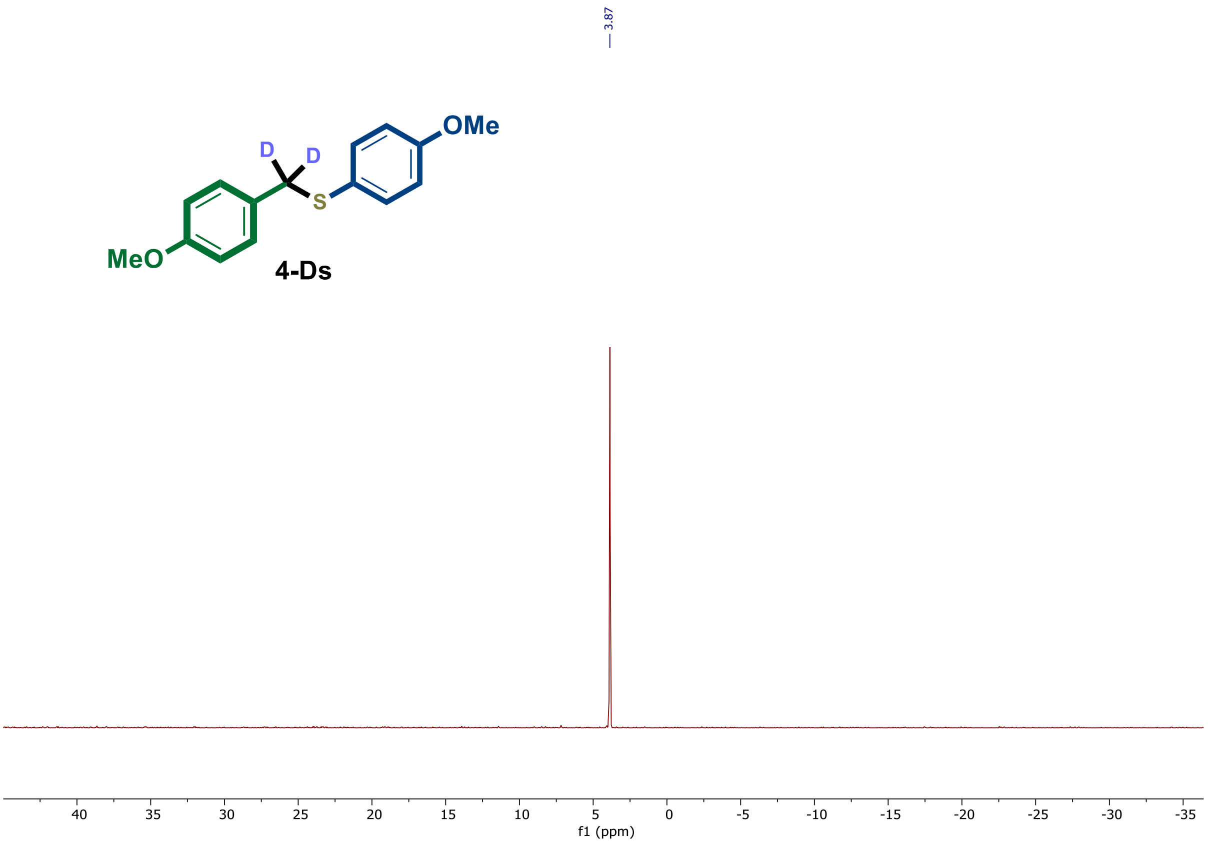

^{1}H NMR (400 MHz, $CDCl_3$) of **4-Dv**

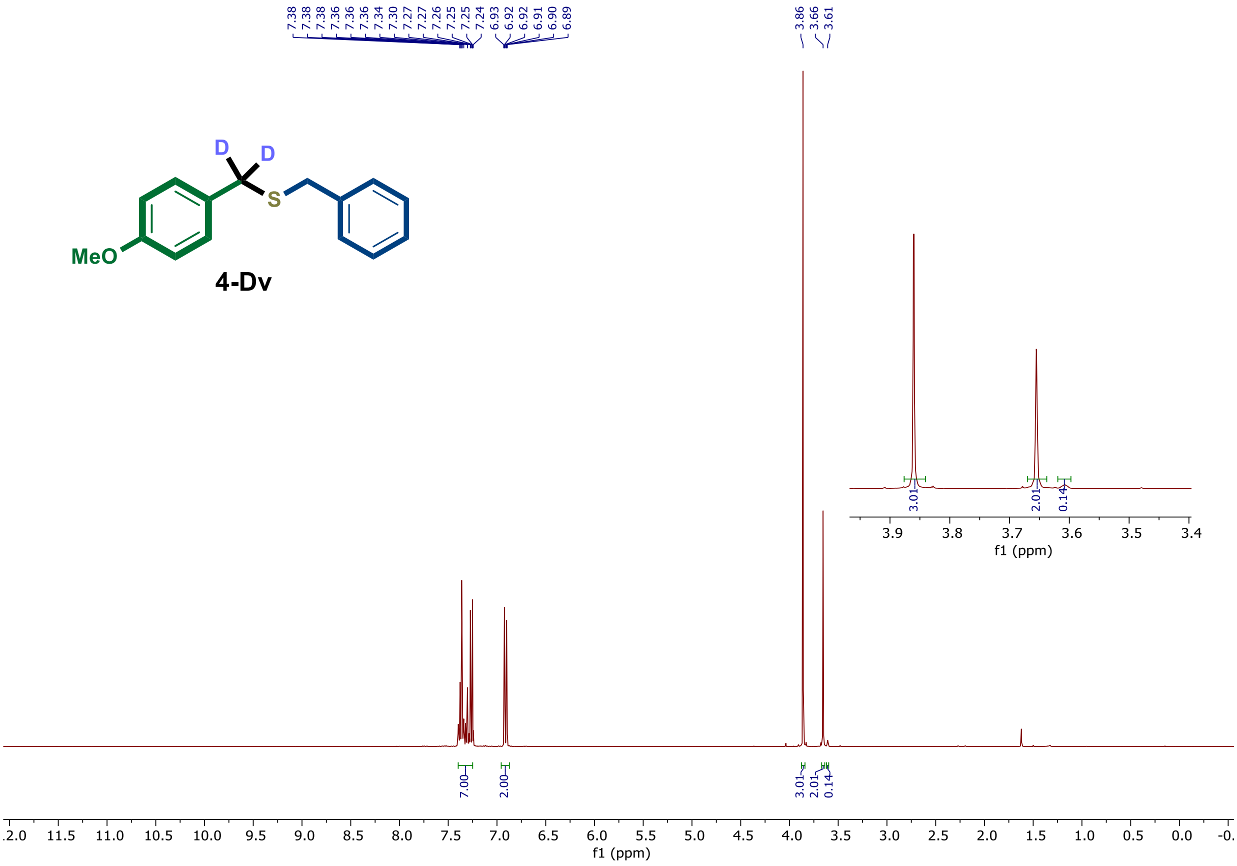

^{13}C NMR (100 MHz, $CDCl_3$) of **4-Dv**

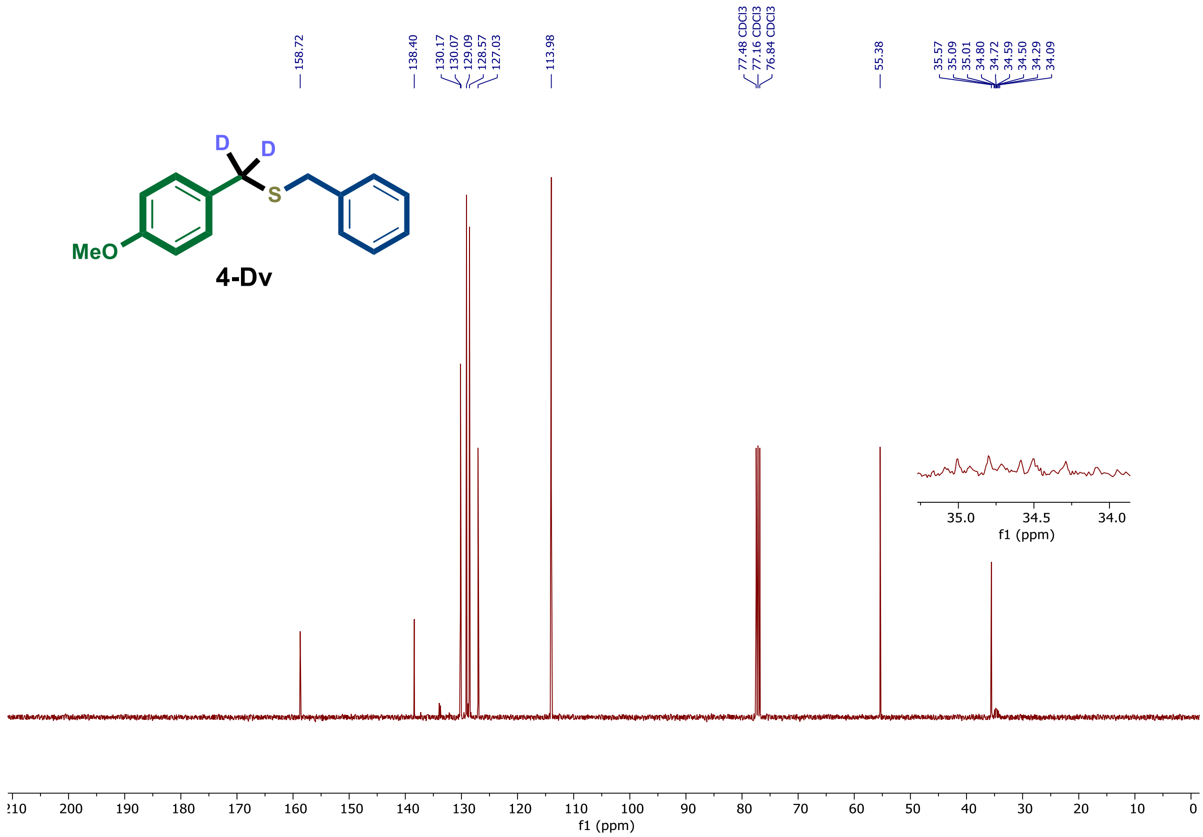

^{1}H NMR (400 MHz, $CDCl_3$) of **4-Dy**

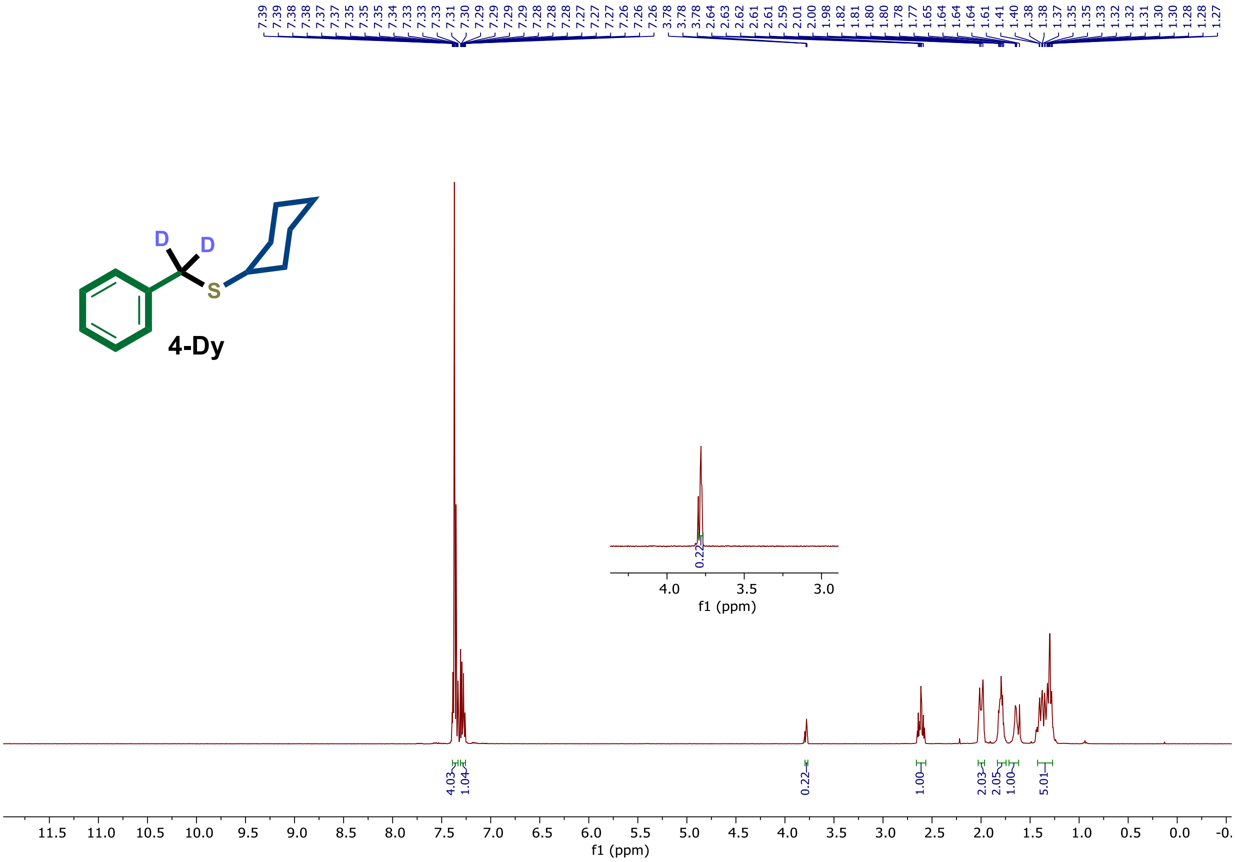

^{13}C NMR (100 MHz, $CDCl_3$) of **4-Dy**

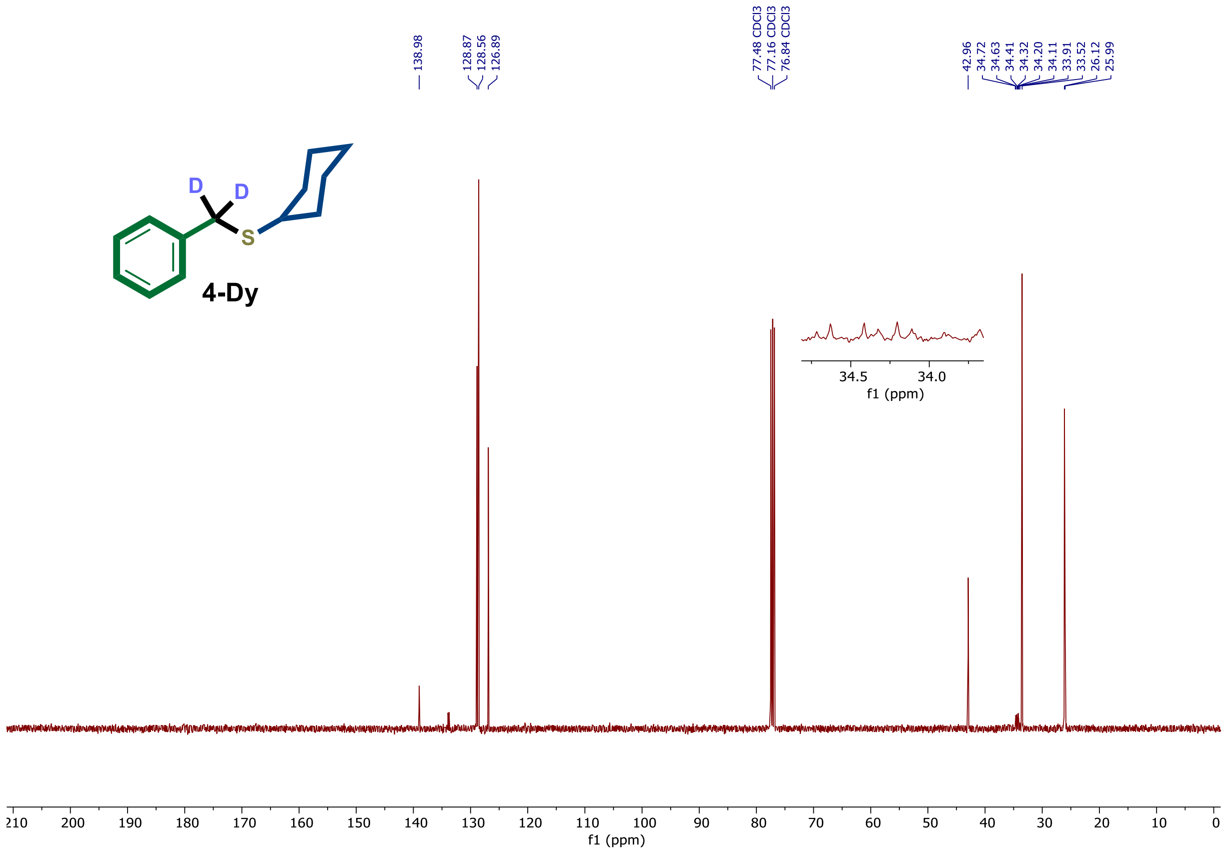

^{1}H NMR (400 MHz, $CDCl_3$) of **4-Dz**

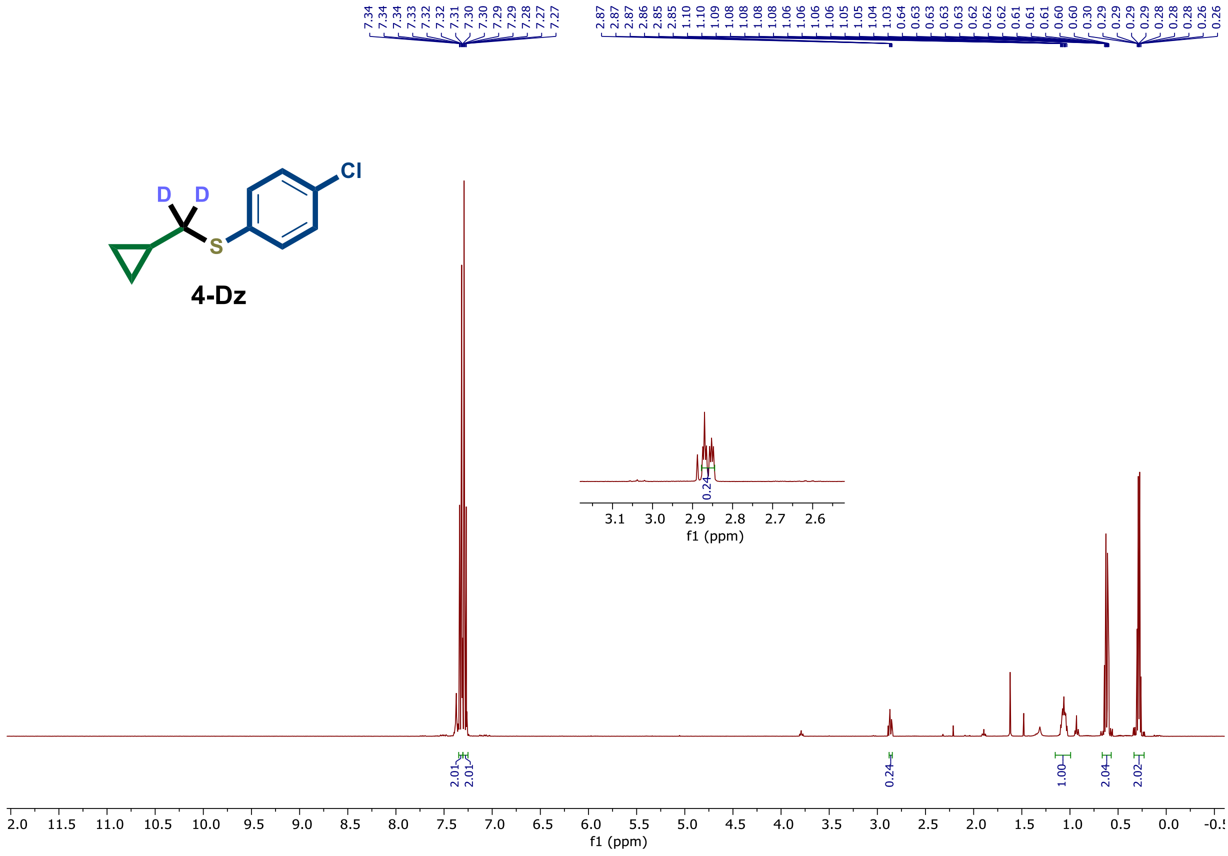

^{13}C NMR (100 MHz, $CDCl_3$) of **4-Dz**

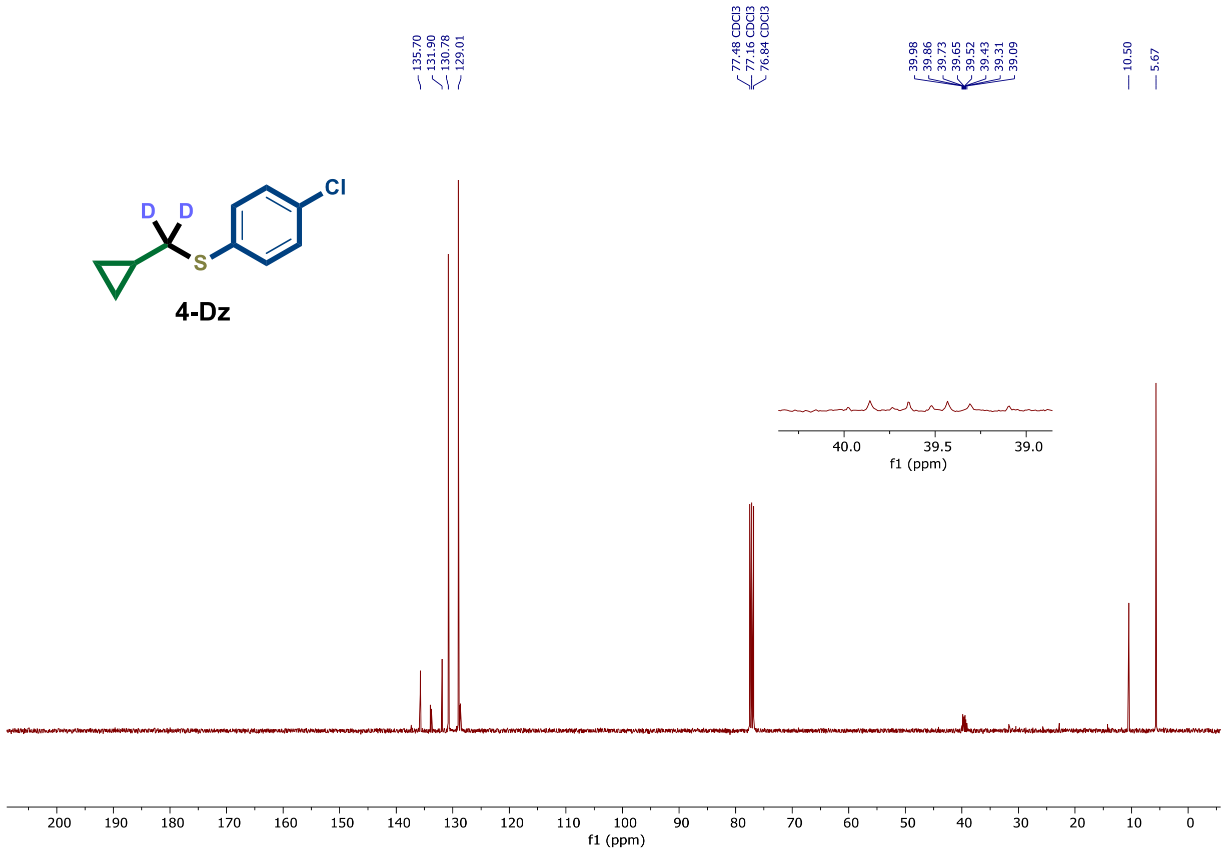

^{1}H NMR (400 MHz, $CDCl_3$) of **4-Daa**

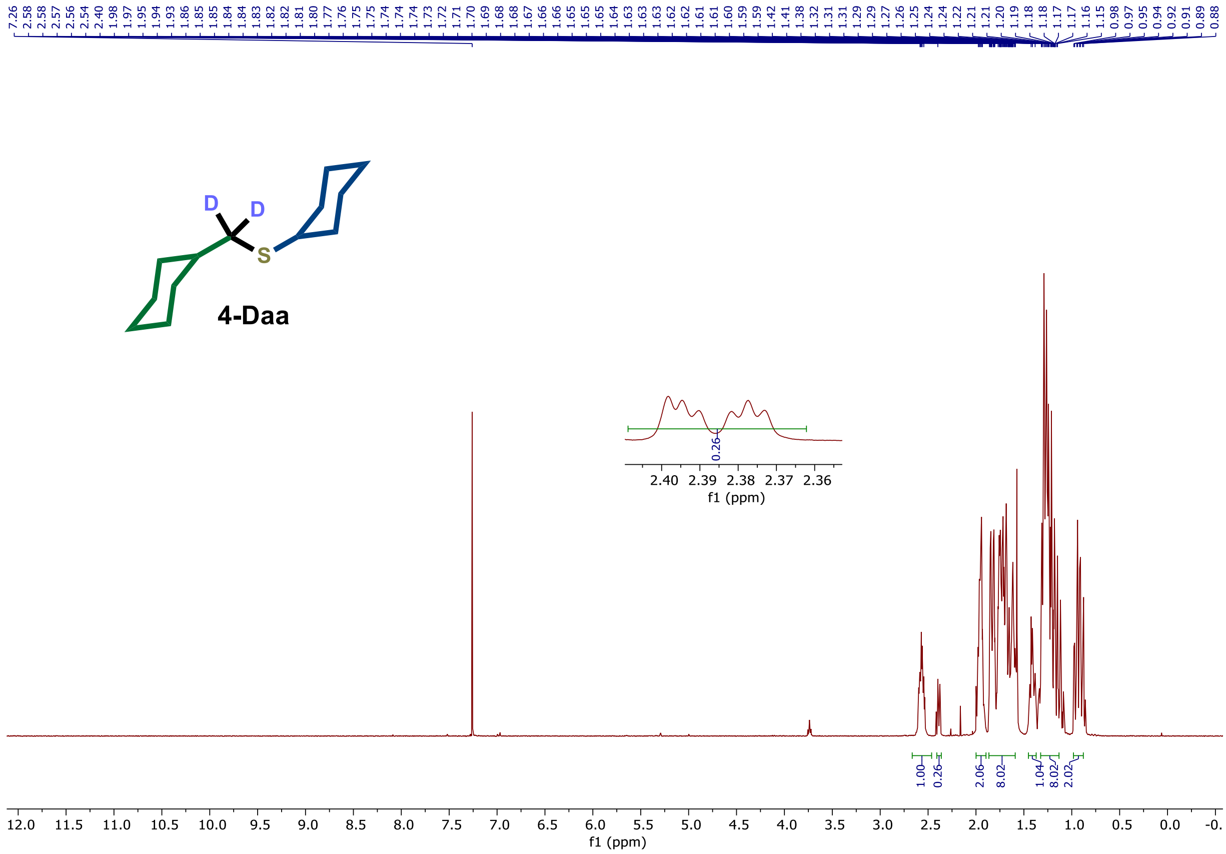

^{13}C NMR (100 MHz, CDCl$_3$) of **4-Daa**

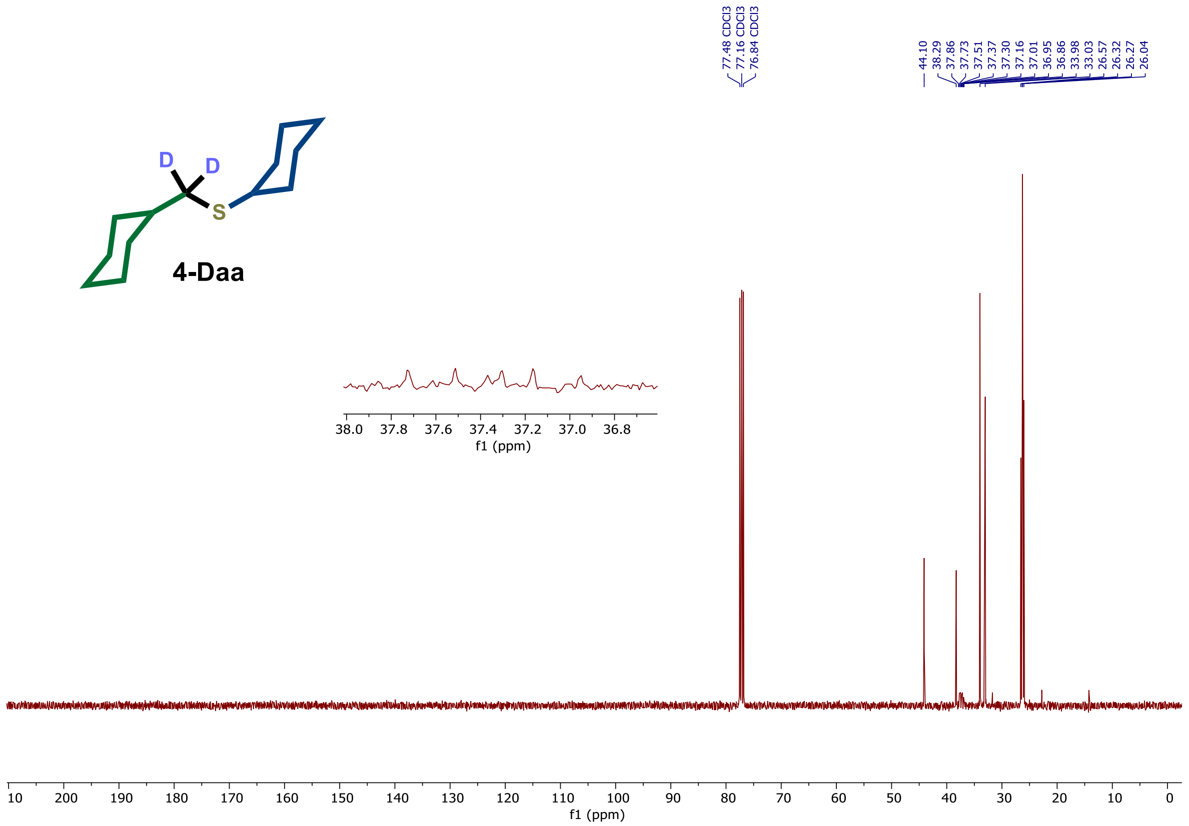

^{1}H NMR (400 MHz, CDCl$_3$) of **4-Dab**

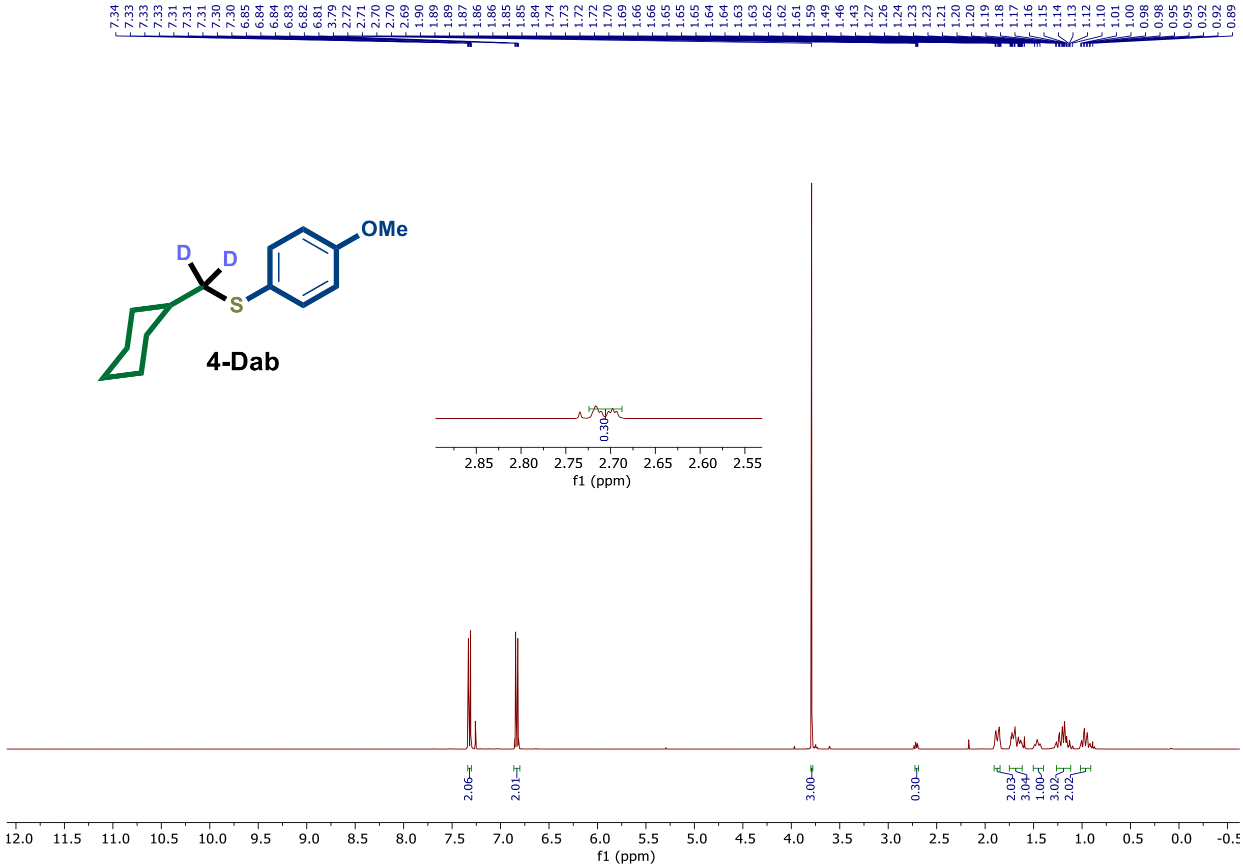

^{13}C NMR (100 MHz, $CDCl_3$) of **4-Dab**

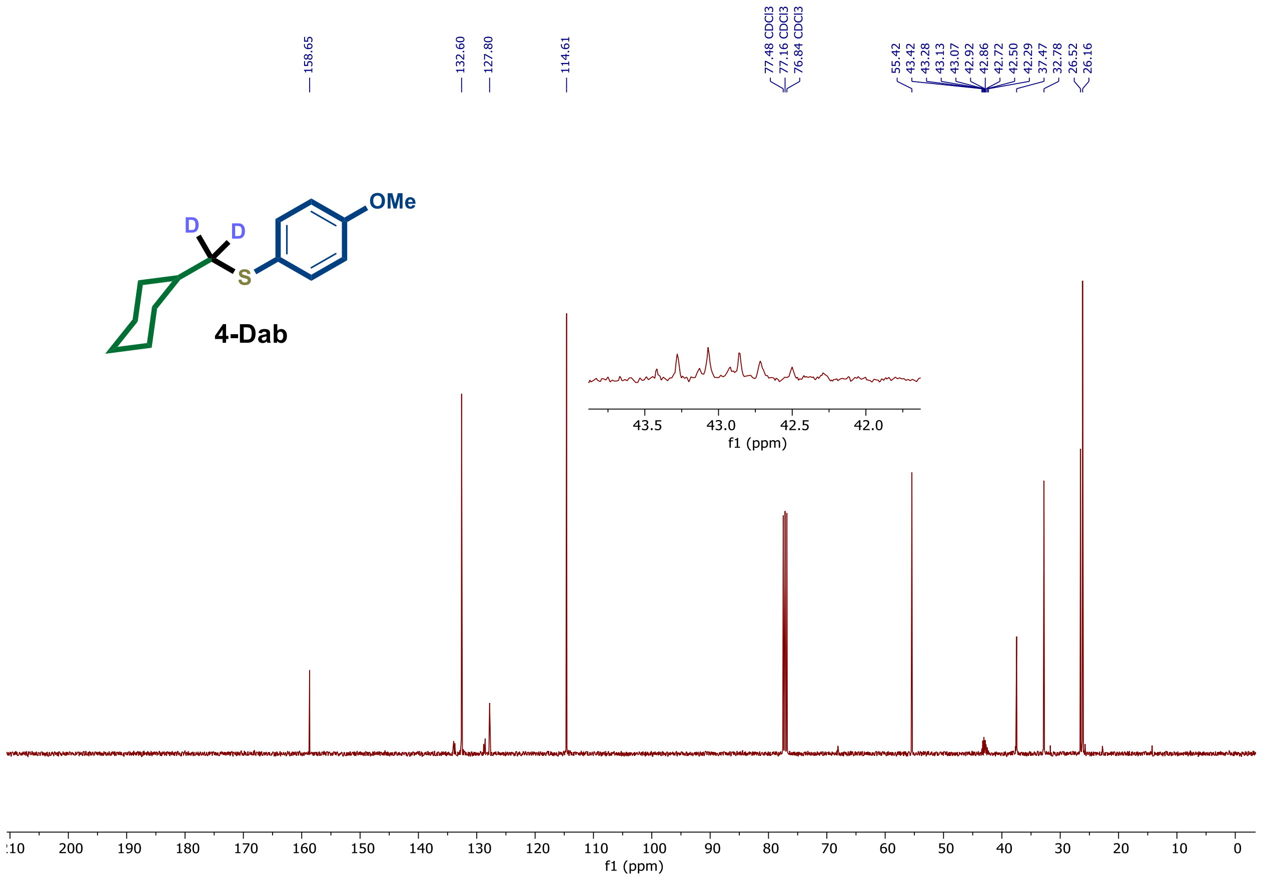

^{1}H NMR (400 MHz, $CDCl_3$) of **5a**

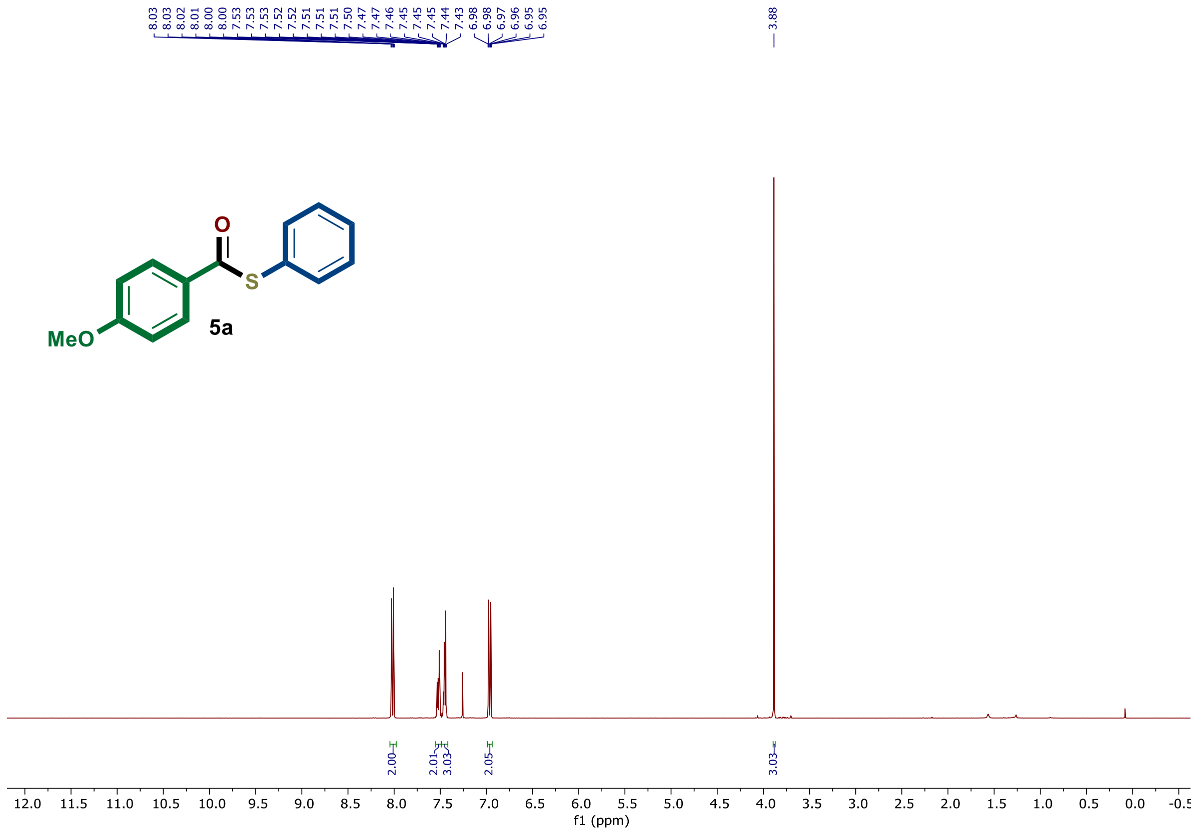

^{13}C NMR (100 MHz, $CDCl_3$) of **5a**

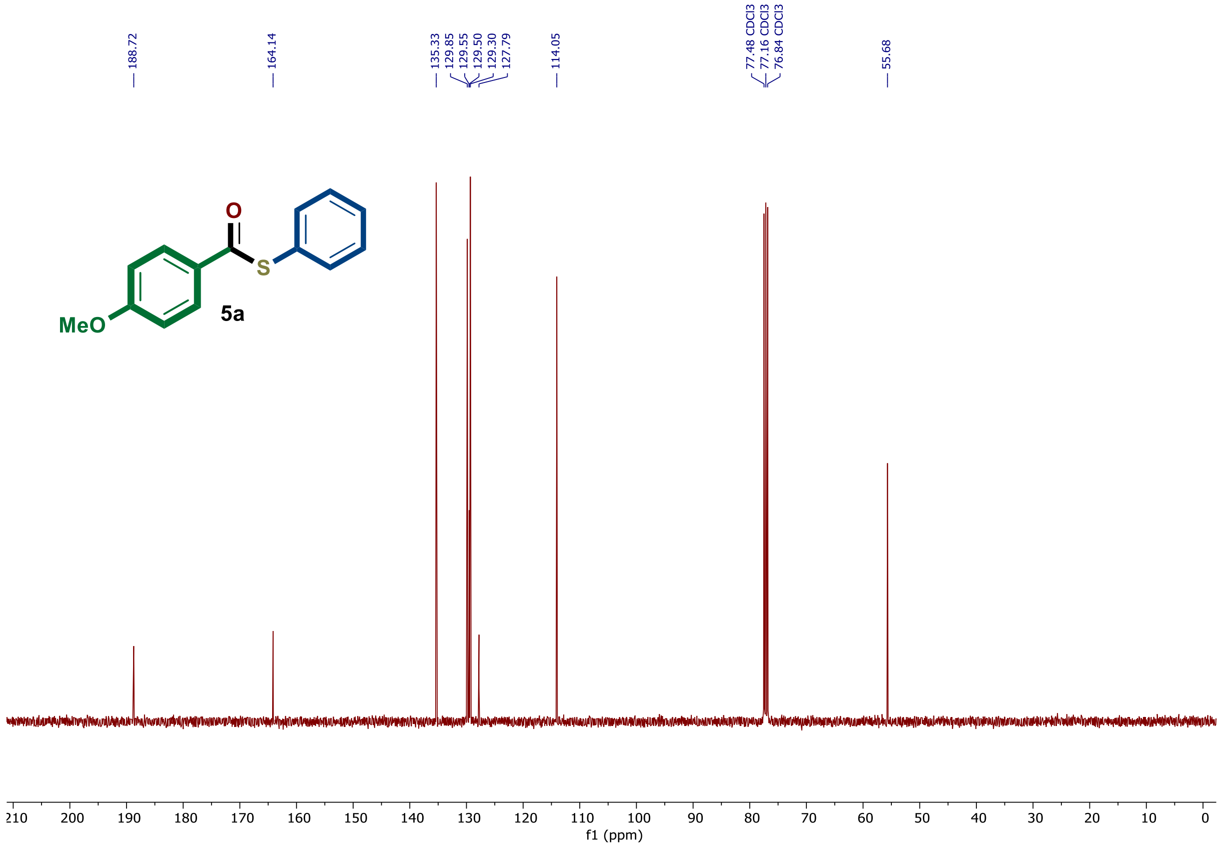

^{1}H NMR (400 MHz, $CDCl_3$) of **5b**

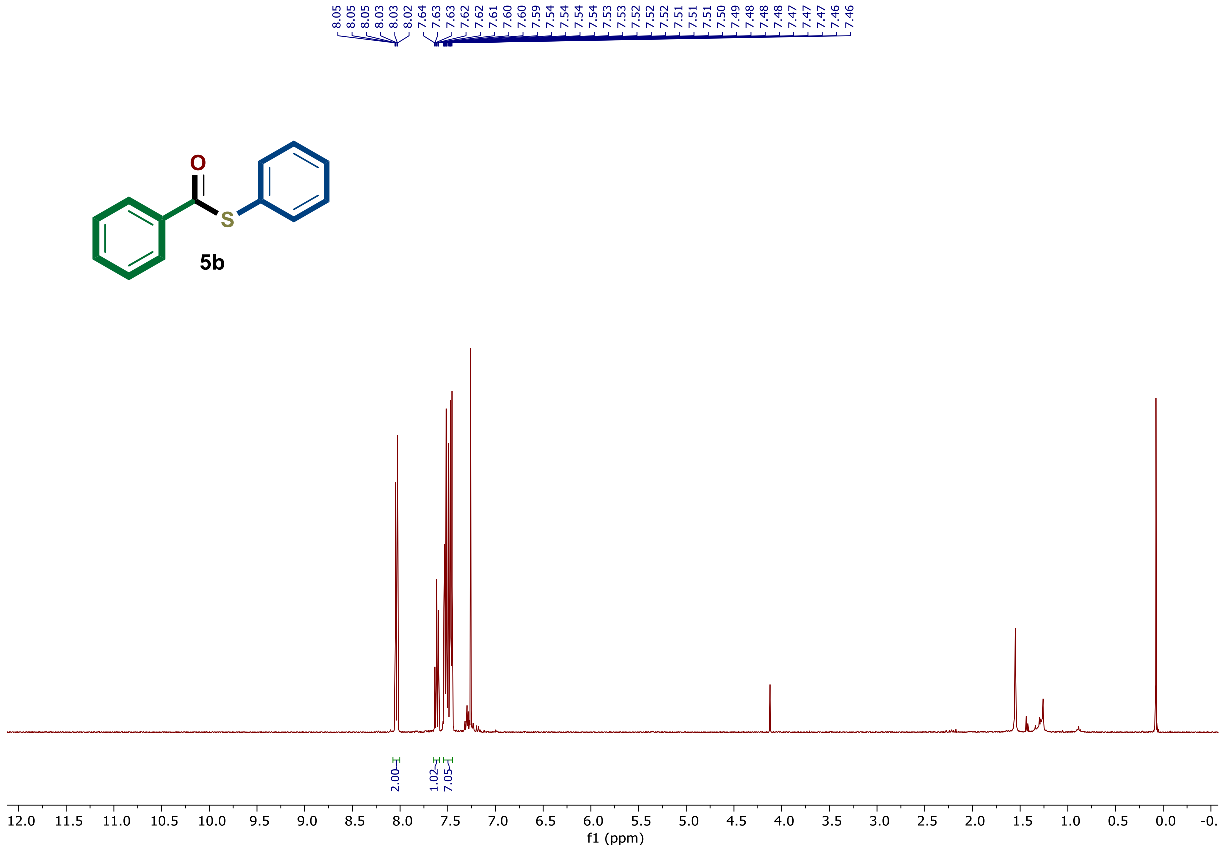

^{13}C NMR (100 MHz, $CDCl_3$) of **5b**

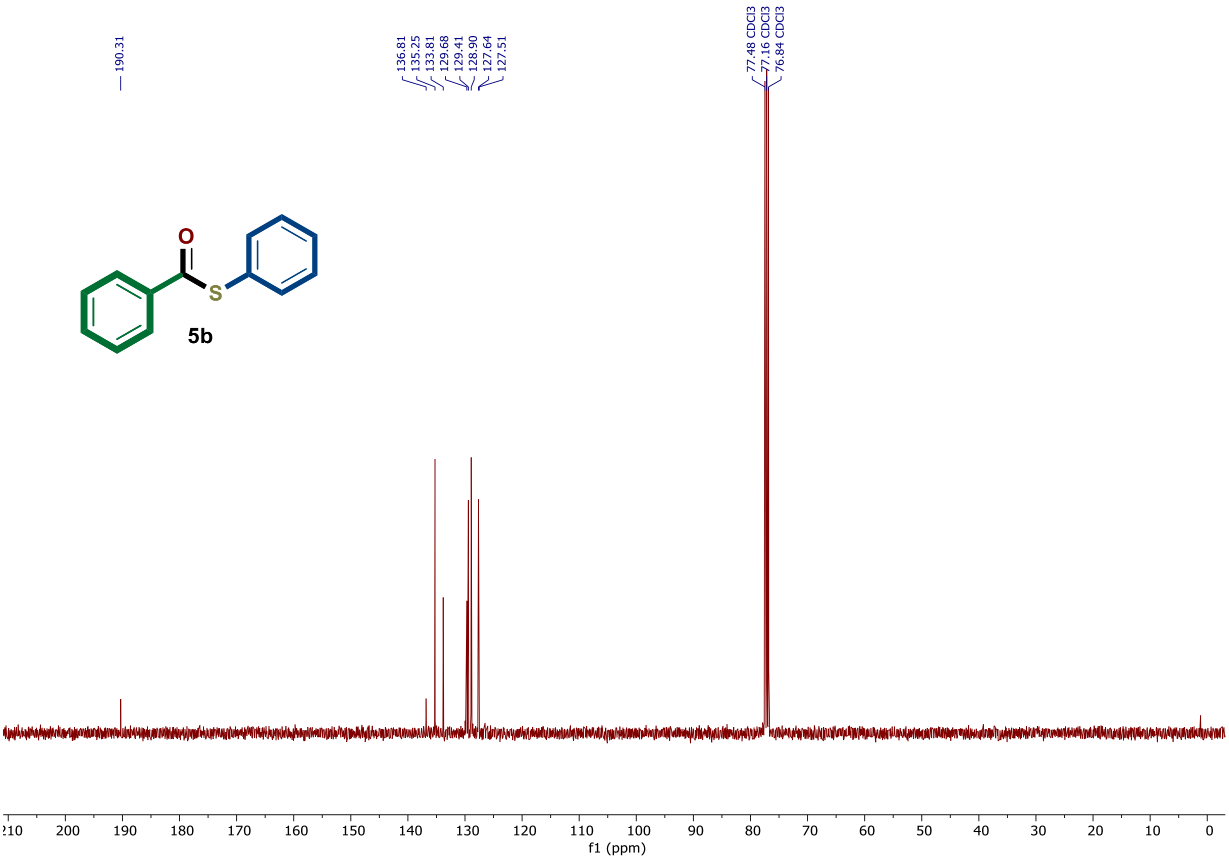

^{1}H NMR (400 MHz, $CDCl_3$) of **5c**

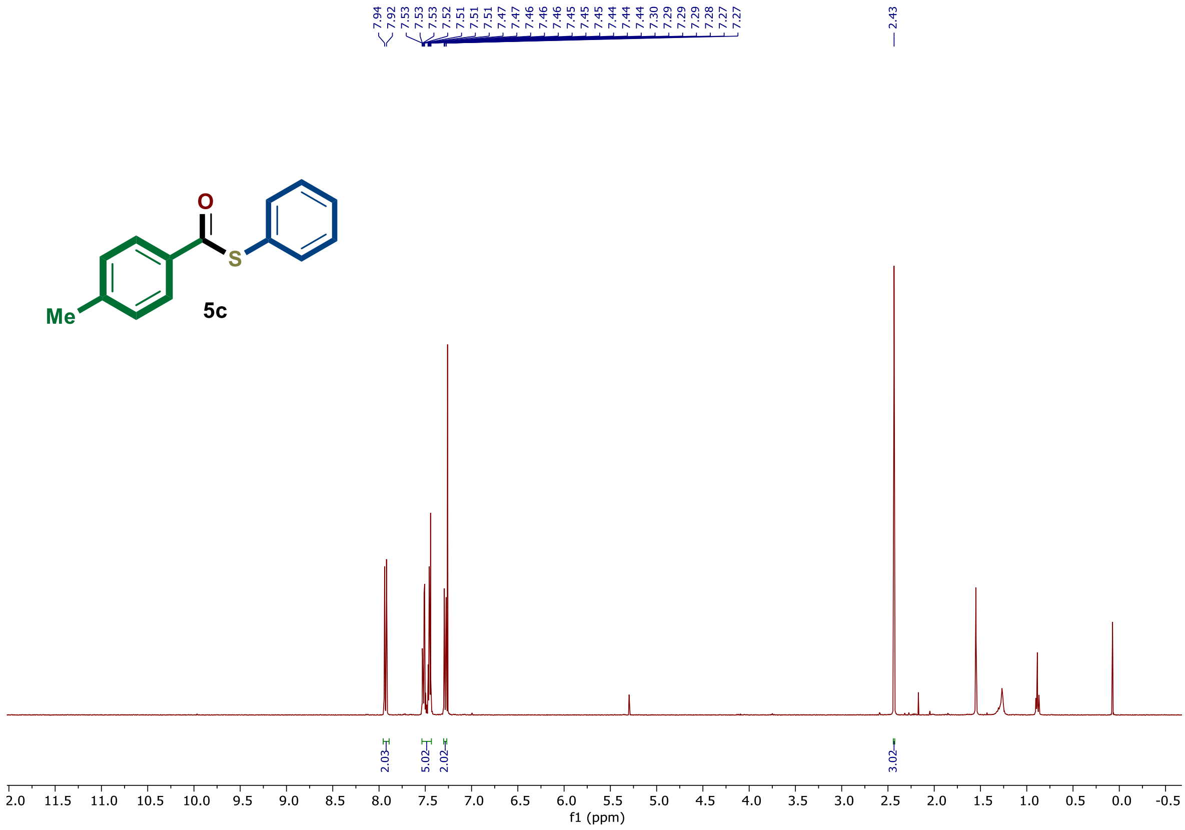

^{13}C NMR (100 MHz, $CDCl_3$) of **5c**

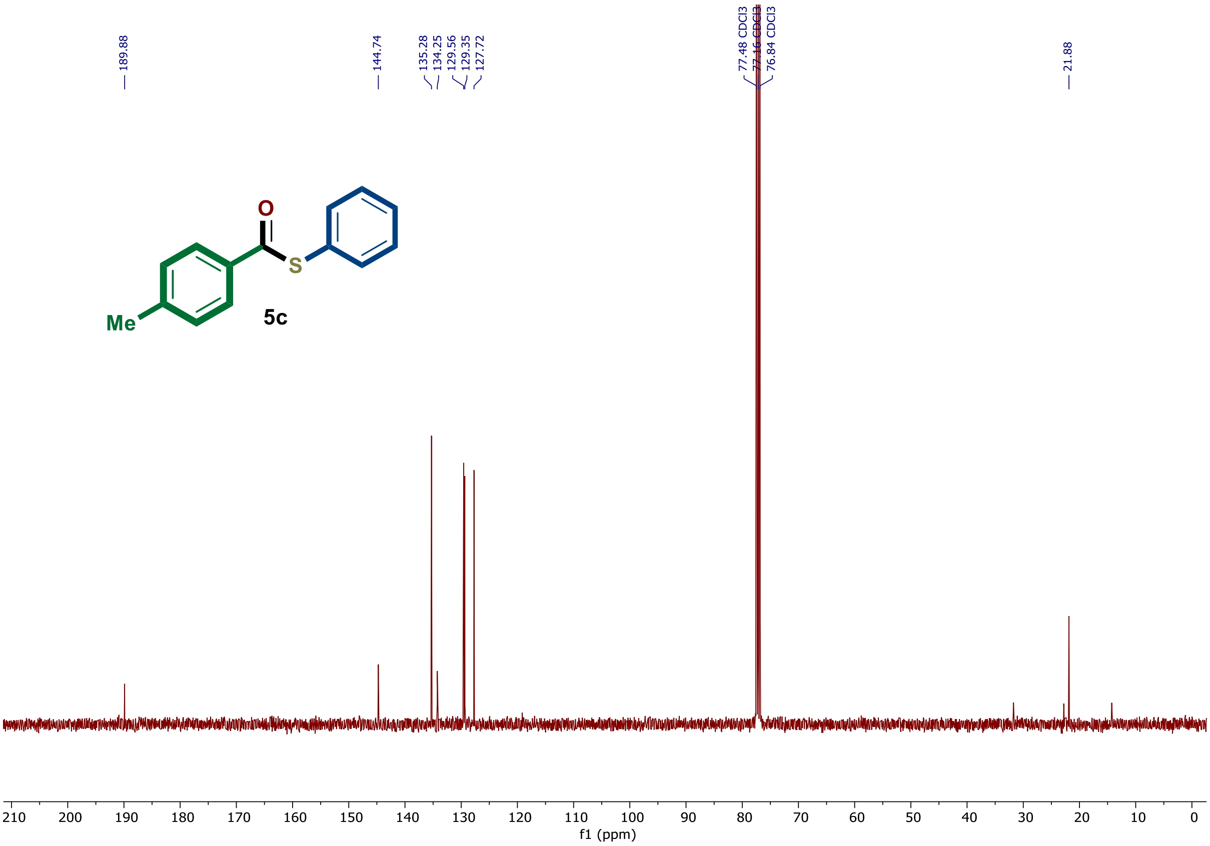

^{1}H NMR (400 MHz, $CDCl_3$) of **5d**

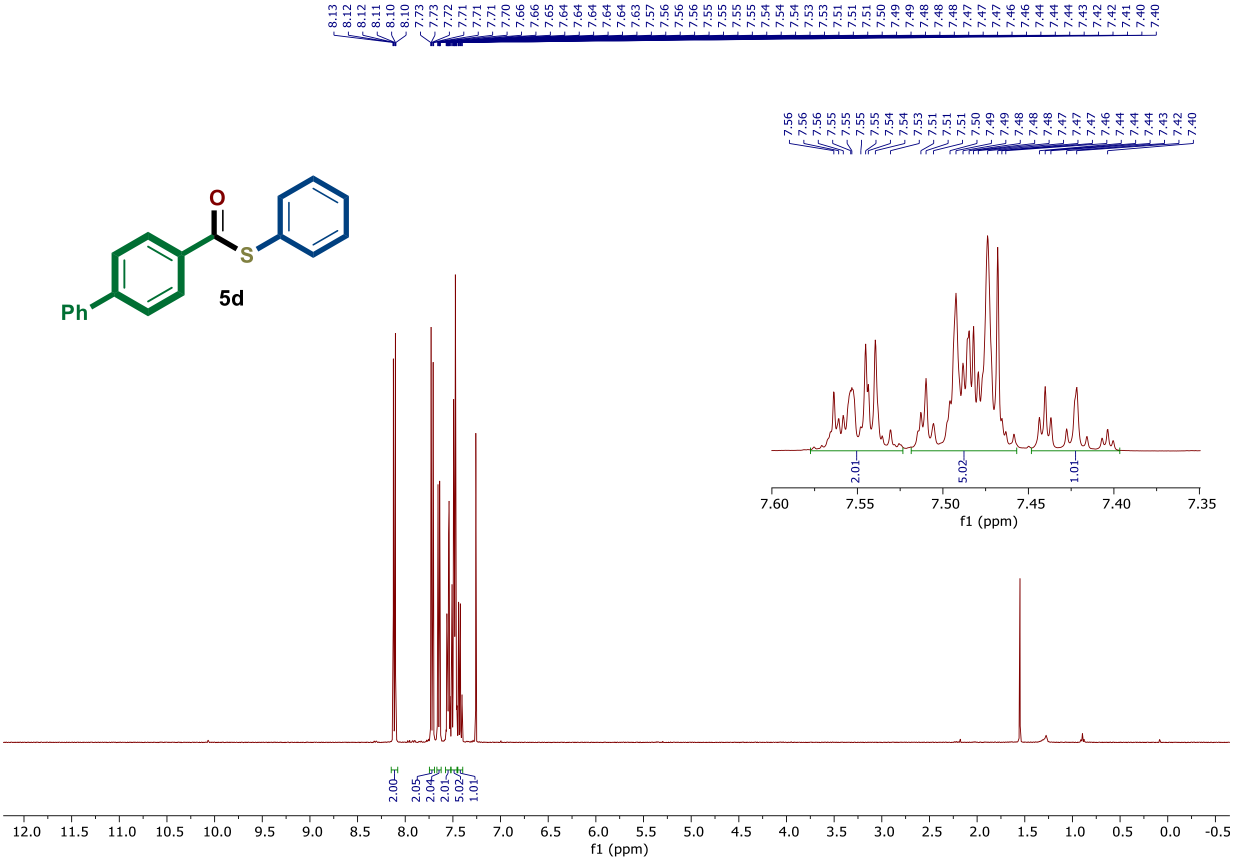

^{13}C NMR (100 MHz, $CDCl_3$) of **5d**

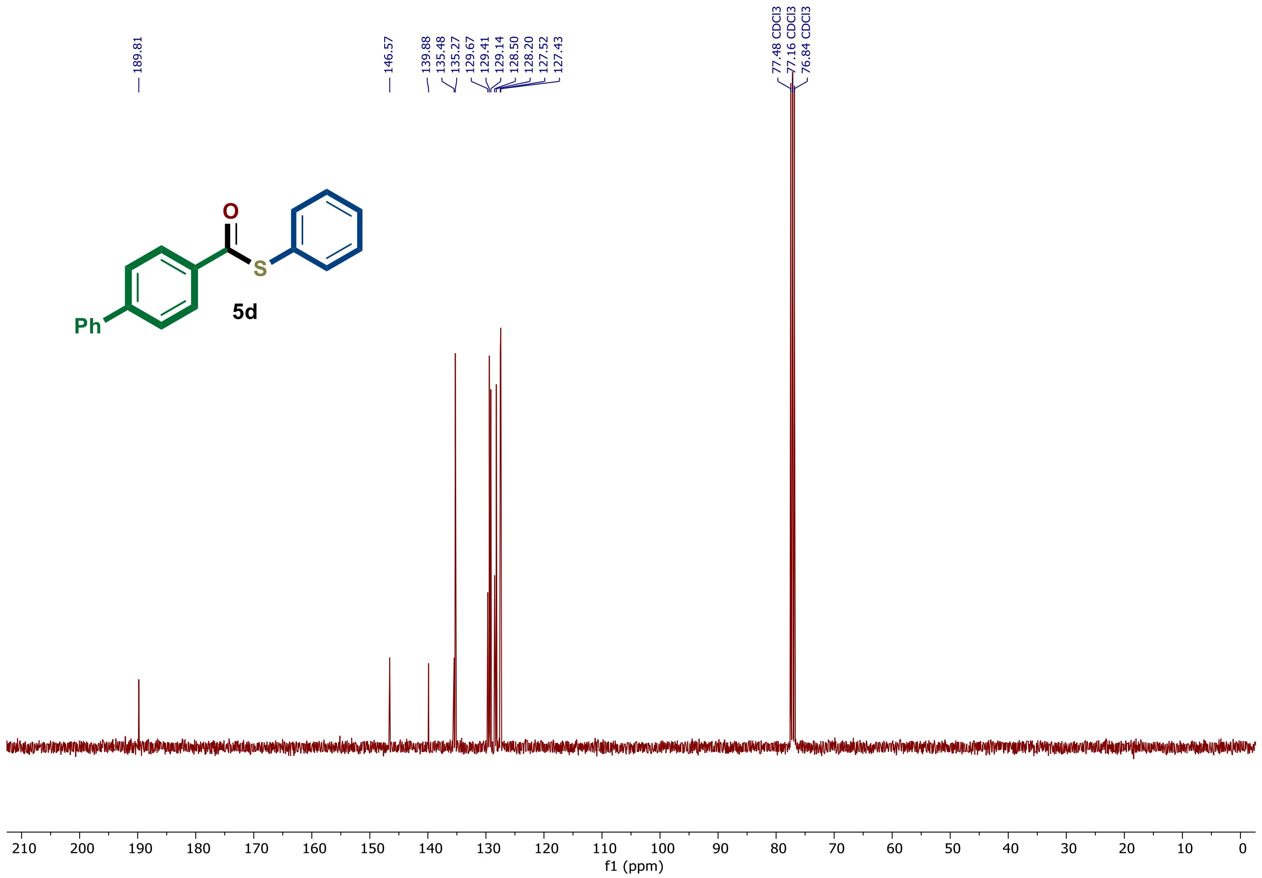

^{1}H NMR (400 MHz, $CDCl_3$) of **5j**

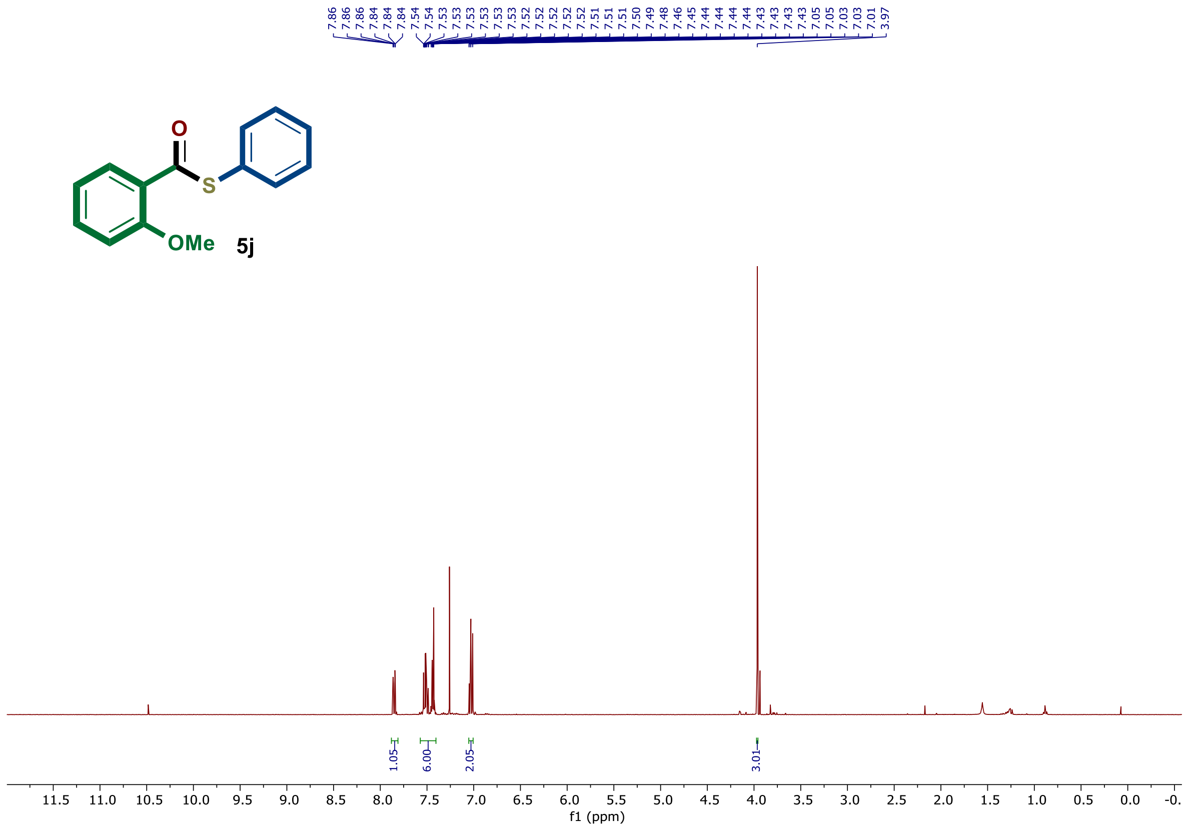

^{13}C NMR (100 MHz, $CDCl_3$) of **5j**

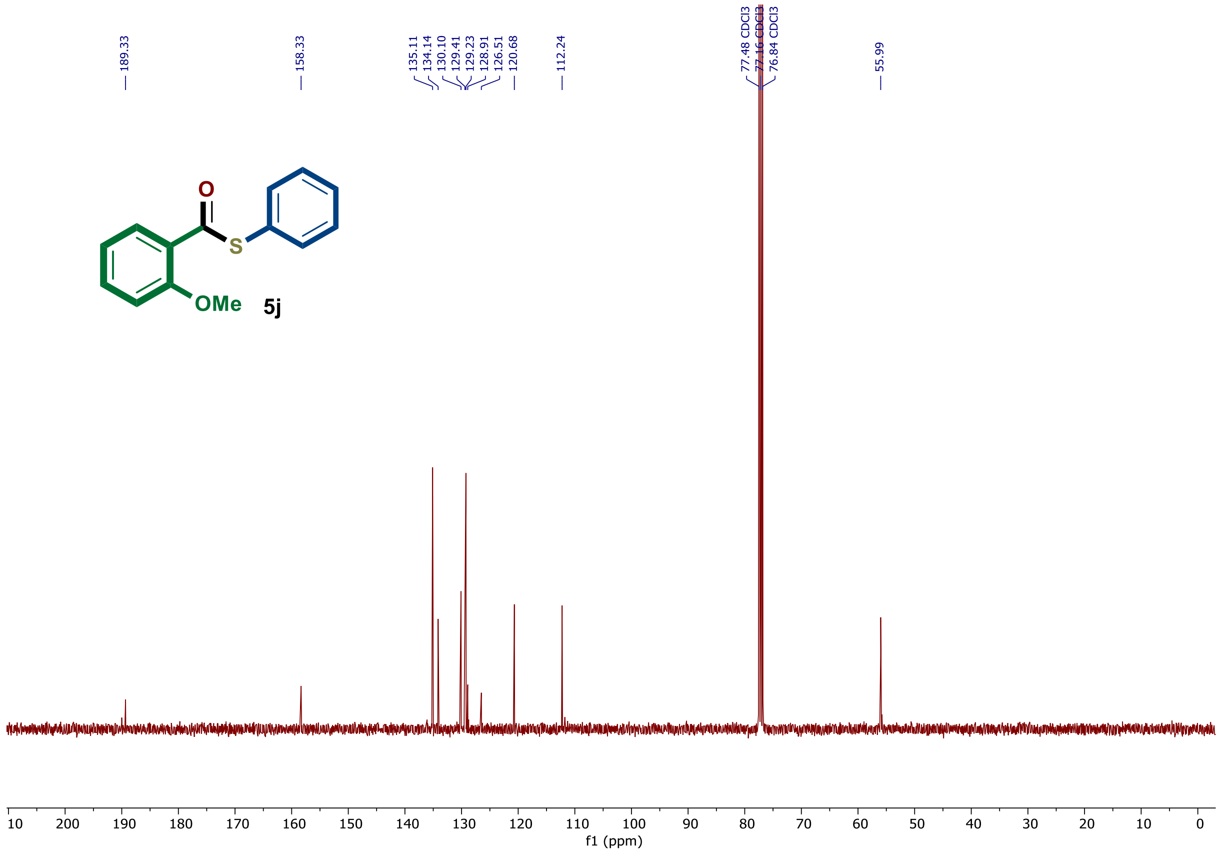

^{1}H NMR (400 MHz, $CDCl_3$) of **5n**

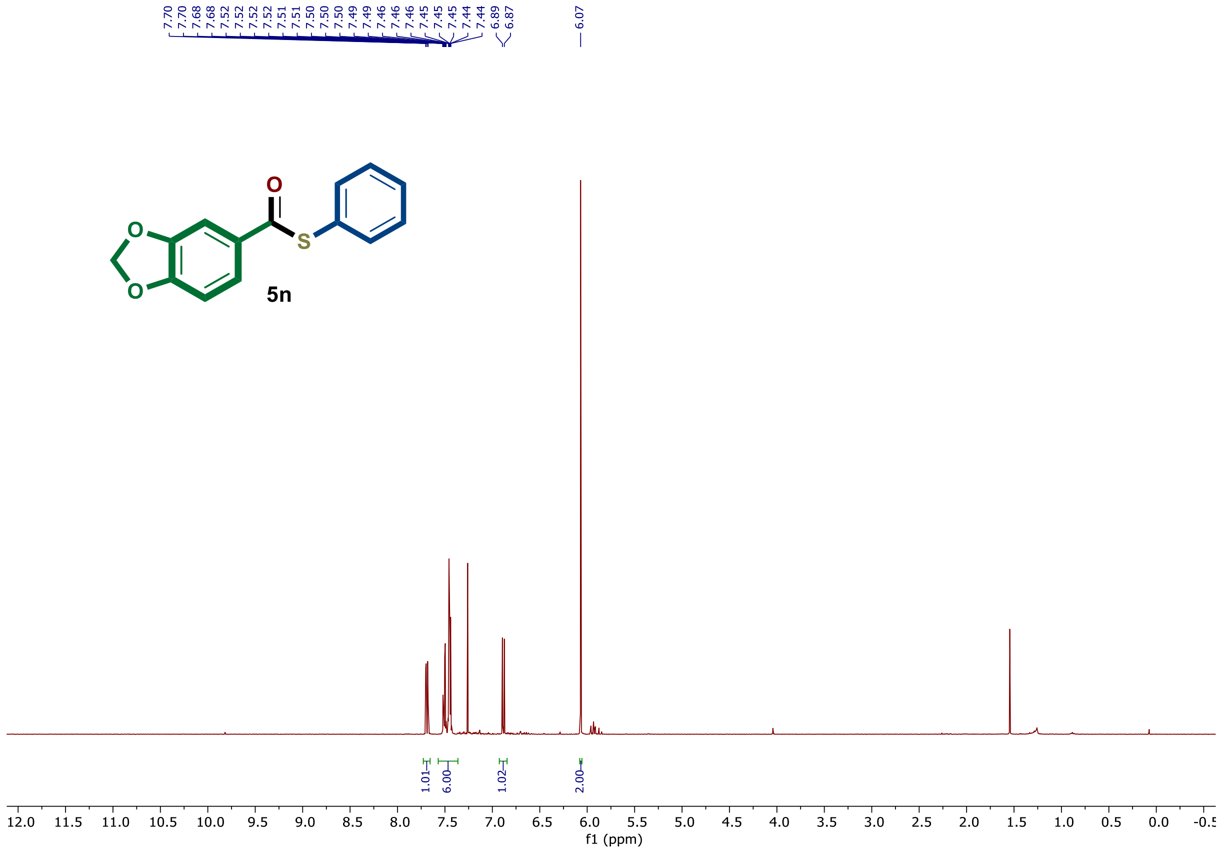

^{13}C NMR (100 MHz, $CDCl_3$) of **5n**

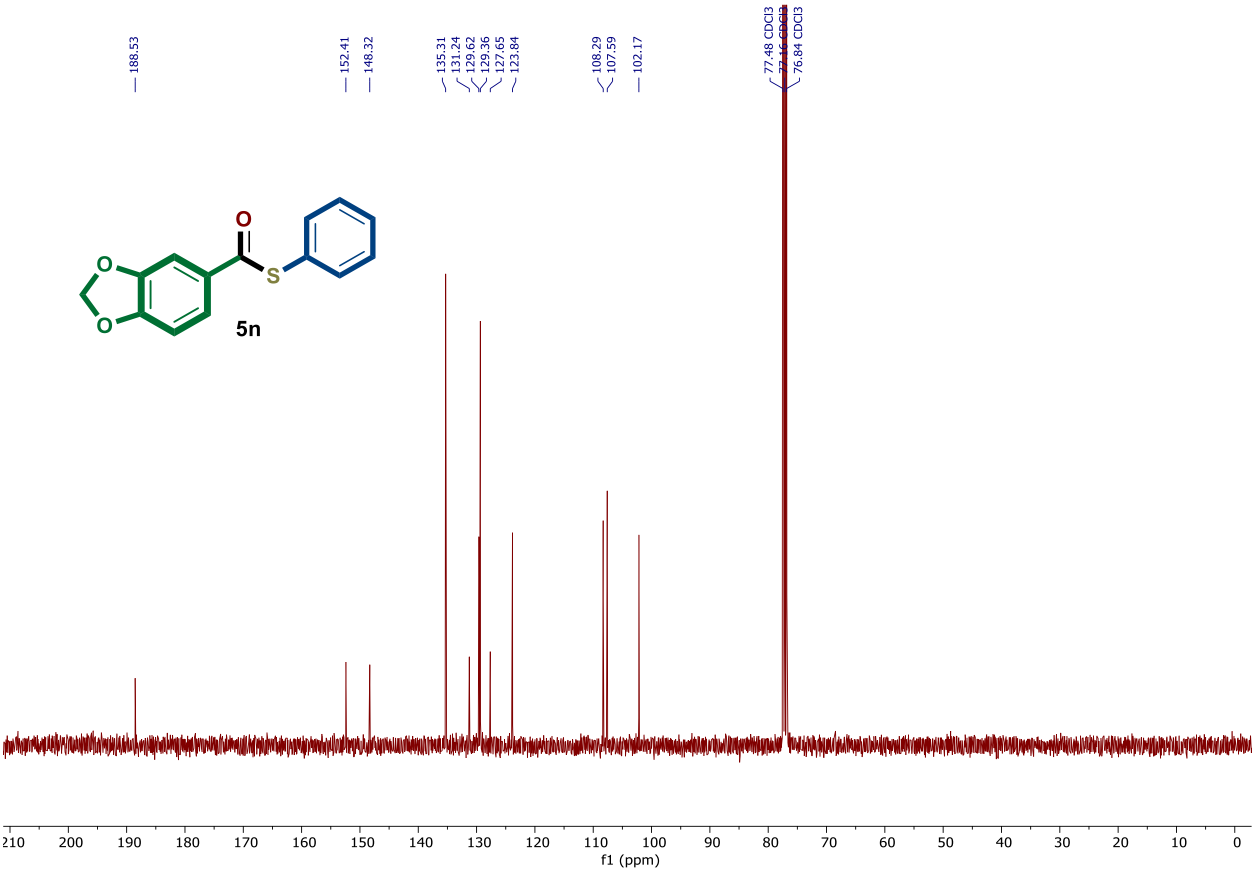

^{1}H NMR (400 MHz, $CDCl_3$) of **5s**

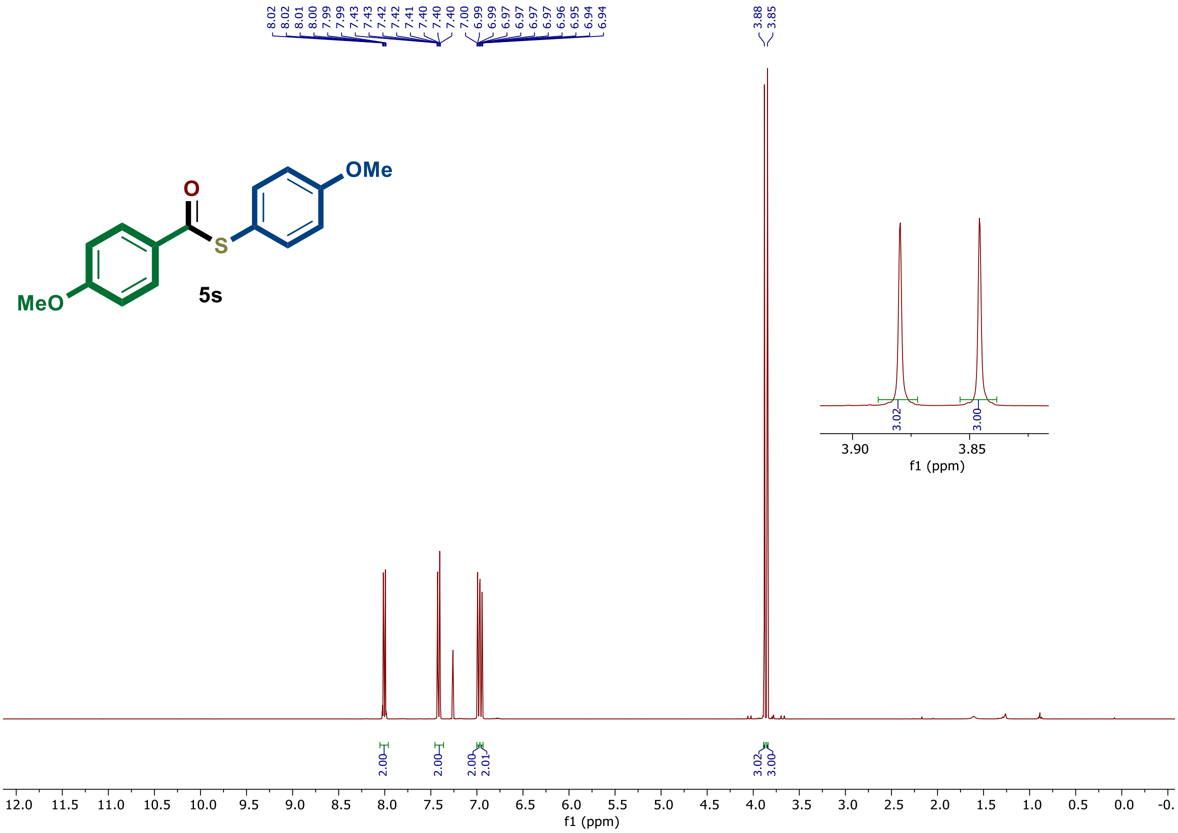

^{13}C NMR (100 MHz, CDCl$_3$) of **5s**

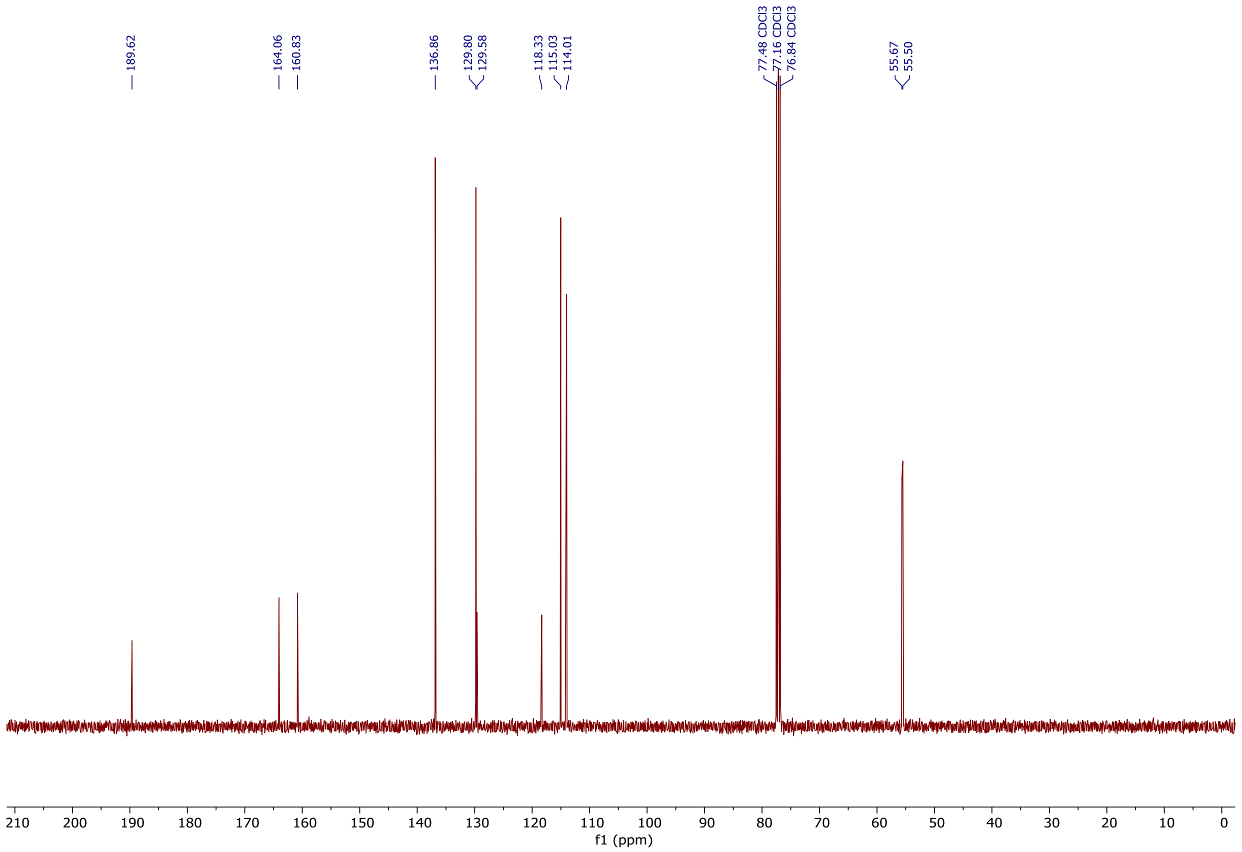

^{1}H NMR (400 MHz, CDCl$_3$) of **5u**

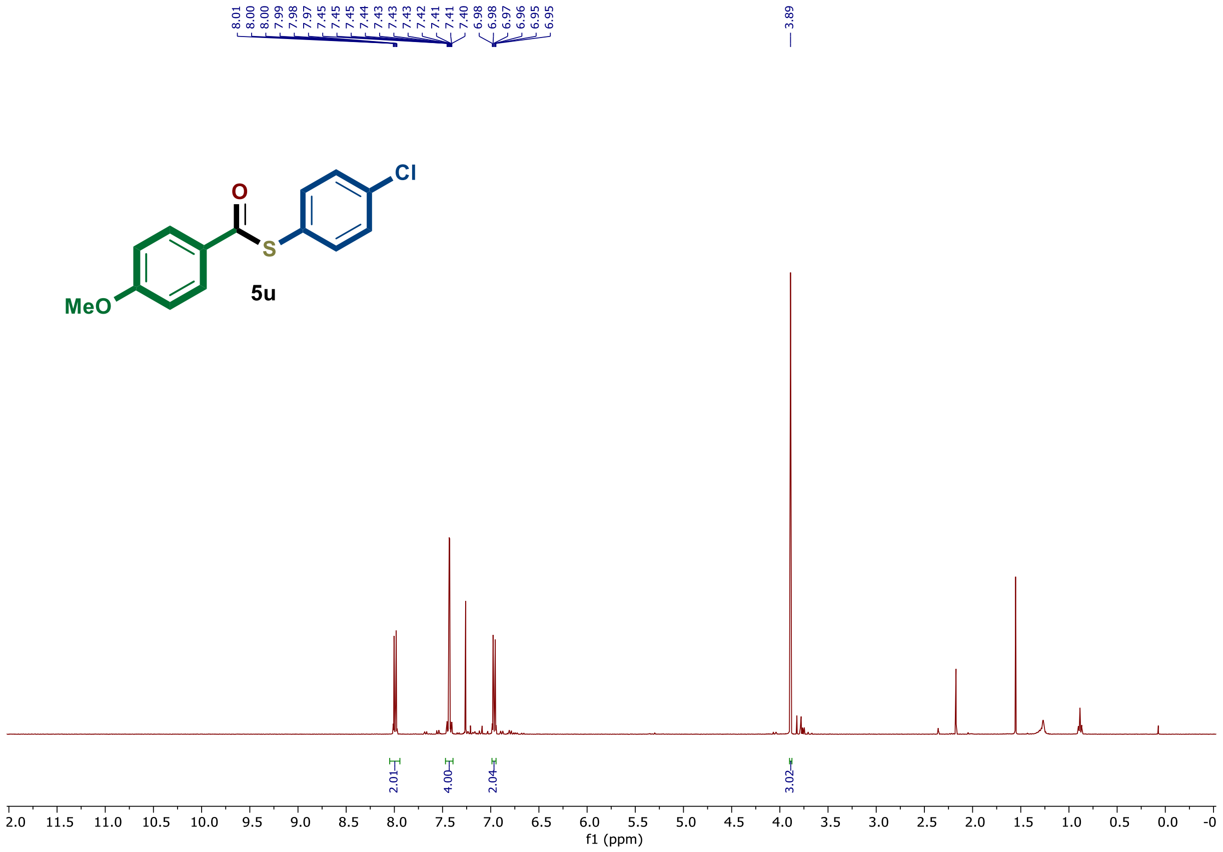

^{13}C NMR (100 MHz, $CDCl_3$) of **5u**

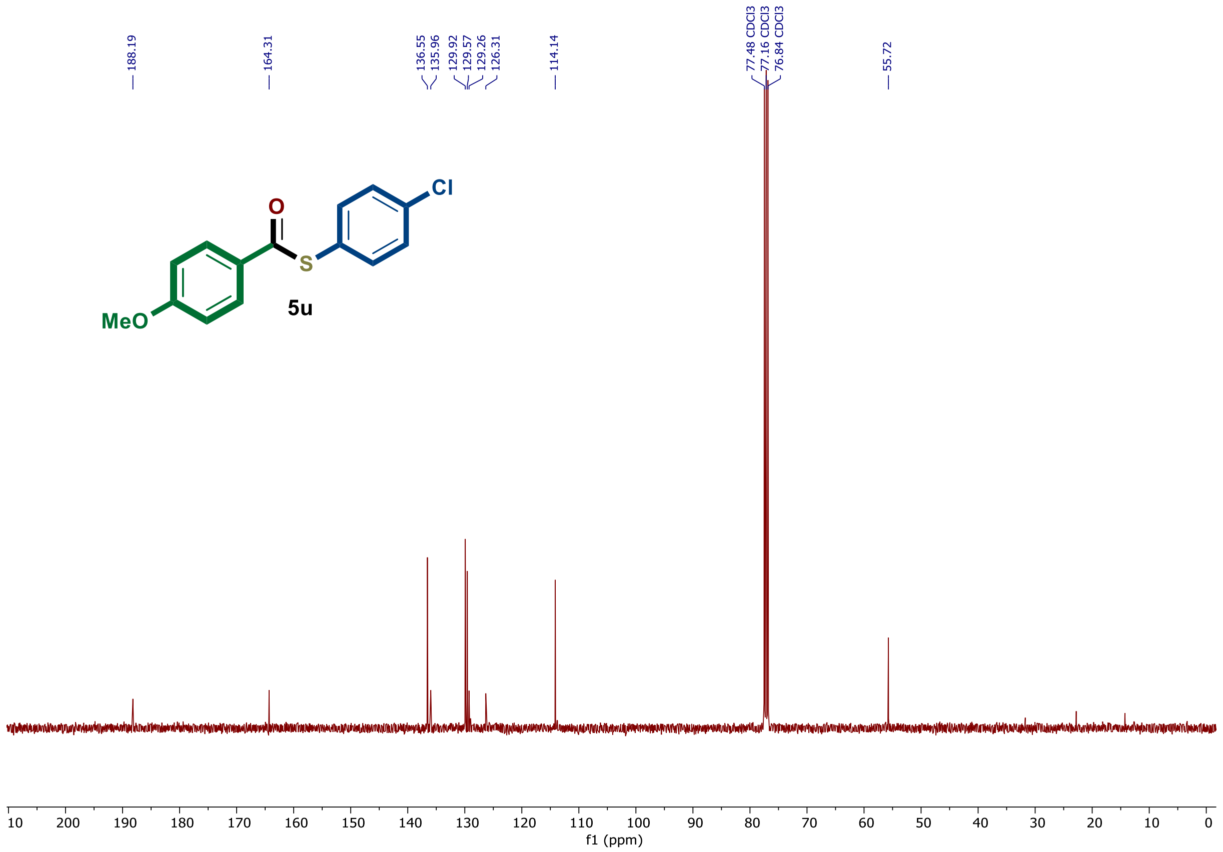

^{1}H NMR (400 MHz, $CDCl_3$) of **6a**

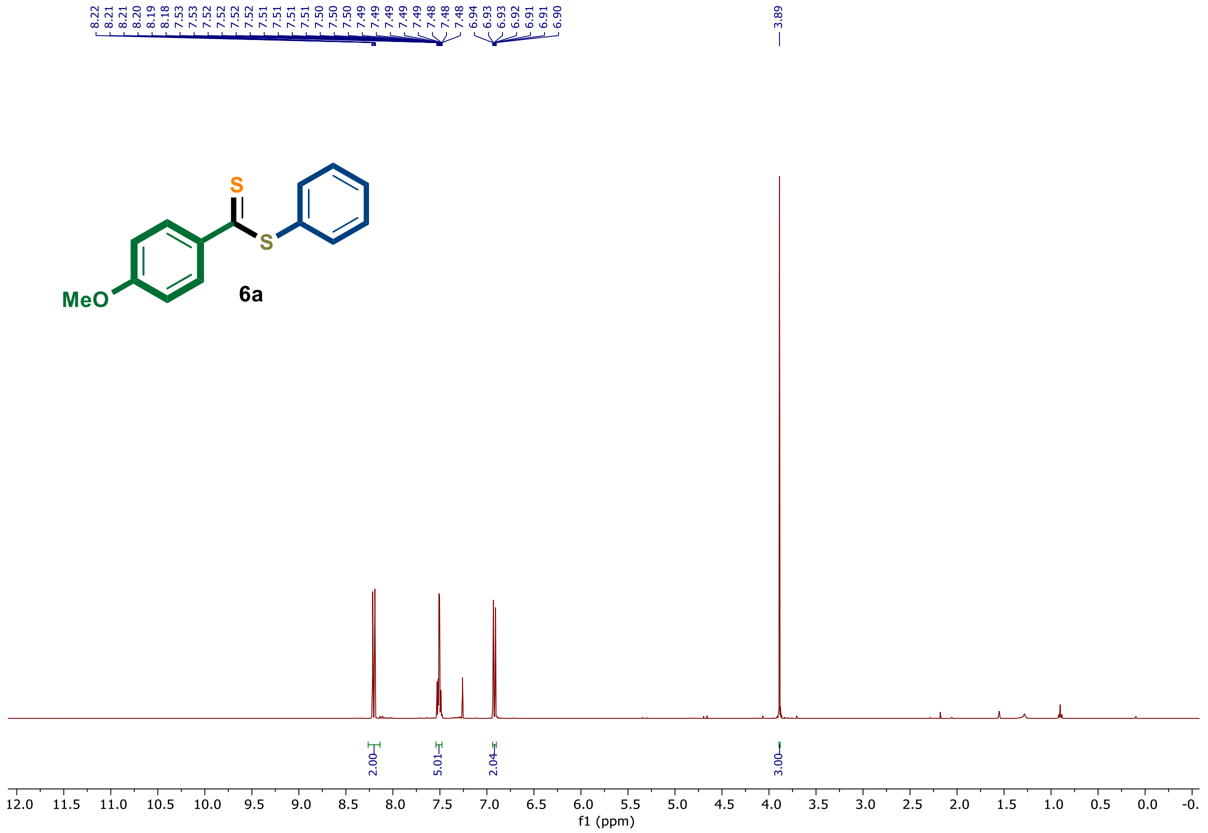

^{13}C NMR (100 MHz, $CDCl_3$) of **6a**

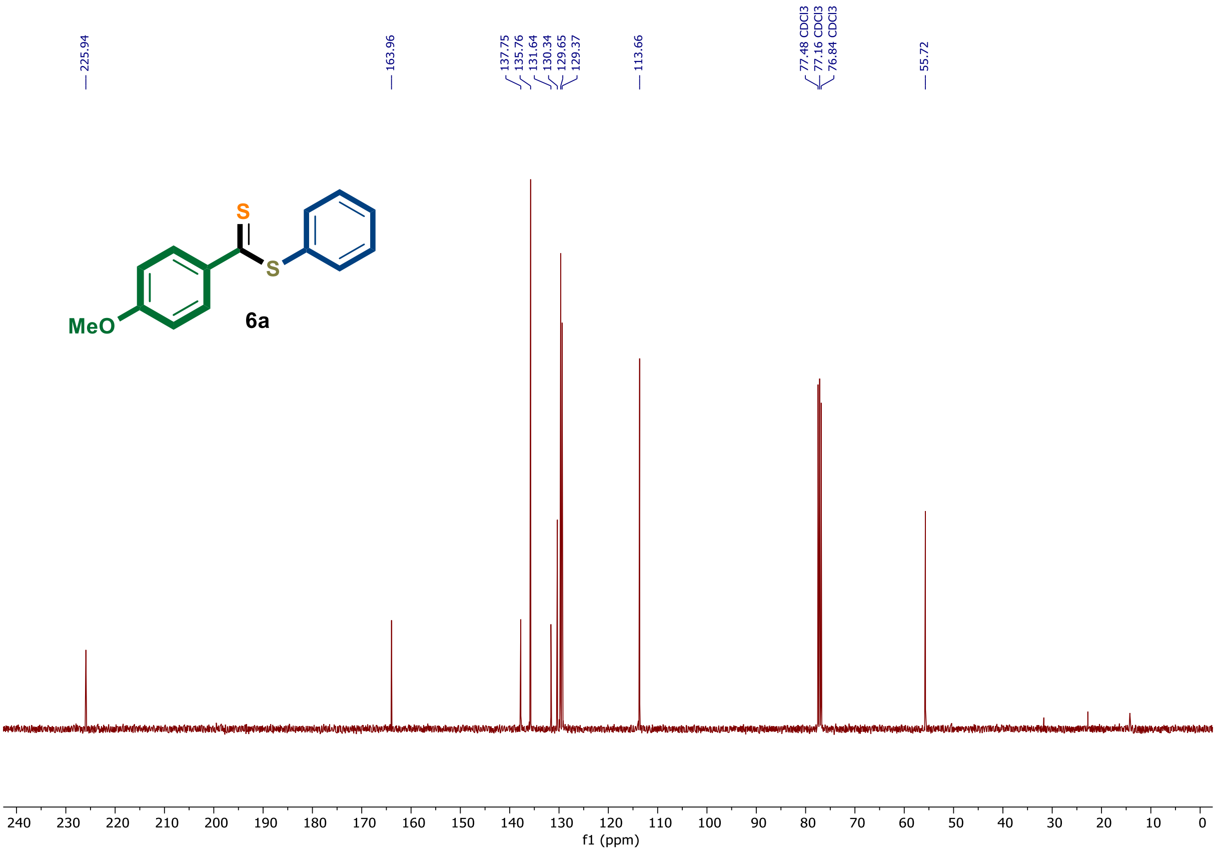

^{1}H NMR (400 MHz, $CDCl_3$) of **6b**

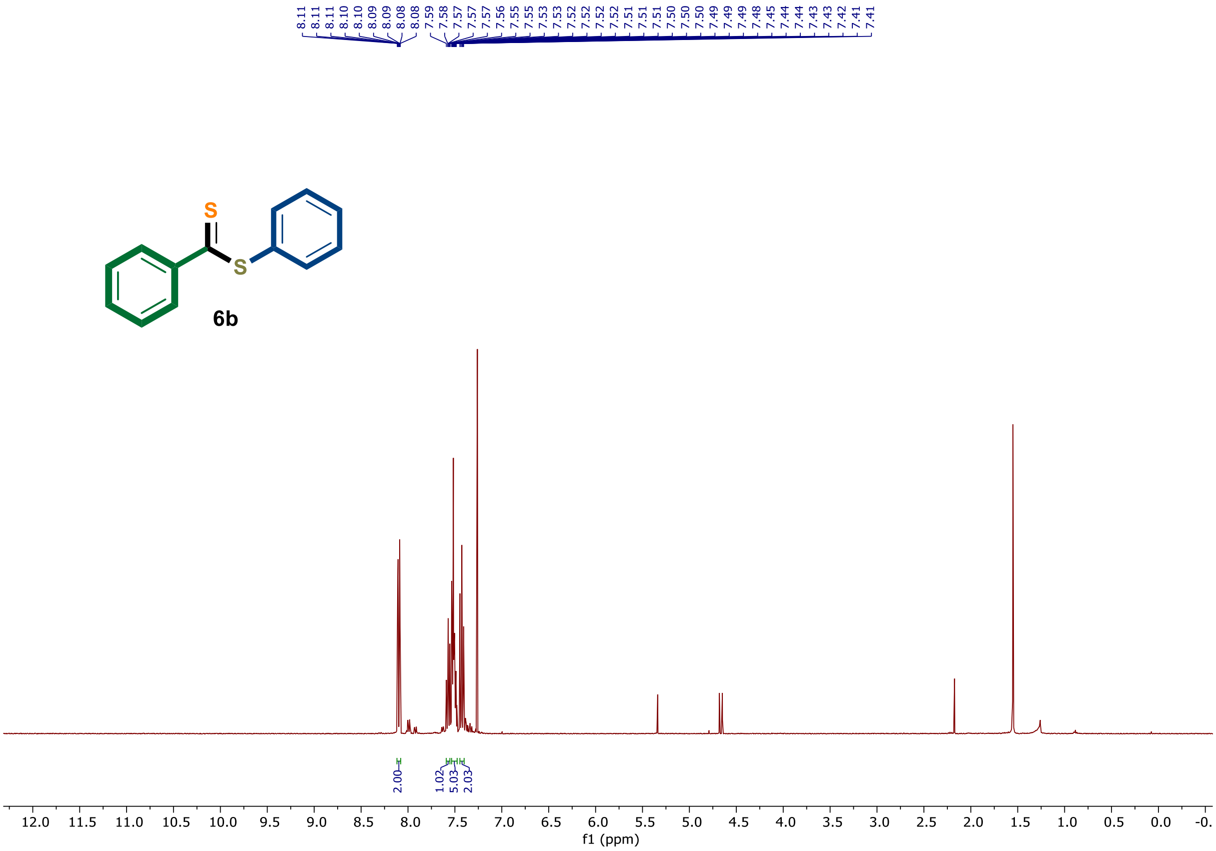

^{13}C NMR (100 MHz, $CDCl_3$) of **6b**

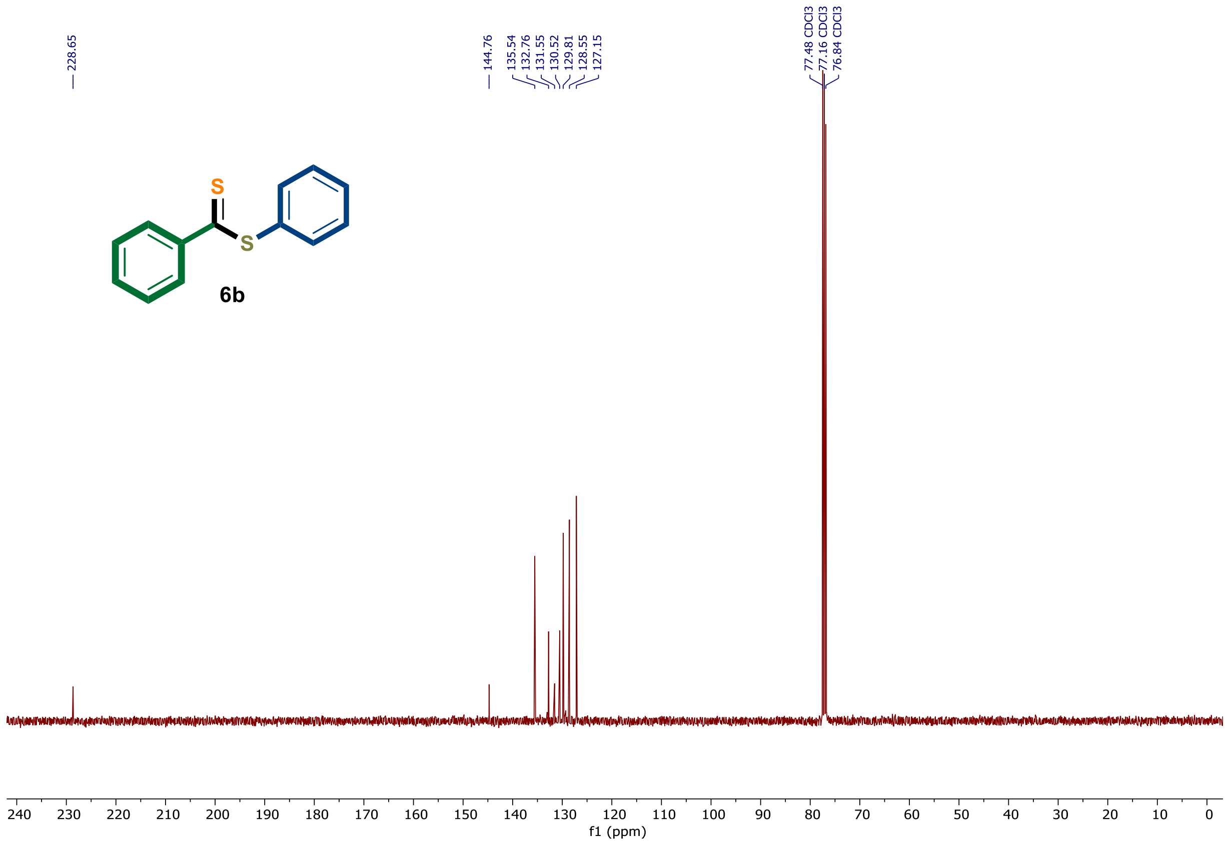

^{1}H NMR (400 MHz, $CDCl_3$) of **6c**

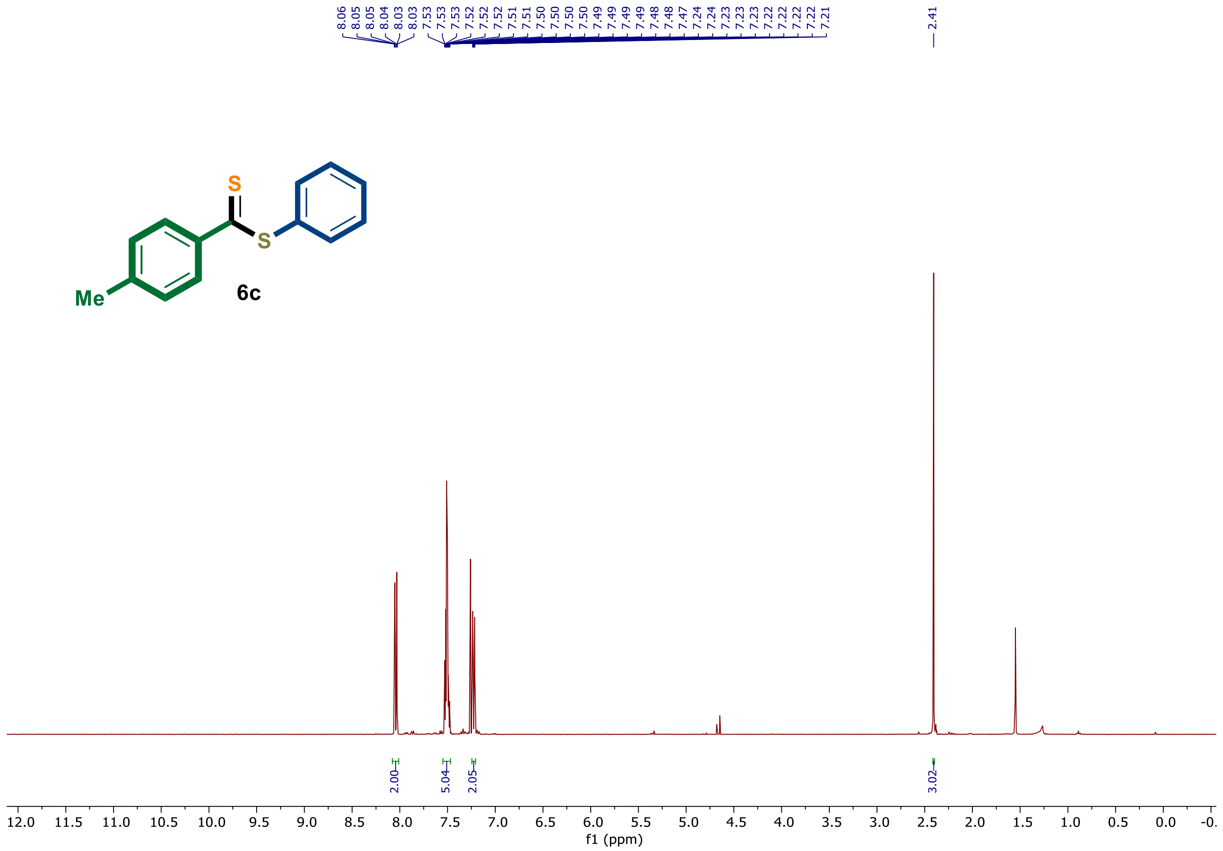

^{13}C NMR (100 MHz, $CDCl_3$) of **6c**

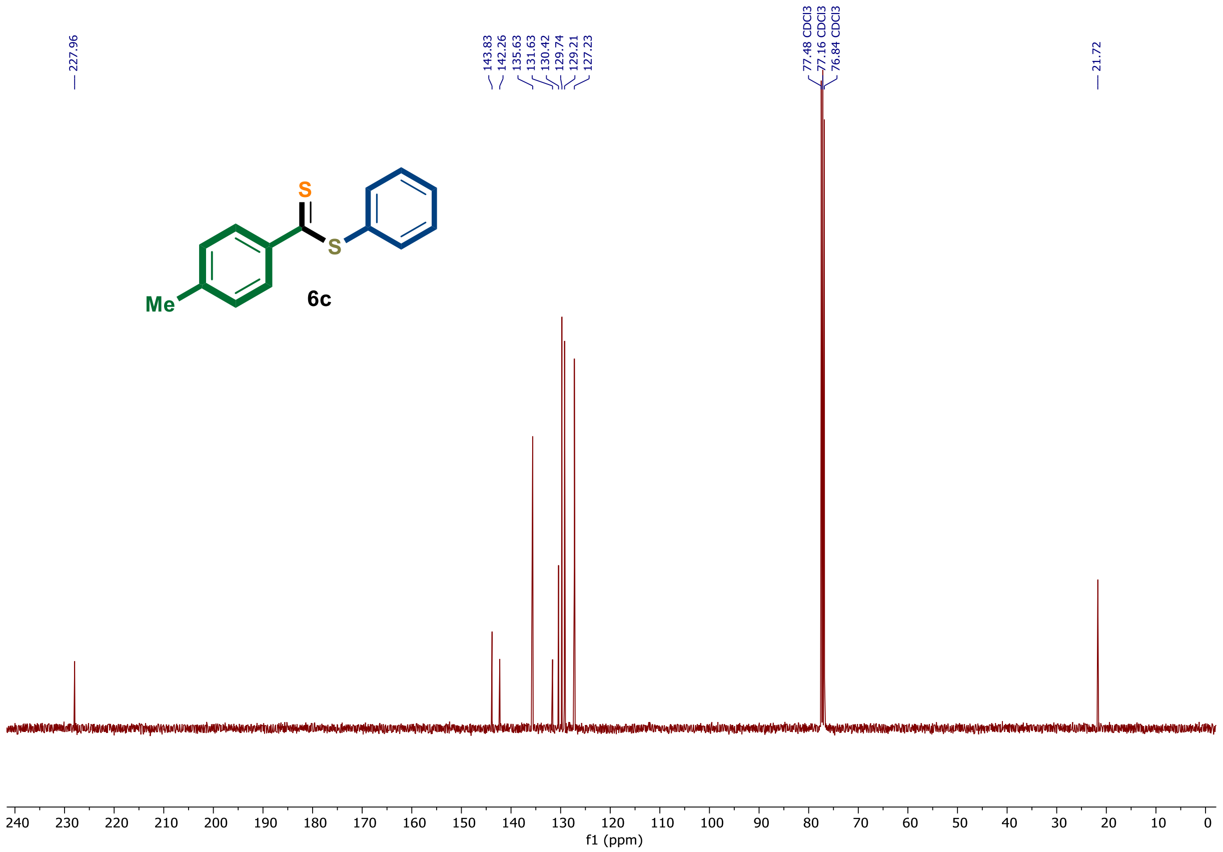

^{1}H NMR (400 MHz, $CDCl_3$) of **6m**

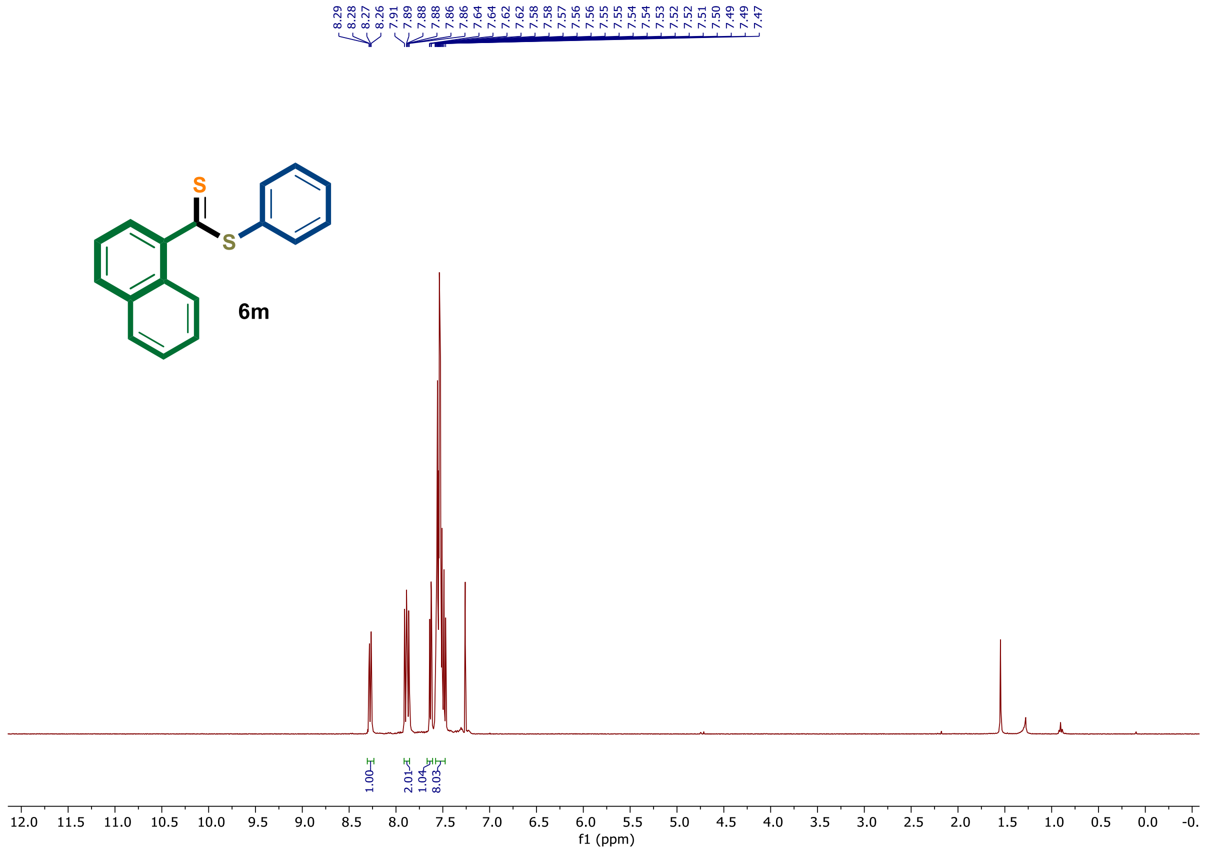

^{13}C NMR (100 MHz, $CDCl_3$) of **6m**

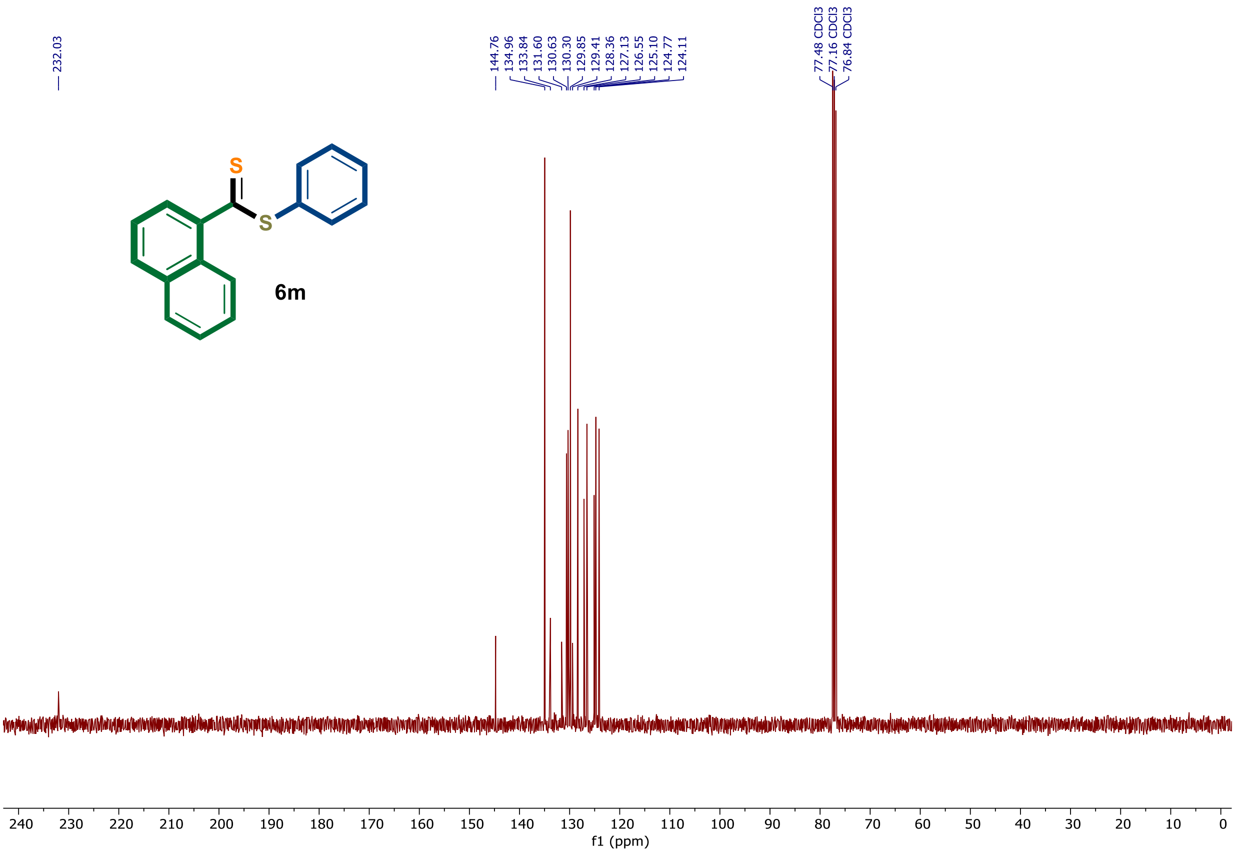

^{1}H NMR (400 MHz, $CDCl_3$) of **6n**

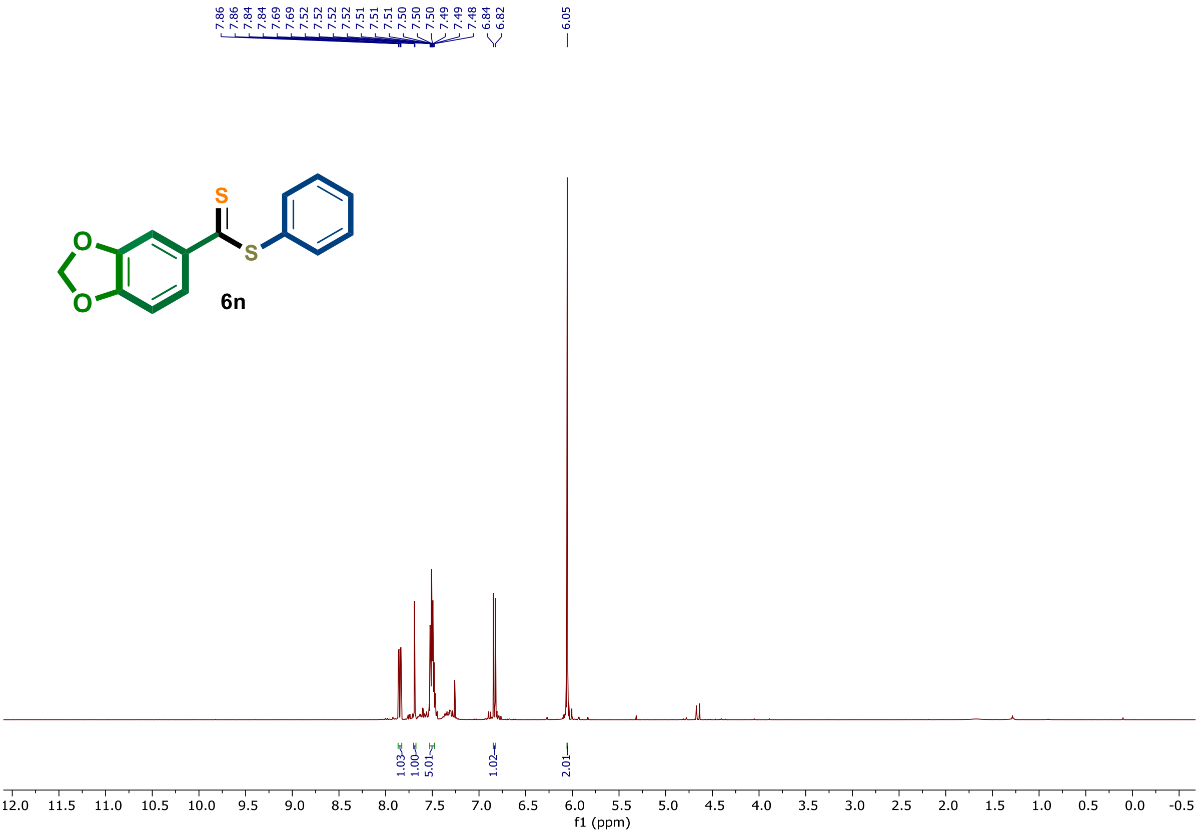

^{13}C NMR (100 MHz, $CDCl_3$) of **6n**

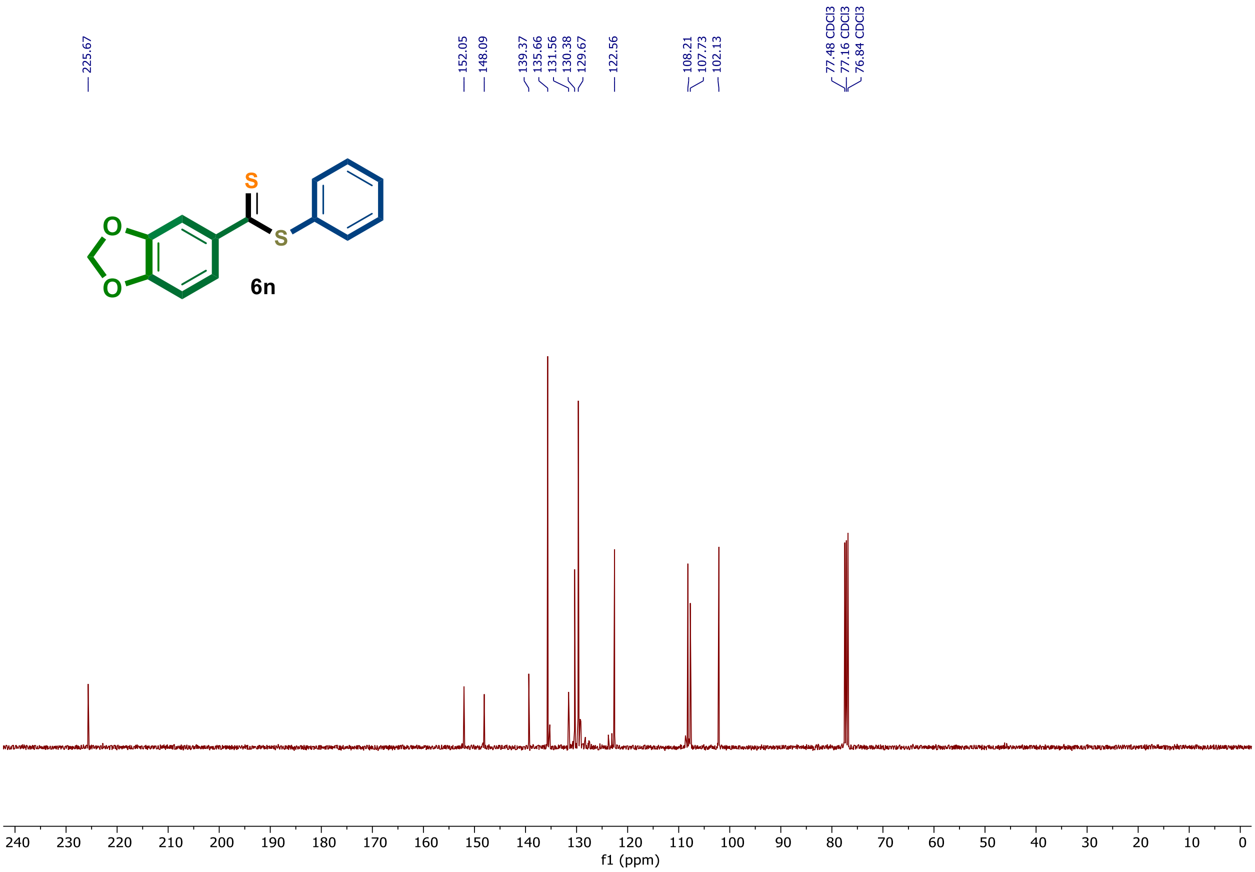

^{1}H NMR (400 MHz, $CDCl_3$) of **6s**

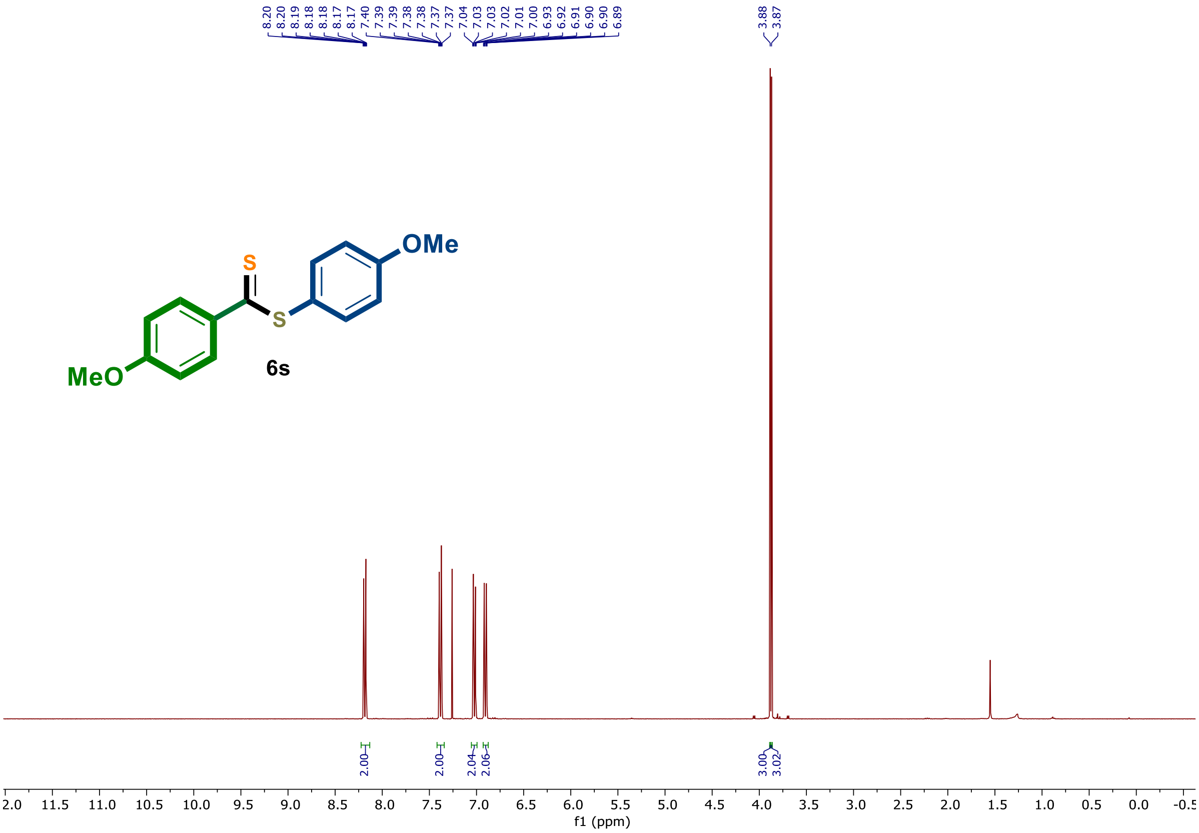

^{13}C NMR (100 MHz, $CDCl_3$) of **6s**

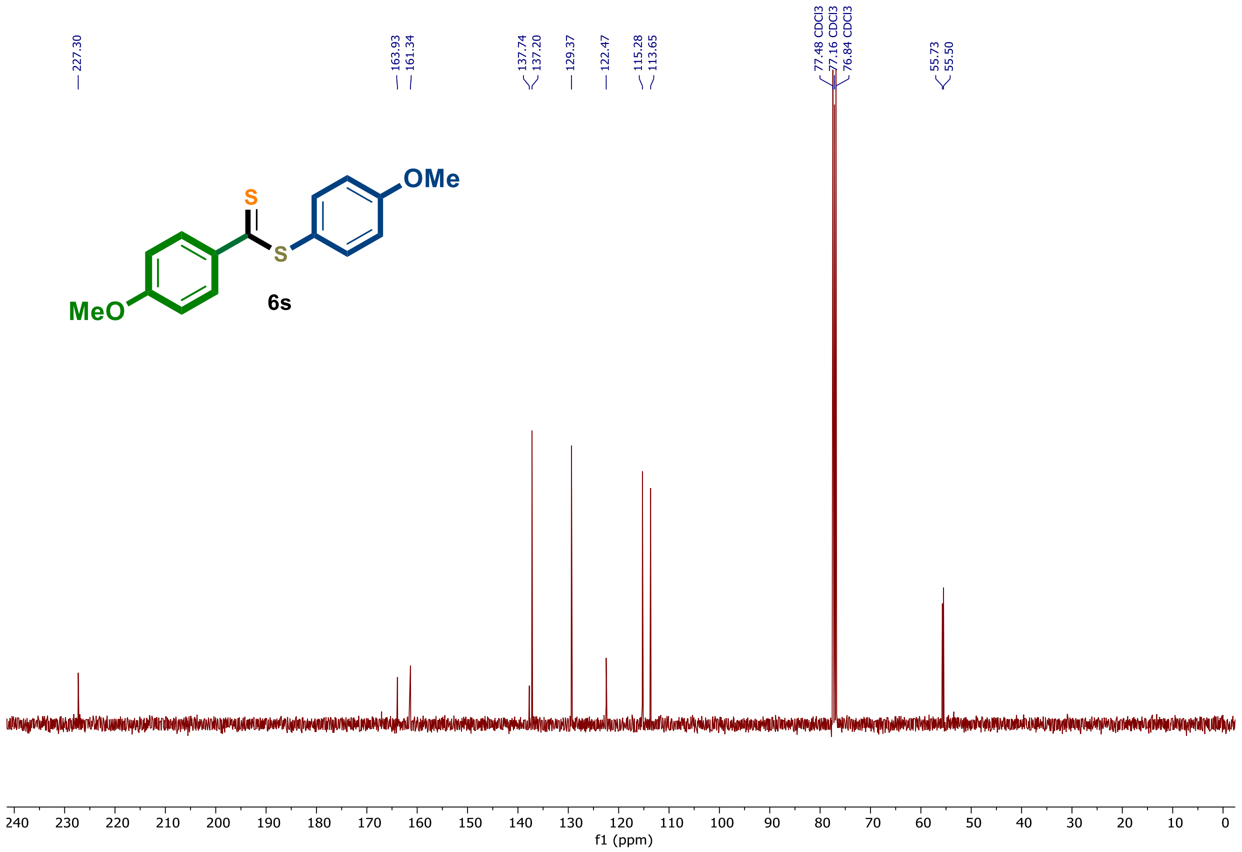

^{1}H NMR (400 MHz, $CDCl_3$) of **6t**

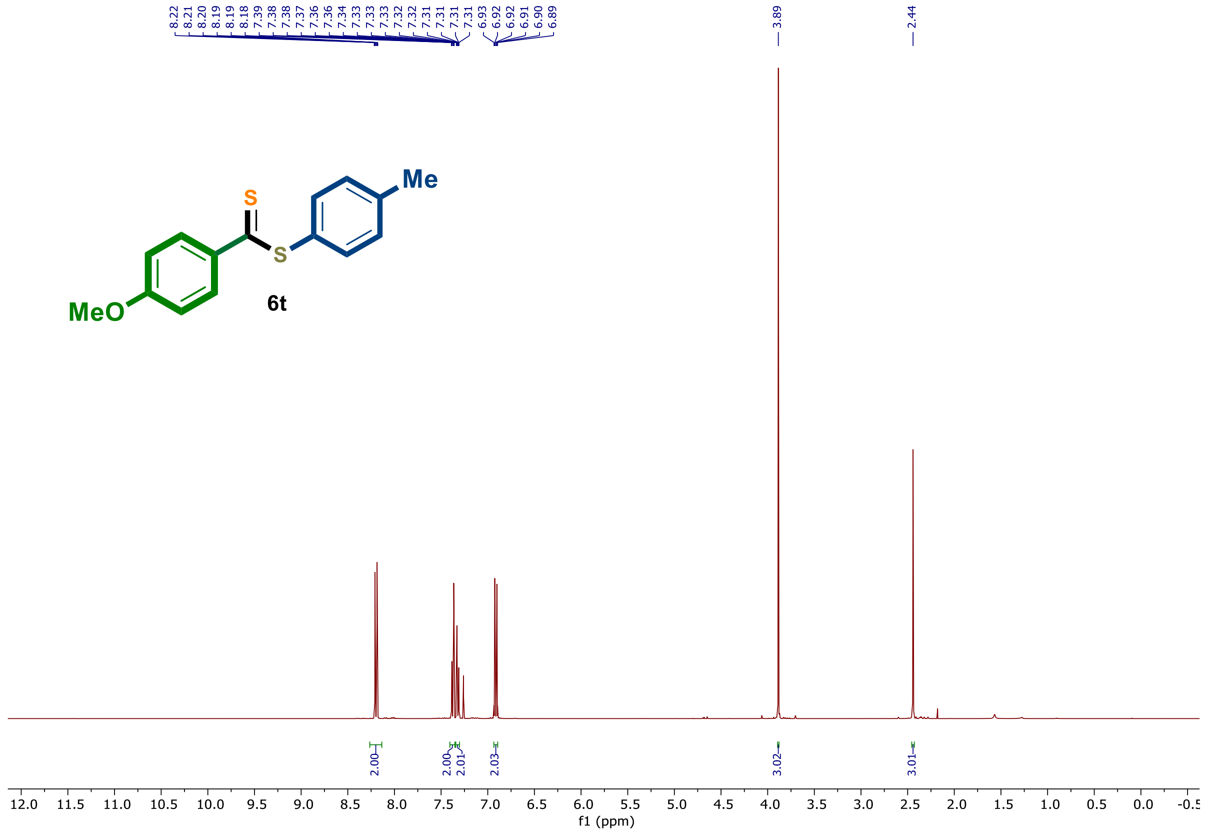

^{13}C NMR (100 MHz, CDCl$_3$) of **6t**

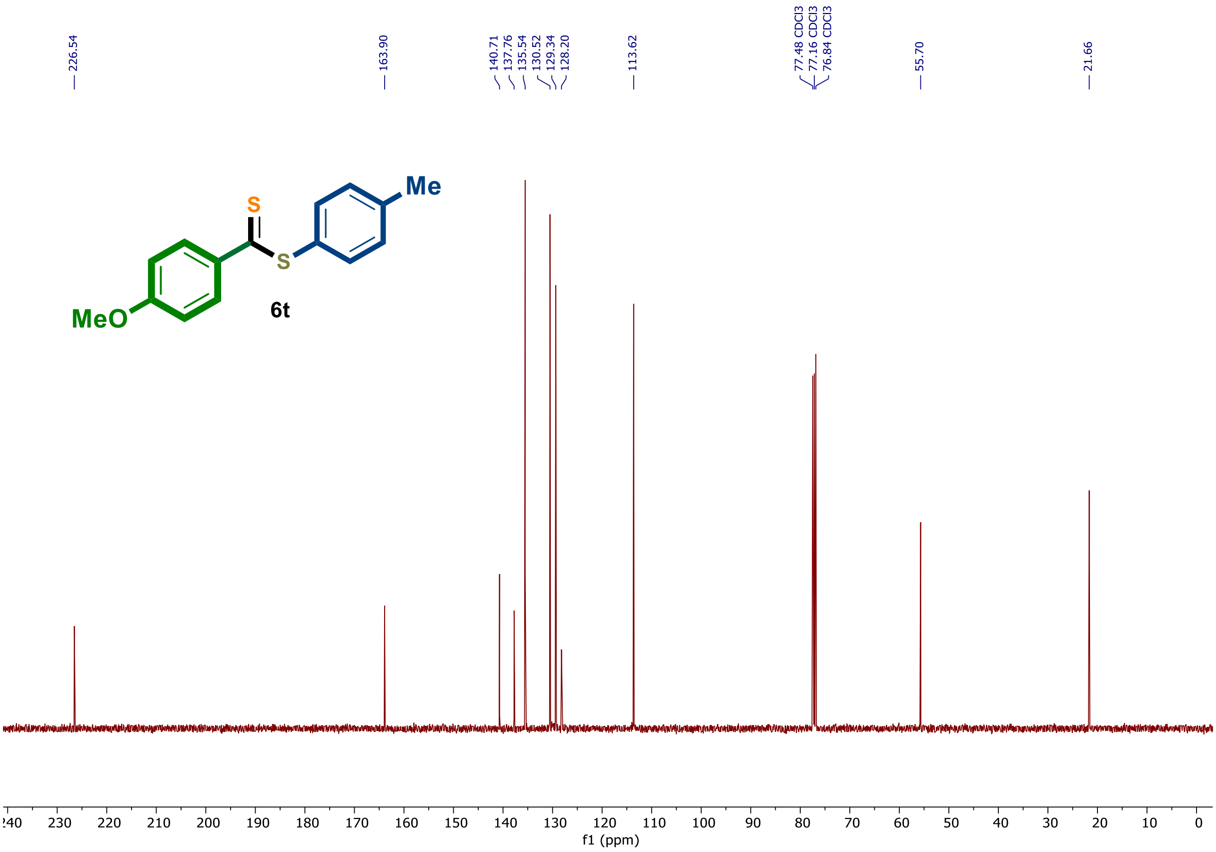

^{1}H NMR (400 MHz, CDCl$_3$) of **6u**

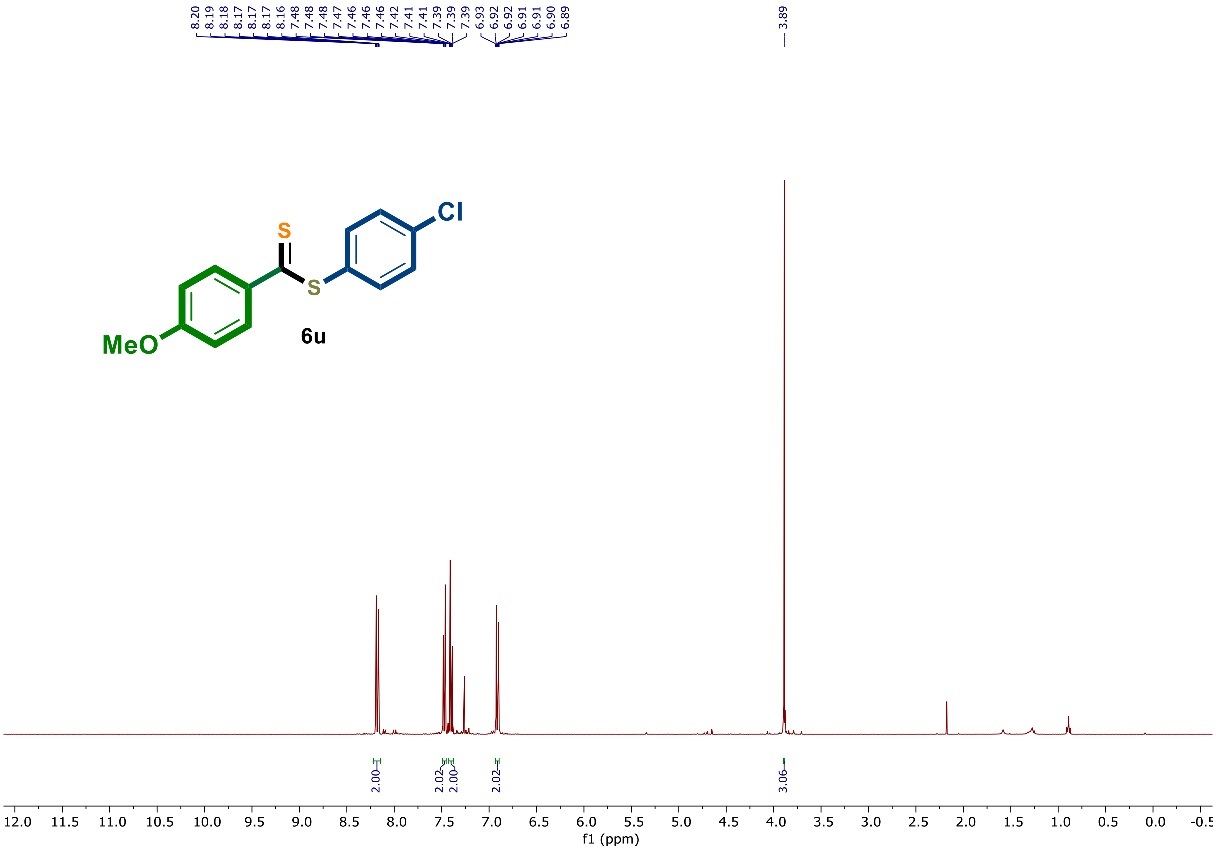

^{13}C NMR (100 MHz, $CDCl_3$) of **6u**

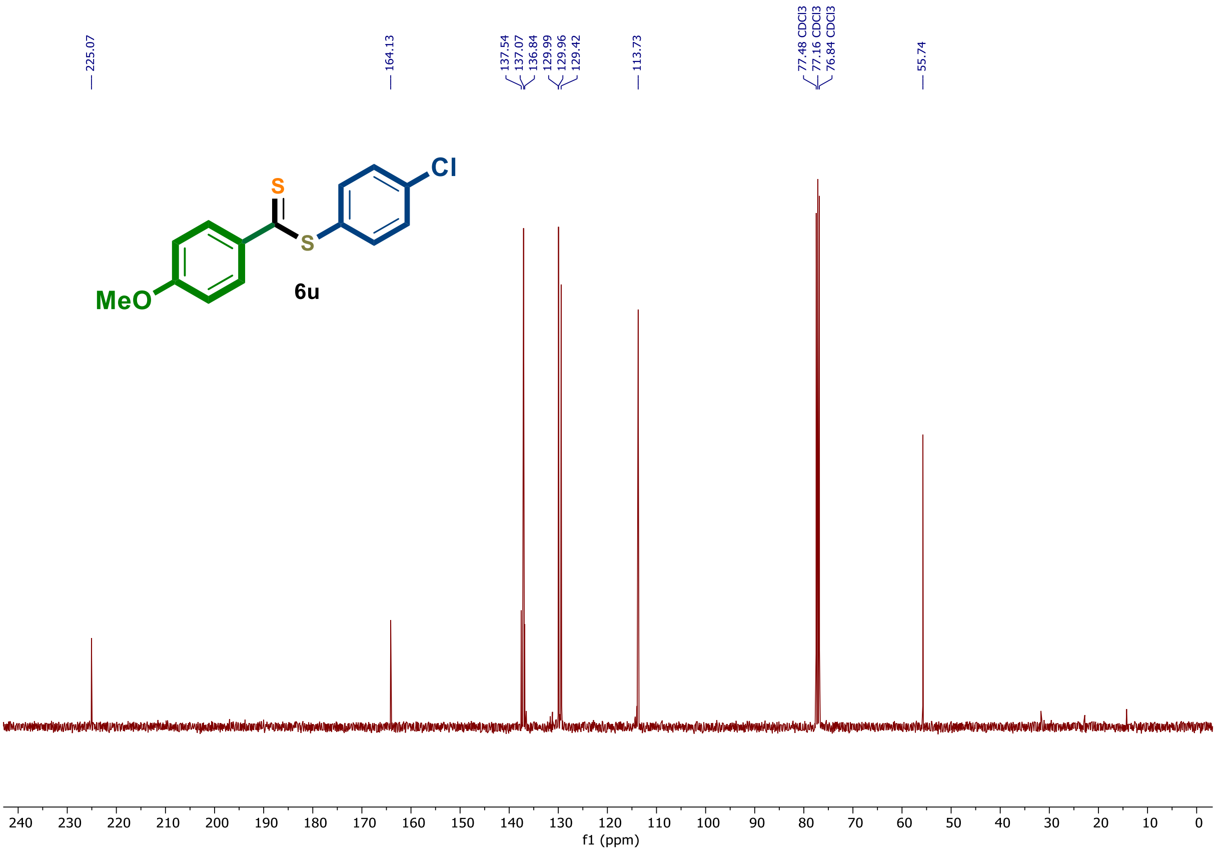

^{1}H NMR (400 MHz, $CDCl_3$) of **6v**

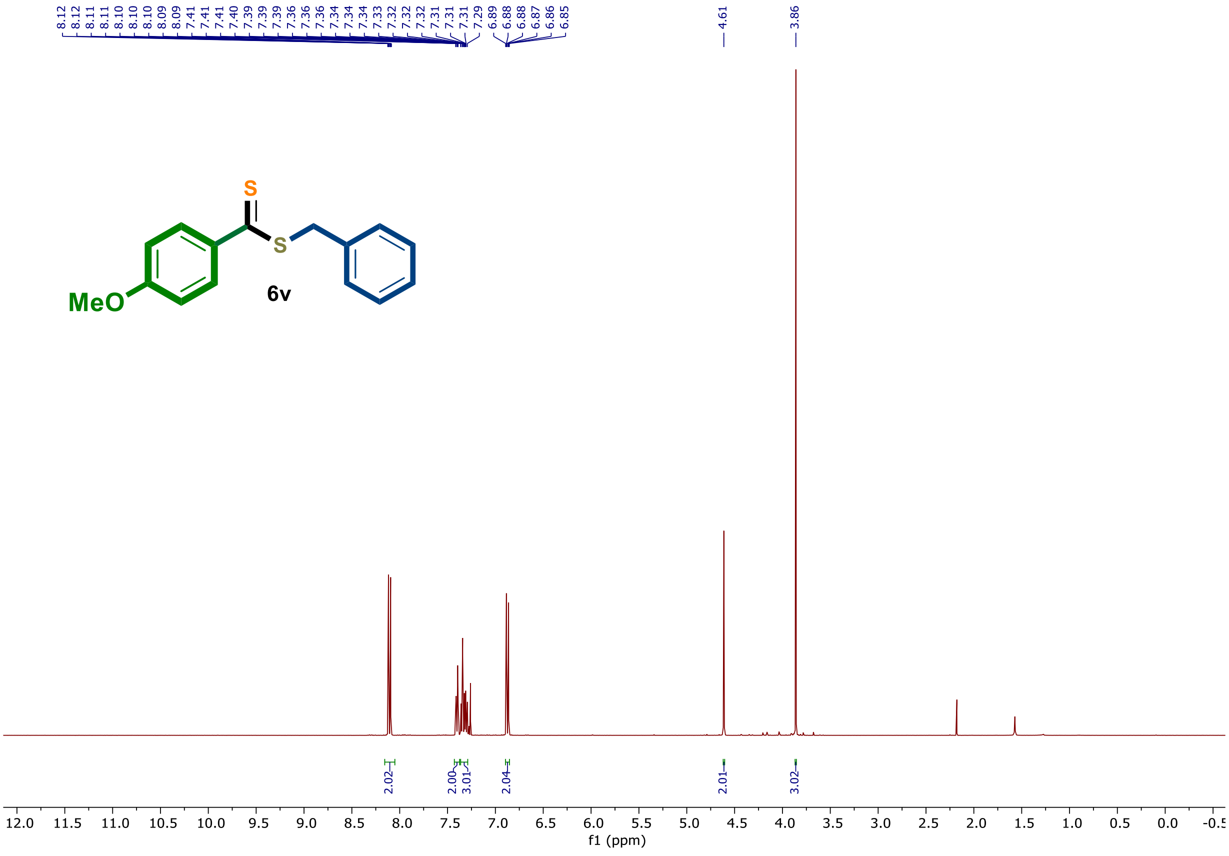

^{13}C NMR (100 MHz, $CDCl_3$) of **6v**

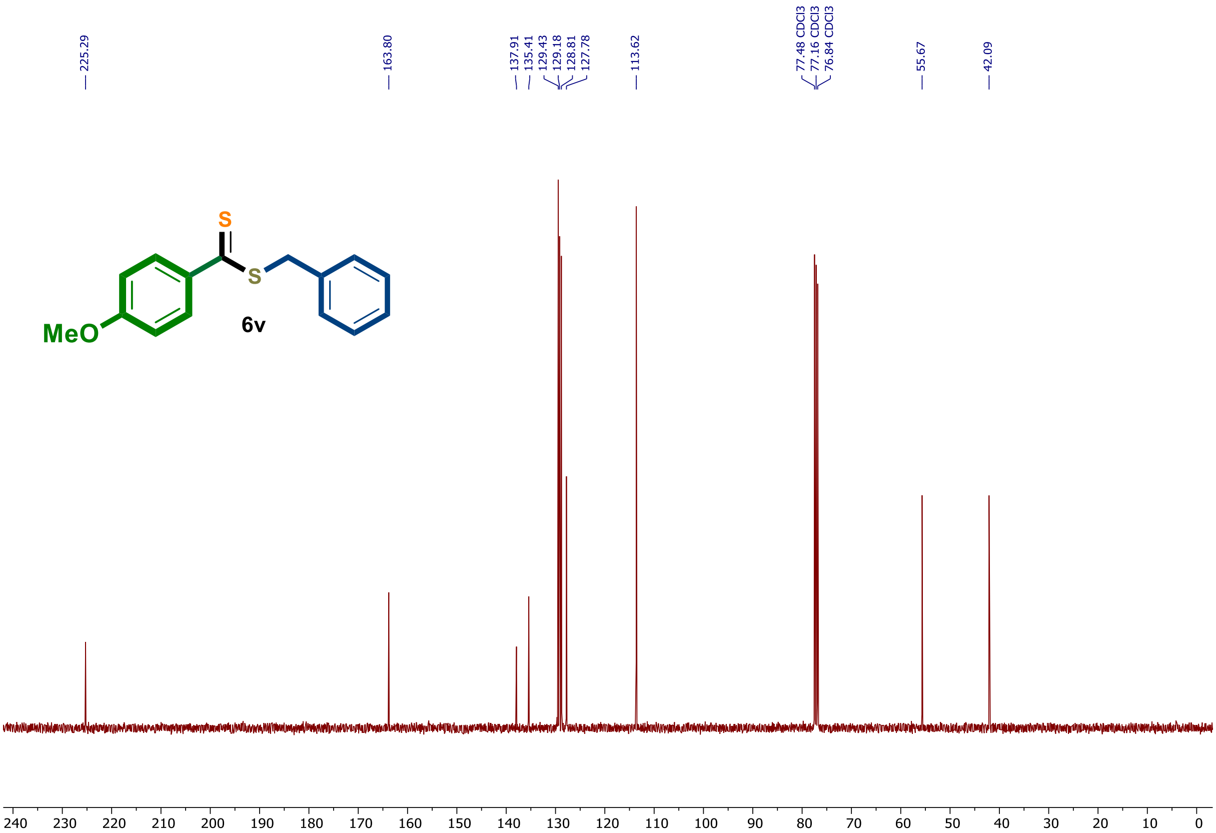

^{1}H NMR (400 MHz, $CDCl_3$) of **6x**

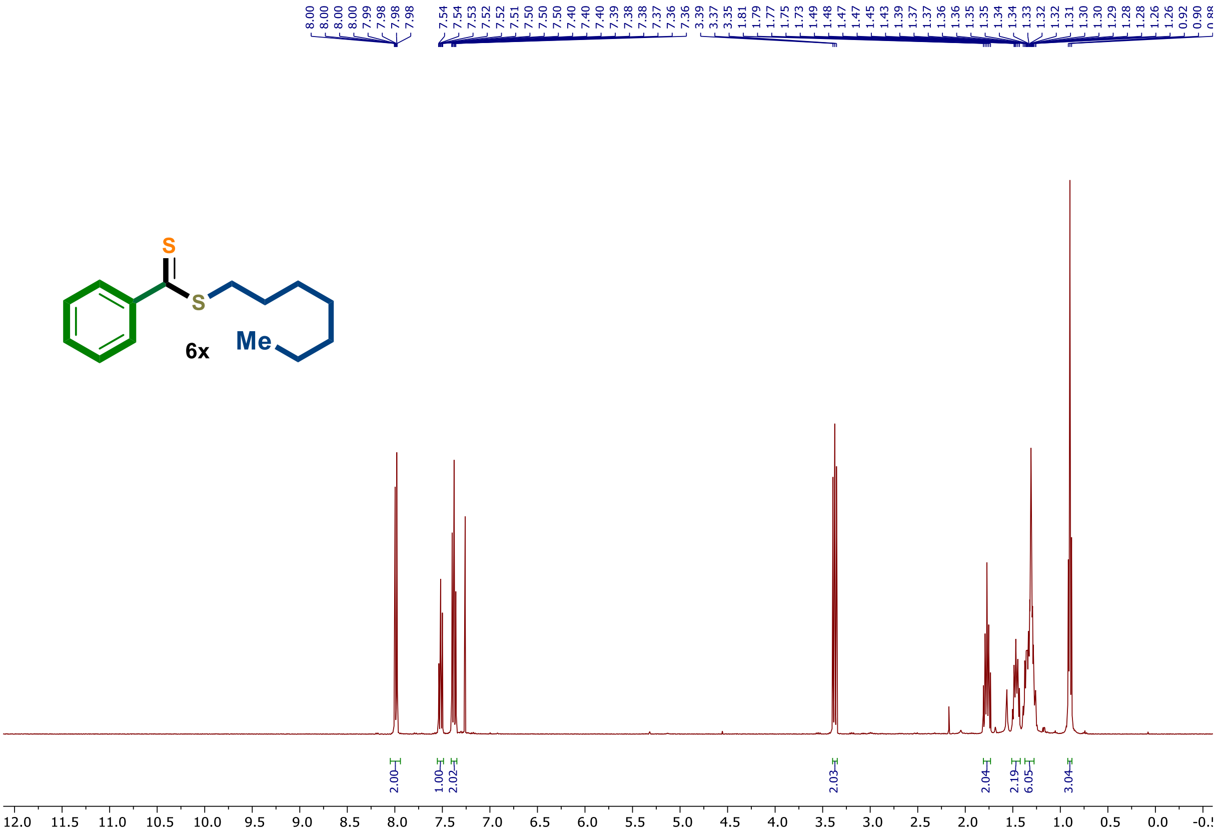

^{13}C NMR (100 MHz, $CDCl_3$) of **6x**

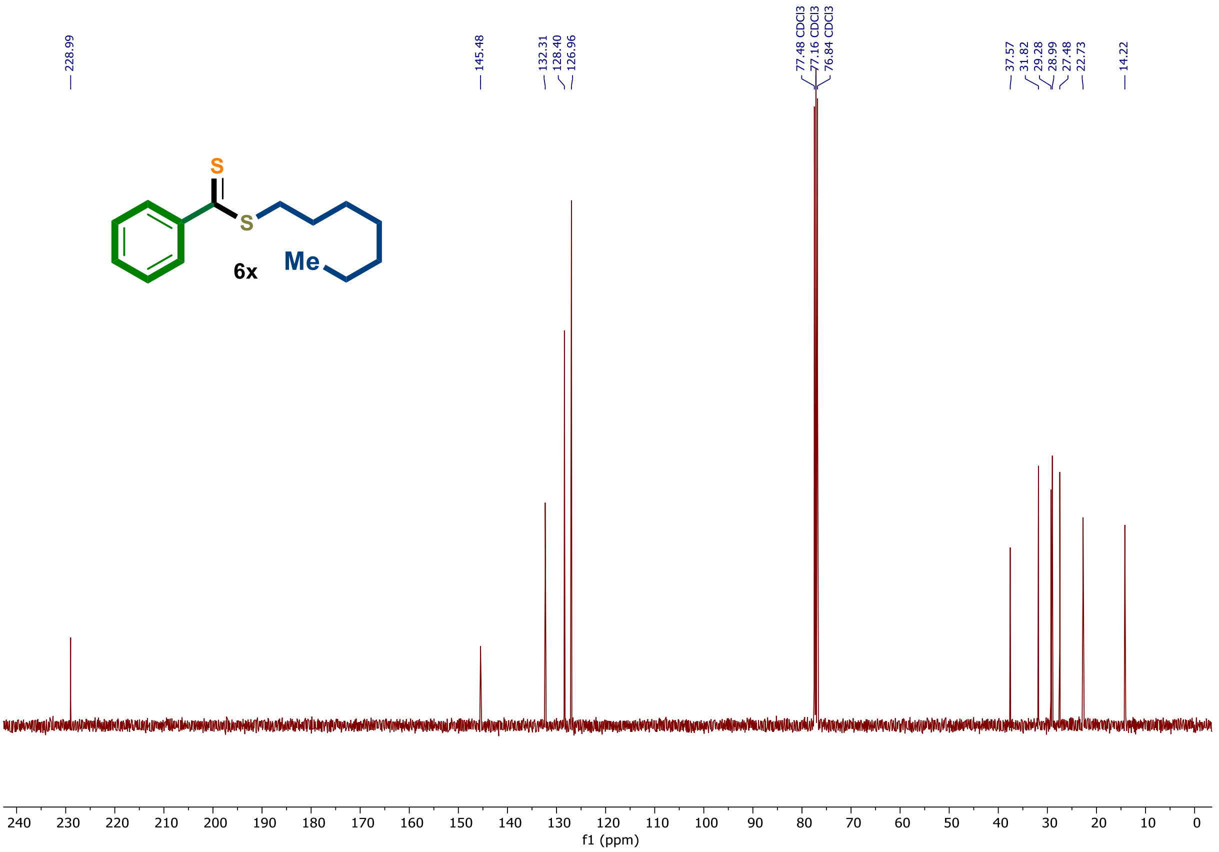

^{1}H NMR (400 MHz, $CDCl_3$) of **6y**

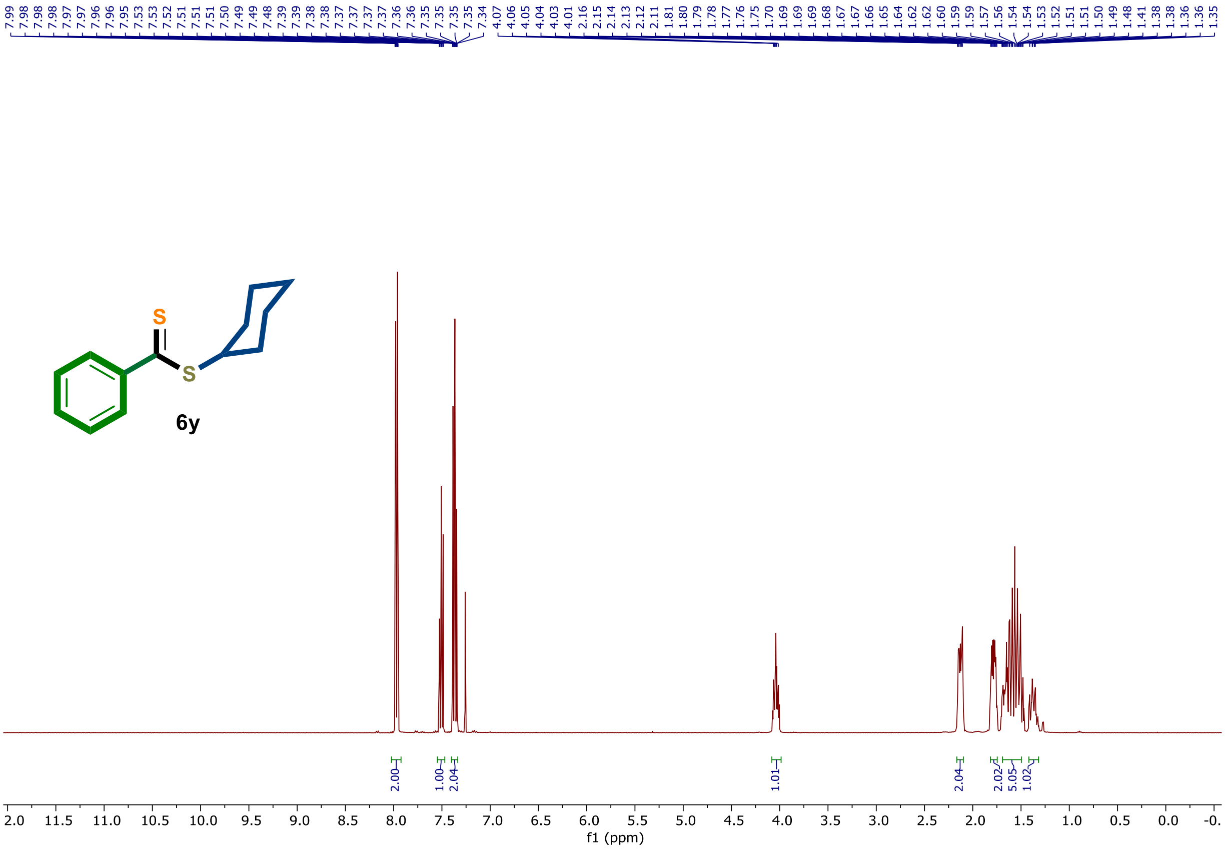

^{13}C NMR (100 MHz, $CDCl_3$) of **6y**

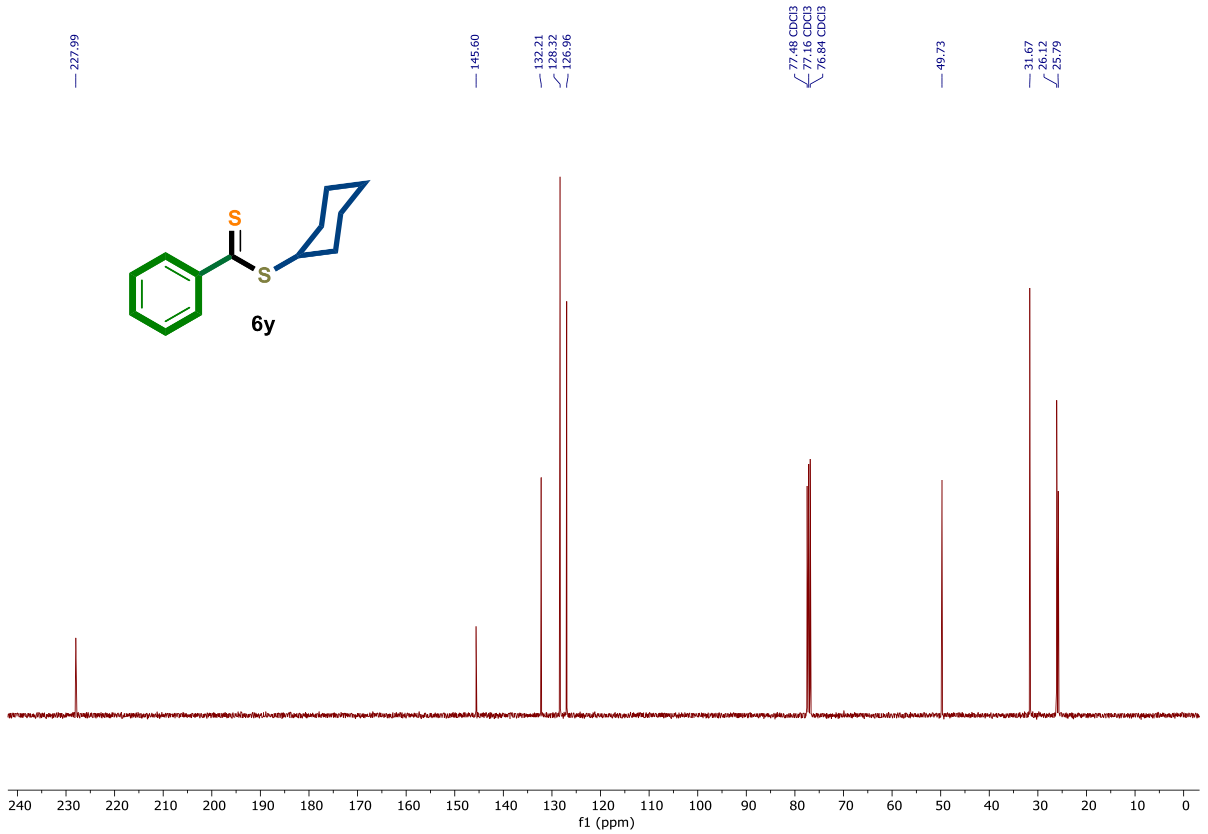

^{1}H NMR (400 MHz, $CDCl_3$) of **6aa**

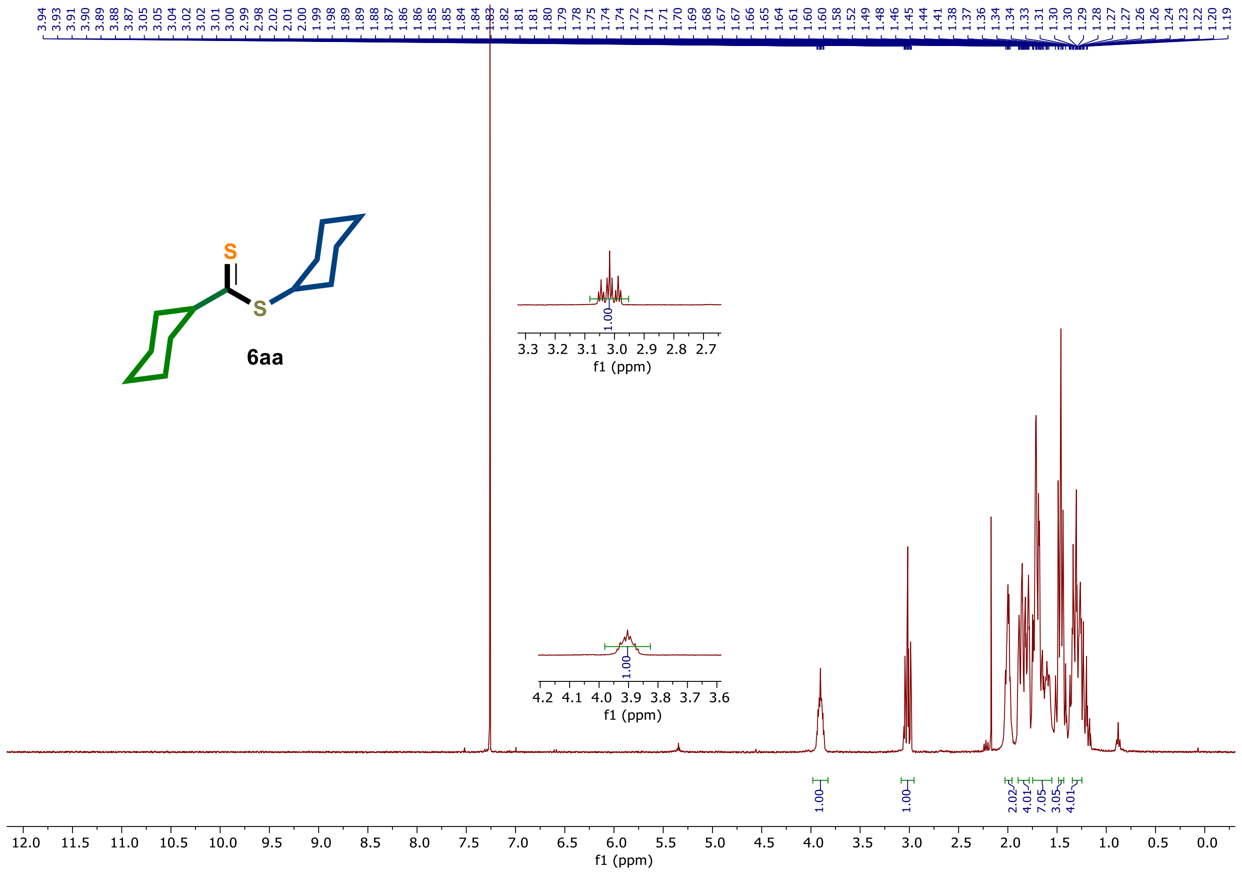

^{13}C NMR (100 MHz, $CDCl_3$) of **6aa**

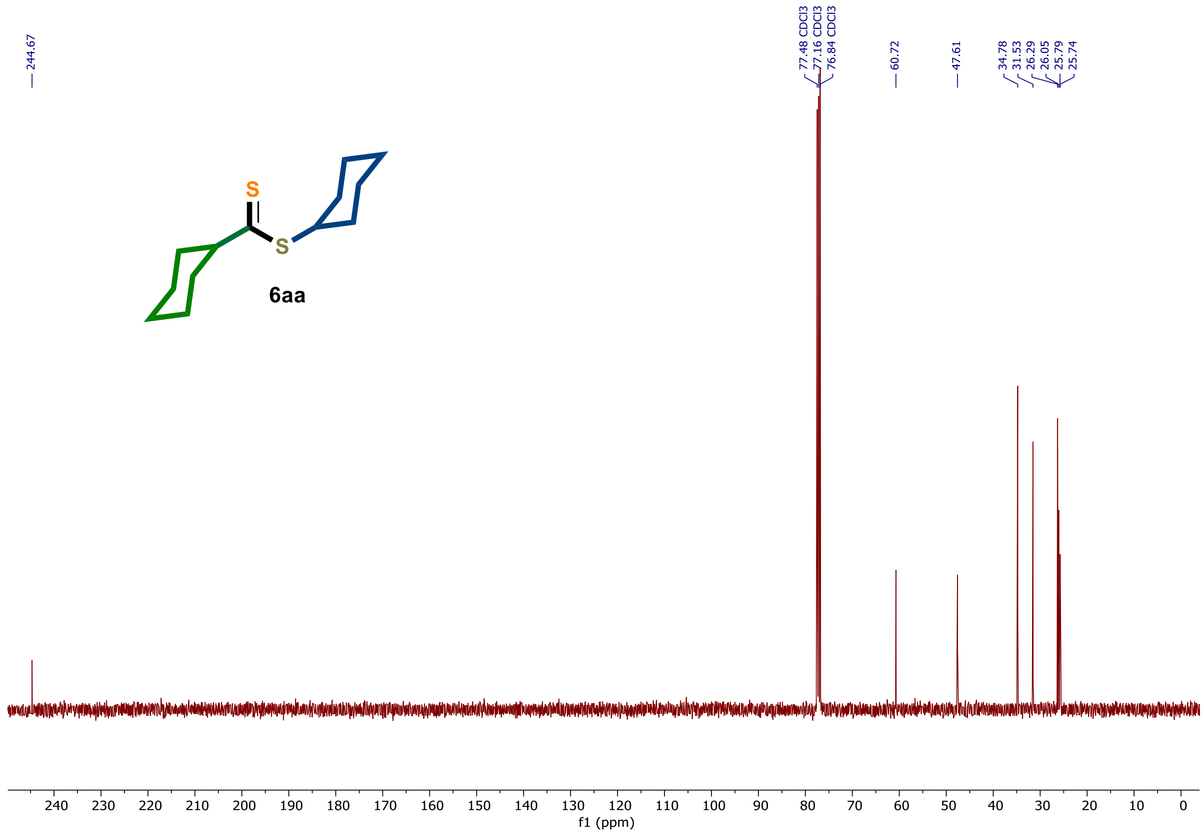

^{1}H NMR (400 MHz, $CDCl_3$) of **6ab**

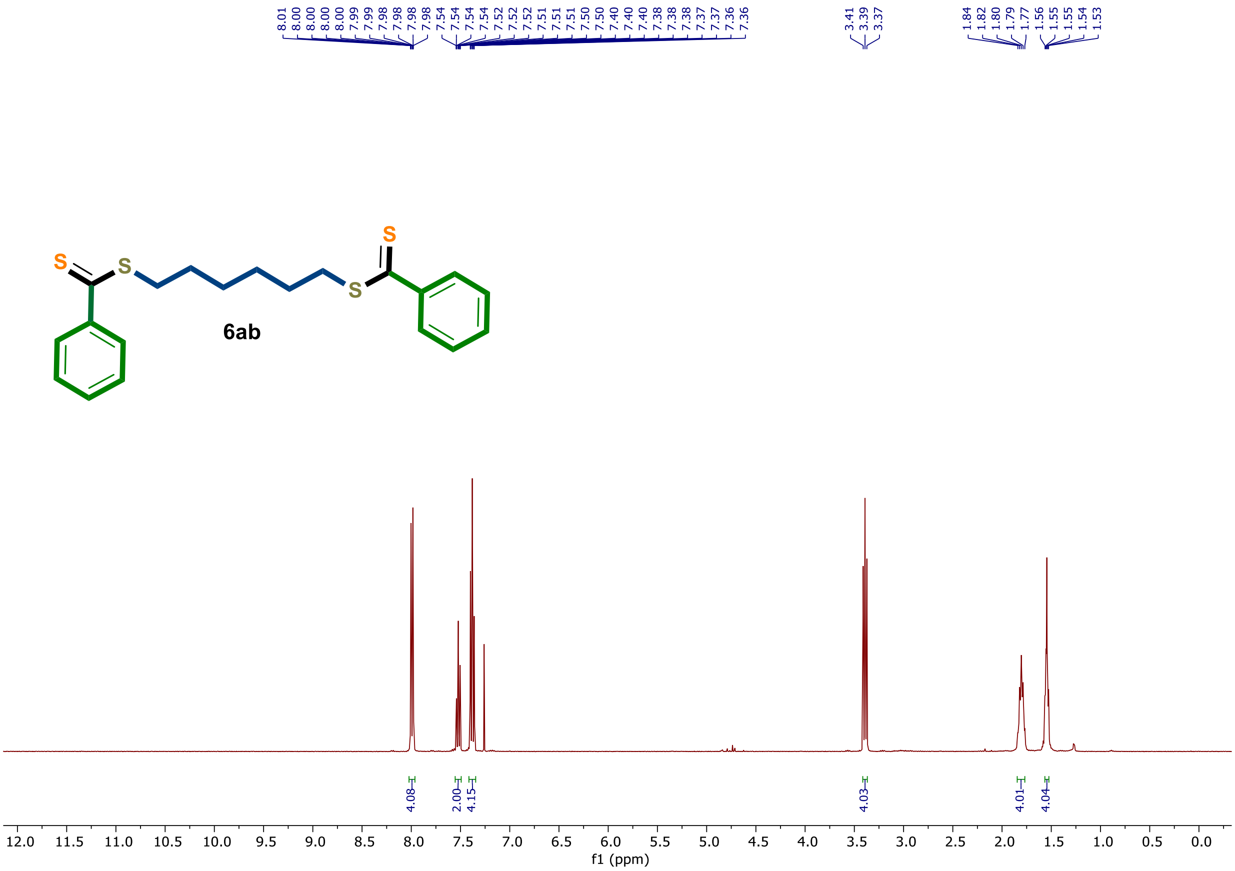

^{13}C NMR (100 MHz, CDCl$_3$) of **6ab**

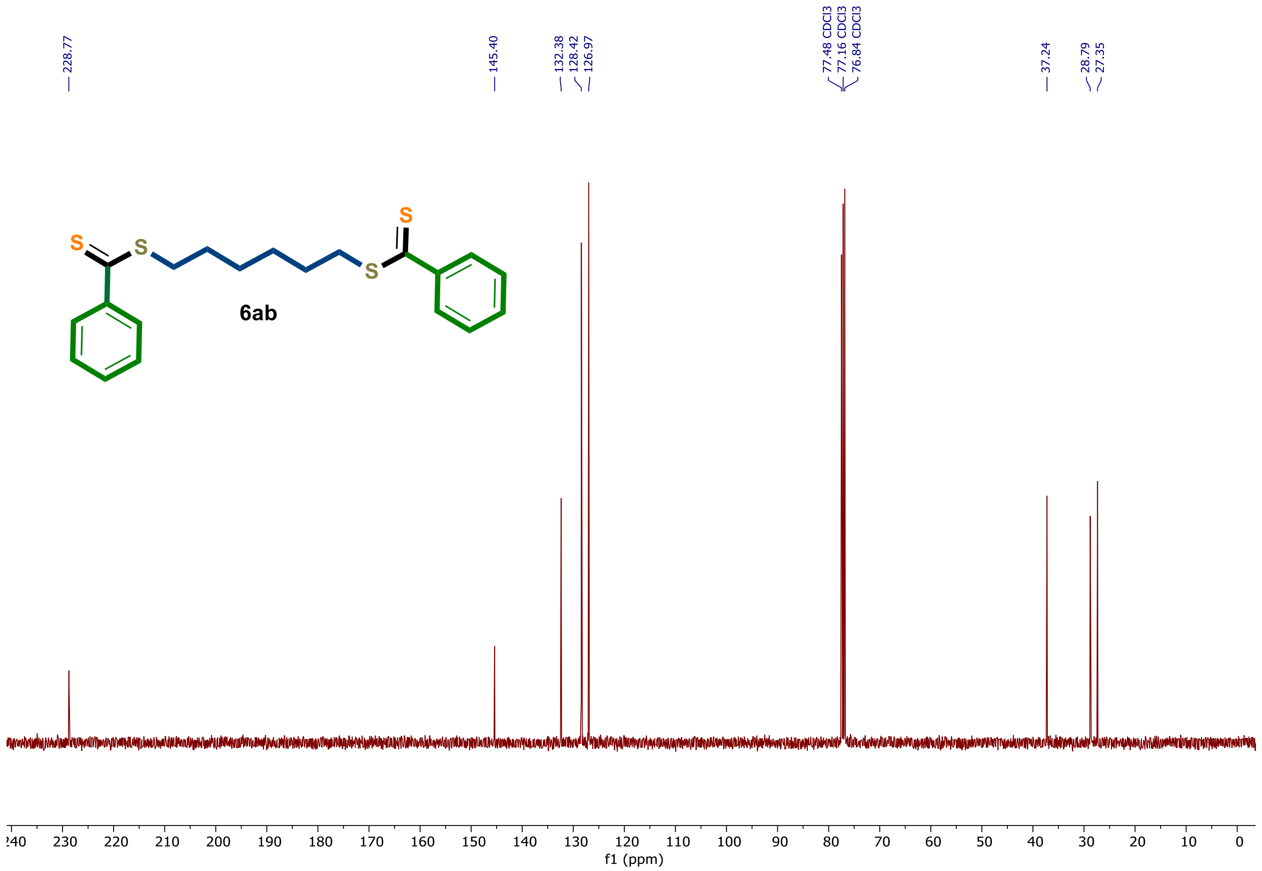

Supplement: Supplementary file 1 — ol3c02422_si_001.pdf [file ol3c02422_si_001.pdf]
